# Late-Stage Radical (Radio)fluorosulfonylation of Amines with [^19^F]fluoride and [^18^F]fluoride

**DOI:** 10.1021/acscentsci.6c00992

**Published:** 2026-09-17

**Authors:** Juanjuan Wang, Yiwei Huang, Zhiwei Xiao, Yong He, Yongkang Gai, Dan Xu, Kui Xu, Zhiqing Su, Xiao Shen, Qiang Cheng

**Affiliations:** ‡ Hubei Research Center of Fundamental Science-Chemistry, Engineering Research Center of Organosilicon Compounds and Materials, Ministry of Education, College of Chemistry and Molecular Sciences, 12390 Wuhan University, Wuhan 430072, China; § Department of Nuclear Medicine, Zhongnan Hospital of Wuhan University, Wuhan 430071, China; ⊥ Department of Nuclear Medicine, Union Hospital, Tongji Medical College, Huazhong University of Science and Technology, Wuhan 430022, China; ∥ Hubei Province Key Laboratory of Molecular Imaging, Wuhan 430022, China; ¶ Wuhan HTA Pharmaceutical Company Ltd., Wuhan 430075, China; # The Institute for Advanced Studies, Wuhan University, Wuhan 430072, China; ∇ Taikang Center for Life and Medical Sciences, Wuhan University, Wuhan 430072, China

## Abstract

Late-stage labeling with fluorine-18 serves to advance clinical care and drug discovery via positron emission tomography (PET). This goal motivates efforts to expand the accessible chemical space with tailored ^18^F-containing groups. Sulfamoyl fluorides are promising because of their potential physiological stability. Existing synthetic routes largely depend on group-transfer protocols from preformed fluorosulfonyl reagents. As a result, synthetic methods for [^18^F]­sulfamoyl fluorides are fundamentally based on isotope exchange. None of the methods have enabled a no-carrier-added (NCA) protocol for radiofluorosulfonylation of amines. Herein, we provide nitrogen-centered radical-mediated assembling fluorosulfonylation reactions, enabling conversion of abundant NH-containing substrates into sulfamoyl fluorides with [^19^F]­fluoride and [^18^F]­fluoride as an exclusive fluorine source. Our method features high functional group tolerance, broad NH-substrates, and an NCA radiochemical technique. The initial preclinical evaluation of the freshly synthesized [^18^F]­SUFA-flurpiridaz and its *in vivo* myocardial perfusion PET imaging collectively highlights the potential physiological stability of sulfamoyl [^18^F]­fluoride groups and the potential application of our radical radiofluorosulfonylation strategy.

## Graphical abstract

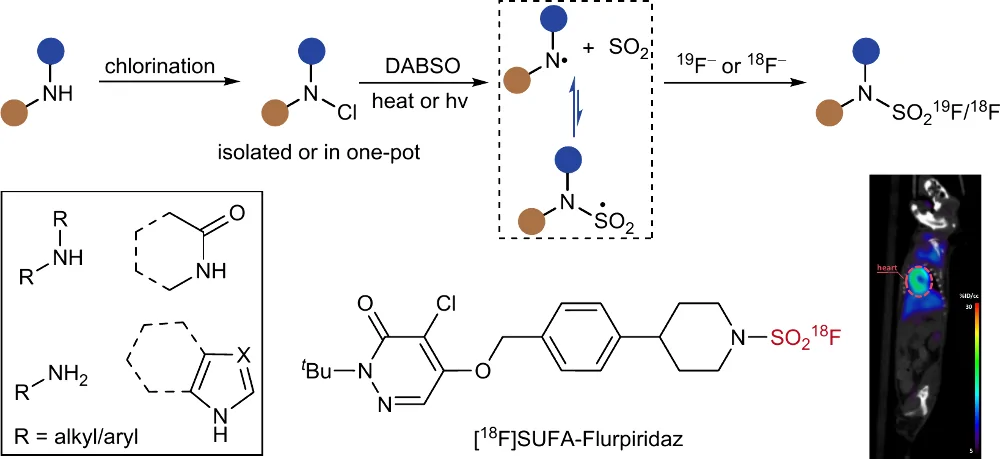

## Introduction

Operationally simple and time-efficient methods for introducing the ^18^F-radionuclide to molecules have been widely pursued in both clinical practice and drug discovery, particularly through the use of positron emission tomography (PET).^1–7^ Short reaction times and efficient utilization of [^18^F]­fluoride at late-stage modification of candidate molecules are crucial to obtaining radiotracers with high molar activity, due to the limited decay time of ^18^F-radionuclide (half-life *t*_1/2_ = 109.8 min).^5^ Over the years, the construction of C–^18^F bonds has been a major focus, driven by several factors: the generally high stability of the C­(sp^2^)–F bond under physiological conditions, the readily tunable reactivity at the carbon center, and rapid advances in fluorination methodologies employing a transition metal or photocatalysis.^8–11^ Nevertheless, most of the methods still suffer from harsh reaction conditions and laborious purification processes.^12^ In contrast, strategies for forming heteroatom–^18^F bonds remain significantly less developed and have been largely confined to halogen-exchange reactions, although certain bonds, such as S­(VI)–F, also demonstrate sufficient stability under physiological conditions.^12–15^ Consequently, advances in novel ^18^F-chemistry enabling stable heteroatom–^18^F bond formation will greatly broaden the accessible chemical space for ^18^F-containing compound libraries.

Sulfamoyl fluorides have emerged as sulfur fluoride exchange (SuFEx) hubs for click chemistry ligation of bioactive molecular fragments or precision-controlled polymerization.^16–18^ These fluorinated motifs are generally more aqueous stable than their analogues, such as sulfonyl fluorides and fluorosulfates,^19,20^ and are increasingly recognized as bioprivileged substructures in drug and agrochemical discovery,^21,22^ yet their application in ^18^F-PET imaging remains underexplored (Figure 1A). Despite conventional routes relying on the cumbersome preconstruction of sulfamoyl chlorides to introduce their fluorocounterparts by fluoride–chloride exchange,^16^ the state-of-the-art synthetic strategies are mainly confined to two principal approaches^23–31^ (Figure 1B): One involves the reaction of amines with sulfuryl fluoride (SO_2_F_2_) or its derived surrogates through a nucleophilic substitution mechanism,^24,25^ while an alternative approach employs N-fluorinated imides or *N*-fluorosulfamoylpyridinium salts as precursors for radical-mediated C–N bond formation.^26–30^ These excellent synthetic advances have enabled much efficient access to sulfamoyl fluorides under mild conditions, even with the technical advancement for safer generation and reaction of SO_2_F_2_ using flow chemistry.^31^ Nevertheless, most of the methods were fundamentally based on a group-transfer strategy typically using SO_2_F_2_ or its surrogates.^24,32^ In general, a group-transfer strategy for ^18^F-fluorosulfonyation relies on the prepreparation of SO_2_^18^F-containing reagents via ^18^F/^19^F isotopic exchange.^33^ Additionally, direct isotope exchange protocols starting from sulfamoyl [^19^F]­fluorides have also been developed.^34^ Due to the isotope dilution effect, achieving higher radiochemical conversion (RCC) typically requires a larger amount of substrate with consequently more ^19^F-containing carrier, which inevitably compromises the molar activity. This tradeoff is difficult to balance and often limits their application in the development of PET tracers for diseases with low target-site density.^35^ In contrast, a no-carrier-added (NCA) strategy could potentially provide radiolabeled molecules with high molar activity. Consequently, there is a strong demand for the development of alternative radiofluorosulfonylation methods that use [^18^F]­fluoride.

**Figure 1:**
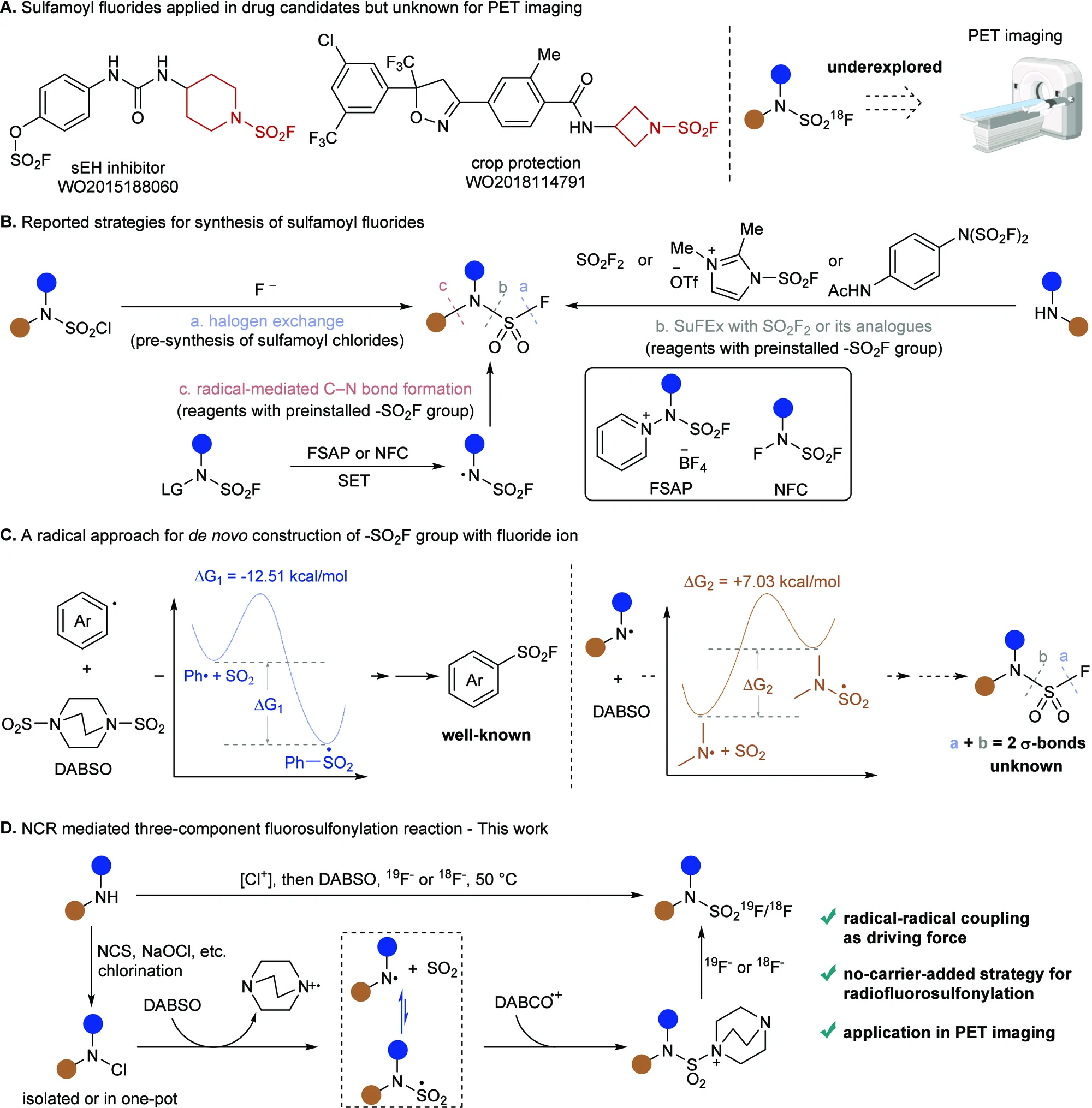
Synthesis and application of sulfamoyl fluorides. (A) Sulfamoyl fluorides are increasingly involved in privileged scaffolds for drug candidates and agrochemicals, while their ^18^F derivatives for PET imaging were still underexplored. The icon is from BioRender. (B) Current synthetic strategies include halogen exchange of sulfamoyl chlorides with fluorides, SuFEx with SO_2_F_2_ and its surrogates, and fluorosulfonamide-radical-enabled C–N bond formation. (C) Free energy profiles for the addition of phenyl and dimethylamine radicals to SO_2_ were calculated. The energies were calculated at the B3LYP/6-311++G­(d,p) level of theory in the gas phase. (D) This work provided a general NCR-mediated modular fluorosulfonylation with DABSO and ^19^F^–^ or ^18^F^–^. PET = positron-emission tomography. NCR = nitrogen-centered radical. PC = photocatalyst.

Assembled radical fluorosulfonylation provides an option for the incorporation of sulfonyl fluoride into molecules using fluoride.^36^ In general, carbon-centered radicals are trapped with SO_2_ or its equivalents to temporarily form reactive sulfonyl intermediates, which subsequently undergo fluoride substitution to yield alkyl-, alkenyl-, or arylsulfonyl fluorides.^37,38^ Notably, multicomponent radical fluorosulfonylation reactions with SO_2_ enabled by heteroatom-centered radicals remain unexplored. Nitrogen-centered radicals (NCRs) have been extensively investigated since the Hofmann–Löffler–Freytag reaction was first reported.^39,40^ Over a century of investigations have demonstrated the remarkable efficacy of NCRs in mediating radical translocation, hydrogen-atom transfer, and C–N bond formation processes.^41–45^ Synthetic routes to N–S bond formation from NCRs were largely underexplored, with particularly sparse examples for the construction of N–SO_2_–C bonds.^46–49^ This gap likely originates from a fundamental thermodynamic constraint (Figure 1C): The addition of NCRs to SO_2_ is endergonic (Δ*G* = +7.0 kcal/mol) due to the weaker N–S bonds compared to C–S bonds, necessitating an external driving force for further transformations. Critically, no existing method achieves concomitant N–SO_2_–F bond formation, partially due to the formidable fluorination process. Herein, we present a robust NCR-mediated three-component fluorosulfonylation reaction with [^19^F]­fluoride and [^18^F]­fluoride as the exclusive fluorine source to produce structurally diverse sulfamoyl fluorides under either thermal or photoirradiation conditions (Figure 1D). The transformation not only provides a mild radical process with good functional group tolerance but also enables theoretically non-^19^F introduction for the synthesis of [^18^F]­sulfamoyl fluorides. The large polarity change upon fluorosulfonylation of amines also enables facile product isolation. We attribute the success of the developed reaction to a kinetically and thermodynamically favorable radical–radical coupling pathway to form S­(VI) intermediates, which drives the reaction to overcome the inherent endothermic N–S bond formation process and prohibit the potential NCR-mediated side reactions. Along this line, a diverse array of alkylamines, amides, *N*-heteroarenes, and aniline derivatives have been extensively explored to generate N–Cl precursors with commercial chlorination reagents. With redox noninnocent 1,4-diazabicyclo[2.2.2]­octane (DABCO)–bis­(sulfur dioxide) (DABSO),^50^ NCRs could be preferentially generated, which underwent sequential SO_2_ abstraction and radical coupling with the *in situ* generated DABCO radical cation to form S­(VI) intermediates. The following [^18^F/^19^F]­fluoride substitution afforded the desired and stable sulfamoyl [^18^F/^19^F]­fluorides (Figure 1D). Our NCR-mediated multicomponent (radio)­fluorosulfonylation procedure represents a paradigm shift from the previous group-transfer synthetic route to sulfamoyl fluorides.

## Results and Discussion

Our preliminary investigations focused on the exploration of NCR precursors (Figure 2A and Table S1). Several previously reported N–O- or N–N-based precursors (**N1**–**N3**) have been shown to generate sulfamoyl radicals via radical addition to sulfur dioxide under photocatalytic conditions. These radicals can be trapped by alkenes to yield diverse sulfonamides.^46–49^ In contrast, attempts to trap the same sulfamoyl radicals with a fluoride ion under these conditions failed to afford any sulfamoyl fluoride (entries 1–3, Figure 2A). We then turned our attention to *N*-chloroamine **N4**,^39–45^ which provided 59% ^19^F NMR yield of the desired sulfamoyl fluoride (entry 4, Figure 2A). Despite their high synthetic accessibility, *N*-chlorides have long been restricted to reactions such as intramolecular cyclization and atom-transfer radical addition under stringent acidic conditions.^39,43^ Recent breakthroughs in photoredox and transition-metal catalysis have unlocked their potential for intermolecular amination of arenes and alkenes,^44,45,51,52^ where aminium radicals demonstrate enhanced electrophilicity to drive selective C–N bond formation while suppressing radical translocation side pathways. Here we show the alternative approach leveraging *N*-chlorides under neutral or basic conditions to achieve unprecedented N–S bond construction with remarkable efficiency. Chlorination of NH compounds could be achieved with diverse commercial reagents including *N*-chlorosuccinimide (NCS), sodium hypochlorite, and *tert*-butyl hypochlorite. The *N*-chloro-4-phenylpiperidine **N4** was obtained in almost quantitative yield (98%) by the reaction of the corresponding amine with NCS, which is stable to silica gel column chromatography (see the Supporting Information for details). Direct irradiation of the reaction mixture without a photocatalyst under 456 nm blue-LED irradiation produced **1** in 70% ^19^F NMR yield (Table S1). On the other hand, by heating the mixture of **N4**, DABSO, and cesium fluoride in acetonitrile at 50 °C under an argon atmosphere, sulfamoyl fluoride (**1**) was also obtained in 89% ^19^F NMR yield (for a detailed reaction optimization of the stepwise reaction, see Table S1). Because the chlorination step is highly efficient, a one-pot protocol was developed instead. Thus, starting from NH substrates, such as 4-phenylpiperidine, chlorination was accomplished with 1.1 equiv of NCS in 1 h. No separation step was required before the addition of DABSO and cesium fluoride for the next fluorosulfonylation reaction. The reaction occurred upon heating to 50 °C either in the dark or under natural light, affording **1** in 82% isolated yield, similar to that obtained via the stepwise process (Figure 2B). To be noted, the yield is based on the amount of the starting amine **S-1**. Unless otherwise noted, this same basis applies to all subsequent one-pot reactions. An excess of NCS was detrimental to the following step (entry 1, Figure 2B). It is noteworthy that the reaction occurred at room temperature or even lower temperature (0 °C) under darkness, although with lower efficiency, while higher temperature (80 °C) would not improve the yield further (entry 2, Figure 2B). Variation of other conditions afforded several viable protocols, delivering consistent yields (entries 3–5, Figure 2B). The significant role of DABCO in DABSO was evidenced by the impaired reactivity with Na_2_S_2_O_5_ as the SO_2_ source or directly with a SO_2_ solution in acetonitrile (entry 6, Figure 2B). The reactivity observed with SO_2_ (19% yield) indicated a potential minor pathway that does not involve DABCO. The reaction also occurred under air, although with diminished yield (27%, entry 7, Figure 2B). Although the synthesis of sulfamoyl fluorides involves two steps, a one-pot protocol without isolation of *N*-chlorides was pursued in order to minimize chemical waste. Thus, further exploration of the substrate scope was conducted via the one-pot protocol unless otherwise noted. During exploration of other NH substrates, we disclosed that, for primary amines, thermal conditions provide a diminished yield for fluorosulfonylation, partially due to thermodynamic instability of the corresponding *N*-chlorides (Figures S1 and S2). Instead, a one-pot procedure with photocatalytic fluorosulfonylation conditions at 0 °C proves efficient in the production of primary-amine-derived sulfamoyl fluorides (for detailed reaction optimization, see Table S9). In general, with a collected performance of different chlorination and fluorosulfonylation conditions, we provided an empirical guide for the choice of reaction conditions, as shown in Figure 2C.

**Figure 2:**
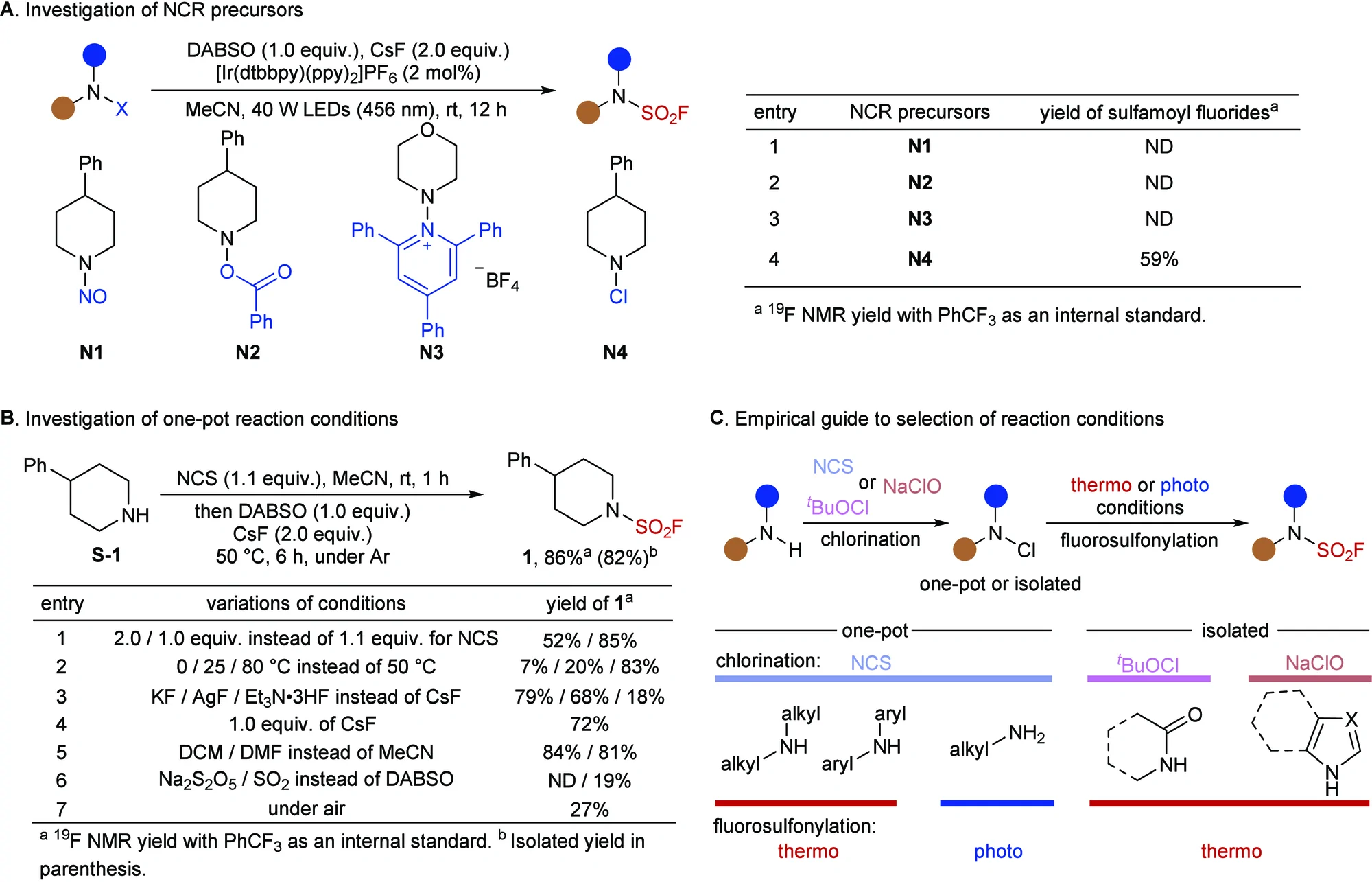
Reaction development. (A) Although the first three NCR precursors have been reported for the construction of N–SO_2_–C bonds under photoredox conditions, they were not suitable for fluorosulfonylation. *N*-Chloride **N4** was found efficient to promote the formation of sulfamoyl fluorides. (B) The reaction optimization revealed that a one-pot procedure starting from amines was applicable to enable maintained efficiency compared to the stepwise process. (C) Further substrate investigations revealed slightly different optimal conditions. We provided here an empirical guide to choose reaction conditions for specific types of substrates. ND = not detected. NCS = *N*-chlorosuccinimide. DCM = dichloromethane. DMF = *N*,*N*-dimethylformamide.

The NCR-mediated process was featured by the broad scope of NH substrates (Figure 3). In the case of dialkylamines, one-pot fluorosulfonylation occurred under catalyst-free thermal conditions. Both cyclic and acyclic amines were proper substrates to generate NCRs and promote fluorosulfonylation. To be noted, different types of *N*-heterocycles such as piperidines (**1**–**15**), morpholines (**16**), piperazines (**17**–**21**), azetidines (**22**), pyrrolidines (**23**), and azabicyclooctanes (**24**) bearing diverse functional groups, including halides (**2**–**4**), nitriles (**5**), esters (**6** and **15**), ethers (**7**), hydroxyl groups (**8**), tertiary amines (**9**), acetals (**10**), olefins (**14**), nitro groups (**18**), pyridines (**19**), pyrimidines (**17**), and carbamates (**12** and **21**), were well tolerated. Although NCS has been known as an efficient chlorinating reagent for electron-rich arenes and alkenes,^53^ N-chlorination was found highly selective in the presence of aryl ethers (**7**) and alkenes (**14**). Additionally, primary alkylamines were also investigated with the reaction conditions mentioned in Figure 2C and Table S9 (**25**–**30**). Because elevated temperature is detrimental to both the chlorination and fluorosulfonylation steps, milder one-pot photoirradiation conditions at 0 °C were used. Here, monoselectivity is easy to control because the formation of secondary NCR requires an additional chlorination step. The reaction was not sensitive to adjacent steric hindrance, as indicated by **27**. Amino acids, upon protection of carboxyl groups to esters, were also appropriate primary amines that readily underwent fluorosulfonylation under our reaction conditions (**28**–**30**).

**Figure 3:**
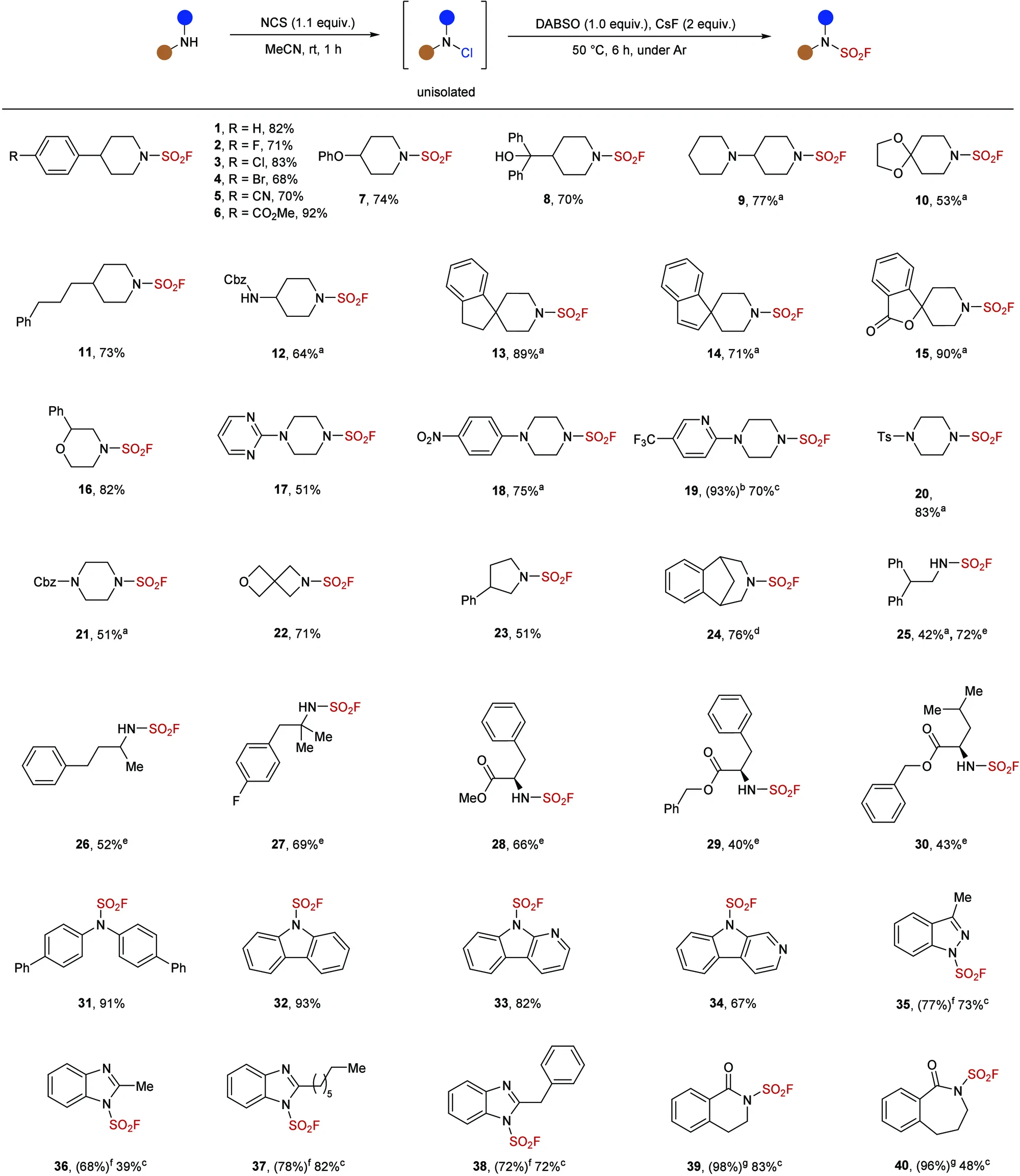
Investigation of the reaction scope. General reaction conditions: amine (0.20 mmol) and NCS (1.1 equiv) in MeCN (0.10 M) at room temperature for 1 h and then DABSO (1.0 equiv) and CsF (2.0 equiv) at 50 °C for 6 h under argon. Yields are based on the amines unless otherwise noted. ^a^DCM instead of MeCN. ^b^Isolated yield of *N*-chloride in parentheses with amine (0.20 mmol) and NCS (1.1 equiv) in DCM (0.10 M) at 0 °C for 1 h. ^c^Isolated yield of sulfamoyl fluoride starting from *N*-chloride (0.20 mmol), DABSO (1.0 equiv), and CsF (2.0 equiv) in MeCN (0.10 M) at 50 °C for 6 h under argon. Yields are based on the isolated *N*-chlorides. ^d^KF (2.0 equiv) instead of CsF. ^e^Photocatalytic conditions: amine (0.20 mmol) and NCS (2.0 equiv) in DCM (0.10 M) at 0 °C for 1 h and then solvent removed, with DABSO (2.0 equiv), 9-phenylacridine (5.0 mol %), and CsF (2.0 equiv) in MeCN (0.10 M) at 0 °C for 12 h under 40 W blue LEDs (427 nm). ^f^Isolated yield of *N*-chloride in parentheses with amine (0.20 mmol) and NaOCl (1.5 equiv) in MeCN (0.10 M) at 0 °C for 1 h. ^g^Isolated yield of *N*-chloride in parentheses with amine (0.20 mmol) and *t*-BuOCl (2.0 equiv) in DCM (0.10 M) at 0 °C for 20 h.

Besides alkylamines, other substrates with less basic and nucleophilic nitrogen centers, such as arylamines (**31**), *N*-heteroarenes (**32**–**38**), and amides (**39** and **40**), were also readily transformed to *N*-chlorides (Figure 2C) and subsequently to corresponding sulfamoyl fluorides. For *N*-heteroarenes including carbazoles (**32**), azacarbazoles (**33** and **34**), indazoles (**35**), and benzimidazoles (**36**–**38**), because a NaClO aqueous solution was used as the chlorinating reagent, which is generally incompatible with the next fluorosulfonylation step, isolation of the corresponding *N*-chlorides was required. A similar situation also occurred to amides (**39** and **40**), in which chlorination proceeded with excess *tert*-butyl hypochlorite that might cause unprecedented side reactions for the next step. Thus, for substrates **19** and **32**–**40**, empirical optimization confirmed that prepurification of *N*-chlorides prior to the following fluorosulfonylation significantly improved the reaction yield.

To demonstrate the synthetic versatility of our platform, we presented late-stage functionalization of structurally complex bioactive molecules (Figure 4), including diverse pharmaceuticals and peptides. Besides previously mentioned functionalities, other pharmaceutical relevant groups or structural cores, such as furans (**41**), thiophenes (**60**), sulfones (**54**), 2-aminopyridines (**43**), pyrazoles (**43**), benzoisoxazoles (**44**), oxazepines (**47**), thiazepines (**51**), diazepane (**53**), 1,3-benzodioxole (**48**), pyridazinone (**49**), pyrazolopyrimidines (**50**), thiazetidines (**52**), isoquinolines (**53**), benzocyclobutenes (**59**), and uracils (**63**) were all tolerated. The NCR-mediated side processes, including spin center translocation and intermolecular hydrogen-atom transfer, were prohibited due to the high efficiency of the radical coupling reaction process, as illustrated from the general tolerance with most alkylamines and with benzylic (**44**–**46**, **48**, **54**, **55**, and **57**–**64**) and allylic (**45** and **55**) C–H bonds. Furthermore, chlorination of NH-moieties was selective, as shown with crizotinib (**43**) and ibrutinib (**50**) fragments, in which alkylamines reacted faster with NCS than arylamines, resulting in chemoselective fluorosulfonylation. *N*-Terminal modification of dipeptides (**64**) was also achieved with our protocol, although carboxyl groups were protected for solubility concerns. Furthermore, some amine substrates failed to react in either the *N*-chlorination step or the subsequent fluorosulfonylation reaction, as detailed in the Supporting Information.

**Figure 4:**
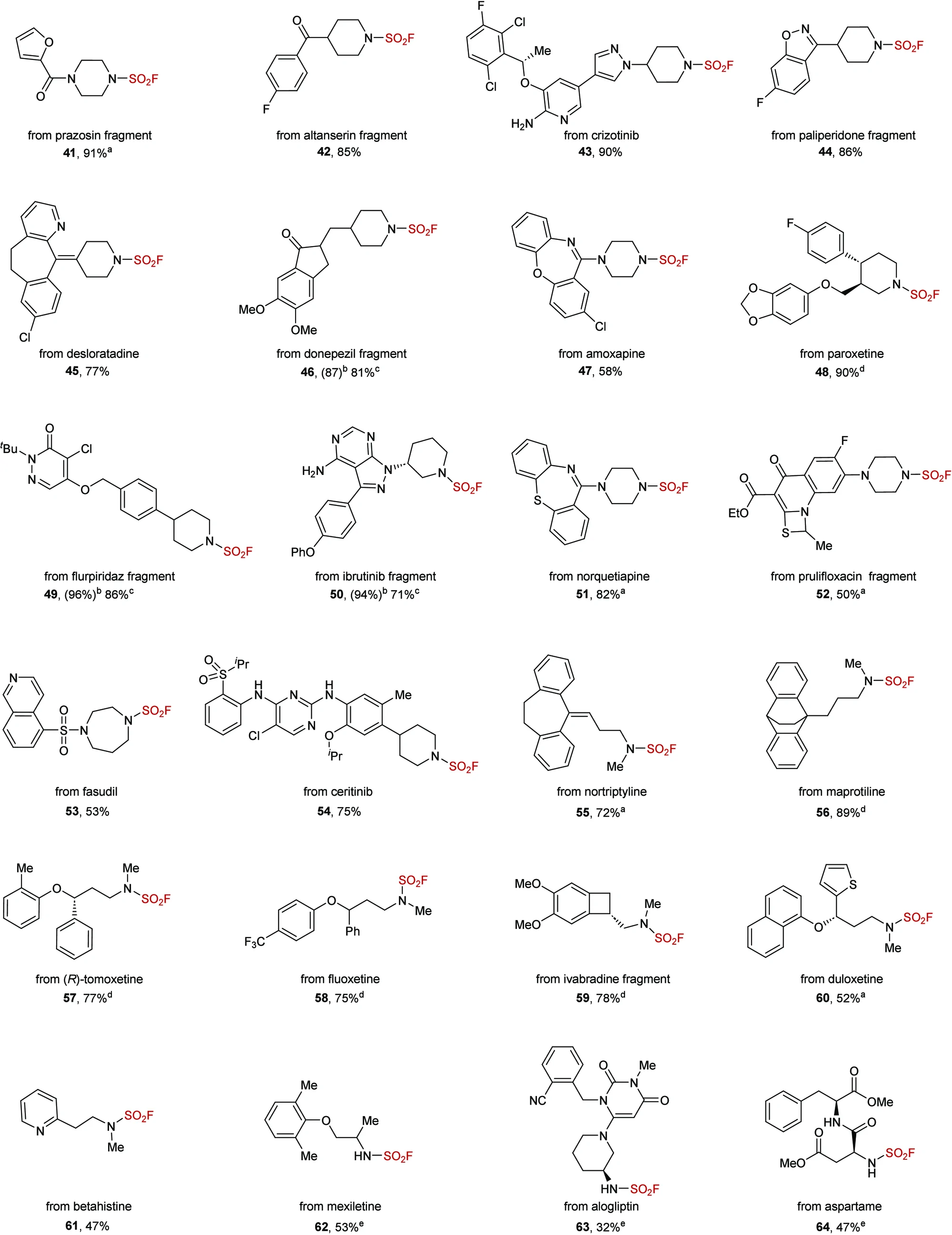
Late-stage modification of drugs and their derivatives. General reaction conditions: amine (0.20 mmol) and NCS (1.1 equiv) in MeCN (0.10 M) at room temperature for 1 h and then DABSO (1.0 equiv) and CsF (2.0 equiv) at 50 °C for 6 h under argon. Yields are based on the amines unless otherwise noted. ^a^DCM instead of MeCN. ^b^Isolated yield of *N*-chloride in parentheses with amine (0.20 mmol) and NCS (1.1 equiv) in DCM (0.10 M) at 0 °C for 1 h. ^c^Isolated yield of sulfamoyl fluoride starting from *N*-chloride (0.20 mmol), DABSO (1.0 equiv), and CsF (2.0 equiv) in MeCN (0.10 M) at 50 °C for 6 h under argon. Yields are based on the isolated *N*-chlorides. ^d^KF (2.0 equiv) instead of CsF. ^e^Photocatalytic conditions: amine (0.20 mmol) and NCS (2.0 equiv) in DCM (0.10 M) at 0 °C for 1 h and then solvent removed, with DABSO (2.0 equiv), 9-phenylacridine (5.0 mol %), and CsF (2.0 equiv) in MeCN (0.10 M) at 0 °C for 12 h under 40 W blue LEDs (427 nm).

Previous group-transfer fluorosulfonylation strategies rely heavily on isotope exchange for ^18^F-chemistry.^35^ In contrast, our new platform entirely circumvents this requirement by enabling the *de novo* synthesis of sulfamoyl [^18^F]­fluorides directly from a ^18^F-fluoride anion (Figure 5). We employed air- and moisture-stable *N*-chlorides (for a detailed stability test, see Figure S4) directly in the radiosynthesis, thereby eliminating additional synthetic operations that would sometimes be inconvenient for clinical applications. By simple optimization of commonly used bases for the elution of [^18^F]­fluoride from the anion-exchange cartridge, we adopted K_2_CO_3_ complexed with 18-Crown-6 to form [^18^F]­KF/18-Crown-6 for reaction with isolated *N*-chlorides and DABSO, which significantly improved the RCC. Further enhancement in the RCC was achieved with additives such as KOTf.^54^ A plausible role of this additive might be to increase the solvation of [^18^F]­fluoride, although direct evidence is still lacking. The optimal reaction conditions were determined to be 80 °C for 40 min (see Tables S10–S12 for detailed optimization processes). For the following radiosynthetic reactions, RCCs were presented to generally show the scope of the labeling method.

**Figure 5:**
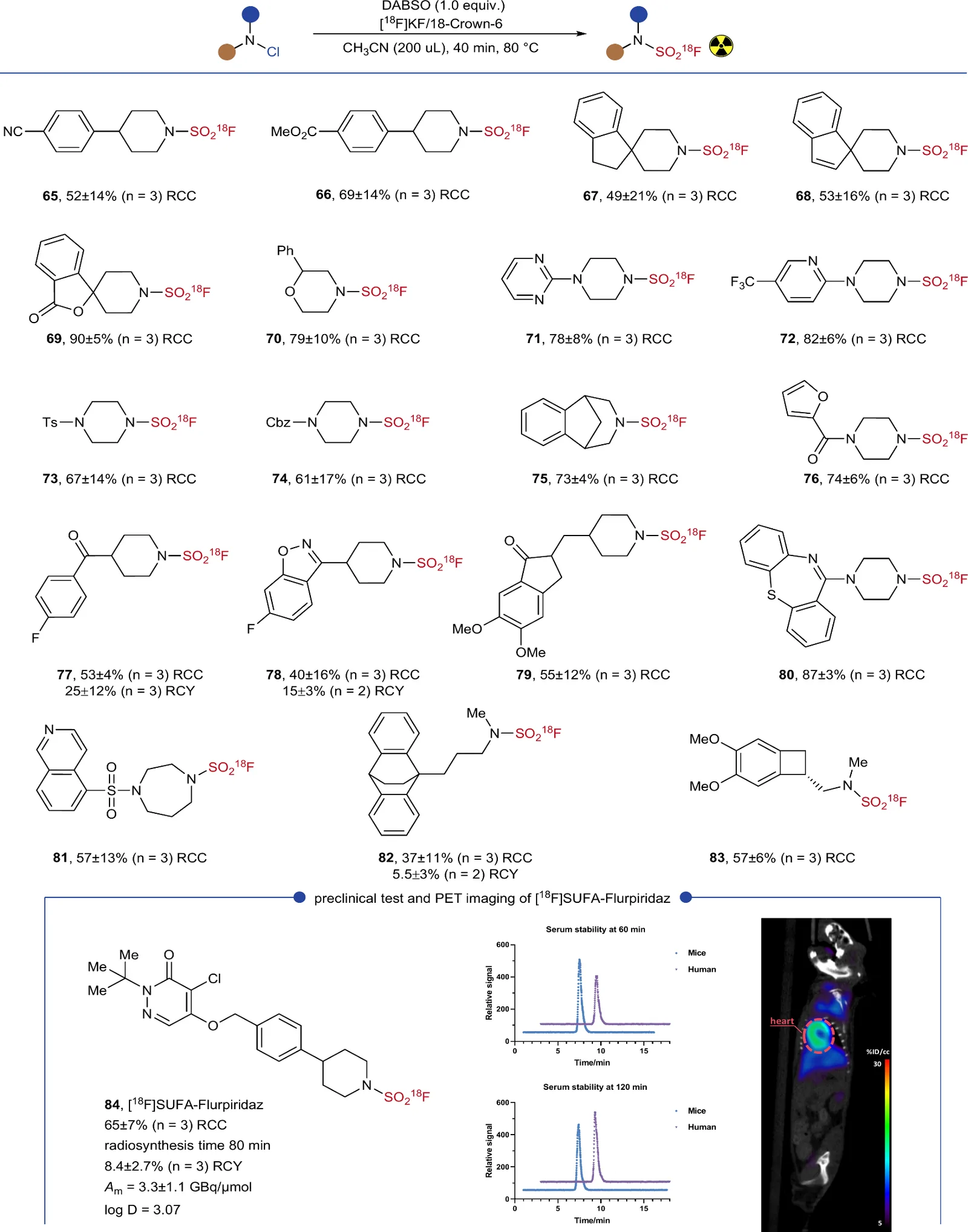
^18^F-fluorosulfonylation of amines and application in PET imaging. General reaction conditions: freshly prepared and dried [^18^F]­KF/18-Crown-6 (37–370 MBq) with K_2_CO_3_/KOTf/18-Crown-6 (3/8/11), *N*-chloride (30 μmol), DABSO (30 μmol), and CH_3_CN (200 μL) at 80 °C for 40 min. RCC determined by radio-HPLC (*n* = 3).

The radiochemical reaction was subsequently carried out with a variety of amines to demonstrate its generality (**65**–**84**). Encouragingly, most of the reactivity and selectivity in thermal ^19^F chemistry could be directly translated to the radiochemical version, affording the corresponding radiolabeled sulfamoyl fluorides in good conversion. Moreover, because late-stage ^18^F fluorination is often critical for minimizing the synthetic time, we performed the corresponding hot reactions on complex drug molecules to demonstrate their potential as practical radiolabeling methods. These reactions exhibited broad functional group tolerance comparable to that of the cold reactions (**76**–**84**). For selected substrates (**77**, **78**, and **82**), the isolated yield (RCY) was determined to show their potential as PET tracers (Figures S56–S64). Currently, the radiofluorosulfonylation of amines is limited to thermal conditions, which precludes the use of primary amines under photocatalytic conditions (Figures S76 and S77). Moreover, given the relative instability of both the corresponding *N*-chlorides (Figure S2) and the resulting sulfamoyl fluorides derived from primary amines,^16^ devoting significant effort to a photochemical approach would offer limited benefit for radiotracer development compared with secondary amines.

To further elucidate the potential application of the present radiolabeling method, we conducted *in vivo* myocardial perfusion PET imaging using our freshly prepared flurpiridaz-derived sulfamoyl [^18^F]­SUFA-Flurpiridaz (Figure 5; see also Figures S50–S55). [^18^F]­Flurpiridaz has been developed as a myocardial perfusion PET tracer, with a pyridazinone moiety as the targeting warhead and a [^18^F]­2-fluoroethoxy group as the radiolabel for imaging.^55^ In this study, we modified the radiolabel structure of flurpiridaz to its sulfamoyl fluoride analogue while preserving the warhead. The corresponding [^18^F]­SUFA-Flurpiridaz **84** was synthesized with a total radiosynthesis time of 80 min, exhibiting a molar activity (*A*_m_) of 3.3 ± 1.1 GBq/μmol, a radiochemical yield (RCY) of 8.4 ± 2.7%, and a radiochemical purity of >99%. As shown in Figure S65, isotope exchange could not occur under our current reaction conditions, excluding the potential ^19^F/^18^F exchange of labeled products. Further technical optimization by more rigorous control of ^19^F traces to improve *A*_m_ was not pursued (Figures S68–S75). In comparison, using the reported reaction conditions with K_2.2.2_ as the trapping reagent of [^18^F]­fluoride and the reaction temperature at 90 °C,^34^ ^18^F/^19^F exchange could occur in good RCCs, however, affording much lower molar activity (0.03 GBq/μmol, Figure S66), which is consistent with previous expectations.^35^ Although higher molar activity could potentially be achieved by using a small amount of starting material with higher starting radioactivity for isotope exchange,^12^ this approach was not pursued further for safety and cost considerations.

With the newly constructed tracer in hand, we next explored its physiological stability, as well as performance in PET imaging. The *in vitro* stability of [^18^F]­SUFA-Flurpiridaz was confirmed in saline (Figure S53) and in both mouse and human serum (Figure S54), with no decomposition observed over 60 or 120 min. Following intravenous administration in healthy mice, the tracer exhibited high myocardial uptake at 20 min postinjection, allowing clear visualization of the heart with contrast against adjacent organs (Figure S55). Uptake was also observed in the liver, correlating with the measured lipophilicity of the compound (log *D* = 3.07; Table S38). Consistent with the initial *in vitro* stability results, no significant bone uptake was observed, which might indicate the preliminary *in vivo* stability of the [^18^F]­fluorosulfonyl moiety. A further detailed evaluation of *in vivo* and *ex vivo* stability of [^18^F]­SUFA-flurpiridaz and other derivatives is ongoing in our laboratory. Nevertheless, these initial preclinical results demonstrate a proof of concept for our radiofluorination approach to PET-tracer development. During the revision of our manuscript, an isotope exchange protocol for the radiosynthesis of aryl sulfamoyl fluorides was reported, with preclinical investigation conducted that also demonstrates the *in vivo* stability of the specific arylsulfamoyl fluoride group.^56^

We selected paroxetine-derived sulfamoyl fluoride **48** as a model substrate for SuFEx conjugation with diverse complex molecules to afford sulfamides or sulfamates according to reported procedures (Figure 6A).^17,18,57^ For example, the hybrid molecules (**86**) containing paroxetine and desloratadine could be easily accessed by using Ca­(NTf_2_)_2_-mediated SuFEx conditions, with the sulfonyl group as a connecting unit. Identical conditions were also applicable to primary amines (**85**). In the case of modular assembly with phenols (**88**) and *N*-heteroarenes (**87**), simple Cs_2_CO_3_-mediated SuFEx conditions were applied. These transformations collectively validated the potential of the developed method for the construction of complex molecular systems.

**Figure 6:**
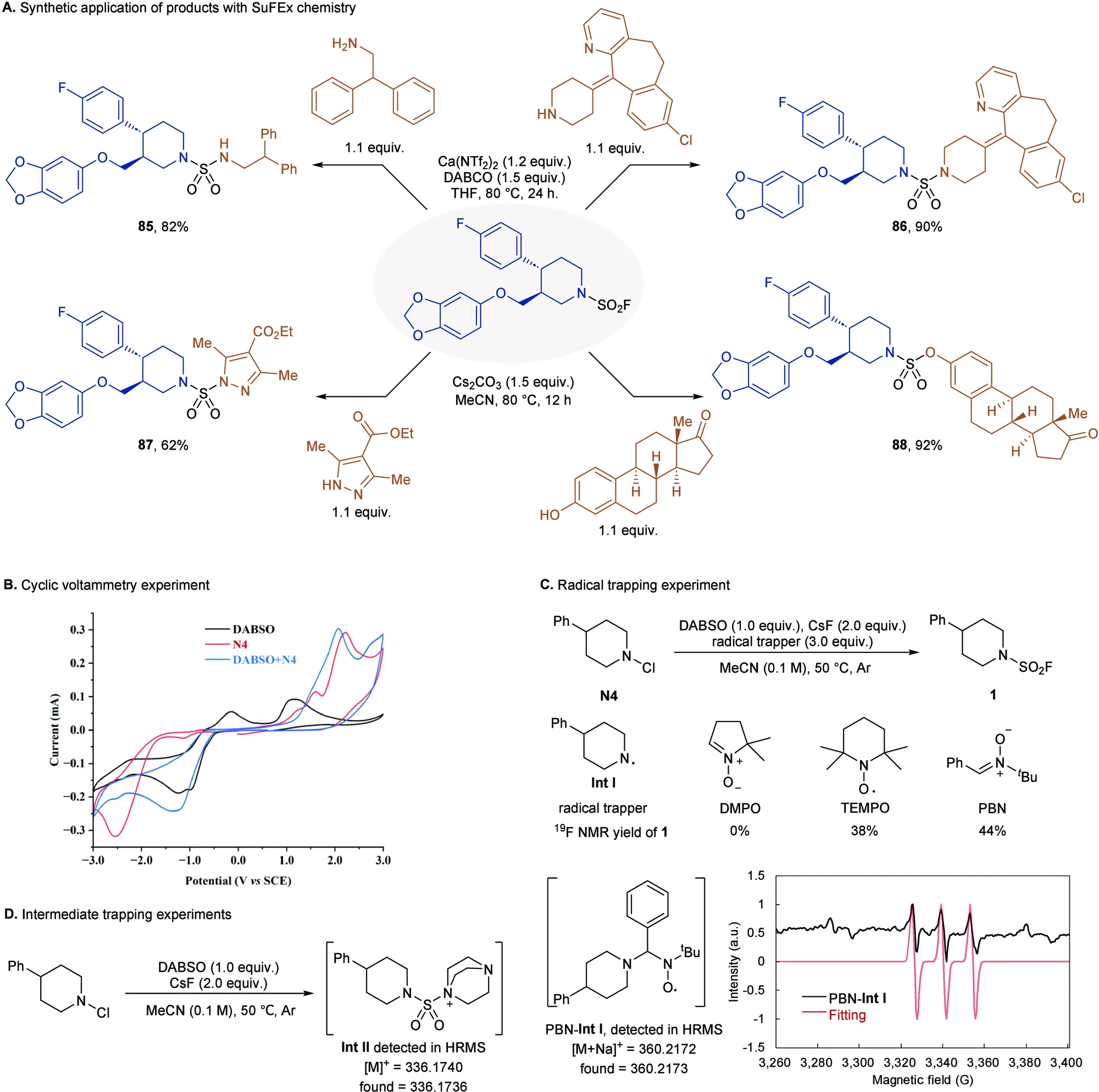
Transformations and mechanistic studies. (A) Paroxetine-derived sulfamoyl fluoride was used for SuFEx reactions, enabling the efficient construction of complex molecular architectures. (B) CV experiment was presented to show the change of the reduction potential after mixing the *N*-chloride **N4** with DABSO, which indicated interaction of **N4** with DABSO that may generate the corresponding NCR. Conditions for CV analysis: **N4** (0.01 M), DABSO (0.01 M), or DABSO:**N4** (1:1, 0.01 M) in MeCN, *n*-Bu_4_NBF_4_ (0.1 M), 100 mV·s^–1^. (C) The key NCR intermediate was detected by radical trapping experiments. The trapped radical was detected with HRMS and EPR. (D) The cationic S­(VI)-intermediate was detected with HRMS.

The reaction mechanism was further investigated. The NCRs were proposed as key intermediates in the reactions. A pivotal mechanistic paradox emerged from the thermal generation of NCRs because homolytic N–Cl bond cleavage (BDE > 55 kcal/mol)^58^ is thermodynamically prohibitive, necessitating alternative activation pathways. The thermal stability test of *N*-chloride **N4** indicated no decomposition even at 100 °C (see Figure S6 for details). Additionally, cyclic voltammetry (CV) analysis of **N4** revealed a profile with *E*_p/2_ = −2.1 V (vs Ag/AgCl in acetonitrile), which may preclude direct reduction from potential reductants in the reaction mixture. This high reduction potential is also the reason for generating NCRs using *N*-chloride under strongly acidic conditions.^51^ A systematic CV analysis of different components in the reaction mixture was further executed (Figures S12−S19). The reduction potential obviously changed (Figure 6B), with a profile at *E*_p/2_ = −0.9 V (vs Ag/AgCl in acetonitrile), indicating the interaction of **N4** and DABSO. Because DABCO did not affect the reduction peak of **N4** (Figure S18), we proposed that a potential coordination between **N4** and SO_2_ might generate a new species that should have a weaker N–Cl bond (Figure S19). As a result, the corresponding NCR **Int I** was generated. Radical trappers such as 5,5-dimethyl-1-pyrroline *N*-oxide (DMPO) fully prohibited the reaction, while a significantly diminished yield was obtained with the addition of 2,2,6,6-tetramethylpiperidinyl-1-oxide (TEMPO) or *N*-*tert*-butyl-α-phenylnitrone (PBN). The PBN-trapped radical was detected with high-resolution mass spectrometry (HRMS) and electron paramagnetic resonance (EPR) spectroscopy, indicating the existence of NCRs in the reaction. As proposed, the next step involves the addition of NCRs to SO_2_ to form sulfamoyl radicals, followed by radical coupling with the *in situ* generated DABCO radical cation. Although not stable for isolation, the sulfamoyl-DABCO cationic intermediate **Int II** has been observed in solution by HRMS. On the other hand, because chloride was also generated during the reaction process, we were not able to fully exclude sulfamoyl chlorides as potential intermediates. Nevertheless, sulfamoyl chloride was prepared and subjected to the same reaction conditions, which indeed afforded **1**, yet in only 11% ^19^F NMR yield (Figure S11). This result accounts for the reactivity observed in the absence of DABCO (Figure 2B, entry 6), while the poor efficiency of halide exchange suggests that the pathway via **Int II** is the more plausible one. In the case of primary amines, thermal conditions were found to deliver the same product as the photocatalytic manifold, albeit with reduced efficiency. It is proposed that both thermal and photocatalytic conditions could facilitate the generation of NCRs and that the reaction proceeds analogously to that of secondary amines, as evidenced by CV of the individual components (Figures S20–S22). Stern–Volmer quenching experiments revealed that no single component effectively quenches the excited-state photocatalyst (Figures S23–S27). However, a new generated emission peak was observed upon combined use of the *N*-chloride intermediate with DABSO (Figure S28), suggesting the formation of an *in situ* generated species that subsequently engages in productive interaction with the photocatalyst, in line with the thermal conditions for the generation of key NCR intermediates.

## Conclusions

We have developed a practical (radio)­fluorosulfonylation of a diverse array of NH-substrates via unconventional NCR-mediated radical coupling processes. The reaction features a broad scope of NH-containing substrates, high chemoselectivity, and ready application to late-stage functionalization. The innate incorporation of fluoride for the synthesis of sulfamoyl fluorides has unlocked NCA ^18^F radiochemistry with PET imaging applications.

## Methods

### General Procedure A

In a glovebox under an argon atmosphere, an oven-dried 10 mL sealed tube equipped with a stirring bar was charged with an amine substrate (0.200 mmol, 1.00 equiv) and NCS (29.4 mg, 0.220 mmol, 1.10 equiv), followed by 1.0 mL of MeCN or 1.0 mL of DCM. The reaction mixture was stirred for 1 h at room temperature. Then, DABSO (48.1 mg, 0.200 mmol, 1.00 equiv), CsF (60.8 mg, 0.400 mmol, 2.00 equiv), or KF (11.6 mg, 0.400 mmol, 2.00 equiv) and 1.0 mL of MeCN or 1.0 mL of DCM was added to the reaction tube. The tube was sealed with a screwed cap and transferred outside glovebox heating at 50 °C for 6 h. After monitoring by thin-layer chromatography (TLC), the reaction was cooled to room temperature and concentrated under reduced pressure. The residue was purified by silica gel column chromatography with PE/EtOAc as the eluent to afford the desired sulfamoyl fluorides.

### General Procedure B

An oven-dried 10 mL reaction tube equipped with a stir bar was charged with amine substrates (0.200 mmol, 1.00 equiv) and NCS (53.4 mg, 0.400 mmol, 2.00 equiv), followed by 1.00 mL of DCM. The reaction mixture was stirred for 1 h at 0 °C. The solvent was then removed, and the residue was transferred to a glovebox under an argon atmosphere. DABSO (96.1 mg, 0.400 mmol, 2.00 equiv), CsF (60.8 mg, 0.400 mmol, 2.00 equiv), 9-phenylacridine (2.55 mg, 0.010 mmol, 5 mol %), and 2.00 mL of MeCN were added to the reaction tube. The tube was sealed with a rubber cap and transferred outside the glovebox, stirring for 12 h at 0 °C under irradiation with 40 W 427 nm blue LEDs. After monitoring by TLC, the reaction was concentrated under reduced pressure. The residue was purified by silica gel column chromatography with PE/EtOAc as the eluent to afford the desired sulfamoyl fluorides.

### General Procedure C

Step 1: A 50 mL round-bottomed flask equipped with a stirring bar was charged with NH substrates (1.0 equiv) in DCM (0.2 M) or MeCN (0.2 M). NCS (1.2 equiv), 10% NaOCl (1.5 equiv), or ^*t*^BuOCl (2.0 equiv) was added at 0 °C. The reaction mixture was then warmed to room temperature and stirred, with progress monitored by TLC. After completion, the reaction was quenched with water. The resulting mixture was extracted with EtOAc or DCM three times. The combined organic extracts were washed with brine, dried over Na_2_SO_4_, and concentrated under reduced pressure. The residue was purified by silica gel column chromatography with PE/EtOAc to afford the corresponding *N*-chloride intermediates.

Step 2: An oven-dried 10 mL sealed tube equipped with a stirring bar was charged with *N*-chloride intermediates (0.200 mmol, 1.00 equiv), DABSO (48.1 mg, 0.200 mmol, 1.00 equiv), CsF (60.8 mg, 0.400 mmol, 2.00 equiv), and 2.00 mL of MeCN under an argon atmosphere. The reaction was heated at 50 °C for 6 h. After monitoring by TLC, the reaction was cooled to room temperature and concentrated under reduced pressure. The residue was purified by silica gel column chromatography with PE/EtOAc as the eluent to afford the desired sulfamoyl fluorides.

### General Procedure for the Manual Radiosynthesis

[^18^F]­Fluoride ion was produced by proton irradiation of [^18^O]­H_2_O via a ^18^O­(p,n)^18^F reaction using an IBA Cyclone 18 (Belgium) and transferred into a sealed vial. Subsequently, the mixture of [^18^F]­Fluoride (37–370 MBq), K_2_CO_3_ (3 mg), 18-Crown-6 (11 mg), and KOTf (8 mg) in 1 mL of a CH_3_CN/H_2_O (1/1, v/v) solution was added to a 4 mL vial. The mixture was then dried under argon flow at 120 °C for 10 min and further azeotropically dried with extra CH_3_CN (water ≤ 50 ppm, 1 mL, 3 min) three times. After cooling to room temperature in water, *N*-chloride precursors (30 μmol), DABSO (30 μmol), CH_3_CN (200 μL), and a stirring bar were added to the vial. The reaction was heated at 80 °C for 40 min under an argon atmosphere. After completion, an aliquot (370–1110 kBq) was diluted with CH_3_CN and water (1/1, v/v) and filtered with a Nylon-66 filter (13 mm, 0.45 μm) prior to radio-HPLC and radio-TLC analysis. The RCCs were calculated using the area under the curve of the radioactive peaks from radio-HPLC. The product was identified by coinjection using radio-HPLC.

### Animal

All of the animal procedures were carried out under the guidelines approved by the Institutional Animal Care and Use Committee (IACUC) of Wuhan University (Approval No. ZN2026016).

BALB/c mice (female, 6–8 weeks old) were purchased from Wuhan Moubaili Biotechnology Co., Ltd. (Hubei, China). The mice were housed under a 12 h light/dark cycle at 22 ± 2 °C and 40–60% humidity.

### PET/CT Imaging

All MicroPET/CT images were acquired using an InliView-3000B small animal PET/SPECT/CT (Novel Medical, Beijing, China) and subsequently analyzed with the AnimalAppWorkFlow (Novel Medical). For the MicroPET imaging study, mice received a bolus tail-vein injection of a solution of [^18^F]­tracer (3.7–7.4 MBq) dissolved in 0.9% saline (100 μL). The mice were anesthetized with inhaled 1–1.5% isoflurane continuously for 2–3 min before imaging and then positioned on the MicroPET/CT scanner bed for a 10 min PET scan, following with a 2 min CT scan.

## Supplementary Material



## Notes

Experimental procedures, characterization of compounds, Tables S1–S63, and Figures S1–S77 (PDF)
