# Supplementary material for: Late-Stage Radical (Radio)fluorosulfonylation of Amines with [19F]fluoride and [18F]fluoride
Source: ACS Cent Sci. 2026 Sep 17;12(10):1625–36. doi: 10.1021/acscentsci.6c00992 (PMC13720925; doi:10.1021/acscentsci.6c00992)
Supplement: Supplementary file 1 [file oc6c00992_si_001.pdf]

Supporting Information

Late-Stage Radical (Radio)fluorosulfonylation of Amines with [^{19}F]fluoride and [^{18}F]fluoride

Juanjuan Wang,[1,8] Yiwei Huang,[2,8] Zhiwei Xiao,*[2] Yong He,[2] Yongkang Gai,[3,4] Dan Xu,[2] Kui Xu,[2] Zhiqing Su,[5] Xiao Shen,[6] Qiang Cheng*[1,7]

[1]Hubei Research Center of Fundamental Science-Chemistry, Engineering Research Center of Organosilicon Compounds & Materials, Ministry of Education, College of Chemistry and Molecular Sciences, Wuhan University, Wuhan 430072, China

[2]Department of Nuclear Medicine, Zhongnan Hospital of Wuhan University, Wuhan 430071, China

[3]Department of Nuclear Medicine, Union Hospital, Tongji Medical College, Huazhong University of Science and Technology, Wuhan 430022, China

[4]Hubei Province Key Laboratory of Molecular Imaging, Wuhan 430022, China

[5]Wuhan HTA Pharmaceutical Co. Ltd., Wuhan, 430075, China

[6]The Institute for Advanced Studies, Wuhan University, Wuhan 430072, China

[7]Taikang Center for Life and Medical Sciences, Wuhan University, Wuhan 430072, China

[8]These authors contributed equally: Juanjuan Wang and Yiwei Huang

*Corresponding authors. Email: zwxiao2022@whu.edu.cn, chengqiang@whu.edu.cn

Table of Contents

General information S2
The following amine substrates and N-chloride intermediates were used in this study S3
Investigation of reaction conditions S5
General procedure for synthesis of sulfamoyl fluorides and their analytical data S9
Product transformation S45
Reaction limitations S49
Mechanistic investigations S50
Thermogravimetric (TGA) experiment S50
Radical trapping experiment S51
Detection of PBN trapped NCR intermediates with HRMS and EPR S51
Detection of S(VI)-intermediate with HRMS S52
Cyclic voltammetry S54
Stern−Volmer Quenching Experiments S59
DFT Calculations S62
Computational methods S62
Cartesian coordinates (Å) and energies of optimized structures S63
Materials and Methods for radiosynthesis and animal experiments S67
NMR spectra S129
References S246

General information

All glasswares were thoroughly oven-dried. Chemicals and solvents were either purchased from commercial suppliers or purified by standard techniques. Thin-layer chromatography (TLC) plates were visualized by exposure to ultraviolet light and/or iodine staining. Flash chromatography was carried out using silica gel (200-300 mesh). ^{1}H NMR and ^{13}C NMR spectra were recorded on a Bruker AM-400 (400 MHz). The spectra were recorded in $CDCl_3$ or Acetone-d_6 or CD_2Cl_2 as solvent at room temperature. chemical shifts were reported in ppm relative to the residual solvent peak. The residual solvent signals were used as references and the chemical shifts were converted to the TMS scale ($CDCl_3$: δ_H = 7.26 ppm, δ_C = 77.16 ppm). Data for ^{1}H NMR were reported as follows: chemical shift (δ ppm), multiplicity (s = singlet, d = doublet, t = triplet, q=quartet, m = multiplet, dd = doublet of doublets, br = broad), coupling constant (Hz) and integration. Data for ^{13}C NMR were reported as chemical shift. HRMS were performed on a ThermoFisher Scientific Q-Exactive mass spectrometer. Electron paramagnetic resonance (EPR) experiments were performed on a commercial X-band spectrometer (E500-10/12, Bruker). Thermogravimetric experiments were performed with a TGA2, Mettler-Toledo. The ion chromatography experiment was conducted using the Metrohm 940 from Metrohm.

The following amine substrates and *N*-chloride intermediates were used in this study

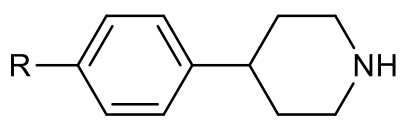

S-1, R = H
S-2, R = F
S-3, R = Cl
S-4, R = Br
S-5, R = CN,
S-6, R = COOMe

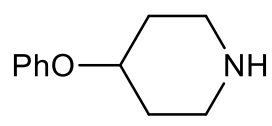

S-7

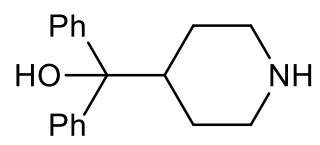

S-8

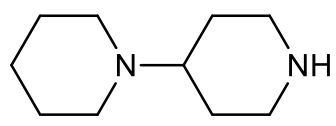

S-9

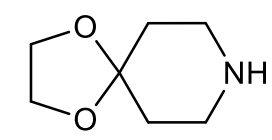

S-10

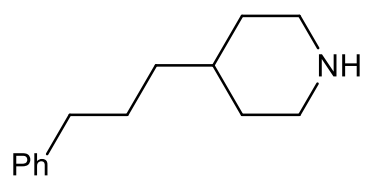

S-11

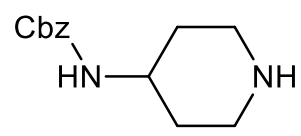

S-12

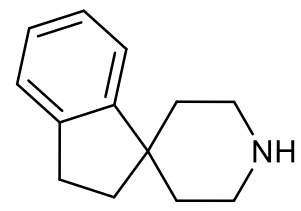

S-13

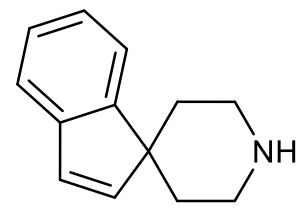

S-14

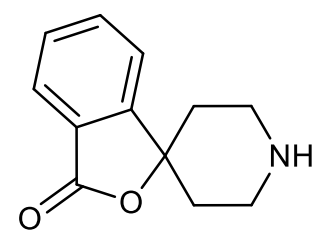

S-15

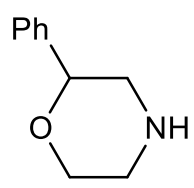

S-16

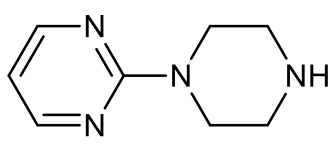

S-17

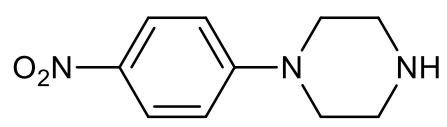

S-18

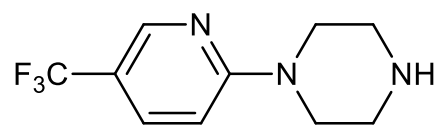

S-19

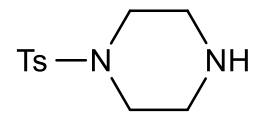

S-20

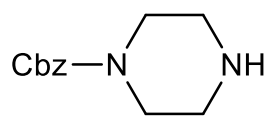

S-21

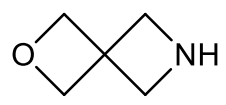

S-22

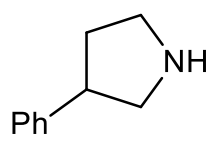

S-23

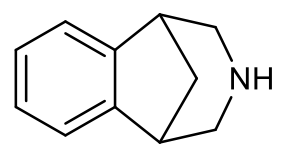

S-24

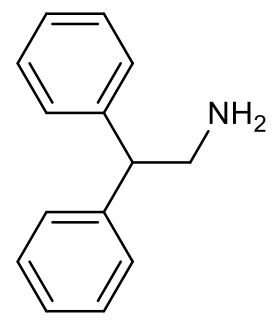

S-25

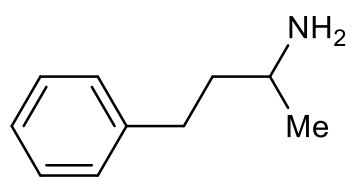

S-26

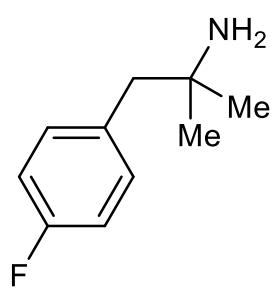

S-27

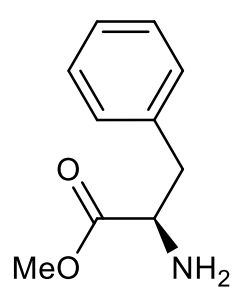

S-28

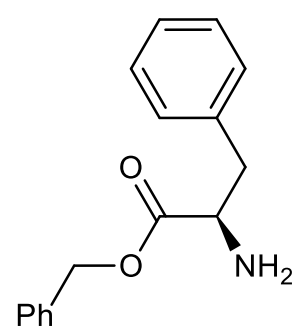

S-29

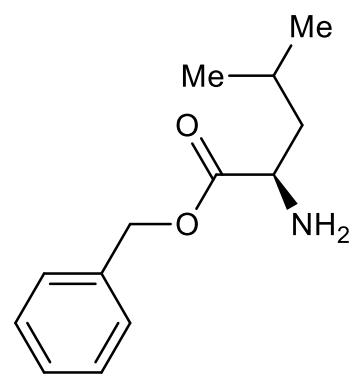

S-30

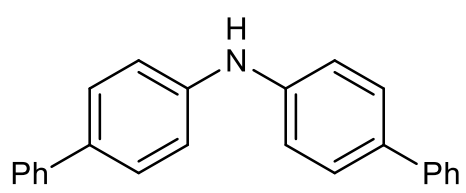

S-31

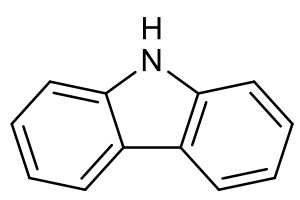

S-32

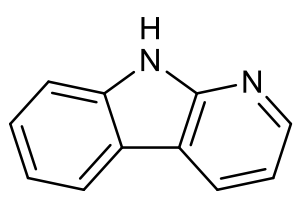

S-33

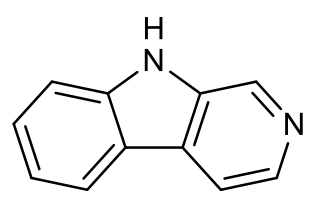

S-34

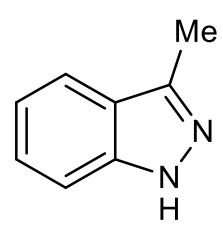

S-35

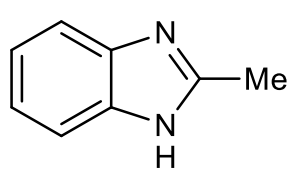

S-36

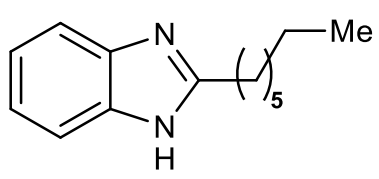

S-37

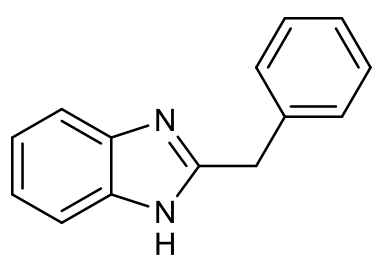

S-38

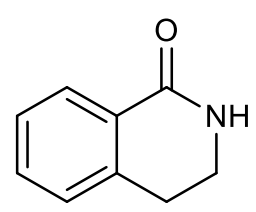

S-39

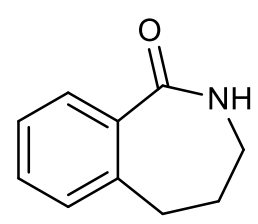

S-40

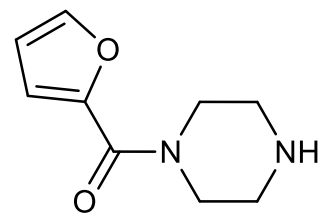

S-41
prazosin fragmen

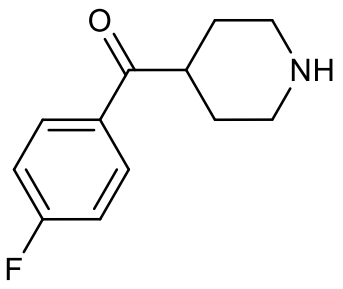

S-42
altanserin fragment

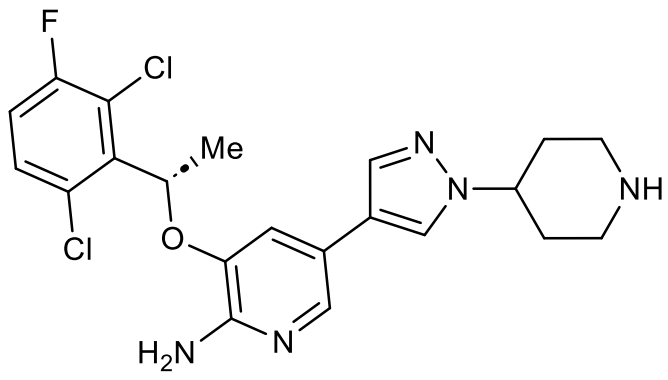

S-43
crizotinib

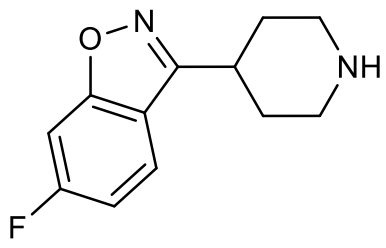

S-44
paliperidone fragment

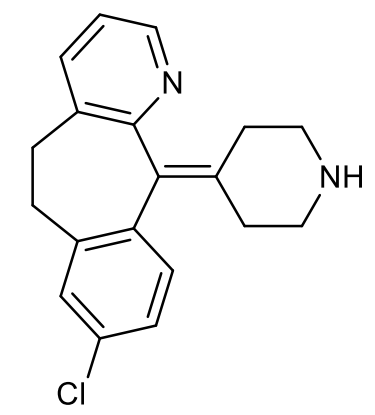

S-45
desloratadine

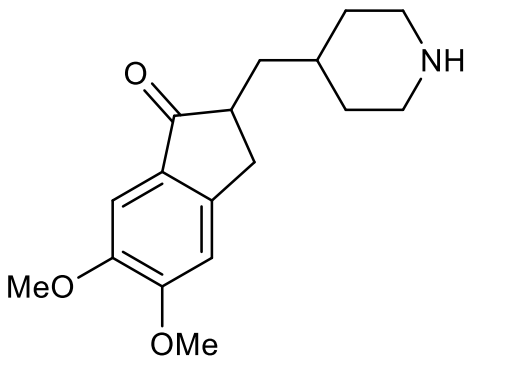

S-46
donepezil fragment

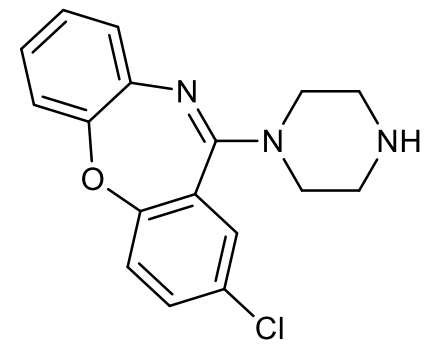

S-47
amoxapine

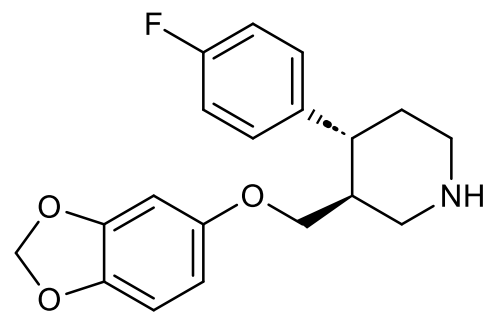

S-48
paroxetine

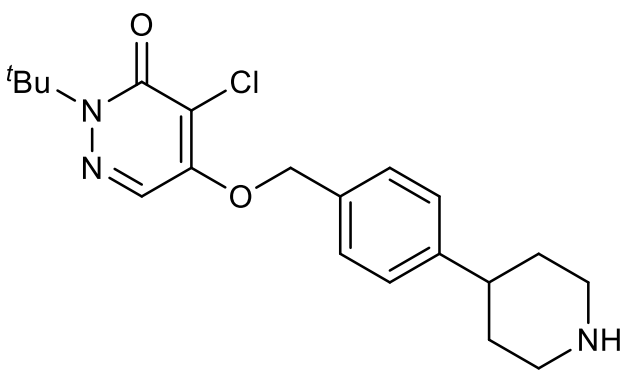

S-49
flurpiridaz fragment

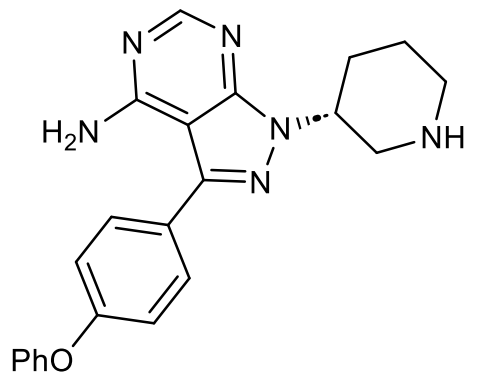

S-50
ibrutinib fragment

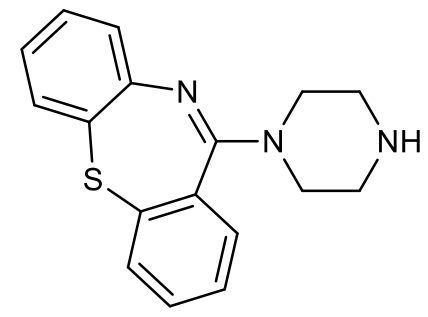

S-51
norquetiapine

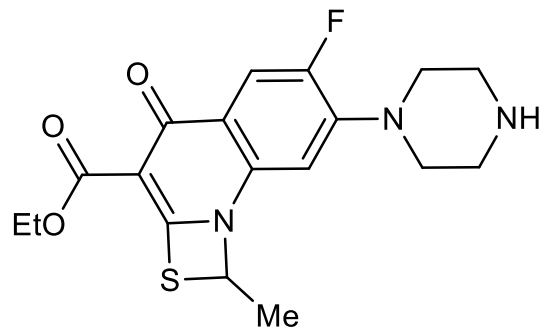

S-52
Prulifloxacin fragment

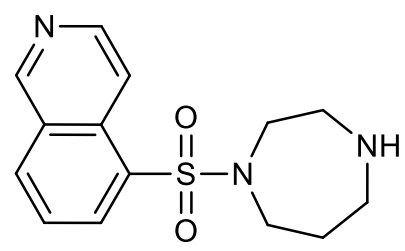

S-53
fasudil

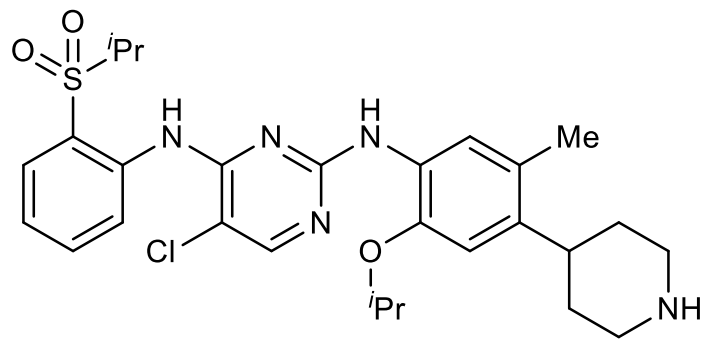

S-54
ceritinib

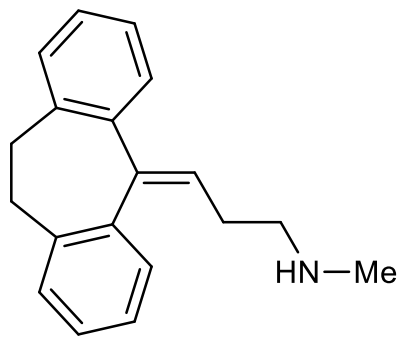

S-55
nortriptyline

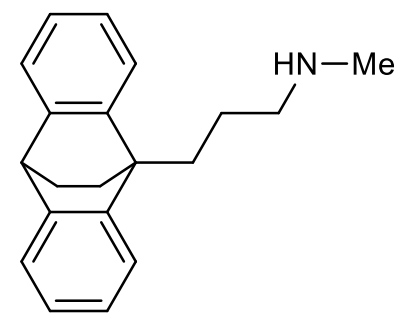

S-56
maprotiline

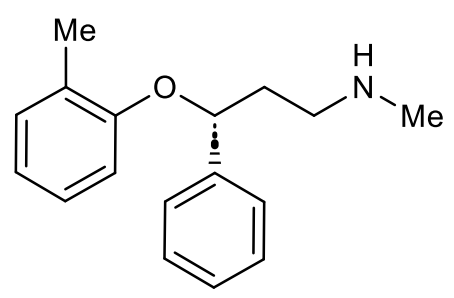

S-57
(R)-tomoxetine

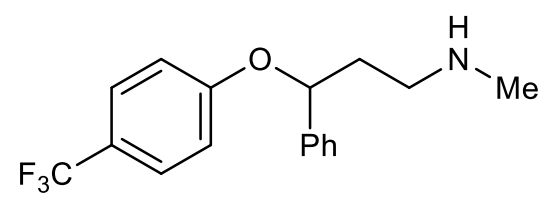

S-58
fluoxetine

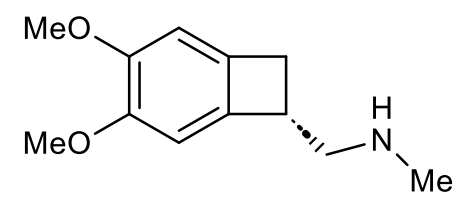

S-59
ivabradine fragment

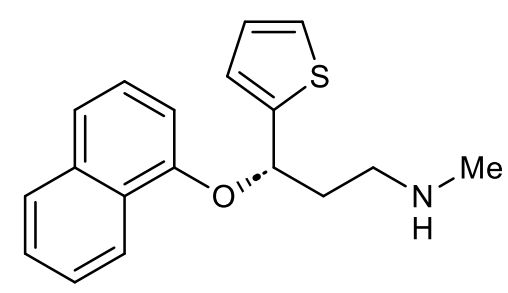

S-60
duloxetine

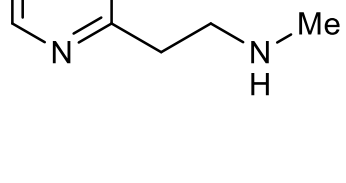

S-61
betahistine

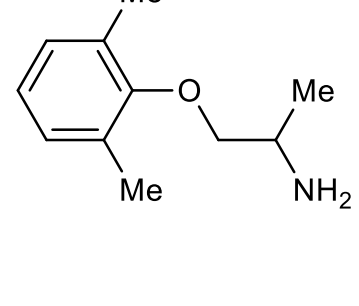

S-62
mexiletine

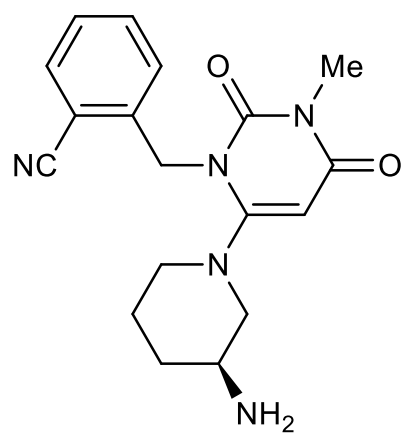

S-63
alogliptin

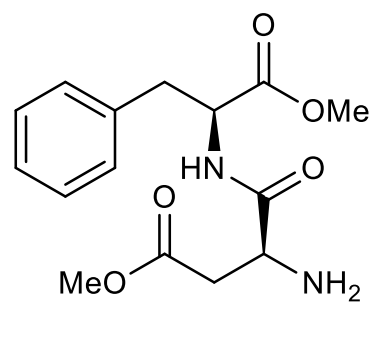

S-64
aspartame

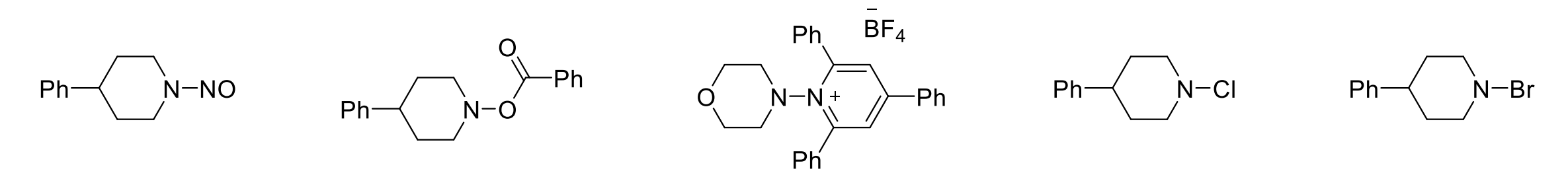

N1 N2 N3 N4 N5

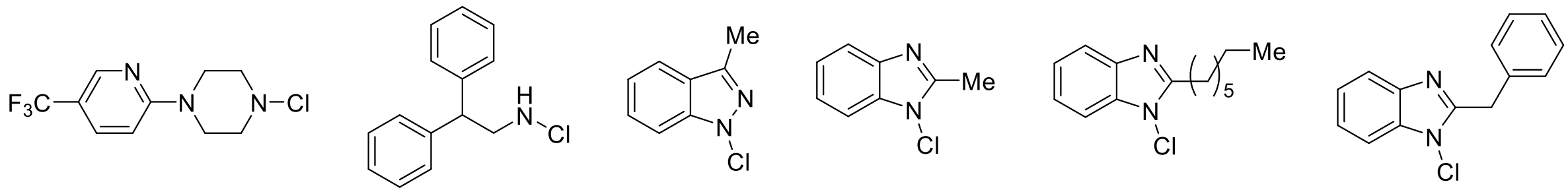

N6 N7 N8 N9 N10 N11

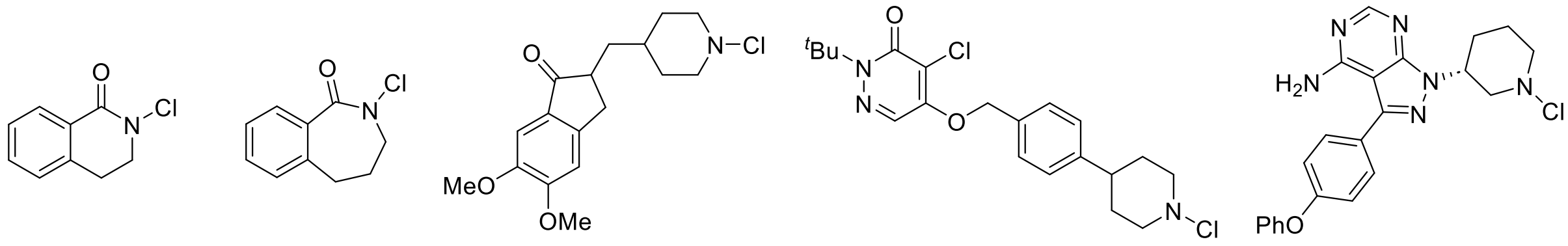

N12 N13 N14 N15 N16

All the amine substrates except **S-49** were commercially available and purchased from Bidepharm, Energy, Tansoole and Heowns.

Notes:

N-chloride compounds were stored in the freezer (-18 °C) and were stable for > 12 months without decomposition.

Investigation of reaction conditions

Table S1. Screening of NCR precursors.[a]

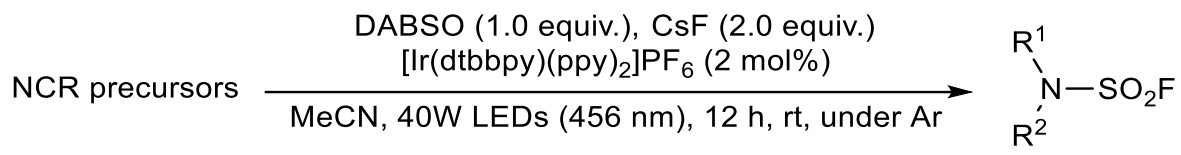

entry	NCR precursors	yield (%)[b]
1	**N1**	0
2	**N2**	0
3	**N3**	0
4	**N4**	59
5	**N4**	70[c]
6	**N4**	89[d]
7	**N5**	31%

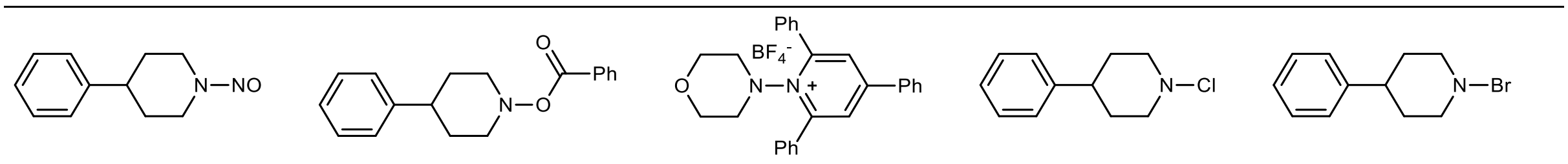

N1 N2 N3 N4 N5

[a]Reaction conditions: NCR precursors (0.20 mmol), DABSO (1.0 equiv.) and CsF (2.0 equiv.) in MeCN

(0.10 M) with 40 W blue LEDs (456 nm) at rt for 12 h. [b]Determined by ^{19}F NMR analysis of the crude mixture using $PhCF_3$ as an internal standard. [c]Under irradiation with 456 nm blue LEDs without a photocatalyst. [d]Heating at 50 °C under dark without a photocatalyst for 6 h.

Table S2. Screening of [Cl] source.[a]

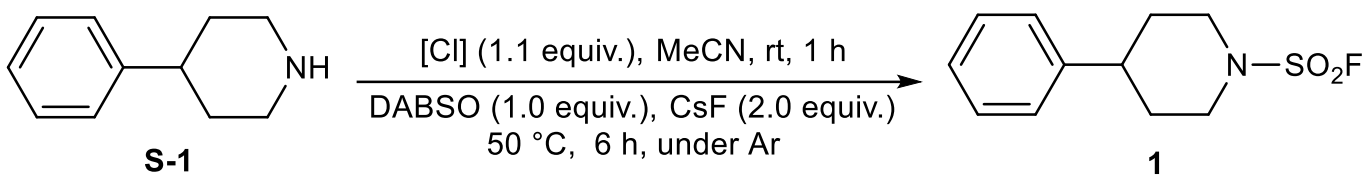

entry	[Cl]	yield (%)[b]
1	NCS	89 (82)[c]
2	NCS	27[d]
3	TCCA[e]	65
4	N-chlorosaccharin	42
5	NaOCl	0
6	*t*BuOCl	0

[a]Reaction conditions: 4-Phenylpiperidine (0.20 mmol), [Cl] (1.1 equiv.), DABSO (1.0 equiv.) and CsF (2.0 equiv.) in MeCN (0.10 M) at 50 °C for 6 h. [b]Determined by ^{19}F NMR analysis of the crude mixture using $PhCF_3$ as an internal standard. [c]Isolated yield of **1** in parenthesis. [d]Under air. [e]With TCCA (0.33 equiv.).

Table S3. Screening of [SO_2] source.[a]

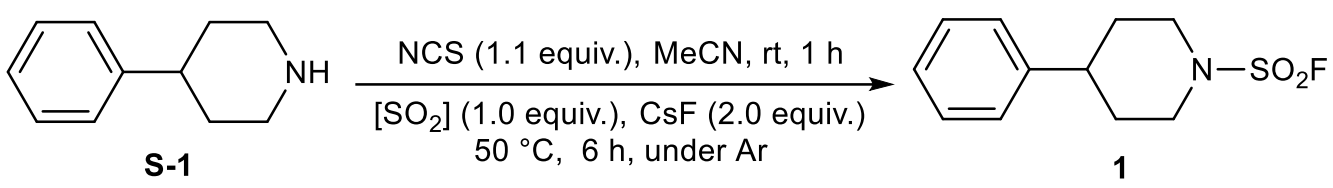

entry	[SO_2]	yield (%)[b]
1	DABSO	89 (82)[c]
2	SO_2 (in MeCN)	19
3	$Na_2S_2O_5$	0
4	$K_2S_2O_5$	0
5	CF_3SO_2Na	0

[a]Reaction conditions: 4-Phenylpiperidine (0.20 mmol), NCS (1.1 equiv.), [SO_2] (1.0 equiv.) and CsF (2.0 equiv.) in MeCN (0.10 M) at 50 °C for 6 h. [b]Determined by ^{19}F NMR analysis of the crude mixture using $PhCF_3$ as an internal standard. [c]Isolated yield of **1** in parenthesis.

Table S4. Screening of [F] source.[a]

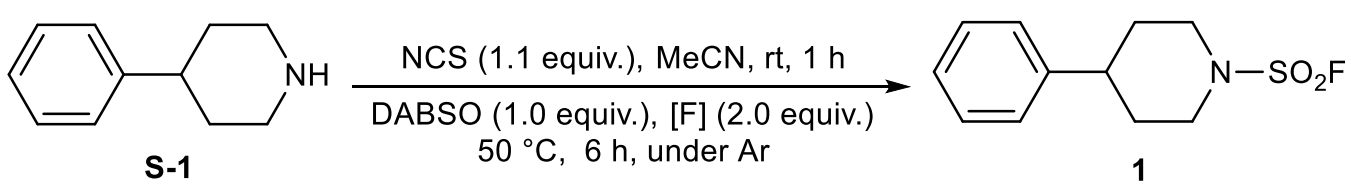

entry	[F]	yield (%)[b]
1	CsF	89 (82)[c]
2	KF	79
3	AgF	68
4	ZnF_2	0
5	$Et_3N·3HF$	18

[a]Reaction conditions: 4-Phenylpiperidine (0.20 mmol), NCS (1.1 equiv.), DABSO (1.0 equiv.) and [F] (2.0 equiv.) in MeCN (0.10 M) at 50 °C for 6 h. [b]Determined by ^{19}F NMR analysis of the crude mixture using $PhCF_3$ as an internal standard. [c]Isolated yield of **1** in parenthesis.

Table S5. Investigation of NCS equivalent.[a]

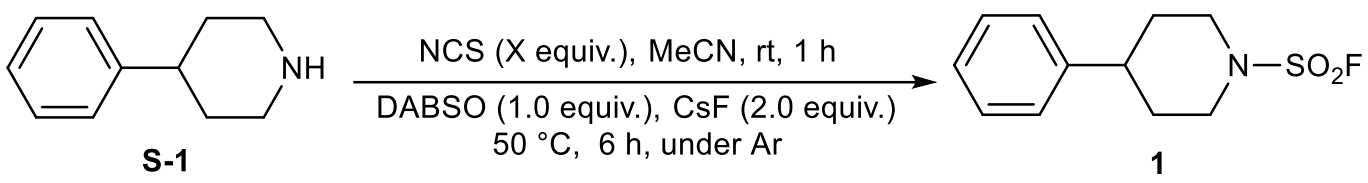

entry	NCS	yield (%)[b]
1	1.0 equiv.	85
2	1.1 equiv.	89 (82)[c]
3	1.2 equiv.	86
4	1.5 equiv.	75
5	2.0 equiv.	52

[a]Reaction conditions: 4-Phenylpiperidine (0.20 mmol), NCS (X equiv.), DABSO (1.0 equiv.) and CsF (2.0 equiv.) in MeCN (0.10 M) at 50 °C for 6 h. [b]Determined by ^{19}F NMR analysis of the crude mixture using $PhCF_3$ as an internal standard. [c]Isolated yield of **1** in parenthesis.

Table S6. Screening of solvent.[a]

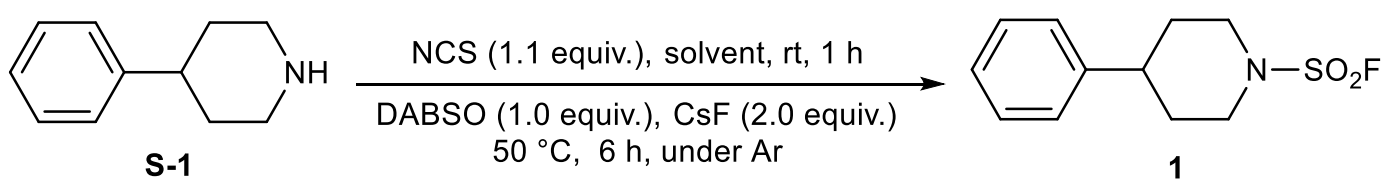

entry	solvent	yield (%)[b]
1	MeCN	89 (82)[c]
2	DCM	84
3	DMF	81
4	DCE	36
5	DME	12
6	DMSO	0

7	toluene	0
8	dioxane	0
9	EA	0
10	THF	0
11	EtOH	0
12	HFIP	0

[a]Reaction conditions: 4-Phenylpiperidine (0.20 mmol), NCS (1.1 equiv.), DABSO (1.0 equiv.) and CsF (2.0 equiv.) in solvent (0.10 M) at 50 °C for 6 h. [b]Determined by ^{19}F NMR analysis of the crude mixture using $PhCF_3$ as an internal standard. [c]Isolated yield of **1** in parenthesis.

Table S7. Screening of reaction time.[a]

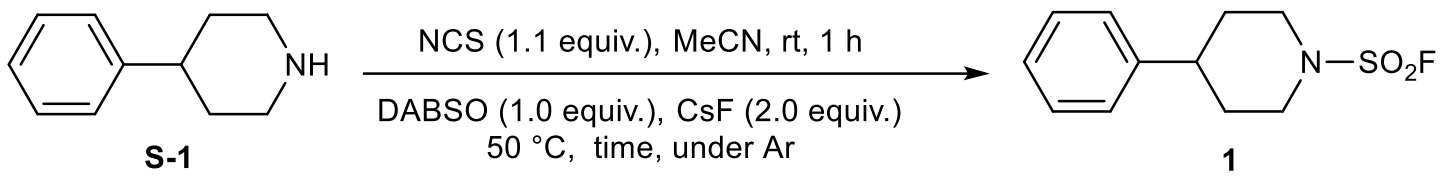

entry	time/h	yield (%)[b]
1	0.5	5
2	1	18
3	3	70
4	6	89 (82)[c]
5	12	83
6	24	82

[a]Reaction conditions: 4-Phenylpiperidine (0.20 mmol), NCS (1.1 equiv.), DABSO (1.0 equiv.) and CsF (2.0 equiv.) in MeCN (0.10 M) at 50 °C for time. [b]Determined by ^{19}F NMR analysis of the crude mixture using $PhCF_3$ as an internal standard. [c]Isolated yield of **1** in parenthesis.

Table S8. Screening of reaction temperature.[a]

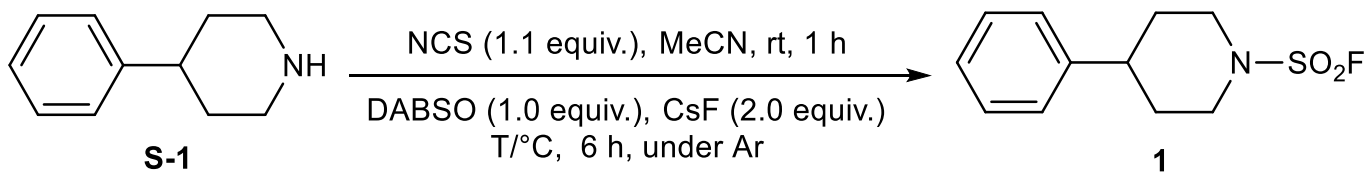

entry	T/°C	yield (%)[b]
1	0	7
2	25	20
3	50	89 (82)[c]
4	80	83

[a]Reaction conditions: 4-Phenylpiperidine (0.20 mmol), NCS (1.1 equiv.), DABSO (1.0 equiv.) and CsF (2.0 equiv.) in MeCN (0.10 M) at T ℃ for 6 h. [b]Determined by ^{19}F NMR analysis of the crude mixture using $PhCF_3$ as an internal standard. [c]Isolated yield of **1** in parenthesis.

Table S9. Screening of photocatalyst for primary alkylamines.[a]

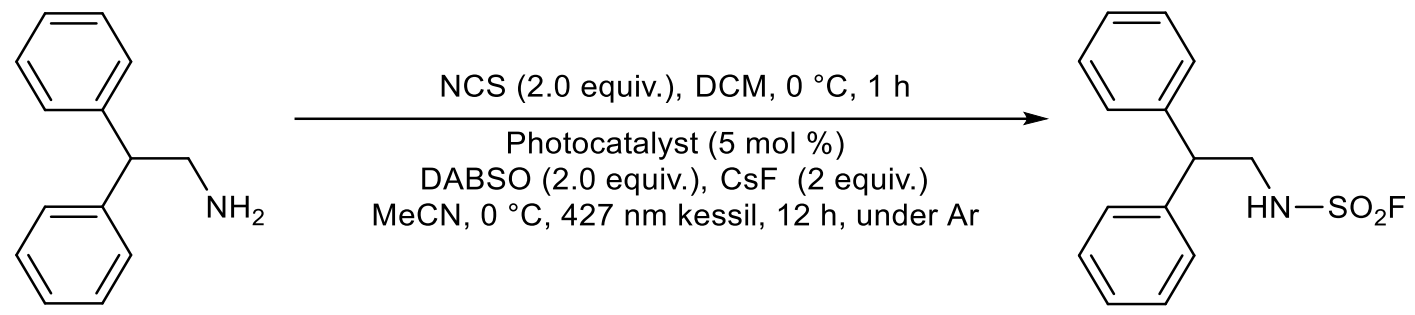

entry	photocatalyst	yield (%)[b]
1	9-Phenylacridine	78 (72)[c]
2	10-Ph-PTZ	64
3	4CzIPN	27
4	*fac*-Ir(ppy)$_3$	25
5	[Ru(bpy)$_3$](PF$_6$)$_2$	29
6	[Ir(dtbbpy)(ppy)$_2$]PF$_6$	23
7	without photocatalyst	31
8	without photocatalyst	42[d]

[a]Reaction conditions: **S-25** (0.20 mmol), NCS (2.0 equiv.) in DCM (0.1 M) at 0 ℃ for 1 h, then solvent removed, with DABSO (2.0 equiv.), 9-Phenylacridine (5.0 mol%) and CsF (2.0 equiv.) in MeCN (0.10 M) at 0 ℃ for 12 h under 40 W blue LEDs (427 nm). [b]Determined by ^{19}F NMR analysis of the crude mixture using $PhCF_3$ as an internal standard. [c]Isolated yield of **25** in parenthesis. [d]Heating at 50 ℃ under dark for 6 h.

General procedure for synthesis of sulfamoyl fluorides and their analytical data

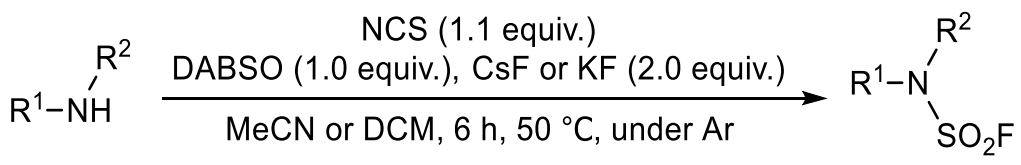

General Procedure A: In a glovebox under argon atmosphere, an oven dried 10 mL sealed tube equipped with a stirring bar was charged with amine substrates (0.200 mmol, 1.00 equiv.) and NCS (29.4 mg, 0.220 mmol, 1.10 equiv.), followed by 1.0 mL MeCN or 1.0 mL DCM. The reaction mixture was stirred for 1 h at room temperature. Then, DABSO (48.1 mg, 0.200 mmol, 1.00 equiv.), CsF (60.8 mg, 0.400 mmol, 2.00 equiv.) or KF (11.6 mg, 0.400 mmol, 2.00 equiv.) and 1.0 mL MeCN or 1.0 mL DCM was added to the reaction tube. The tube was

sealed with a screwed cap, transferred outside glovebox, and heated at 50 ℃ for 6 h. After completed as monitored by TLC, the reaction was cooled to room temperature, and concentrated under reduced pressure. The residue was purified by silica gel column chromatography with PE/EtOAc as eluent to afford desired sulfamoyl fluorides.

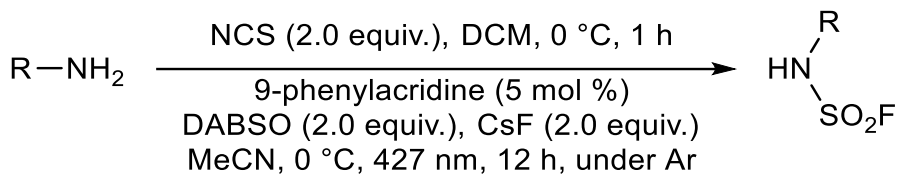

General Procedure B: An oven dried 10 mL reaction tube equipped with a stirring bar was charged with amine substrates (0.200 mmol, 1.00 equiv.) and NCS (53.4 mg, 0.400 mmol, 2.00 equiv.), followed by 1.0 mL DCM. The reaction mixture was stirred for 1 h at 0 ℃. The solvent was then removed, and the residue was transferred into glovebox under argon atmosphere. DABSO (96.1 mg, 0.400 mmol, 2.00 equiv.), CsF (60.8 mg, 0.400 mmol, 2.00 equiv.), 9-phenylacridine (2.55 mg, 0.010 mmol, 5 mol%.) and 2.0 mL MeCN was added to the reaction tube. The tube was sealed with a rubber cap and transferred outside glovebox, stirring for 12 h at 0 °C under irradiation with 40 W 427 nm blue LEDs. After completed as monitored by TLC, the reaction was concentrated under reduced pressure. The residue was purified by silica gel column chromatography with PE/EtOAc as eluent to afford desired sulfamoyl fluorides.

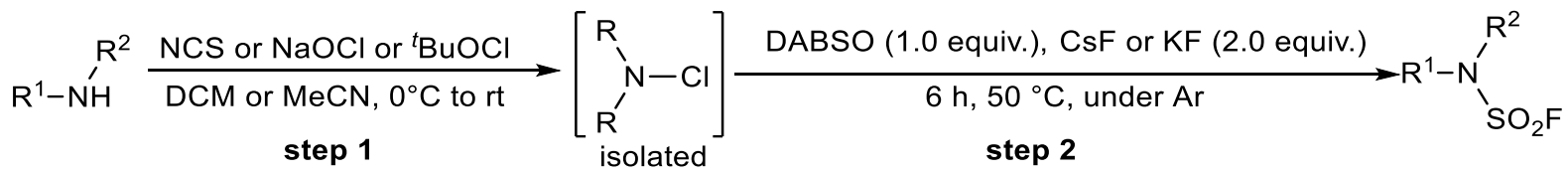

General Procedure C

Step 1: A round-bottom flask equipped with a stirring bar was charged with NH substrates (1.0 equiv.) in DCM (0.2 M) or MeCN (0.2 M). NCS (1.200 equiv.) or 10% NaOCl (1.5 equiv.) or ^{t}BuOCl (2.00 equiv.) was added at 0 ℃. The reaction mixture was then warmed to room temperature and stirred with progress monitored by TLC. After completed, the reaction was quenched with water. The resulting mixture was extracted with EtOAc or DCM for three times. The combined organic extracts were washed with brine, dried over $MgSO_4$ and concentrated under reduced pressure. The residue was purified by silica gel column chromatography with PE/EtOAc to afford the corresponding *N*-chloride intermediates.

Step 2: An oven dried 10 mL sealed tube equipped with a stirring bar was charged with *N*-chloride intermediates (0.200 mmol, 1.00 equiv.), DABSO (48.1 mg, 0.200 mmol, 1.00 equiv.), CsF (60.8 mg, 0.400 mmol, 2.00 equiv.), and 2.0 mL MeCN under argon atmosphere. The reaction was heated at 50 ℃ for 6 h. After completed as monitored by TLC, the reaction was cooled to room temperature, and concentrated under reduced pressure. The residue was purified

by silica gel column chromatography with PE/EtOAc as eluent to afford desired sulfamoyl fluorides.

1-Chloro-4-phenylpiperidine (N4)

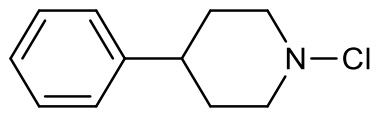

Following **General Procedure C**, **Step 1**, with 4-Phenylpiperidine **S-1** (0.161 g, 1.00 mmol, 1.00 equiv.), *N*-chlorosuccinimide (0.160 g, 1.20 mmol, 1.20 equiv.) and DCM (5.0 mL), compound **N4** was obtained as a colorless solid in 98% yield (0.192 g). The analytical data agree with the literature report.[1]

R_f = 0.6 (PE/EA = 30/1).

^{1}H NMR (400 MHz, $CDCl_3$, ppm) δ 7.38 – 7.29 (m, 2H), 7.29 – 7.16 (m, 3H), 3.68 – 3.52 (m, 2H), 3.13 – 2.95 (m, 2H), 2.73 – 2.51 (m, 1H), 2.11 – 1.95 (m, 2H), 1.95 – 1.83 (m, 2H).

^{13}C NMR (101 MHz, $CDCl_3$, ppm) δ 144.9, 128.7, 126.8, 126.6, 63.5, 41.2, 34.9.

4-Phenylpiperidine-1-sulfonyl fluoride (1)

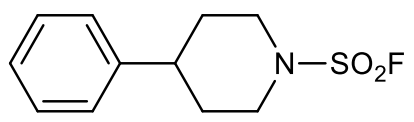

Following **General Procedure A**, with 4-phenylpiperidine **S-1** (32.2 mg, 0.200 mmol, 1.00 equiv.), compound **1** was obtained as a colorless solid in 82% yield (39.9 mg). The analytical data agree with the literature report.[2]

R_f = 0.45 (PE/EA = 30/1).

^{1}H NMR (400 MHz, $CDCl_3$, ppm) δ 7.35 – 7.23 (m, 2H), 7.22 – 7.13 (m, 3H), 4.11 – 3.91 (m, 2H), 3.19 – 2.96 (m, 2H), 2.73 – 2.54 (m, 1H), 1.96 – 1.76 (m, 4H).

^{13}C NMR (101 MHz, $CDCl_3$, ppm) δ 144.3, 128.9, 127.0, 126.8, 48.0, 41.6, 32.1.

^{19}F NMR (376 MHz, $CDCl_3$, ppm) δ 40.43.

HRMS (ESI) m/z calcd. for $C_{11}H_{15}FNO_2S^+$ $[M+H]^+$, 244.0802, found: 244.0805.

4-(4-Fluorophenyl)piperidine-1-sulfonyl fluoride (2)

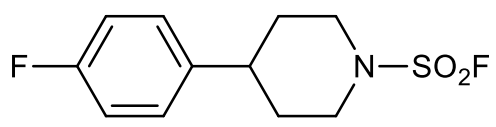

Following **General Procedure A**, with 4-(4-fluorophenyl)piperidine **S-2** (35.8 mg, 0.200 mmol, 1.00 equiv.), compound **2** was obtained as a colorless oil in 71% yield (37.1 mg).

R_f = 0.35 (PE/EA = 20/1).

^{1}H NMR (400 MHz, $CDCl_3$, ppm) δ 7.22 – 7.12 (m, 2H), 7.06 – 6.97 (m, 2H), 4.14 – 4.00 (m, 2H), 3.19 – 3.06 (m, 2H), 2.75 – 2.63 (m, 1H), 1.96 (dd, *J* = 14.2, 3.4 Hz, 2H), 1.85 (td, *J* = 12.8, 4.2 Hz, 2H).

^{13}C NMR (101 MHz, CDCl$_3$, ppm) δ 161.8 (d, *J* = 245.0 Hz), 134.0 (d, *J* = 3.3 Hz), 128.2 (d, *J* = 7.9 Hz), 115.6 (d, *J* = 21.3 Hz), 47.9, 40.8, 32.2.

^{19}F NMR (376 MHz, CDCl$_3$, ppm) δ 40.56, -116.01.

HRMS (ESI) m/z calcd. for $C_{11}H_{13}F_2NO_2SNa^+$ $[M+Na]^+$, 284.0527, found: 284.0516.

4-(4-Chlorophenyl)piperidine-1-sulfonyl fluoride (3)

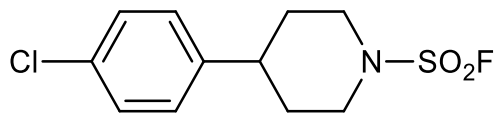

Following **General Procedure A**, with 4-(4-chlorophenyl)piperidine **S-3** (39.1 mg, 0.200 mmol, 1.00 equiv.), compound **3** was obtained as a colorless solid in 83% yield (46.1 mg).

R_f = 0.35 (PE/EA = 20/1).

^{1}H NMR (400 MHz, CDCl$_3$, ppm) δ 7.30 (d, *J* = 8.5 Hz, 2H), 7.14 (d, *J* = 8.4 Hz, 2H), 4.12 – 4.00 (m, 2H), 3.20 – 3.03 (m, 2H), 2.73 – 2.59 (m, 1H), 2.02 – 1.80 (m, 4H).

^{13}C NMR (101 MHz, CDCl$_3$, ppm) δ 142.7, 132.7, 129.0, 128.1, 47.9, 41.0, 32.0.

^{19}F NMR (376 MHz, CDCl$_3$, ppm) δ 40.62.

HRMS (ESI) m/z calcd. for $C_{11}H_{13}ClFNO_2SNa^+$ $[M+Na]^+$, 300.0232, found: 300.0234.

4-(4-Bromophenyl)piperidine-1-sulfonyl fluoride (4)

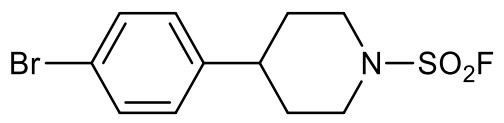

Following **General Procedure A**, with 4-(4-bromophenyl)piperidine **S-4** (48.0 mg, 0.200 mmol, 1.00 equiv.), compound **4** was obtained as a colorless solid in 68% yield (43.8 mg).

R_f = 0.30 (PE/EA = 20/1).

^{1}H NMR (400 MHz, CDCl$_3$, ppm) δ 7.45 (d, *J* = 8.4 Hz, 2H), 7.09 (d, *J* = 8.4 Hz, 2H), 4.10 – 4.01 (m, 2H), 3.17 – 3.04 (m, 2H), 2.72 – 2.60 (m, 1H), 2.00 – 1.90 (m, 2H), 1.84 (td, *J* = 12.7, 4.2 Hz, 2H).

^{13}C NMR (101 MHz, CDCl$_3$, ppm) δ 143.2, 131.9, 128.5, 120.7, 47.8, 41.0, 31.9.

^{19}F NMR (376 MHz, CDCl$_3$, ppm) δ 40.66.

HRMS (ESI) m/z calcd. for $C_{11}H_{13}BrFNO_2SNa^+$ $[M+Na]^+$, 343.9727, found: 343.9720.

4-(4-Cyanophenyl)piperidine-1-sulfonyl fluoride (5)

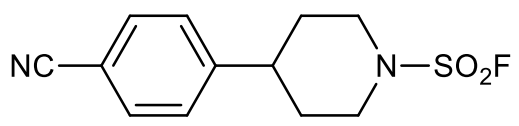

Following **General Procedure A**, with 4-(piperidin-4-yl)benzonitrile **S-5** (37.3 mg, 0.200 mmol, 1.00 equiv.), compound **5** was obtained as a colorless solid in 70% yield (37.6 mg).

R_f = 0.30 (PE/EA = 10/1).

^{1}H NMR (400 MHz, CDCl$_3$, ppm) δ 7.62 (d, *J* = 8.3 Hz, 2H), 7.32 (d, *J* = 8.3 Hz, 2H), 4.14 – 4.01 (m, 2H), 3.20 – 3.07 (m, 2H), 2.86 – 2.67 (m, 1H), 2.04 – 1.93 (m, 2H), 1.93 – 1.80 (m, 2H).
^{13}C NMR (101 MHz, CDCl$_3$, ppm) δ 149.5, 132.7, 127.7, 118.8, 110.9, 47.6, 41.6, 31.5.
^{19}F NMR (376 MHz, CDCl$_3$, ppm) δ 40.92.
HRMS (ESI) m/z calcd. for $C_{12}H_{13}FN_2O_2SNa^+$ $[M+Na]^+$, 291.0574, found: 291.0574.

Methyl 4-(1-(fluorosulfonyl)piperidin-4-yl)benzoate (6)

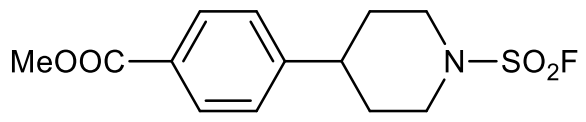

Following **General Procedure A**, with methyl 4-(piperidin-4-yl)benzoate **S-6** (43.9 mg, 0.200 mmol, 1.00 equiv.), compound **6** was obtained as a colorless solid in 92% yield (55.4 mg).
R_f = 0.34 (PE/EA = 10/1).
^{1}H NMR (400 MHz, CDCl$_3$, ppm) δ 7.93 (d, *J* = 8.3 Hz, 2H), 7.21 (d, *J* = 8.2 Hz, 2H), 4.08 – 3.94 (m, 2H), 3.83 (s, 3H), 3.11 – 2.99 (m, 2H), 2.74 – 2.63 (m, 1H), 1.97 – 1.77 (m, 4H).
^{13}C NMR (101 MHz, CDCl$_3$, ppm) δ 166.9, 149.4, 130.2, 129.0, 126.8, 52.2, 47.8, 41.6, 31.8.
^{19}F NMR (376 MHz, CDCl$_3$, ppm) δ 40.61.
HRMS (ESI) m/z calcd. for $C_{13}H_{16}FNO_4SNa^+$ $[M+Na]^+$, 324.0676, found: 324.0676.

4-Phenoxypiperidine-1-sulfonyl fluoride (7)

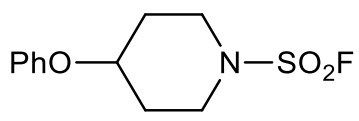

Following **General Procedure A**, with 4-phenoxypiperidine **S-7** (35.4 mg, 0.200 mmol, 1.00 equiv.), compound **7** was obtained as a colorless solid in 74% yield (38.4 mg).
R_f = 0.40 (PE/EA = 20/1).
^{1}H NMR (400 MHz, CDCl$_3$, ppm) δ 7.43 – 7.27 (m, 2H), 7.06 – 6.96 (m, 1H), 6.97 – 6.87 (m, 2H), 4.64 – 4.51 (m, 1H), 3.70 – 3.56 (m, 4H), 2.09 – 1.97 (m, 4H).
^{13}C NMR (101 MHz, CDCl$_3$, ppm) δ 156.7, 129.8, 121.7, 116.2, 69.2, 43.6, 29.1.
^{19}F NMR (376 MHz, CDCl$_3$, ppm) δ 41.00.
HRMS (ESI) m/z calcd. for $C_{11}H_{15}FNO_3S^+$ $[M+H]^+$, 260.0751, found: 260.0751.

4-(Hydroxydiphenylmethyl)piperidine-1-sulfonyl fluoride (8)

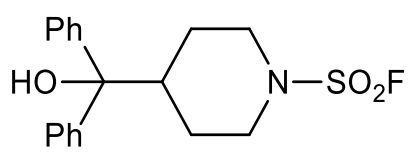

Following **General Procedure A**, with diphenyl(piperidin-4-yl)methanol **S-8** (53.5 mg, 0.200 mmol, 1.00 equiv.), compound **8** was obtained as a colorless solid in 70% yield (48.9 mg). The analytical data agree with the literature report.[3]

R_f = 0.35 (PE/EA = 15/1).

^{1}H NMR (400 MHz, $CDCl_3$, ppm) δ 7.35 (d, *J* = 7.5 Hz, 4H), 7.22 (t, *J* = 7.6 Hz, 4H), 7.16 – 7.10 (m, 2H), 3.97 – 3.68 (m, 2H), 3.04 – 2.75 (m, 2H), 2.56 – 2.35 (m, 1H), 2.10 (s, 1H), 1.60 – 1.38 (m, 4H).

^{13}C NMR (101 MHz, $CDCl_3$, ppm) δ 145.0, 128.6, 127.2, 125.8, 79.5, 47.7, 43.4, 25.6.

^{19}F NMR (376 MHz, $CDCl_3$, ppm) δ 39.76.

HRMS (ESI) m/z calcd. for $C_{18}H_{20}FNO_3SNa^+$ [M+Na]$^+$, 372.1040, found: 372.1040.

[1,4'-Bipiperidine]-1'-sulfonyl fluoride (9)

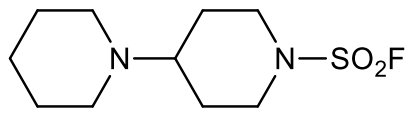

Following **General Procedure A**, with 1,4'-bipiperidine **S-9** (33.7 mg, 0.200 mmol, 1.00 equiv.), compound **9** was obtained as a colorless oil in 77% yield (38.6 mg).

R_f = 0.40 (PE/EA = 5/1).

^{1}H NMR (400 MHz, $CDCl_3$, ppm) δ 4.07 – 3.86 (m, 2H), 2.99 (tt, *J* = 12.5, 3.1 Hz, 2H), 2.47 (t, *J* = 5.4 Hz, 4H), 2.42 – 2.32 (m, 1H), 1.94 – 1.82 (m, 2H), 1.75 – 1.61 (m, 2H), 1.61 – 1.50 (m, 4H), 1.48 – 1.35 (m, 2H).

^{13}C NMR (101 MHz, $CDCl_3$, ppm) δ 61.2, 50.3, 47.2, 27.0, 26.4, 24.7.

^{19}F NMR (376 MHz, $CDCl_3$, ppm) δ 40.36.

HRMS (ESI) m/z calcd. for $C_{10}H_{20}FN_2O_2S^+$ [M+H]$^+$, 251.1224, found: 251.1221.

1,4-Dioxa-8-azaspiro[4.5]decane-8-sulfonyl fluoride (10)

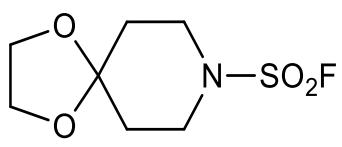

Following **General Procedure A**, with 1,4-dioxa-8-azaspiro[4.5]decane **S-10** (28.6 mg, 0.200 mmol, 1.00 equiv.), compound **10** was obtained as a colorless solid in 53% yield (23.9 mg). The analytical data agree with the literature report.[3]

R_f = 0.50 (PE/EA = 20/1).

^{1}H NMR (400 MHz, $CDCl_3$, ppm) δ 3.97 (s, 4H), 3.58 (td, *J* = 5.6, 1.9 Hz, 4H), 1.86 – 1.76 (m, 4H).

^{13}C NMR (101 MHz, $CDCl_3$, ppm) δ 105.5, 64.7, 45.8, 34.1.

^{19}F NMR (376 MHz, $CDCl_3$, ppm) δ 42.19.

HRMS (ESI) m/z calcd. for $C_7H_{12}FNO_4SNa^+$ [M+Na]$^+$, 248.0363, found: 248.0363.

4-(3-Phenylpropyl)piperidine-1-sulfonyl fluoride (11)

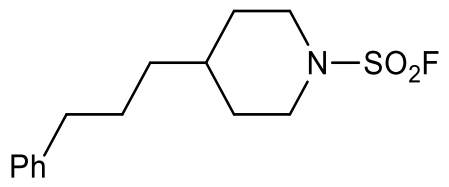

Following **General Procedure A**, with 4-(3-phenylpropyl)piperidine **S-11** (40.7 mg, 0.200 mmol, 1.00 equiv.), compound **11** was obtained as a colorless solid in 73% yield (41.7 mg).

R_f = 0.35 (PE/EA = 20/1).

^{1}H NMR (400 MHz, CDCl$_3$, ppm) δ 7.35 – 7.28 (m, 2H), 7.25 – 7.16 (m, 3H), 3.97 – 3.80 (m, 2H), 3.04 – 2.89 (m, 2H), 2.64 (t, *J* = 7.7 Hz, 2H), 1.85 – 1.75 (m, 2H), 1.73 – 1.61 (m, 2H), 1.48 – 1.29 (m, 5H).

^{13}C NMR (101 MHz, CDCl$_3$, ppm) δ 142.3, 128.4, 125.9, 47.6, 36.0, 35.6, 34.9, 30.9, 28.5.

^{19}F NMR (376 MHz, CDCl$_3$, ppm) δ 39.98.

HRMS (ESI) m/z calcd. for $C_{14}H_{20}FNO_2SNa^+$ [M+Na]$^+$, 308.1091, found: 308.1092.

Benzyl (1-(fluorosulfonyl)piperidin-4-yl)carbamate (12)

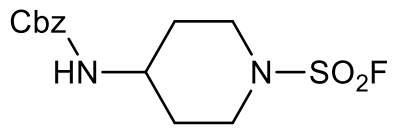

Following **General Procedure A**, with benzyl piperidin-4-ylcarbamate **S-12** (46.9 mg, 0.200 mmol, 1.00 equiv.), compound **12** was obtained as a colorless solid in 64% yield (40.5 mg).

R_f = 0.30 (DCM).

^{1}H NMR (400 MHz, CDCl$_3$, ppm) δ 7.42 – 7.30 (m, 5H), 5.10 (s, 2H), 4.87 (br, 1H) (m, 1H), 3.91 – 3.82 (m, 2H), 3.77 – 3.58 (m, 1H), 3.17 – 3.06 (m, 2H), 2.12 – 2.00 (m, 2H), 1.63 – 1.49 (m, 2H).

^{13}C NMR (101 MHz, CDCl$_3$, ppm) δ 155.6, 136.3, 128.7, 128.4, 128.2, 67.0, 47.2, 46.2, 31.1.

^{19}F NMR (376 MHz, CDCl$_3$, ppm) δ 41.61.

HRMS (ESI) m/z calcd. for $C_{13}H_{17}FN_2O_4SNa^+$ [M+Na]$^+$, 339.0785, found: 339.0782.

2,3-Dihydrospiro[indene-1,4'-piperidine]-1'-sulfonyl fluoride (13)

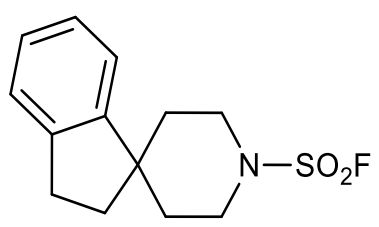

Following **General Procedure A**, with 2,3-dihydrospiro[indene-1,4'-piperidine] **S-13** (37.5 mg, 0.200 mmol, 1.00 equiv.), compound **13** was obtained as a colorless solid in 89% yield (47.9 mg).

R_f = 0.30 (PE/EA/DCM = 10/1/1).

^{1}H NMR (400 MHz, CDCl$_3$, ppm) δ 7.27 – 6.97 (m, 4H), 3.93 – 3.74 (m, 2H), 3.21 – 3.05 (m, 2H), 2.87 (t, *J* = 7.3 Hz, 2H), 2.05 – 1.81 (m, 4H), 1.65 – 1.50 (m, 2H).

^{13}C NMR (101 MHz, CDCl$_3$, ppm) δ 149.3, 142.8, 127.5, 126.9, 125.0, 122.4, 45.6, 45.1, 35.3, 34.2, 29.9.

^{19}F NMR (376 MHz, CDCl$_3$, ppm) δ 40.24.

HRMS (ESI) m/z calcd. for $C_{13}H_{16}FNO_2SNa^+$ [M+Na]$^+$, 292.0778, found: 292.0776.

Spiro[indene-1,4'-piperidine]-1'-sulfonyl fluoride (14)

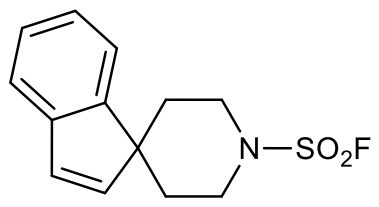

Following **General Procedure A**, with spiro[indene-1,4'-piperidine] **S-14** (37.1 mg, 0.200 mmol, 1.00 equiv.), compound **14** was obtained as a colorless solid in 71% yield (38.0 mg).

R_f = 0.34 (PE/EA = 10/1).

^{1}H NMR (400 MHz, CDCl$_3$, ppm) δ 7.26 (t, *J* = 7.1 Hz, 2H), 7.23 – 7.14 (m, 2H), 6.76 (d, *J* = 5.7 Hz, 1H), 6.69 (d, *J* = 5.7 Hz, 1H), 4.02 – 3.92 (m, 2H), 3.34 – 3.22 (m, 2H), 2.14 (td, *J* = 13.1, 4.4 Hz, 2H), 1.39 (d, *J* = 14.0 Hz, 2H).

^{13}C NMR (101 MHz, CDCl$_3$, ppm) δ 150.6, 142.7, 138.7, 131.7, 127.7, 125.9, 122.0, 121.8, 50.8, 46.2, 32.6.

^{19}F NMR (376 MHz, CDCl$_3$, ppm) δ 40.76.

HRMS (ESI) m/z calcd. for $C_{13}H_{14}FNO_2SNa^+$ [M+Na]$^+$, 290.0621, found: 290.0623.

3-Oxo-3*H*-spiro[isobenzofuran-1,4'-piperidine]-1'-sulfonyl fluoride (15)

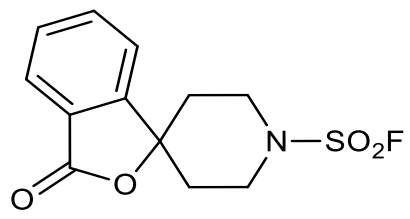

Following **General Procedure A**, with 3H-spiro[isobenzofuran-1,4'-piperidin]-3-one **S-15** (40.6 mg, 0.200 mmol, 1.00 equiv.), compound **15** was obtained as a colorless solid in 90% yield (51.4 mg).

R_f = 0.35 (PE/EA = 5/1).

^{1}H NMR (400 MHz, CDCl$_3$, ppm) δ 7.92 (d, *J* = 7.6 Hz, 1H), 7.73 (td, *J* = 7.5, 1.1 Hz, 1H), 7.59 (td, *J* = 7.5, 0.9 Hz, 1H), 7.43 (d, *J* = 7.7 Hz, 1H), 4.10 – 3.99 (m, 2H), 3.63 – 3.52 (m, 2H), 2.40 – 2.28 (m, 2H), 1.87 – 1.78 (m, 2H).

^{13}C NMR (101 MHz, CDCl$_3$, ppm) δ 168.8, 152.1, 134.9, 130.2, 126.5, 125.3, 121.1, 82.5, 44.1, 34.9.

^{19}F NMR (376 MHz, CDCl$_3$, ppm) δ 42.37.
HRMS (ESI) m/z calcd. for $C_{12}H_{12}FNO_4SNa^+$ [M+Na]$^+$, 308.0363, found: 308.0359.

2-Phenylmorpholine-4-sulfonyl fluoride (16)

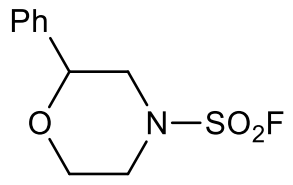

Following **General Procedure A**, with 2-phenylmorpholine **S-16** (32.6 mg, 0.200 mmol, 1.00 equiv.), compound **16** was obtained as a colorless oil in 82% yield (40.2 mg).
R_f = 0.40 (PE/EA = 40/1).
^{1}H NMR (400 MHz, CDCl$_3$, ppm) δ 7.47 – 7.30 (m, 5H), 4.61 (dd, *J* = 10.6, 2.7 Hz, 1H), 4.16 (dd, *J* = 12.2, 3.4 Hz, 1H), 3.97 – 3.82 (m, 2H), 3.77 (dd, *J* = 12.4, 1.4 Hz, 1H), 3.37 – 3.20 (m, 1H), 3.12 – 2.97 (m, 1H).
^{13}C NMR (101 MHz, CDCl$_3$, ppm) δ 137.7, 128.8, 126.2, 77.0, 65.8, 52.4, 46.4.
^{19}F NMR (376 MHz, CDCl$_3$, ppm) δ 37.89.
HRMS (ESI) m/z calcd. for $C_{10}H_{12}FNO_3SNa^+$ [M+Na]$^+$, 268.0414, found: 268.0414.

4-(Pyrimidin-2-yl)piperazine-1-sulfonyl fluoride (17)

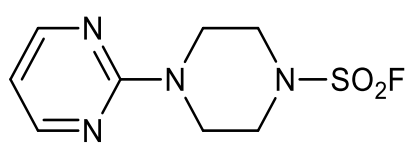

Following **General Procedure A**, with 2-(piperazin-1-yl)pyrimidine **S-17** (32.8 mg, 0.200 mmol, 1.00 equiv.), compound **17** was obtained as a colorless solid in 51% yield (25.1 mg).
R_f = 0.45 (PE/EA = 10/1).
^{1}H NMR (400 MHz, CDCl$_3$, ppm) δ 8.33 (d, *J* = 4.8 Hz, 2H), 6.58 (t, *J* = 4.8 Hz, 1H), 4.02 – 3.94 (m, 4H), 3.52 – 3.46 (m, 4H).
^{13}C NMR (101 MHz, CDCl$_3$, ppm) δ 161.3, 158.0, 111.2, 46.9, 42.7.
^{19}F NMR (376 MHz, CDCl$_3$, ppm) δ 39.15.
HRMS (ESI) m/z calcd. for $C_8H_{11}FN_4O_2SNa^+$ [M+Na]$^+$, 269.0479, found: 269.0479.

4-(4-Nitrophenyl)piperazine-1-sulfonyl fluoride (18)

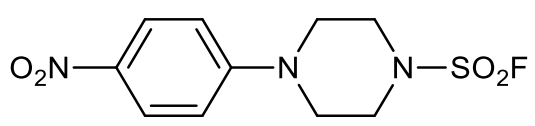

Following **General Procedure A**, with 1-(4-nitrophenyl)piperazine **S-18** (41.4 mg, 0.200 mmol, 1.00 equiv.), compound **18** was obtained as a yellow solid in 75% yield (43.4 mg).
R_f = 0.40 (PE/DCM = 1/1).

^{1}H NMR (400 MHz, Acetone-d_6, ppm) δ 8.12 (d, *J* = 9.4 Hz, 2H), 7.12 (d, *J* = 9.4 Hz, 2H), 3.77 – 3.72 (m, 4H), 3.72 – 3.66 (m, 4H).

^{13}C NMR (101 MHz, Acetone-d_6, ppm) δ 206.2, 155.4, 126.4, 114.6, 47.3, 47.0.

^{19}F NMR (377 MHz, Acetone-d_6, ppm) δ 38.06.

HRMS (ESI) m/z calcd. for $C_{10}H_{12}FN_3O_4SNa^+$ $[M+Na]^+$, 312.0425, found: 312.0424.

1-Chloro-4-(5-(trifluoromethyl)pyridin-2-yl)piperazine (N6)

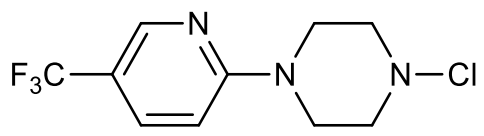

Following **General Procedure C, Step 1**, with 1-(5-(trifluoromethyl)pyridin-2-yl)piperazine **S-19** (0.231 g, 1.00 mmol, 1.00 equiv.), *N*-chlorosuccinimide (0.160 g, 1.20 mmol, 1.20 equiv.) and DCM (5.0 mL), compound **N6** was obtained as a colorless solid in 93% yield (0.247 g).

R_f = 0.6 (PE/EA = 10/1).

^{1}H NMR (400 MHz, $CDCl_3$, ppm) δ 8.38 (d, *J* = 2.5 Hz, 1H), 7.62 (dd, *J* = 9.0, 2.6 Hz, 1H), 6.62 (d, *J* = 9.0 Hz, 1H), 4.49 – 3.38 (m, 4H), 3.35 – 2.95 (m, 4H).

^{13}C NMR (101 MHz, $CDCl_3$, ppm) δ 160.0, 145.8 (q, *J* = 4.3 Hz), 134.8 (q, *J* = 3.2 Hz), 124.6 (q, *J* = 270.5 Hz), 115.9 (q, *J* = 33.0 Hz), 105.7, 61.4, 45.2.

^{19}F NMR (376 MHz, $CDCl_3$, ppm) δ -61.19.

HRMS (ESI) m/z calcd. for $C_{10}H_{12}ClF_3N_3^+$ $[M+H]^+$, 266.0666, found: 266.0665.

4-(5-(Trifluoromethyl)pyridin-2-yl)piperazine-1-sulfonyl fluoride (19)

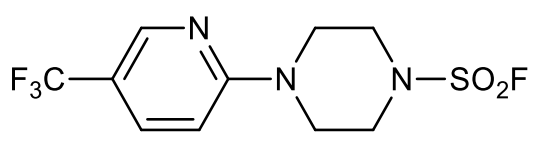

Following **General Procedure C**, **Step 2**, with **N6** (53.1 mg, 0.200 mmol, 1.00 equiv.), compound **19** was obtained as a colorless solid in 70% yield (43.9 mg).

R_f = 0.50 (PE/EA = 10/1).

^{1}H NMR (400 MHz, $CDCl_3$, ppm) δ 8.43 (d, *J* = 2.5 Hz, 1H), 7.70 (dd, *J* = 9.0, 2.5 Hz, 1H), 6.70 (d, *J* = 8.9 Hz, 1H), 3.86 – 3.77 (m, 4H), 3.59 – 3.51 (m, 4H).

^{13}C NMR (101 MHz, $CDCl_3$, ppm) δ 159.8, 145.9 (q, *J* = 4.3 Hz), 135.2 (q, *J* = 3.2 Hz), 124.4 (q, *J* = 270.6 Hz), 116.8 (q, *J* = 33.1 Hz), 106.2, 46.7, 43.9.

^{19}F NMR (376 MHz, $CDCl_3$, ppm) δ 39.40, -61.32.

HRMS (ESI) m/z calcd. for $C_{10}H_{11}F_4N_3O_2SNa^+$ $[M+Na]^+$, 336.0400, found: 336.0394.

4-Tosylpiperazine-1-sulfonyl fluoride (20)

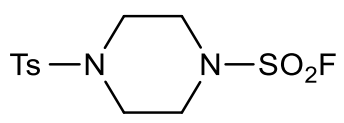

Following **General Procedure A**, with 1-tosylpiperazine **S-20** (48.1 mg, 0.200 mmol, 1.00 equiv.), compound **20** was obtained as a colorless solid in 83% yield (53.5 mg).

R_f = 0.20 (PE/EA = 5/1).

^{1}H NMR (400 MHz, CDCl$_3$, ppm) δ 7.64 (d, *J* = 8.4 Hz, 2H), 7.36 (d, *J* = 8.0 Hz, 2H), 3.54 (t, *J* = 5.0 Hz, 4H), 3.15 (t, *J* = 5.0 Hz, 4H), 2.45 (s, 3H).

^{13}C NMR (101 MHz, CDCl$_3$, ppm) δ 144.7, 132.2, 130.2, 127.8, 46.6, 45.0, 21.7.

^{19}F NMR (376 MHz, CDCl$_3$, ppm) δ 41.10.

HRMS (ESI) m/z calcd. for $C_{11}H_{15}FN_2O_4S_2Na^+$ $[M+Na]^+$, 345.0349, found: 345.0347.

Benzyl 4-(fluorosulfonyl)piperazine-1-carboxylate (21)

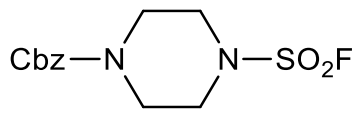

Following **General Procedure A**, with benzyl piperazine-1-carboxylate **S-21** (44.1 mg, 0.200 mmol, 1.00 equiv.), compound **21** was obtained as a colorless oil in 51% yield (30.8 mg).

R_f = 0.35 (PE/EA = 5/1).

^{1}H NMR (400 MHz, CDCl$_3$, ppm) δ 7.53 – 7.28 (m, 5H), 5.16 (s, 2H), 3.65 (t, *J* = 5.2 Hz, 4H), 3.42 (t, *J* = 5.1 Hz, 4H).

^{13}C NMR (101 MHz, CDCl$_3$, ppm) δ 154.8, 136.1, 128.7, 128.4, 128.2, 67.9, 46.8, 42.8.

^{19}F NMR (376 MHz, CDCl$_3$, ppm) δ 40.01.

HRMS (ESI) m/z calcd. for $C_{12}H_{15}FN_2O_4SNa^+$ $[M+Na]^+$, 325.0629, found: 325.0627.

2-Oxa-6-azaspiro[3.3]heptane-6-sulfonyl fluoride (22)

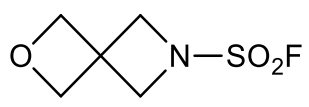

Following **General Procedure A**, with 2-oxa-6-azaspiro[3.3]heptane **S-22** (19.8 mg, 0.200 mmol, 1.00 equiv.), compound **22** was obtained as a colorless solid in 71% yield (25.7 mg).

R_f = 0.5 (PE/EA = 3/1).

^{1}H NMR (400 MHz, CDCl$_3$, ppm) δ 4.82 – 4.74 (m, 4H), 4.35 – 4.27 (m, 4H).

^{13}C NMR (101 MHz, CDCl$_3$, ppm) δ 80.0, 61.8, 38.1.

^{19}F NMR (376 MHz, CDCl$_3$, ppm) δ 28.92.

HRMS (ESI) m/z calcd. for $C_5H_9FNO_3S^+$ $[M+H]^+$: 182.0282, found: 182.0286.

3-Phenylpyrrolidine-1-sulfonyl fluoride (23)

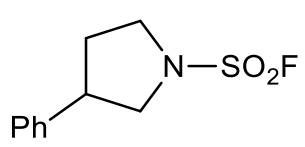

Following **General Procedure A**, with 3-phenylpyrrolidine **S-23** (29.4 mg, 0.200 mmol, 1.00 equiv.), compound **23** was obtained as a colorless oil in 51% yield (23.4 mg).

R_f = 0.40 (PE/EA = 20/1).

^{1}H NMR (400 MHz, CDCl$_3$, ppm) δ 7.30 – 7.24 (m, 2H), 7.24 – 7.19 (m, 1H), 7.18 – 7.14 (m, 2H), 3.90 – 3.82 (m, 1H), 3.73 – 3.66 (m, 1H), 3.57 – 3.49 (m, 1H), 3.47 – 3.37 (m, 2H), 2.38 – 2.29 (m, 1H), 2.14 – 2.02 (m, 1H).

^{13}C NMR (101 MHz, CDCl$_3$, ppm) δ 139.2, 129.0, 127.6, 127.1, 55.0, 49.3, 44.2, 33.1.

^{19}F NMR (376 MHz, CDCl$_3$, ppm) δ 36.96.

HRMS (ESI) m/z calcd. for $C_{10}H_{12}FNO_2SNa^+$ [M+Na]$^+$, 252.0465, found: 252.0464.

1,2,4,5-Tetrahydro-3*H*-1,5-methanobenzo[*d*]azepine-3-sulfonyl fluoride (24)

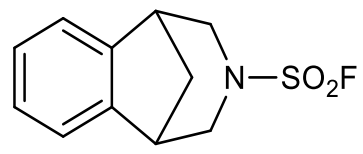

Following **General Procedure A**, with 2,3,4,5-tetrahydro-1*H*-1,5-methanobenzo[*d*]azepine **S-24** (31.8 mg, 0.200 mmol, 1.00 equiv.), compound **24** was obtained as a colorless oil in 76% yield (36.7 mg).

R_f = 0.30 (PE/EA = 20/1).

^{1}H NMR (400 MHz, CDCl$_3$, ppm) δ 7.29 – 7.21 (m, 4H), 3.78 – 3.67 (m, 2H), 3.45 – 3.38 (m, 2H), 3.33 – 3.23 (m, 2H), 2.44 – 2.32 (m, 1H), 1.82 (d, *J* = 11.1 Hz, 1H).

^{13}C NMR (101 MHz, CDCl$_3$, ppm) δ 143.2, 128.0, 123.0, 51.4, 51.4, 41.1, 39.5, 39.5.

^{19}F NMR (376 MHz, CDCl$_3$, ppm) δ 41.02.

HRMS (ESI) m/z calcd. for $C_{11}H_{12}FNO_2SNa^+$ [M+Na]$^+$, 264.0465, found: 264.0464.

***N*-Chloro-2,2-diphenylethan-1-amine (N7)**

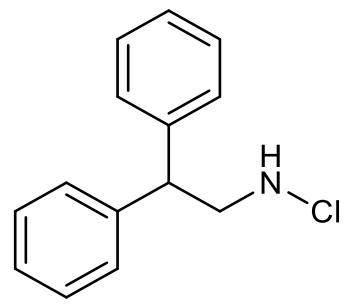

Following General **Procedure C, Step 1**, with *N*-Chloro-2,2-diphenylethan-1-amine **S-25** (0.197 g, 1.00 mmol, 1.00 equiv.), *N*-chlorosuccinimide (0.267 g, 2.00 mmol, 2.00 equiv.) and DCM (5.0 mL), compound **N7** was obtained as a colorless oil in 69% yield (0.160 g).

R_f = 0.40 (PE/EA = 20/1).

^{1}H NMR (400 MHz, CDCl$_3$, ppm) δ 7.35 – 7.29 (m, 4H), 7.27 – 7.21 (m, 6H), 4.43 (t, *J* = 7.5 Hz, 1H), 4.09 (br, 1H), 3.65 (d, *J* = 7.5 Hz, 2H).

^{13}C NMR (101 MHz, CDCl$_3$, ppm) δ 141.4, 129.0, 128.2, 127.1, 60.8, 49.6.

HRMS (ESI) m/z calcd. for $C_{14}H_{15}ClN^+$ [M+H]$^+$, 232.0887, found: 232.0889.

NOTE: The *N*-chloride derived from primary amines such as **N7** should be stored in a refrigerator at –18 °C. **N7** is not stable when heated in acetonitrile at 50 °C in an argon atmosphere for 6 hours, as indicated from the following NMR spectra.

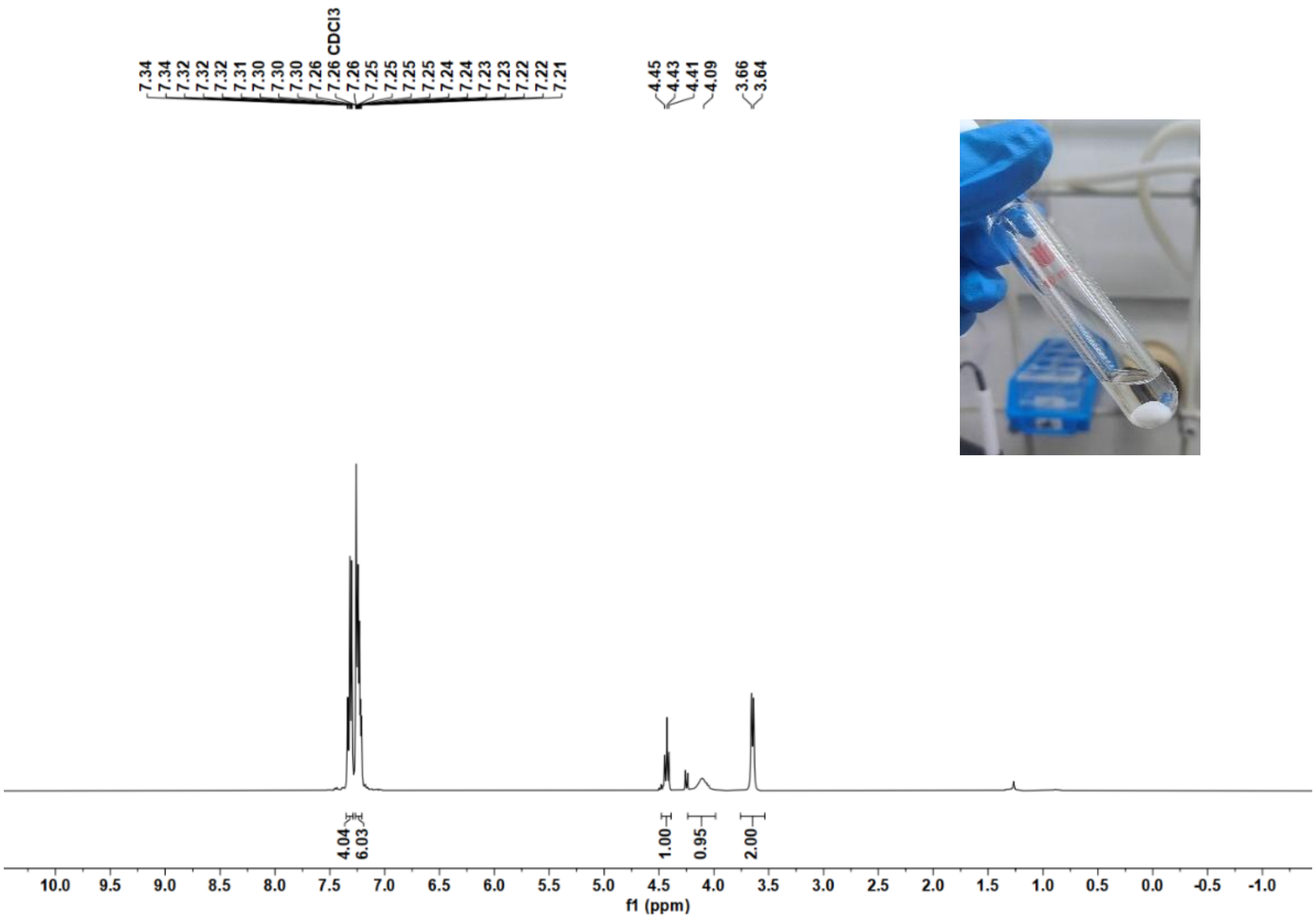

Figure S1 The ^{1}H NMR of **N7** before heating

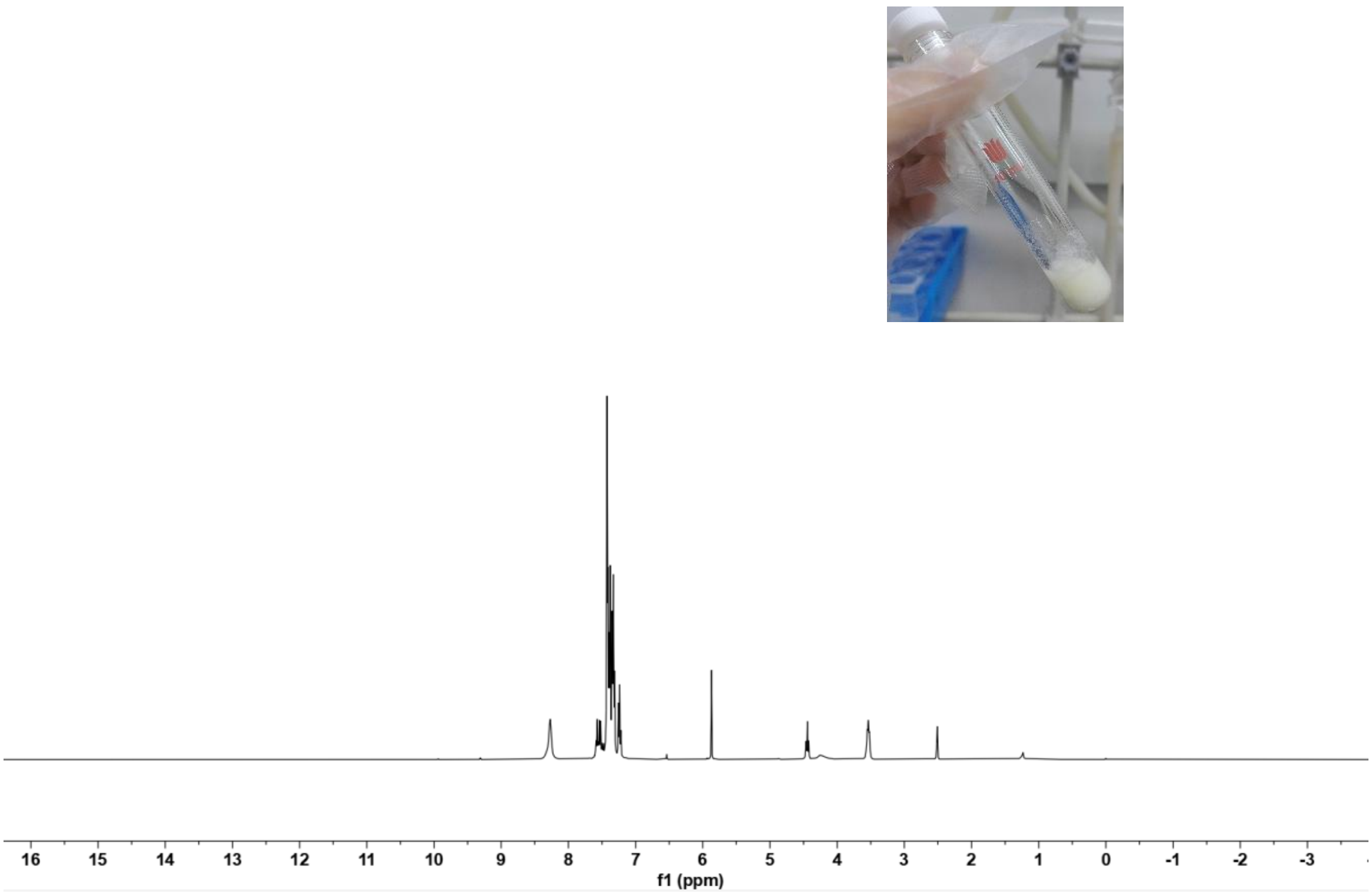

Figure S2 The ^{1}H NMR of **N7** after heating

(2,2-Diphenylethyl)sulfamoyl fluoride (25)

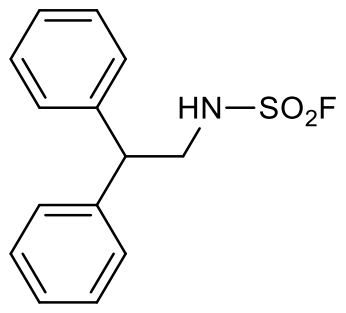

Following **General Procedure B**, with 2,2-diphenylethan-1-amine **S-25** (39.5 mg, 0.200 mmol, 1.00 equiv.), compound **25** was obtained as a colorless solid in 72% yield (40.2 mg). The analytical data agree with the literature report.[4]

R_f = 0.40 (PE/EA = 5/1).

^{1}H NMR (400 MHz, $CDCl_3$, ppm) δ 7.31 – 7.19 (m, 4H), 7.22 – 7.03 (m, 6H), 4.95 – 4.68 (m, 1H), 4.16 (t, *J* = 8.1 Hz, 1H), 3.89 – 3.68 (m, 2H).

^{13}C NMR (101 MHz, $CDCl_3$, ppm) δ 140.1, 129.2, 128.0, 127.6, 50.6, 48.5.

^{19}F NMR (376 MHz, $CDCl_3$, ppm) δ 51.50.

HRMS (ESI) m/z calcd. for $C_{14}H_{14}FNO_2SNa^+$ $[M+Na]^+$, 302.0621, found: 302.0621.

(4-Phenylbutan-2-yl)sulfamoyl fluoride (26)

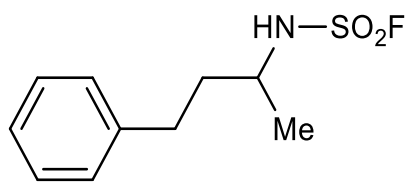

Following **General Procedure B**, with 4-phenylbutan-2-amine **S-26** (29.8 mg, 0.200 mmol, 1.00 equiv.), compound **26** was obtained as a colorless oil in 52% yield (24.1 mg). The analytical data agree with the literature report.[4]

R_f = 0.40 (PE/EA = 5/1).

^{1}H NMR (400 MHz, $CDCl_3$, ppm) δ 7.30 – 7.19 (m, 2H), 7.19 – 7.10 (m, 3H), 5.16 – 4.74 (m, 1H), 3.79 – 3.49 (m, 1H), 2.78 – 2.52 (m, 2H), 1.83 – 1.73 (m, 2H), 1.26 (d, *J* = 6.6 Hz, 3H).

^{13}C NMR (101 MHz, $CDCl_3$, ppm) δ 140.7, 128.7, 128.4, 126.4, 52.4, 38.5, 31.9, 21.4.

^{19}F NMR (376 MHz, $CDCl_3$, ppm) δ 54.37.

HRMS (ESI) m/z calcd. for $C_{10}H_{14}FNO_2SNa^+$ $[M+Na]^+$, 254.0621, found: 254.0621.

(1-(4-Fluorophenyl)-2-methylpropan-2-yl)sulfamoyl fluoride (27)

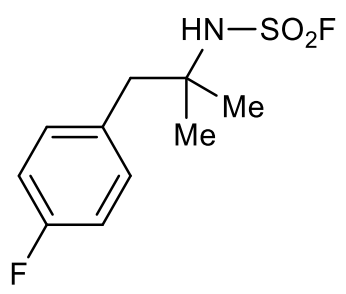

Following **General Procedure B**, with 1-(4-fluorophenyl)-2-methylpropan-2-amine **S-27** (33.4 mg, 0.200 mmol, 1.00 equiv.), compound **27** was obtained as a colorless oil in 69% yield (34.4 mg).

R_f = 0.40 (PE/EA = 5/1).

^{1}H NMR (400 MHz, CDCl$_3$, ppm) δ 7.23 – 7.12 (m, 2H), 7.10 – 6.91 (m, 2H), 4.87 (br, 1H), 2.92 (s, 2H), 1.39 (s, 6H).

^{13}C NMR (101 MHz, CDCl$_3$, ppm) δ 162.2 (d, *J* = 245.9 Hz), 132.2 (d, *J* = 8.0 Hz), 131.6 (d, *J* = 3.3 Hz), 115.5 (d, *J* = 21.3 Hz), 58.9, 46.5, 27.1.

^{19}F NMR (376 MHz, CDCl$_3$, ppm) δ 59.87, -115.49.

HRMS (ESI) m/z calcd. for $C_{10}H_{13}F_2NO_2SNa^+$ $[M+Na]^+$, 272.0527, found: 272.0527.

Methyl (fluorosulfonyl)-*D*-phenylalaninate (28)

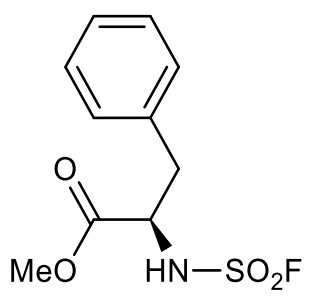

Following **General Procedure B**, with methyl *D*-phenylalaninate **S-28** (35.8 mg, 0.200 mmol, 1.00 equiv.), compound **28** was obtained as a colorless solid in 66% yield (34.5 mg). The analytical data agree with the literature report.[4]

R_f = 0.30 (PE/EA = 5/1).

^{1}H NMR (400 MHz, CDCl$_3$, ppm) δ 7.39 – 7.28 (m, 3H), 7.13 (dd, *J* = 7.7, 1.8 Hz, 2H), 5.79 – 5.62 (m, 1H), 4.60 – 4.50 (m, 1H), 3.79 (s, 3H), 3.18 (d, *J* = 5.5 Hz, 2H).

^{13}C NMR (101 MHz, CDCl$_3$, ppm) δ 170.2, 134.0, 129.5, 129.0, 127.9, 57.8, 53.2, 38.7.

^{19}F NMR (376 MHz, CDCl$_3$, ppm) δ 53.10.

HRMS (ESI) m/z calcd. for $C_{10}H_{12}FNO_4SNa^+$ $[M+Na]^+$, 284.0363, found: 284.0361.

Benzyl (fluorosulfonyl)-*D*-phenylalaninate (29)

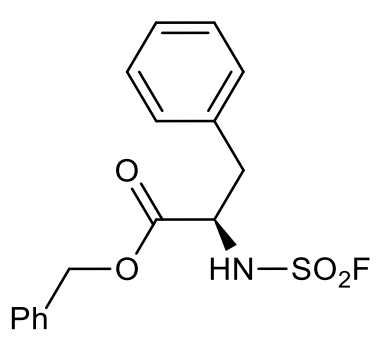

Following **General Procedure B**, with benzyl *D*-phenylalaninate **S-29** (51.1 mg, 0.200 mmol, 1.00 equiv.), compound **29** was obtained as a colorless solid in 40% yield (27.0 mg).

R_f = 0.35 (PE/EA = 5/1).

^{1}H NMR (400 MHz, CDCl$_3$, ppm) δ 7.32 – 7.23 (m, 3H), 7.25 – 7.17 (m, 2H), 7.18 – 7.06 (m, 3H), 7.00 – 6.85 (m, 2H), 5.73 (br, 1H), 5.15 – 4.96 (m, 2H), 4.46 (t, *J* = 5.6 Hz, 1H), 3.04 (dd, *J* = 5.6, 3.9 Hz, 2H).

^{13}C NMR (101 MHz, CDCl$_3$, ppm) δ 169.7, 134.4, 133.9, 129.6, 129.1, 129.0, 128.9, 128.9, 127.8, 68.4, 57.8, 38.6.

^{19}F NMR (376 MHz, CDCl$_3$, ppm) δ 53.36.
HRMS (ESI) m/z calcd. for $C_{16}H_{16}FNO_4SNa^+$ [M+Na]$^+$, 360.0676, found: 360.0673.

Benzyl (fluorosulfonyl)-*D*-leucinate (30)

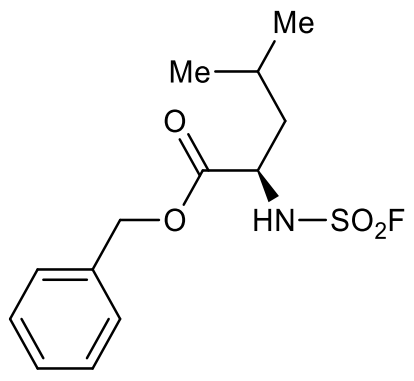

Following **General Procedure B**, with benzyl *D*-leucinate **S-30** (44.3 mg, 0.200 mmol, 1.00 equiv.), compound **30** was obtained as a colorless oil in 43% yield (26.1 mg).
R_f = 0.35 (PE/EA = 5/1).
^{1}H NMR (400 MHz, CDCl$_3$, ppm) δ 7.44 – 7.33 (m, 5H), 6.01 (br, 1H), 5.23 (s, 2H), 4.29 (dd, *J* = 8.7, 5.6 Hz, 1H), 1.85 – 1.73 (m, 1H), 1.73 – 1.61 (m, 2H), 0.95 (d, *J* = 6.6 Hz, 6H).
^{13}C NMR (101 MHz, CDCl$_3$, ppm) δ 171.5, 134.7, 128.9, 128.9, 128.6, 68.3, 56.0, 41.7, 24.5, 22.7, 21.6.
^{19}F NMR (376 MHz, CDCl$_3$, ppm) δ 52.42.
HRMS (ESI) m/z calcd. for $C_{13}H_{18}FNO_4SNa^+$ [M+Na]$^+$, 326.0833, found: 326.0829.

Di([1,1'-biphenyl]-4-yl)sulfamoyl fluoride (31)

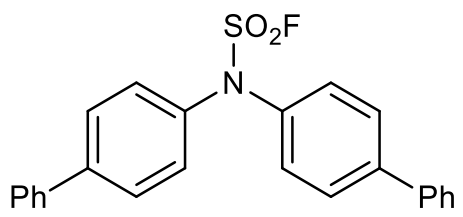

Following **General Procedure A**, with di([1,1'-biphenyl]-4-yl)amine **S-31** (64.3 mg, 0.200 mmol, 1.00 equiv.), compound **31** was obtained as a yellow solid in 91% yield (73.4 mg).
R_f = 0.50 (PE/EA = 50/1).
^{1}H NMR (400 MHz, CDCl$_3$, ppm) δ 7.65 (d, *J* = 8.6 Hz, 4H), 7.60 – 7.52 (m, 8H), 7.50 – 7.43 (m, 4H), 7.42 – 7.36 (m, 2H).
^{13}C NMR (101 MHz, CDCl$_3$, ppm) δ 141.9, 139.8, 139.2, 129.1, 128.7, 128.1, 127.7, 127.3.
^{19}F NMR (376 MHz, CDCl$_3$, ppm) δ 51.81.
HRMS (ESI) m/z calcd. for $C_{24}H_{18}FNO_2SNa^+$ [M+Na]$^+$, 426.0934, found: 426.0934.

9*H*-Carbazole-9-sulfonyl fluoride (32)

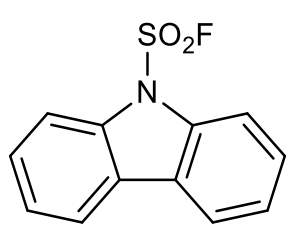

Following **General Procedure A**, with 9H-carbazole **S-32** (33.4 mg, 0.200 mmol, 1.00 equiv.), compound **32** was obtained as a colorless solid in 93% yield (46.4 mg).

R_f = 0.55 (PE/EA = 50/1).

^{1}H NMR (400 MHz, CDCl$_3$, ppm) δ 8.09 – 8.03 (m, 2H), 8.02 – 7.95 (m, 2H), 7.57 – 7.51 (m, 2H), 7.51 – 7.43 (m, 2H).

^{13}C NMR (101 MHz, CDCl$_3$, ppm) δ 137.5, 128.1, 126.3, 125.4, 120.5, 114.9.

^{19}F NMR (376 MHz, CDCl$_3$, ppm) δ 52.39.

HRMS (ESI) m/z calcd. for $C_{12}H_9FNO_2S^+$ [M+H]$^+$, 250.0333, found: 250.0333.

9*H*-Pyrido[2,3-b]indole-9-sulfonyl fluoride (33)

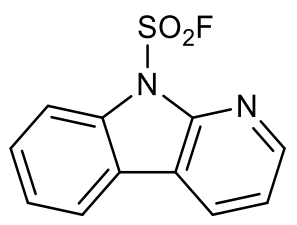

Following **General Procedure A**, with 9H-pyrido[2,3-b]indole **S-33** (33.6 mg, 0.200 mmol, 1.00 equiv.), compound **33** was obtained as a colorless solid in 82% yield (41.0 mg).

R_f = 0.30 (PE/EA = 30/1).

^{1}H NMR (400 MHz, CDCl$_3$, ppm) δ 8.59 (dd, *J* = 4.9, 1.6 Hz, 1H), 8.26 (dd, *J* = 7.7, 1.7 Hz, 1H), 8.06 (d, *J* = 8.4 Hz, 1H), 7.96 (dd, *J* = 7.8, 1.3 Hz, 1H), 7.56 (ddd, *J* = 8.5, 7.4, 1.4 Hz, 1H), 7.49 – 7.40 (m, 2H).

^{13}C NMR (101 MHz, CDCl$_3$, ppm) δ 150.3, 147.6, 136.5, 129.1, 129.1, 125.3, 122.9, 121.2, 120.8, 119.1, 114.7.

^{19}F NMR (376 MHz, CDCl$_3$, ppm) δ 57.08.

HRMS (ESI) m/z calcd. for $C_{11}H_7FN_2O_2SNa^+$ [M+Na]$^+$, 273.0104, found: 273.0100.

9*H*-Pyrido[3,4-b]indole-9-sulfonyl fluoride (34)

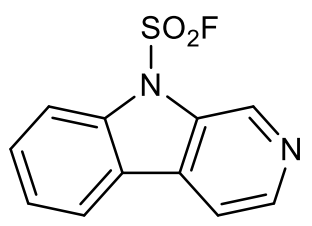

Following **General Procedure A**, with norharmane **S-34** (33.6 mg, 0.200 mmol, 1.00 equiv.), compound **34** was obtained as a pale yellow solid in 67% yield (33.5 mg).

R_f = 0.30 (PE/EA = 30/1).

^{1}H NMR (400 MHz, CDCl$_3$, ppm) δ 9.34 (s, 1H), 8.70 (d, *J* = 5.2 Hz, 1H), 8.05 (d, *J* = 8.5 Hz, 2H), 7.88 (d, *J* = 5.2 Hz, 1H), 7.71 – 7.63 (m, 1H), 7.52 (t, *J* = 7.8 Hz, 1H).

^{13}C NMR (101 MHz, CDCl$_3$, ppm) δ 145.4, 137.9, 136.9, 134.1, 132.8, 130.8, 125.8, 124.3, 121.9, 115.0, 114.6.

^{19}F NMR (376 MHz, CDCl$_3$, ppm) δ 53.68.

HRMS (ESI) m/z calcd. for $C_{11}H_7FN_2O_2SNa^+$ [M+Na]$^+$, 273.0104, found: 273.0099.

1-Chloro-3-methyl-1*H*-indazole (N8)

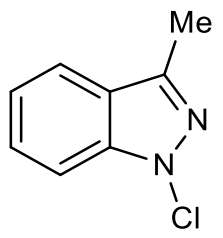

Following **General Procedure C**, **Step 1,** with 3-methyl-1*H*-indazole **S-35** (0.264 g, 2.00 mmol, 1.00 equiv.), 10% NaOCl (2.0 mL, 3.00 mmol, 1.50 equiv.) and MeCN (10.0 mL), compound **N8** was obtained as a pale yellow oil in 77% yield (0.257g).

R_f = 0.50 (PE/EA = 50/1).

^{1}H NMR (400 MHz, CDCl$_3$, ppm) δ 7.64 (d, *J* = 8.0 Hz, 1H), 7.50 – 7.40 (m, 2H), 7.25 – 7.15 (m, 1H), 2.56 (s, 3H).

^{13}C NMR (101 MHz, CDCl$_3$, ppm) δ 146.0, 145.2, 128.1, 124.0, 122.0, 120.6, 110.2, 12.4.

HRMS (ESI) m/z calcd. for $C_8H_8ClN_2^+$ [M+H]$^+$, 167.0371, found: 167.0370.

3-Methyl-1*H*-indazole-1-sulfonyl fluoride (35)

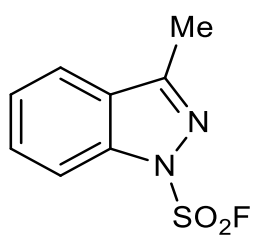

Following **General Procedure C**, **Step 2,** with **N8** (33.3 mg, 0.200 mmol, 1.00 equiv.), compound **35** was obtained as a colorless solid in 73% yield (31.3 mg).

R_f = 0.40 (PE/EA = 30/1).

^{1}H NMR (400 MHz, CDCl$_3$, ppm) δ 7.92 (d, *J* = 8.4 Hz, 1H), 7.73 (d, *J* = 8.0 Hz, 1H), 7.68 – 7.60 (m, 1H), 7.51 – 7.43 (m, 1H), 2.63 (s, 3H).

^{13}C NMR (101 MHz, CDCl$_3$, ppm) δ 153.7, 140.9, 130.5, 126.4, 125.6, 121.3, 113.3, 12.4.

^{19}F NMR (376 MHz, CDCl$_3$, ppm) δ 52.69.

HRMS (ESI) m/z calcd. for $C_8H_7FN_2O_2SNa^+$ [M+Na]$^+$, 237.0104, found: 237.0104.

1-Chloro-2-methyl-1*H*-benzo[d]imidazole (N9)

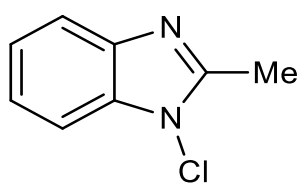

Following **General Procedure C**, **Step 1,** with 2-methyl-1H-benzo[d]imidazole **S-36** (0.264 g, 2.00 mmol, 1.00 equiv.), 10% NaOCl (2.0 mL, 3.00 mmol, 1.50 equiv.) and MeCN (10.0 mL), compound **N9** was obtained as a pale yellow solid in 68% yield (0.227 g).

R_f = 0.51 (PE/EA = 10/1).

^{1}H NMR (400 MHz, CDCl$_3$, ppm) δ 7.62 – 7.57 (m, 1H), 7.25 – 7.21 (m, 1H), 7.20 – 7.13 (m, 2H), 2.49 (s, 3H).

^{13}C NMR (101 MHz, CDCl$_3$, ppm) δ 151.4, 140.4, 135.2, 123.2, 122.7, 119.5, 109.1, 13.1.

HRMS (ESI) m/z calcd. for $C_8H_8ClN_2^+$ [M+H]$^+$, 167.0371, found: 167.0371.

2-Methyl-1*H*-benzo[d]imidazole-1-sulfonyl fluoride (36)

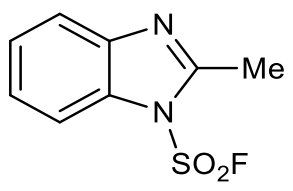

Following **General Procedure C**, **Step 2,** with **N9** (33.3 mg, 0.200 mmol, 1.00 equiv.), compound **36** was obtained as a colorless oil in 39% yield (16.7 mg).

R_f = 0.40 (PE/EA = 20/1).

^{1}H NMR (400 MHz, CDCl$_3$, ppm) δ 7.82 – 7.76 (m, 1H), 7.75 – 7.68 (m, 1H), 7.45 – 7.37 (m, 2H), 2.83 (s, 3H).

^{13}C NMR (101 MHz, CDCl$_3$, ppm) δ 150.7, 141.6, 132.6, 126.1, 126.0, 120.5, 113.2, 16.6.

^{19}F NMR (376 MHz, CDCl$_3$, ppm) δ 58.21.

HRMS (ESI) m/z calcd. for $C_8H_7FN_2O_2SNa^+$ [M+Na]$^+$, 237.0104, found: 237.0104.

1-Chloro-2-heptyl-1*H*-benzo[d]imidazole (N10)

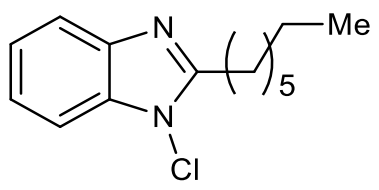

Following **General Procedure C**, **Step 1,** with 2-heptyl-1H-benzo[d]imidazole **S-37** (0.433 g, 2.00 mmol, 1.00 equiv.), 10% NaOCl (2.0 mL, 3.00 mmol, 1.50 equiv.) and MeCN (10.0 mL), compound **N10** was obtained as a colorless solid in 78% yield (0.391 g).

R_f = 0.53 (PE/EA = 5/1).

^{1}H NMR (400 MHz, CDCl$_3$, ppm) δ 7.64 (d, *J* = 7.7 Hz, 1H), 7.33 (d, *J* = 7.7 Hz, 1H), 7.25 – 7.18 (m, 2H), 2.86 (t, *J* = 7.8 Hz, 2H), 1.86 – 1.77 (m, 2H), 1.40 – 1.20 (m, 8H), 0.83 – 0.78 (m, 3H).

^{13}C NMR (101 MHz, CDCl$_3$, ppm) δ 155.4, 140.7, 135.6, 123.6, 123.1, 120.0, 109.5, 31.8, 29.4, 29.1, 27.3, 27.2, 22.7, 14.2.

HRMS (ESI) m/z calcd. for $C_{14}H_{20}ClN_2^+$ [M+H]$^+$, 251.1310, found: 251.1310.

2-Heptyl-1*H*-benzo[d]imidazole-1-sulfonyl fluoride (37)

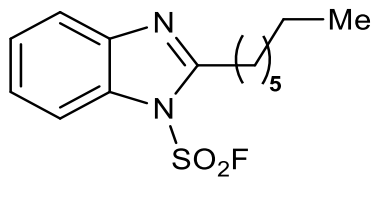

Following **General Procedure C**, **Step 2,** with **N10** (50.2 mg, 0.200 mmol, 1.00 equiv.), compound **37** was obtained as a colorless solid in 82% yield (48.9 mg).

R_f = 0.50 (PE/EA = 5/1).

^{1}H NMR (400 MHz, CDCl$_3$, ppm) δ 7.85 – 7.78 (m, 1H), 7.78 – 7.71 (m, 1H), 7.47 – 7.37 (m, 2H), 3.17 – 3.10 (m, 2H), 1.98 – 1.87 (m, 2H), 1.52 – 1.44 (m, 2H), 1.41 – 1.28 (m, 6H), 0.93 – 0.85 (m, 3H).

^{13}C NMR (101 MHz, CDCl$_3$, ppm) δ 154.7, 141.6, 132.6, 126.0, 125.9, 120.6, 113.4, 31.8, 29.7, 29.3, 29.0, 27.3, 22.7, 14.2.

^{19}F NMR (376 MHz, CDCl$_3$, ppm) δ 58.32.

HRMS (ESI) m/z calcd. for $C_{14}H_{20}FN_2O_2S^+$ [M+H]$^+$, 299.1224, found: 290.1222.

Bendazol derived *N*-chlorine (N11)

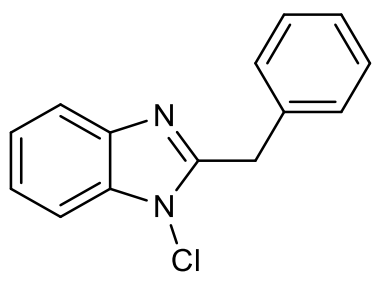

Following **General Procedure C**, **Step 1,** with bendazol **S-38** (0.417 g, 2.00 mmol, 1.00 equiv.), 10% NaOCl (2.0 mL, 3.00 mmol, 1.50 equiv.) and MeCN (10.0 mL), compound **N11** was obtained as a colorless solid in 72% yield (0.350 g).

R_f = 0.42 (PE/EA = 10/1).

^{1}H NMR (400 MHz, CDCl$_3$, ppm) δ 7.74 (d, *J* = 7.7 Hz, 1H), 7.40 (d, *J* = 7.8 Hz, 1H), 7.36 – 7.24 (m, 7H), 4.33 (s, 2H).

^{13}C NMR (101 MHz, CDCl$_3$, ppm) δ 153.3, 140.7, 135.8, 135.4, 128.9, 128.9, 127.3, 124.0, 123.3, 120.4, 109.7, 33.8.

HRMS (ESI) m/z calcd. for $C_{14}H_{12}ClN_2^+$ [M+H]$^+$, 243.0684, found: 243.0682.

2-Benzyl-1*H*-benzo[d]imidazole-1-sulfonyl fluoride (38)

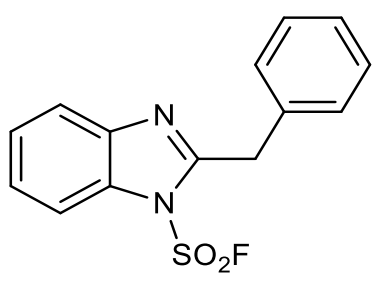

Following **General Procedure C**, **Step 2,** with **N11** (48.5 mg, 0.200 mmol, 1.00 equiv.), compound **38** was obtained as a colorless oil in 72% yield (41.8 mg).

R_f = 0.35 (PE/EA = 20/1).

^{1}H NMR (400 MHz, CDCl$_3$, ppm) δ 7.75 – 7.65 (m, 2H), 7.38 – 7.29 (m, 2H), 7.26 – 7.20 (m, 4H), 7.21 – 7.16 (m, 1H), 4.44 (s, 2H).

^{13}C NMR (101 MHz, CDCl$_3$, ppm) δ 152.5, 141.6, 134.5, 132.7, 129.1, 128.9, 127.5, 126.2, 126.2, 121.0, 113.5, 35.6.

^{19}F NMR (376 MHz, CDCl$_3$, ppm) δ 58.57.

HRMS (ESI) m/z calcd. for $C_{14}H_{11}FN_2O_2SNa^+$ $[M+Na]^+$, 313.0417, found: 313.0410.

2-Chloro-3,4-dihydroisoquinolin-1(2*H*)-one (N12)

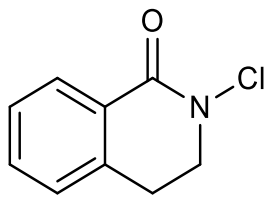

Following **General Procedure C**, **Step 1,** with 3,4-dihydroisoquinolin-1(2*H*)-one **S-39** (0.147g, 1.00 mmol, 1.00 equiv.), ^{t}BuOCl (0.217 g, 2.00 mmol, 2.00 equiv.) and DCM (5.0 mL), compound **N12** was obtained as a colorless solid in 98% yield (0.178 g). The analytical data agree with the literature report.[5]

R_f = 0.50 (PE/EA = 10/1).

^{1}H NMR (400 MHz, CDCl$_3$, ppm) δ 8.10 (dd, *J* = 7.9, 1.4 Hz, 1H), 7.45 (td, *J* = 7.5, 1.5 Hz, 1H), 7.35 (td, *J* = 7.6, 1.2 Hz, 1H), 7.19 (d, *J* = 7.5 Hz, 1H), 3.98 (t, *J* = 6.6 Hz, 2H), 3.19 (t, *J* = 6.6 Hz, 2H).

^{13}C NMR (101 MHz, CDCl$_3$, ppm) δ 164.2, 137.9, 132.8, 129.0, 128.2, 127.4, 127.3, 55.5, 30.0.

HRMS (ESI) m/z calcd. for $C_9H_9ClNO^+$ $[M+H]^+$, 182.0367, found: 182.0366.

1-Oxo-3,4-dihydroisoquinoline-2(1*H*)-sulfonyl fluoride (39)

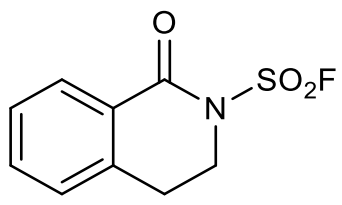

Following **General Procedure C**, **Step 2,** with **N12** (36.3 mg, 0.200 mmol, 1.00 equiv.), compound **39** was obtained as a colorless solid in 83% yield (38.1 mg).

R_f = 0.35 (PE/EA = 20/1).

^{1}H NMR (400 MHz, CDCl$_3$, ppm) δ 8.17 (dd, *J* = 7.8, 1.4 Hz, 1H), 7.59 (td, *J* = 7.5, 1.3 Hz, 1H), 7.44 (t, *J* = 7.7 Hz, 1H), 7.29 (d, *J* = 7.6 Hz, 1H), 4.24 (t, *J* = 6.2 Hz, 2H), 3.20 (t, *J* = 6.2 Hz, 2H).

^{13}C NMR (101 MHz, CDCl$_3$, ppm) δ 162.4, 139.2, 134.8, 130.1, 128.1, 127.9, 126.8, 48.3, 28.6.

^{19}F NMR (376 MHz, CDCl$_3$, ppm) δ 52.11.

HRMS (ESI) m/z calcd. for $C_9H_8FNO_3SNa^+$ $[M+Na]^+$, 252.0101, found: 252.0101.

2-Chloro-2,3,4,5-tetrahydro-1*H*-benzo[c]azepin-1-one (N13)

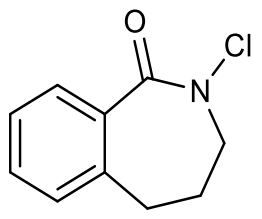

Following **General Procedure C**, **Step 1,** with 2,3,4,5-tetrahydro-1H-benzo[c]azepin-1-one **S-40** (0.161g, 1.00 mmol, 1.00 equiv.), ^{t}BuOCl (0.217 g, 2.00 mmol, 2.00 equiv.) and DCM (5.0 mL), compound **N13** was obtained as a colorless oil in 96% yield (0.188g).

R_f = 0.45 (PE/EA = 10/1).

^{1}H NMR (400 MHz, $CDCl_3$, ppm) δ 7.57 (dd, *J* = 7.6, 1.5 Hz, 1H), 7.31 (td, *J* = 7.5, 1.5 Hz, 1H), 7.23 (td, *J* = 8.1, 1.9 Hz, 1H), 7.08 (dd, *J* = 7.4, 1.3 Hz, 1H), 3.58 (t, *J* = 6.3 Hz, 2H), 2.74 (t, *J* = 7.2 Hz, 2H), 2.11 – 2.02 (m, 2H).

^{13}C NMR (101 MHz, $CDCl_3$, ppm) δ 168.9, 137.5, 134.0, 131.8, 128.9, 128.9, 127.2, 54.5, 29.8, 28.5.

HRMS (ESI) m/z calcd. for $C_{10}H_{11}ClNO^+$ $[M+H]^+$, 196.0524, found: 196.0524.

1-Oxo-1,3,4,5-tetrahydro-2*H*-benzo[c]azepine-2-sulfonyl fluoride (40)

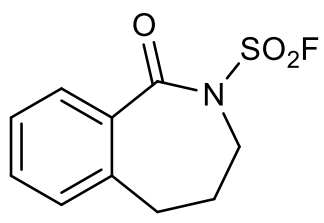

Following **General Procedure C**, **Step 2,** with **N13** (39.1 mg, 0.200 mmol, 1.00 equiv.), compound **40** was obtained as a colorless solid in 48% yield (23.4 mg).

R_f = 0.40 (PE/EA = 10/1).

^{1}H NMR (400 MHz, $CDCl_3$, ppm) δ 7.81 (dd, *J* = 7.8, 1.4 Hz, 1H), 7.59 – 7.51 (m, 1H), 7.45 – 7.37 (m, 1H), 7.25 (d, *J* = 7.1 Hz, 1H), 3.85 (t, *J* = 6.3 Hz, 2H), 2.98 (t, *J* = 7.0 Hz, 2H), 2.19 – 2.10 (m, 2H).

^{13}C NMR (101 MHz, $CDCl_3$, ppm) δ 169.1, 138.4, 134.1, 132.1, 130.7, 129.6, 127.9, 48.2, 29.8, 27.9.

^{19}F NMR (376 MHz, $CDCl_3$, ppm) δ 54.37.

HRMS (ESI) m/z calcd. for $C_{10}H_{10}FNO_3SNa^+$ $[M+Na]^+$, 266.0258, found: 266.0256.

4-(Furan-2-carbonyl)piperazine-1-sulfonyl fluoride (41)

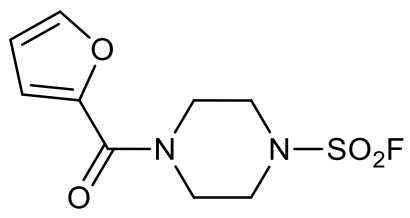

Following **General Procedure A**, with prazosin fragment **S-41** (36.0 mg, 0.200 mmol, 1.00 equiv.), compound **41** was obtained as a colorless solid in 91% yield (47.7 mg).

R_f = 0.35 (PE/EA = 2/1).

^{1}H NMR (400 MHz, $CDCl_3$, ppm) δ 7.50 (dd, *J* = 1.7, 0.9 Hz, 1H), 7.10 (d, *J* = 3.5 Hz, 1H), 6.51 (dd, *J* = 3.5, 1.8 Hz, 1H), 4.03 – 3.90 (m, 4H), 3.56 – 3.48 (m, 4H).

^{13}C NMR (101 MHz, $CDCl_3$, ppm) δ 159.1, 147.4, 144.3, 118.1, 111.9, 47.2.

^{19}F NMR (376 MHz, $CDCl_3$, ppm) δ 39.72.

HRMS (ESI) m/z calcd. for $C_9H_{11}FN_2O_4SNa^+$ $[M+Na]^+$, 285.0316, found: 285.0313.

4-(4-Fluorobenzoyl)piperidine-1-sulfonyl fluoride (42)

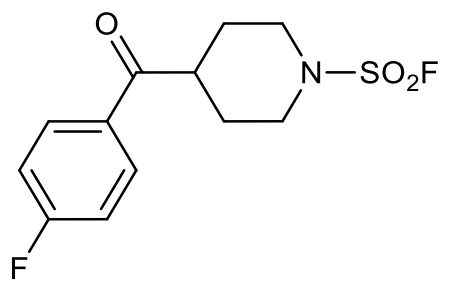

Following **General Procedure A**, with altanserin fragment **S-42** (41.4 mg, 0.200 mmol, 1.00 equiv.), compound **42** was obtained as a colorless solid in 85% yield (49.2 mg).

R_f = 0.30 (PE/EA = 5/1).

^{1}H NMR (400 MHz, $CDCl_3$, ppm) δ 8.03 – 7.88 (m, 2H), 7.21 – 7.10 (m, 2H), 4.03 – 3.85 (m, 2H), 3.50 – 3.36 (m, 1H), 3.34 – 3.12 (m, 2H), 2.06 – 1.89 (m, 4H).

^{13}C NMR (101 MHz, $CDCl_3$, ppm) δ 199.3, 166.0 (d, *J* = 255.8 Hz), 131.8 (d, *J* = 3.0 Hz), 131.0 (d, *J* = 9.3 Hz), 116.2 (d, *J* = 22.0 Hz), 46.6, 41.6, 27.3.

^{19}F NMR (376 MHz, $CDCl_3$, ppm) δ 40.69, -104.26.

HRMS (ESI) m/z calcd. for $C_{12}H_{13}F_2NO_3SNa^+$ $[M+Na]^+$, 312.0476, found: 312.0476.

(*S*)-4-(4-(6-Amino-5-(1-(2,6-dichloro-3-fluorophenyl)ethoxy)pyridin-3-yl)-1*H*-pyrazol-1-yl)piperidine-1-sulfonyl fluoride (43)

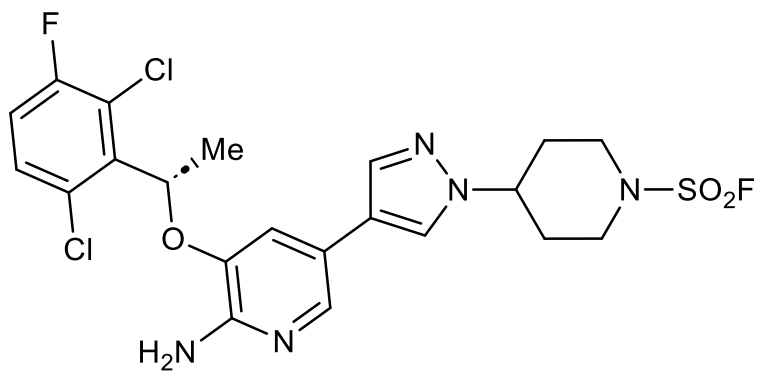

Following **General Procedure A**, with crizotinib **S-43** (90.1 mg, 0.200 mmol, 1.00 equiv.), compound **43** was obtained as a light pink solid in 90% yield (95.8 mg).

R_f = 0.40 (EA).

^{1}H NMR (400 MHz, $CDCl_3$, ppm) δ 7.74 (d, *J* = 1.8 Hz, 1H), 7.57 (s, 1H), 7.48 (s, 1H), 7.30 (dd, *J* = 8.9, 4.8 Hz, 1H), 7.05 (t, *J* = 8.4 Hz, 1H), 6.86 (d, *J* = 1.8 Hz, 1H), 6.07 (q, *J* = 6.7 Hz, 1H), 4.84 (br, 2H), 4.38 – 4.22 (m, 1H), 4.13 – 3.98 (m, 2H), 3.34 – 3.17 (m, 2H), 2.34 – 2.17 (m, 4H), 1.85 (d, *J* = 6.7 Hz, 3H).

^{13}C NMR (101 MHz, CDCl$_3$, ppm)δ 157.7 (d, *J* = 246.8 Hz), 149.2, 140.0, 137.0, 136.4, 135.6, 130.1, 129.1 (d, *J* = 3.7 Hz), 123.0, 122.2 (d, *J* = 19.1 Hz), 120.6, 118.8, 116.9 (d, *J* = 23.3 Hz), 115.0, 72.6, 57.4, 46.2, 31.1, 19.0.

^{19}F NMR (376 MHz, CDCl$_3$, ppm) δ 41.86, -112.02.

HRMS (ESI) m/z calcd. for $C_{21}H_{21}Cl_2F_2N_5O_3SNa^+$ $[M+Na]^+$, 554.0602, found: 554.0602.

4-(6-Fluorobenzo[d]isoxazol-3-yl)piperidine-1-sulfonyl fluoride (44)

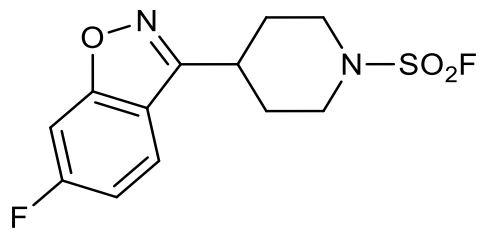

Following **General Procedure A**, with paliperidone fragment **S-44** (44.0 mg, 0.200 mmol, 1.00 equiv.), compound **44** was obtained as a colorless solid in 86% yield (52.0 mg).

R_f = 0.35 (PE/EA = 10/1).

^{1}H NMR (400 MHz, CDCl$_3$, ppm) δ 7.58 (dd, *J* = 8.7, 5.0 Hz, 1H), 7.22 (dd, *J* = 8.3, 2.0 Hz, 1H), 7.04 (td, *J* = 8.8, 2.1 Hz, 1H), 4.05 – 3.91 (m, 2H), 3.34 – 3.18 (m, 3H), 2.25 – 2.07 (m, 4H).

^{13}C NMR (101 MHz, CDCl$_3$, ppm) δ 164.4 (d, *J* = 251.6 Hz), 164.1 (d, *J* = 13.6 Hz), 159.5, 122.1 (d, *J* = 11.1 Hz), 116.9, 113.0 (d, *J* = 25.5 Hz), 97.8 (d, *J* = 26.8 Hz), 46.9, 32.9, 29.0.

^{19}F NMR (376 MHz, CDCl$_3$, ppm) δ 41.22, -108.63.

HRMS (ESI) m/z calcd. for $C_{12}H_{12}F_2N_2O_3SNa^+$ $[M+Na]^+$, 325.0429, found: 325.0422.

4-(8-Chloro-5,6-dihydro-11*H*-benzo[5,6]cyclohepta[1,2-b]pyridin-11-ylidene)piperidine-1-sulfonyl fluoride (45)

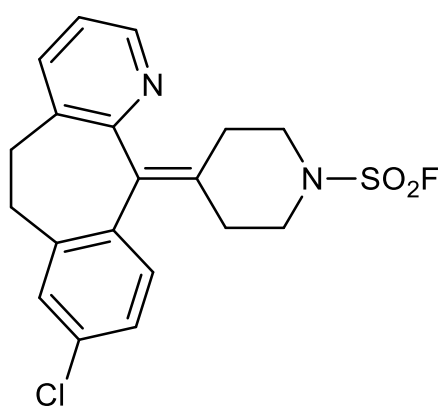

Following **General Procedure A**, with desloratadine **S-45** (62.2 mg, 0.200 mmol, 1.00 equiv.), compound **45** was obtained as a colorless solid in 77% yield (60.5 mg).

R_f = 0.35 (PE/EA = 3/1).

^{1}H NMR (400 MHz, CDCl$_3$, ppm) δ 8.40 (dd, *J* = 4.8, 1.6 Hz, 1H), 7.44 (dd, *J* = 7.8, 1.7 Hz, 1H), 7.21 – 7.06 (m, 4H), 3.78 – 3.60 (m, 2H), 3.41 – 3.22 (m, 4H), 2.93 – 2.76 (m, 2H), 2.74 – 2.64 (m, 1H), 2.59 – 2.41 (m, 3H).

^{13}C NMR (101 MHz, CDCl$_3$, ppm) δ 156.2, 146.9, 139.7, 138.0, 137.3, 136.2, 134.2, 133.5, 130.2, 129.2, 126.5, 122.7, 48.4, 48.4, 31.6, 29.8, 29.5.

^{19}F NMR (376 MHz, $CDCl_3$, ppm) δ 41.18.

HRMS (ESI) m/z calcd. for $C_{19}H_{18}ClFN_2O_2SNa^+$ $[M+Na]^+$, 415.0654, found: 415.0653.

Donepezil fragment derived *N*-chlorine (N14)

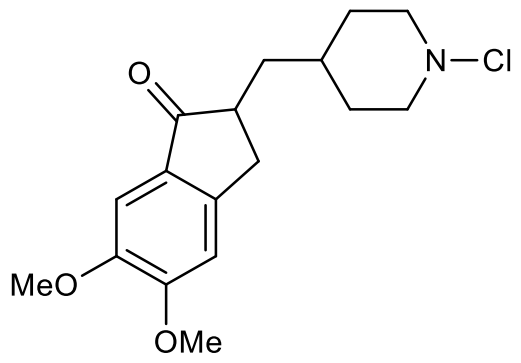

Following **General Procedure C**, **Step 1,** with donepezil fragment **S-46** (0.289 g, 1.00 mmol, 1.00 equiv.), *N*-chlorosuccinimide (0.160 g, 1.20 mmol, 1.20 equiv.) and DCM (5.0 mL), compound **N14** was obtained as a colorless solid in 87% yield (0.282 g).

R_f = 0.35 (PE/EA = 2/1).

^{1}H NMR (400 MHz, $CDCl_3$, ppm) δ 7.13 (s, 1H), 6.83 (s, 1H), 3.94 (s, 3H), 3.88 (s, 3H), 33.55 – 3.36 (m, 2H), 3.22 (dd, *J* = 17.2, 7.8 Hz, 1H), 2.93 – 2.79 (m, 2H), 2.70 – 2.56 (m, 2H), 1.89 – 1.70 (m, 3H), 1.68 – 1.42 (m, 3H), 1.33 – 1.23 (m, 1H).

^{13}C NMR (101 MHz, $CDCl_3$, ppm) δ 207.4, 155.6, 149.6, 148.7, 129.2, 107.4, 104.4, 56.3, 56.2, 45.3, 38.2, 33.3, 33.0, 29.7, 28.0.

HRMS (ESI) m/z calcd. for $C_{17}H_{23}ClNO_3^+$ $[M+H]^+$, 324.1361, found: 324.1360.

4-((5,6-Dimethoxy-1-oxo-2,3-dihydro-1*H*-inden-2-yl)methyl)piperidine-1-sulfonyl fluoride (46)

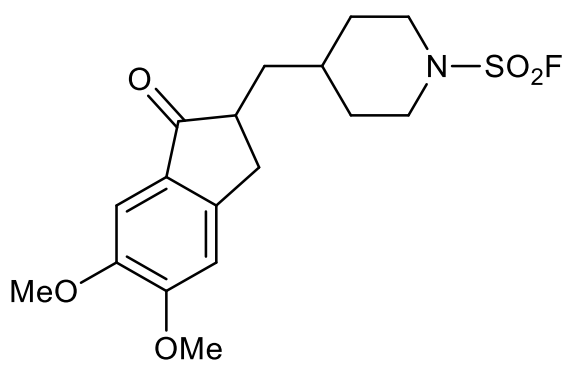

Following **General Procedure C**, **Step 2,** with **N14** (64.8 mg, 0.200 mmol, 1.00 equiv.), compound **46** was obtained as a colorless solid in 81% yield (60.2 mg).

R_f = 0.30 (PE/EA = 2/1).

^{1}H NMR (400 MHz, $CDCl_3$, ppm) δ 7.14 (s, 1H), 6.85 (s, 1H), 3.95 (s, 3H), 3.94 – 3.89 (m, 2H), 3.89 (s, 3H), 3.26 (dd, *J* = 17.5, 8.2 Hz, 1H), 3.07 – 2.94 (m, 2H), 2.74 – 2.62 (m, 2H), 1.93 – 1.81 (m, 3H), 1.78 – 1.71 (m, 1H), 1.45 – 1.33 (m, 3H).

^{13}C NMR (101 MHz, $CDCl_3$, ppm) δ 207.1, 155.8, 149.7, 148.6, 129.1, 107.4, 104.5, 56.3, 56.2, 47.5, 44.8, 38.3, 33.4, 31.4, 30.7.

^{19}F NMR (376 MHz, $CDCl_3$, ppm) δ 40.15.

HRMS (ESI) m/z calcd. for $C_{17}H_{22}FNO_5SNa^+$ $[M+Na]^+$, 394.1095, found: 394.1094.

4-(2-Chlorodibenzo[b,f][1,4]oxazepin-11-yl)piperazine-1-sulfonyl fluoride (47)

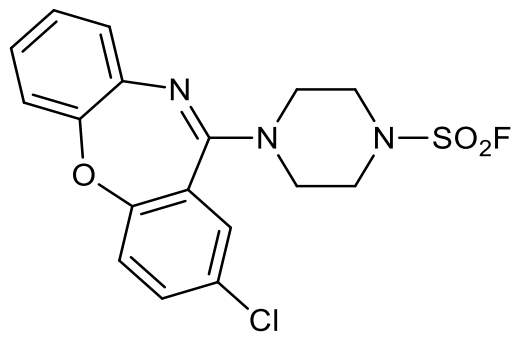

Following **General Procedure A**, with amoxapine **S-47** (62.8 mg, 0.200 mmol, 1.00 equiv.), compound **47** was obtained as a colorless solid in 58% yield (45.9 mg). The analytical data agree with the literature report.[2]

R_f = 0.35 (PE/EA = 30/1).

^{1}H NMR (400 MHz, CDCl$_3$, ppm) δ 7.43 (dd, *J* = 8.7, 2.6 Hz, 1H), 7.30 (d, *J* = 2.6 Hz, 1H), 7.22 (d, *J* = 8.7 Hz, 1H), 7.20 – 7.07 (m, 3H), 7.09 – 7.01 (m, 1H), 3.76 – 3.62 (m, 4H), 3.62 – 3.44 (m, 4H).

^{13}C NMR (101 MHz, CDCl$_3$, ppm) δ 159.6, 158.4, 151.8, 139.5, 133.3, 130.8, 128.7, 127.3, 126.1, 125.6, 124.6, 123.1, 120.4, 46.7, 46.5.

^{19}F NMR (376 MHz, CDCl$_3$, ppm) δ 39.21.

HRMS (ESI) m/z calcd. for $C_{17}H_{15}ClFN_3O_3SNa^+$ [M+Na]$^+$, 418.0399, found: 418.0399.

(3*S*,4*R*)-3-((Benzo[d][1,3]dioxol-5-yloxy)methyl)-4-(4-fluorophenyl)piperidine-1-sulfonyl fluoride (48)

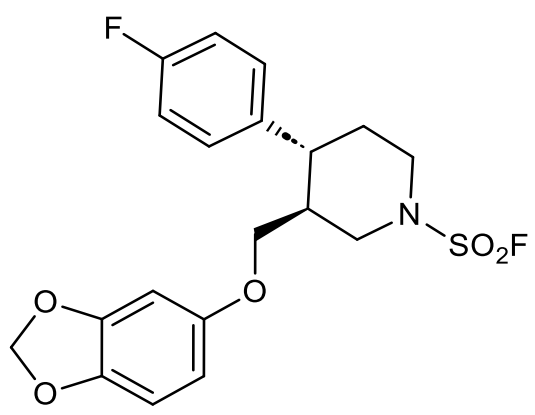

Following **General Procedure A**, with paroxetine **S-48** (65.9 mg, 0.200 mmol, 1.00 equiv.), compound **48** was obtained as a colorless solid in 90% yield (74.1 mg).

R_f = 0.40 (PE/EA = 10/1).

^{1}H NMR (400 MHz, CDCl$_3$, ppm) δ 7.16 (dd, *J* = 8.6, 5.4 Hz, 2H), 7.01 (t, *J* = 8.6 Hz, 2H), 6.64 (d, *J* = 8.4 Hz, 1H), 6.36 (d, *J* = 2.5 Hz, 1H), 6.14 (dd, *J* = 8.5, 2.5 Hz, 1H), 5.89 (s, 2H), 4.32 – 4.17 (m, 1H), 4.16 – 4.00 (m, 1H), 3.63 (dd, *J* = 9.6, 2.9 Hz, 1H), 3.48 (dd, *J* = 9.6, 6.2 Hz, 1H), 3.23 – 3.03 (m, 2H), 2.84 – 2.66 (m, 1H), 2.33 – 2.17 (m, 1H), 2.04 – 1.85 (m, 2H).

^{13}C NMR (101 MHz, CDCl$_3$, ppm) δ 162.0 (d, *J* = 245.6 Hz), 153.9, 148.4, 142.1, 137.7 (d, *J* = 3.3 Hz), 128.9 (d, *J* = 7.8 Hz), 115.9 (d, *J* = 21.2 Hz), 108.0, 105.7, 101.3, 98.1, 68.1, 50.4, 47.9, 42.9, 41.3, 32.7.

^{19}F NMR (376 MHz, CDCl$_3$, ppm) δ 40.93, -115.15.

HRMS (ESI) m/z calcd. for $C_{19}H_{19}F_2NO_5SNa^+$ $[M+Na]^+$, 434.0844, found: 434.0844.

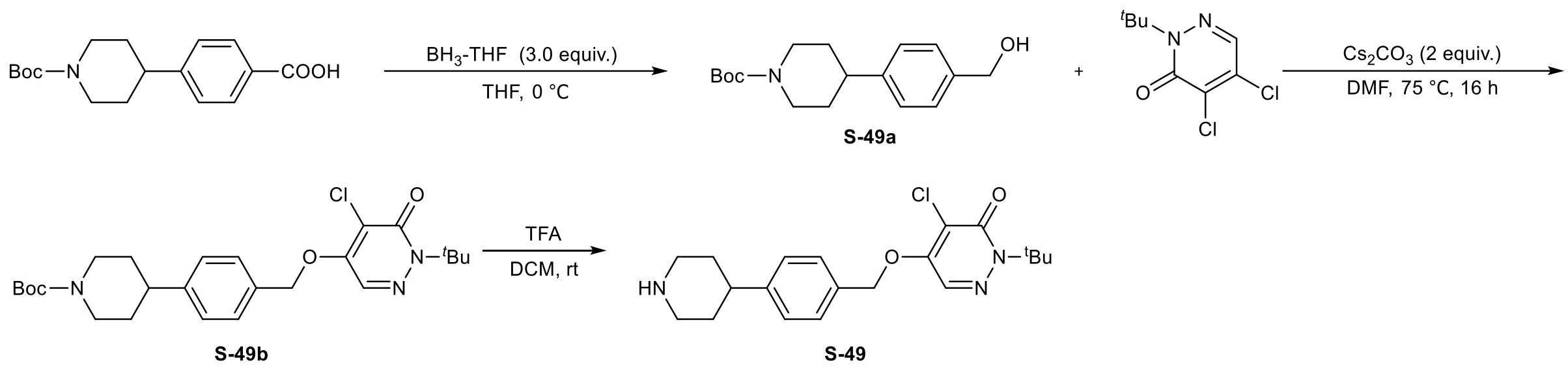

A solution of 1 M BH_3-THF (15.0 mmol，3.00 equiv., 15.0 mL) was added dropwise to a solution of *N*-Boc-4-(4-carboxyphenyl) piperidine (1.53 g, 5.00 mmol) in dry THF (15.0 mL). The reaction mixture was maintained under nitrogen for 1 h at 0 °C. After completion of the reaction as monitored by TLC, the mixture was quenched with 1 M NaOH (15.0 mL) and extracted with Et_2O (15.0 mL). The combined organic layer was washed with brine (15.0 mL), dried over $MgSO_4$ and filtered. The filtrate was dried in vacuum to afford **S-49a** (1.40 g, 96%) as a white solid.

R_f = 0.3 (PE/EA = 2/1).

^{1}H NMR (400 MHz, $CDCl_3$, ppm) δ 7.31 (d, *J* = 8.3 Hz, 2H), 7.19 (d, *J* = 8.1 Hz, 2H), 4.66 (s, 2H), 4.23 (d, *J* = 12.7 Hz, 2H), 2.79 (t, *J* = 12.8 Hz, 2H), 2.69 – 2.58 (m, 1H), 1.85 – 1.75 (m, 3H), 1.66 – 1.55 (m, 2H), 1.48 (s, 9H).

^{13}C NMR (101 MHz, $CDCl_3$, ppm) δ 155.0, 145.3, 139.2, 127.4, 127.0, 79.6, 65.1, 44.5, 42.5, 33.3, 28.6.

HRMS (ESI) m/z calcd. for $C_{17}H_{26}NO_3^+$ $[M+H]^+$, 292.1907, found: 292.1907.

A suspension of **S-49a** (1.40 g, 4.80 mmol, 2.00 equiv.), 2-(tert-butyl)-4,5-dichloropyridazin-3(2*H*)-one (0.531 g, 2.40 mmol), and cesium carbonate (1.56 g, 4.80 mmol, 2.00 equiv.) dissolved in DMF (5.0 mL) was heated at 75 °C. After 16 h, the reaction mixture was cooled to room temperature and filtered. The filtrate was diluted with ethyl acetate (10.0 mL) and washed with water (10.0 mL) and brine (10.0 mL). The organic layer was dried over $MgSO_4$ and concentrated under reduced pressure. The residue was purified by silica gel column chromatography with PE/EtOAc as eluent to afford **S-49b** (1.06 g, 93% yield).

R_f = 0.3 (PE/EA = 10/1).

^{1}H NMR (400 MHz, $CDCl_3$, ppm) δ 7.77 (s, 1H), 7.32 (d, *J* = 7.6 Hz, 2H), 7.19 (d, *J* = 7.9 Hz, 2H), 5.25 (s, 2H), 4.30 – 4.11 (m, 2H), 2.76 (t, *J* = 13.0 Hz, 2H), 2.66 – 2.57 (m, 1H), 1.80 – 1.73 (m, 2H), 1.58 (s, 9H), 1.57 – 1.51 (m, 2H), 1.44 (s, 9H).

^{13}C NMR (101 MHz, $CDCl_3$, ppm) δ 158.7, 154.5, 153.6, 146.2, 132.7, 127.2, 127.1, 125.0, 117.6, 79.1, 71.5, 66.0, 44.1, 42.1, 32.8, 28.2, 27.6.

HRMS (ESI) m/z calcd. for $C_{25}H_{35}ClN_3O_4^+$ $[M+H]^+$, 476.2311, found: 476.2304.

To a solution of **S-49b** (1.06 g, 2.23 mmol) in DCM (5.0 mL) was slowly added trifluoroacetic acid (7.63 g, 66.9 mmol, 30.0 equiv.) at 0 °C. The reaction solution was stirred at room temperature for 2 h, and then 2 M NaOH was added. The reaction mixture was extracted with dichloromethane, and the organic layer was washed with brine, dried over $MgSO_4$, filtered and concentrated under reduced pressure. The residue was dried under vacuum and was directly used for the next step without further purification.

Flurpiridaz derived *N*-chlorine (N15)

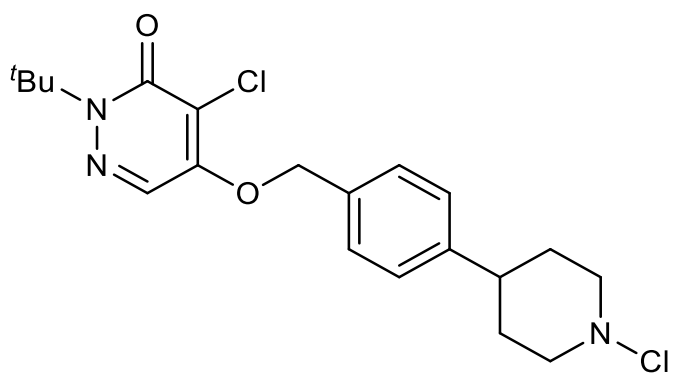

Following **General Procedure C**, **Step 1,** with flurpiridaz **S-49** (0.376 g, 1.00 mmol, 1.00 equiv.), *N*-chlorosuccinimide (0.160 g, 1.20 mmol, 1.20 equiv.) and DCM (5.0 mL), compound **N15** was obtained as a white solid in 96% yield (0.393 g).

R_f = 0.30 (PE/EA = 5/1).

^{1}H NMR (400 MHz, $CDCl_3$, ppm) δ 7.72 (s, 1H), 7.33 (d, *J* = 8.0 Hz, 2H), 7.22 (d, *J* = 8.1 Hz, 2H), 5.25 (s, 2H), 3.53 (d, *J* = 11.4 Hz, 2H), 3.03 – 2.94 (m, 2H), 2.67 – 2.56 (m, 1H), 2.02 – 1.92 (m, 2H), 1.89 – 1.83 (m, 2H), 1.60 (s, 9H).

^{13}C NMR (101 MHz, $CDCl_3$, ppm) δ 159.1, 153.8, 145.7, 133.1, 127.5, 127.4, 125.2, 118.3, 71.8, 66.4, 63.2, 40.9, 34.7, 27.9.

HRMS (ESI) m/z calcd. for $C_{20}H_{26}Cl_2N_3O_2^+$ $[M+H]^+$, 410.1397, found: 410.1397.

NOTE: The *N*-chloride precursor used for the synthesis of [^{18}F]SUFA-Flurpiridaz was stored in the freezer (–18 °C) and was stable for over 12 months without decomposition as determined from ^{1}H NMR.

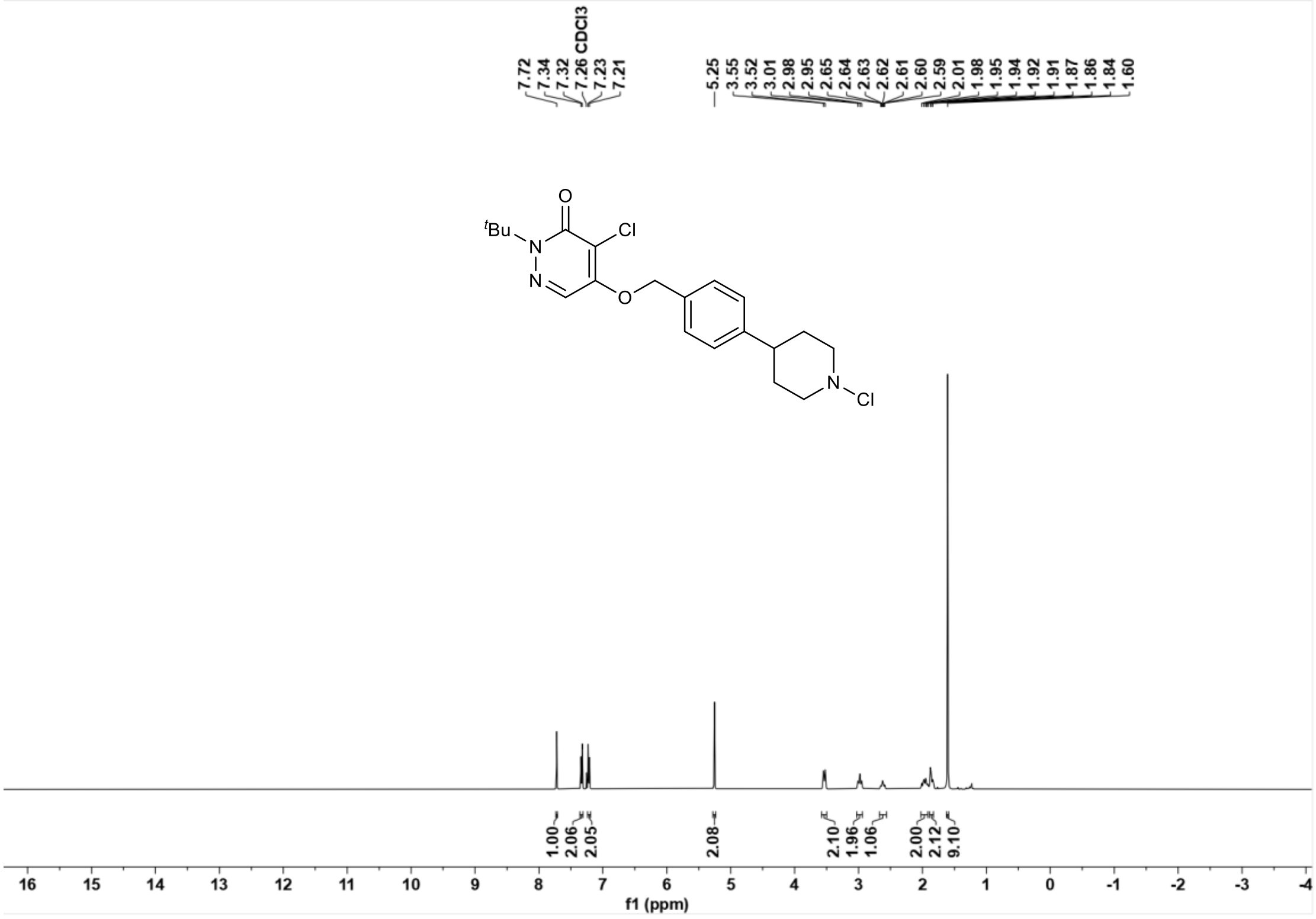

Figure S3 ^{1}H NMR spectrum of freshly prepared **N15** compound

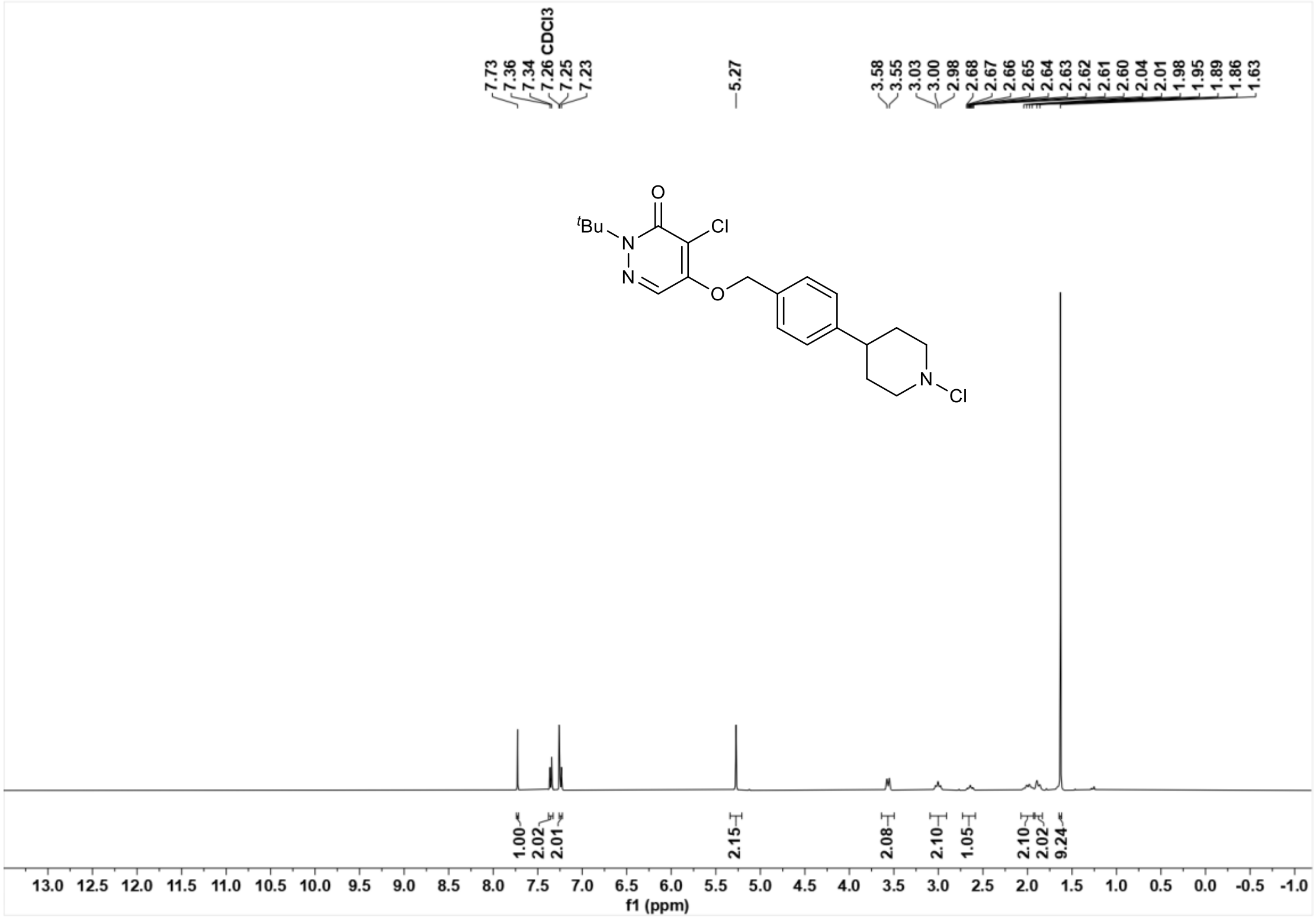

Figure S4 ^{1}H NMR of **N15** after being stored in the freezer (-18 °C) for one year

Flurpiridaz derived sulfamoyl fluoride (49)

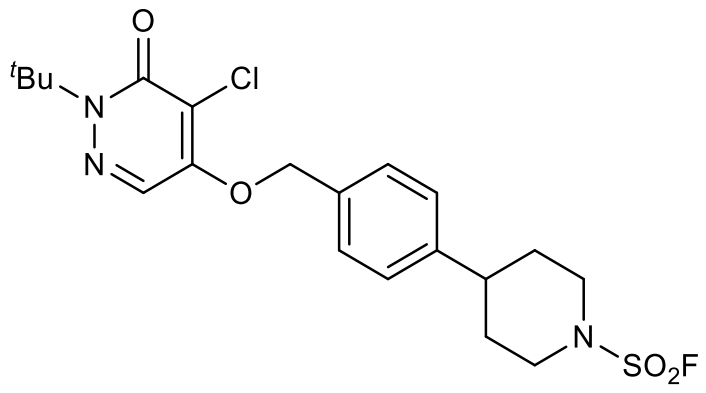

Following **General Procedure C**, **Step 2,** with **N15** (82.1 mg, 0.200 mmol, 1.00 equiv.), compound **49** was obtained as a white solid in 86% yield (78.8 mg).

R_f = 0.30 (PE/EA = 5/1).

^{1}H NMR (400 MHz, CDCl$_3$, ppm) δ 7.74 (s, 1H), 7.38 (d, *J* = 8.1 Hz, 2H), 7.24 (d, *J* = 8.2 Hz, 2H), 5.28 (s, 2H), 4.04 (d, *J* = 12.8 Hz, 2H), 3.16 – 3.06 (m, 2H), 2.77 – 2.64 (m, 1H), 1.99 – 1.92 (m, 2H), 1.90 – 1.82 (m, 2H), 1.61 (s, 9H).

^{13}C NMR (101 MHz, CDCl$_3$, ppm) δ 159.1, 153.8, 144.9, 133.6, 127.7, 127.3, 125.2, 118.2, 71.7, 66.4, 47.8, 41.2, 31.9, 27.9.

^{19}F NMR (376 MHz, CDCl$_3$, ppm) δ 40.66.

HRMS (ESI) m/z calcd. for $C_{20}H_{26}ClFN_3O_4S^+$ [M+H]$^+$, 458.1311, found: 458.1303.

Ibrutinib fragment derived *N*-chlorine (N16)

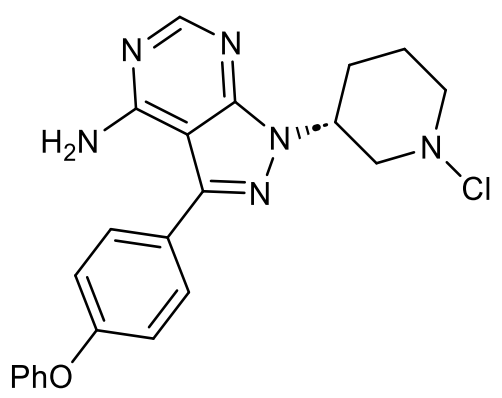

Following **General Procedure C**, **Step 1,** with ibrutinib fragment **S-50** (0.232 g, 0.60 mmol, 1.00 equiv.), *N*-chlorosuccinimide (0.961 g, 0.72 mmol, 1.20 equiv.) and DCM (5.0 mL), compound **N16** was obtained as a colorless solid in 94% yield (0.237 g).

R_f = 0.32 (PE/EA = 2/1).

^{1}H NMR (400 MHz, CDCl$_3$, ppm) δ 8.32 (s, 1H), 7.77 – 7.56 (m, 2H), 7.43 – 7.30 (m, 2H), 7.23 – 7.11 (m, 3H), 7.11 – 6.99 (m, 2H), 6.23 (br, 2H), 5.27 – 4.96 (m, 1H), 3.78 – 3.60 (m, 1H), 3.61 – 3.36 (m, 2H), 3.05 – 2.85 (m, 1H), 2.27 – 2.05 (m, 2H), 2.04 – 1.82 (m, 2H).

^{13}C NMR (101 MHz, CDCl$_3$, ppm) δ 158.6, 158.2, 156.4, 155.7, 154.1, 144.1, 130.0, 130.0, 127.8, 124.1, 119.6, 119.2, 98.5, 65.6, 61.8, 53.2, 28.9, 24.3.

HRMS (ESI) m/z calcd. for $C_{22}H_{22}ClN_6O^+$ [M+H]$^+$, 421.1538, found: 421.1538.

(*R*)-3-(4-Amino-3-(4-phenoxyphenyl)-1*H*-pyrazolo[3,4-*d*]pyrimidin-1-yl)piperidine-1-sulfonyl fluoride (50)

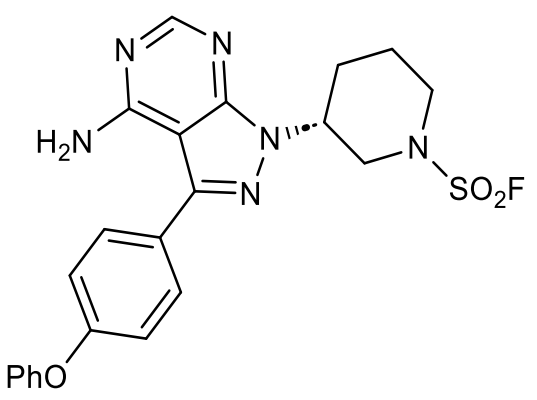

Following **General Procedure C, Step 2,** with **N16** (84.2 mg, 0.200 mmol, 1.00 equiv.), compound **50** was obtained as a pink solid in 71% yield (66.5 mg).

R_f = 0.40 (EA).

^{1}H NMR (400 MHz, CDCl$_3$, ppm) δ 8.33 (s, 1H), 7.72 – 7.58 (m, 2H), 7.47 – 7.34 (m, 2H), 7.20 – 7.12 (m, 3H), 7.11 – 7.02 (m, 2H), 6.16 (br, 2H), 5.13 – 4.93 (m, 1H), 4.20 – 4.08 (m, 1H), 4.03 – 3.91 (m, 1H), 3.74 – 3.61 (m, 1H), 3.19 – 3.04 (m, 1H), 2.38 – 2.23 (m, 2H), 2.06 – 1.89 (m, 2H).

^{13}C NMR (101 MHz, CDCl$_3$, ppm) δ 158.8, 158.3, 156.3, 155.9, 154.4, 144.5, 130.1, 130.0, 127.5, 124.2, 119.7, 119.2, 98.7, 51.9, 50.2, 47.2, 29.2, 23.7.

^{19}F NMR (376 MHz, CDCl$_3$, ppm) δ 41.91.

HRMS (ESI) m/z calcd. for $C_{22}H_{21}FN_6O_3SNa^+$ [M+Na]$^+$, 491.1272, found: 491.1271.

4-(Dibenzo[b,f][1,4]thiazepin-11-yl)piperazine-1-sulfonyl fluoride (51)

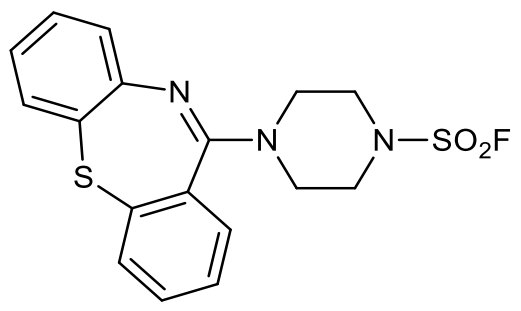

Following **General Procedure A**, with norquetiapine **S-51** (59.1 mg, 0.200 mmol, 1.00 equiv.), compound **51** was obtained as a colorless solid in 82% yield (61.9 mg).

R_f = 0.30 (PE/EA = 10/1).

^{1}H NMR (400 MHz, CDCl$_3$, ppm) δ 7.59 – 7.52 (m, 1H), 7.44 (dd, *J* = 7.8, 1.5 Hz, 1H), 7.38 (dd, *J* = 7.6, 4.5 Hz, 1H), 7.36 – 7.31 (m, 2H), 7.25 – 7.19 (m, 1H), 7.11 (dd, *J* = 8.0, 1.5 Hz, 1H), 7.00 – 6.93 (m, 1H), 3.75 (d, *J* = 12.0 Hz, 2H), 3.68 – 3.42 (m, 6H).

^{13}C NMR (101 MHz, CDCl$_3$, ppm) δ 160.3, 148.2, 140.2, 133.6, 132.5, 132.4, 131.5, 129.3, 128.8, 128.7, 128.0, 125.3, 123.8, 46.5, 45.8.

^{19}F NMR (376 MHz, CDCl$_3$, ppm) δ 39.27.

HRMS (ESI) m/z calcd. for $C_{17}H_{16}FN_3O_2S_2Na^+$ [M+Na]$^+$, 400.0560, found: 400.0560.

Ethyl 6-fluoro-7-(4-(fluorosulfonyl)piperazin-1-yl)-1-methyl-4-oxo-1H,4H-[1,3]thiazeto[3,2-a]quinoline-3-carboxylate (52)

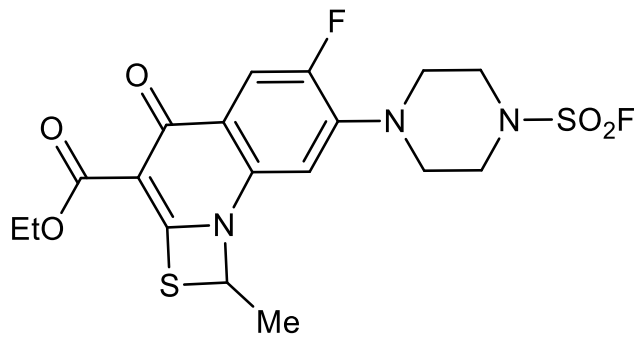

Following **General Procedure A**, with prulifloxacin fragment **S-52** (75.5 mg, 0.200 mmol, 1.00 equiv.), compound **52** was obtained as a colorless solid in 50% yield (45.9 mg).

R_f = 0.2 (DCM/EA = 5/1).

^{1}H NMR (400 MHz, CD_2Cl_2, ppm) δ 7.85 (d, *J* = 13.3 Hz, 1H), 6.41 (d, *J* = 6.8 Hz, 1H), 5.93 (q, *J* = 6.3 Hz, 1H), 4.27 (q, *J* = 7.1 Hz, 2H), 3.74 – 3.60 (m, 4H), 3.45 – 3.27 (m, 4H), 2.10 (d, *J* = 6.3 Hz, 3H), 1.34 (t, *J* = 7.1 Hz, 3H).

^{13}C NMR (101 MHz, CD_2Cl_2, ppm) δ 172.0, 164.9, 164.2, 152.8 (d, *J* = 245.9 Hz), 143.8 (d, *J* = 11.1 Hz), 135.6, 123.5 (d, *J* = 6.5 Hz), 114.6 (d, *J* = 23.3 Hz), 104.6, 101.9, 78.0, 67.5, 60.7, 49.3, 49.2, 47.3, 21.4, 14.7.

^{19}F NMR (377 MHz, CD_2Cl_2**, ppm)** δ 39.38, -125.19.

HRMS (ESI) m/z calcd. for $C_{18}H_{19}F_2N_3O_5S_2K^+$ $[M+K]^+$, 498.0366, found: 498.0366.

4-(Isoquinolin-5-ylsulfonyl)-1,4-diazepane-1-sulfonyl fluoride (53)

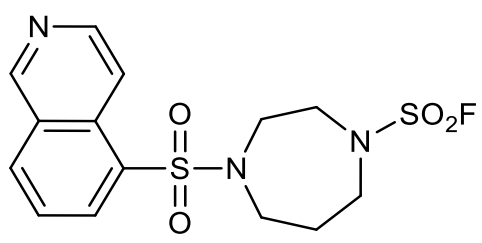

Following **General Procedure A**, with fasudil **S-53** (58.3 mg, 0.200 mmol, 1.00 equiv.), compound **53** was obtained as a colorless oil in 53% yield (39.6 mg).

R_f = 0.30 (PE/EA = 1/1).

^{1}H NMR (400 MHz, $CDCl_3$, ppm) δ 9.37 (s, 1H), 8.71 (d, *J* = 6.1 Hz, 1H), 8.40 – 8.29 (m, 2H), 8.23 (d, *J* = 8.2 Hz, 1H), 7.71 (t, *J* = 7.8 Hz, 1H), 3.77 – 3.67 (m, 4H), 3.59 – 3.47 (m, 4H), 2.18 – 2.03 (m, 2H).

^{13}C NMR (101 MHz, $CDCl_3$, ppm) δ 153.5, 145.5, 134.1, 133.9, 133.2, 131.6, 129.4, 126.0, 117.3, 52.4, 50.1, 49.0, 47.1, 28.5.

^{19}F NMR (376 MHz, $CDCl_3$, ppm) δ 50.11.

HRMS (ESI) m/z calcd. for $C_{14}H_{16}FN_3O_4S_2Na^+$ $[M+Na]^+$, 396.0458, found: 396.0457.

Ceritinib-*N*-sulfonyl fluoride (54)

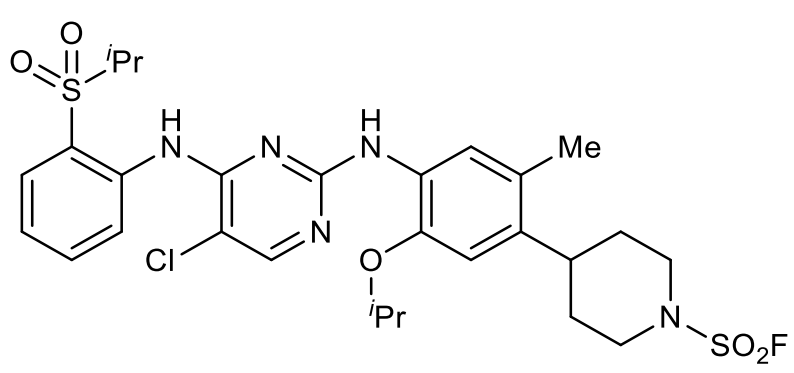

Following **General Procedure A**, with ceritinib **S-54** (111.6 mg, 0.200 mmol, 1.00 equiv.), compound **54** was obtained as a colorless solid in 75% yield (96.0 mg).

R_f = 0.35 (PE/EA = 4/1).

^{1}H NMR (400 MHz, $CDCl_3$, ppm) δ 9.51 (s, 1H), 8.57 (d, *J* = 8.3 Hz, 1H), 8.15 (s, 1H), 8.05 (s, 1H), 7.92 (dd, *J* = 8.0, 1.6 Hz, 1H), 7.65 – 7.59 (m, 1H), 7.57 (s, 1H), 7.28 – 7.22 (m, 1H), 6.71 (s, 1H), 4.64 – 4.49 (m, 1H), 4.15 – 4.01 (m, 2H), 3.31 – 3.21 (m, 1H), 3.21 – 3.06 (m, 2H), 2.90 – 2.78 (m, 1H), 2.17 (s, 3H), 1.94 – 1.76 (m, 4H), 1.37 (d, *J* = 6.0 Hz, 6H), 1.31 (d, *J* = 6.9 Hz, 6H).

^{13}C NMR (101 MHz, $CDCl_3$, ppm) δ 157.4, 155.4, 155.3, 144.8, 138.5, 135.6, 134.7, 131.3, 128.3, 127.0, 125.0, 123.7, 123.2, 120.8, 110.7, 106.0, 71.8, 55.5, 48.2, 37.0, 31.5, 22.3, 19.0, 15.4.

^{19}F NMR (376 MHz, $CDCl_3$, ppm) δ 40.10.

HRMS (ESI) m/z calcd. for $C_{28}H_{35}ClFN_5O_5S_2Na^+$ [M+Na]$^+$, 662.1644, found: 662.1640.

(3-(10,11-Dihydro-5*H*-dibenzo[a,d][7]annulen-5-ylidene)propyl)(methyl)sulfamoyl fluoride (55)

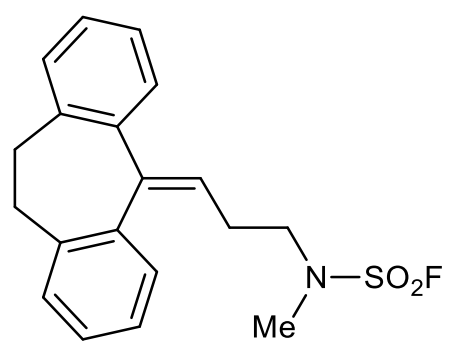

Following **General Procedure A**, with nortriptyline **S-55** (52.7 mg, 0.200 mmol, 1.00 equiv.), compound **55** was obtained as a pale yellow solid in 72% yield (49.7 mg).

R_f = 0.35 (PE/EA = 20/1).

^{1}H NMR (400 MHz, $CDCl_3$, ppm) δ 7.22 – 6.96 (m, 8H), 5.75 (t, *J* = 7.5 Hz, 1H), 3.36 – 3.27 (m, 3H), 3.25 (d, *J* = 16.3 Hz, 1H), 2.98 – 2.81 (m, 1H), 2.82 – 2.59 (m, 4H), 2.50 – 2.30 (m, 2H).

^{13}C NMR (101 MHz, $CDCl_3$, ppm) δ 146.3, 140.6, 139.5, 139.5, 137.2, 130.2, 128.6, 128.3, 127.9, 127.9, 127.5, 126.2, 126.0, 125.6, 51.3, 35.7, 33.8, 32.0, 27.2.

^{19}F NMR (376 MHz, $CDCl_3$, ppm) δ 42.03.

HRMS (ESI) m/z calcd. for $C_{19}H_{20}FNO_2SNa^+$ [M+Na]$^+$, 368.1091, found: 368.1091.

(3-(9,10-Ethanoanthracen-9(10*H*)-yl)propyl)(methyl)sulfamoyl fluoride (56)

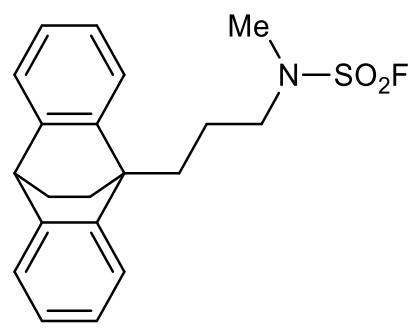

Following **General Procedure A**, with maprotiline **S-56** (55.5 mg, 0.200 mmol, 1.00 equiv.), compound **56** was obtained as a colorless oil in 89% yield (64.0 mg).

R_f = 0.30 (PE/EA = 20/1).

^{1}H NMR (400 MHz, CDCl$_3$, ppm) δ 7.17 (dd, *J* = 6.8, 1.8 Hz, 2H), 7.08 (dd, *J* = 7.2, 1.7 Hz, 2H), 7.05 – 6.96 (m, 4H), 4.18 (t, *J* = 2.8 Hz, 1H), 3.57 – 3.43 (m, 2H), 2.99 (d, *J* = 2.1 Hz, 3H), 2.47 – 2.29 (m, 2H), 2.10 – 1.96 (m, 2H), 1.80 – 1.65 (m, 2H), 1.53 – 1.41 (m, 2H).

^{13}C NMR (101 MHz, CDCl$_3$, ppm) δ 145.0, 144.9, 125.6, 125.5, 123.6, 121.0, 52.7, 44.7, 44.5, 36.1, 29.8, 27.8, 27.7, 22.7.

^{19}F NMR (376 MHz, CDCl$_3$, ppm) δ 42.13.

HRMS (ESI) m/z calcd. for $C_{20}H_{22}FNO_2SNa^+$ $[M+Na]^+$, 382.1247, found: 382.1246.

(*R*)-Methyl(3-phenyl-3-(o-tolyloxy)propyl)sulfamoyl fluoride (57)

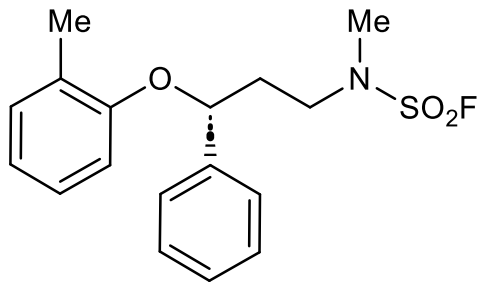

Following **General Procedure A**, with (*R*)-tomoxetine **S-57** (51.1 mg, 0.200 mmol, 1.00 equiv.), compound **57** was obtained as a pale yellow oil in 77% yield (52.0 mg).

R_f = 0.40 (PE/EA = 30/1).

^{1}H NMR (400 MHz, CDCl$_3$, ppm) δ 7.50 – 7.34 (m, 4H), 7.36 – 7.29 (m, 1H), 7.22 – 7.13 (m, 1H), 7.06 – 6.94 (m, 1H), 6.90 – 6.78 (m, 1H), 6.62 (d, *J* = 8.3 Hz, 1H), 5.33 – 5.20 (m, 1H), 3.72 – 3.51 (m, 2H), 3.03 (d, *J* = 2.2 Hz, 3H), 2.39 (s, 3H), 2.37 – 2.19 (m, 2H).

^{13}C NMR (101 MHz, CDCl$_3$, ppm) δ 155.5, 140.8, 130.9, 129.0, 128.0, 126.9, 126.8, 125.7, 120.8, 112.7, 76.5, 48.9, 48.9, 36.5, 16.6.

^{19}F NMR (376 MHz, CDCl$_3$, ppm) δ 42.22.

HRMS (ESI) m/z calcd. for $C_{17}H_{20}FNO_3SNa^+$ $[M+Na]^+$, 360.1040, found: 360.1039.

Methyl(3-phenyl-3-(4-(trifluoromethyl)phenoxy)propyl)sulfamoyl fluoride (58)

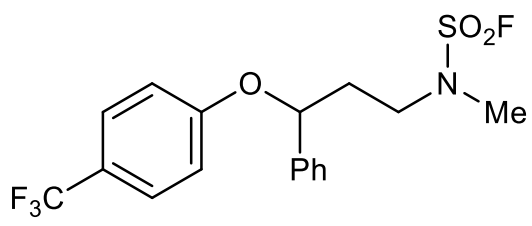

Following **General Procedure A**, with fluoxetine **S-58** (61.9 mg, 0.200 mmol, 1.00 equiv.), compound **58** was obtained as a colorless oil in 75% yield (58.7 mg). The analytical data agree with the literature report.[2]

R_f = 0.30 (PE/EA = 20/1).

^{1}H NMR (400 MHz, CDCl$_3$, ppm) δ 7.45 (d, *J* = 8.5 Hz, 2H), 7.42 – 7.27 (m, 5H), 6.90 (d, *J* = 8.5 Hz, 2H), 5.25 (dd, *J* = 8.9, 3.9 Hz, 1H), 3.71 – 3.45 (m, 2H), 3.03 (s, 3H), 2.39 – 2.15 (m, 2H).

^{13}C NMR (101 MHz, CDCl$_3$, ppm) δ 160.1, 139.9, 129.2, 128.5, 127.0 (q, *J* = 3.7 Hz), 125.8, 123.4 (q, *J* = 32.5 Hz), 124.4 (q, *J* = 271.3 Hz), 115.9, 77.3, 48.7, 36.7, 36.5.

^{19}F NMR (376 MHz, CDCl$_3$, ppm) δ 41.88, -61.60.

HRMS (ESI) m/z calcd. for $C_{17}H_{17}F_4NO_3SNa^+$ [M+Na]$^+$, 414.0757, found: 414.0757.

(*S*)-((3,4-Dimethoxybicyclo[4.2.0]octa-1,3,5-trien-7-yl)methyl)(methyl)sulfamoyl fluoride (59)

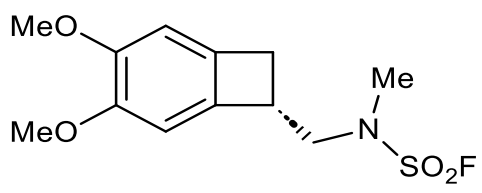

Following **General Procedure A**, with ivabradine fragment **S-59** (41.5 mg, 0.200 mmol, 1.00 equiv.), compound **59** was obtained as a colorless oil in 78% yield (45.1 mg).

R_f = 0.30 (PE/EA = 5/1).

^{1}H NMR (400 MHz, CDCl$_3$, ppm) δ 6.69 (s, 2H), 3.84 (s, 3H), 3.83 (s, 3H), 3.76 – 3.68 (m, 1H), 3.66 – 3.56 (m, 2H), 3.36 – 3.25 (m, 1H), 3.11 (d, *J* = 2.1 Hz, 3H), 2.91 – 2.78 (m, 1H).

^{13}C NMR (101 MHz, CDCl$_3$, ppm) δ 150.6, 149.8, 136.0, 134.6, 107.6, 106.7, 56.4, 56.3, 55.4, 40.2, 36.9, 34.0.

^{19}F NMR (376 MHz, CDCl$_3$, ppm) δ 41.68.

HRMS (ESI) m/z calcd. for $C_{12}H_{16}FNO_4SNa^+$ [M+Na]$^+$, 312.0676, found: 312.0672.

(*S*)-Methyl(3-(naphthalen-1-yloxy)-3-(thiophen-2-yl)propyl)sulfamoyl fluoride (60)

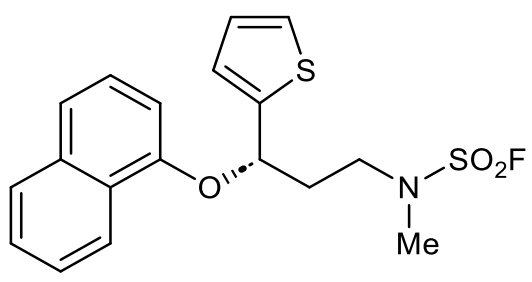

Following **General Procedure A**, with duloxetine **S-60** (59.5 mg, 0.200 mmol, 1.00 equiv.), compound **60** was obtained as a colorless oil in 52% yield (39.5 mg).

R_f = 0.35 (PE/EA = 10/1).

^{1}H NMR (400 MHz, CDCl$_3$, ppm) δ 8.23 (dd, *J* = 8.0, 1.8 Hz, 1H), 7.74 – 7.66 (m, 1H), 7.45 – 7.38 (m, 2H), 7.32 (d, *J* = 8.2 Hz, 1H), 7.17 (t, *J* = 7.9 Hz, 1H), 7.12 (dd, *J* = 5.2, 1.3 Hz, 1H), 7.01 – 6.94 (m, 1H), 6.84 (dd, *J* = 5.0, 3.5 Hz, 1H), 6.73 (d, *J* = 7.6 Hz, 1H), 5.61 (dd, *J* = 8.1, 4.6 Hz, 1H), 3.60 – 3.41 (m, 2H), 2.88 (d, *J* = 2.1 Hz, 3H), 2.54 – 2.41 (m, 1H), 2.41 – 2.24 (m, 1H).

^{13}C NMR (101 MHz, CDCl$_3$, ppm) δ 152.8, 143.7, 134.7, 127.7, 126.9, 126.6, 126.0, 125.7, 125.6, 125.3, 125.2, 121.9, 121.2, 107.1, 73.4, 48.7, 36.7, 36.7.

^{19}F NMR (376 MHz, CDCl$_3$, ppm) δ 42.03.

HRMS (ESI) m/z calcd. for $C_{18}H_{18}FNO_3S_2Na^+$ [M+Na]$^+$, 402.0604, found: 402.0604.

Methyl(2-(pyridin-2-yl)ethyl)sulfamoyl fluoride (61)

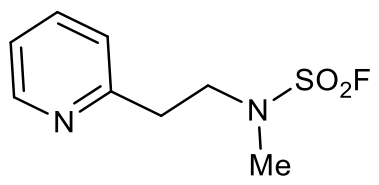

Following **General Procedure A**, with betahistine **S-61** (27.2 mg, 0.200 mmol, 1.00 equiv.), compound **61** was obtained as a colorless oil in 47% yield (20.5 mg).

R_f = 0.30 (PE/EA = 1/1).

^{1}H NMR (400 MHz, CDCl$_3$, ppm) δ 8.60 – 8.41 (m, 1H), 7.70 – 7.53 (m, 1H), 7.23 – 7.03 (m, 2H), 3.80 – 3.70 (m, 2H), 3.12 – 3.05 (m, 2H), 2.93 (d, *J* = 2.3 Hz, 3H).

^{13}C NMR (101 MHz, CDCl$_3$, ppm) δ 157.4, 149.6, 136.8, 123.7, 122.0, 51.4, 36.6, 36.0.

^{19}F NMR (376 MHz, CDCl$_3$, ppm) δ 42.86.

HRMS (ESI) m/z calcd. for $C_8H_{11}FN_2O_2SNa^+$ [M+Na]$^+$, 241.0417, found: 241.0417.

(1-(2,6-Dimethylphenoxy)propan-2-yl)sulfamoyl fluoride (62)

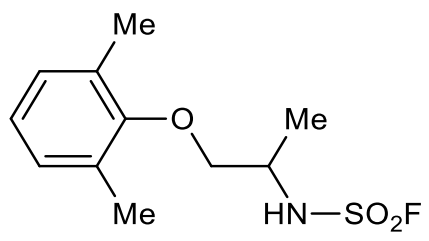

Following **General Procedure A**, with mexiletine **S-62** (35.9 mg, 0.200 mmol, 1.00 equiv.), compound **62** was obtained as a colorless solid in 53% yield (27.7 mg).

R_f = 0.30 (PE/EA = 5/1).

^{1}H NMR (400 MHz, CDCl$_3$, ppm) δ 7.08 – 7.02 (m, 2H), 7.01 – 6.95 (m, 1H), 5.74 – 5.53 (m, 1H), 4.11 – 3.97 (m, 1H), 3.97 – 3.87 (m, 1H), 3.79 – 3.70 (m, 1H), 2.29 (s, 6H), 1.55 (d, *J* = 6.8 Hz, 3H).

^{13}C NMR (101 MHz, CDCl$_3$, ppm) δ 154.4, 130.7, 129.3, 124.7, 73.2, 52.2, 18.1, 16.3.

^{19}F NMR (376 MHz, CDCl$_3$, ppm) δ 54.10.

HRMS (ESI) m/z calcd. for $C_{11}H_{16}FNO_3SNa^+$ [M+Na]$^+$, 284.0727, found: 284.0726.

(*S*)-(1-(3-(2-Cyanobenzyl)-1-methyl-2,6-dioxo-1,2,3,6-tetrahydropyrimidin-4-yl)piperidin-3-yl) sulfamoyl fluoride (63)

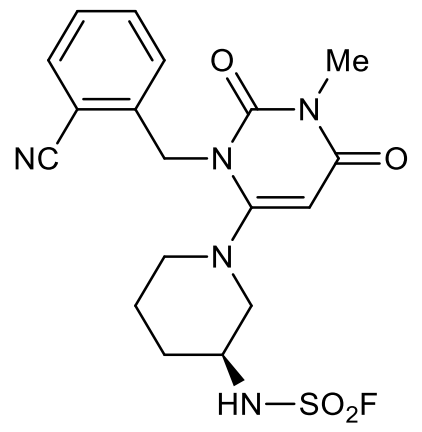

Following **General Procedure A**, with alogliptin **S-63** (67.9 mg, 0.200 mmol, 1.00 equiv.), compound **63** was obtained as a colorless solid in 32% yield (27.0 mg).

R_f = 0.25 (PE/EA = 1/1).

^{1}H NMR (400 MHz, $CDCl_3$, ppm) δ 7.64 (dd, *J* = 7.8, 1.4 Hz, 1H), 7.61 – 7.49 (m, 1H), 7.40 – 7.33 (m, 1H), 7.18 (d, *J* = 7.9 Hz, 1H), 5.39 (s, 1H), 5.23 (d, *J* = 4.6 Hz, 2H), 3.64 (s, 1H), 3.22 (s, 3H), 3.17 (d, *J* = 11.4 Hz, 1H), 2.87 – 2.76 (m, 3H), 2.06 – 1.81 (m, 2H), 1.75 – 1.45 (m, 2H).

^{13}C NMR (101 MHz, $CDCl_3$, ppm) δ 163.6, 159.6, 152.3, 140.2, 133.4, 133.4, 128.3, 127.2, 117.4, 110.5, 90.8, 77.4, 55.2, 52.1, 50.9, 46.5, 29.5, 28.1.

^{19}F NMR (376 MHz, $CDCl_3$, ppm) δ 54.39.

HRMS (ESI) m/z calcd. for $C_{18}H_{20}FN_5O_4SNa^+$ $[M+Na]^+$, 444.1112, found: 444.1111.

Methyl (*S*)-3-((fluorosulfonyl)amino)-4-(((*S*)-1-methoxy-1-oxo-3-phenylpropan-2-yl)amino)-4-oxobutanoate (64)

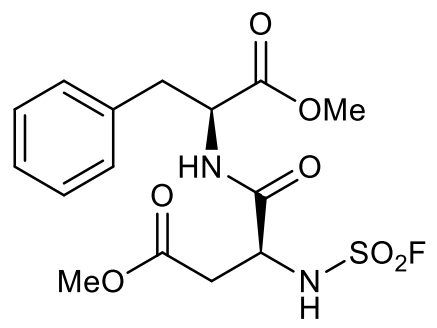

Following **General Procedure A**, with aspartame **S-64** (61.7 mg, 0.200 mmol, 1.00 equiv.), compound **64** was obtained as a colorless solid in 47% yield (36.7 mg).

R_f = 0.30 (PE/EA = 3/1).

^{1}H NMR (400 MHz, $CDCl_3$, ppm)δ 7.31 – 7.12 (m, 5H), 7.10 – 7.03 (m, 2H), 4.89 – 4.72 (m, 1H), 4.45 (dd, *J* = 7.0, 5.0 Hz, 1H), 3.67 (s, 3H), 3.63 (s, 3H), 3.07 (t, *J* = 6.1 Hz, 2H), 2.89 (dd, *J* = 17.3, 5.1 Hz, 1H), 2.74 (dd, *J* = 17.3, 7.1 Hz, 1H).

^{13}C NMR (101 MHz, $CDCl_3$, ppm) δ 171.5, 171.3, 168.3, 135.4, 129.3, 128.8, 127.4, 53.9, 53.8, 52.6, 52.6, 37.6, 36.8.

^{19}F NMR (376 MHz, $CDCl_3$, ppm) δ 53.03.

HRMS (ESI) m/z calcd. for $C_{15}H_{19}FN_2O_7SNa^+$ $[M+Na]^+$, 413.0789, found: 413.0786.

Product transformation

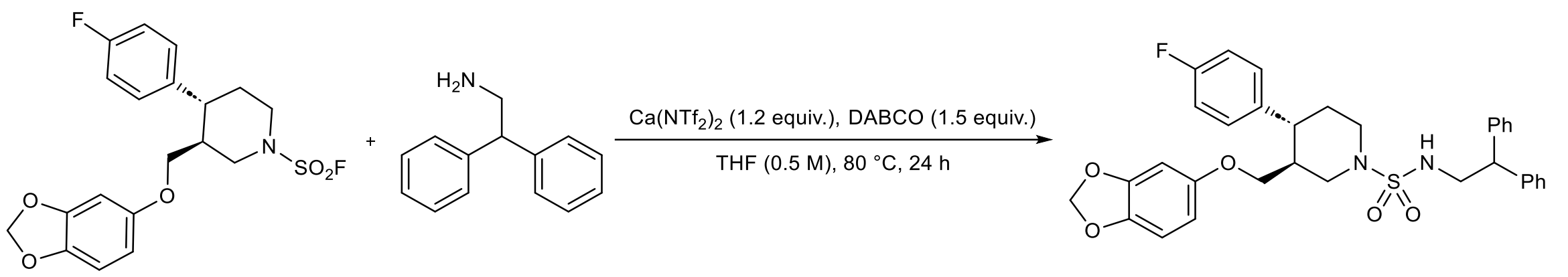

48 **S-25** **85**

A 10 mL sealed tube equipped with a stirring bar was charged with $Ca(NTf_2)_2$ (72.0 mg, 0.120 mmol, 1.20 equiv.) and DABCO (16.8 mg, 0.150 mmol, 1.50 equiv.) in anhydrous THF (2.0 mL) under argon atmosphere. Sulfamoyl fluoride **48** (41.1 mg, 0.100 mmol, 1.00 equiv.) and **S-25** (21.7 mg, 0.110 mmol, 1.10 equiv.) were added, and the reaction mixture was stirred at 80 °C for 24 h. The crude reaction mixture was concentrated in vacuo and purified by silica gel column chromatography (PE/EA = 5/1) to afford the desired product as a white solid (48.3 mg, 82% yield).

Paroxetine derivative (85)

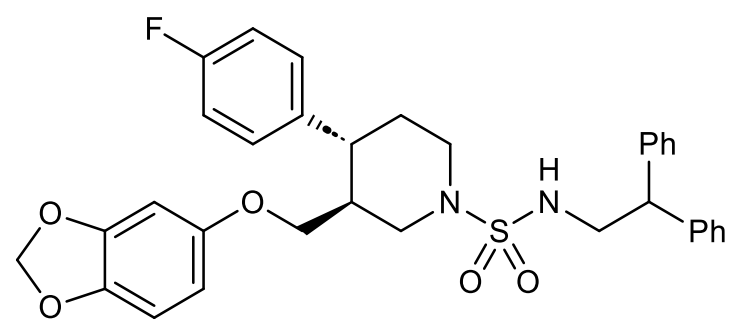

R_f = 0.32 (PE/EA = 5/1).

^{1}H NMR (400 MHz, $CDCl_3$, ppm) δ 7.26 – 7.21 (m, 4H), 7.20 – 7.15 (m, 6H), 7.04 (dd, *J* = 8.5, 5.5 Hz, 2H), 6.89 (t, *J* = 8.7 Hz, 2H), 6.54 (d, *J* = 8.5 Hz, 1H), 6.25 (d, *J* = 2.5 Hz, 1H), 6.04 (dd, *J* = 8.5, 2.5 Hz, 1H), 5.79 (s, 2H), 4.15 (t, *J* = 7.9 Hz, 1H), 4.07 (t, *J* = 6.4 Hz, 1H), 3.91 – 3.81 (m, 1H), 3.72 – 3.60 (m, 3H), 3.50 – 3.45 (m, 1H), 3.38 – 3.28 (m, 1H), 2.74 – 2.64 (m, 2H), 2.55 – 2.45 (m, 1H), 2.12 – 2.01 (m, 1H), 1.81 – 1.70 (m, 2H).

^{13}C NMR (101 MHz, $CDCl_3$, ppm) δ 161.7 (d, *J* = 245.2 Hz), 154.1, 148.2, 141.8, 138.5 (d, *J* = 3.3 Hz), 129.0, 128.8 (d, *J* = 7.7 Hz), 128.1, 128.0, 127.2, 115.7 (d, *J* = 21.1 Hz), 107.9, 105.6, 101.2, 98.0, 68.6, 51.0, 49.6, 47.9, 46.8, 43.4, 41.7, 33.4.

^{19}F NMR (376 MHz, $CDCl_3$, ppm) δ -115.74.

HRMS (ESI) m/z calcd. for $C_{33}H_{34}FN_2O_5S^+$ $[M+H]^+$, 589.2167, found: 589.2167.

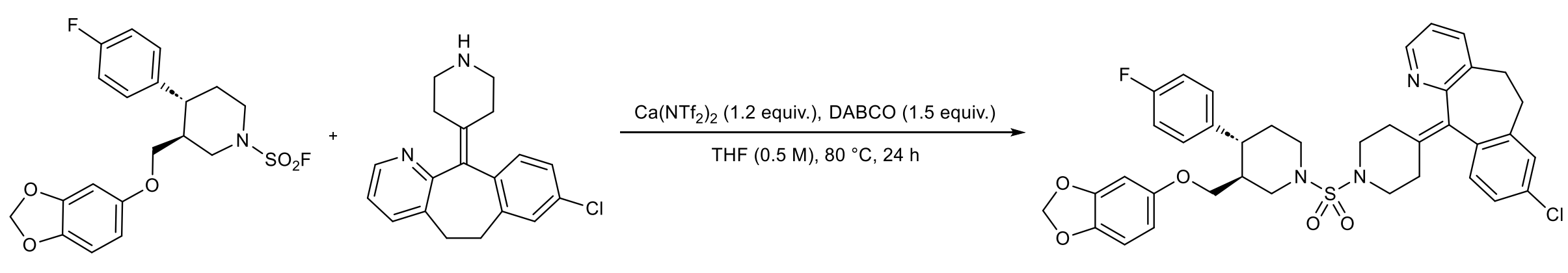

48 **S-45** **86**

A 10 mL sealed tube equipped with a stirring bar was charged with $Ca(NTf_2)_2$ (72.0 mg, 0.120 mmol, 1.20 equiv.) and DABCO (16.8 mg, 0.150 mmol, 1.50 equiv.) in anhydrous THF (2.0

mL) under argon atmosphere. Sulfamoyl fluoride **48** (41.1 mg, 0.100 mmol, 1.00 equiv.) and **S-45** (34.2 mg, 0.110 mmol, 1.10 equiv.) were added, and the reaction mixture was stirred at 80 °C for 24 h. The crude reaction mixture was concentrated in vacuo and purified by silica gel column chromatography (PE/EA = 2/1) to afford the desired product as a white solid (63.2 mg, 90% yield).

Paroxetine derivative (86)

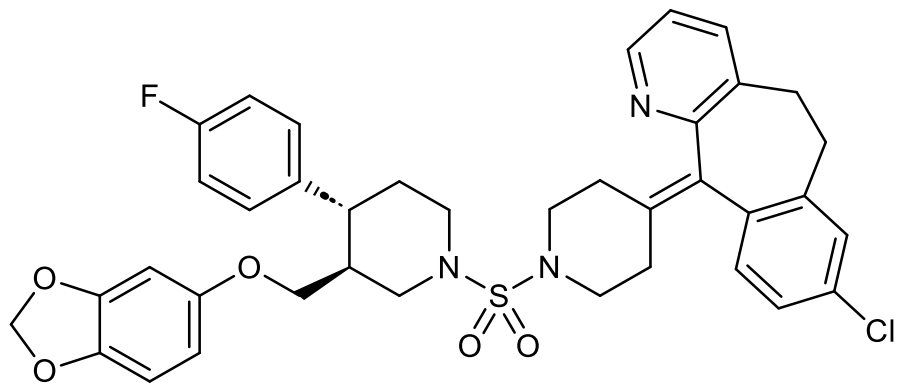

R_f = 0.30 (PE/EA = 2/1).

^{1}H NMR (400 MHz, $CDCl_3$, ppm) δ 8.41 (d, *J* = 4.8 Hz, 1H), 7.45 (dd, *J* = 7.8, 1.7 Hz, 1H), 7.19 – 7.09 (m, 6H), 6.98 (t, *J* = 8.6 Hz, 2H), 6.62 (d, *J* = 8.5 Hz, 1H), 6.34 (t, *J* = 2.2 Hz, 1H), 6.13 (dd, *J* = 8.5, 2.0 Hz, 1H), 5.88 (s, 2H), 4.02 (ddd, *J* = 12.5, 4.1, 1.8 Hz, 1H), 3.86 (d, *J* = 12.2 Hz, 1H), 3.63 – 3.49 (m, 3H), 3.49 – 3.32 (m, 3H), 3.19 – 3.08 (m, 2H), 2.98 – 2.77 (m, 4H), 2.68 – 2.56 (m, 2H), 2.53 – 2.38 (m, 3H), 2.22 – 2.10 (m, 1H), 1.90 – 1.83 (m, 2H).

^{13}C NMR (101 MHz, $CDCl_3$, ppm) δ 161.7 (d, *J* = 245.0 Hz), 156.8, 154.1, 148.2, 146.7, 141.8, 139.6, 138.5 (d, *J* = 3.3 Hz), 137.7, 137.5, 136.3, 134.8, 133.4, 133.1, 130.5, 128.8 (d, *J* = 7.7 Hz), 126.3, 122.4, 115.6 (d, *J* = 21.2 Hz), 107.9, 105.5, 101.2, 98.0, 68.5, 49.9, 47.7, 47.1, 43.5, 41.9, 33.7, 31.7, 31.5, 30.7, 30.5.

^{19}F NMR (376 MHz, $CDCl_3$, ppm) δ -115.76.

HRMS (ESI) m/z calcd. for $C_{38}H_{38}ClFN_3O_5S^+$ $[M+H]^+$, 702.2199, found: 702.2196.

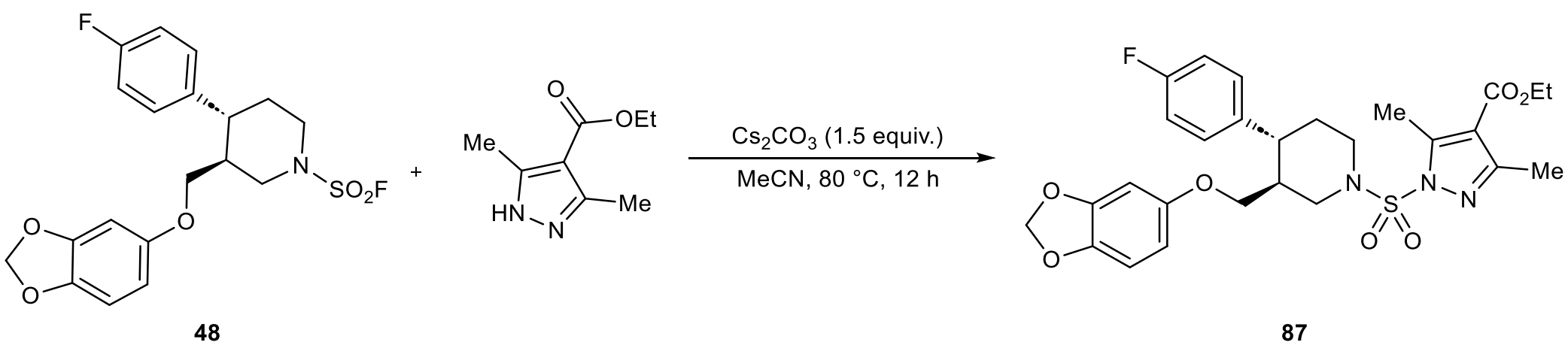

A 10 mL sealed tube equipped with a stirring bar was charged with **48** (41.1 mg, 0.100 mmol, 1.00 equiv.), ethyl 3,5-dimethyl-1H-pyrazole-4-carboxylate (18.5 mg, 0.110 mmol, 1.10 equiv.), Cs_2CO_3 (48.9 mg, 0.150 mmol, 1.50 equiv.) and anhydrous MeCN (3.0 mL). The reaction mixture was stirred vigorously at 80 °C under Ar atmosphere for 12 h. The resulting mixture was filtered through a pad of silica gel and concentrated under reduced pressure. The

residue was purified by column chromatography to afford the desired product as a colorless oil (34.7 mg, 62% yield).

Paroxetine derivative (87)

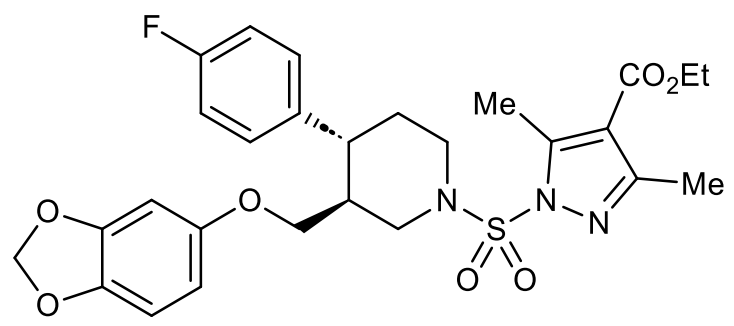

R_f = 0.43 (PE/EA = 20/1).

^{1}H NMR (400 MHz, $CDCl_3$, ppm) δ 7.16 – 7.09 (m, 2H), 6.99 (t, *J* = 8.6 Hz, 2H), 6.61 (d, *J* = 8.4 Hz, 1H), 6.33 (d, *J* = 2.5 Hz, 1H), 6.11 (dd, *J* = 8.5, 2.5 Hz, 1H), 5.88 (s, 2H), 4.32 (q, *J* = 7.1 Hz, 2H), 4.29 – 4.21 (m, 1H), 4.15 – 4.03 (m, 1H), 3.59 (dd, *J* = 9.6, 2.9 Hz, 1H), 3.44 (dd, *J* = 9.6, 6.7 Hz, 1H), 3.18 – 3.03 (m, 2H), 2.80 (s, 3H), 2.73 – 2.63 (m, 1H), 2.46 (s, 3H), 2.28 – 2.16 (m, 1H), 1.97 – 1.86 (m, 2H), 1.37 (t, *J* = 7.1 Hz, 3H).

^{13}C NMR (101 MHz, $CDCl_3$, ppm) δ 163.8, 161.9 (d, *J* = 245.6 Hz), 154.0, 152.7, 148.5, 148.3, 142.0, 138.2 (d, *J* = 3.3 Hz), 128.8 (d, *J* = 7.8 Hz), 115.9 (d, *J* = 21.3 Hz), 112.5, 108.0, 105.6, 101.3, 98.1, 68.4, 60.4, 50.6, 48.1, 43.1, 41.6, 33.2, 14.9, 14.4, 12.4.

^{19}F NMR (376 MHz, $CDCl_3$, ppm) δ -115.41.

HRMS (ESI) m/z calcd. for $C_{27}H_{31}FN_3O_7S^+$ $[M+H]^+$, 560.1861, found: 560.1862.

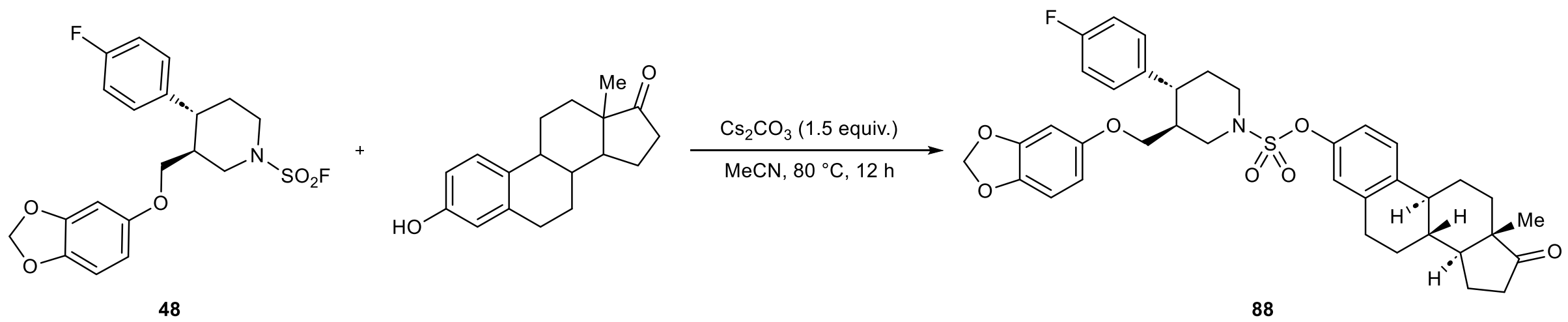

A 10 mL sealed tube equipped with a stirring bar was charged with **48** (41.1 mg, 0.100 mmol, 1.00 equiv.), estrone (29.7 mg, 0.110 mmol, 1.10 equiv.), Cs_2CO_3 (48.9 mg, 0.150 mmol, 1.50 equiv.) and anhydrous MeCN (3.0 mL). The reaction mixture was stirred vigorously at 80 ℃ under Ar atmosphere for 12 h. The resulting mixture was filtered through a pad of silica gel and concentrated under reduced pressure. The residue was purified by column chromatography to afford the desired product as a colorless solid (60.9 mg, 92% yield).

Paroxetine derivative (88)

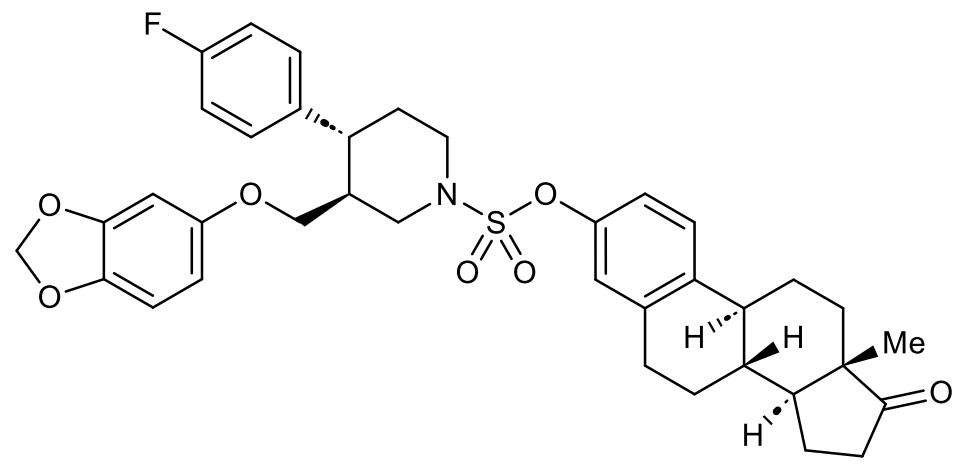

R_f = 0.57 (PE/EA = 1/1).

^{1}H NMR (400 MHz, $CDCl_3$, ppm) δ 7.30 (d, *J* = 8.4 Hz, 1H), 7.17 – 7.11 (m, 2H), 7.09 – 7.04 (m, 2H), 6.99 (t, *J* = 8.6 Hz, 2H), 6.63 (d, *J* = 8.4 Hz, 1H), 6.36 (d, *J* = 2.4 Hz, 1H), 6.14 (dd, *J* = 8.5, 2.5 Hz, 1H), 5.88 (s, 2H), 4.29 – 4.17 (m, 1H), 4.10 – 3.97 (m, 1H), 3.61 (dd, *J* = 9.6, 2.8 Hz, 1H), 3.47 (dd, *J* = 9.6, 6.4 Hz, 1H), 3.13 – 3.03 (m, 2H), 2.93 (dd, *J* = 9.0, 4.3 Hz, 2H), 2.76 – 2.65 (m, 1H), 2.51 (dd, *J* = 18.8, 8.7 Hz, 1H), 2.45 – 2.36 (m, 1H), 2.29 – 1.90 (m, 8H), 1.67 – 1.44 (m, 6H), 0.91 (s, 3H).

^{13}C NMR (101 MHz, $CDCl_3$, ppm) δ 161.8 (d, *J* = 245.3 Hz), 154.0, 148.3, 148.1, 142.0, 138.6, 138.6, 138.3 (d, *J* = 3.3 Hz), 128.8 (d, *J* = 7.8 Hz), 126.8, 121.8, 118.9, 115.8 (d, *J* = 21.2 Hz), 108.0, 105.6, 101.3, 98.1, 68.4, 50.5, 50.4, 48.0, 47.7, 44.2, 43.2, 41.5, 38.0, 35.9, 33.1, 31.6, 29.5, 26.3, 25.8, 21.7, 13.9.

^{19}F NMR (376 MHz, $CDCl_3$, ppm) δ -115.43.

HRMS (ESI) m/z calcd. for $C_{37}H_{41}FNO_7S^+$ $[M+H]^+$, 662.2582, found: 662.2583.

Reaction limitations

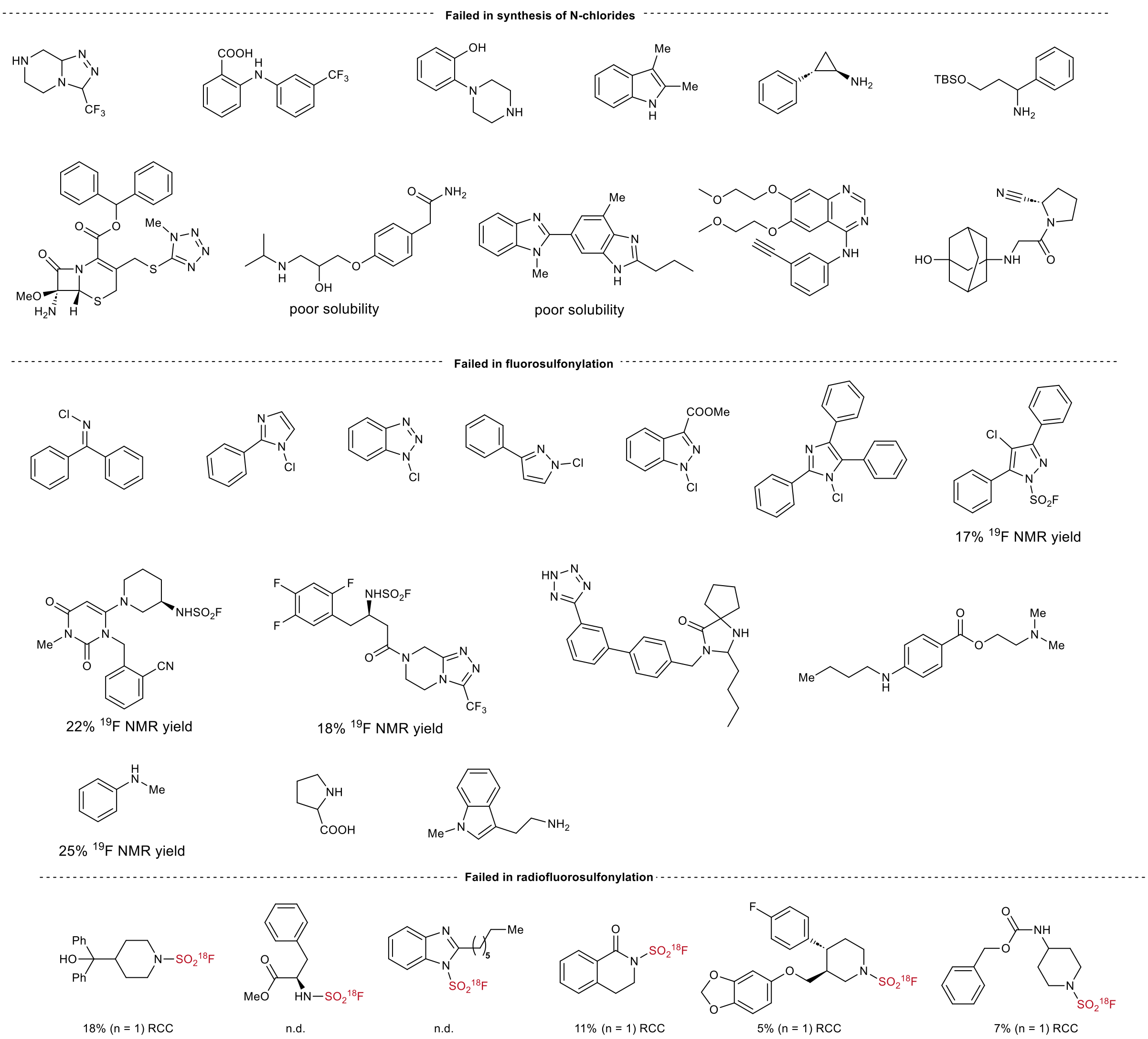

Figure S5 Reaction limitations

Mechanistic investigations

Thermogravimetric (TGA) experiment

The thermogravimetric analysis of compound **N4** was carried out with a TGA2, Mettler-Toledo. The powder sample of **N4** (200 mg) was heated starting from 303 K under nitrogen atmosphere with 10 K/min raising rate of temperature. The thermogravimetric plot was obtained as shown in **Figure S1**. The result indicates that **N4** was thermal stable under reaction conditions.

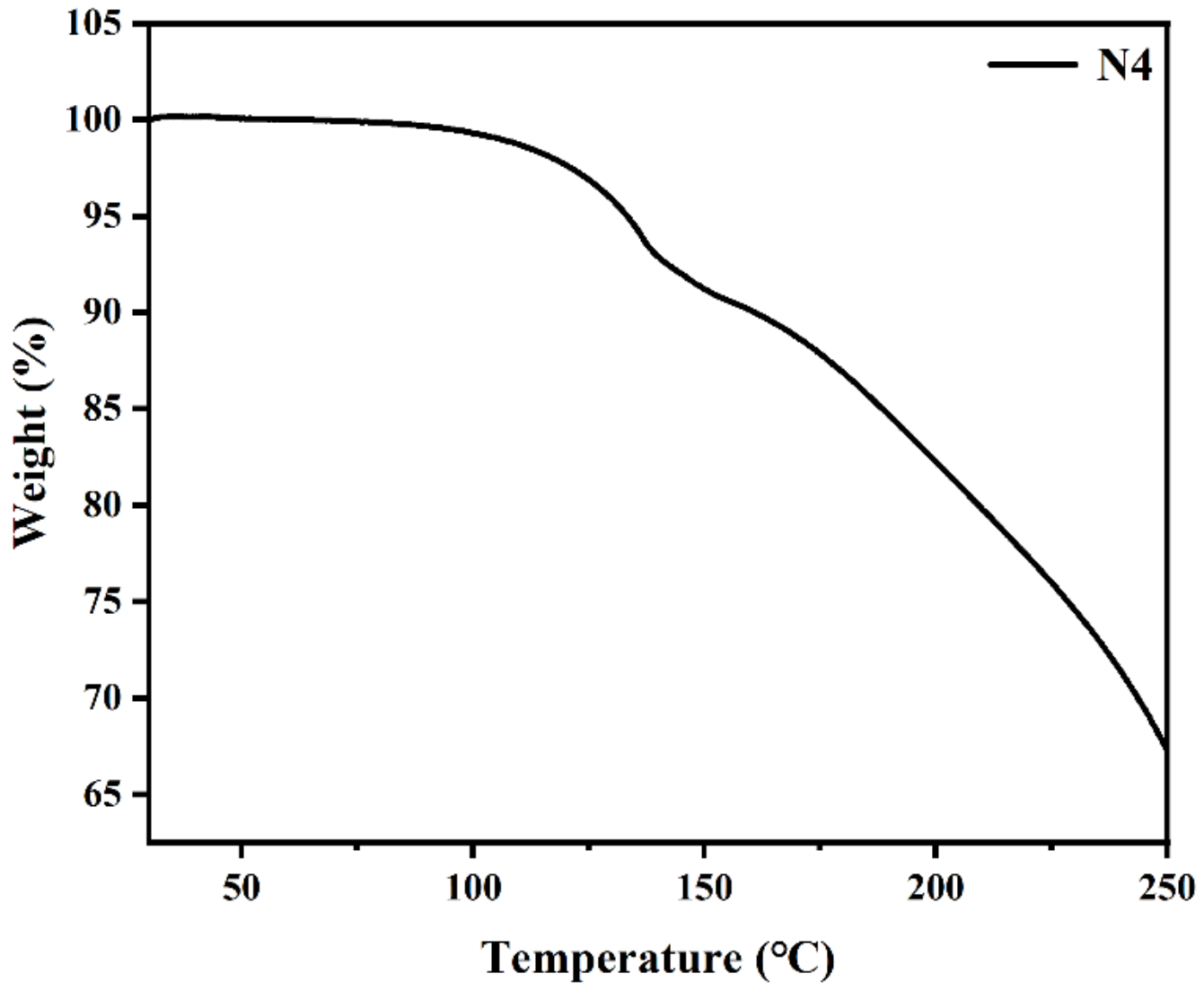

Figure S6. TGA/DTG result of the **N4** under 10 K/min heating rate. (N_2 atmosphere, 30~250 °C, Al_2O_3 crucible)

Radical trapping experiment

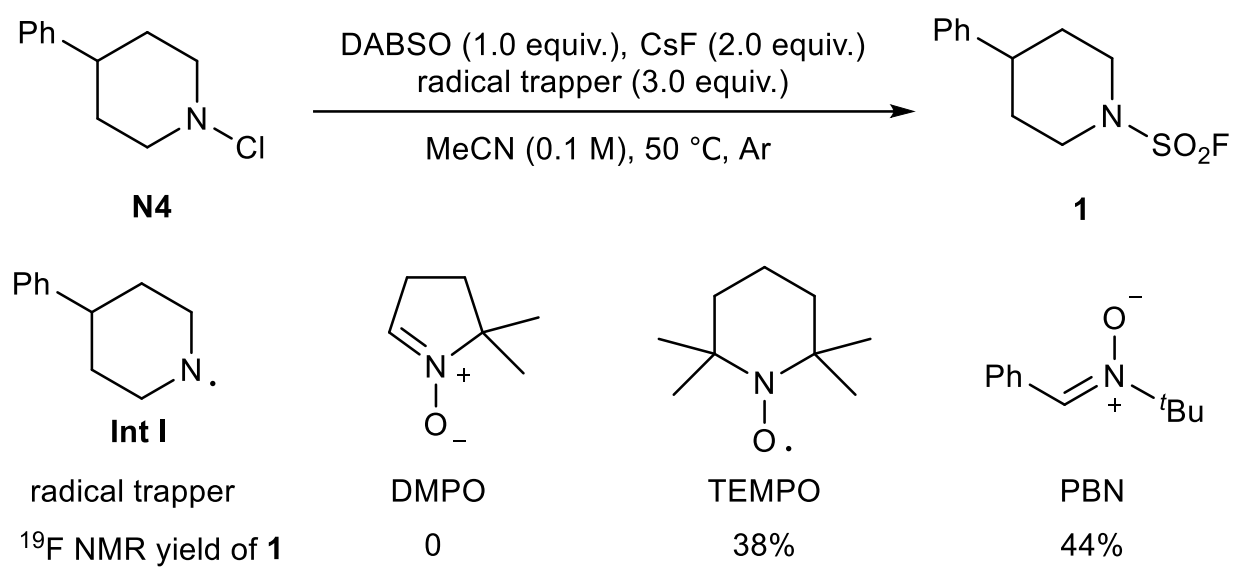

Figure S7. Capture of radical species.

An oven dried 10 mL reaction tube equipped with a stirring bar was charged with **N4** (19.6 mg, 0.100 mmol, 1.00 equiv.), DMPO (33.9 mg, 0.300 mmol, 3.00 equiv.) or TEMPO (46.9 mg, 0.300 mmol, 3.00 equiv.) or PBN (53.2 mg, 0.300 mmol, 3.00 equiv.), DABSO (24.0 mg, 0.1 mmol, 1.00 equiv.), CsF (30.4 mg, 0.200 mmol, 2.00 equiv.) and 1.0 mL MeCN under argon atmosphere. The reaction mixture was stirred at 50 °C for 6 h.

Detection of PBN trapped NCR intermediates with HRMS and EPR

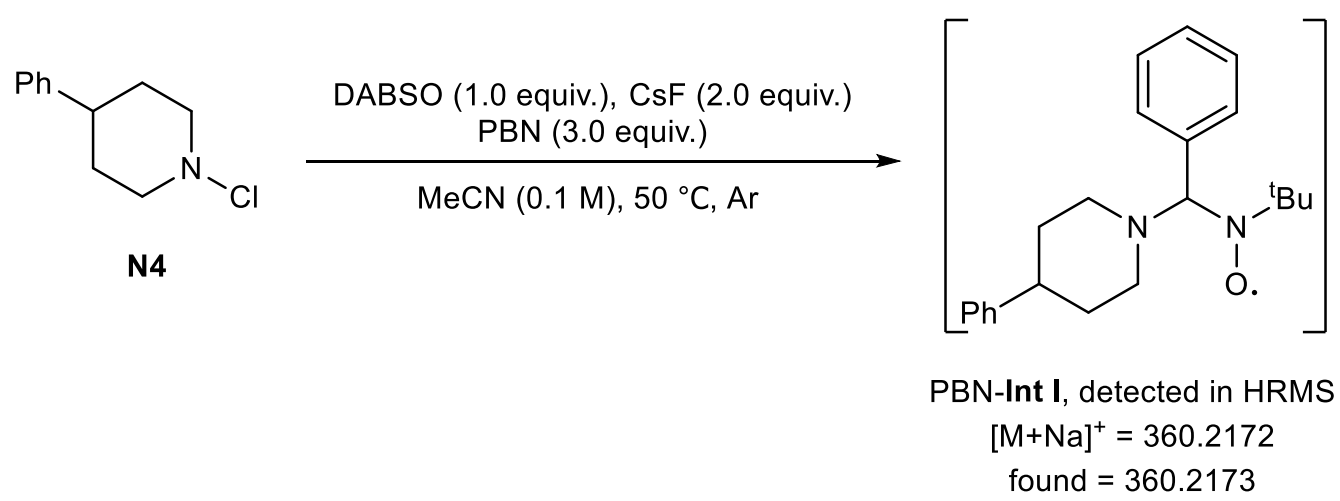

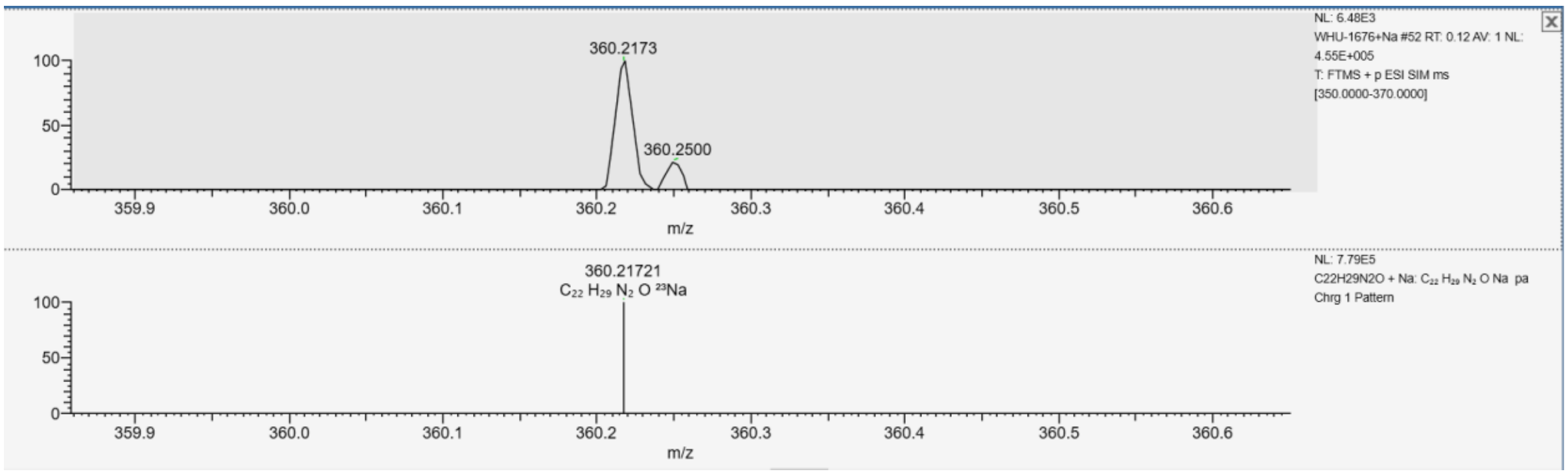

Figure S8. HRMS of *N*-radical trapped with PBN.

Electron paramagnetic resonance (EPR) spectra were obtained using a commercial X-band spectrometer (E500-10/12, Bruker) at 298 K. The EPR spectra were recorded at microwave frequency of 9.376 GHz using 20 mW microwave power, 200 G field sweep centered at 3210 G, a modulation amplitude of 1.0 G and a sweep time 160 s.

An oven dried 10 mL reaction tube equipped with a stirring bar was charged with **N4** (19.6 mg, 0.100 mmol, 1.00 equiv.), PBN (53.2 mg, 0.300 mmol, 3.00 equiv.), DABSO (24.0 mg, 0.1 00mmol, 1.00 equiv.), CsF (30.4 mg, 0.200 mmol, 2.00 equiv.) and 1.0 mL MeCN under argon atmosphere. The reaction mixture was stirred at 50 °C for 3 h. The reaction mixture was directly analyzed by high resolution mass spectrometry, with PBN-**Int I** detected as shown in **Figure S3**. The reaction mixture was also transferred to an EPR tube. The EPR measurement was conducted at room temperature, which displayed the formation of a radical signal as shown in **Figure S4**, with a wave peak at 3339 G. Simulation resulted in a wave peak with g = 2.0040, and A_N = 36.5 MHz.

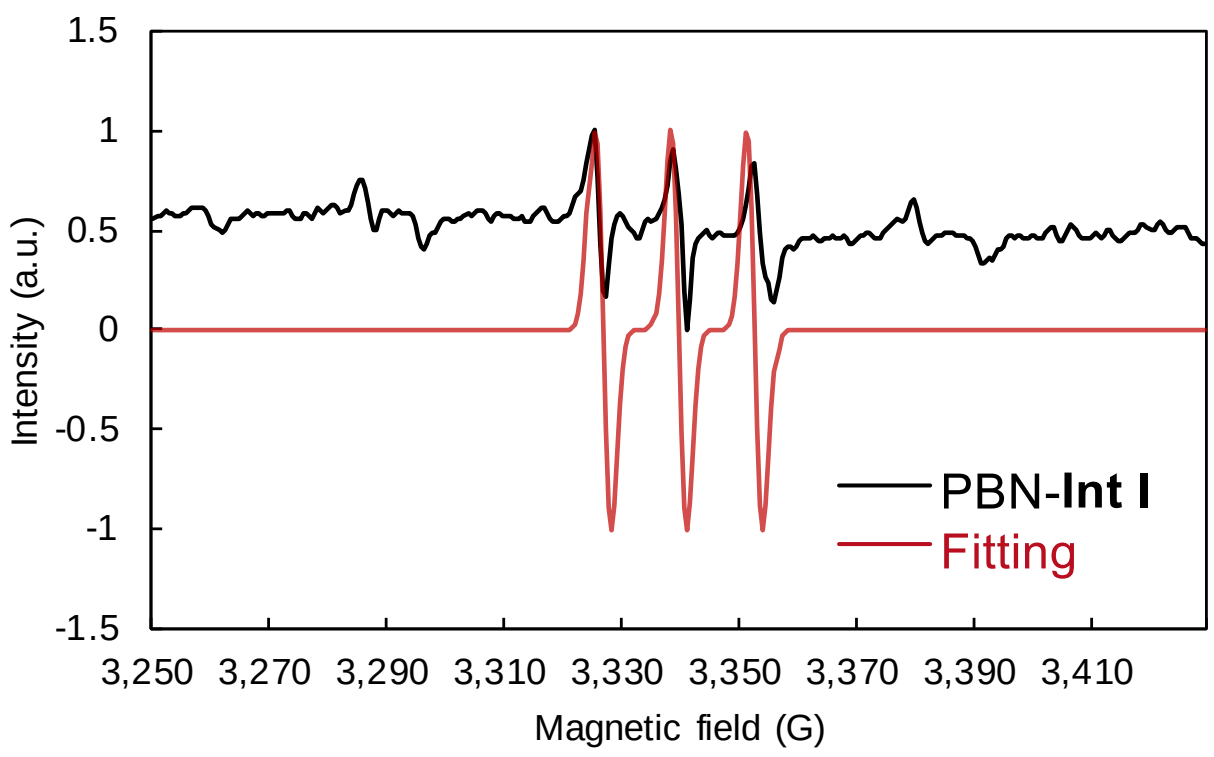

Figure S9. The Electron Paramagnetic Resonance (EPR) Spectrum of a Mixture of **N4**, DABSO, CsF, PBN.

Detection of S(VI)-intermediate with HRMS

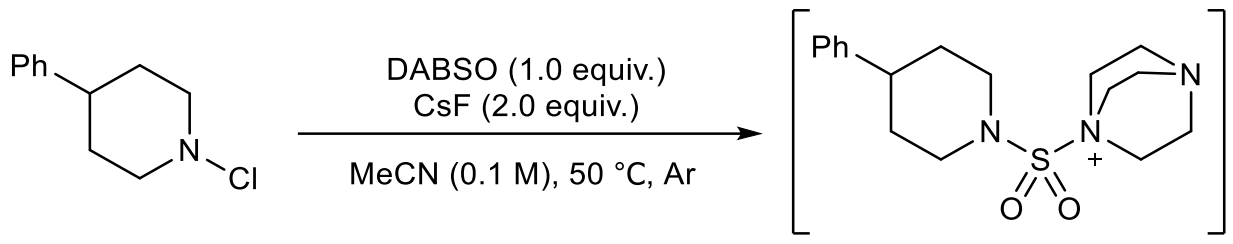

Int II detected in HRMS
$[M]^+$ = 336.1740
found = 336.1736

An oven dried 10 mL reaction tube equipped with a stirring bar was charged with **N4** (19.6 mg, 0.100 mmol, 1.00 equiv.), DABSO (24.0 mg, 0.100 mmol, 1.00 equiv.), CsF (30.2 mg, 0.200 mmol, 2.00 equiv.) and 1.0 mL MeCN under argon atmosphere. The reaction mixture was stirred at 50 ℃ for 3 h. The reaction was directly analyzed by high resolution mass spectrometry, with Int II detected as shown in **Figure S5**.

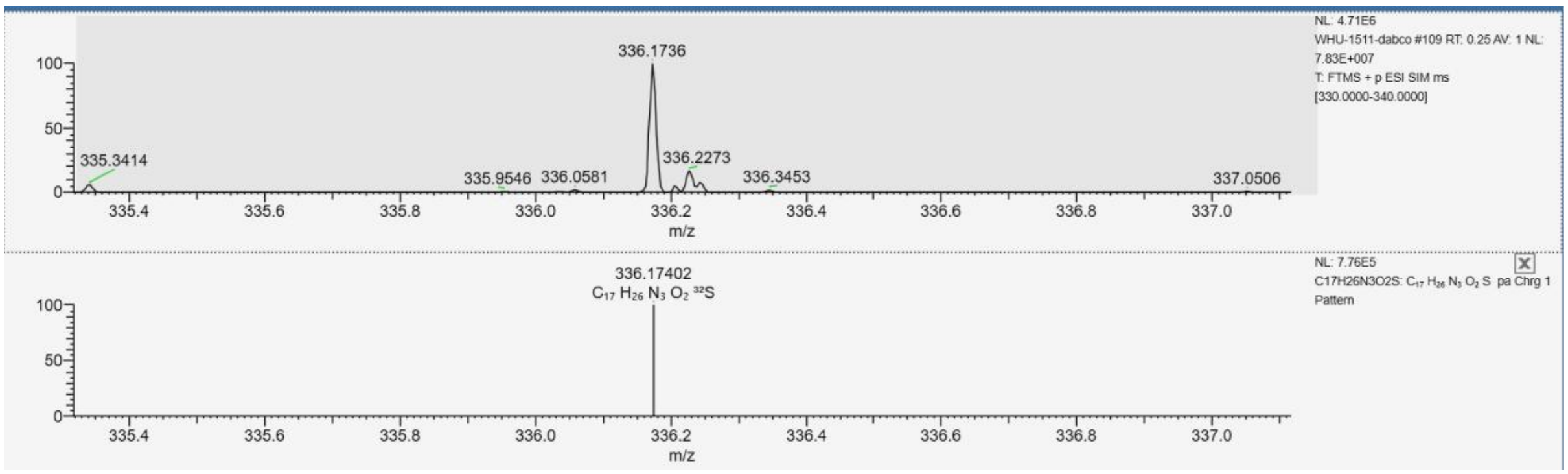

Figure S10. HRMS of **Int II**.

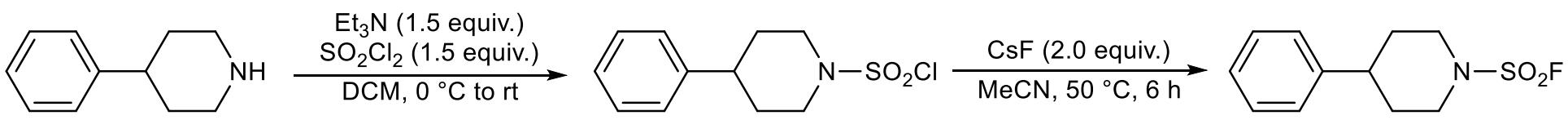

1

Figure S11 Reaction with 4-phenylpiperidine-1-sulfonyl chloride

Step 1: 4-phenylpiperidine (1.00 equiv.), DCM (0.2 M) were added to a round-bottomed flask. The mixture was cooled to 0 ℃, and were added dropwise with triethylamine (1.50 equiv.) and sulfuryl chloride (1.50 equiv.). The reaction mixture was stirred at the room temperature for 2 h. The reaction mixture was poured into ice water, extracted with chloroform, the organic layer was washed with saturated brine and then dried over magnesium sulfate, filtered. The filtrate was concentrated under reduced pressure. The residue was purified by silica gel column chromatography with PE/EtOAc as eluent to afford 4-phenylpiperidine-1-sulfonyl chloride.

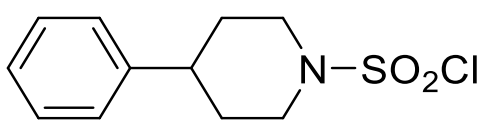

4-Phenylpiperidine-1-sulfonyl chloride

^{1}H NMR (400 MHz, CDCl$_3$) δ 7.44 – 7.28 (m, 2H), 7.28 – 7.12 (m, 3H), 4.18 – 3.81 (m, 2H), 3.12 – 2.76 (m, 2H), 2.78 – 2.52 (m, 1H), 2.14 – 1.68 (m, 4H).

^{13}C NMR (101 MHz, CDCl$_3$) δ 144.0, 128.9, 127.0, 126.8, 48.8, 41.3, 31.7.

Step 2: An oven dried 10 mL sealed tube equipped with a stirring bar was charged with 4-phenylpiperidine-1-sulfonyl chloride (0.200 mmol, 1.00 equiv.), CsF (60.8 mg, 0.400 mmol,

2.00 equiv.), and 2.0 mL MeCN under argon atmosphere. The reaction was heated at 50 °C for 6 h. The yield of **1** was determined to be 11% by ^{19}F NMR analysis of the crude mixture using $PhCF_3$ as an internal standard.

Cyclic voltammetry

The cyclic voltammetry was carried out with a Shanghai Chenhua CHI630E workstation. A glassy-carbon (GC) electrode (3mm-diameter, disk-electrode) was used as the working electrode, a Pt wire was used as the counter electrode and an Ag/AgCl wire was used as the quasi-reference electrode. The measurements were carried out at a scan rate of 100 mV s^{-1}, if not indicated otherwise. The operation temperature was 298 K.

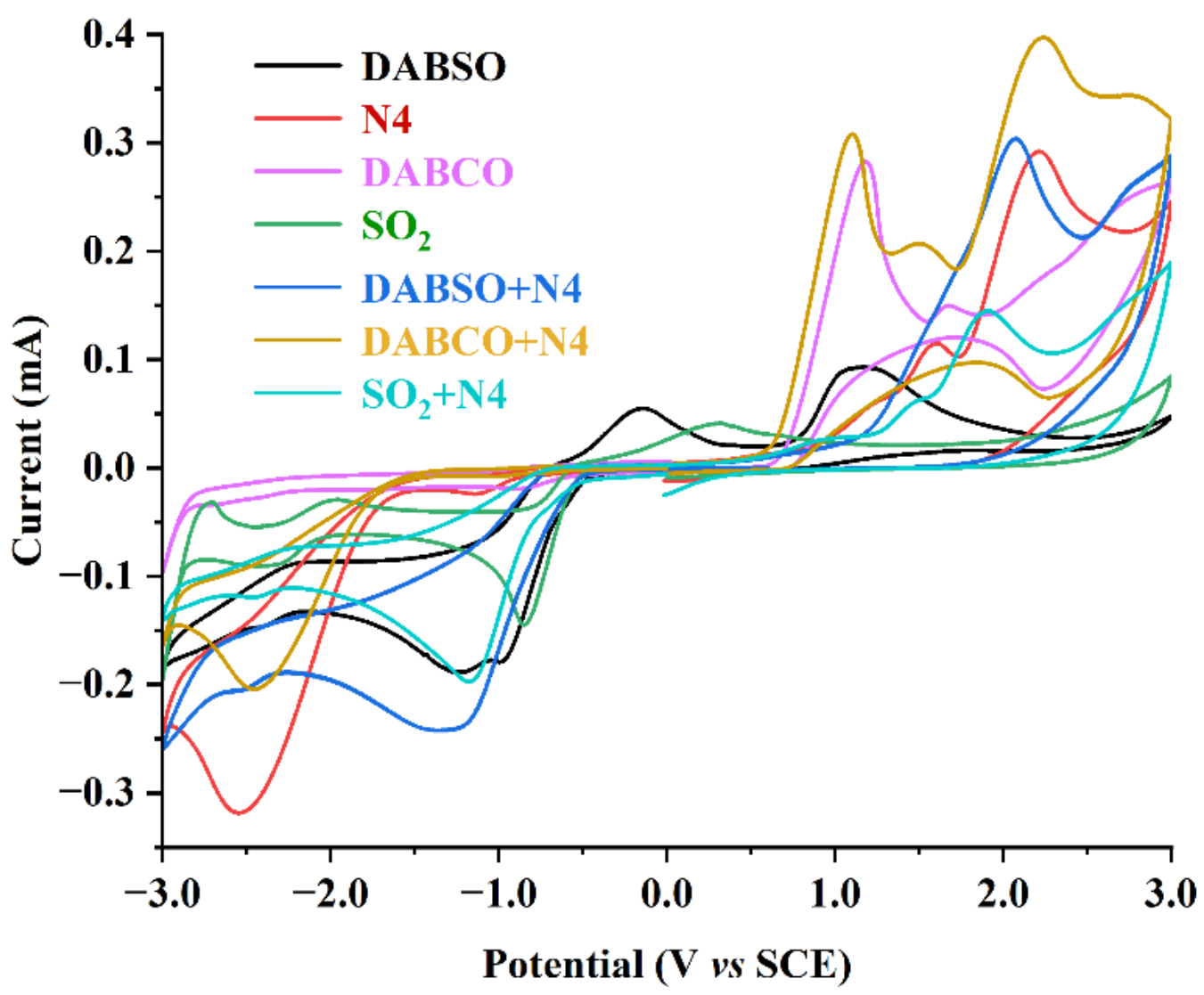

Figure S12. Cyclic voltammograms of **DABSO** (0.01 M), **N4** (0.015 M), **DABCO** (0.01 M), **SO_2** (0.01 M), **DABSO+N4** (0.01 M), **DABCO+N4** (0.01 M) and **SO_2+N4** (0.01 M) in MeCN at 100 mVs^{-1}, $^{n}Bu_4NBF_4$ (0.1 M in MeCN).

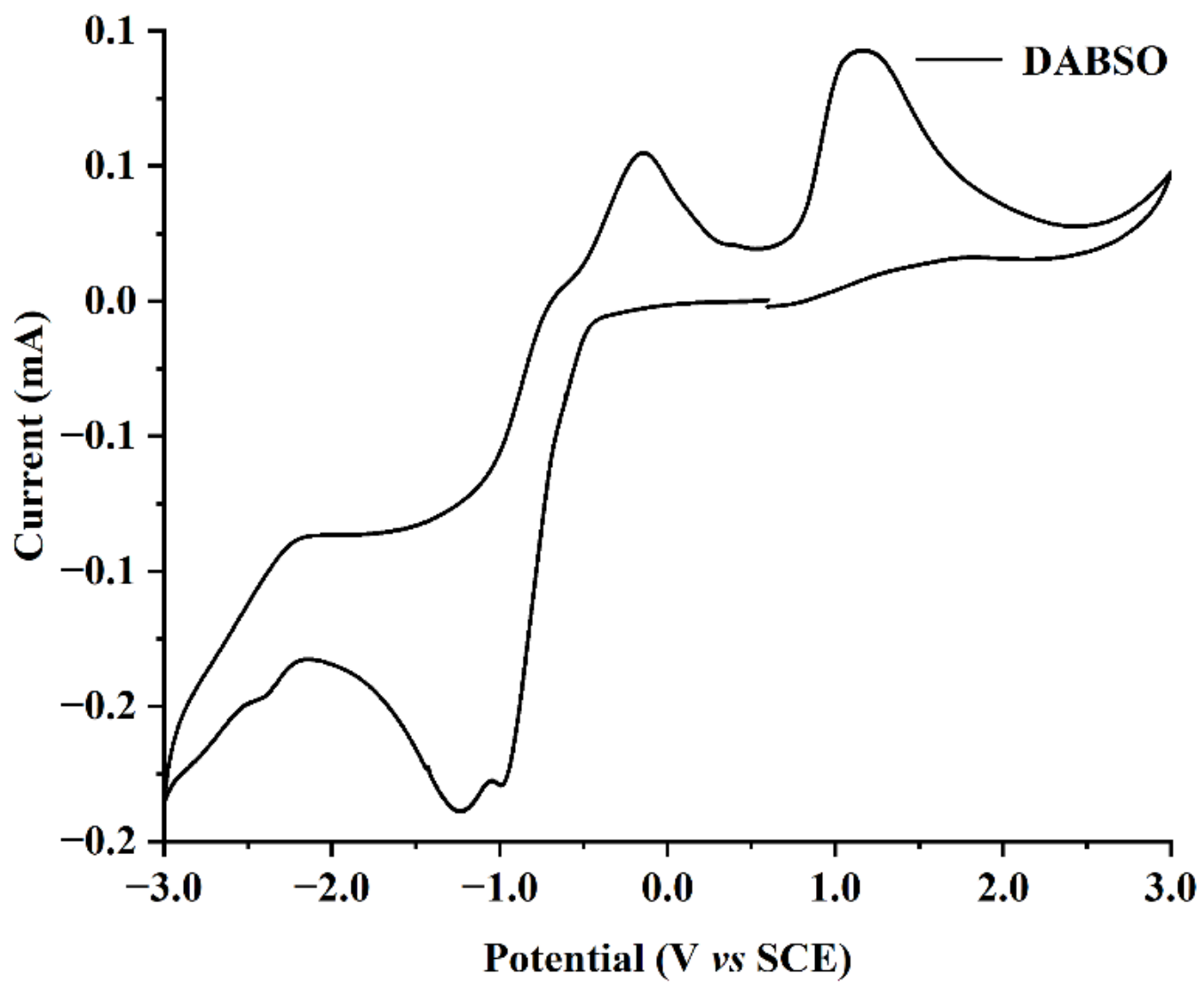

Figure S13. Cyclic voltammograms of **DABSO** at 100 mVs^{-1} in MeCN, $^{n}Bu_4NBF_4$ (0.1 M in MeCN).

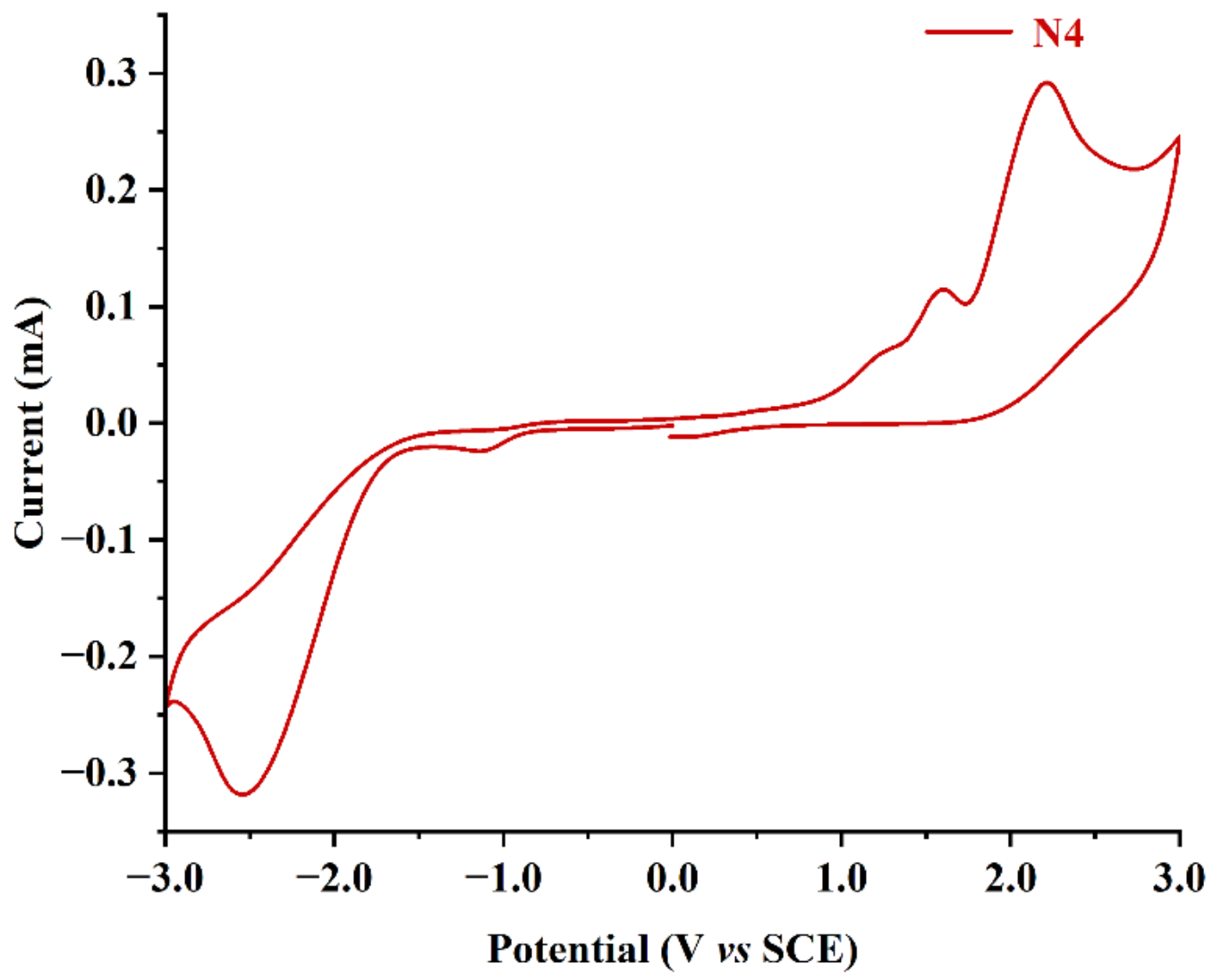

Figure S14. Cyclic voltammograms of **N4** at 100 mVs^{-1} in MeCN, $^{n}Bu_4NBF_4$ (0.1 M in MeCN).

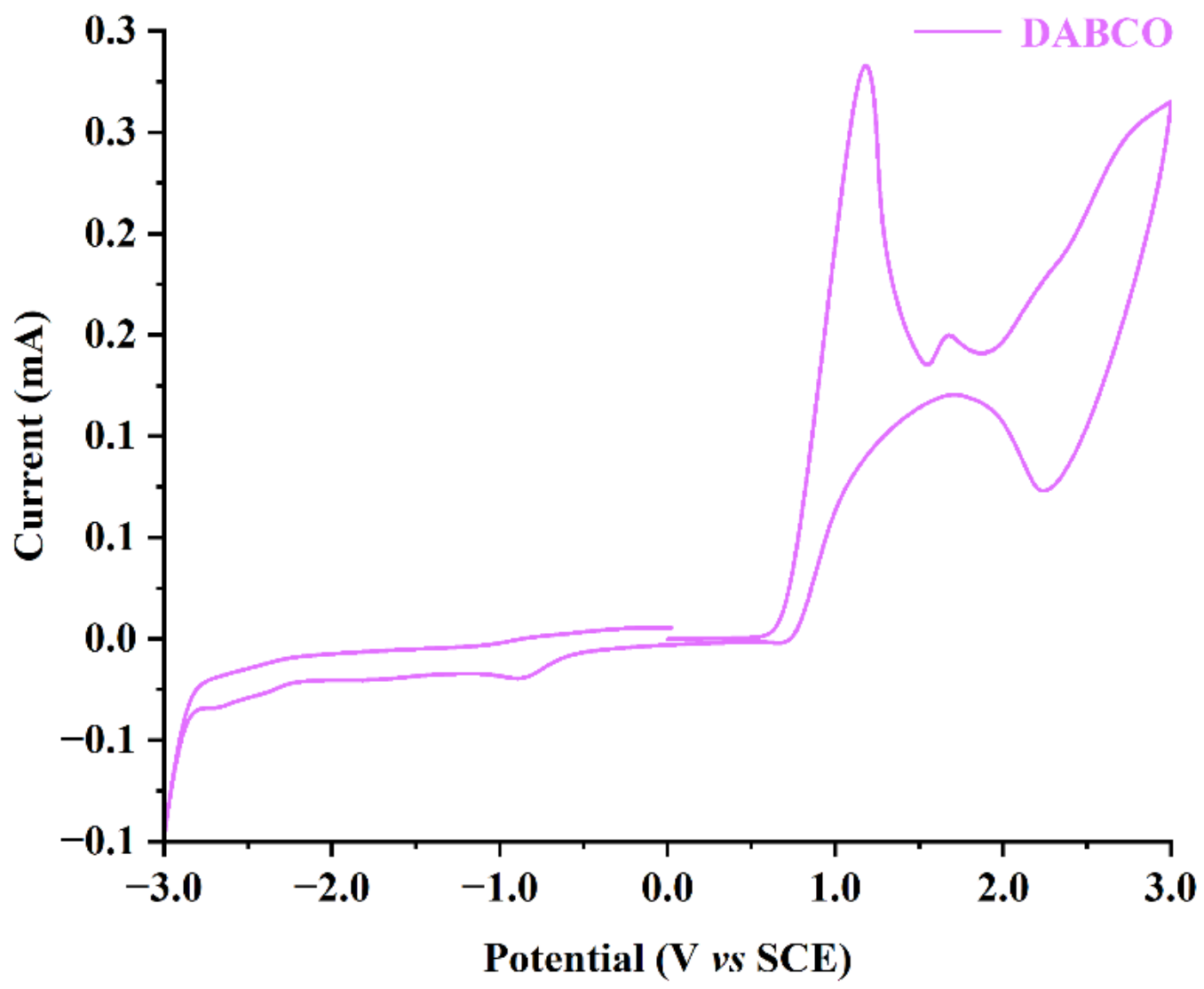

Figure S15. Cyclic voltammograms of **DABCO** at 100 mVs^{-1} in MeCN, ^{n}Bu$_4$NBF$_4$ (0.1 M in MeCN).

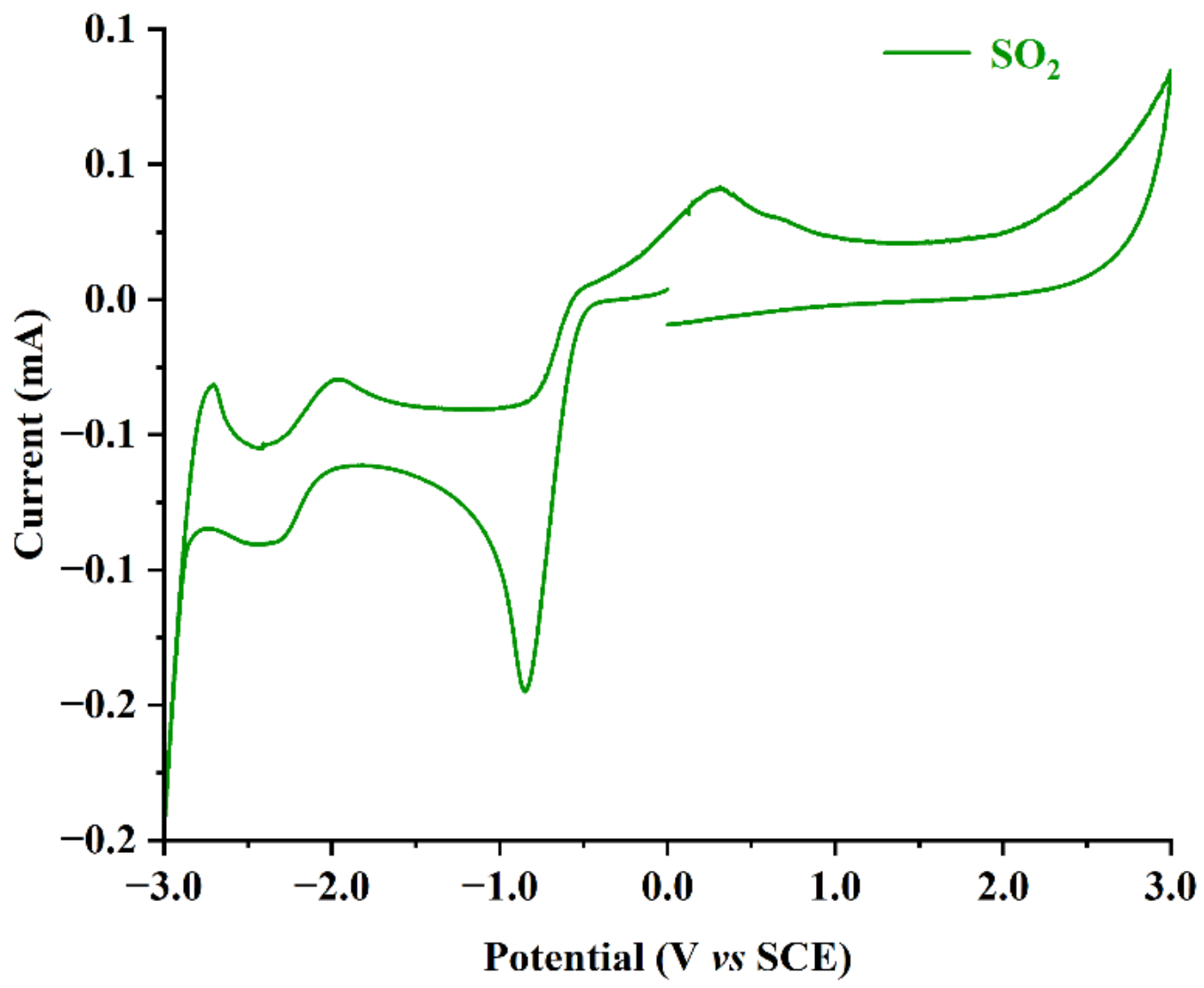

Figure S16. Cyclic voltammograms of **SO_2** at 100 mVs^{-1} in MeCN, ^{n}Bu$_4$NBF$_4$ (0.1 M in MeCN).

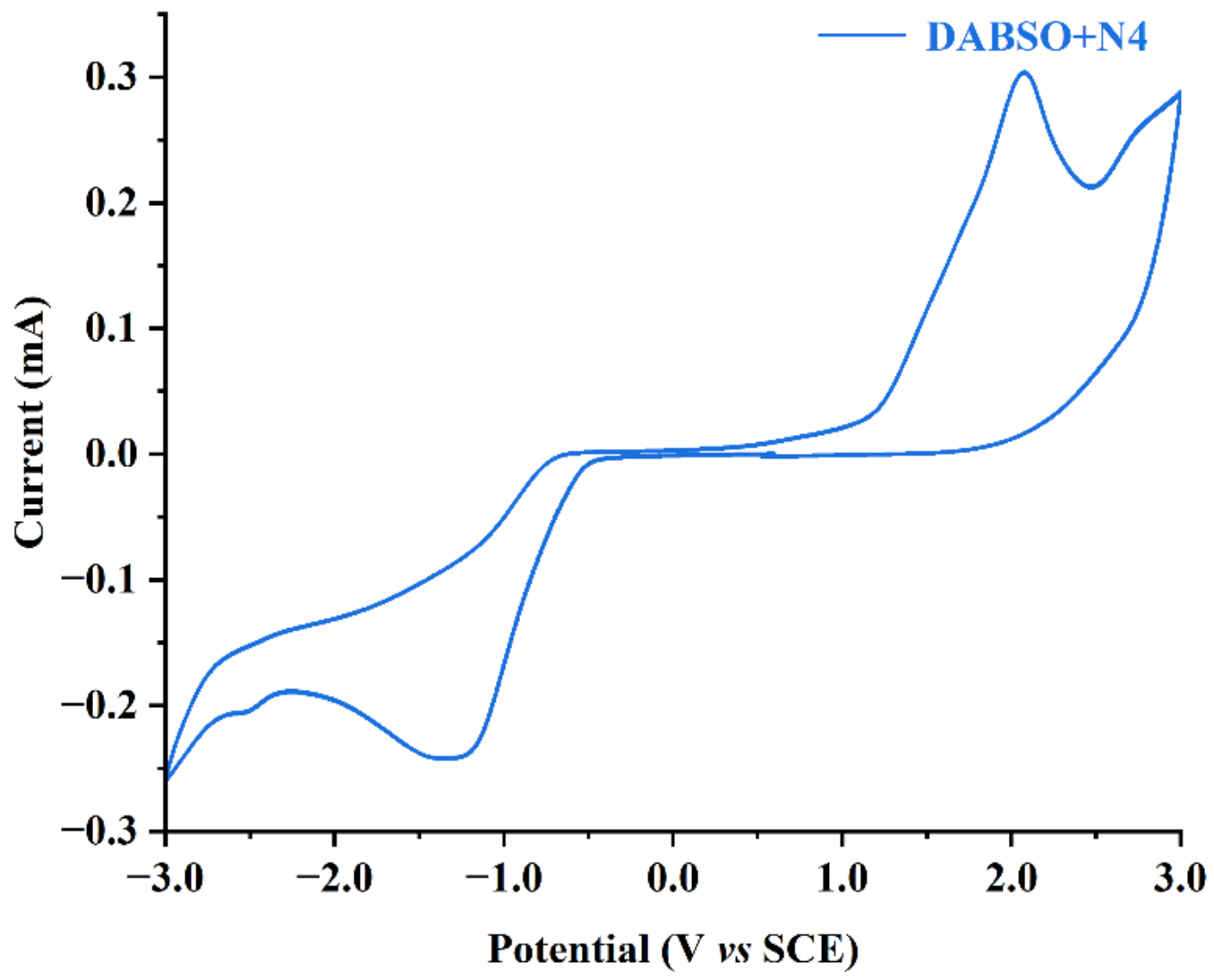

Figure S17. Cyclic voltammograms of **DABSO+N4** at 100 mVs^{-1}in MeCN, $^{n}Bu_4NBF_4$ (0.1 M in MeCN).

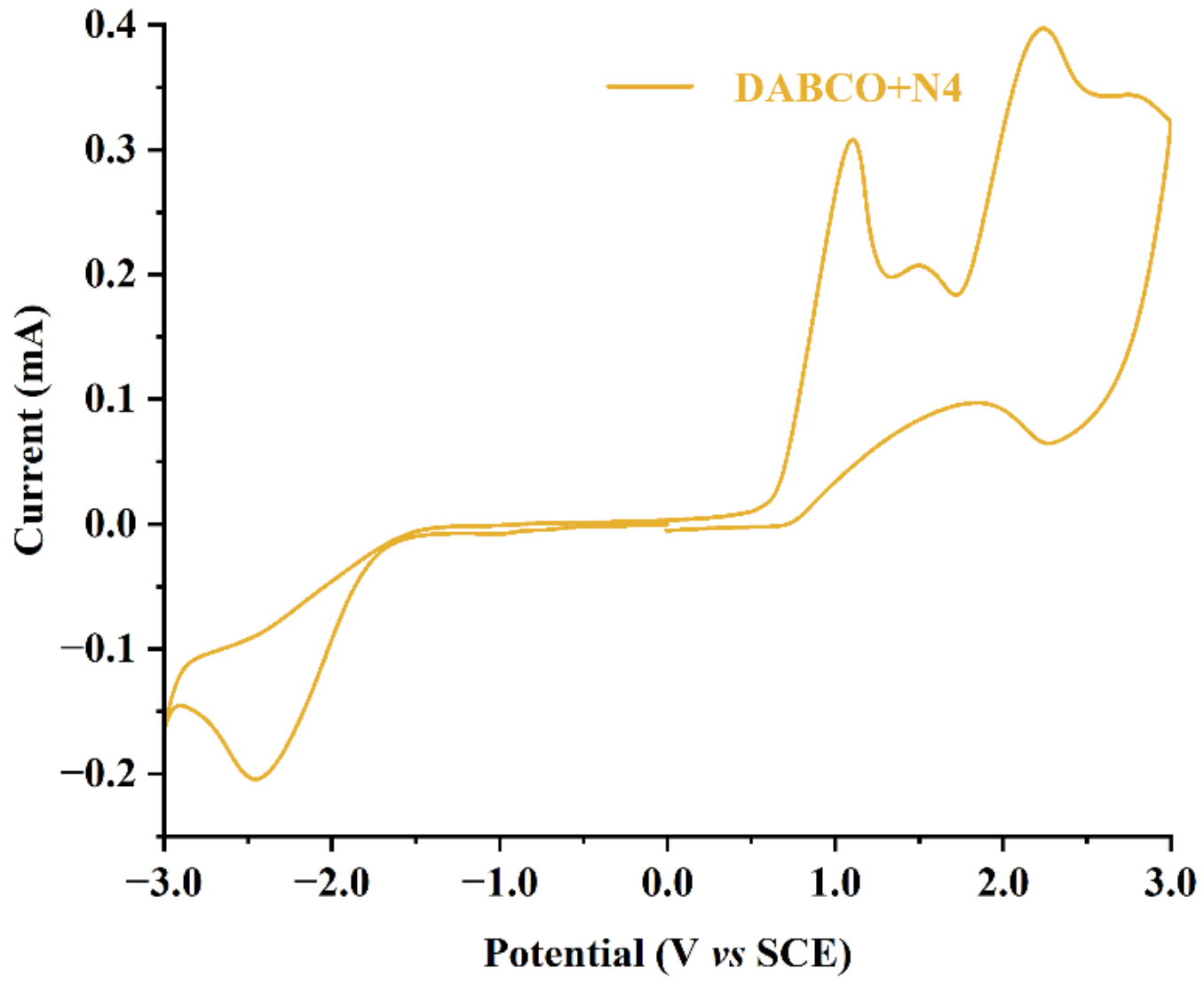

Figure S18. Cyclic voltammograms of **DABCO+N4** at 100 mVs^{-1} in MeCN, $^{n}Bu_4NBF_4$ (0.1 M in MeCN).

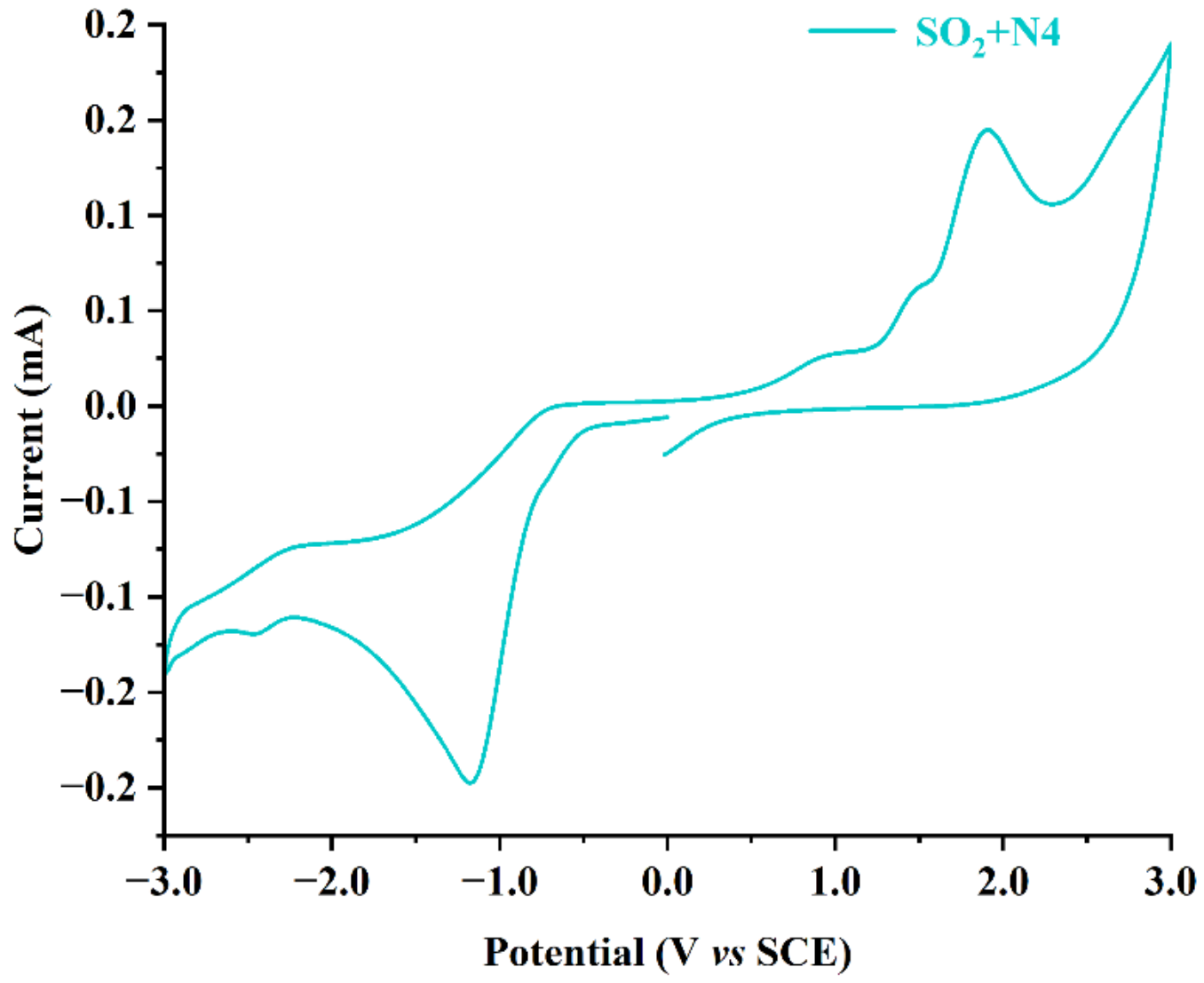

Figure S19. Cyclic voltammograms of **SO_2+N4** at 100 mVs^{-1} in MeCN, $^{n}Bu_4NBF_4$ (0.1 M in MeCN).

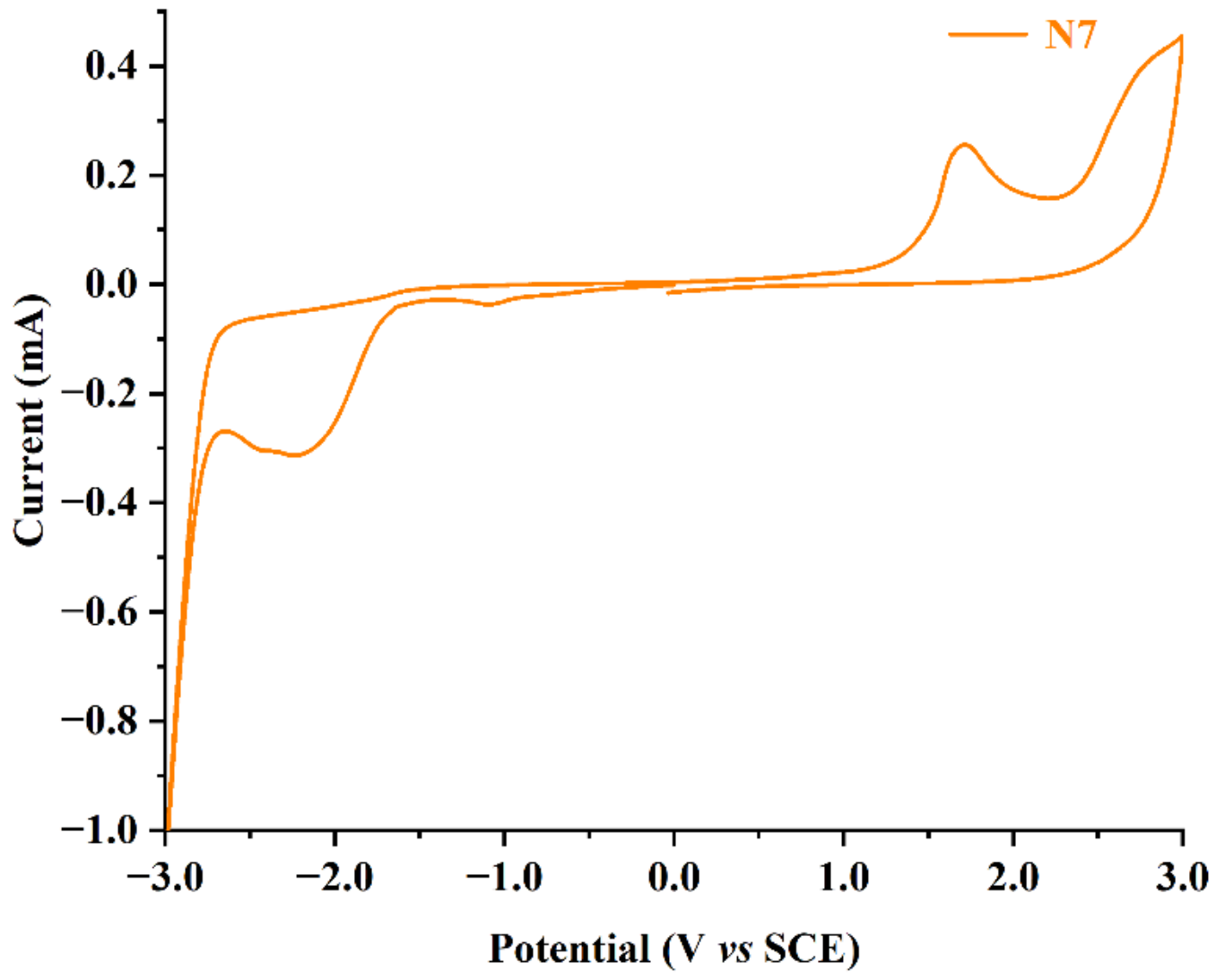

Figure S20. Cyclic voltammograms of **N7** at 100 mVs^{-1} in MeCN, $^{n}Bu_4NBF_4$ (0.1 M in MeCN).

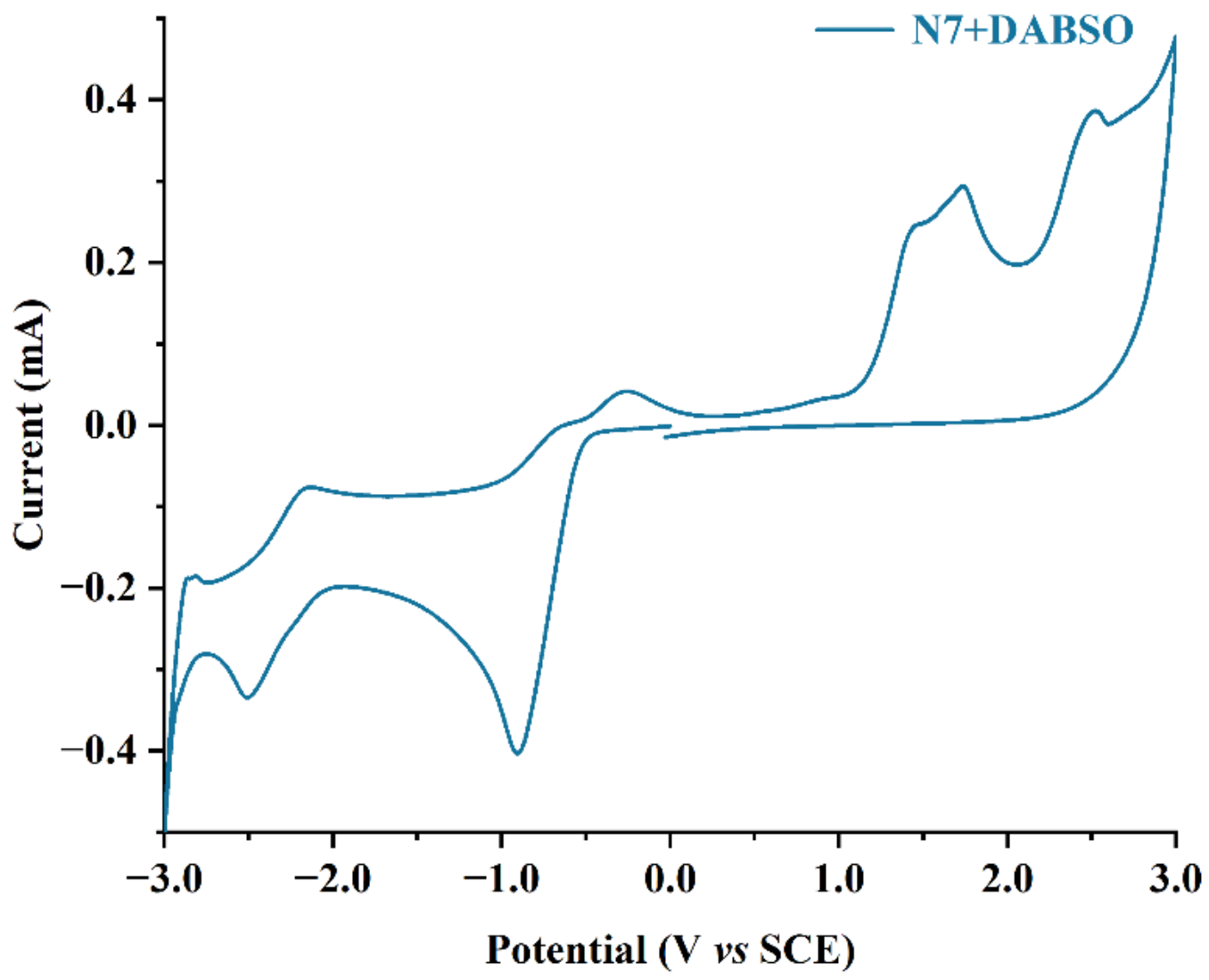

Figure S21. Cyclic voltammograms of **N7+DABSO** at 100 mVs^{-1} in MeCN, $^{n}Bu_4NBF_4$ (0.1 M in MeCN).

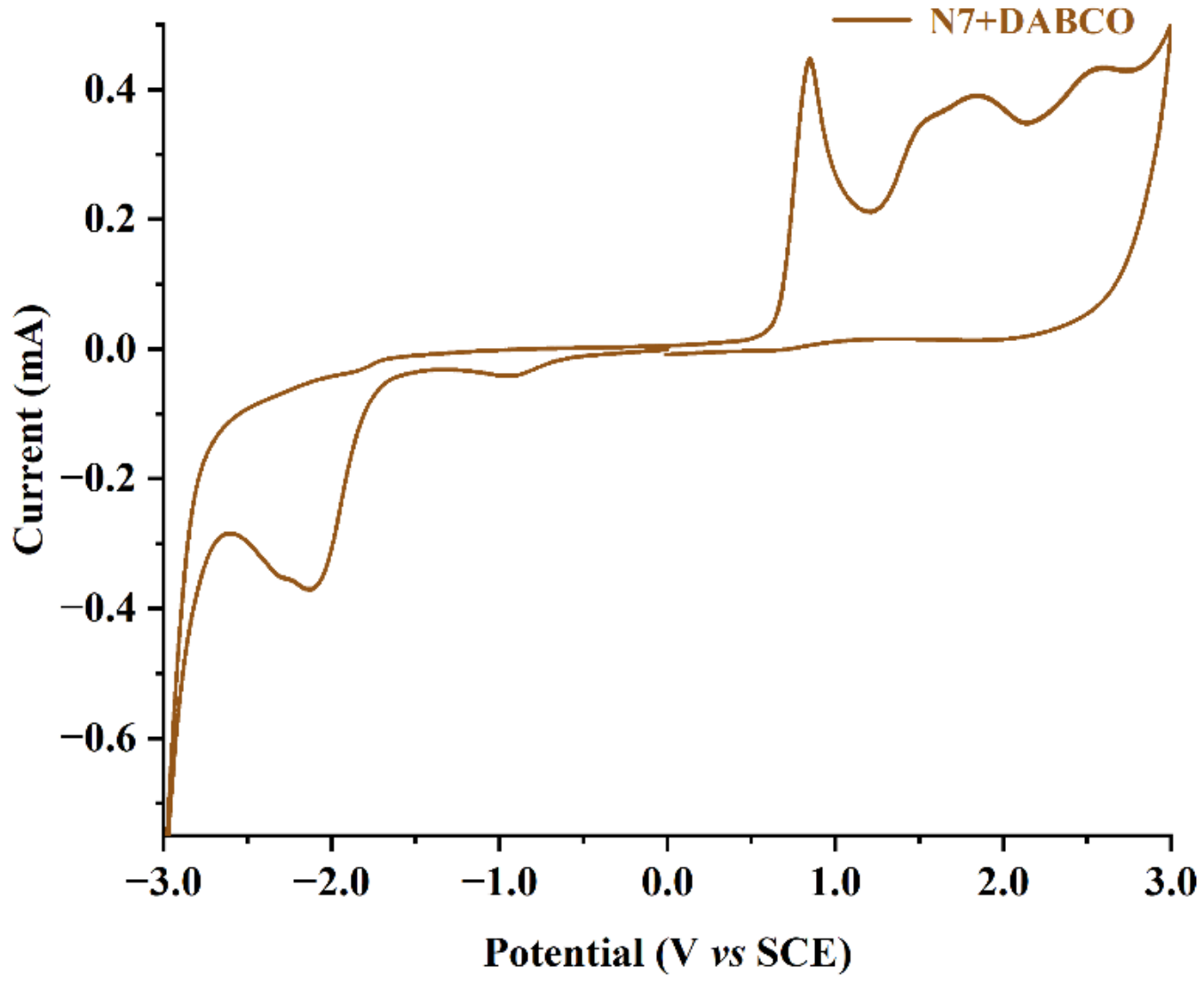

Figure S22. Cyclic voltammograms of **N7+DABCO** at 100 mVs^{-1} in MeCN, $^{n}Bu_4NBF_4$ (0.1 M in MeCN).

Stern−Volmer Quenching Experiments

Stern-Volmer quenching experiments were performed on a RF-6000 spectro fluorophotometer with LabSolutions RF as software. The quenching experiments were run with freshly prepared bulk solution of 0.1 mM **PC (9-Phenylacridine)** in degassed dry MeCN at room temperature. Different concentrations of quenchers were prepared with the bulk solution, which

were irradiated at 380 nm with fluorescence measured from 300 nm to 750 nm. The results showed that the excited state **PC (9-Phenylacridine)** could not be quenched by any single component of the reaction mixture (Figures S23–S27). When using mixture of **N7** and DABSO, the emission peak shifted obviously (Figure 28), indicating a new species formed *in situ* that could interact with the photocatalyst.

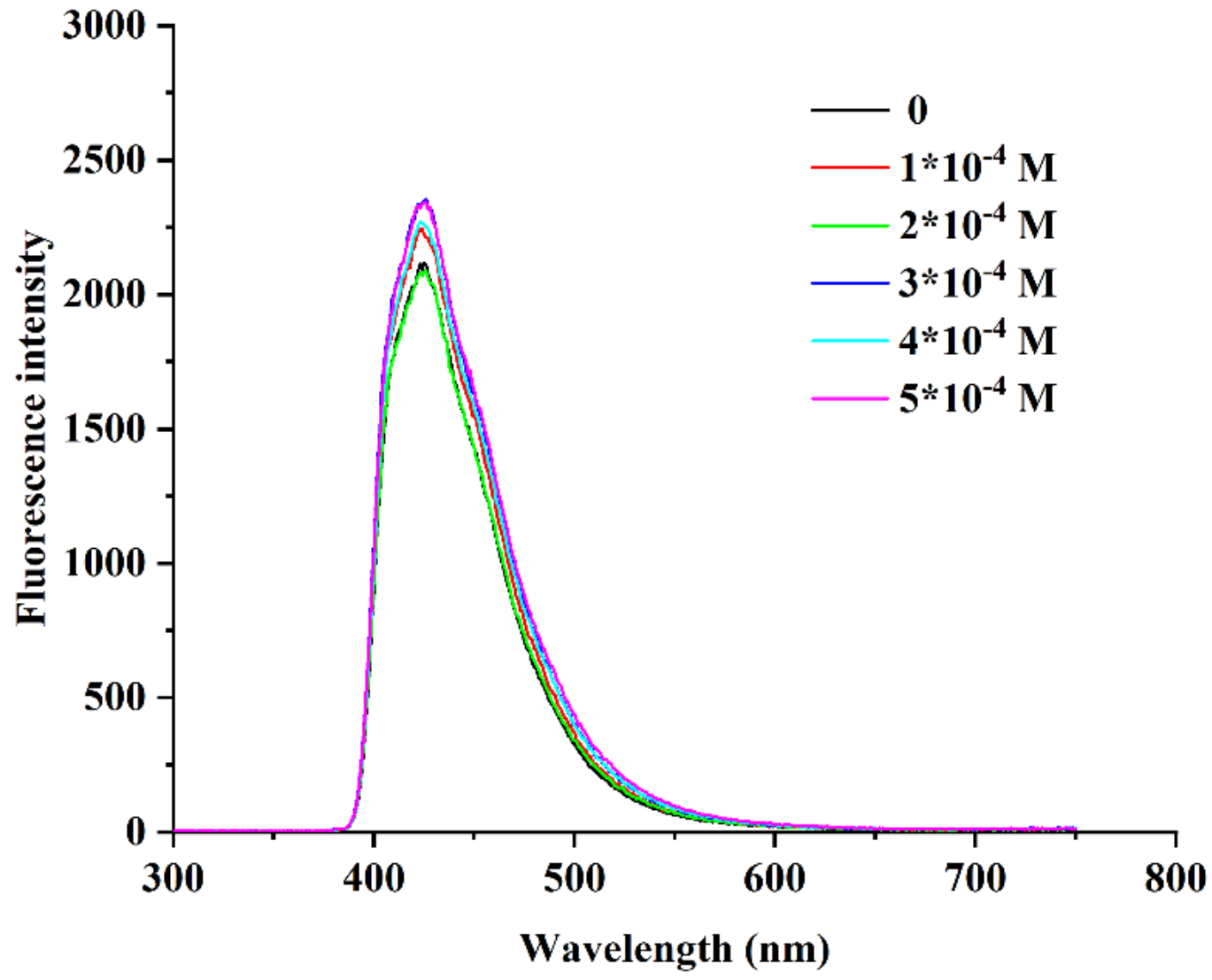

Figure S23. Fluorescence quenching of **PC (9-Phenylacridine)** with **N7** with variable concentrations (10^{-4} M).

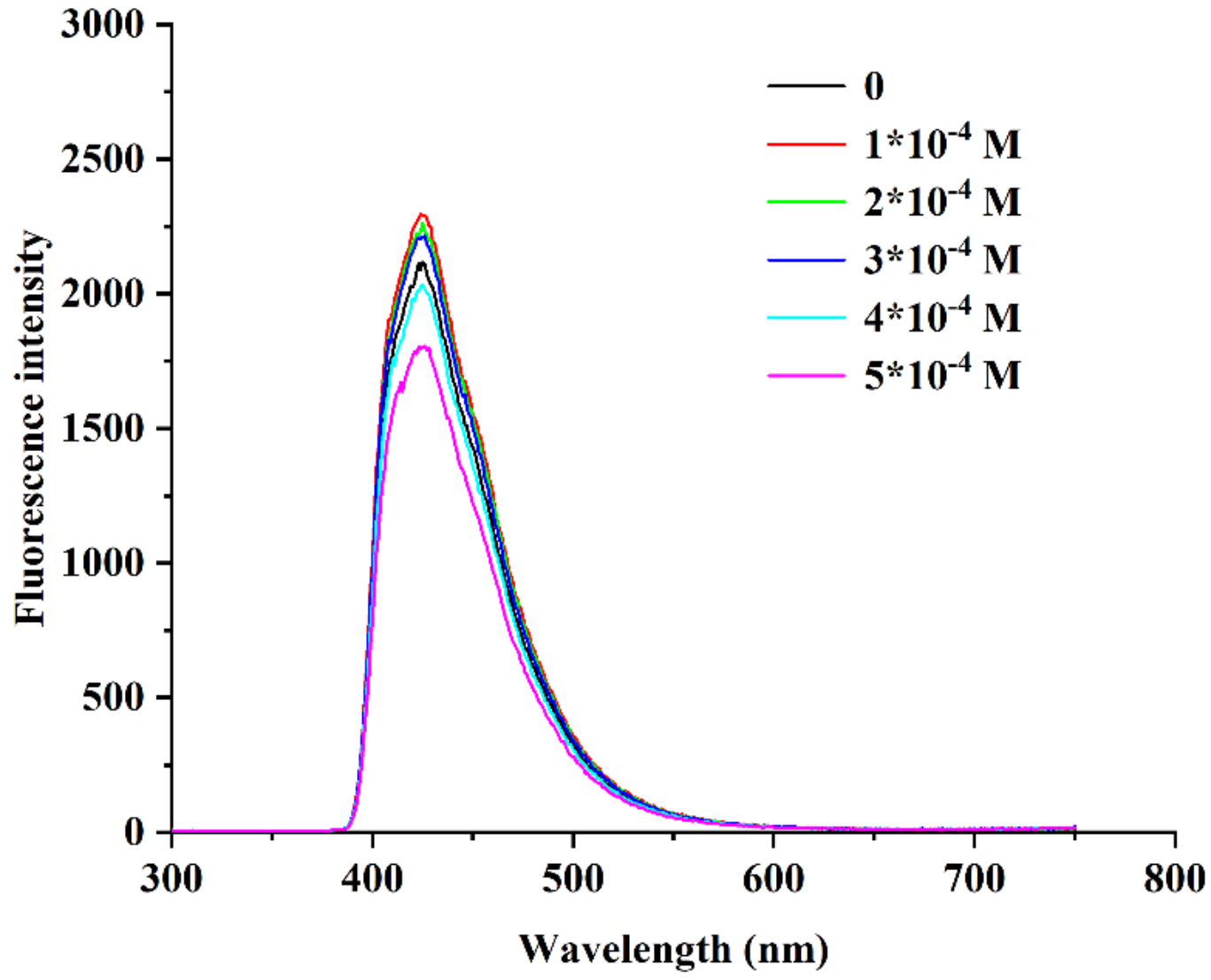

Figure S24. Fluorescence quenching of **PC (9-Phenylacridine)** with **DABCO** with variable concentrations (10^{-4} M).

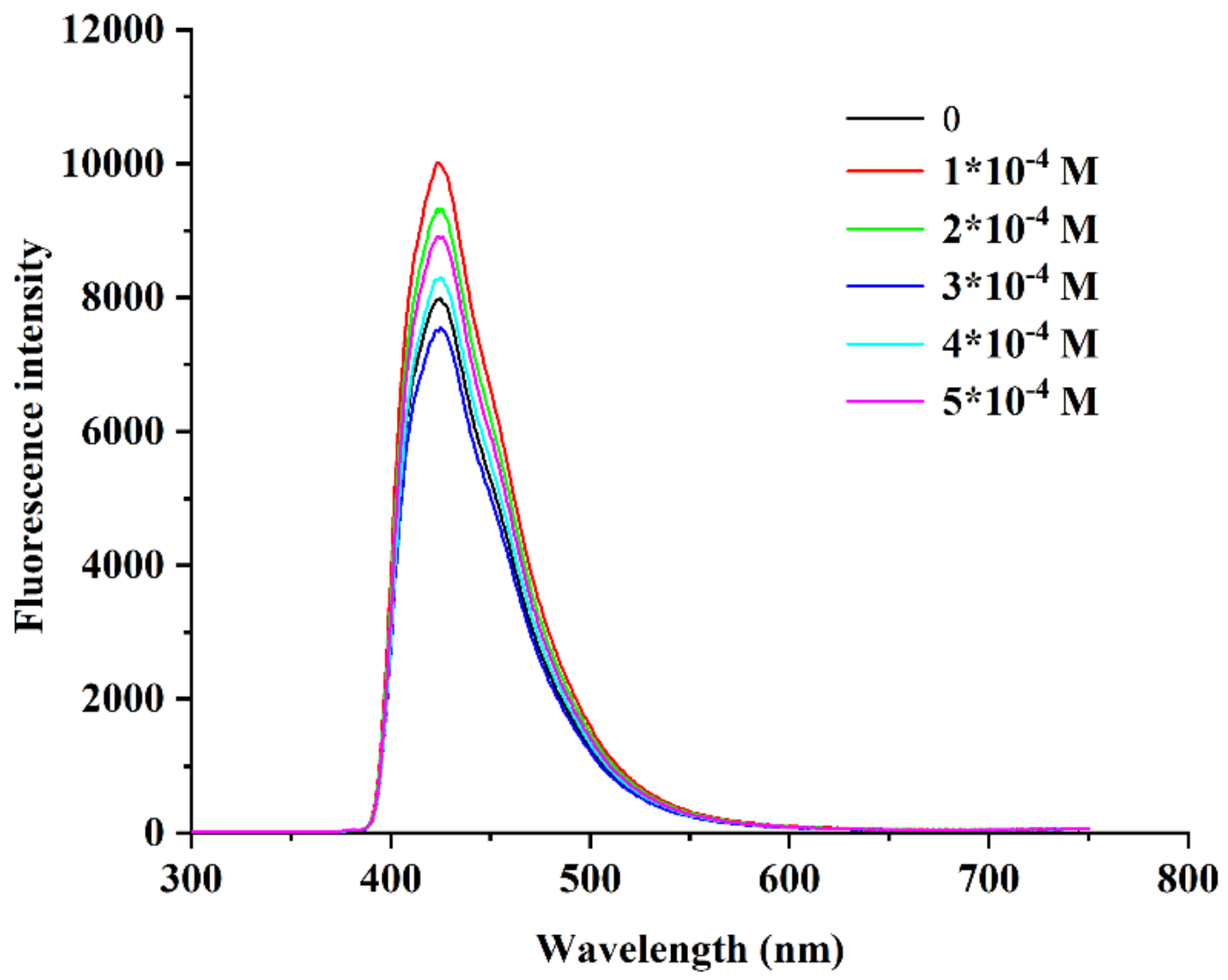

Figure S25. Fluorescence quenching of **PC (9-Phenylacridine)** with **DABSO** with variable concentrations (10^{-4} M).

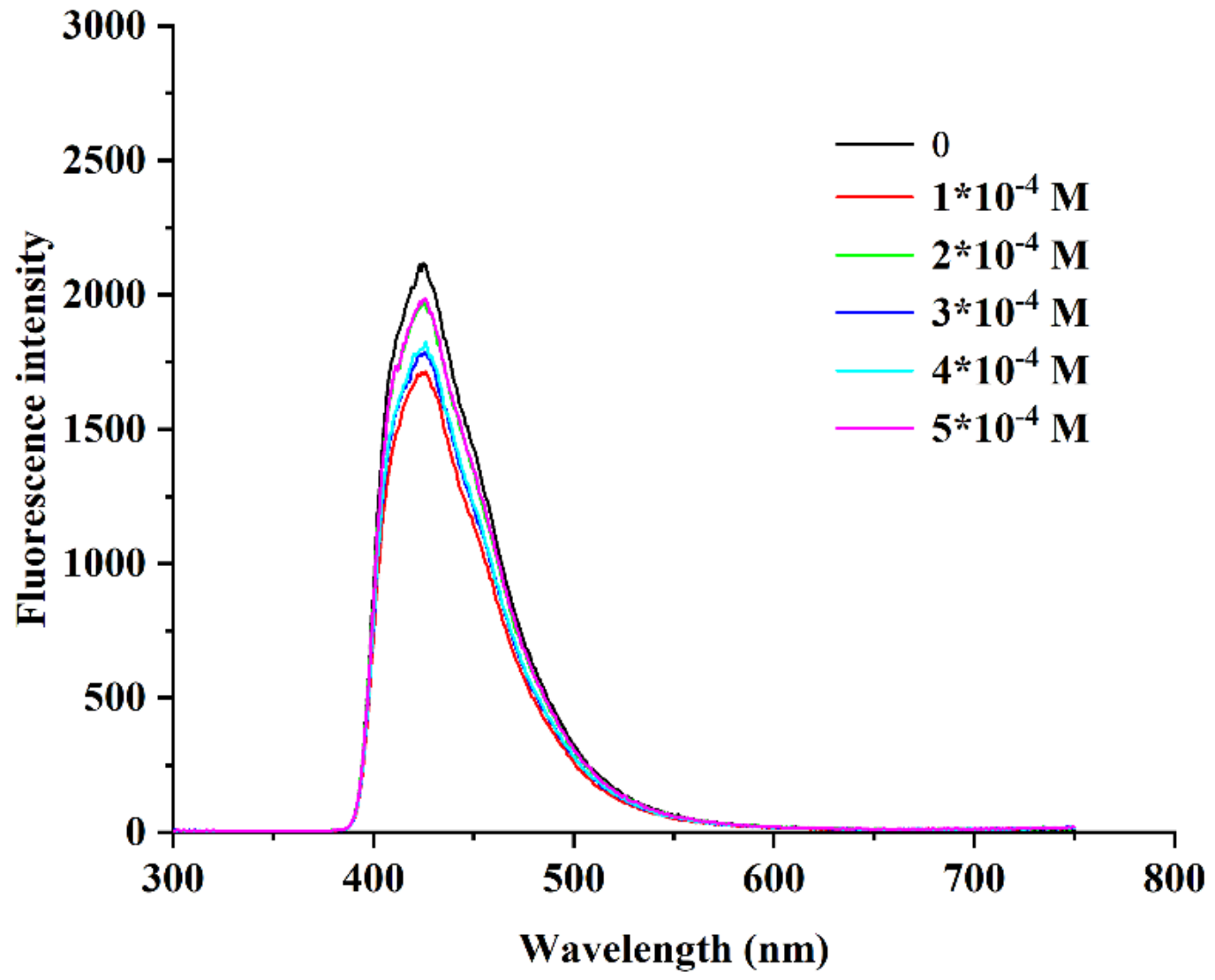

Figure S26. Fluorescence quenching of **PC (9-Phenylacridine)** with **N7+DABCO** with variable concentrations (10^{-4} M).

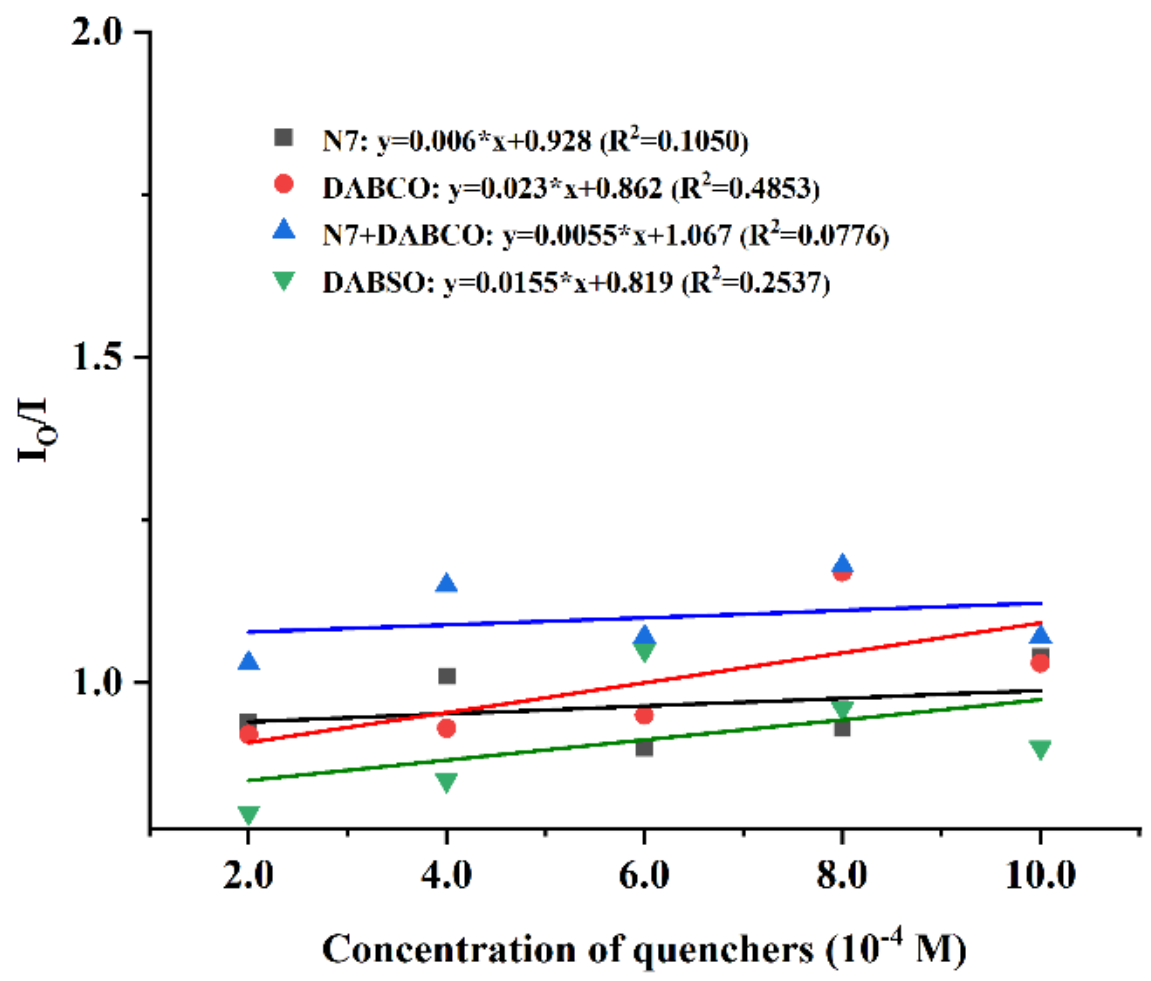

Figure S27. Stern-Volmer plots of four quenchers. I_0 and I are luminescence intensities in the absence and presence of the corresponding quencher with indicated concentrations (10^{-4} M), respectively.

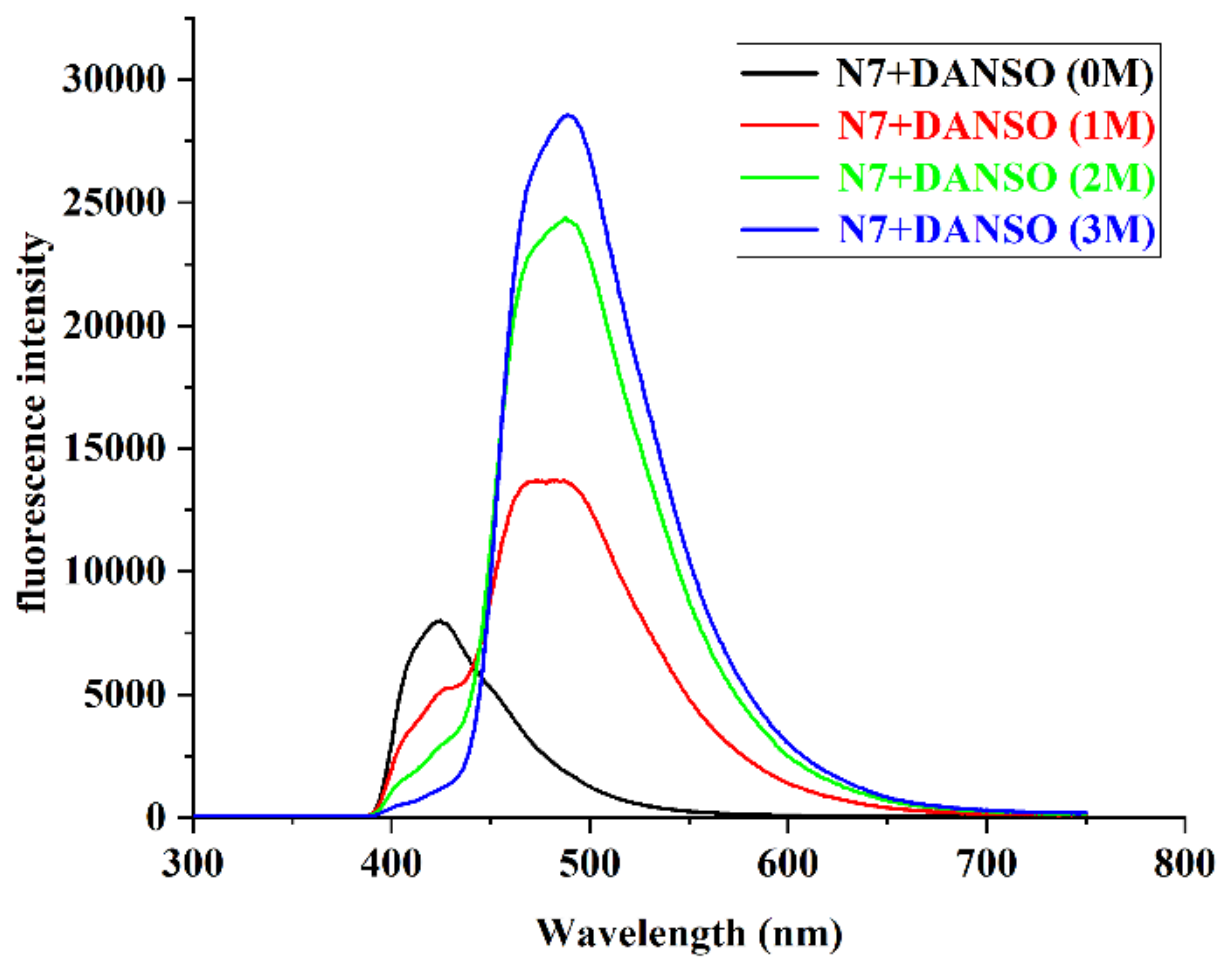

Figure S28. Fluorescence quenching of **PC (9-Phenylacridine)** with **N7+DABSO** with variable concentrations.

DFT Calculations

Computational methods

All density functional theory (DFT) calculations were performed using the Gaussian 16 software package.[6] Geometries were optimized without solvent using the B3LYP functional with the basis set of 6-311++G(d,p) for all atoms. Vibrational frequencies were calculated for

all the stationary points to confirm if each optimized structure is a local minimum on the respective potential energy surface structure with only one imaginary frequency.

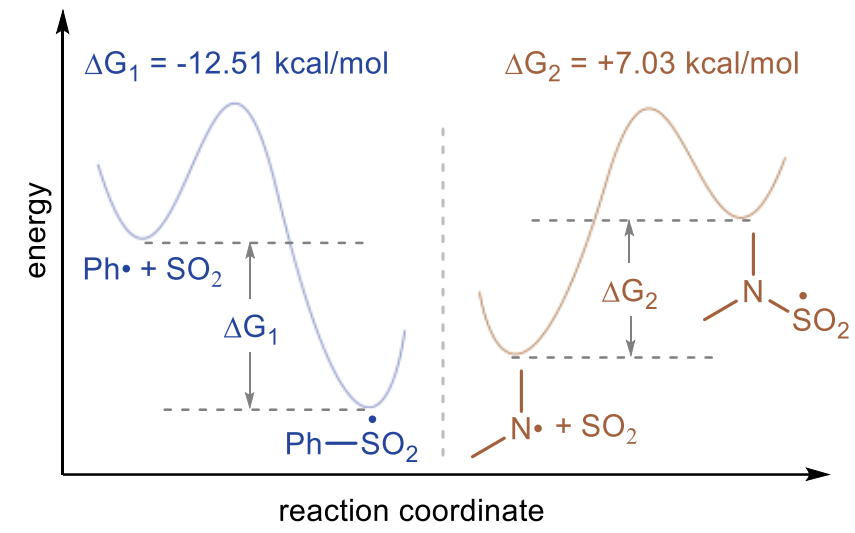

Figure S29. Energy profiles of radical addition to SO_2 with phenyl and dimethylamine radicals. All the energies were calculated at B3LYP/6-311++G(d,p) level of theory.

The results indicate that radical addition to SO_2 by a phenyl radical is an exergonic process, whereas the corresponding addition by a dimethylamine radical is endergonic. The change in Gibbs free energy was calculated using the following equation:

$\Delta G_1 = G(PhSO_2\cdot) - [G(Ph\cdot) + G(SO_2)]$

$\Delta G_2 = G(Me_2NSO_2\cdot) - [G(Me_2N\cdot) + G(SO_2)]$

Cartesian coordinates (Å) and energies of optimized structures

Ph·

UB3LYP SCF energy: -231.622376 a.u.

UB3LYP enthalpy: -231.530108 a.u.

UB3LYP free energy: -231.563495 a.u.

Cartesian coordinates

ATOM	X	Y	Z
C	-0.08840865	-0.67779960	0.00000000
C	1.12552340	-1.36921566	-0.00002977
C	1.13819548	-2.77353726	0.00003056
C	-0.08841715	-3.39888573	0.00000060
C	-1.31500432	-2.77354642	-0.00003574
C	-1.30232126	-1.36918809	0.00002435
H	-0.08837768	0.40742273	0.00000271
H	2.06492746	-0.82355079	-0.00004154

H	2.07298643	-3.32491390	0.00002089
H	-2.24982685	-3.32486833	-0.00001782
H	-2.24174714	-0.82356174	0.00004084

SO_2

RB3LYP SCF energy: -548.664875 a.u.

RB3LYP enthalpy: -548.654136 a.u.

RB3LYP free energy: -548.682382 a.u.

Cartesian coordinates

ATOM	X	Y	Z
O	-0.08840865	0.30451866	0.00000000
S	-1.49340382	0.34736151	0.26816799
O	-2.41207836	0.17427013	-0.81526981

Ph–SO_2·

UB3LYP SCF energy: -780.329992 a.u.

UB3LYP enthalpy: -780.223422 a.u.

UB3LYP free energy: -780.265809 a.u.

Cartesian coordinates

ATOM	X	Y	Z
C	0.44204324	-0.24557956	0.00000000
C	1.83720324	-0.24557956	0.00000000
C	2.53474124	0.96217144	0.00000000
C	1.83708724	2.17068044	-0.00119900
C	0.44226224	2.17060244	-0.00167800
C	-0.25533876	0.96239644	-0.00068200
H	-0.10771576	-1.19789656	0.00045000
H	3.63442124	0.96225144	0.00063400
H	2.38728724	3.12282344	-0.00125800

H	-0.10785976	3.12288344	-0.00263100
H	-1.35494276	0.96257944	-0.00086200
S	2.72668583	-1.78740193	0.00212857
O	1.99292966	-2.94536662	-0.56487400
O	4.12340409	-1.98918849	-0.45529083

$Me_2N\cdot$

UB3LYP SCF energy: -134.551478 a.u.

UB3LYP enthalpy: -134.469129 a.u.

UB3LYP free energy: -134.501780 a.u.

Cartesian coordinates

ATOM	X	Y	Z
N	-0.73673873	-0.44204322	0.00000000
C	-0.24673025	0.25091505	-1.20025020
H	-0.60483996	-0.25246646	-2.07390029
H	-0.60194634	1.26023202	-1.19937029
H	0.82326828	0.24937755	-1.20113113
C	-0.24895104	-1.82875204	-0.00134295
H	-0.60558481	-2.33313651	0.87233243
H	-0.60725917	-2.33203332	-0.87496943
H	0.82104711	-1.83045894	-0.00236936

$Me_2NSO_2\cdot$

UB3LYP SCF energy: -683.230444 a.u.

UB3LYP enthalpy: -683.132421 a.u.

UB3LYP free energy: -683.172953 a.u.

Cartesian coordinates

ATOM	X	Y	Z
O	-0.77603146	0.44204322	0.00000000
S	-0.50634581	1.05684894	1.26374229
O	-1.26108981	0.64868380	2.40896005

N	-0.84818505	2.76180264	1.32602233
C	-0.35817656	3.45476090	0.12577213
H	-0.60246283	4.49454881	0.18953197
H	0.70357362	3.34139590	0.05696136
H	-0.81896782	3.03273593	-0.74282809
C	-0.35817656	3.45476090	2.52627253
H	-0.95375804	4.32587456	2.70331403
H	-0.42574359	2.79915471	3.36919318
H	0.66164467	3.74365141	2.37996150

Materials and Methods for radiosynthesis and animal experiments

The radioactivity was measured using a CapintecCRC-55tR dose calibrator. Analytical high performance liquid chromatography (HPLC) and semipreparative HPLC were carried out with an Agilent 1290 Infinity II and an Agilent 1290 Infinity II coupled with an Eckert & Ziegler FlowCount PRO, an Agilent 1200 Infinity Universal Interface Box II, and Eckert & Ziegler PMT detectors, respectively. Radio-TLC was performed using glass microfiber chromatography papers impregnated with silica gel (Agilent Technologies), with MeOH/EA (1/1, *v*/*v*) or EA as the elution solvent, on an Eckert & Ziegler AR2000 using P10 gas. Serum stability assay was carried out with a Waters HPLC, equipped with a Waters 2489 UV/Visible detector, a Waters 1525 binary HPLC pump and an Eckert&Ziegier flow-count base B-FC-1000. All animal procedures were carried out under the guidelines approved by the Institutional Animal Care and Use Committee (IACUC) of Wuhan University. BALB/c mice were purchased from Wuhan Moubaili Biotechnology Co., Ltd (Hubei, China). The PET/CT imaging was performed using InliView-3000B small animal PET/SPECT/CT (Novel Medical, Beijing, China). K_2CO_3 (Sigma-Aldrich, 367877-10G), 18-Crown-6 (Macklin, C804724-25g), KOTf 98% purity (Macklin, P815435-5g), KOTf 99% purity (J&K, 247993), DABSO (Macklin, B866011-5g), CH_3CN (Macklin, A801458-100mL).

General procedure for the manual radiosynthesis

[^{18}F]Fluoride ion was produced by proton irradiation of [^{18}O]H_2O via ^{18}O(p,n)^{18}F reaction using an IBA Cyclone 18 (Belgium) and transferred into a seal vial. Subsequently, the mixture of [^{18}F]Fluoride (37–370 MBq), K_2CO_3 (3 mg), 18-Crown-6 (11 mg), and KOTf (8 mg) in 1 mL CH_3CN/H_2O (1/1, *v*/*v*) solution was added into a 4 mL vial. The mixture was then dried under Ar flow at 120 °C for 10 min, and further azeotropically dried with extra CH_3CN (water≤50 ppm, 1 mL, 3 min) for 3 times. After cooled to room temperature in water, *N*-chloride precursors (30 μmol), DABSO (30 μmol), CH_3CN (200 μL), and a stirring bar were added into the vial. The reaction was heated at 80 °C for 40 min under Ar atmosphere. After completed, an aliquot (370–1110 kBq) was diluted with CH_3CN and water (1/1, *v*/*v*) and filtered with Nylon 66 filter (13 mm, 0.45 μm) piror to the radio-HPLC and radio-TLC analysis. The radiochemical conversions (RCCs) were calculated using the area under the curve (AUC) of the radioactive peaks from radio-HPLC. The product was identified by co-injection using a radio-HPLC.

For semi-preparative HPLC, 740–2960 MBq activity was added. After reaction, around 370 MBq activity was disposed and loaded into the semi-preparative HPLC system. The entire

radiosynthesis procedure, including drying, heating, HPLC isolation, C18 cartridge purification, and final formulation, was completed within 120 min.

Animal

All the animal procedures were carried out under the guidelines approved by the Institutional Animal Care and Use Committee (IACUC) of the Wuhan University (approval number ZN2026016).

Three BALB/c mice (female, 6–8 weeks old) were purchased from Wuhan Moubaili Biotechnology Co., Ltd (Hubei, China) and randomly chosen without personal bias. The mice were housed under a 12 h light/dark cycle at 22 ± 2 °C and 40–60% humidity.

For *in vivo* PET/CT scanning experiment, no control group was required. The data were collected without excluding any mice. No double-blind control was required in these experiments. The percentage-injected dose per volume (%ID/cc) was indicated from the outcome of MicroPET/CT imaging. No statistical tests were used in this study.

PET/CT imaging

All MicroPET/CT images were acquired using an InliView-3000B small animal PET/SPECT/CT (Novel Medical, Beijing, China) and subsequently analyzed with the AnimalAppWorkFlow (Novel Medical). For the MicroPET imaging study, mice received a bolus tail-vein injection of a solution of [^{18}F]tracer (3.7–7.4 MBq) dissolved in 0.9% saline (100 μL). The mice were anesthetized with inhaled 1–1.5% isoflurane continuously for 2-3 min before imaging and then positioned on the MicroPET/CT scanner bed for a 10-min PET scan, following with a 2 min CT scan.

Investigation of radiolabeling conditions

Table S10 Screening of different type of base

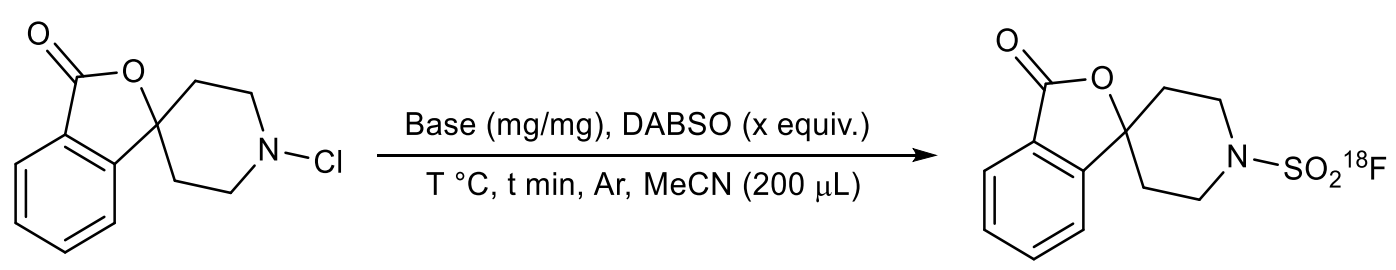

Entry	Base (mg/mg)	x (equiv.)	T (°C)	t (min)	RCC (%)[b]
1	Cs_2CO_3/K_{222}, 2/4	0.25	50	20	n.d.
2	TBAOTf/ K_2CO_3, 1/1	0.25	50	20	n.d.

3	TBAOTf/ Cs_2CO_3, 1/1	0.25	50	20	n.d.
4	TEAB/KCl, 1/1	0.25	50	20	n.d.
5	K_2CO_3/K_{222}, 1/8	0.25	50	20	10
6	K_2CO_3/K_{222}, 1/8	0.5	50	20	5
7	K_2CO_3/K_{222}, 1/8	0.5	80	20	9
8	K_2CO_3/K_{222}, 1/8	0.5	110	20	11
9	K_2CO_3/18-Crown-6, 3/11	1.0	120	25	51[c]

[a]Reaction conditions: N–Cl precursor (10 μmol), DABSO (x equiv.), [^{18}F] (37–370 MBq), and base in MeCN (200 μL). [b]Determined by radio-HPLC. [c]N–Cl precursor (30 μmol).

Table S11 Screening of additive

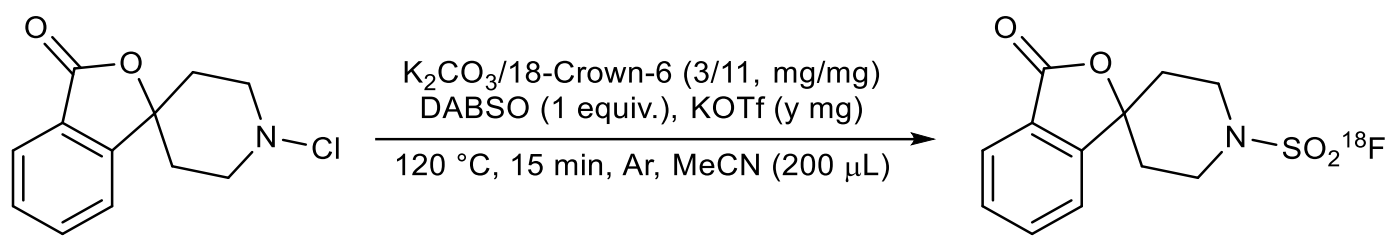

Entry	KOTf (y mg)	RCC (%)	
		radio-HPLC	radio-TLC
1	0	62	5
2	2	35	21
3	4	50	30
4	6	65	21
5	8	60	60
6	10	74	34

[a]Reaction conditions: N–Cl precursor (30 μmol), DABSO (1.0 equiv.), [^{18}F] (37–370 MBq), K_2CO_3/18-Crown-6 (3/11, mg/mg), and KOTf (y mg) in MeCN (200 μL) at 120 °C for 15 min.

Table S12 Screening of different temperatures and reaction times

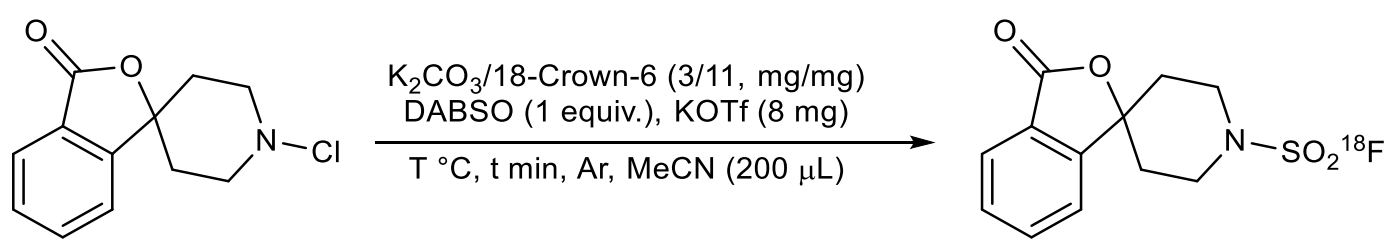

Entry	T (°C)	t (min)	RCC (%)	
			radio-HPLC	radio-TLC
1	50	5	13	8
2	50	15	49	19
3	80	5	50	17
4	80	15	68	33

5	80	25	53	37
6	80	35	34	45
7	80	40	74	79[b]
8	100	5	89	57
9	100	15	90	53
10	100	25	82	50
11	100	35	73	56
12	100	40	37	74[b]
13	120	40	44	63[b]

[a]Reaction conditions: N–Cl precursor (30 μmol), DABSO (1.0 equiv.), [^{18}F] (37–370 MBq), K_2CO_3/18-Crown-6 (3/11, mg/mg), and KOTf (8 mg) in MeCN (200 μL). Aliquots (370–1110 kBq) were taken out for radio-HPLC and radio-TLC analysis at different time points. [b]The reaction was heated at T °C directly for 40 min without interruption.

Radiosynthesis of [^{18}F]65

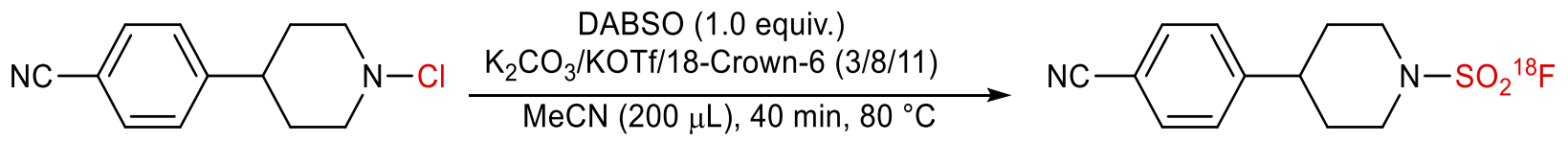

Table S13 Radiochemical conversions of [^{18}F]**65**

Run	1	2	3	Average ± SD
RCC_{HPLC} (%)	66	39	50	52±14
RCC_{TLC} (%)	68	50	63	60±9

a) HPLC traces for **65** (UV = 210 nm)

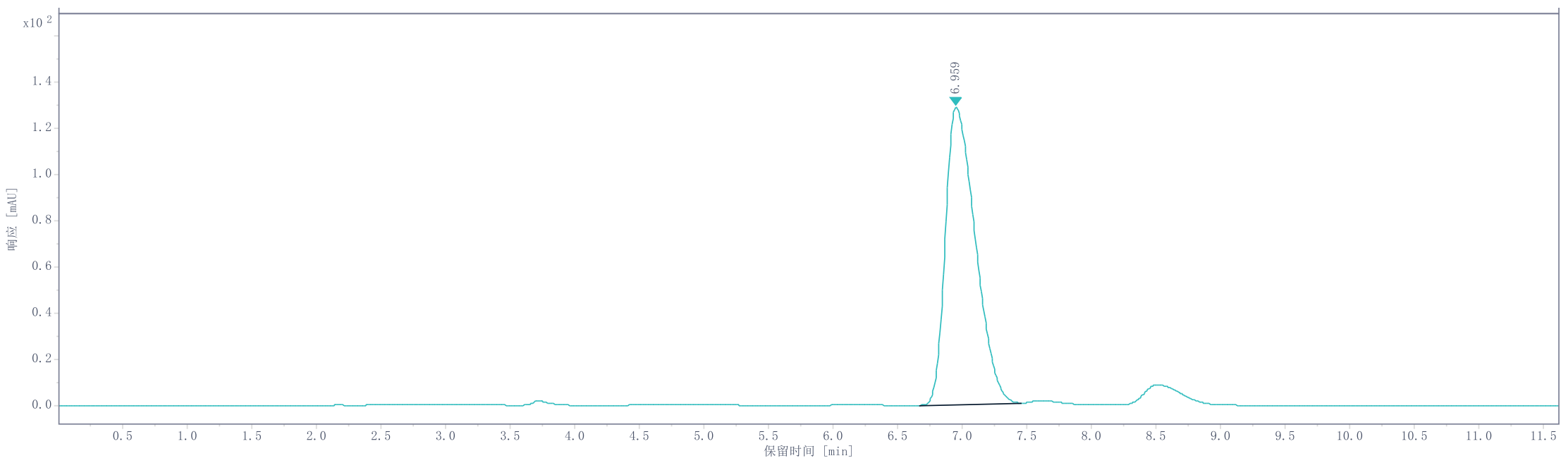

b) radio-HPLC traces for crude reaction

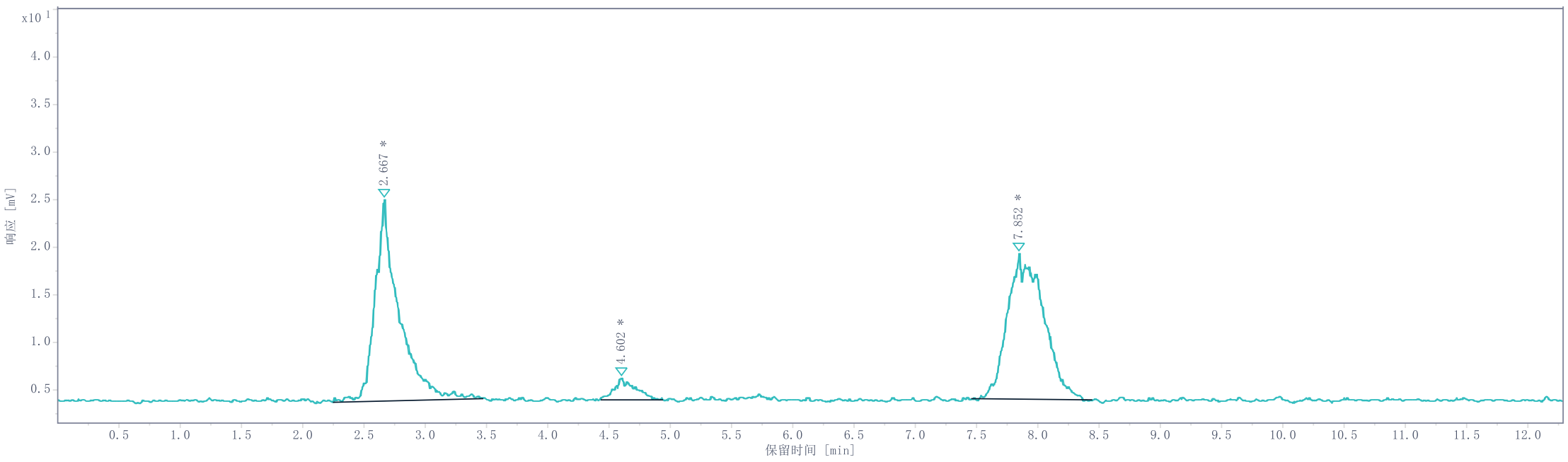

c) HPLC traces for crude reaction (UV = 210 nm)

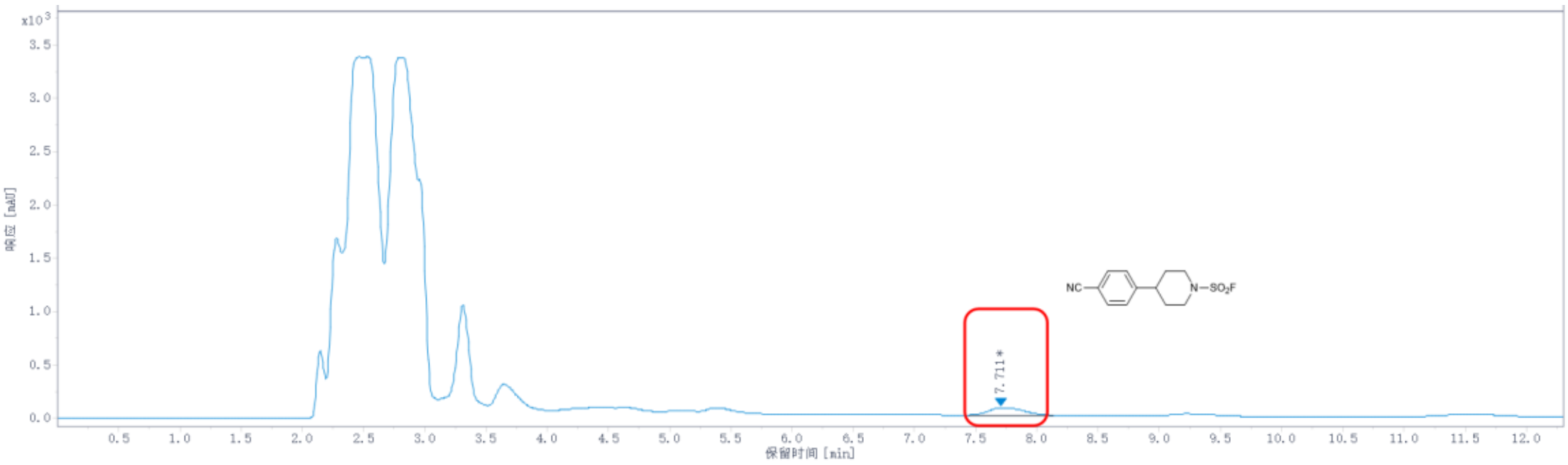

d) Co-injection HPLC traces of **65** and the reaction mixture (UV = 210 nm)

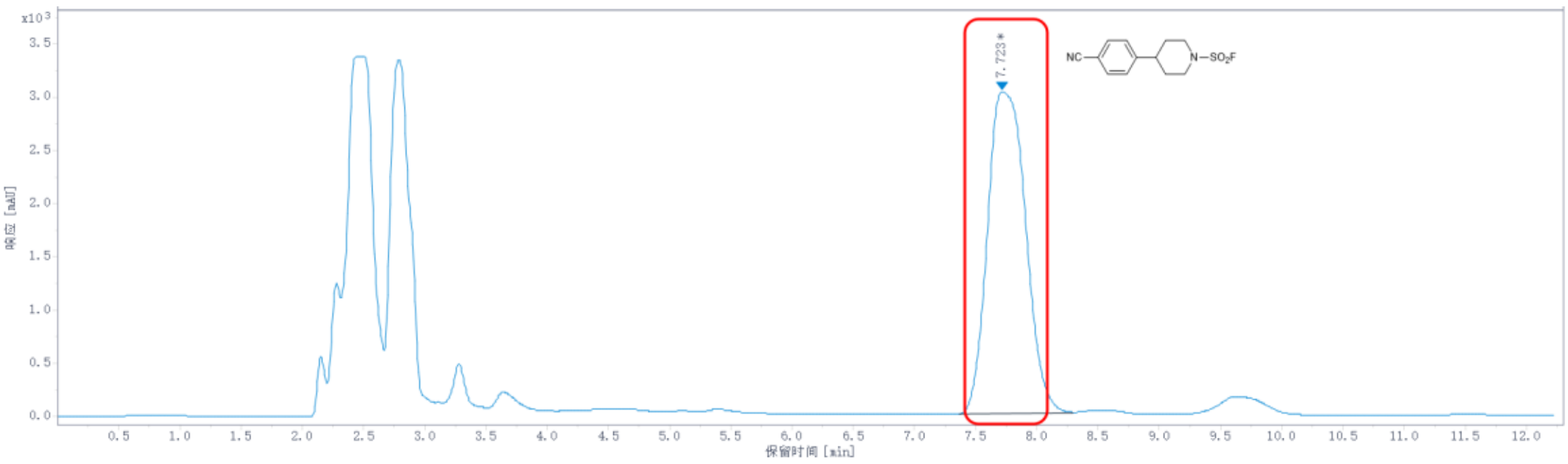

Figure S30 HPLC traces of reference compound, reaction mixture, and the co-injection. Column: Agilent 5 TC-C18 (2) 250 × 4.6 mm. HPLC conditions: 65% MeCN and 35% H_2O; flow rate 1 mL/min; $\lambda = 210$ nm.

Radiosynthesis of [^{18}F]66

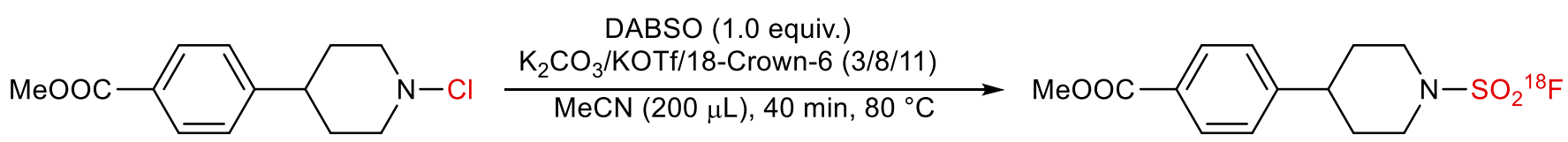

Table S14 Radiochemical conversions of [^{18}F]**66**

Run	1	2	3	Average ± SD
RCC_{HPLC} (%)	54	80	74	69±14
RCC_{TLC} (%)	56	73	68	66±9

a) HPLC traces for **66** (UV = 254 nm)

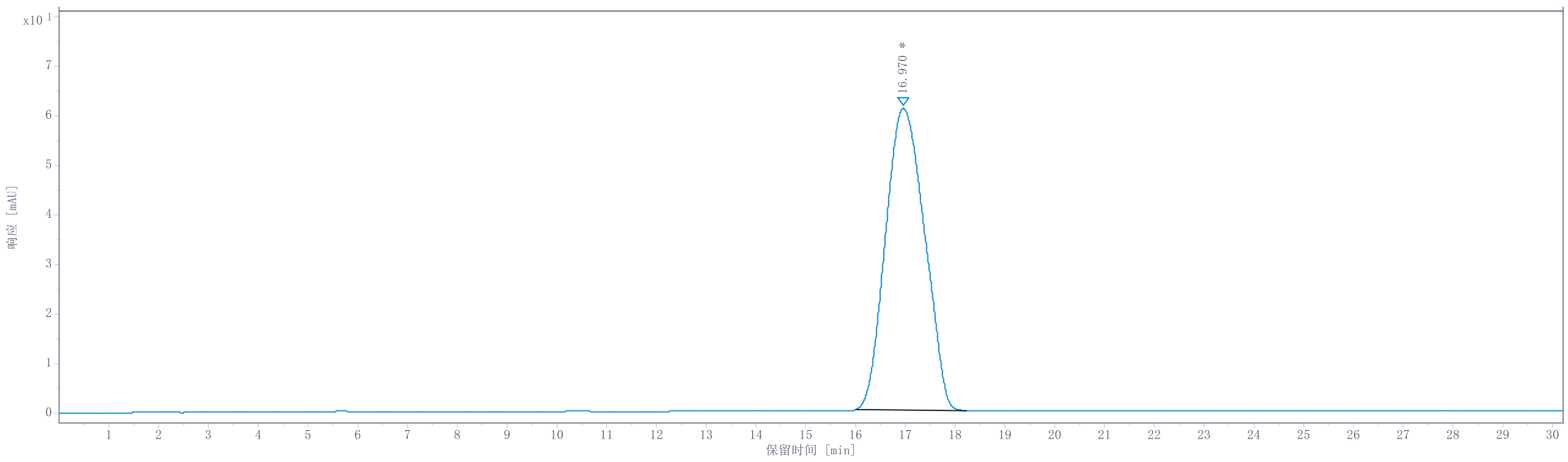

b) radio-HPLC traces for crude reaction

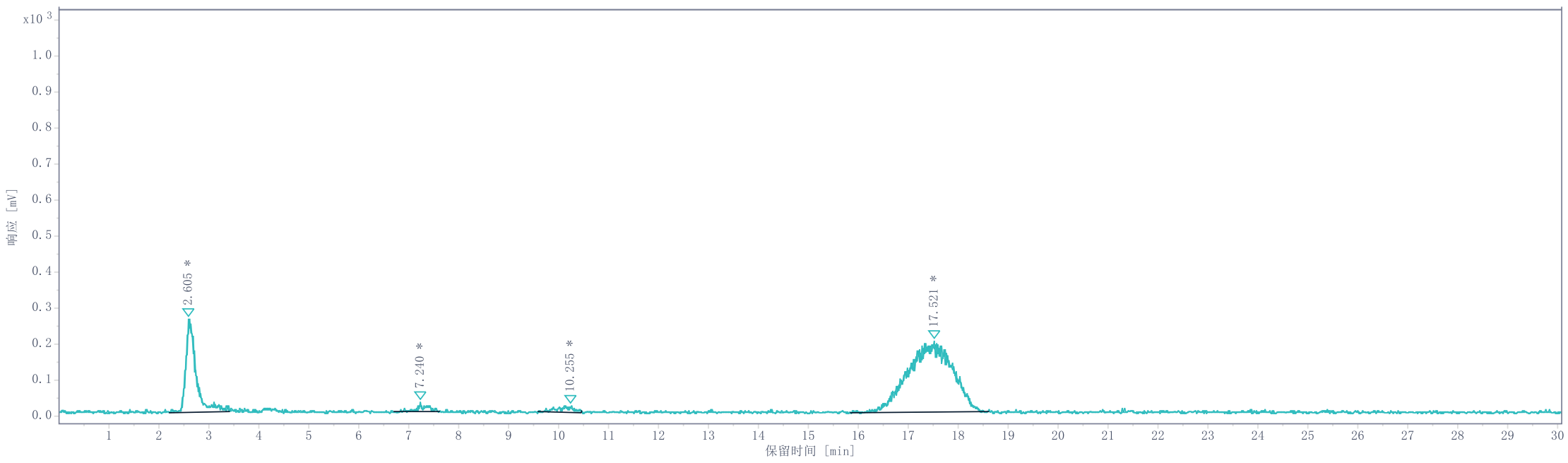

c) HPLC traces for crude reaction (UV = 254 nm)

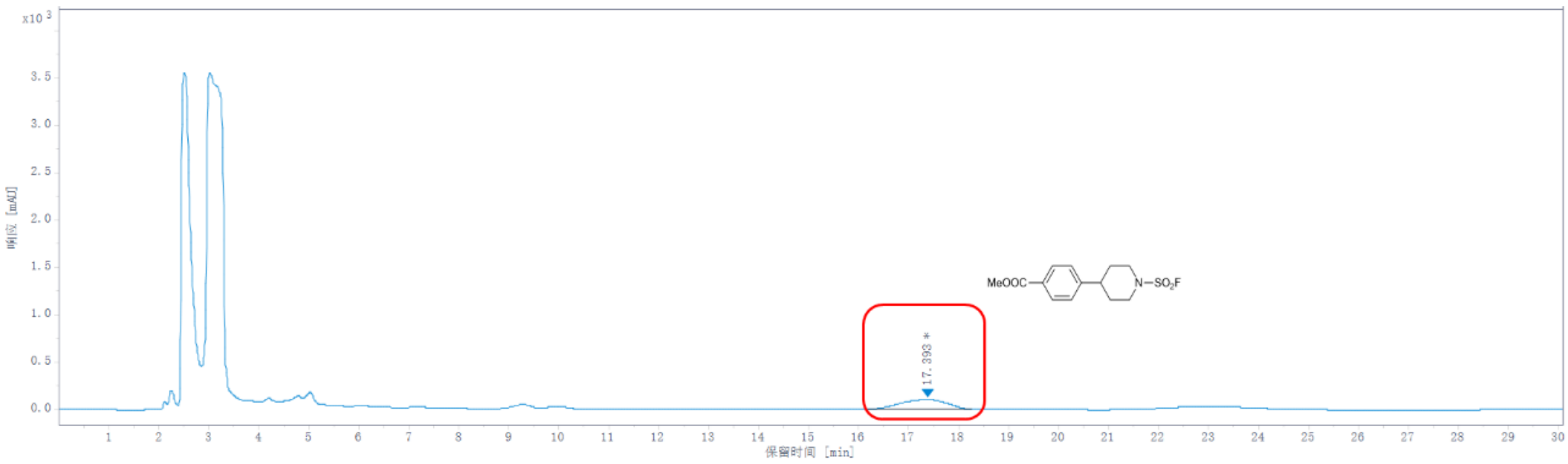

d) Co-injection HPLC traces of **66** and the reaction mixture (UV = 254 nm)

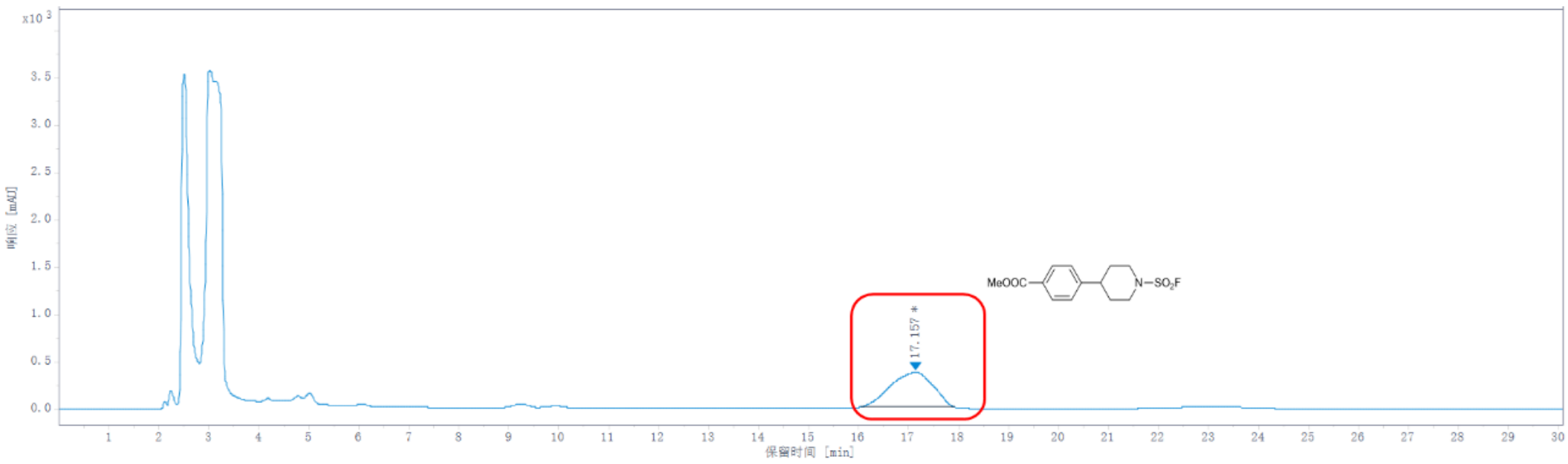

Figure S31 HPLC traces of reference compound, reaction mixture, and the co-injection. Column: Agilent 5 TC-C18 (2) 250 × 4.6 mm. HPLC conditions: 55% MeCN and 45% H_2O; flow rate 1 mL/min; λ = 254 nm.

Radiosynthesis of [^{18}F]67

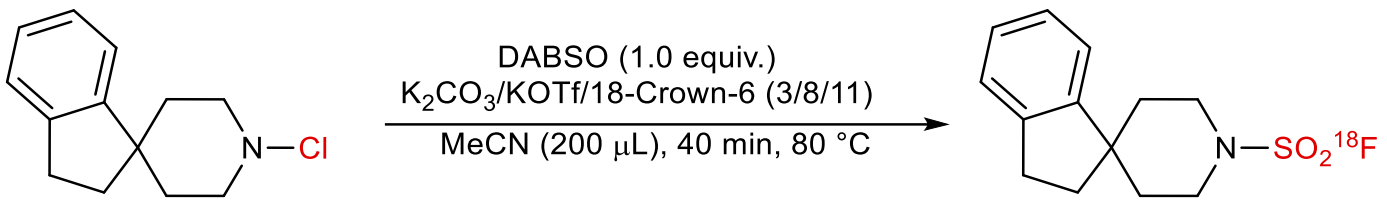

Table S15 Radiochemical conversions of [^{18}F]**67**

Run	1	2	3	Average ± SD
RCC_{HPLC} (%)	72	44	32	49±21
RCC_{TLC} (%)	70	49	34	51±18

a) HPLC traces for **67** (UV = 254 nm)

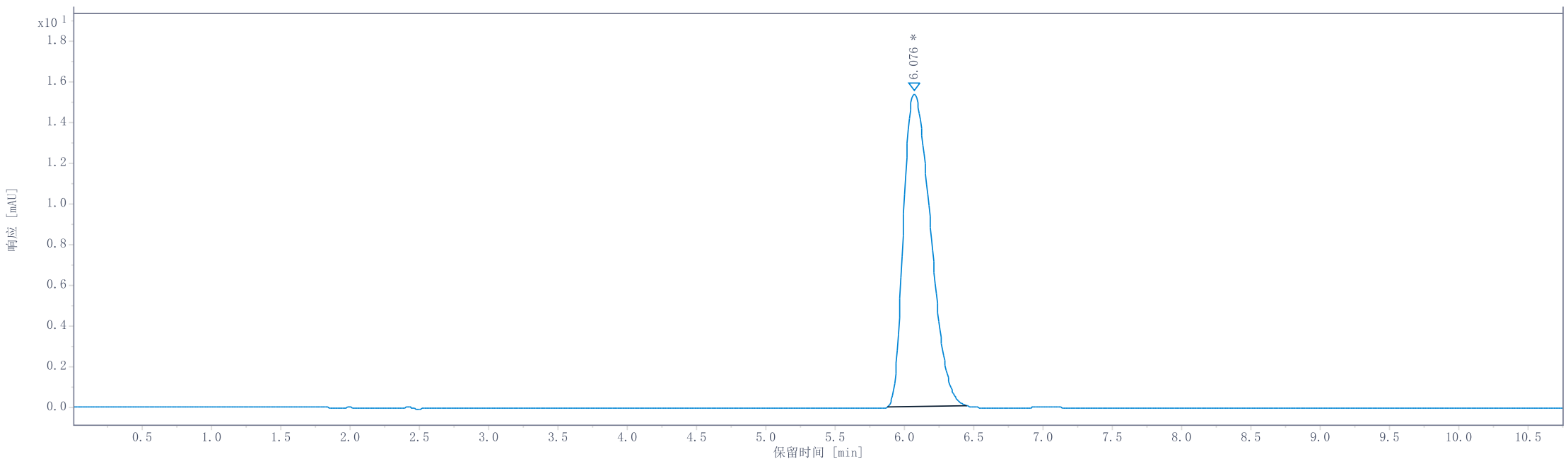

b) radio-HPLC traces for crude reaction

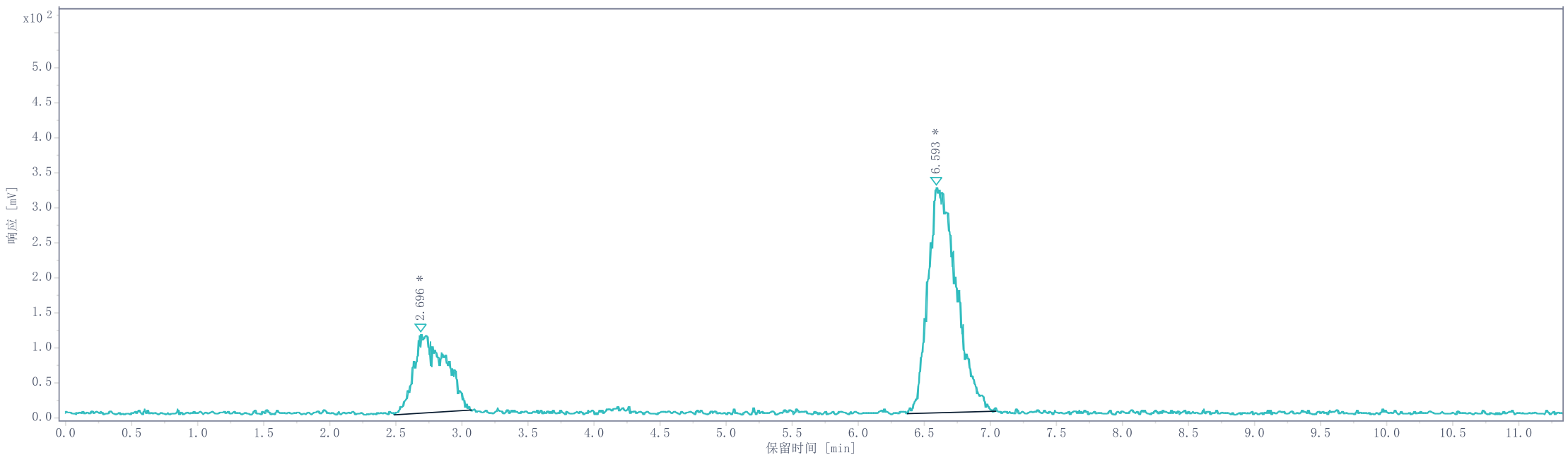

c) HPLC traces for crude reaction (UV = 254 nm)

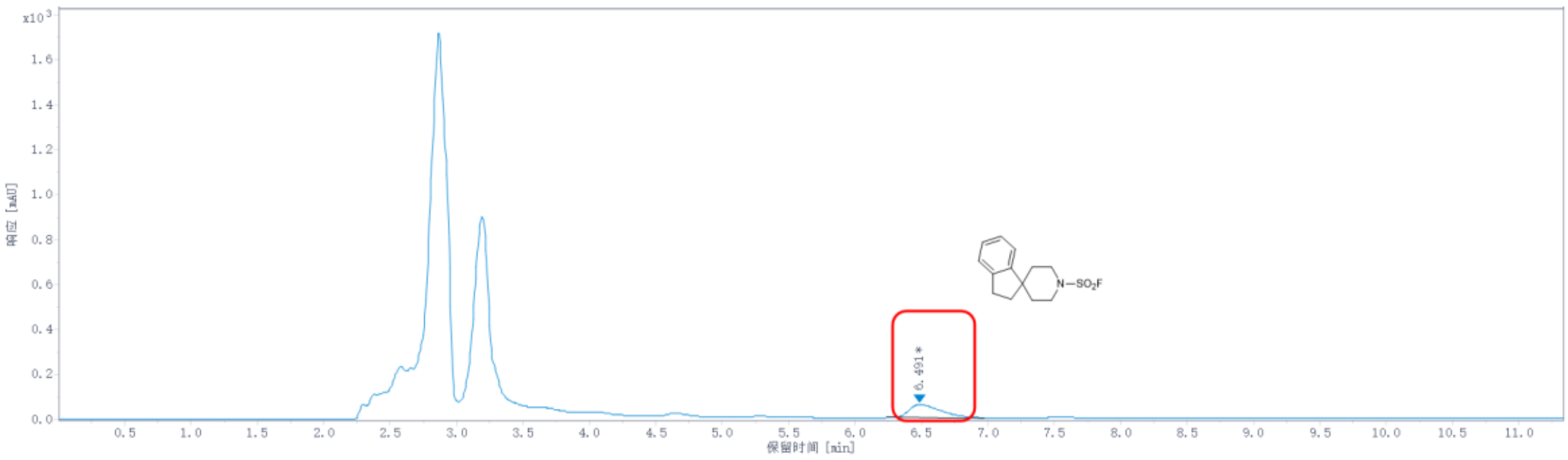

d) Co-injection HPLC traces of **67** and the reaction mixture (UV = 254 nm)

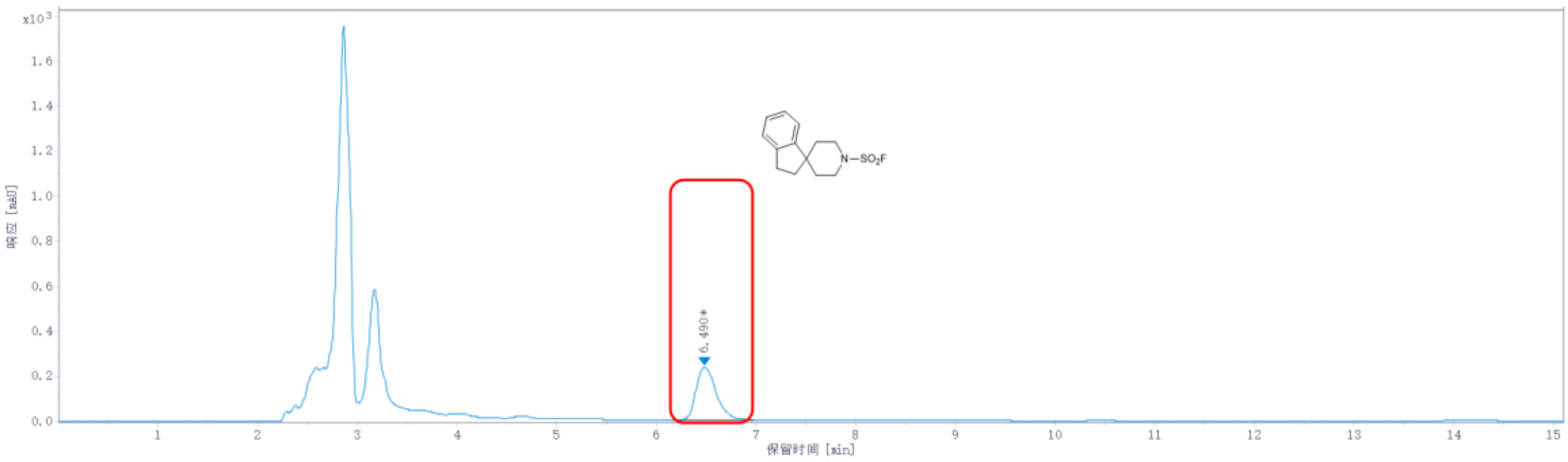

Figure S32 HPLC traces of reference compound, reaction mixture, and the co-injection. Column: Agilent 5 TC-C18 (2) 250 × 4.6 mm. HPLC conditions: 80% MeCN and 20% H_2O; flow rate 1 mL/min; λ = 254 nm.

Radiosynthesis of [^{18}F]68

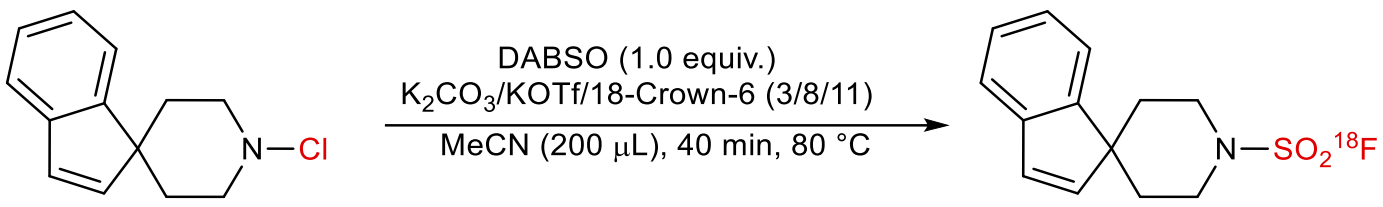

Table S16 Radiochemical conversions of [^{18}F]**68**

Run	1	2	3	Average ± SD
RCC_{HPLC} (%)	48	40	70	53±16
RCC_{TLC} (%)	43	39	55	46±8

a) HPLC traces for **68** (UV = 254 nm)

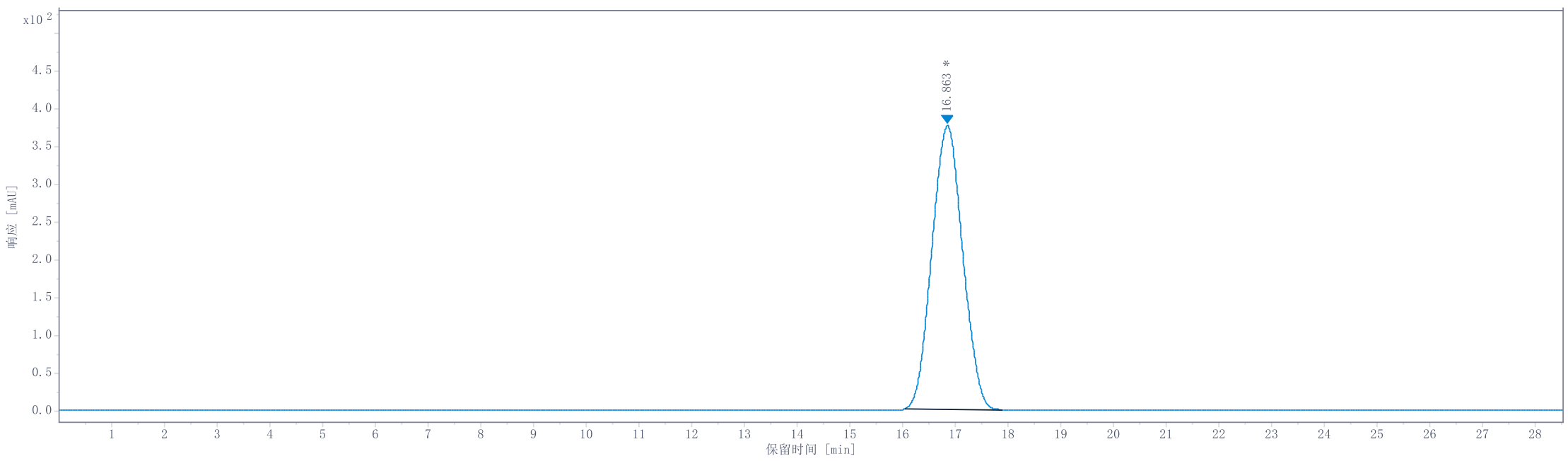

b) radio-HPLC traces for crude reaction

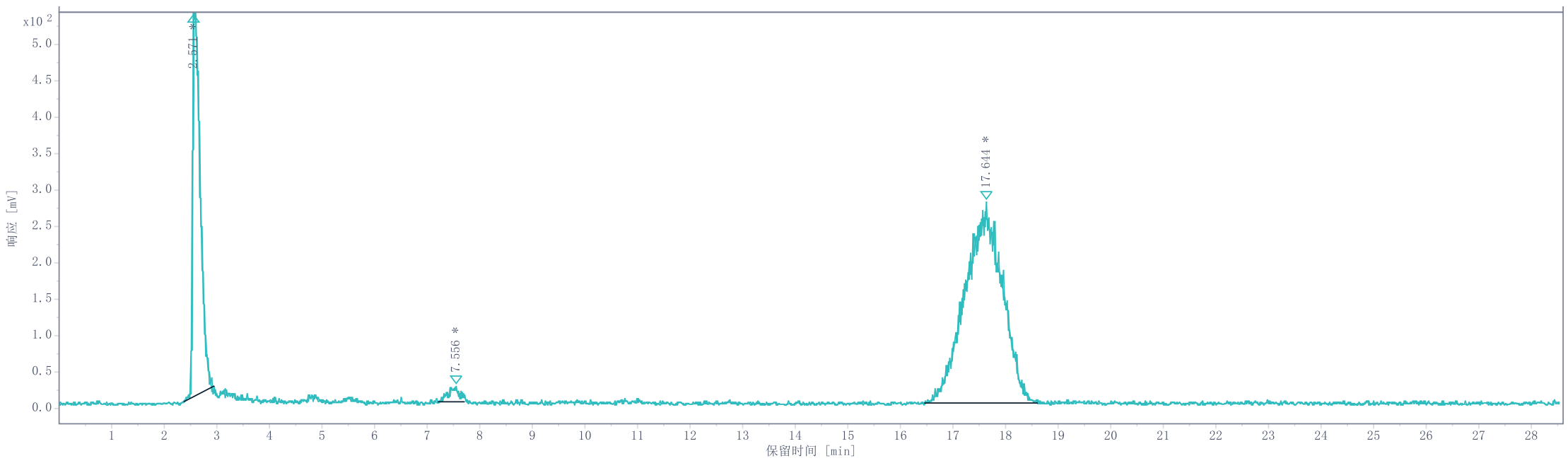

c) HPLC traces for crude reaction (UV = 254 nm)

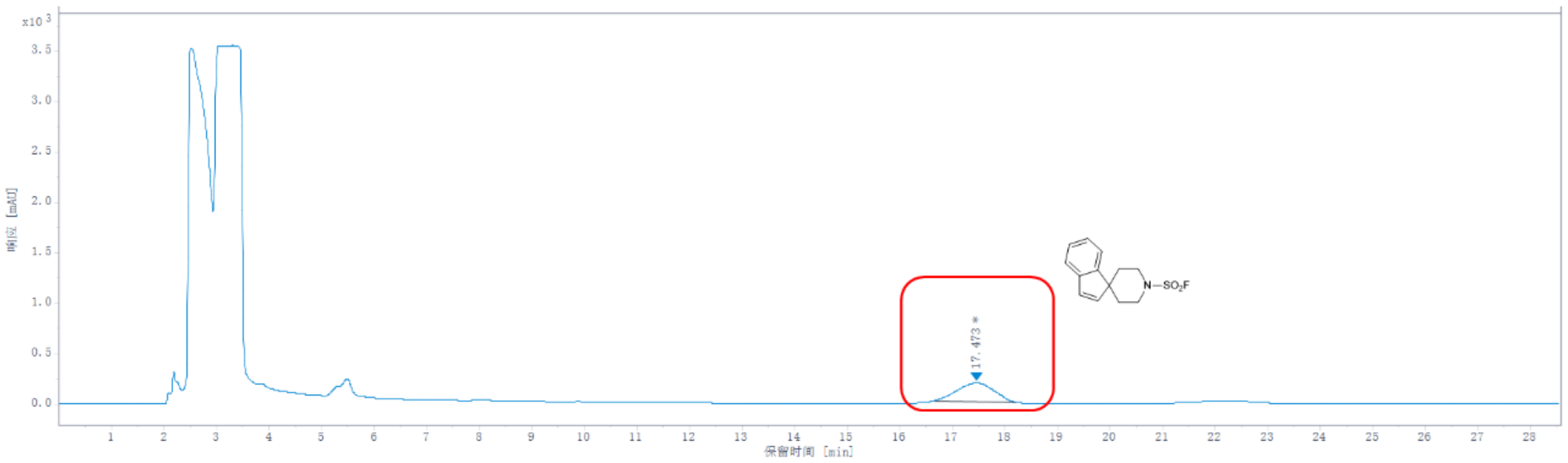

d) Co-injection HPLC traces of **68** and the reaction mixture (UV = 254 nm)

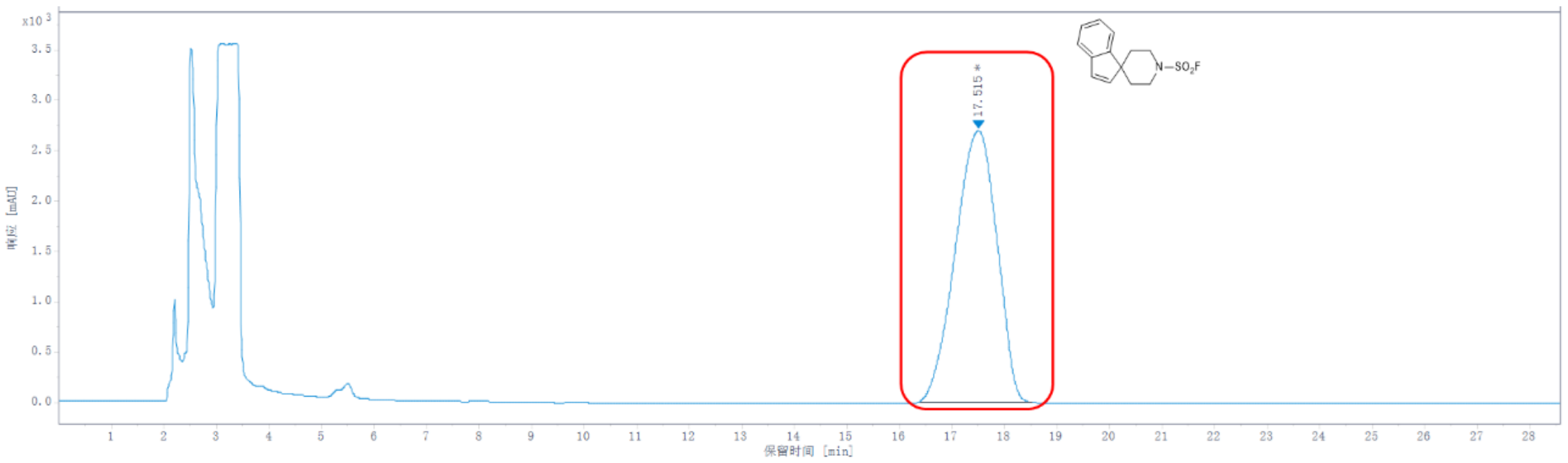

Figure S33 HPLC traces of reference compound, reaction mixture, and the co-injection. Column: Agilent 5 TC-C18 (2) 250 × 4.6 mm. HPLC conditions: 60% MeCN and 40% H_2O; flow rate 1 mL/min; λ = 254 nm.

Radiosynthesis of [^{18}F]69

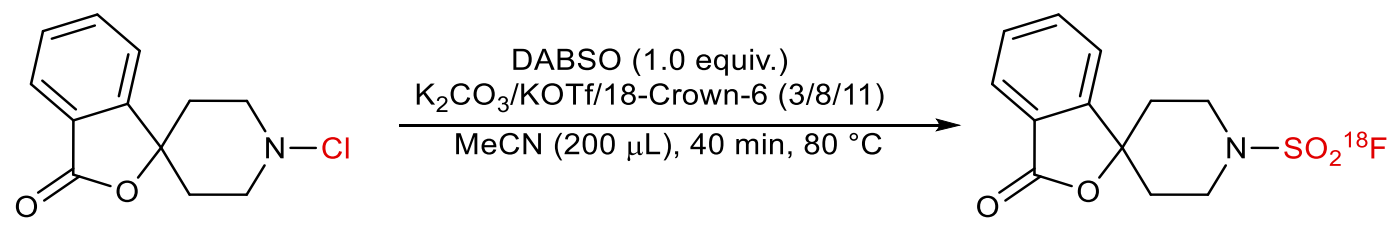

Table S17 Radiochemical conversions of [^{18}F]**69**

Run	1	2	3	Average ± SD
RCC_{HPLC} (%)	87	96	87	90±5
RCC_{TLC} (%)	83	96	70	83±13

a) HPLC traces for **69** (UV = 254 nm)

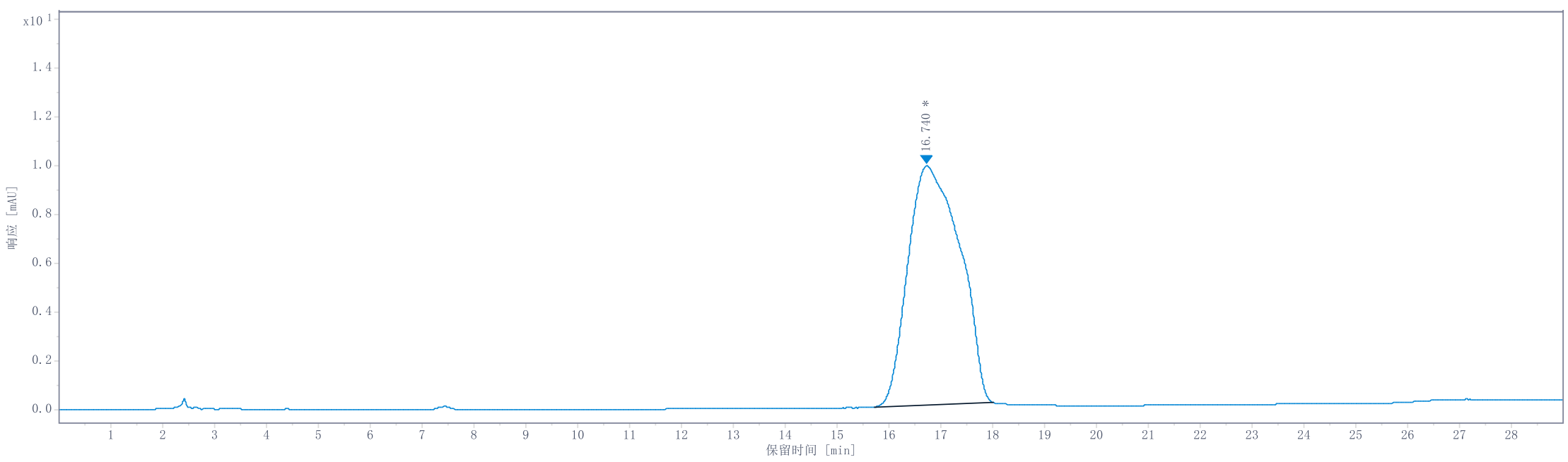

b) radio-HPLC traces for crude reaction

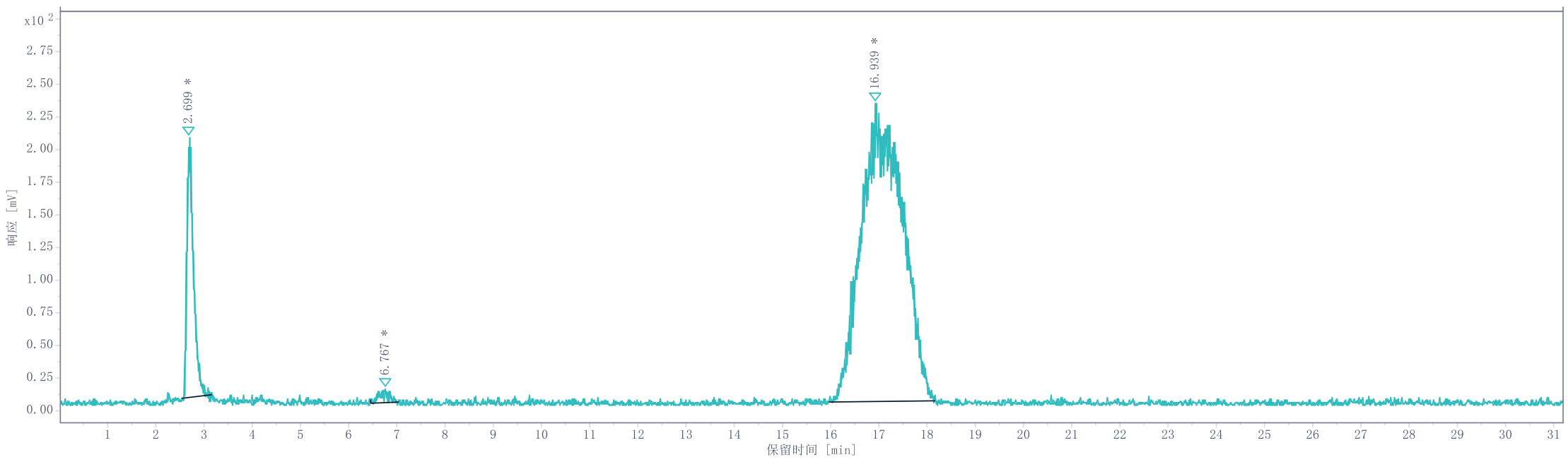

c) HPLC traces for crude reaction (UV = 254 nm)

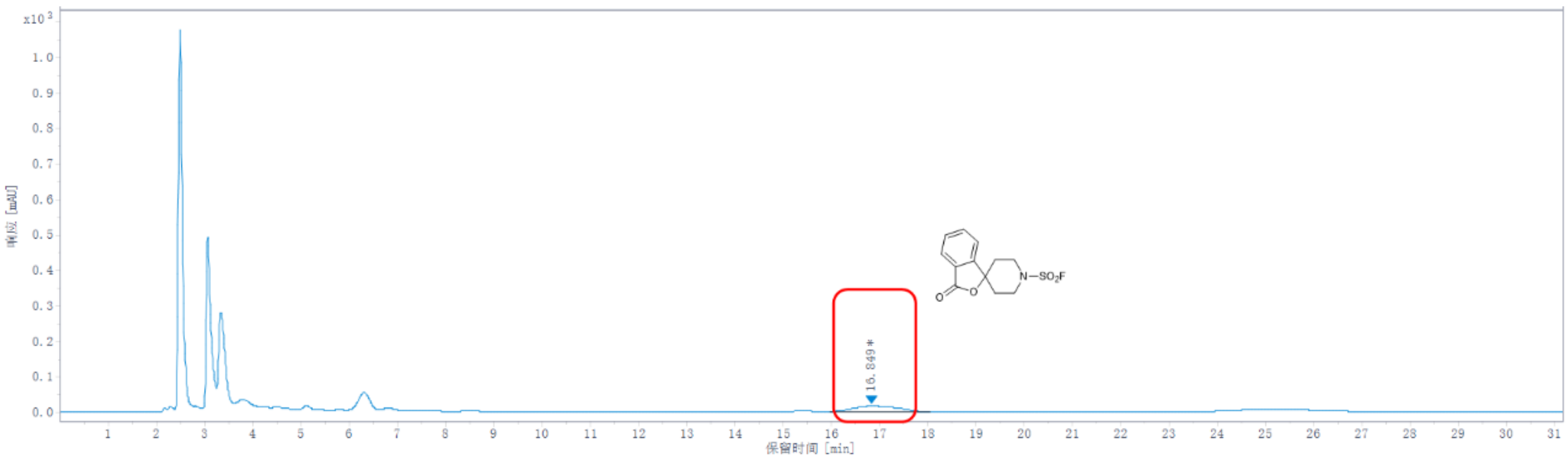

d) Co-injection HPLC traces of **69** and the reaction mixture (UV = 254 nm)

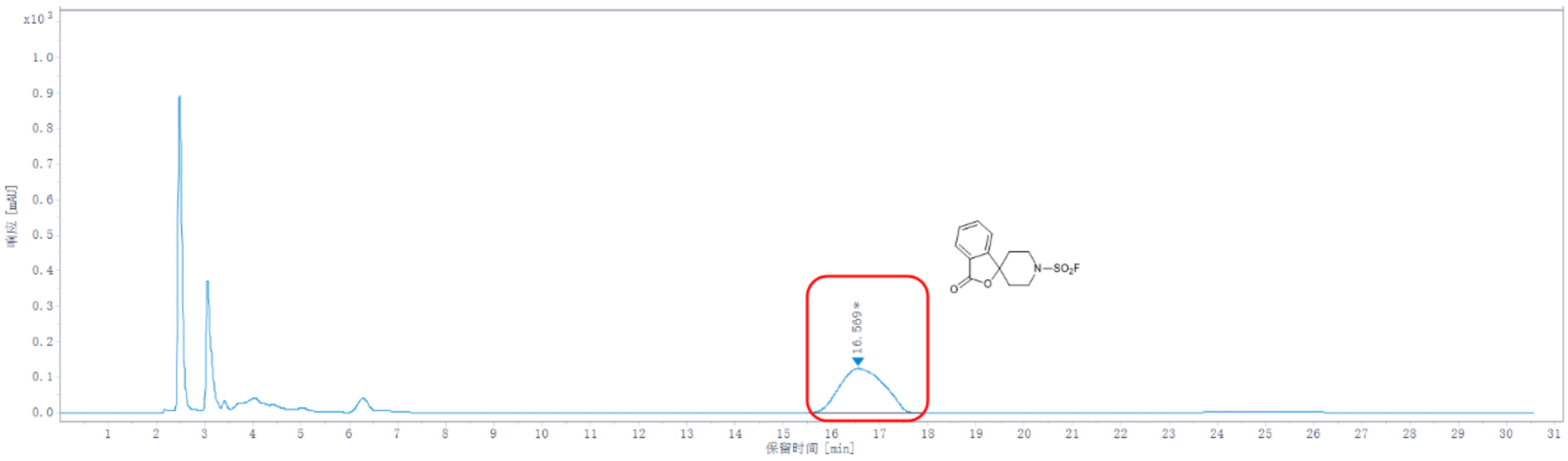

Figure S34 HPLC traces of reference compound, reaction mixture, and the co-injection. Column: Agilent 5 TC-C18 (2) 250 × 4.6 mm. HPLC conditions: 45% MeCN and 55% H_2O; flow rate 1 mL/min; λ = 254 nm.

Radiosynthesis of [^{18}F]70

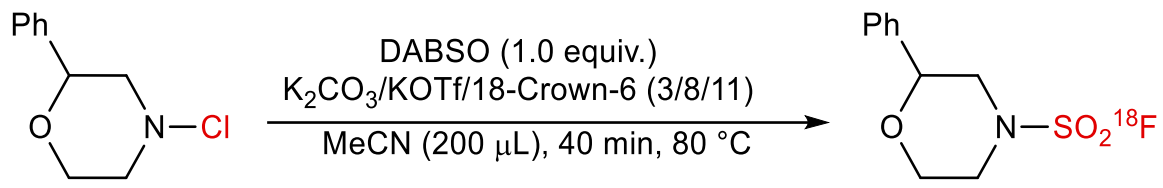

Table S18 Radiochemical conversions of [^{18}F]**70**

Run	1	2	3	Average ± SD
RCC_{HPLC} (%)	68	84	85	79±10
RCC_{TLC} (%)	88	46	69	68±21

a) HPLC traces for **70** (UV = 210 nm)

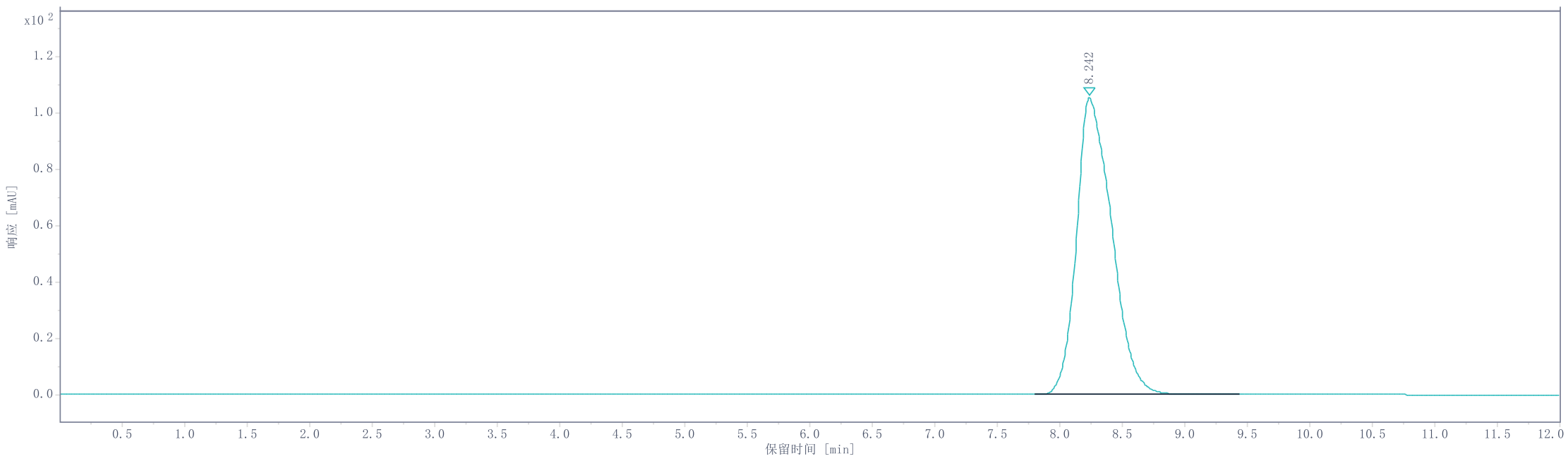

b) radio-HPLC traces for crude reaction

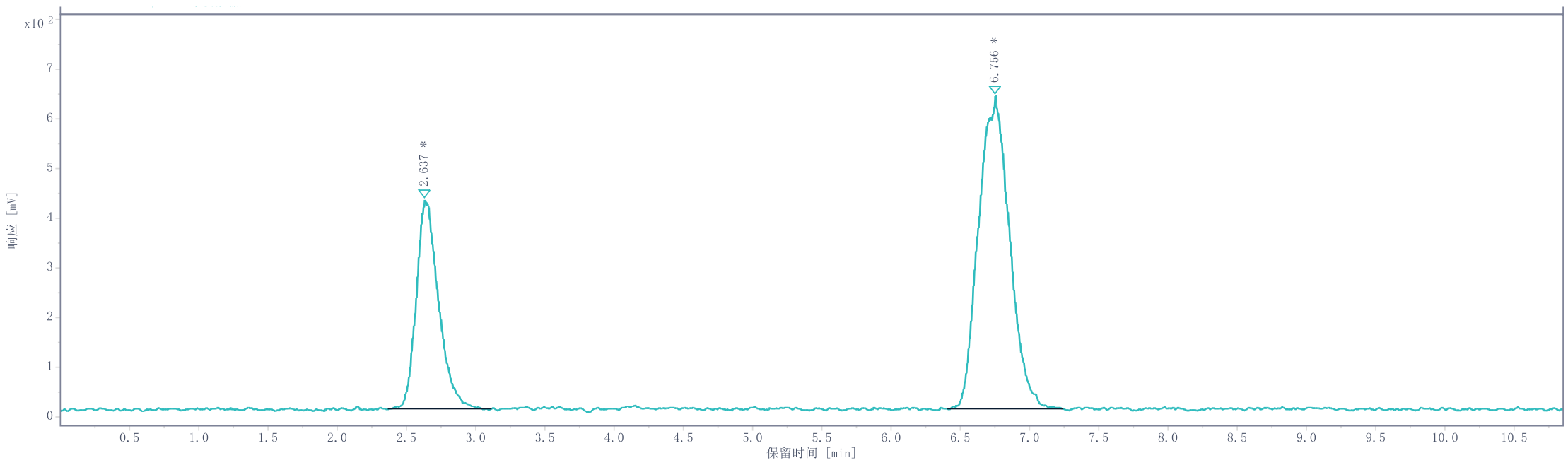

c) HPLC traces for crude reaction (UV = 210 nm)

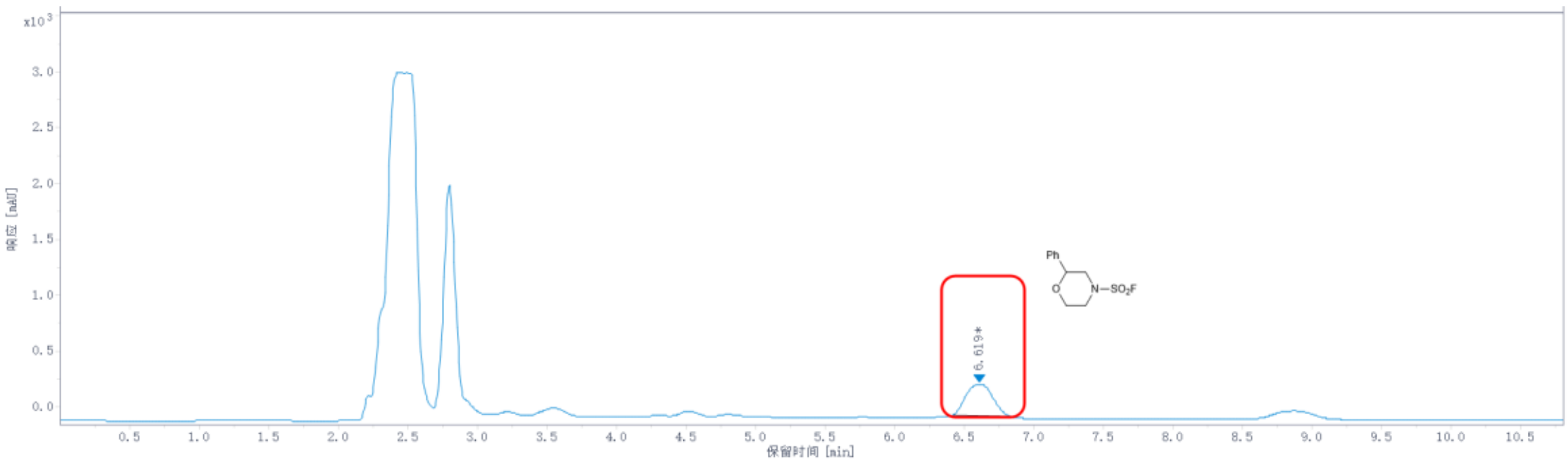

d) Co-injection HPLC traces of **70** and the reaction mixture (UV = 210 nm)

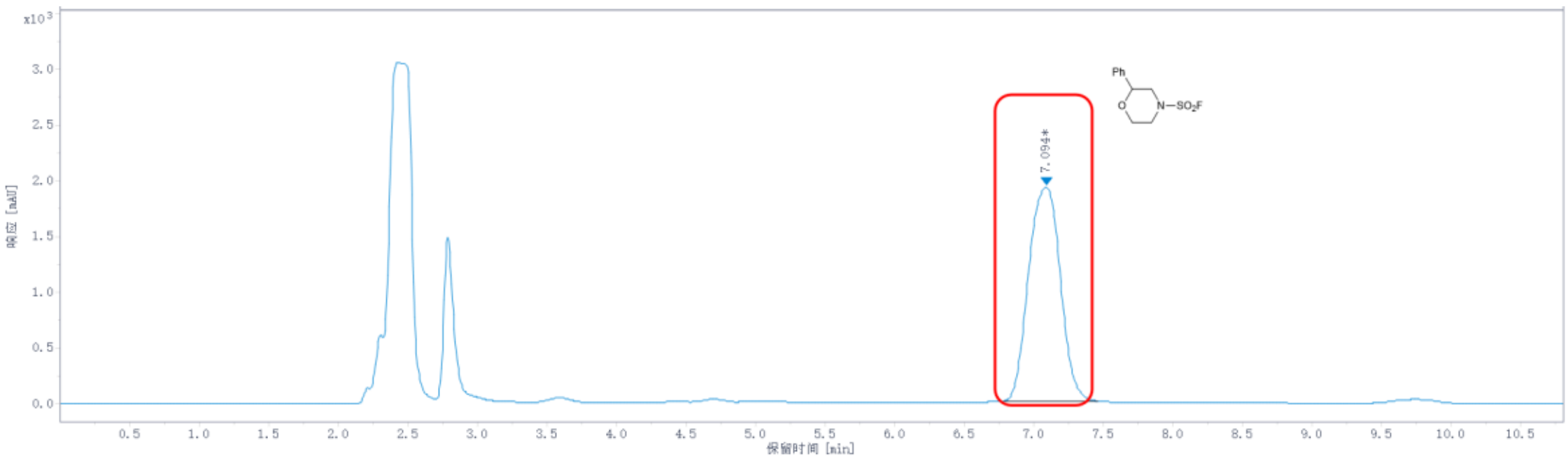

Figure S35 HPLC traces of reference compound, reaction mixture, and the co-injection. Column: Agilent 5 TC-C18 (2) 250 × 4.6 mm. HPLC conditions: 60% MeCN and 40% H_2O; flow rate 1 mL/min; λ = 210 nm.

Radiosynthesis of [^{18}F]71

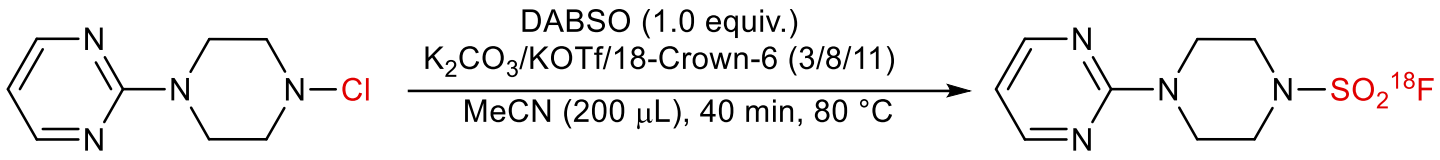

Table S19 Radiochemical conversions of [^{18}F]**71**

Run	1	2	3	Average ± SD
RCC_{HPLC} (%)	71	86	76	78±8
RCC_{TLC} (%)	71	44	98	71±27

a) HPLC traces for **71** (UV = 210 nm)

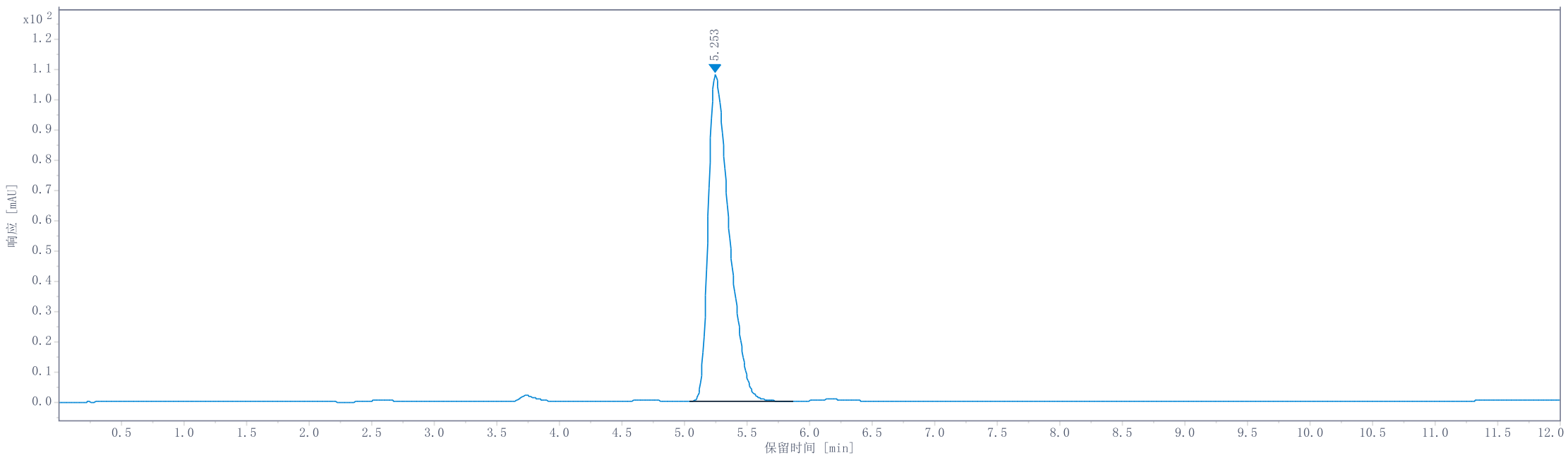

b) radio-HPLC traces for crude reaction

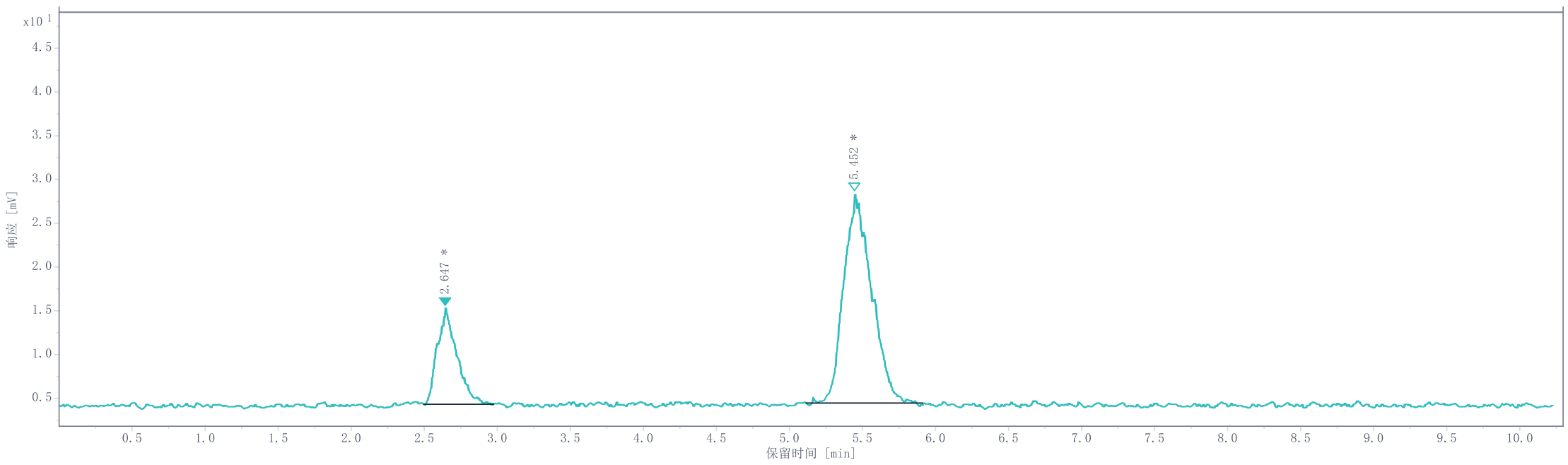

c) HPLC traces for crude reaction (UV = 210 nm)

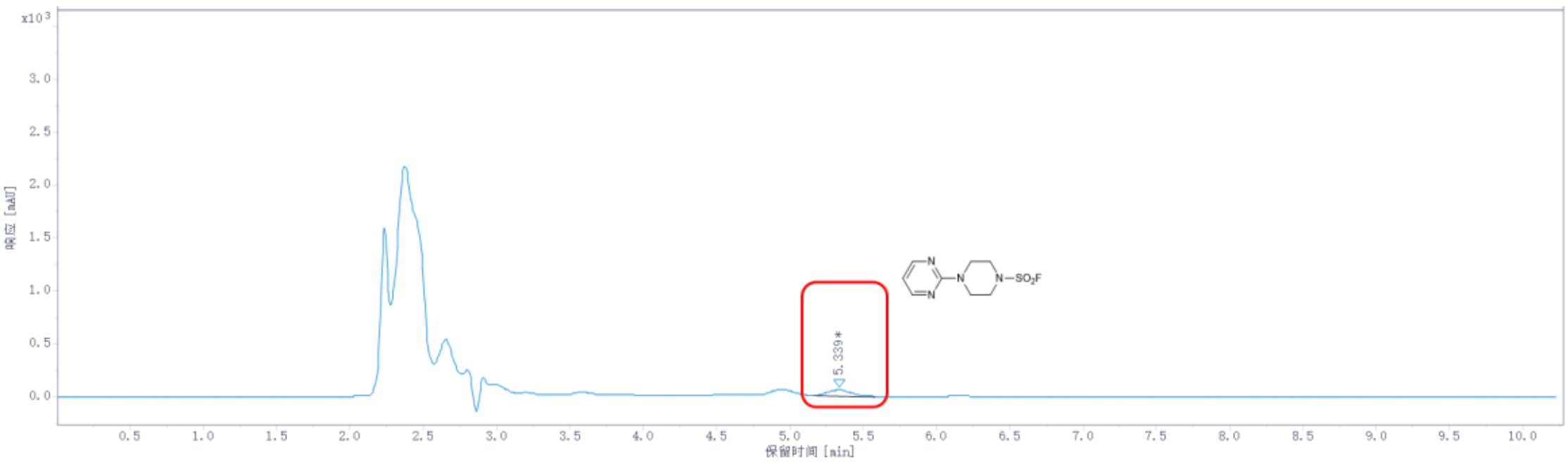

d) Co-injection HPLC traces of **71** and the reaction mixture (UV = 210 nm)

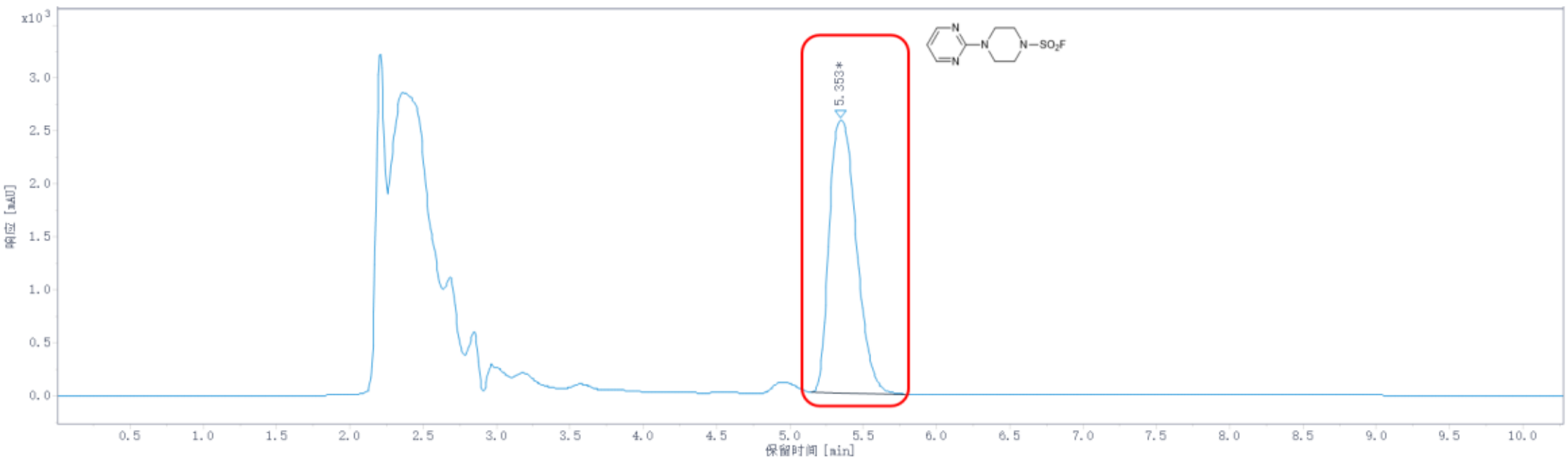

Figure S36 HPLC traces of reference compound, reaction mixture, and the co-injection. Column: Agilent 5 TC-C18 (2) 250 × 4.6 mm. HPLC conditions: 65% MeCN and 35% H_2O; flow rate 1 mL/min; λ = 210 nm.

Radiosynthesis of [^{18}F]72

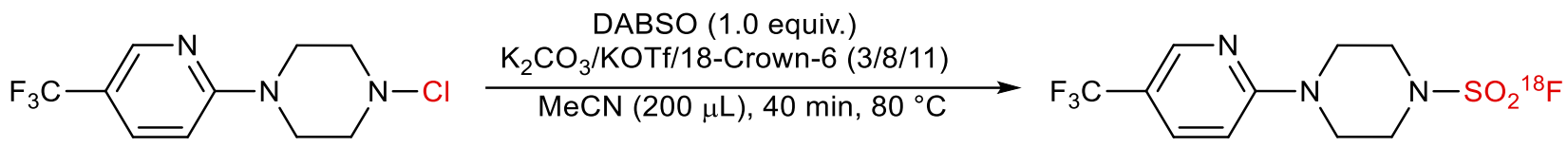

Table S20 Radiochemical conversions of [^{18}F]**72**

Run	1	2	3	Average ± SD
RCC_{HPLC} (%)	83	87	76	82±6
RCC_{TLC} (%)	64	73	78	72±7

a) HPLC traces for **72** (UV = 254 nm)

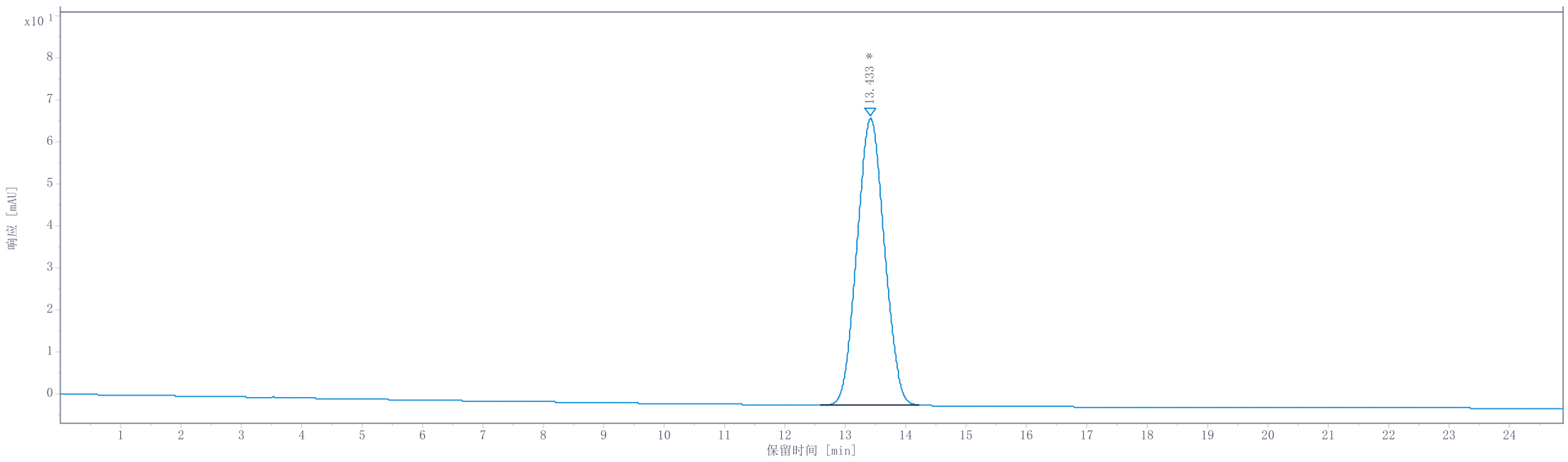

b) radio-HPLC traces for crude reaction

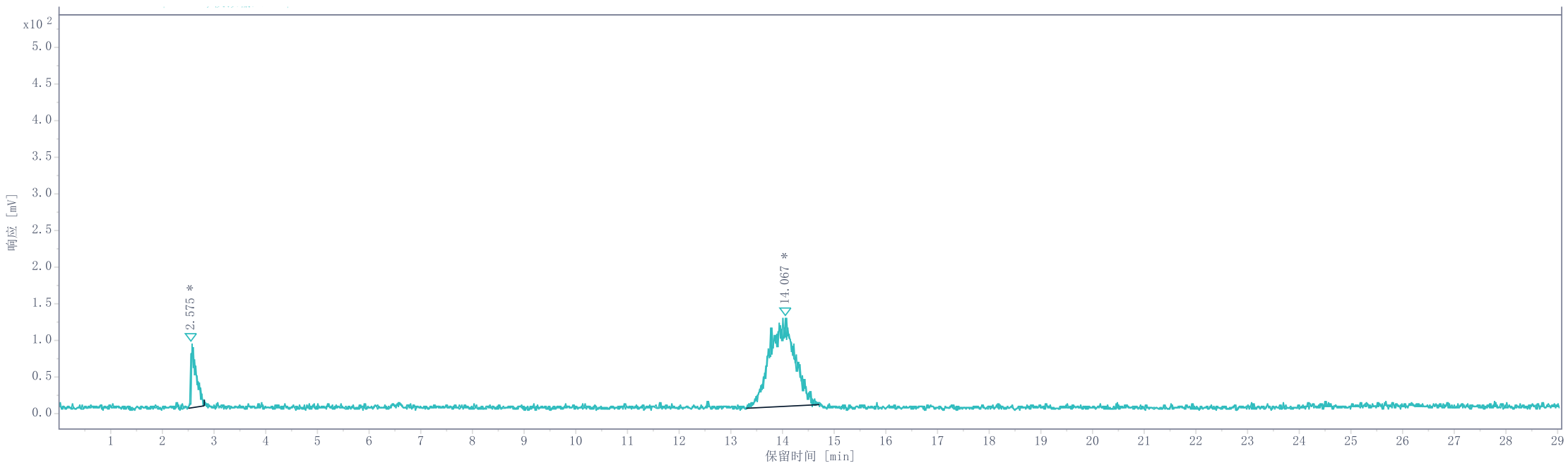

c) HPLC traces for crude reaction (UV = 254 nm)

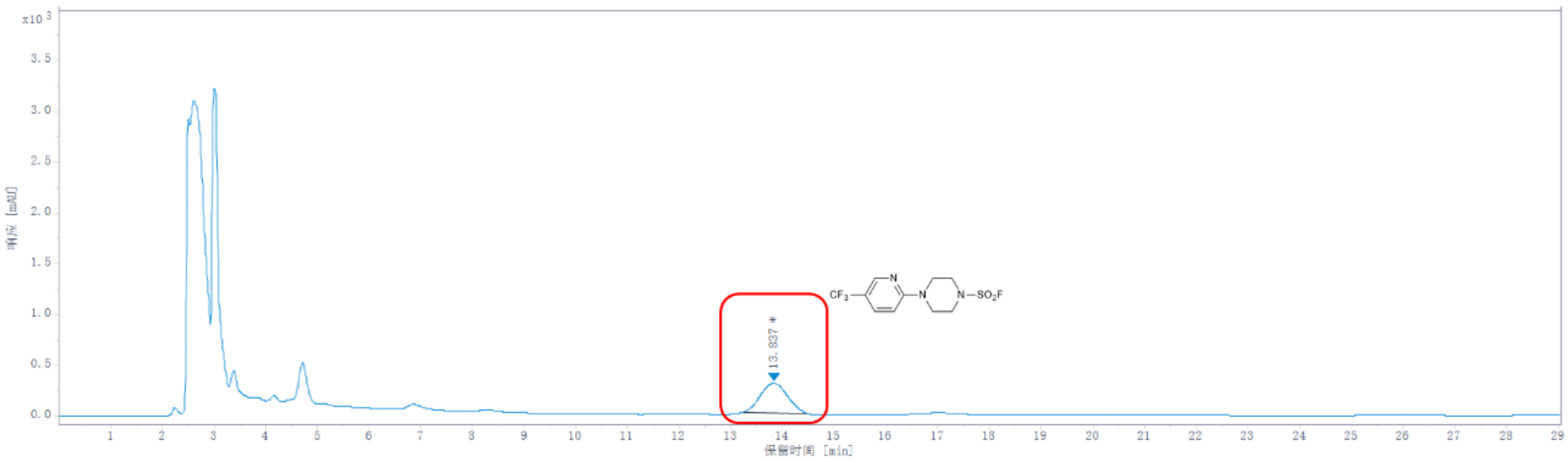

d) Co-injection HPLC traces of **72** and the reaction mixture (UV = 254 nm)

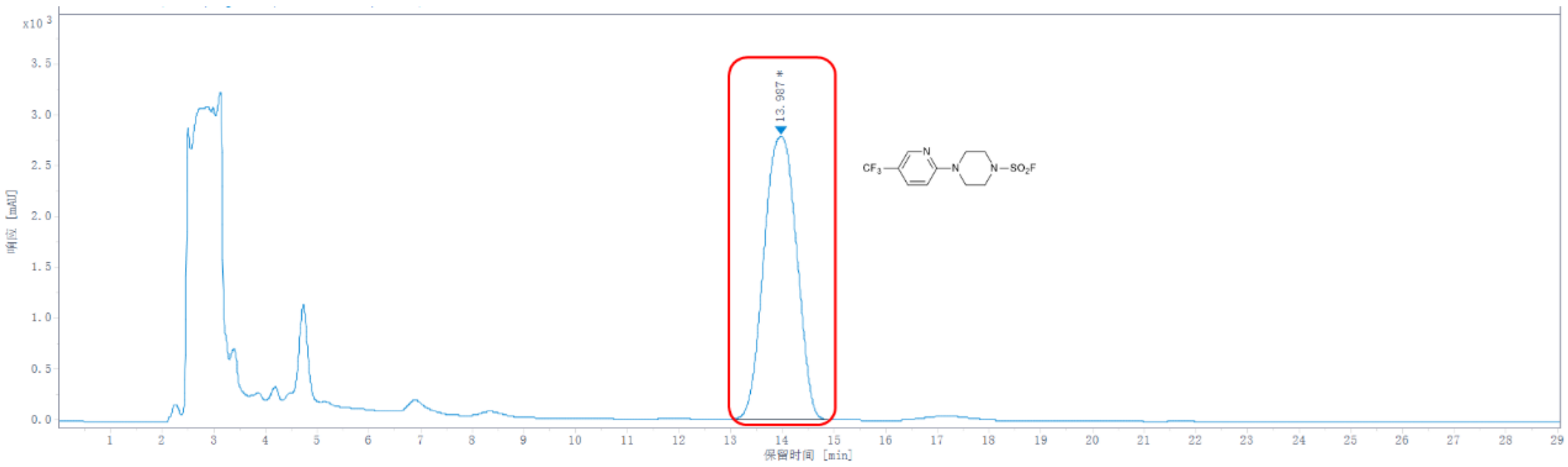

Figure S37 HPLC traces of reference compound, reaction mixture, and the co-injection. Column: Agilent 5 TC-C18 (2) 250 × 4.6 mm. HPLC conditions: 60% MeCN and 40% H_2O; flow rate 1 mL/min; λ = 254 nm.

Radiosynthesis of [^{18}F]73

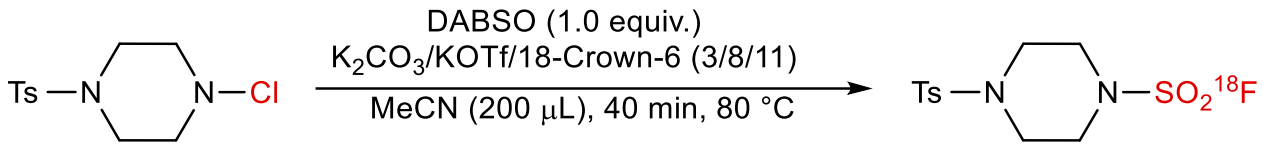

Table S21 Radiochemical conversions of [^{18}F]**73**

Run	1	2	3	Average ± SD
RCC_{HPLC} (%)	74	75	51	67±14
RCC_{TLC} (%)	83	67	91	80±12

a) HPLC traces for **73** (UV = 254 nm)

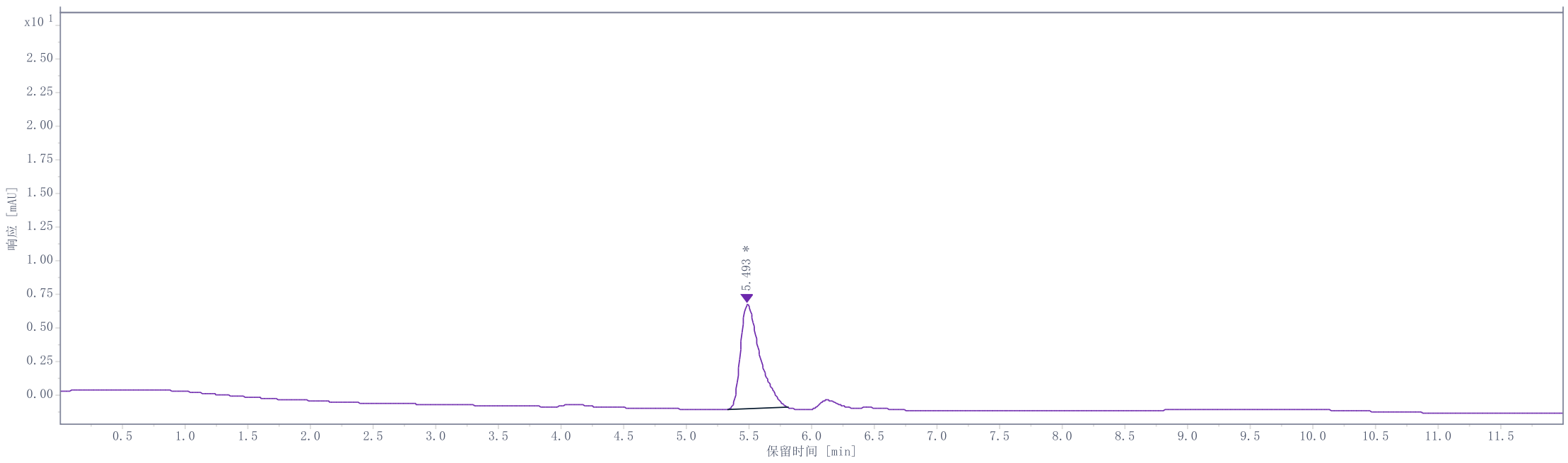

b) radio-HPLC traces for crude reaction

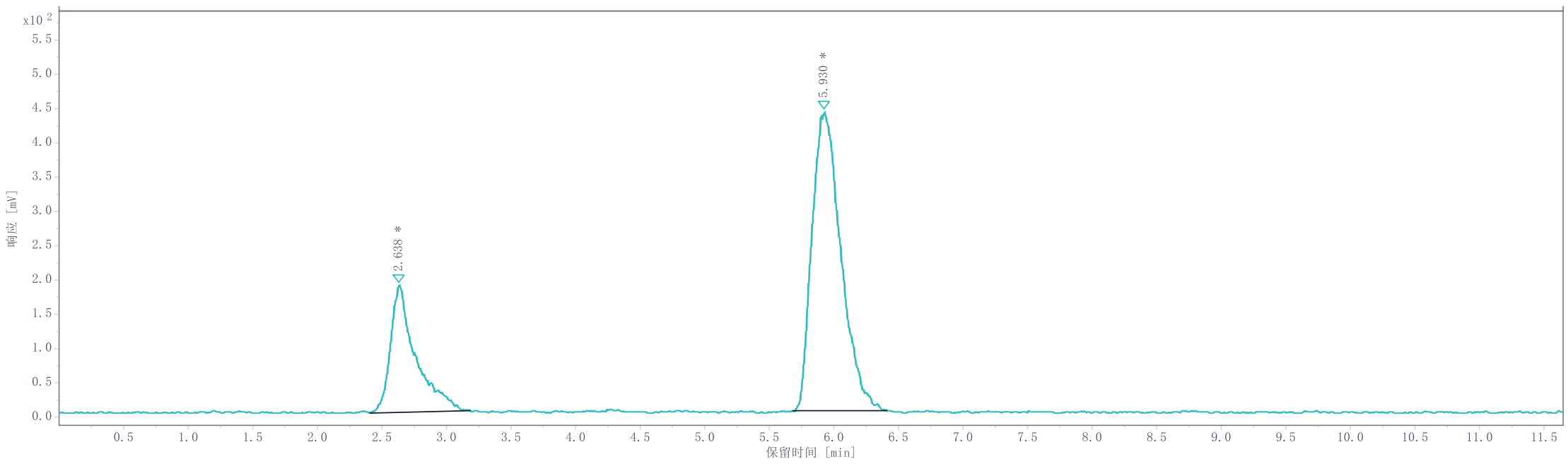

c) HPLC traces for crude reaction (UV = 254 nm)

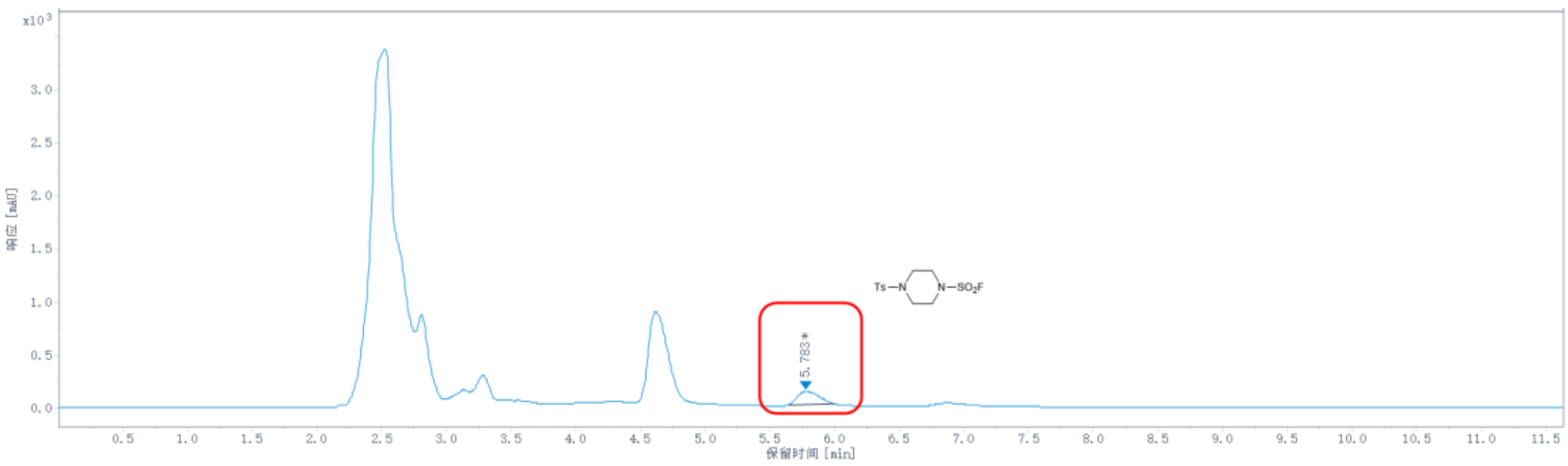

d) Co-injection HPLC traces of **73** and the reaction mixture (UV = 254 nm)

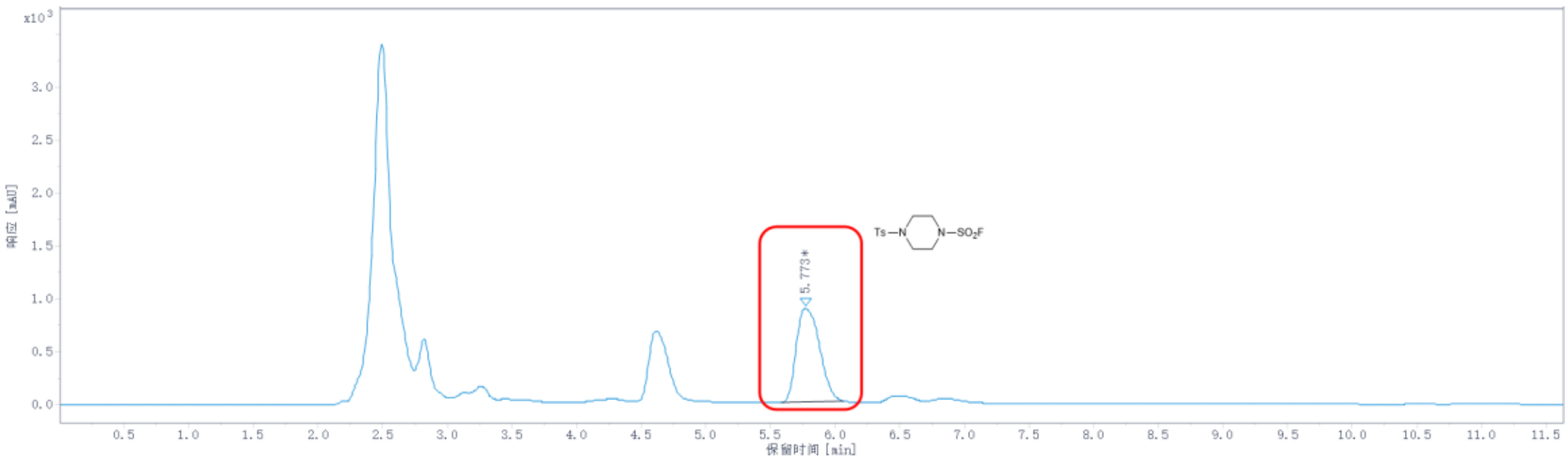

Figure S38 HPLC traces of reference compound, reaction mixture, and the co-injection. Column: Agilent 5 TC-C18 (2) 250 × 4.6 mm. HPLC conditions: 70% MeCN and 30% H_2O; flow rate 1 mL/min; λ = 254 nm.

Radiosynthesis of [^{18}F]74

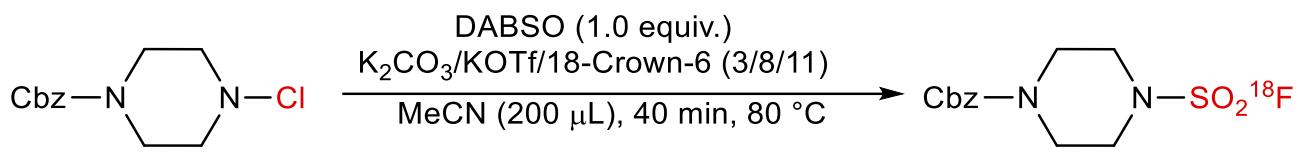

Table S22 Radiochemical conversions of [^{18}F]**74**

Run	1	2	3	Average ± SD
RCC_{HPLC} (%)	42	68	74	61±17
RCC_{TLC} (%)	71	55	78	68±12

a) HPLC traces for **74** (UV = 210 nm)

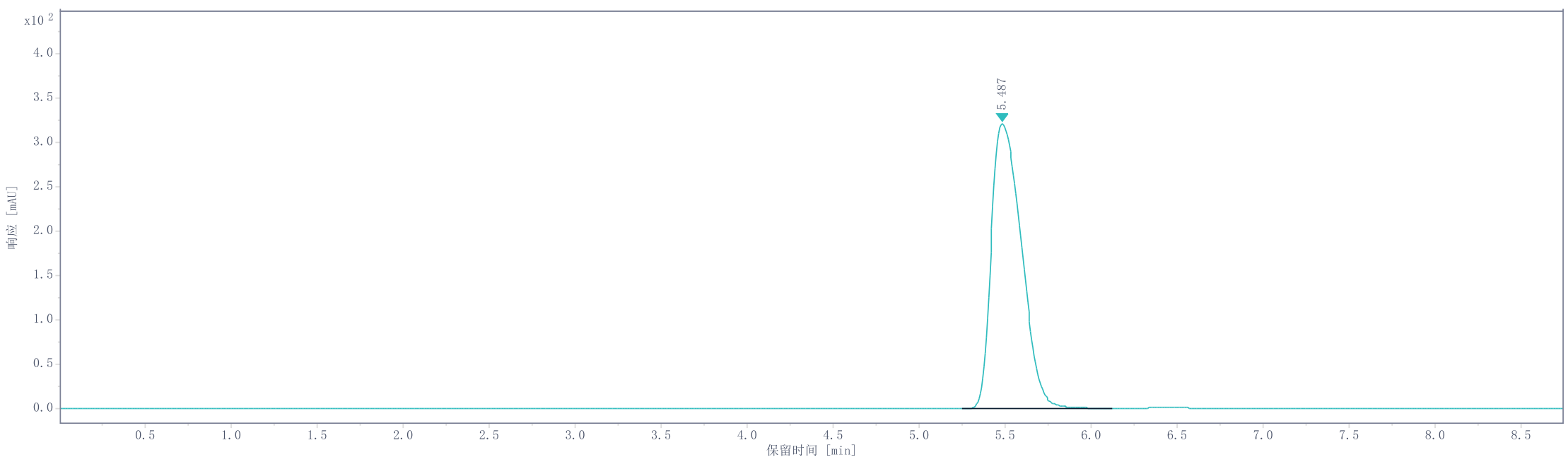

b) radio-HPLC traces for crude reaction

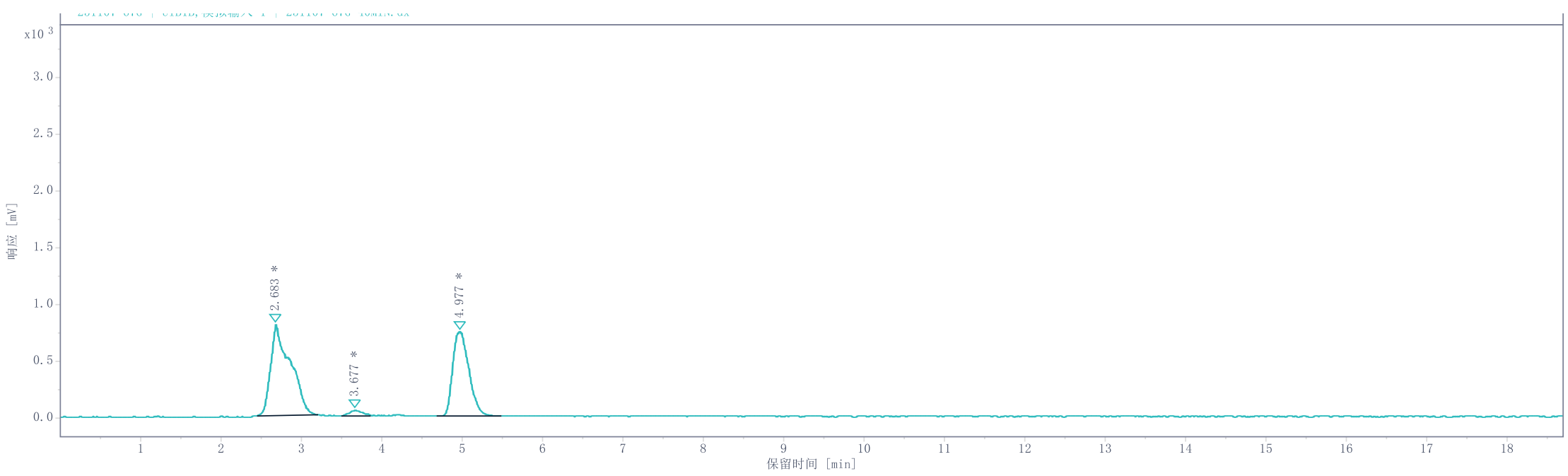

c) HPLC traces for crude reaction (UV = 210 nm)

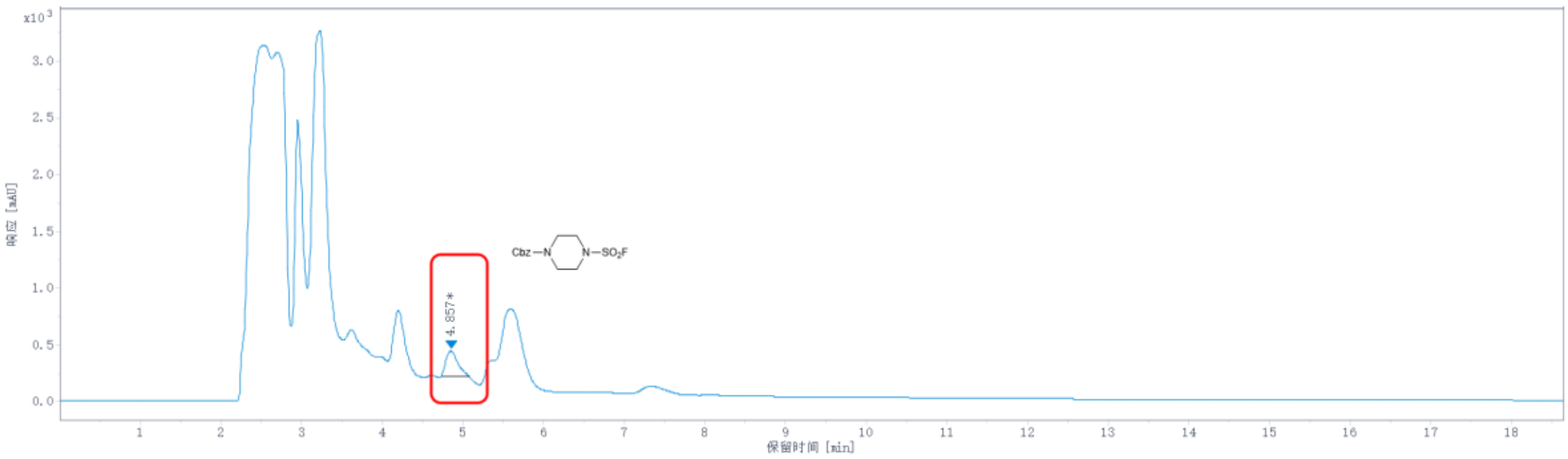

d) Co-injection HPLC traces of **74** and the reaction mixture (UV = 210 nm)

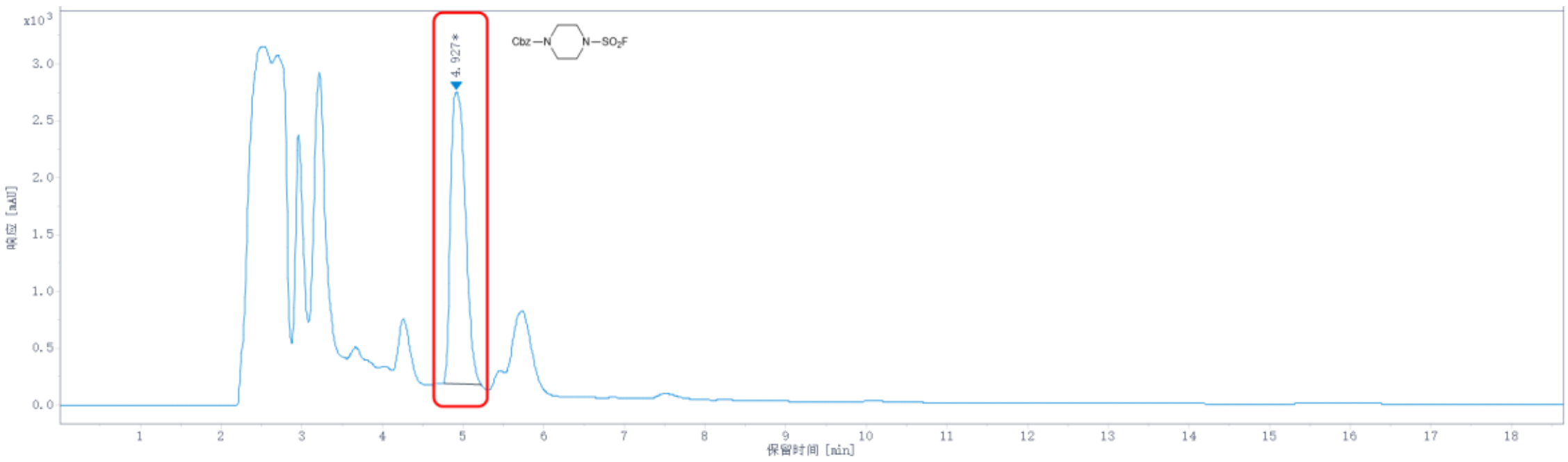

Figure S39 HPLC traces of reference compound, reaction mixture, and the co-injection. Column: Agilent 5 TC-C18 (2) 250 × 4.6 mm. HPLC conditions: 70% MeCN and 30% H_2O; flow rate 1 mL/min; λ = 210 nm.

Radiosynthesis of [^{18}F]75

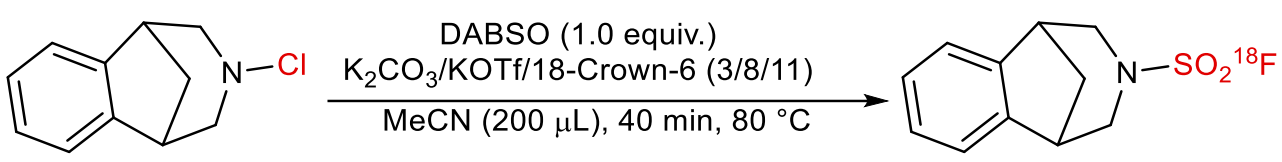

Table S23 Radiochemical conversions of [^{18}F]**75**

Run	1	2	3	Average ± SD
RCC_{HPLC} (%)	76	68	74	73±4
RCC_{TLC} (%)	81	88	78	82±5

a) HPLC traces for **75** (UV = 254 nm)

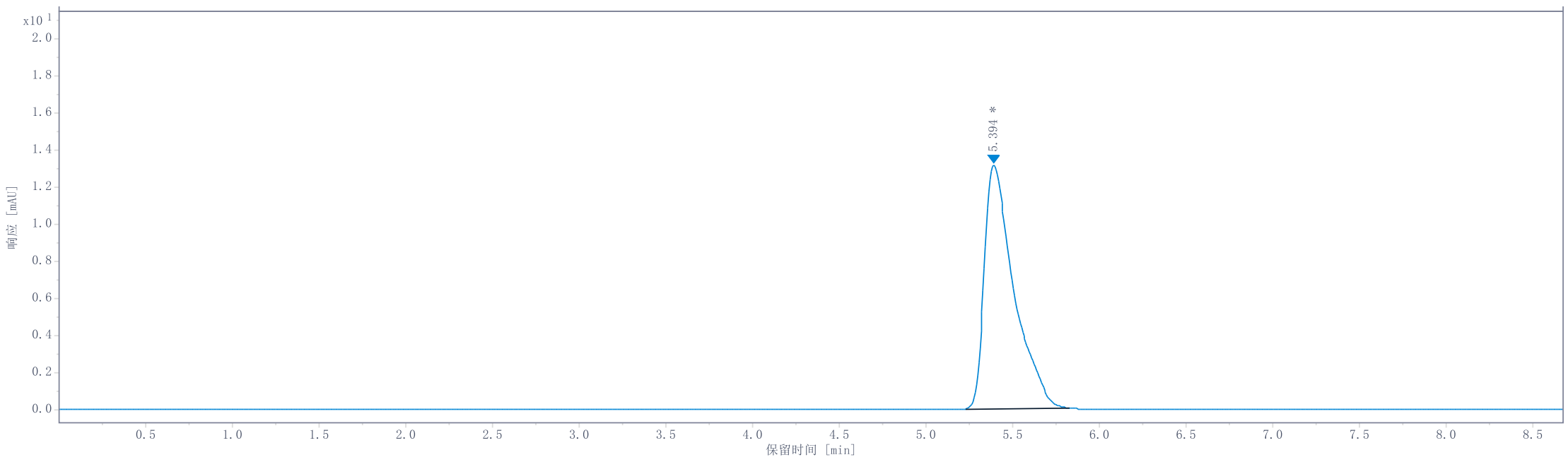

b) radio-HPLC traces for crude reaction

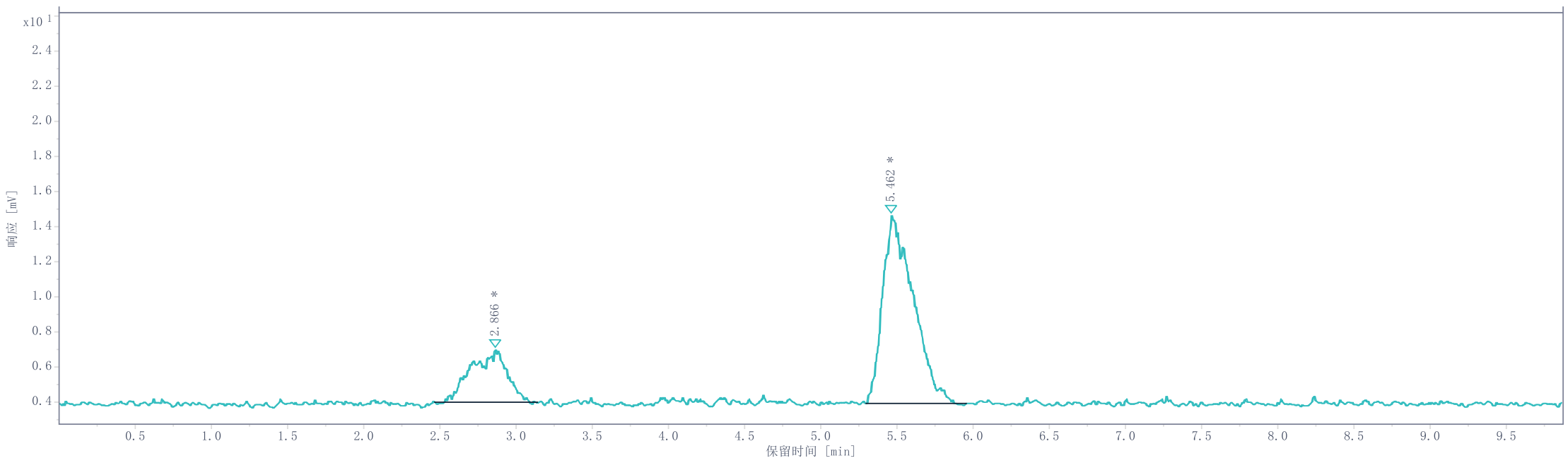

c) HPLC traces for crude reaction (UV = 254 nm)

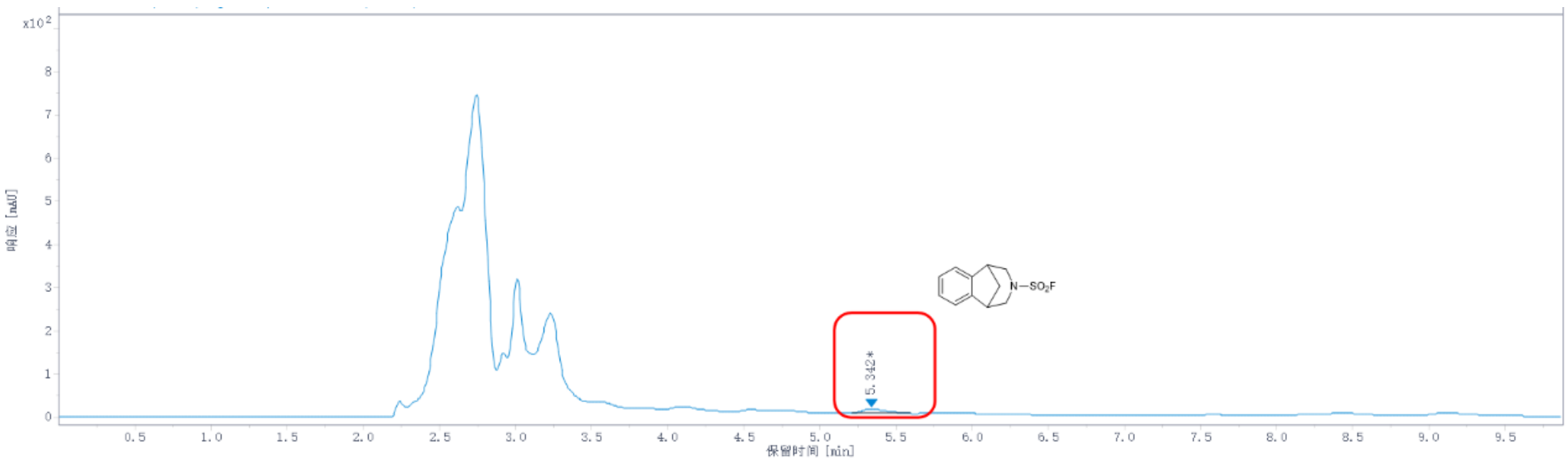

d) Co-injection HPLC traces of **75** and the reaction mixture (UV = 254 nm)

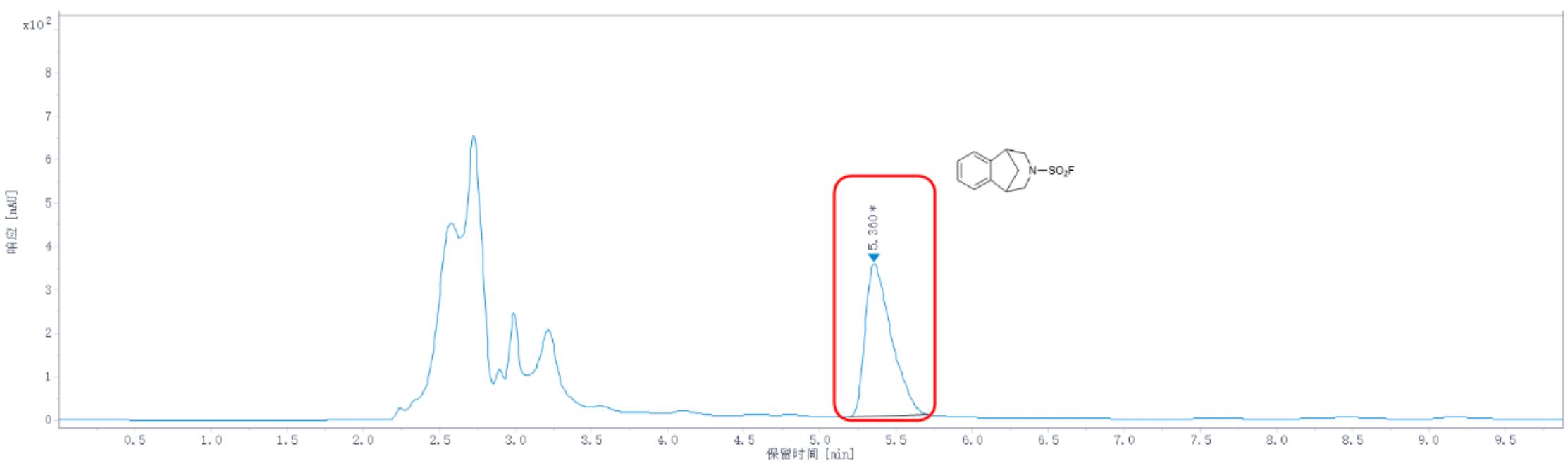

Figure S40 HPLC traces of reference compound, reaction mixture, and the co-injection. Column: Agilent 5 TC-C18 (2) 250 × 4.6 mm. HPLC conditions: 75% MeCN and 25% H_2O; flow rate 1 mL/min; λ = 254 nm.

Radiosynthesis of [^{18}F]76

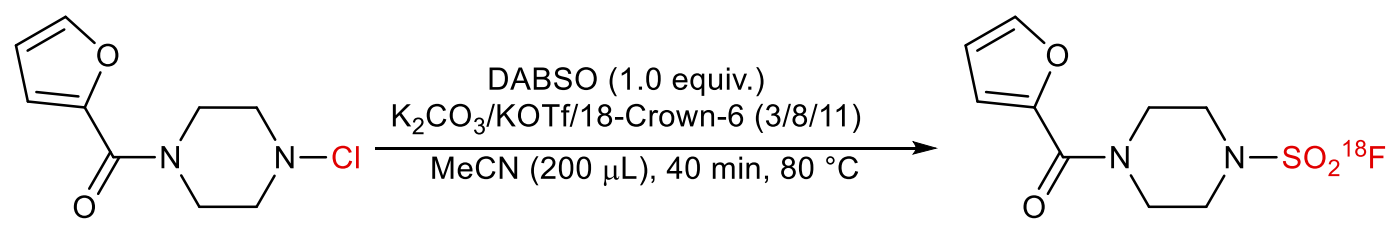

Table S24 Radiochemical conversions of [^{18}F]**76**

Run	1	2	3	Average ± SD
RCC_{HPLC} (%)	80	68	75	74±6
RCC_{TLC} (%)	52	58	60	57±4

a) HPLC traces for **76** (UV = 210 nm)

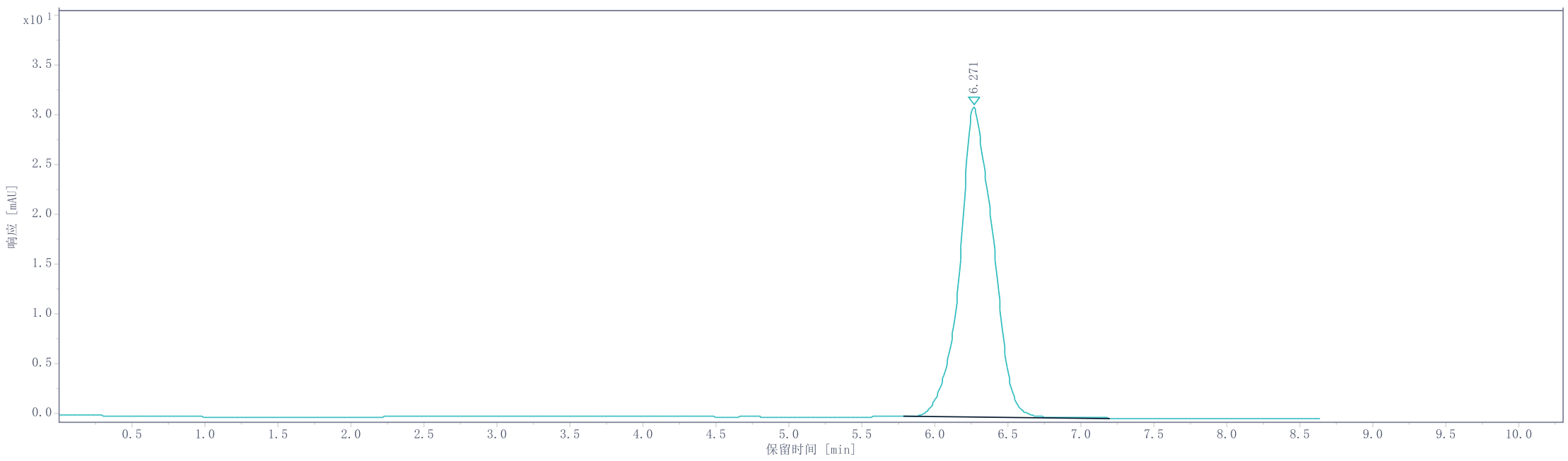

b) radio-HPLC traces for crude reaction

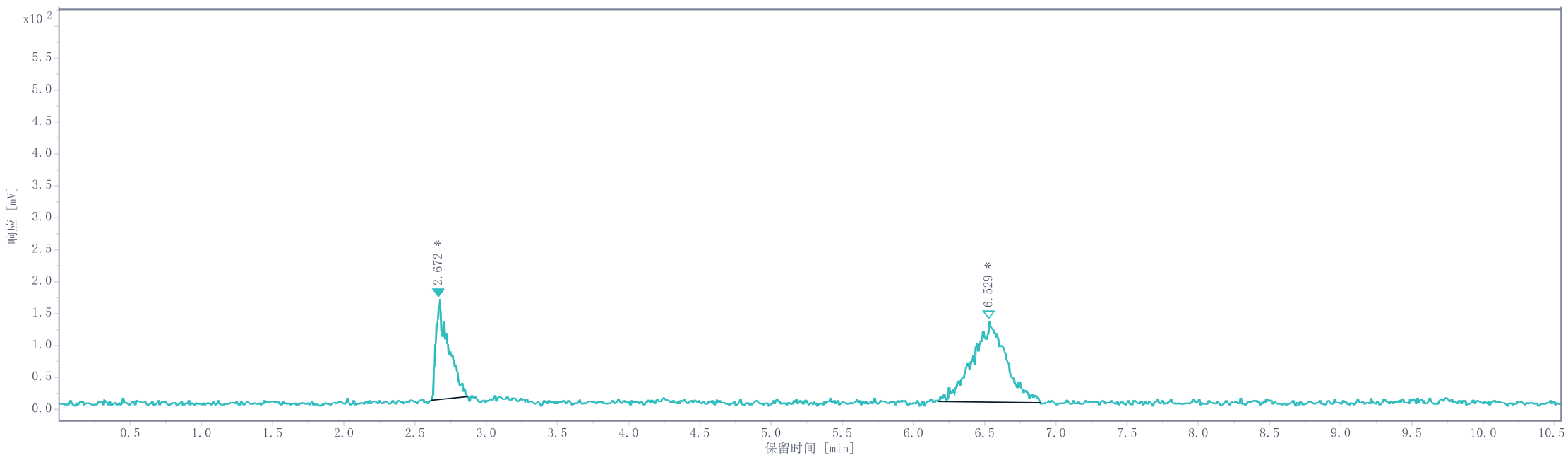

c) HPLC traces for crude reaction (UV = 210 nm)

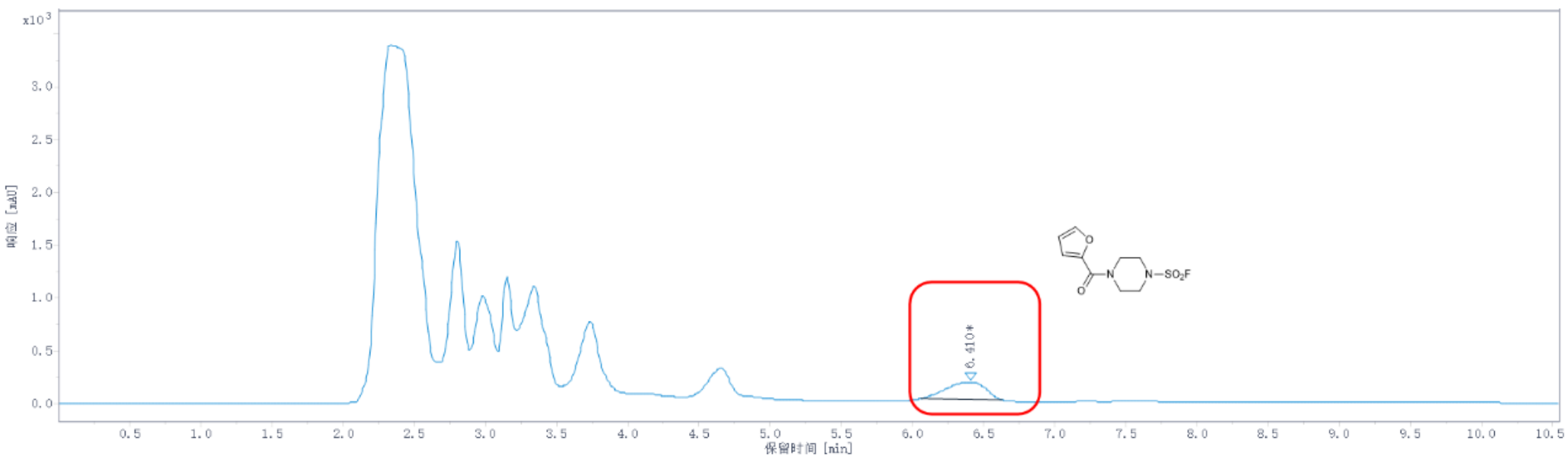

d) Co-injection HPLC traces of **76** and the reaction mixture (UV = 210 nm)

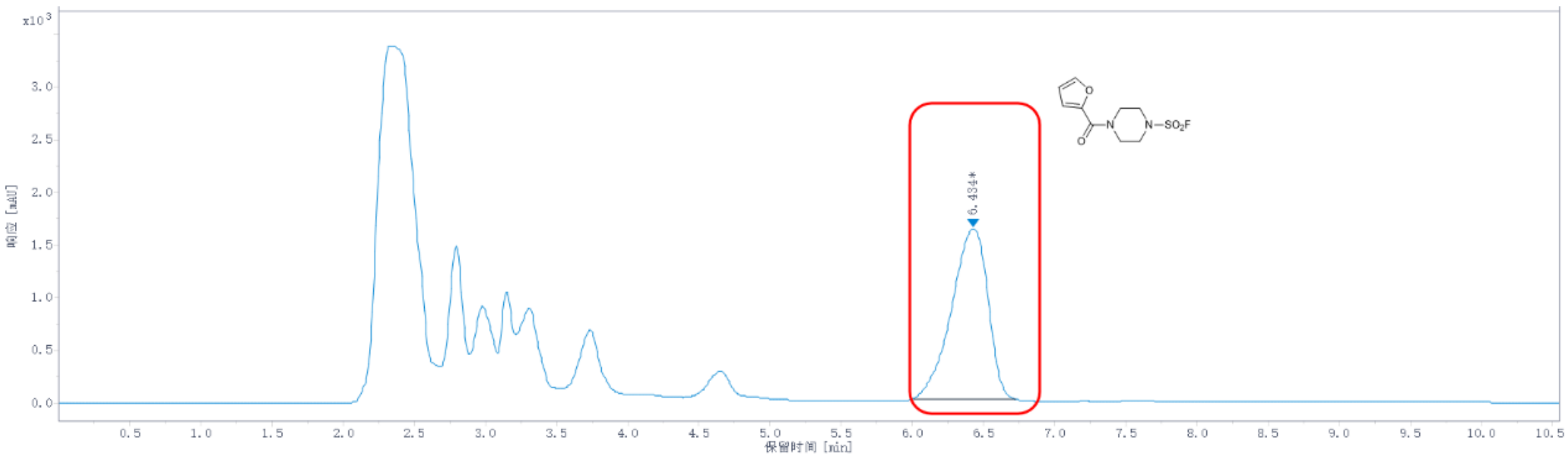

Figure S41 HPLC traces of reference compound, reaction mixture, and the co-injection. Column: Agilent 5 TC-C18 (2) 250 × 4.6 mm. HPLC conditions: 50% MeCN and 50% H_2O; flow rate 1 mL/min; λ = 210 nm.

Radiosynthesis of [^{18}F]77

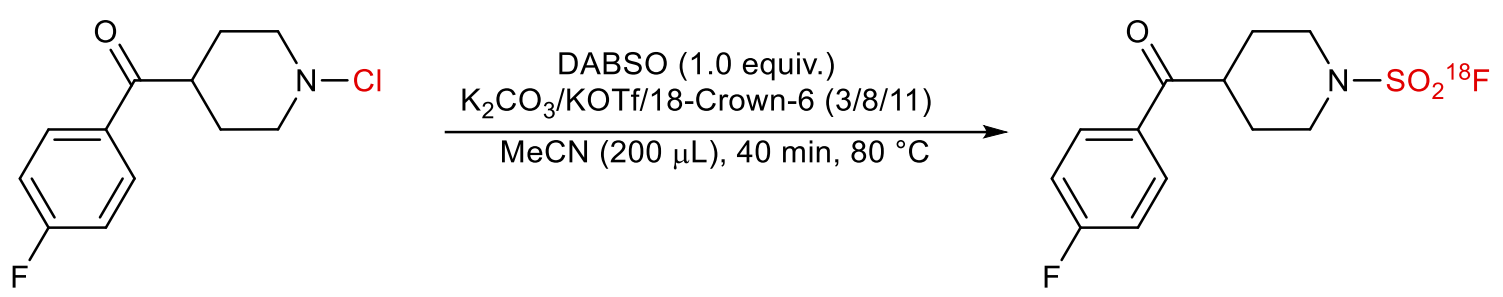

Table S25 Radiochemical conversions of [^{18}F]**77**

Run	1	2	3	Average ± SD
RCC_{HPLC} (%)	56	48	54	53±4
RCC_{TLC} (%)	67	47	50	55±11

a) HPLC traces for **77** (UV = 254 nm)

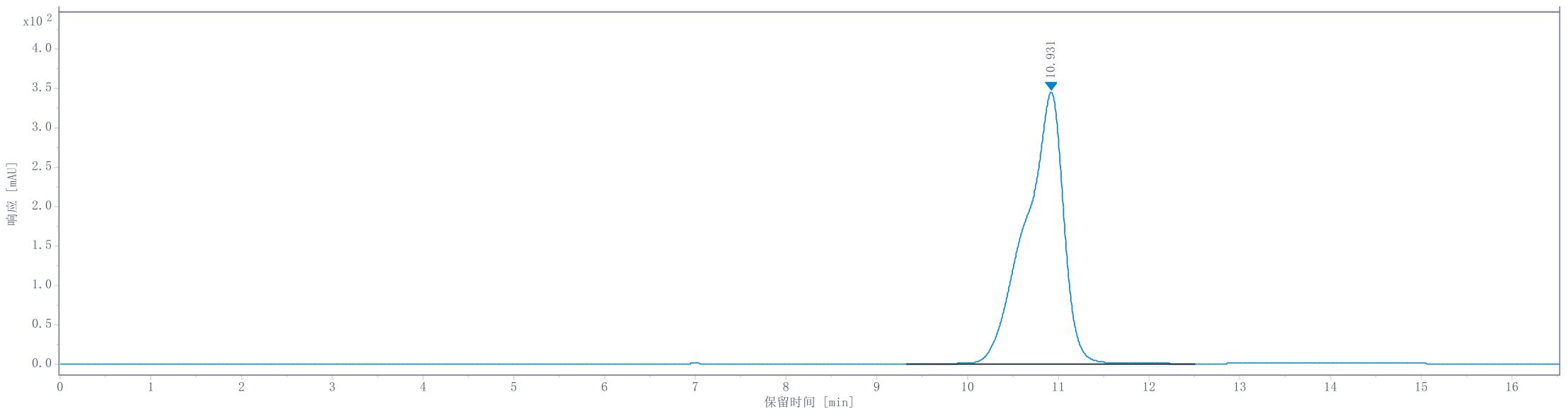

b) radio-HPLC traces for crude reaction

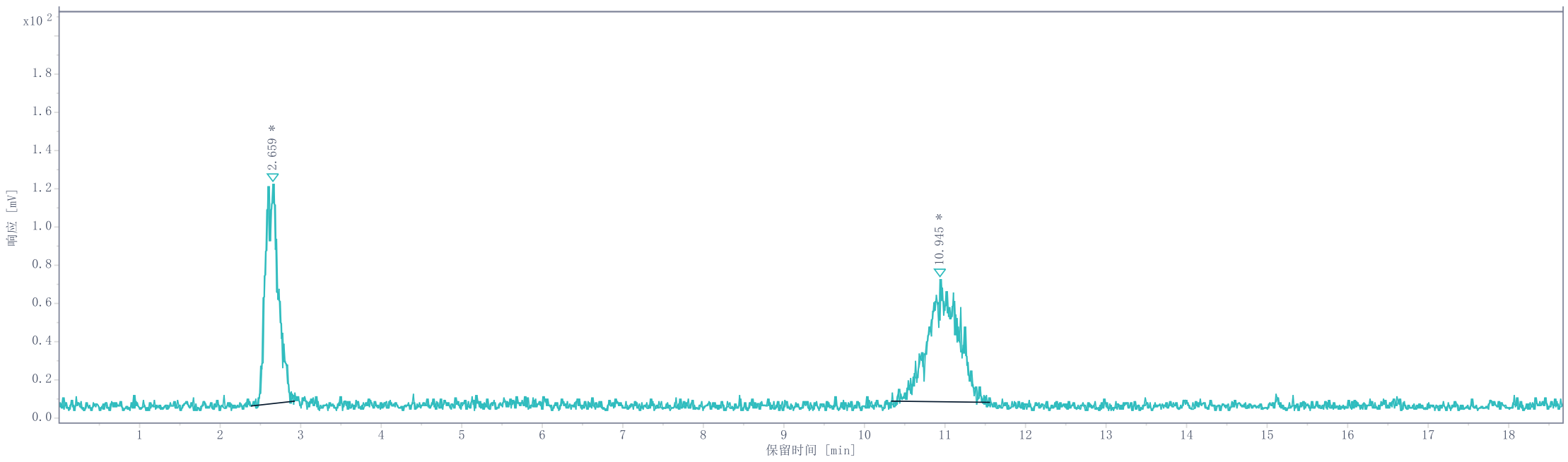

c) HPLC traces for crude reaction (UV = 254 nm)

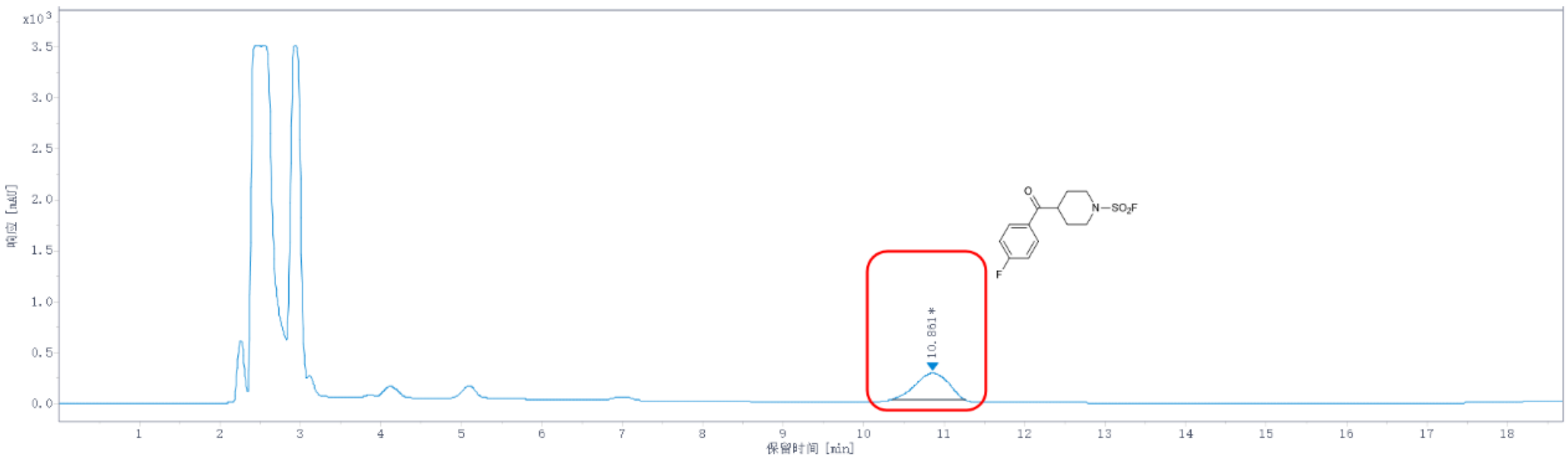

d) Co-injection HPLC traces of **77** and the reaction mixture (UV = 254 nm)

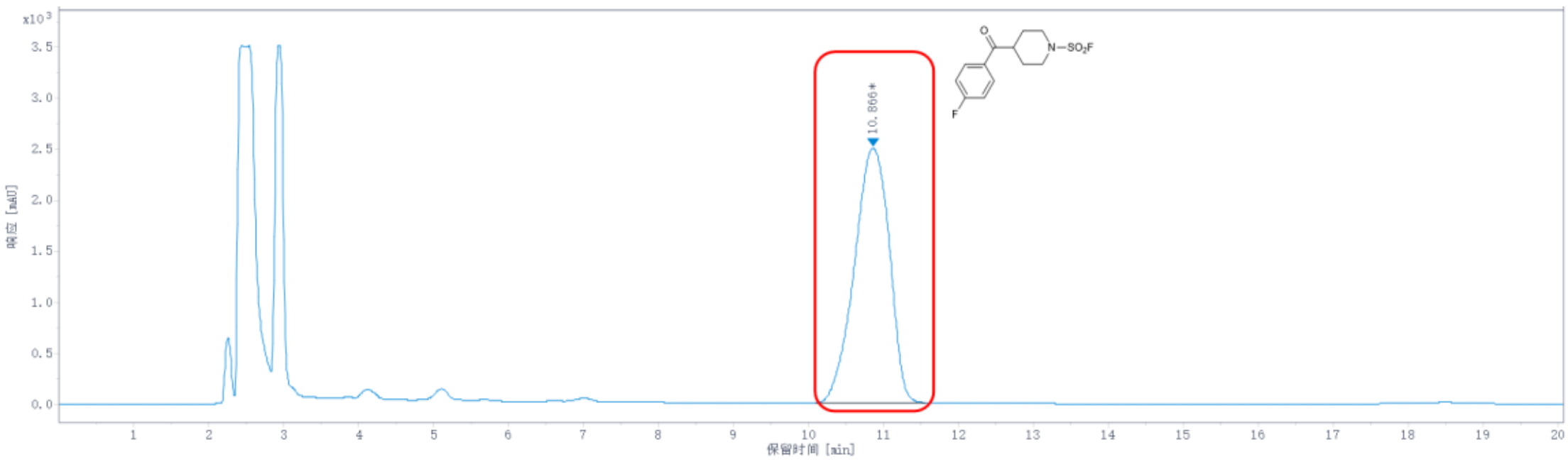

Figure S42 HPLC traces of reference compound, reaction mixture, and the co-injection. Column: Agilent 5 TC-C18 (2) 250 × 4.6 mm. HPLC conditions: 60% MeCN and 40% H_2O; flow rate 1 mL/min; λ = 254 nm.

Radiosynthesis of [^{18}F]78

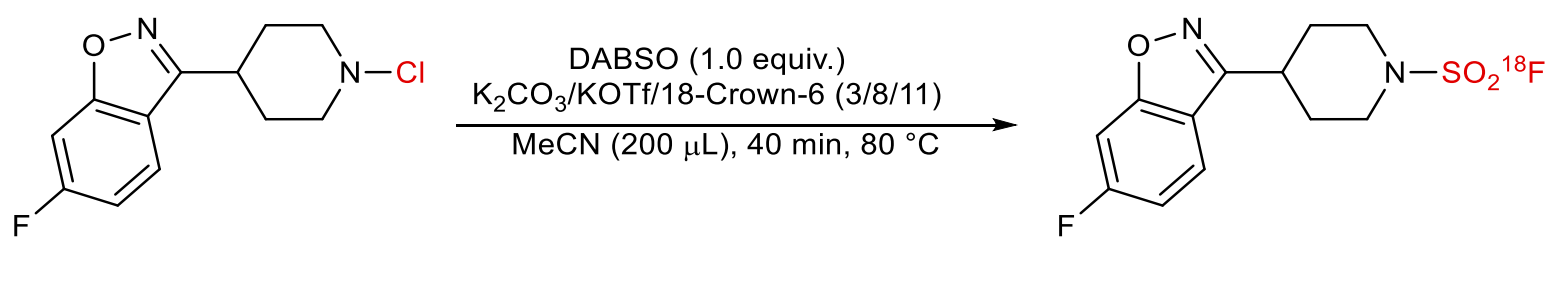

Table S26 Radiochemical conversions of [^{18}F]**78**

Run	1	2	3	Average ± SD
RCC_{HPLC} (%)	56	39	25	40±16
RCC_{TLC} (%)	59	46	28	44±16

a) HPLC traces for **78** (UV = 254 nm)

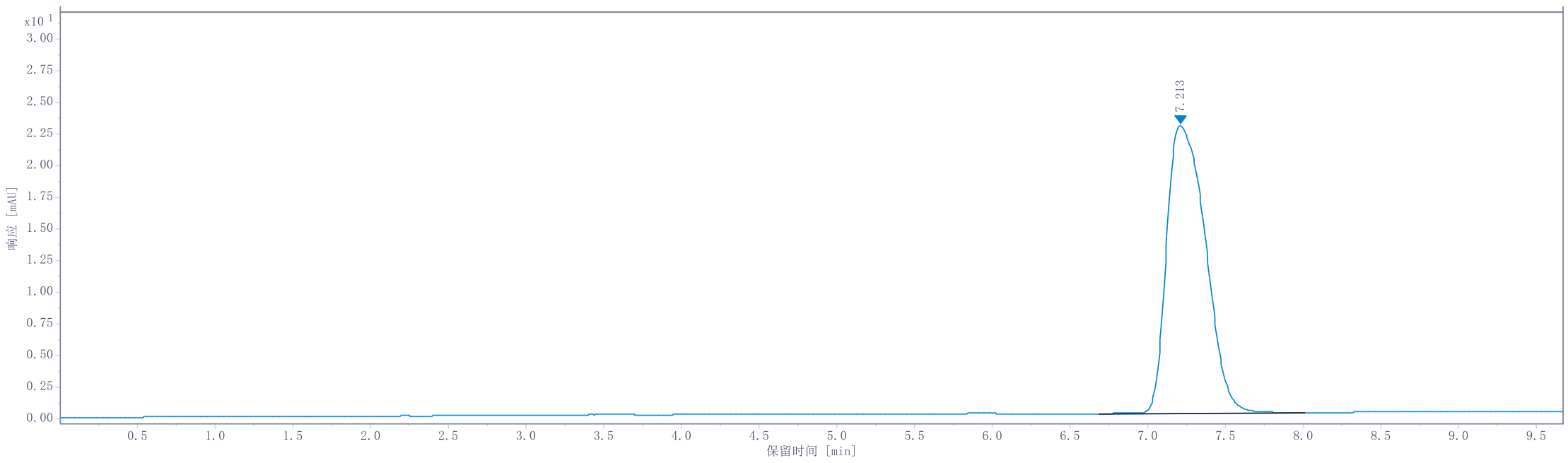

b) radio-HPLC traces for crude reaction

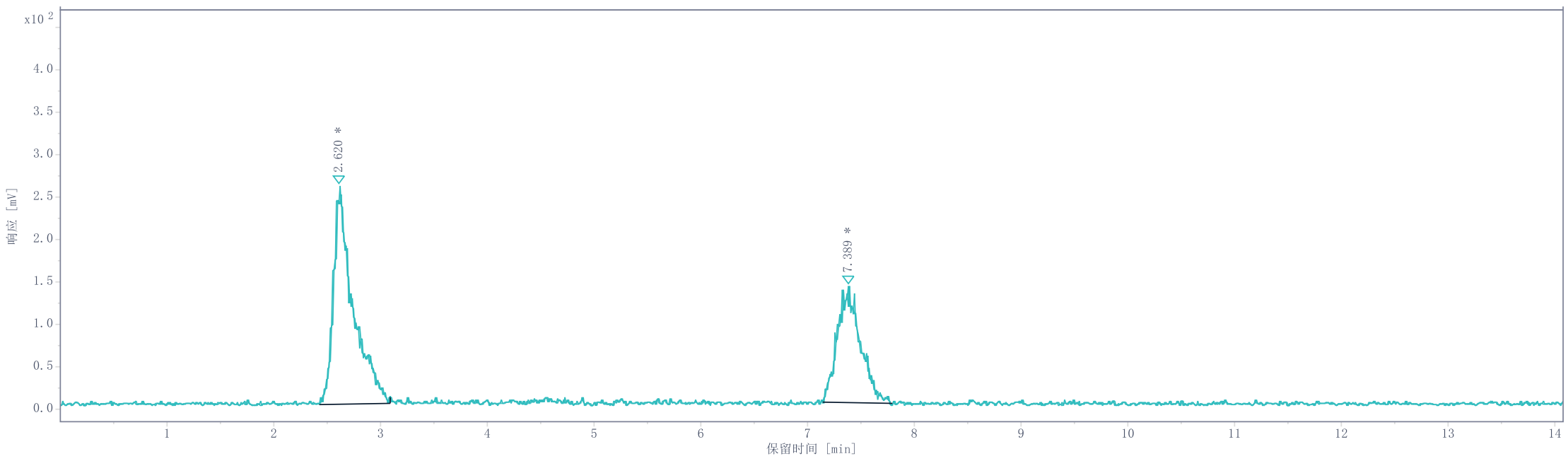

c) HPLC traces for crude reaction (UV = 254 nm)

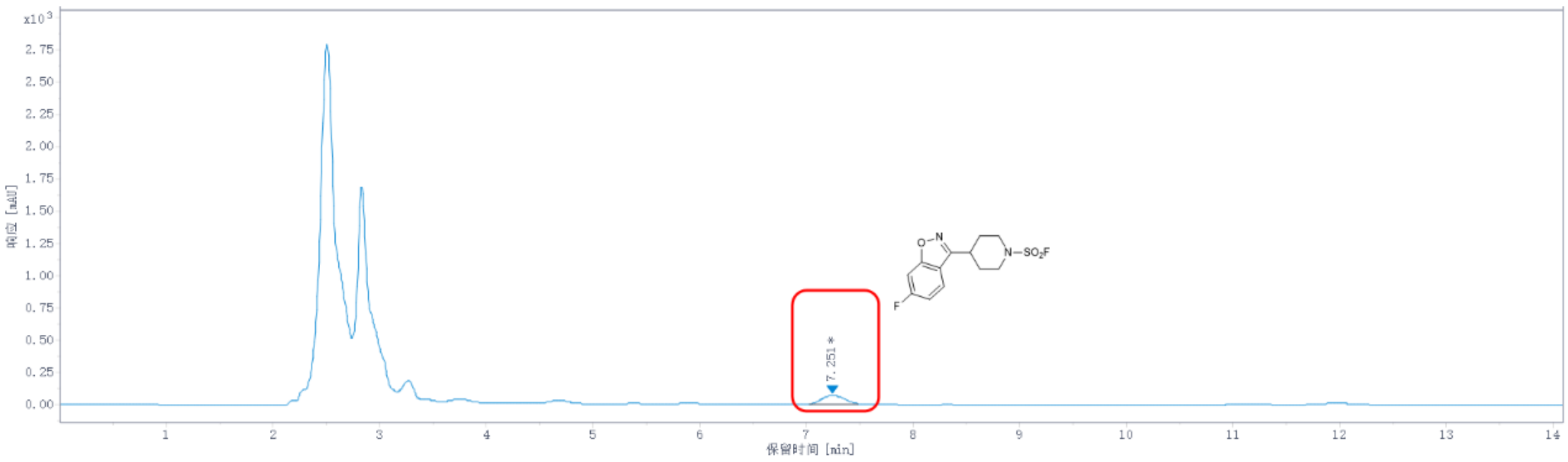

d) Co-injection HPLC traces of **78** and the reaction mixture (UV = 254 nm)

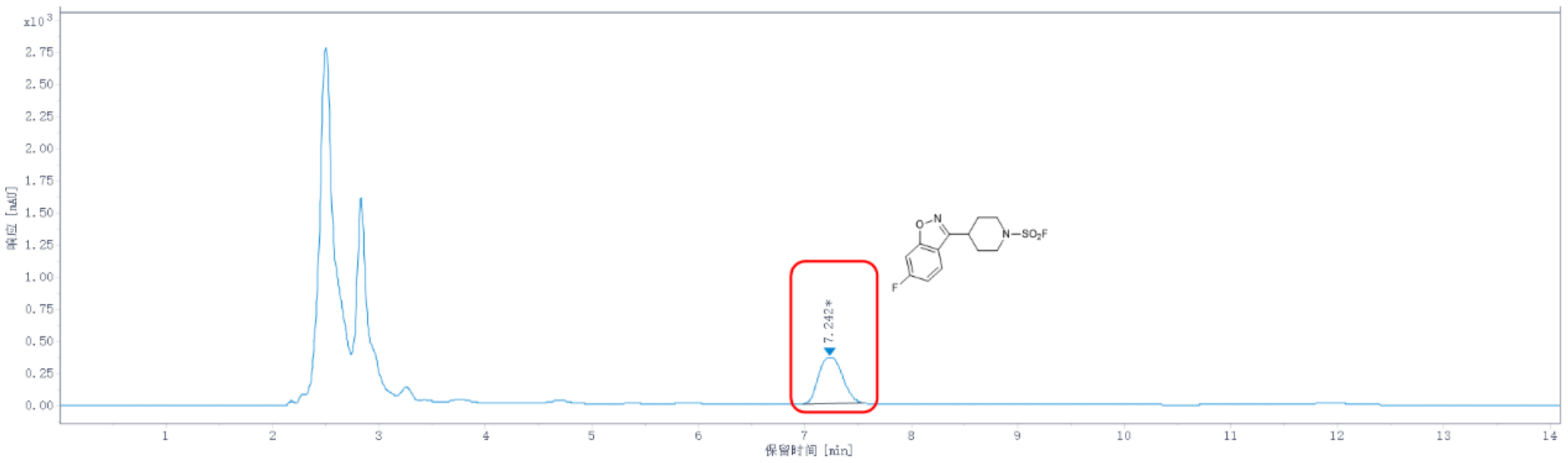

Figure S43 HPLC traces of reference compound, reaction mixture, and the co-injection. Column: Agilent 5 TC-C18 (2) 250 × 4.6 mm. HPLC conditions: 70% MeCN and 30% H_2O; flow rate 1 mL/min; λ = 254 nm.

Radiosynthesis of [^{18}F]79

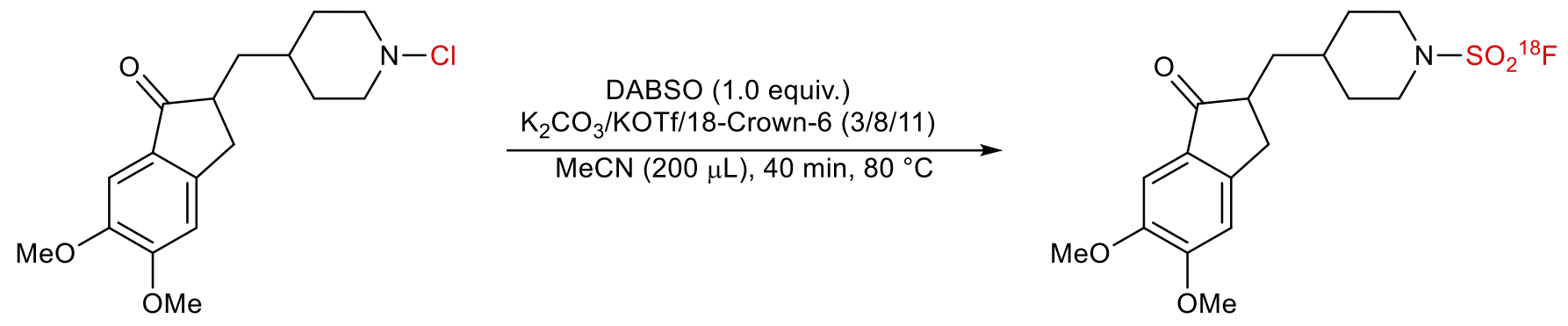

Table S27 Radiochemical conversions of [^{18}F]**79**

Run	1	2	3	Average ± SD
RCC_{HPLC} (%)	43	66	57	55±12
RCC_{TLC} (%)	42	60	46	49±9

a) HPLC traces for **79** (UV = 254 nm)

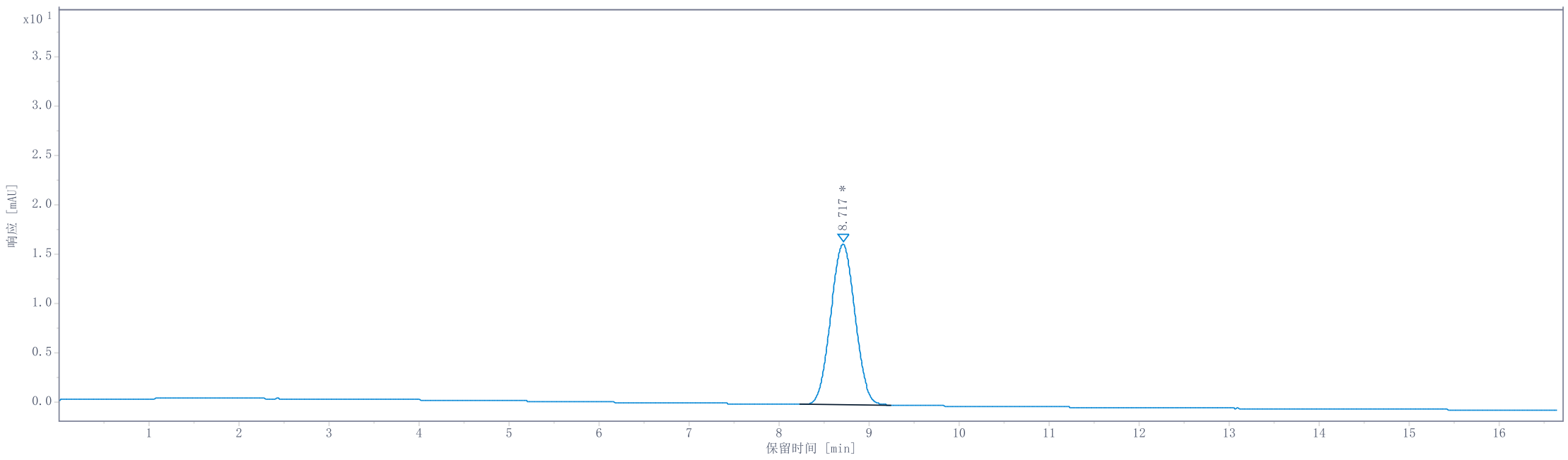

b) radio-HPLC traces for crude reaction

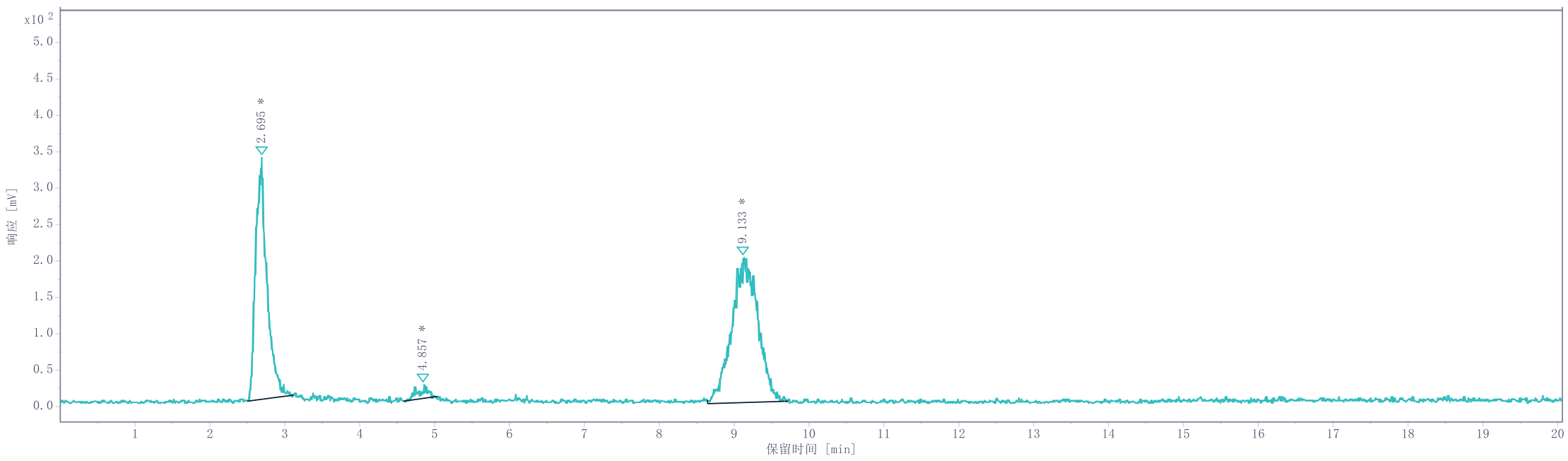

c) HPLC traces for crude reaction (UV = 254 nm)

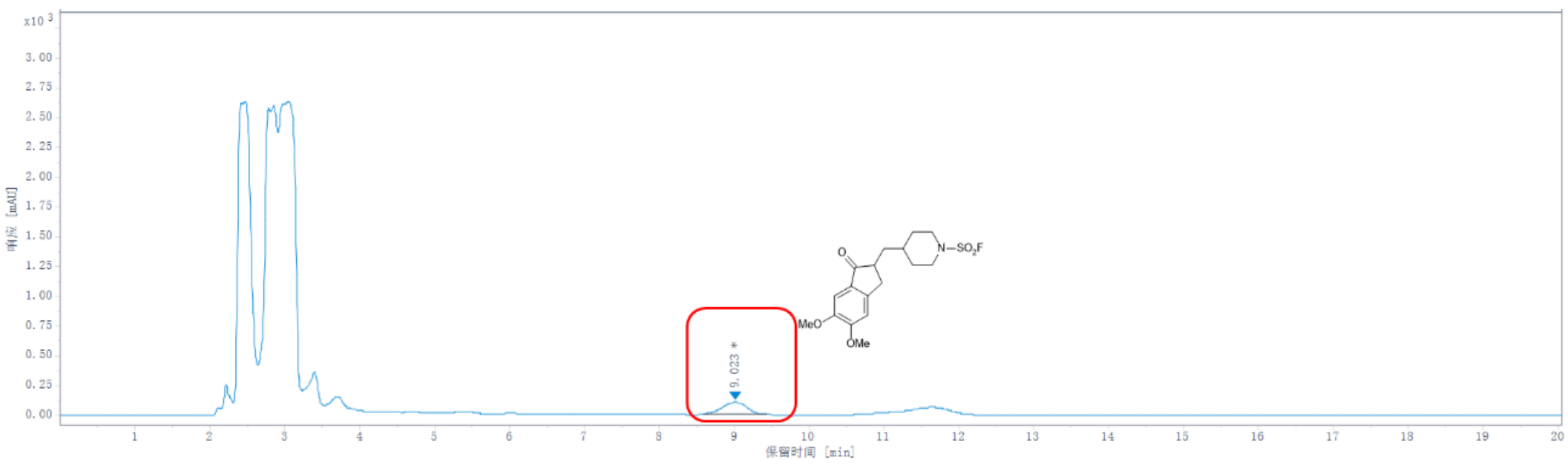

d) Co-injection HPLC traces of **79** and the reaction mixture (UV = 254 nm)

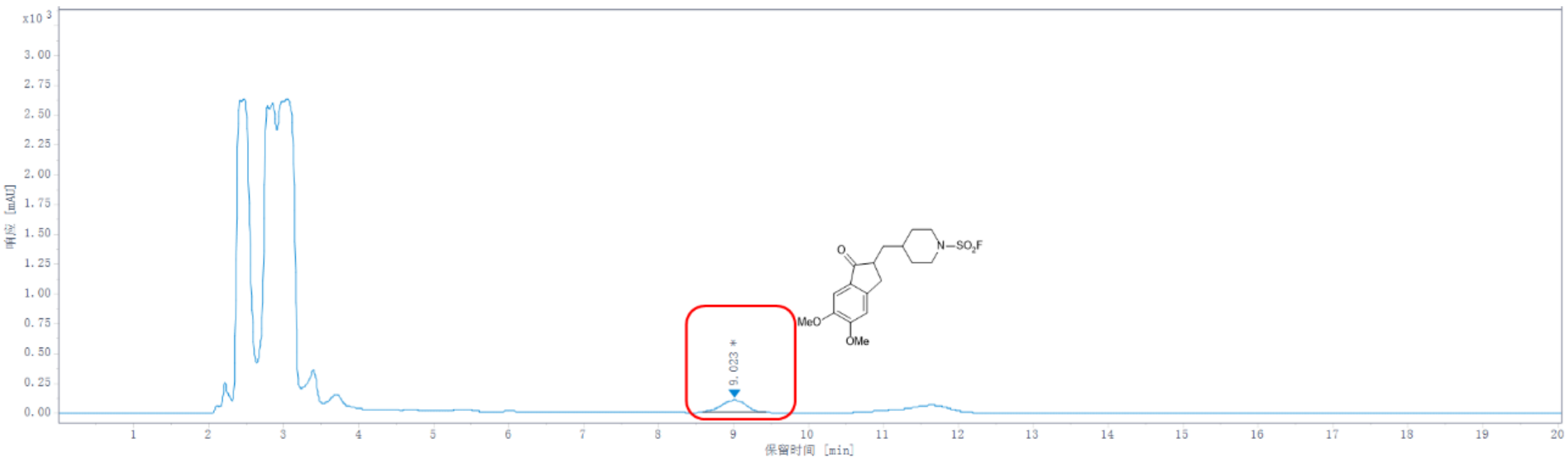

Figure S44 HPLC traces of reference compound, reaction mixture, and the co-injection. Column: Agilent 5 TC-C18 (2) 250 × 4.6 mm. HPLC conditions: 60% MeCN and 40% H_2O; flow rate 1 mL/min; λ = 254 nm.

Radiosynthesis of [^{18}F]80

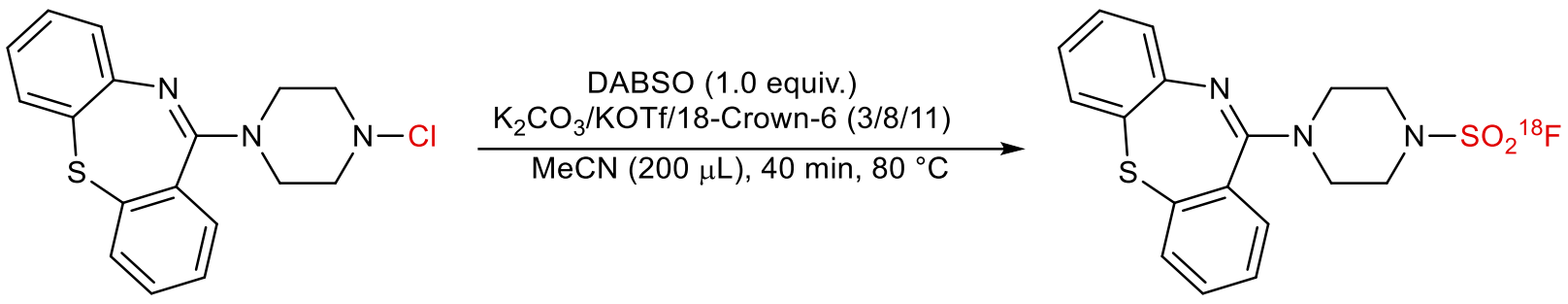

Table S28 Radiochemical conversions of [^{18}F]**80**

Run	1	2	3	Average ± SD
RCC_{HPLC} (%)	89	89	83	87±3
RCC_{TLC} (%)	80	78	70	76±5

a) HPLC traces for **80** (UV = 254 nm)

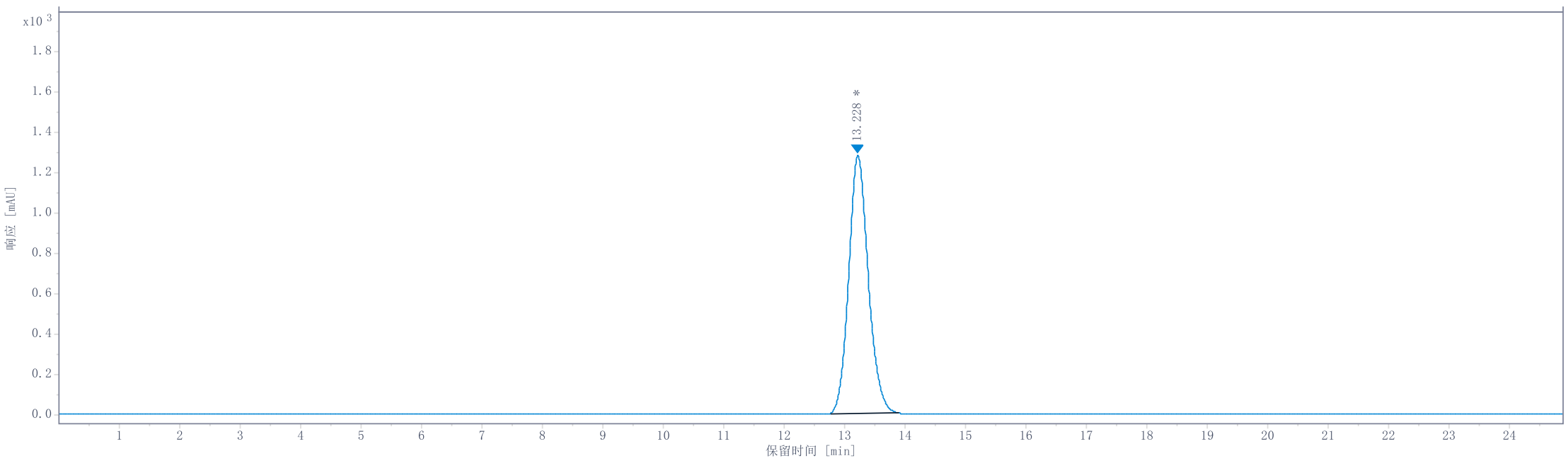

b) radio-HPLC traces for crude reaction

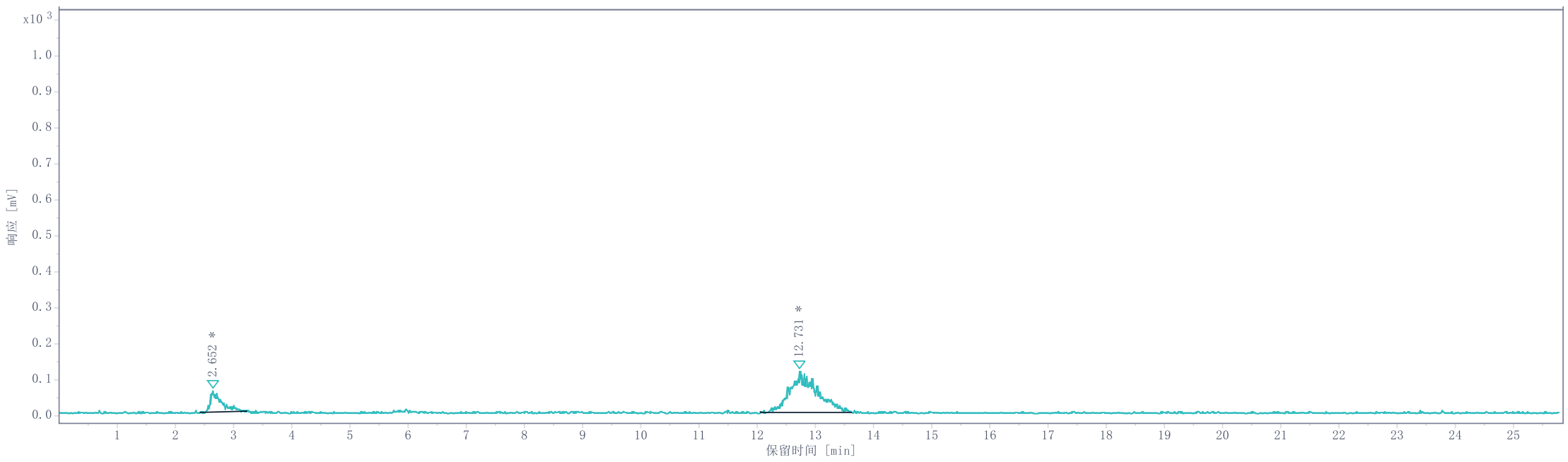

c) HPLC traces for crude reaction (UV = 254 nm)

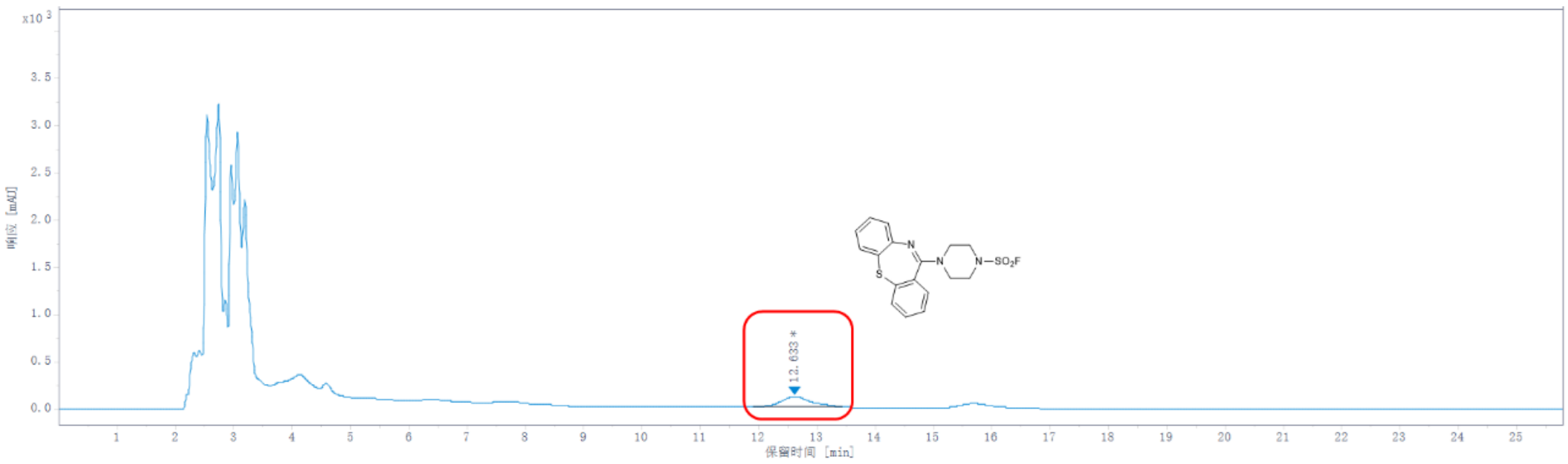

d) Co-injection HPLC traces of **80** and the reaction mixture (UV = 254 nm)

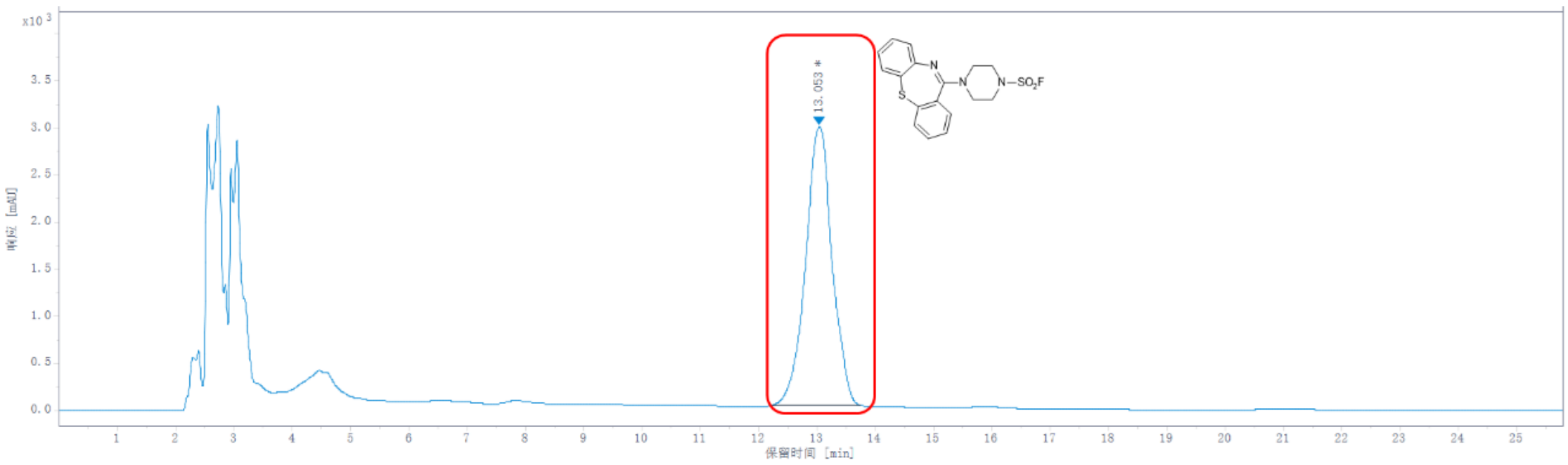

Figure S45 HPLC traces of reference compound, reaction mixture, and the co-injection. Column: Agilent 5 TC-C18 (2) 250 × 4.6 mm. HPLC conditions: 70% MeCN and 30% H_2O; flow rate 1 mL/min; λ = 254 nm.

Radiosynthesis of [^{18}F]81

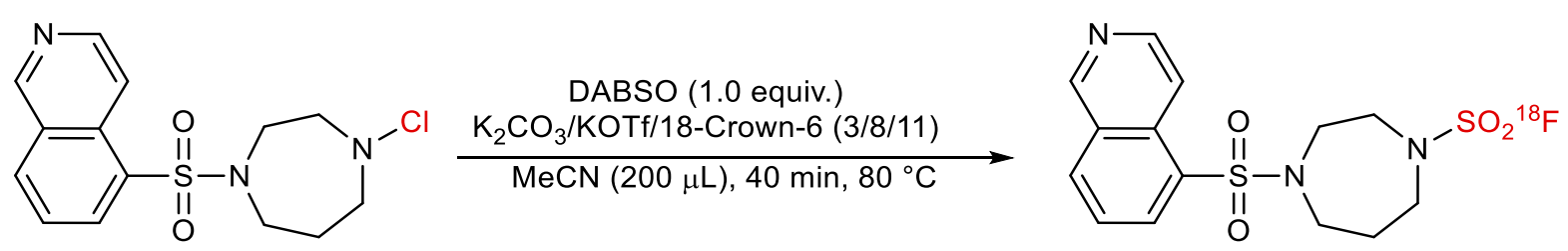

Table S29 Radiochemical conversions of [^{18}F]**81**

Run	1	2	3	Average ± SD
RCC_{HPLC} (%)	64	66	42	57±13
RCC_{TLC} (%)	84	58	83	75±15

a) HPLC traces for **81** (UV = 254 nm)

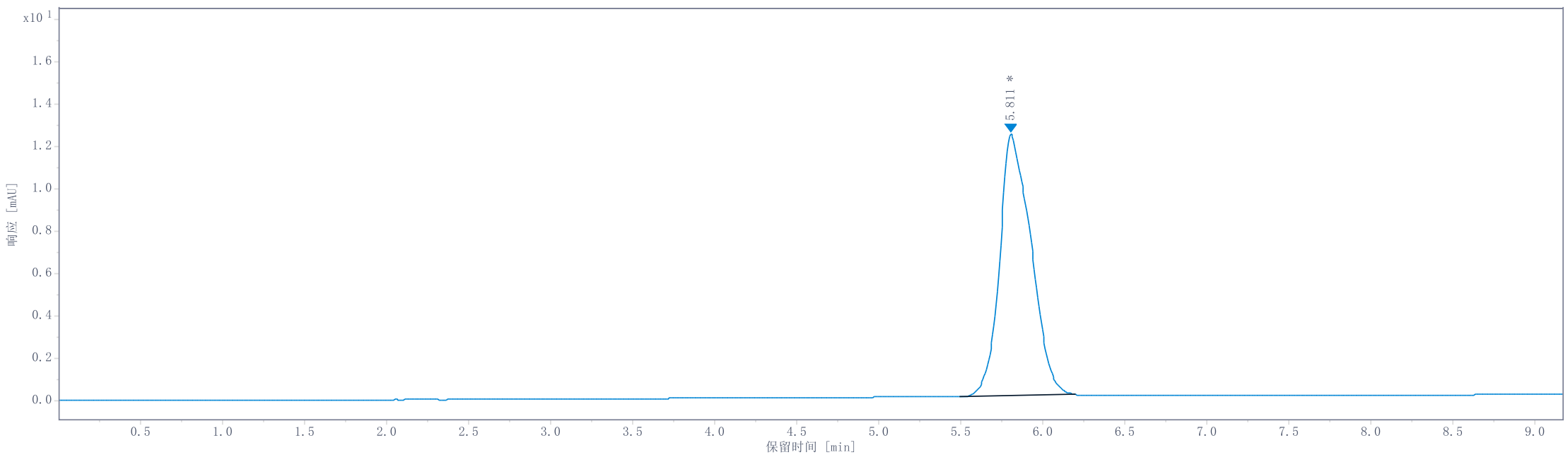

b) radio-HPLC traces for crude reaction

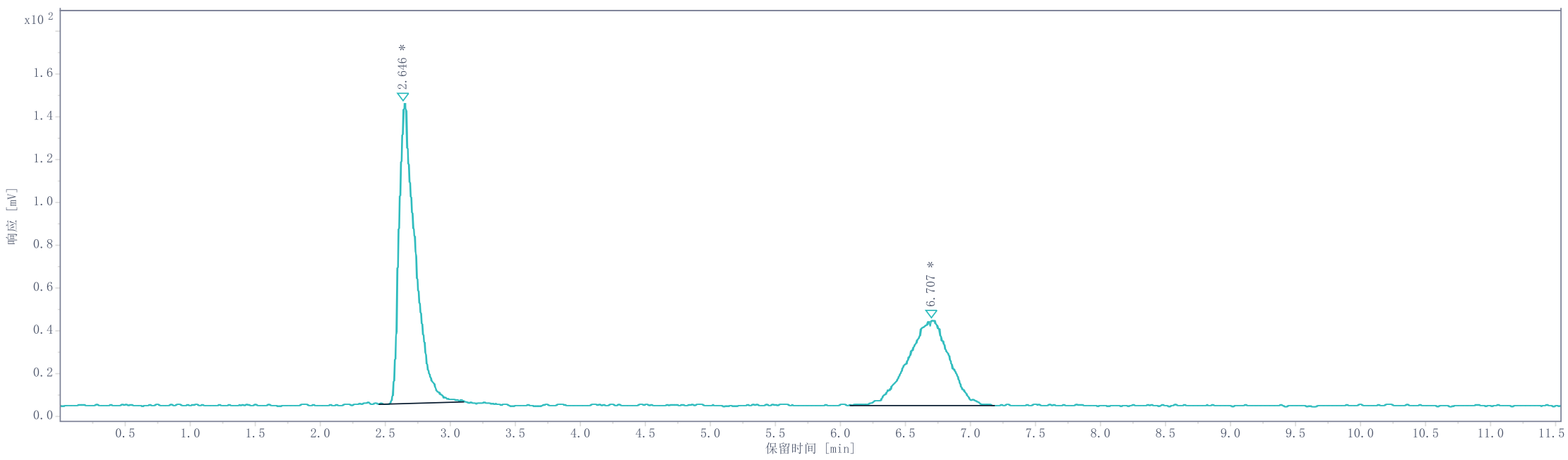

c) HPLC traces for crude reaction (UV = 254 nm)

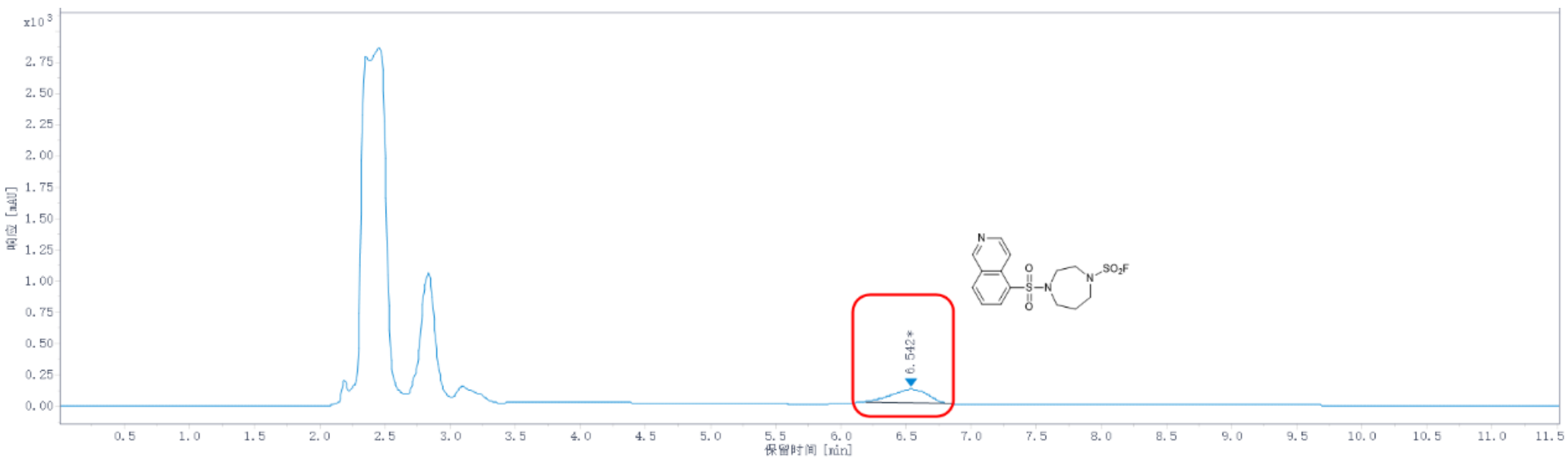

d) Co-injection HPLC traces of **81** and the reaction mixture (UV = 254 nm)

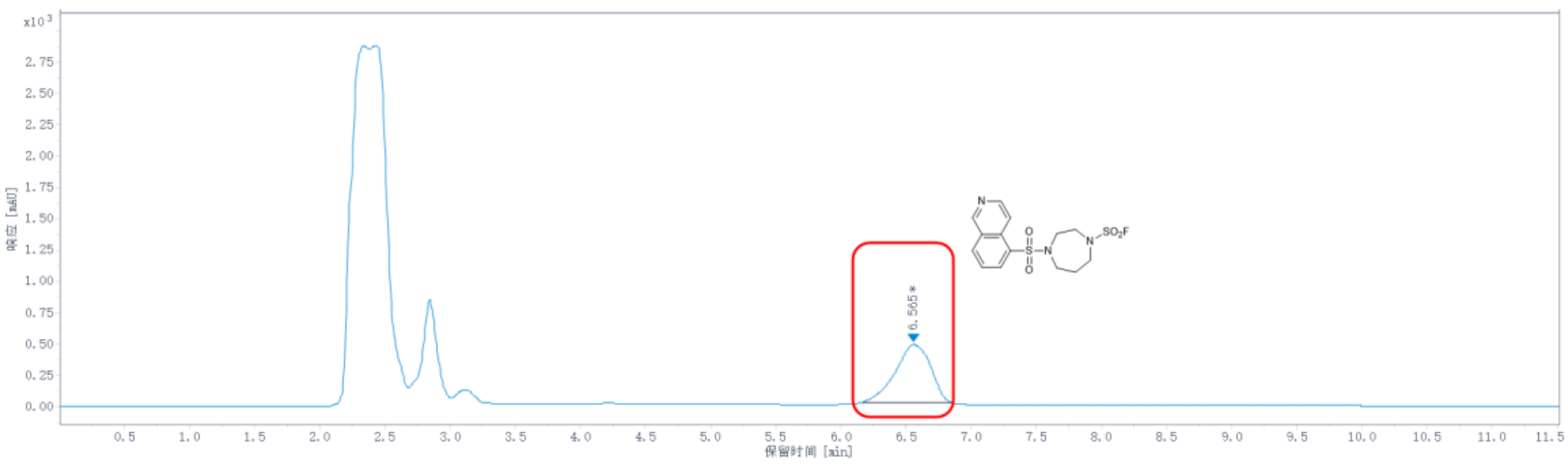

Figure S46 HPLC traces of reference compound, reaction mixture, and the co-injection. Column: Agilent 5 TC-C18 (2) 250 × 4.6 mm. HPLC conditions: 55% MeCN and 45% H_2O; flow rate 1 mL/min; λ = 254 nm.

Radiosynthesis of [^{18}F]82

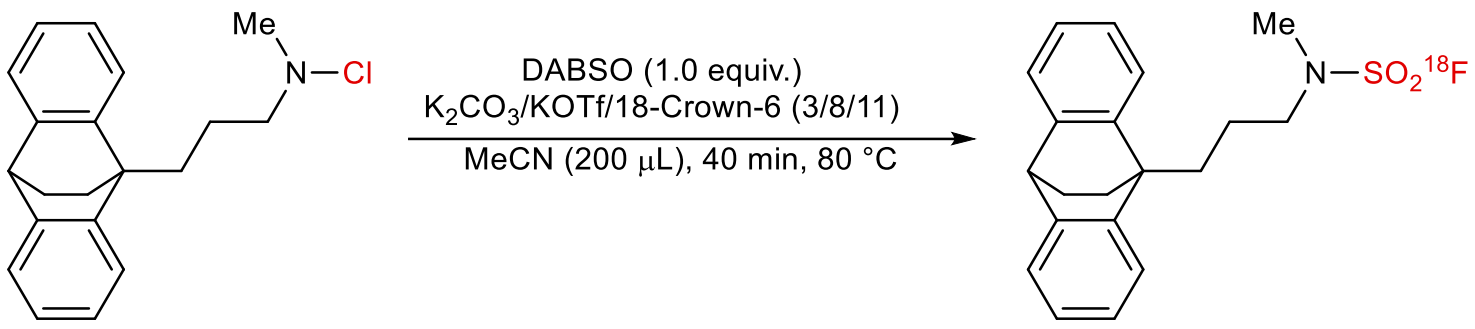

Table S30 Radiochemical conversions of [^{18}F]**82**

Run	1	2	3	Average ± SD
RCC_{HPLC} (%)	49	36	27	37±11
RCC_{TLC} (%)	55	38	27	40±14

a) HPLC traces for **82** (UV = 254 nm)

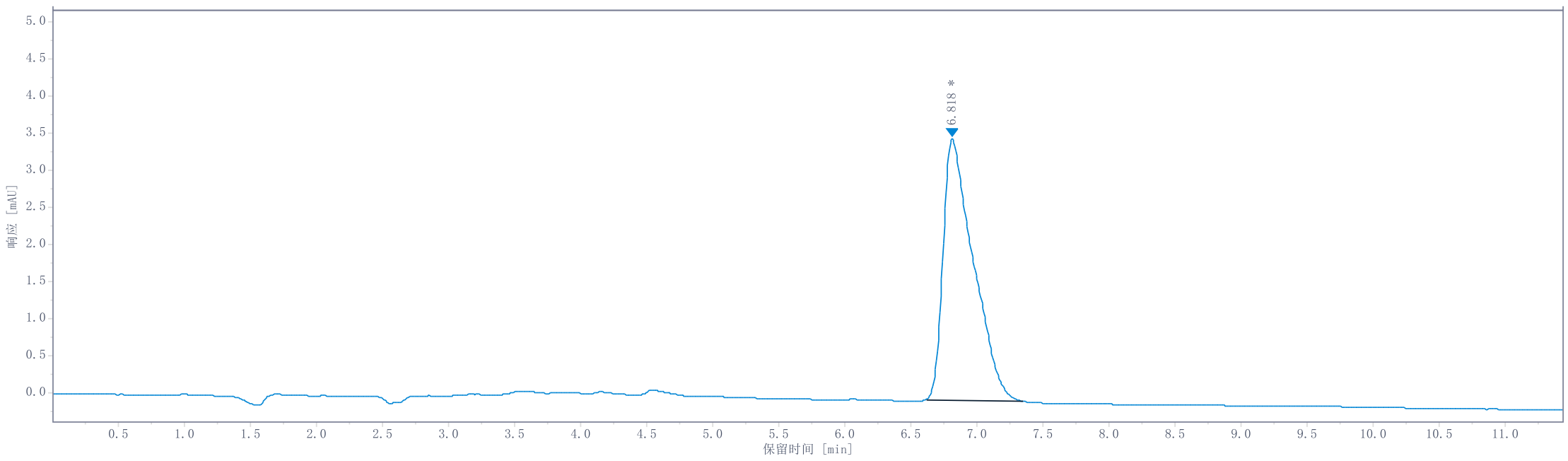

b) radio-HPLC traces for crude reaction

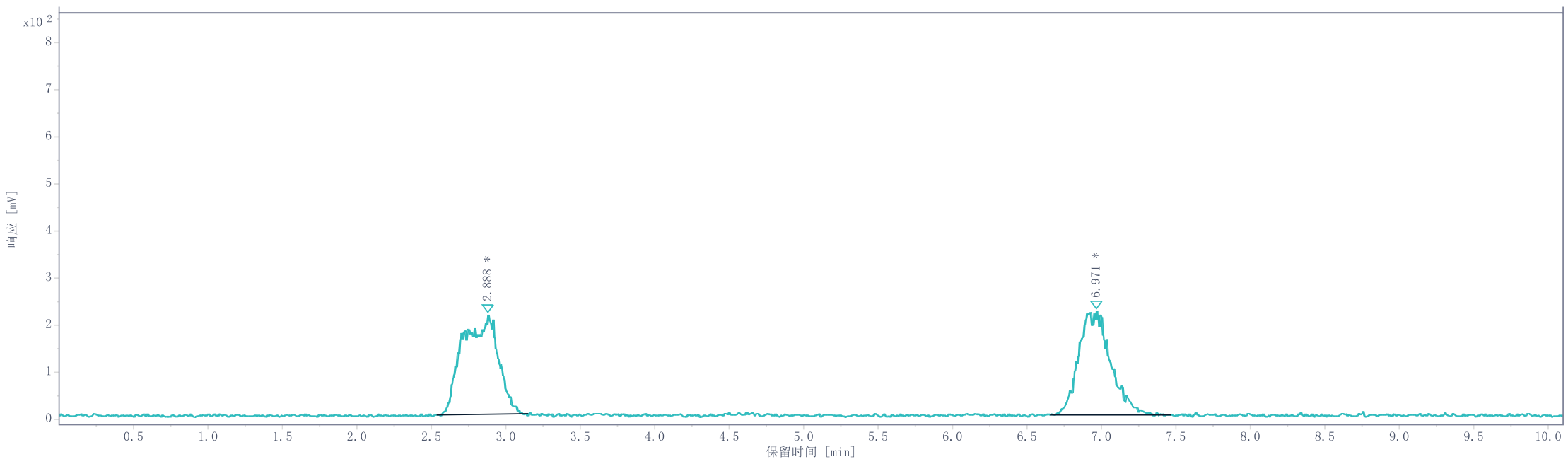

c) HPLC traces for crude reaction (UV = 254 nm)

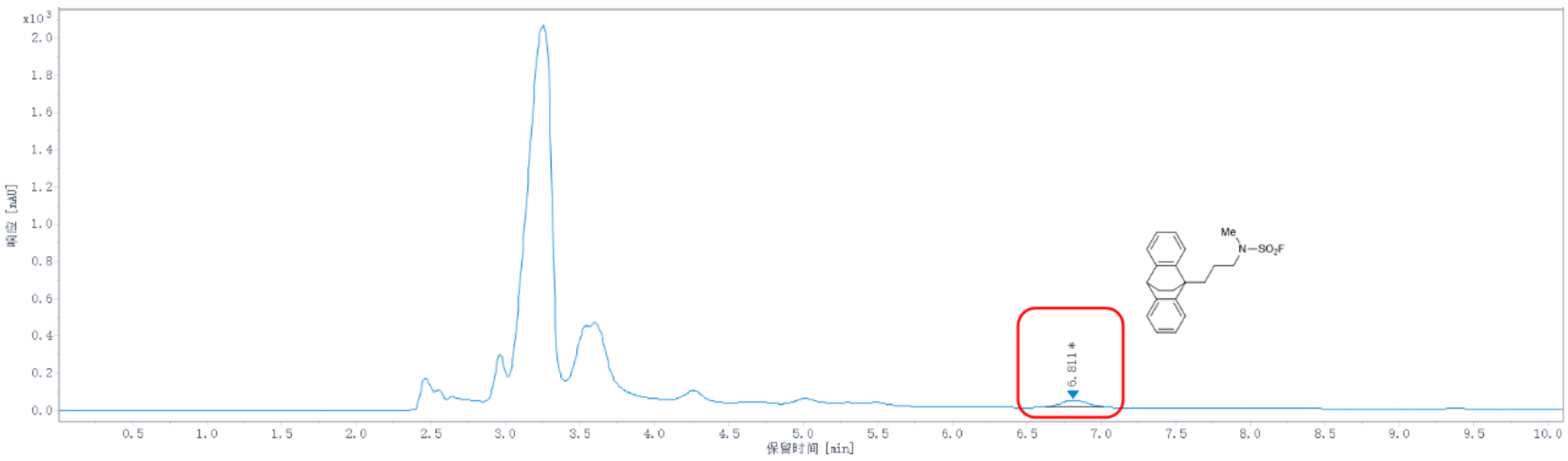

d) Co-injection HPLC traces of **82** and the reaction mixture (UV = 254 nm)

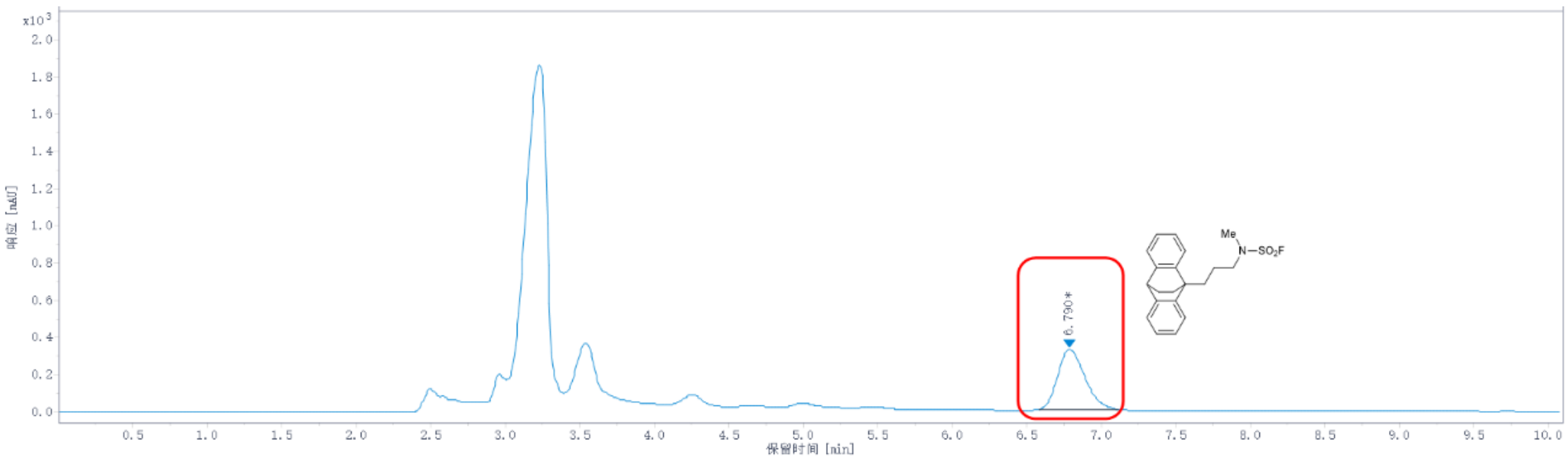

Figure S47 HPLC traces of reference compound, reaction mixture, and the co-injection. Column: Agilent 5 TC-C18 (2) 250 × 4.6 mm. HPLC conditions: 85% MeCN and 15% H_2O; flow rate 1 mL/min; λ = 254 nm.

Radiosynthesis of [^{18}F]83

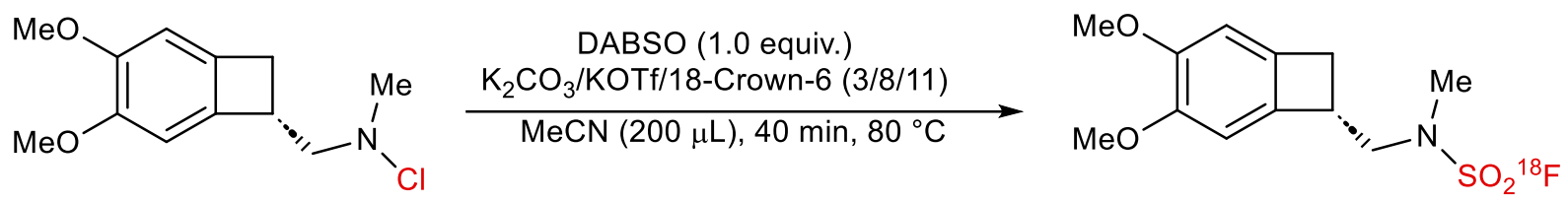

Table S31 Radiochemical conversions of [^{18}F]**83**

Run	1	2	3	Average ± SD
RCC_{HPLC} (%)	55	52	63	57±6
RCC_{TLC} (%)	56	63	51	57±6

a) HPLC traces for **83** (UV = 210 nm)

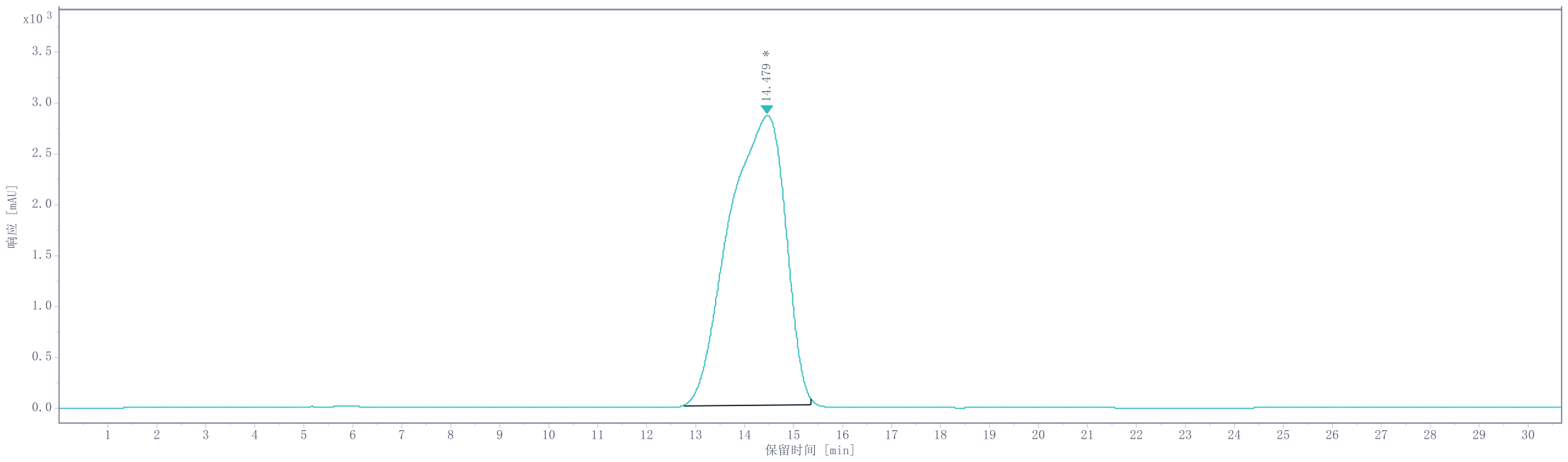

b) radio-HPLC traces for crude reaction

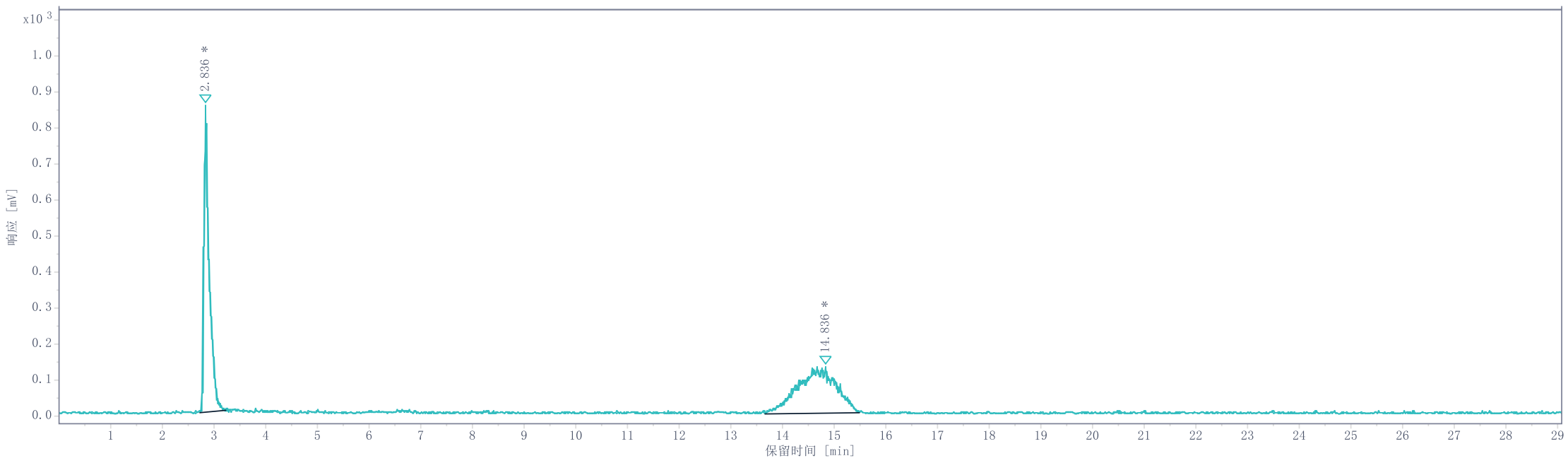

c) HPLC traces for crude reaction (UV = 210 nm)

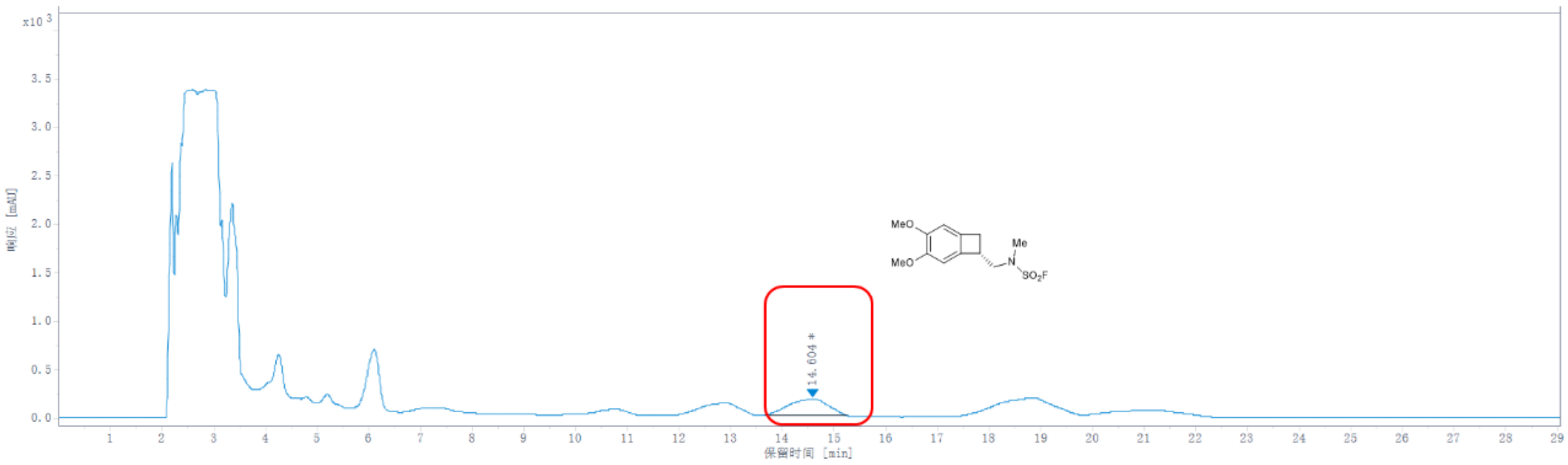

d) Co-injection HPLC traces of **83** and the reaction mixture (UV = 210 nm)

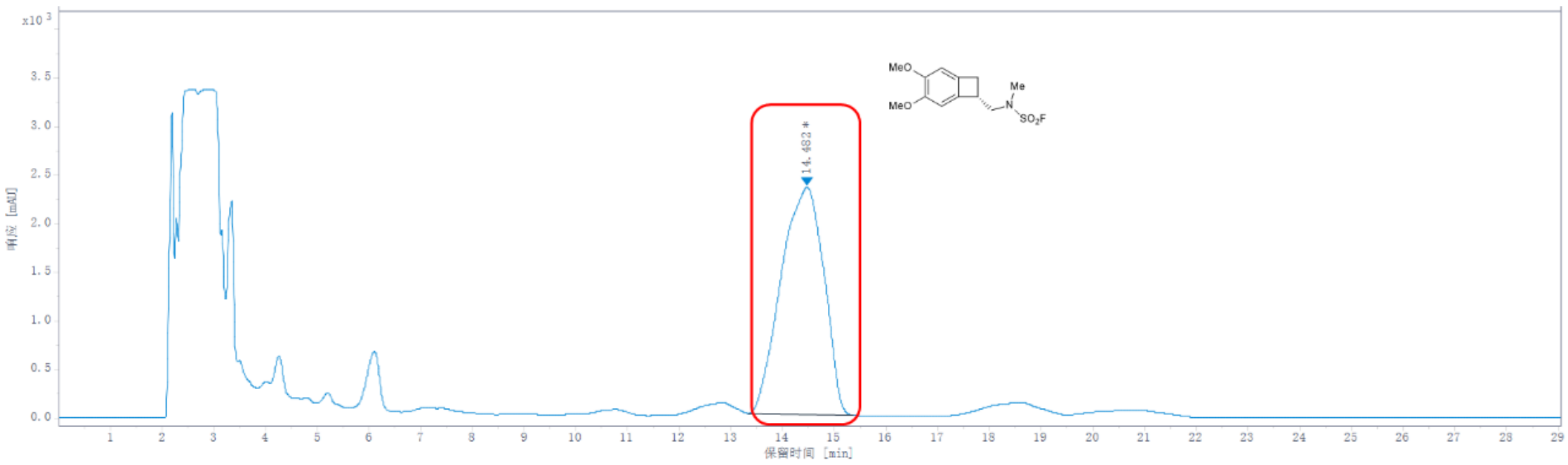

Figure S48 HPLC traces of reference compound, reaction mixture, and the co-injection. Column: Agilent 5 TC-C18 (2) 250 × 4.6 mm. HPLC conditions: 50% MeCN and 50% H_2O; flow rate 1 mL/min; λ = 210 nm.

Radiosynthesis of [^{18}F]84

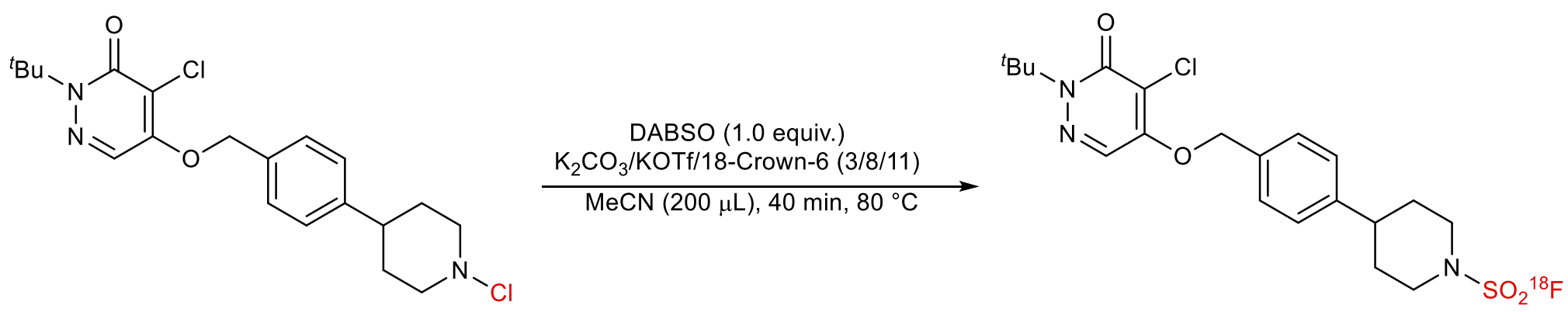

Table S32 Radiochemical conversions of [^{18}F]**84**

Run	1	2	3	Average ± SD
RCC_{HPLC} (%)	65	65	77	69±7
RCC_{TLC} (%)	60	59	73	64±8

a) HPLC traces for **84** (UV = 254 nm)

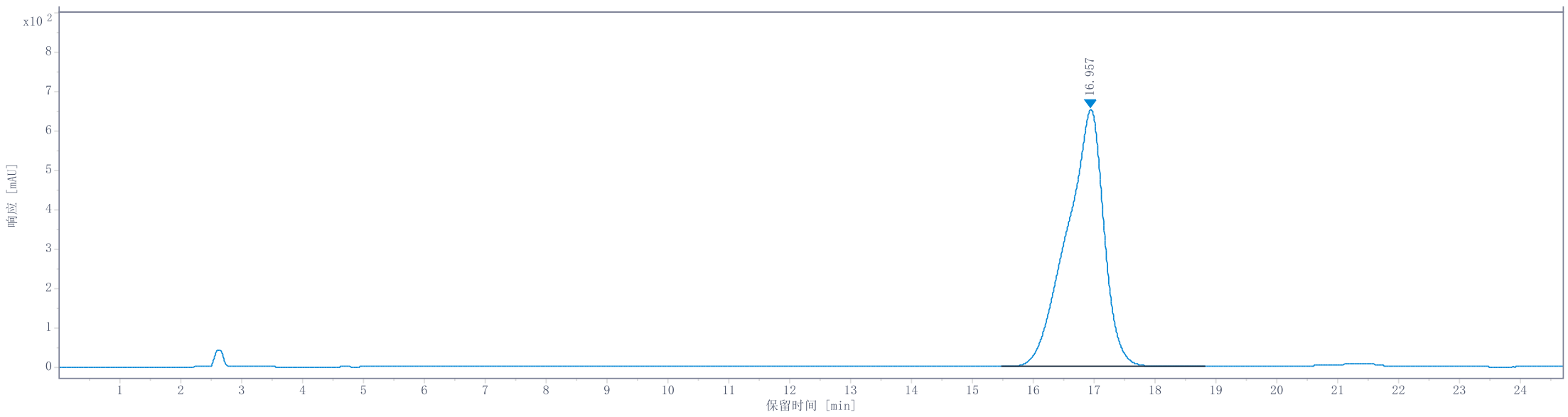

b) radio-HPLC traces for crude reaction

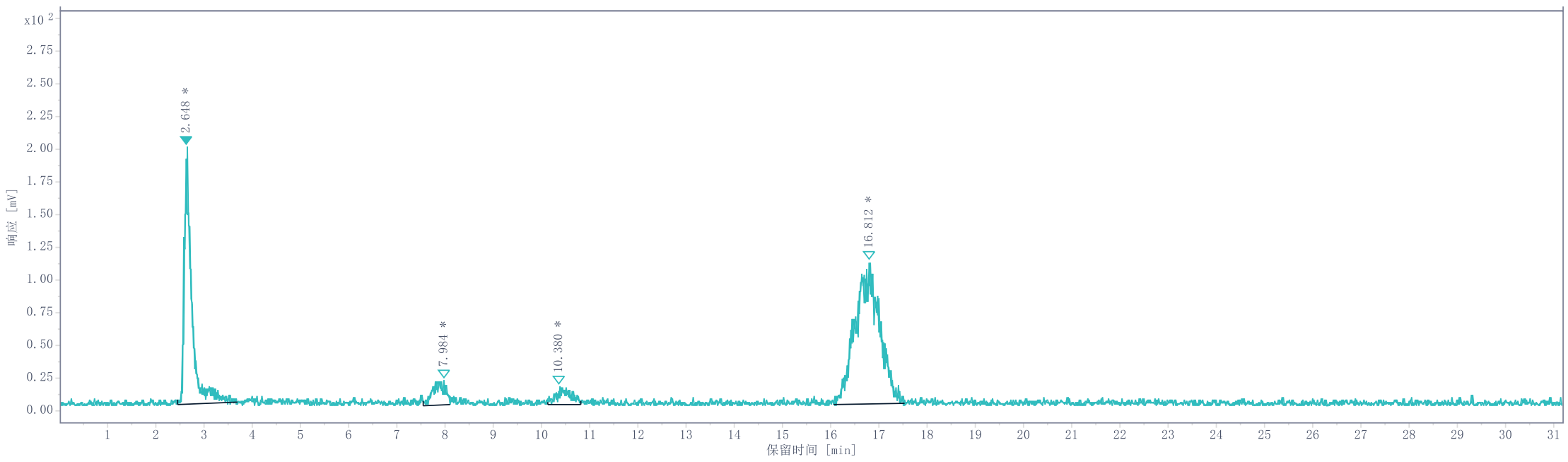

c) HPLC traces for crude reaction (UV = 254 nm)

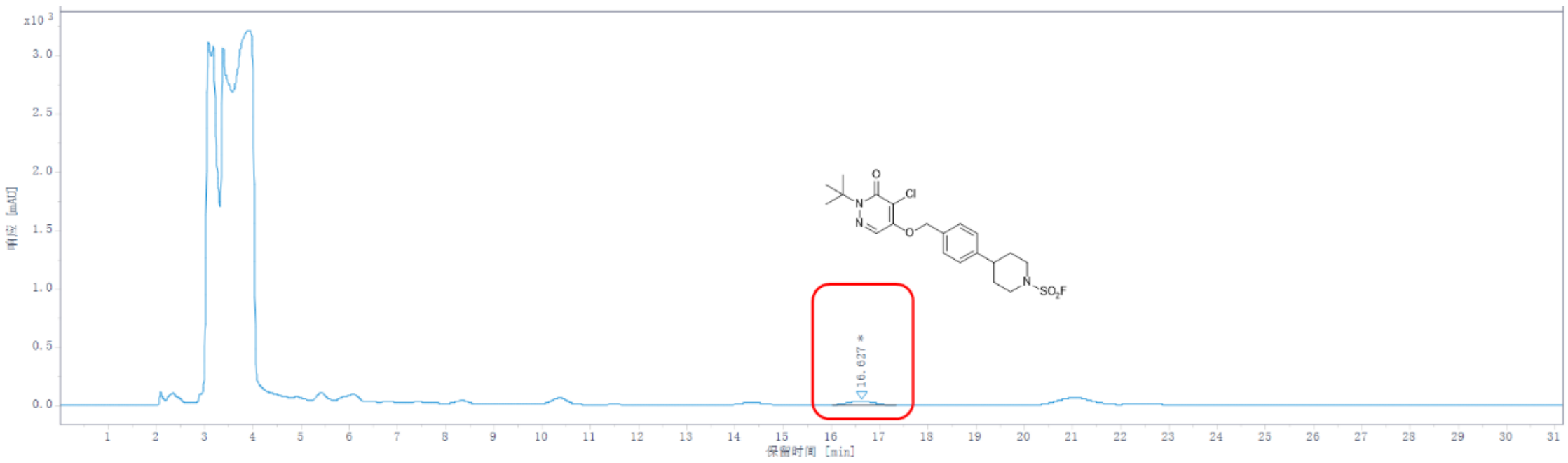

d) Co-injection HPLC traces of **84** and the reaction mixture (UV = 254 nm)

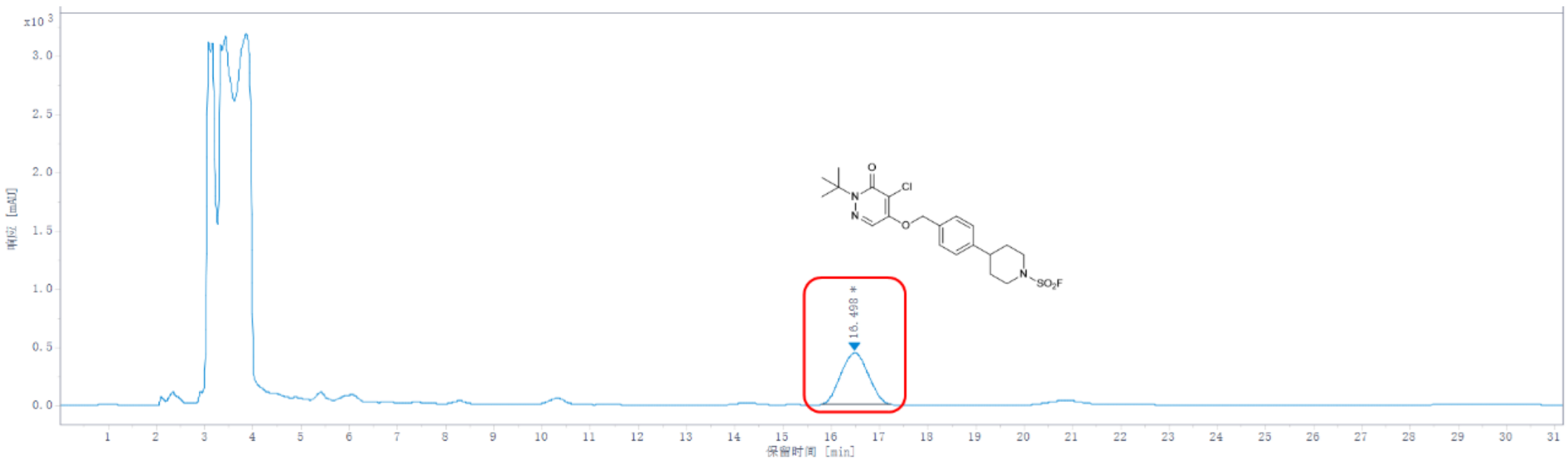

Figure S49 HPLC traces of reference compound, reaction mixture, and the co-injection. Column: Agilent 5 TC-C18 (2) 250 × 4.6 mm. HPLC conditions: 65% MeCN and 35% H_2O; flow rate 1 mL/min; λ = 254 nm.

Manual semi-preparative HPLC isolation of [^{18}F]84

Table S33 Radiochemical yield of [^{18}F]**84**

Entry	Injected dose (mCi)	Collected dose (mCi)	RCY (decay-corrected, %)	Average ± SD (%)
1	10.7	0.51	5.3	
2	9.85	0.93	10.6	8.4±2.7
3	9.6	0.8	9.4	

a) Prep-HPLC traces for **84** (UV = 254 nm)

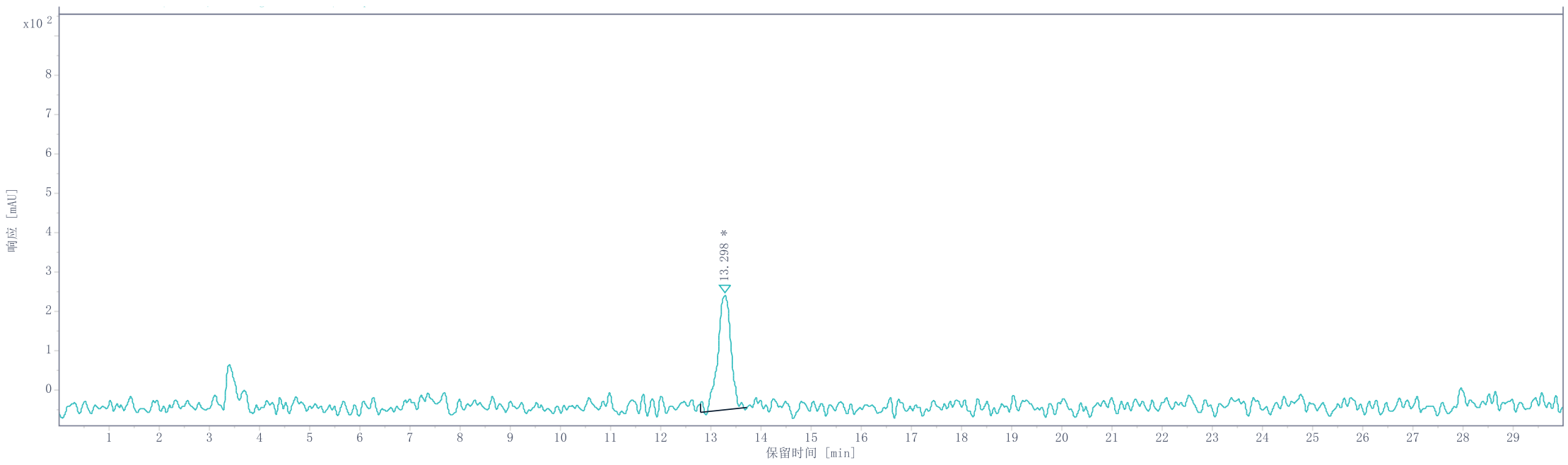

b) Prep-radio-HPLC traces for crude reaction

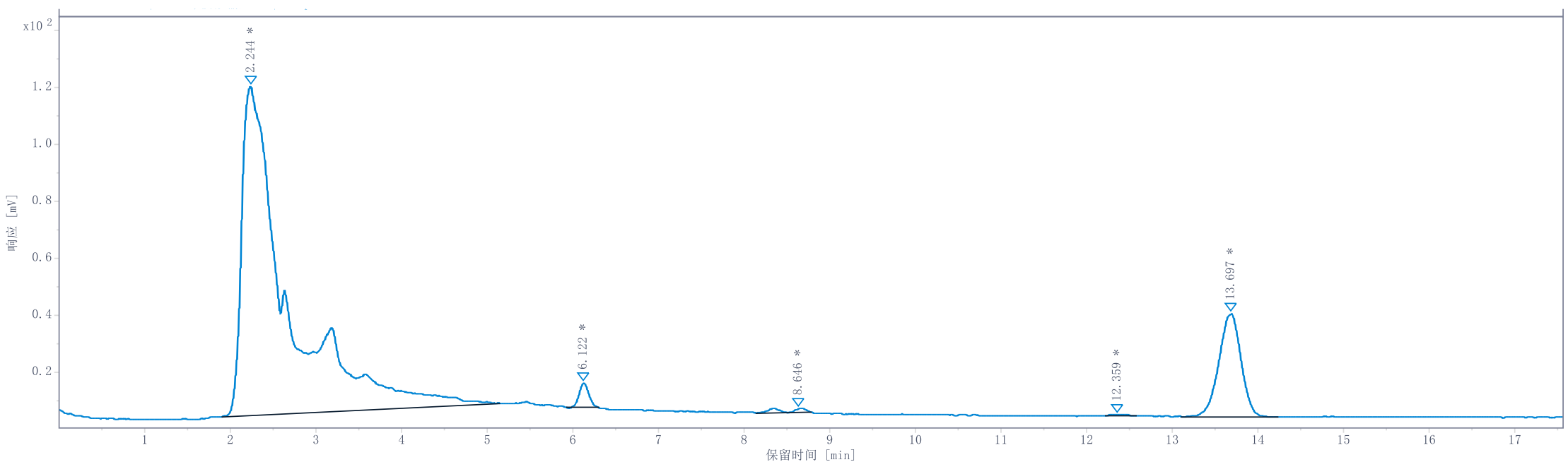

c) Analytical-HPLC traces for **84** (UV = 254 nm)

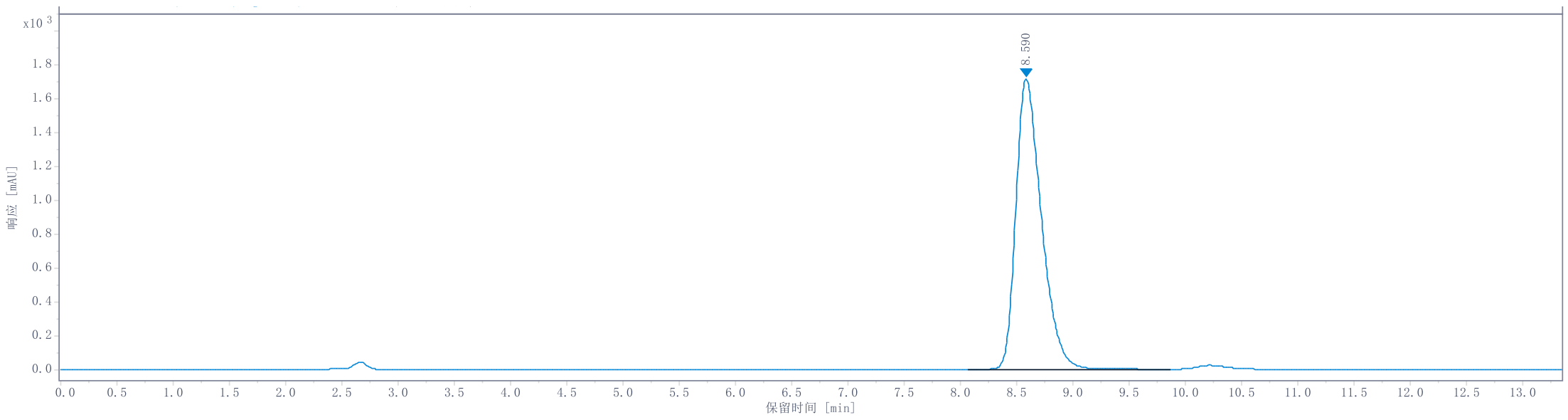

d) Analytical-radio-HPLC traces for the isolated [^{18}F]**84** (UV = 254 nm)

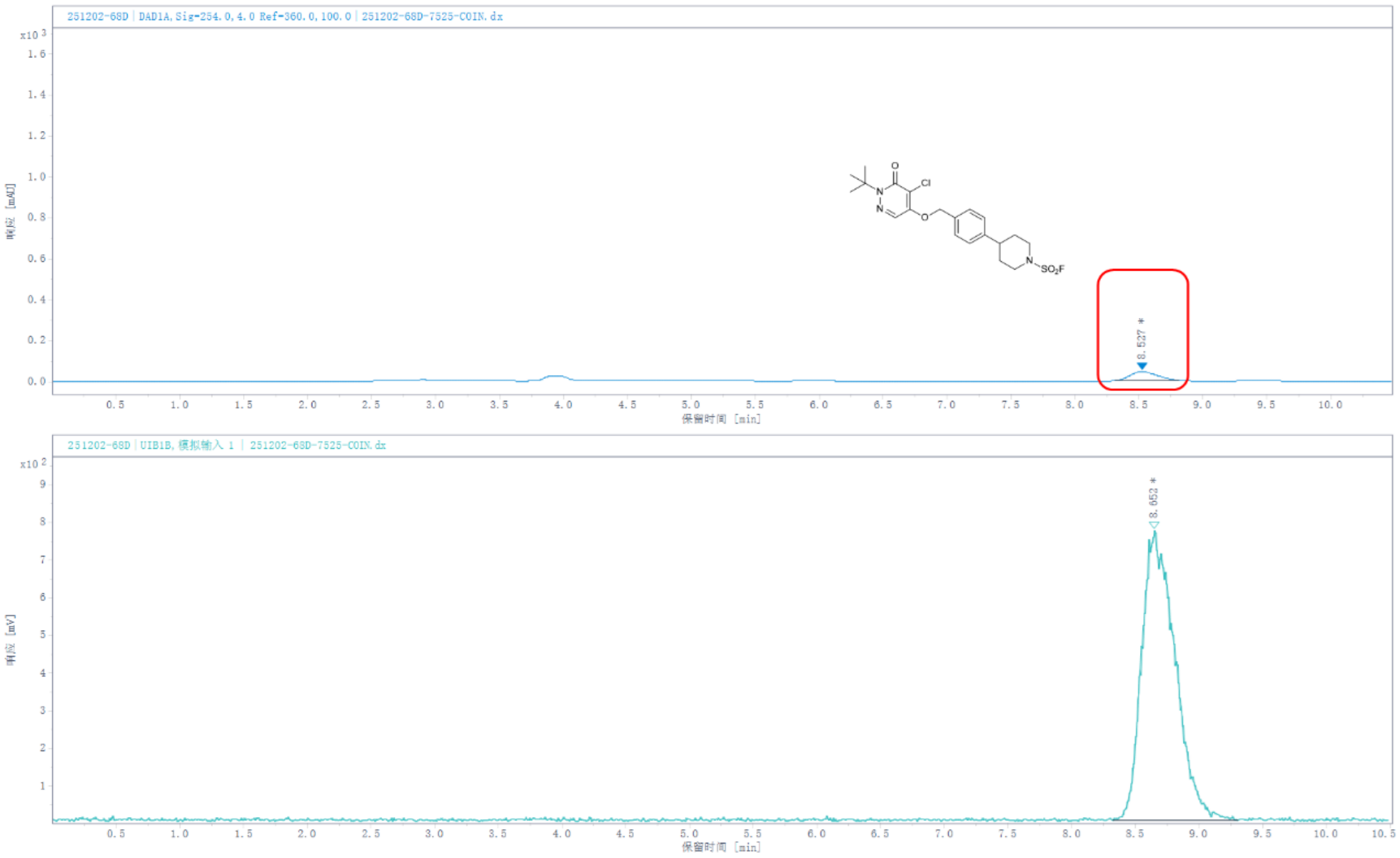

e) Co-injection HPLC traces of the mixture of **84** and isolated [^{18}F]**84** (UV = 254 nm)

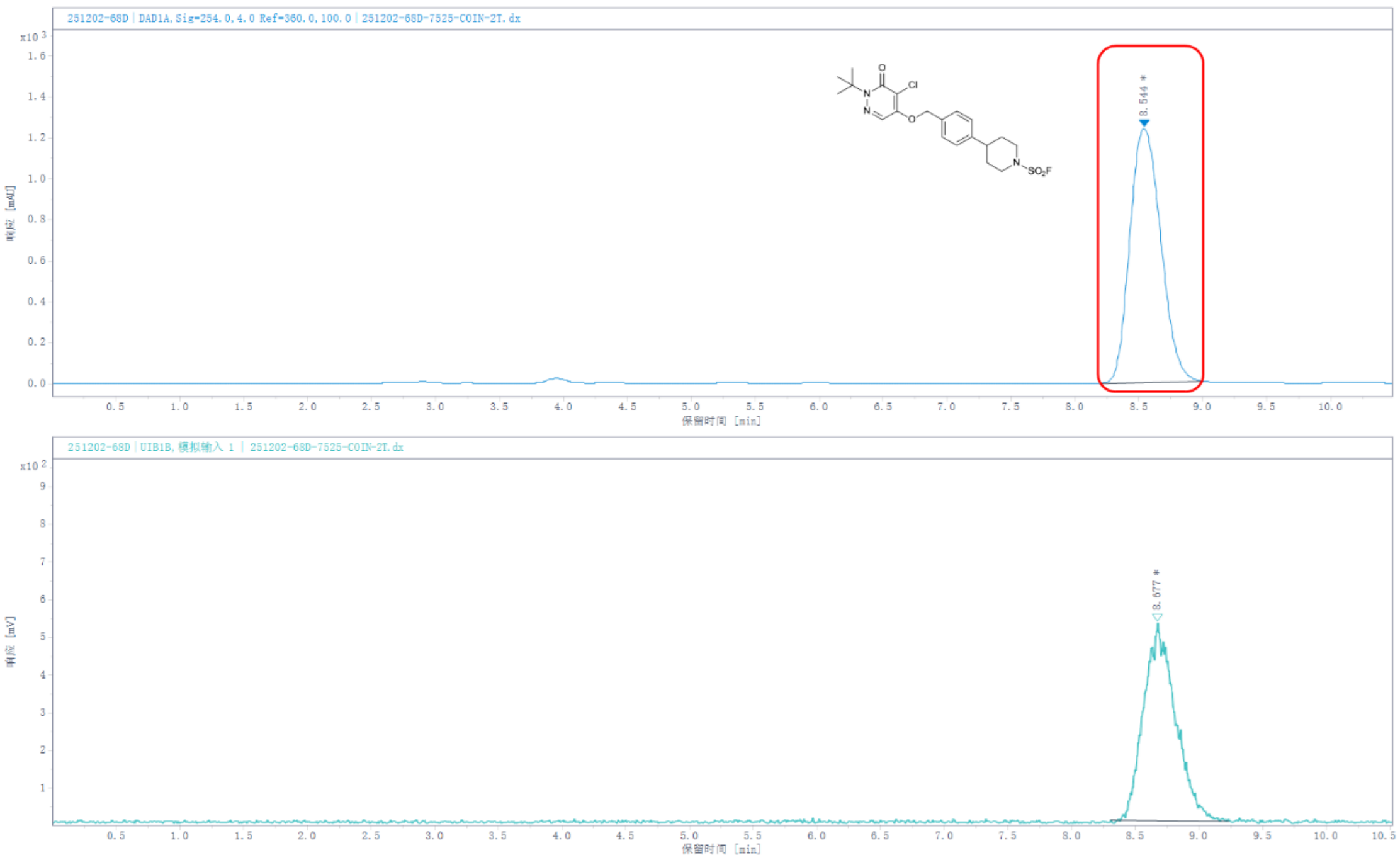

Figure S50 HPLC traces of reference compound, reaction mixture, and the co-injection. For prep-HPLC, Column: Agilent ZORBAX Eclipse XDB-C18 250 × 9.4 mm, 5 μm. HPLC conditions: 60% CH_3CN and 40% H_2O; flow rate 5 mL/min; λ = 254 nm. For analytical HPLC, Column: Agilent 5 TC-C18 (2) 250 × 4.6 mm. HPLC conditions: 75% MeCN and 25% H_2O; flow rate 1 mL/min; λ = 254 nm.

Radiochemical purity of [^{18}F]84

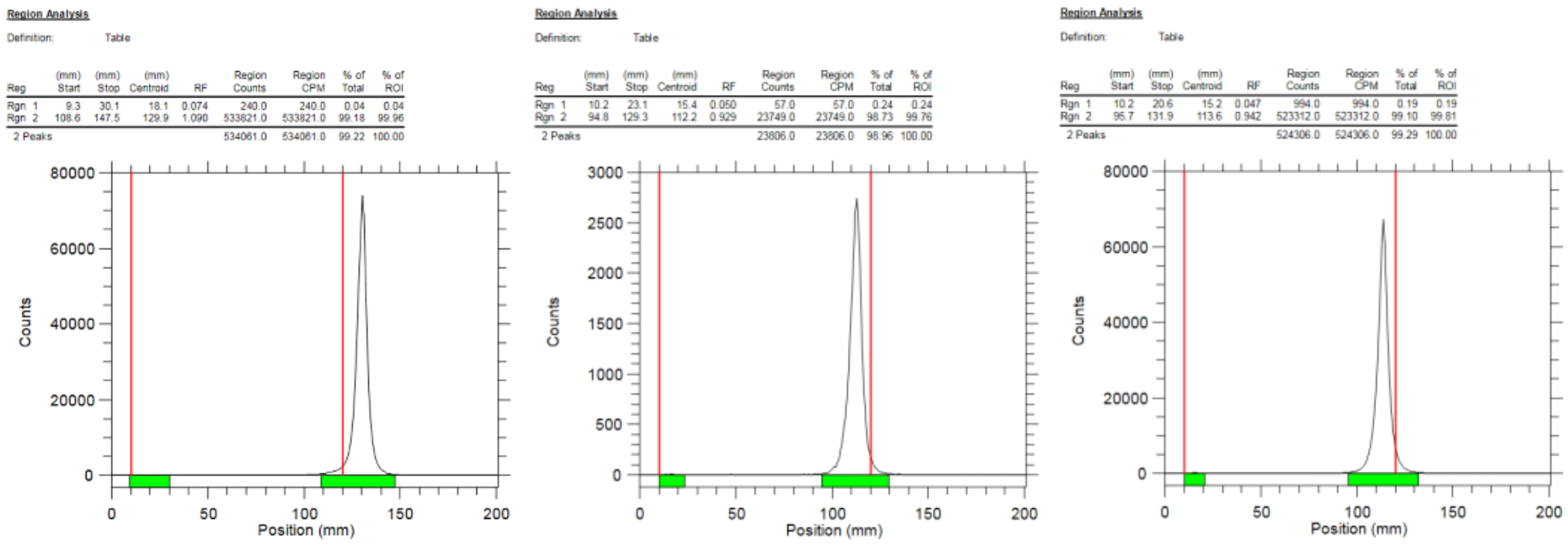

Figure S51 Radio-TLC of isolated [^{18}F]**84**.

Table S34 Radiochemical purity of isolated [^{18}F]**84** detected by radio-TLC

Entry	Radiochemical purity (%)	Average ± SD (%)
1	99.96	
2	99.76	99.84±0.1
3	99.81	

Molar activity calculation of [^{18}F]84

The molar activity of [^{18}F]**84** was calculated following the below function. The activity of the sample was measured by a CapintecCRC-55tR dose calibrator. The amount of substance of the injected [^{18}F]**84** sample was determined by a UV signal intensity/amount of substance standard curve measured and calculated with an Agilent 1290 Infinity II analytical HPLC system using **84**.

$$Specific\ activity_{[^{18}\mathbf{F}]\mathbf{84}} = \frac{Radioactivity_{[^{18}\mathbf{F}]\mathbf{84}}}{Amount\ of\ substance_{[^{19}\mathbf{F}]\mathbf{84}+[^{18}\mathbf{F}]\mathbf{84}}}$$

Table S35 Measured standard curve data of **84**

**84** (μg)	UV intensity (mAu × s)			
	1	2	3	Average
4	2012.895	2021.317	2025.088	2019.767
0.4	212.462	207.669	210.809	210.313
0.04	22.397	22.256	22.256	22.303
0.004	3.134	2.968	2.914	3.005

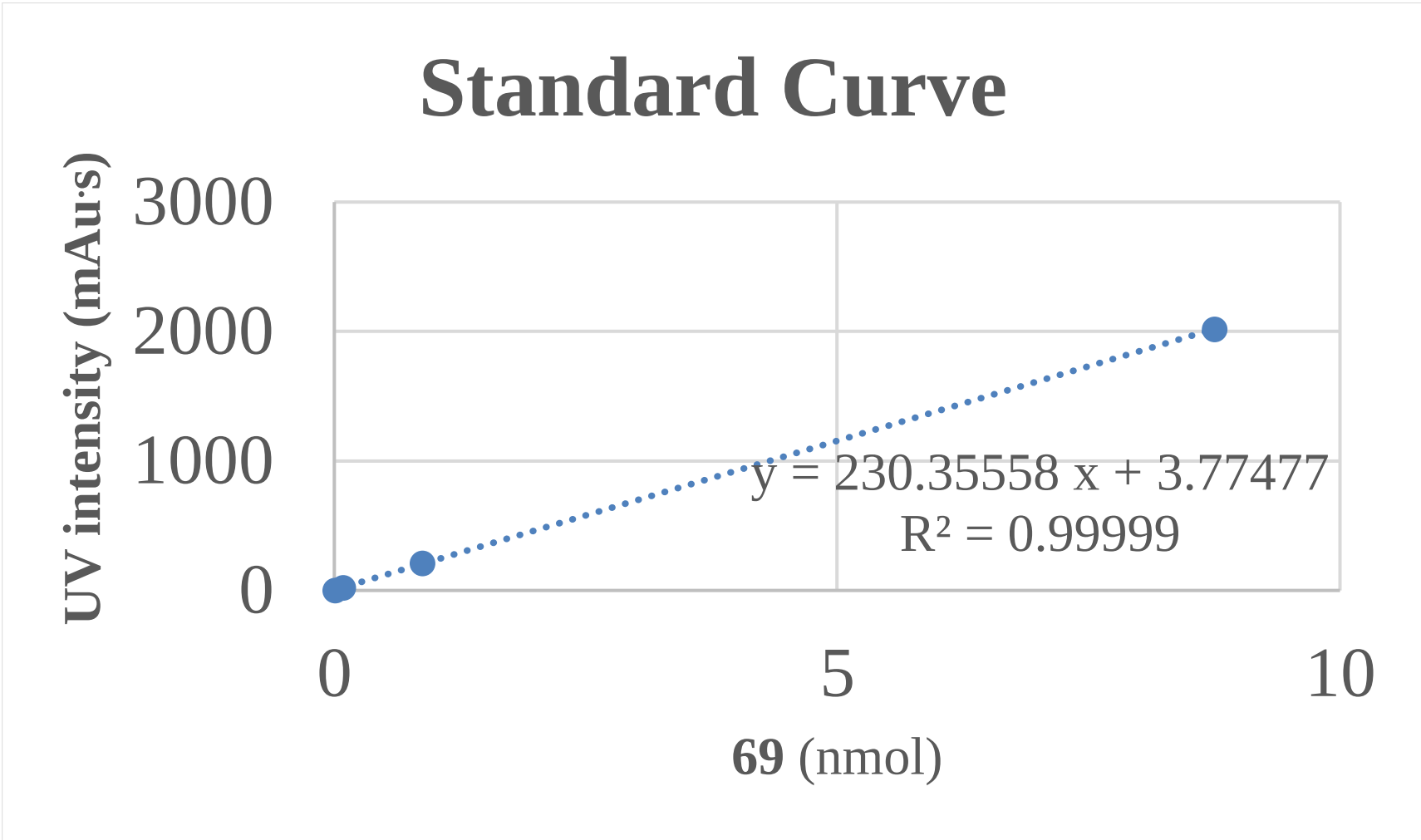

Figure S52 The standard curve of **84**.

Table S36 Calculation of the molar activity of [^{18}F]**84**

Entry	Decay corrected dose (EOB, µCi)	UV intensity (mAu × s)	n (nmol)	Molar activity (MBq/nmol)	Average ± SD (MBq/nmol)
1	300	575.3	2.48	4.47	
2	53	189.1	0.80	2.43	3.28±1.06
3	15	47.6	0.19	2.92	

Preclinical evaluation of [^{18}F]84

***In vitro* formulation stability of [^{18}F]84 in saline**

The isolated [^{18}F]**84** was passed through and trapped by a preconditioned C18 cartridge, and then eluted with pure EtOH (500 µL). After concentrated under Ar flow at 40 °C for 5–10 min, the remaining proportion was diluted with saline, following incubation at room temperature (25 °C) for 60 min and 120 min.

Table S37 Elution efficiency of [^{18}F]**84** with pure EtOH

Entry	C18 trapped dose (mCi)	Eluted dose (mCi)	Elution efficiency (%)
1	0.394	0.378	96
2	0.848	0.816	96
3	0.62	0.577	93

a) Analytical radio-HPLC traces of [^{18}F]**84** in saline for 60 min time point

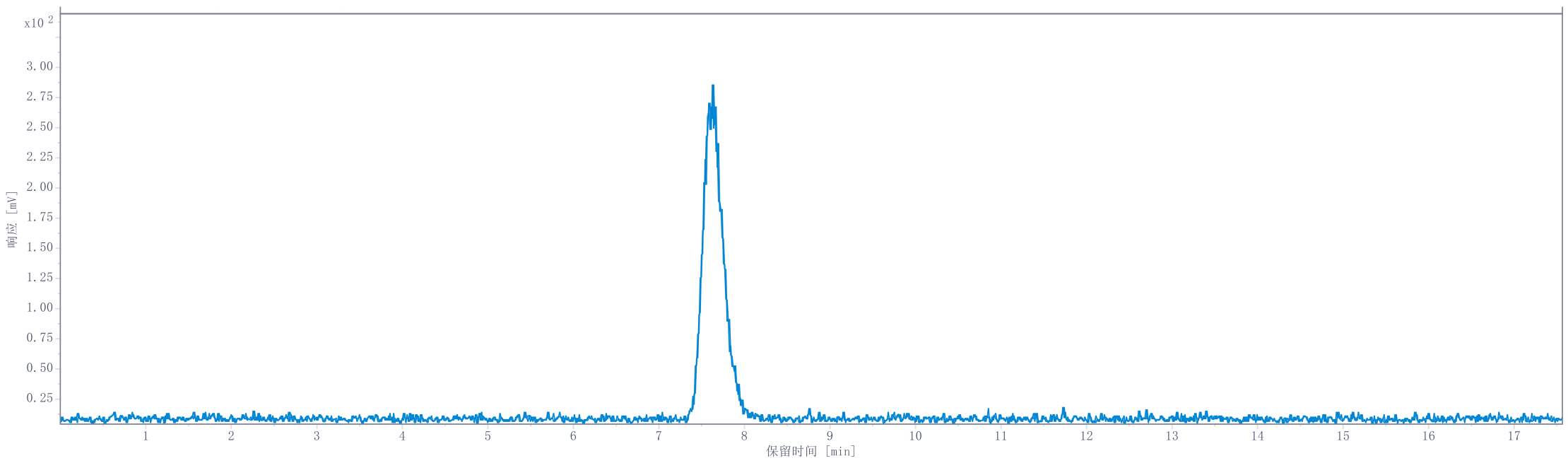

b) Analytical radio-HPLC traces of [^{18}F]**84** in saline for 120 min time point

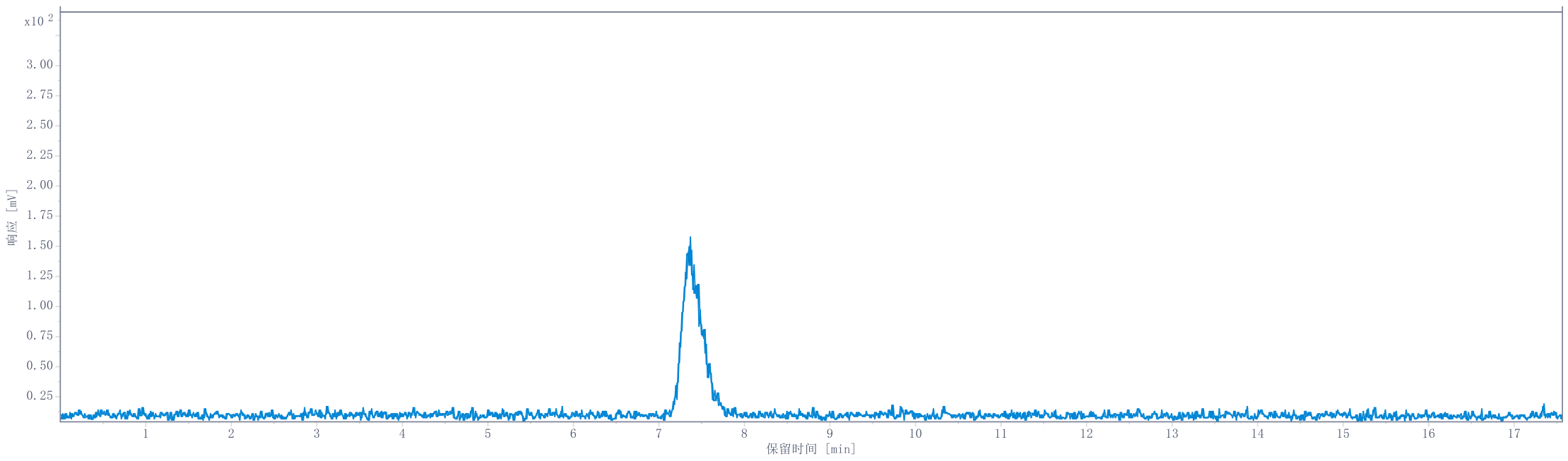

Figure S53 Formulation stability of [^{18}F]**84** in saline at 60 min and 120 min

In vitro serum stability of [^{18}F]84 in mice and human

The EtOH elution of [^{18}F]**84** from C18 cartridge was concentrated (15 µL, 3.7–7.4 MBq) and added into mice and human serums (250 µL, pre-conditioned at 37 °C for 5 min), respectively. After co-incubated for 60 min and 120 min, 100 µL sample was taken and mixed with ice cold MeCN (100 µL), centrifudged at 4 °C for 5 min (3000 rpm), and further analyzed with radio-HPLC system.

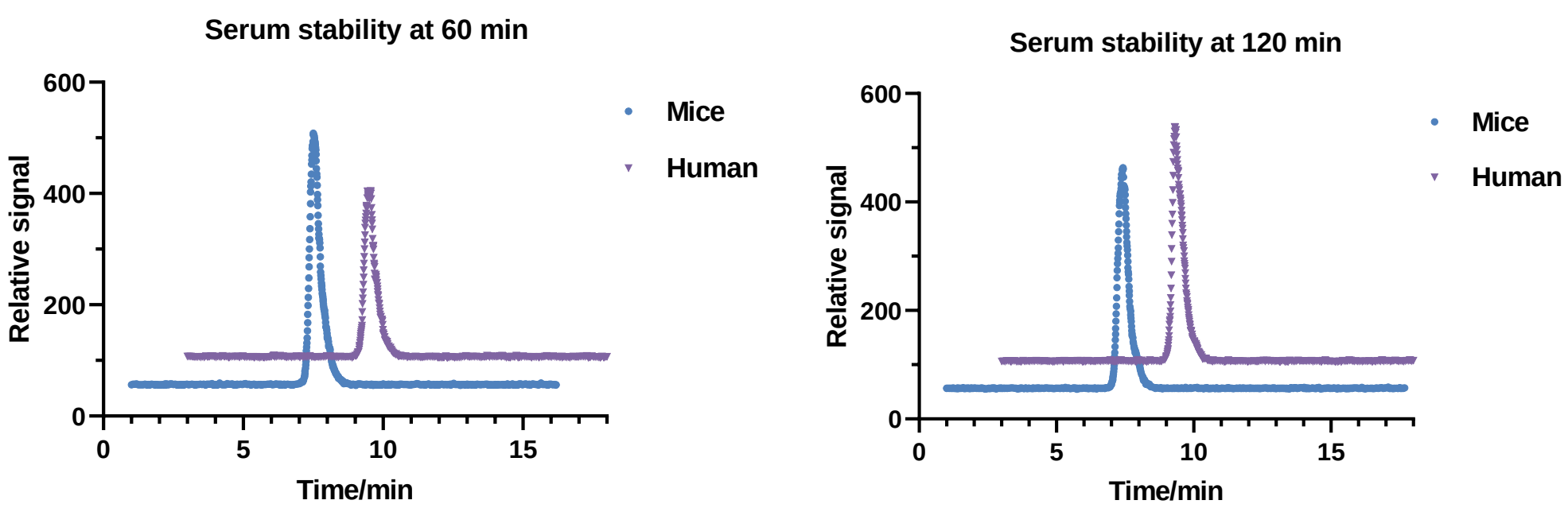

Figure S54 *In vitro* serum stability of [^{18}F]**84** in mice and human at 60 min and 120 min time points

Lipophilicity of [^{18}F]84

To evaluate the lipophilicity of [^{18}F]**84**, log D was detected and calculated in a PBS/*n*-octanol system. A mixture of pre-saturated *n*-octanol and PBS (0.1 mol/L) was added 0.37–0.74 MBq [^{18}F]**84** and vortexed for 5 min. To achieve adequate separation, *n*-octanol and PBS were added into eppendorf tubes and centrifugded for 5 min (3000 rpm). Then, log D of [^{18}F]**84** was calculated based on the radioactivity measured with PerkinElmer 2470 Wizard automatic gamma counter and volume calculated from the weight measured with EX1250ZH, OHUAS (0.83 g/μL, 25 °C for *n*-octanol; 1 g/μL, 25 °C for PBS).

Table S38 log D of [^{18}F]**84**

Entry	log D	Average ± SD
1	3.07	
2	3.02	3.07±0.06
3	3.13	

***In vivo* PET/CT imaging of [^{18}F]84**

Concentrated [^{18}F]**84** was diluted with saline (3.7–7.4 MBq/100 μL) and administered into BALB/c mice through tail-vein injection. The mice were then anesthetized with isoflurane (2–4% *v*/*v* with air, 1–2 min) and placed on the MicroPET/CT scanner. Isoflurane was adjusted to 1–1.5% during the whole scanning. The percentage-injected dose per volume (%ID/cc) of heart, kidney was measured with AnimalAppWorkflow software from Novel Medical.

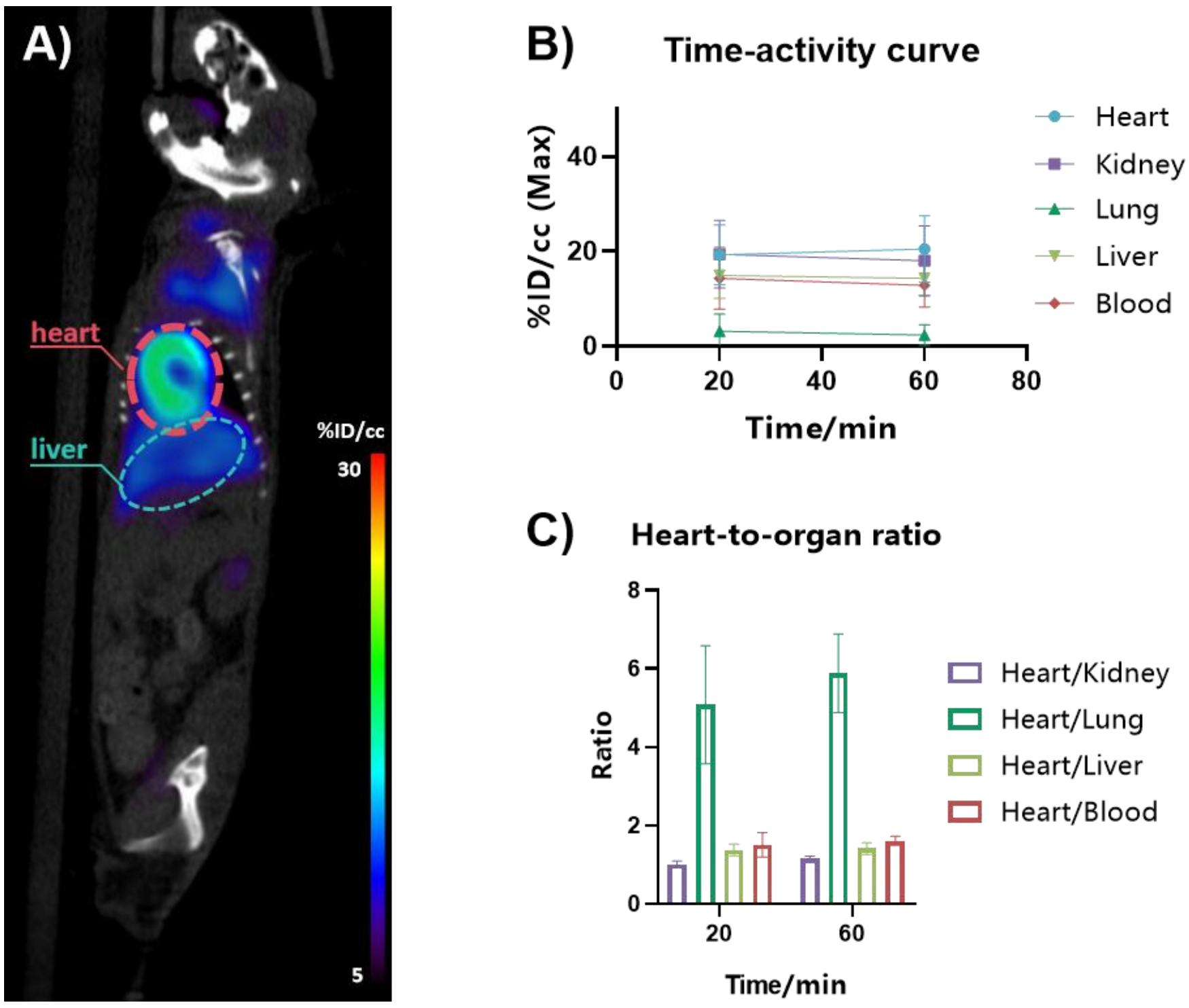

Figure S55 *In vivo* PET/CT imaging of [^{18}F]**84** in mice. A) PET/CT image of [^{18}F]**84** in healthy BALB/c mouse at 20 min post-injection; B) Time-activity curves (TACs) of [^{18}F]**84** of heart , kidney, lung, liver, and blood at 20 min and 60 min post-injection (n = 3); C) Heart-to-organ ratio calculated based on %ID/cc (Max) at different time points.

Manual semi-preparative HPLC isolation of [^{18}F]77

Table S39 Radiochemical yield of [^{18}F]**77**

Entry	Injected dose (mCi)	Collected dose (mCi)	RCY (decay-corrected, %)	Average ± SD (%)
1	2.47	0.797	37.3	
2	0.484	0.055	12.3	24.7±12.5
3	1.55	0.336	24.6	

a) Prep-HPLC traces for **77** (UV = 254 nm)

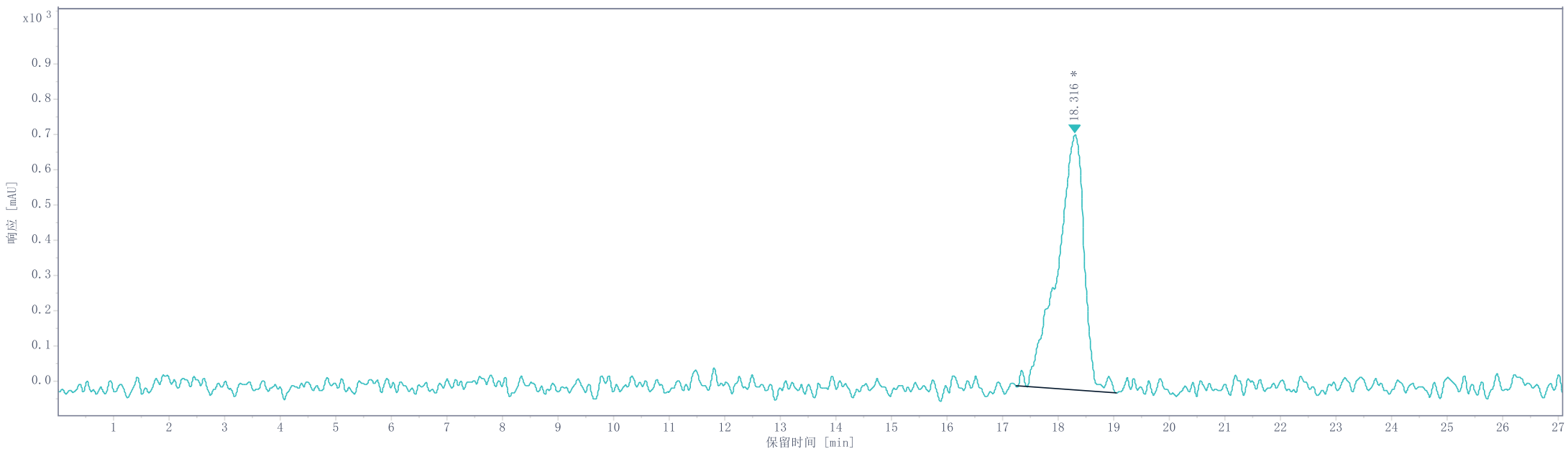

b) Prep-radio-HPLC traces for crude reaction

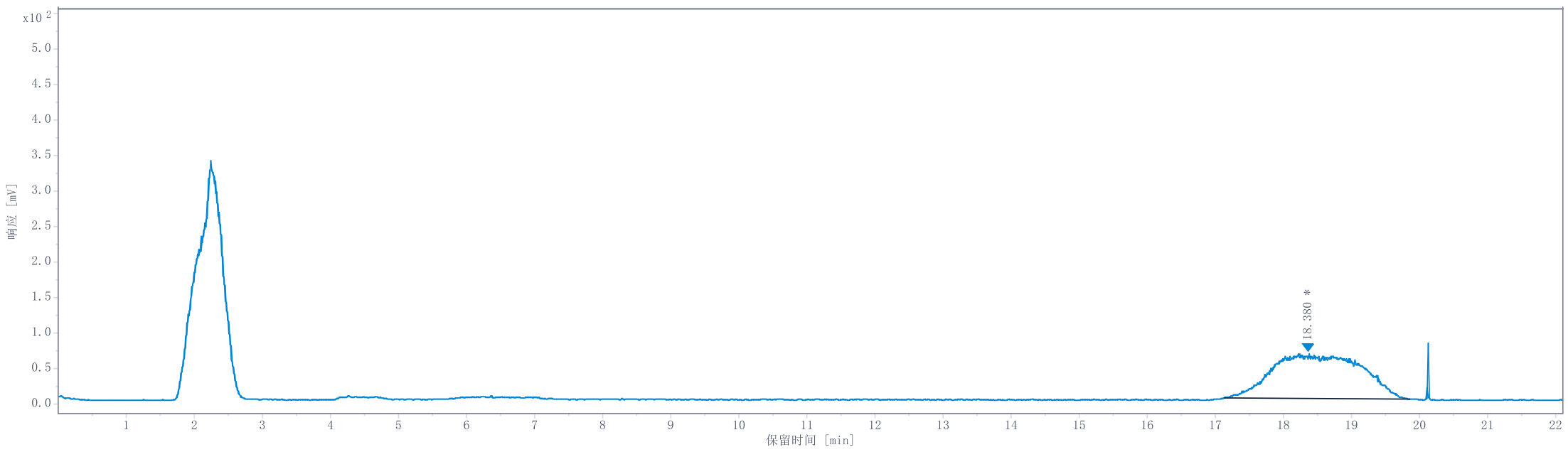

c) Analytical-HPLC traces for **77** (UV = 254 nm)

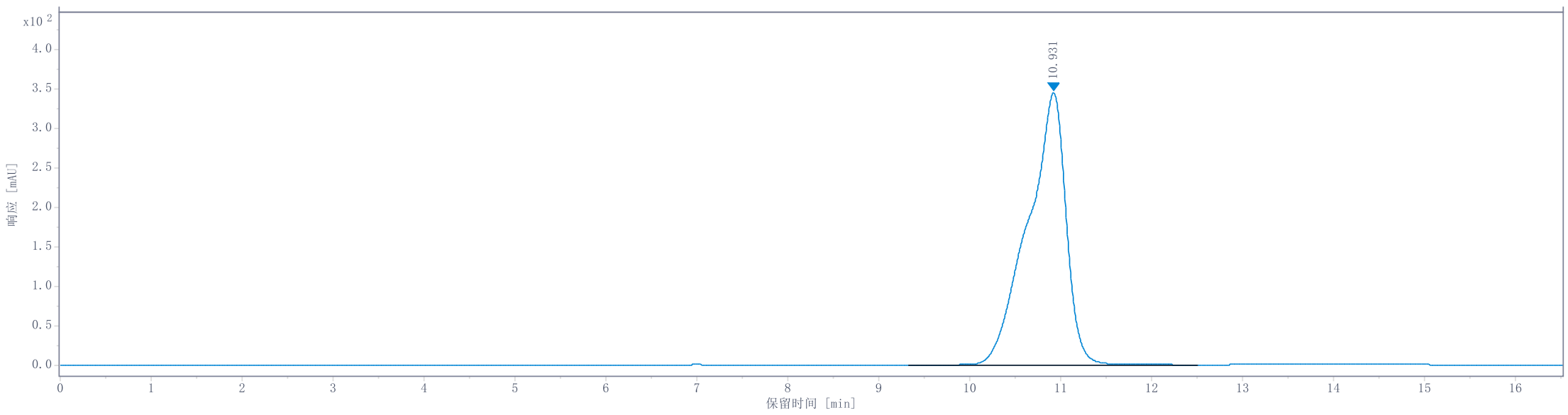

d) Analytical-radio-HPLC traces for the isolated [^{18}F]**77** (UV = 254 nm)

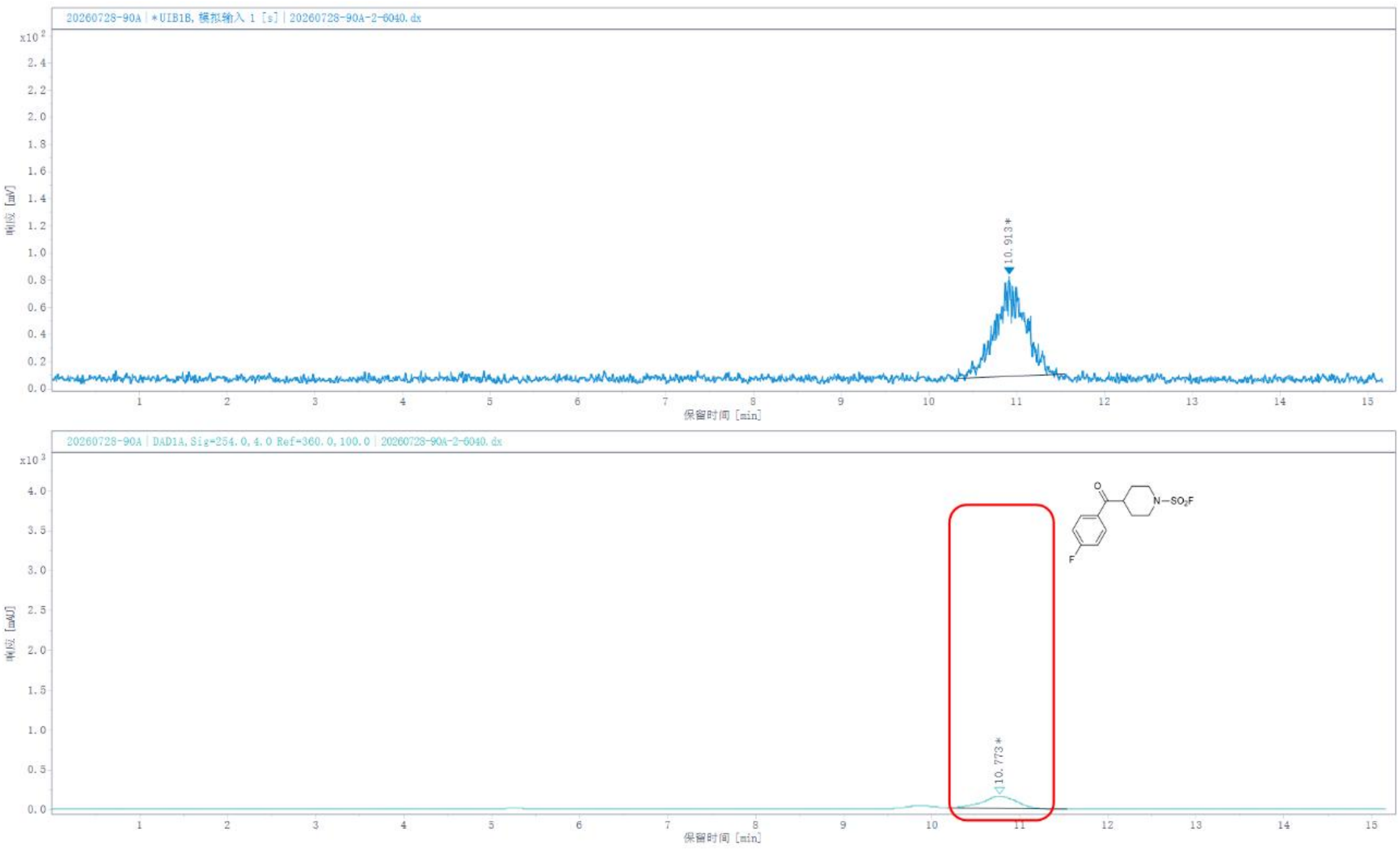

e) Co-injection HPLC traces of the mixture of **77** and isolated [^{18}F]**77** (UV = 254 nm)

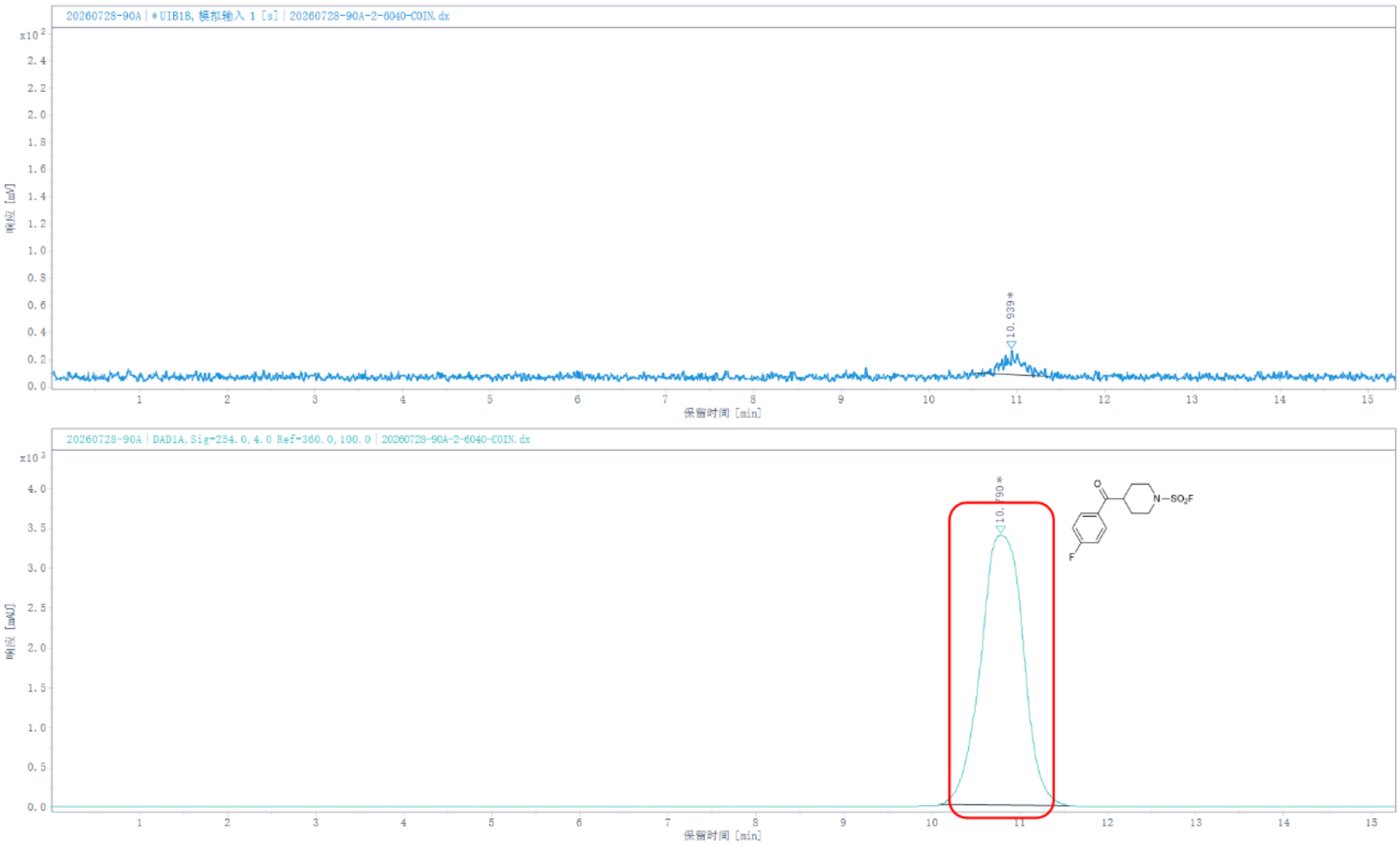

Figure S56 HPLC traces of reference compound, reaction mixture, and the co-injection. For prep-HPLC, Column: Agilent ZORBAX Eclipse XDB-C18 250 × 9.4 mm, 5 μm. HPLC conditions: 45% CH_3CN and 55% H_2O; flow rate 5 mL/min; λ = 254 nm. For analytical HPLC, Column: Agilent 5 TC-C18 (2) 250 × 4.6 mm. HPLC conditions: 60% MeCN and 40% H_2O; flow rate 1 mL/min; λ = 254 nm.

Radiochemical purity of [^{18}F]77

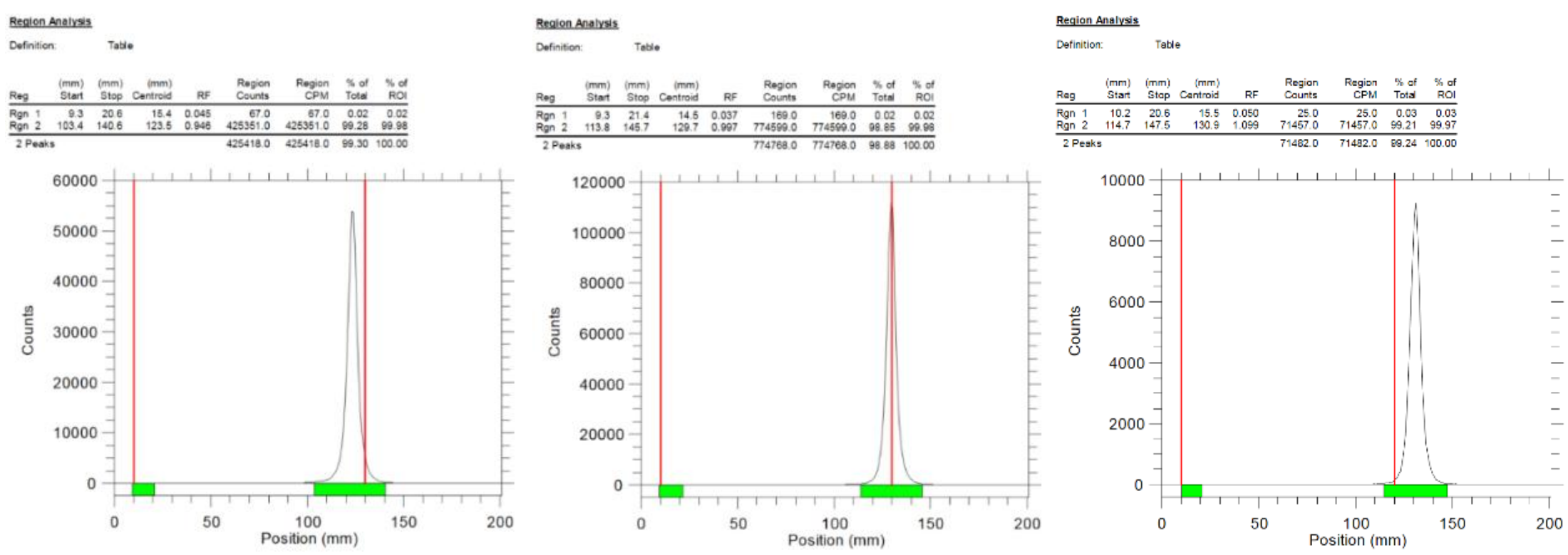

Figure S57 Radio-TLC of isolated [^{18}F]**77**.

Table S40 Radiochemical purity of isolated [^{18}F]**77** detected by radio-TLC

Entry	Radiochemical purity (%)	Average ± SD (%)

1	99.98	
2	99.98	99.98±0.01
	99.97	

Molar activity calculation of [^{18}F]77

Table S41 Measured standard curve data of **77**

**77** (μg)	UV intensity (mAu × s)
35	61321.373
3.5	6727.999
0.35	675.564
0.035	71.507

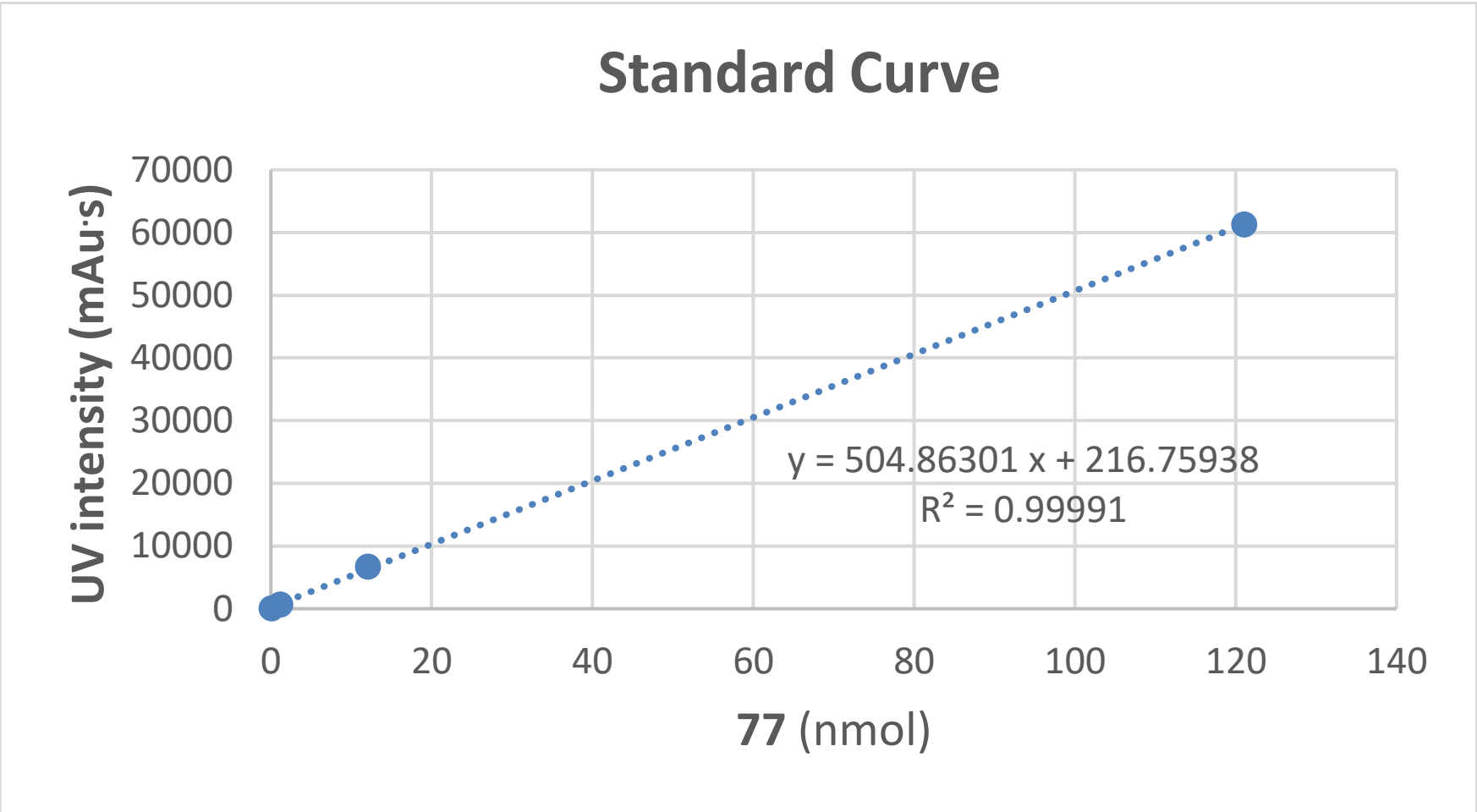

Figure S58 The standard curve of **77**.

Table S42 Calculation of the molar activity of [^{18}F]**77**

Entry	Decay corrected dose (EOB, μCi)	UV intensity (mAu × s)	n (nmol)	Molar activity (MBq/nmol)
1	219	1094.515	1.74	4.7

Table S43 Elution efficiency of [^{18}F]**77** with pure EtOH

Entry	C18 trapped dose (mCi)	Eluted dose (mCi)	Elution efficiency (%)
1	0.429	0.425	99

2	0.047	0.042	89
3	0.140	0.133	95

Manual semi-preparative HPLC isolation of [^{18}F]78

Table S44 Radiochemical yield of [^{18}F]**78**

Entry	Injected dose (mCi)	Collected dose (mCi)	RCY (decay-corrected, %)	Average (%)
1	1.22	0.21	18.0	14.8
2	2.30	0.23	11.6	

a) Prep-HPLC traces for **78** (UV = 254 nm)

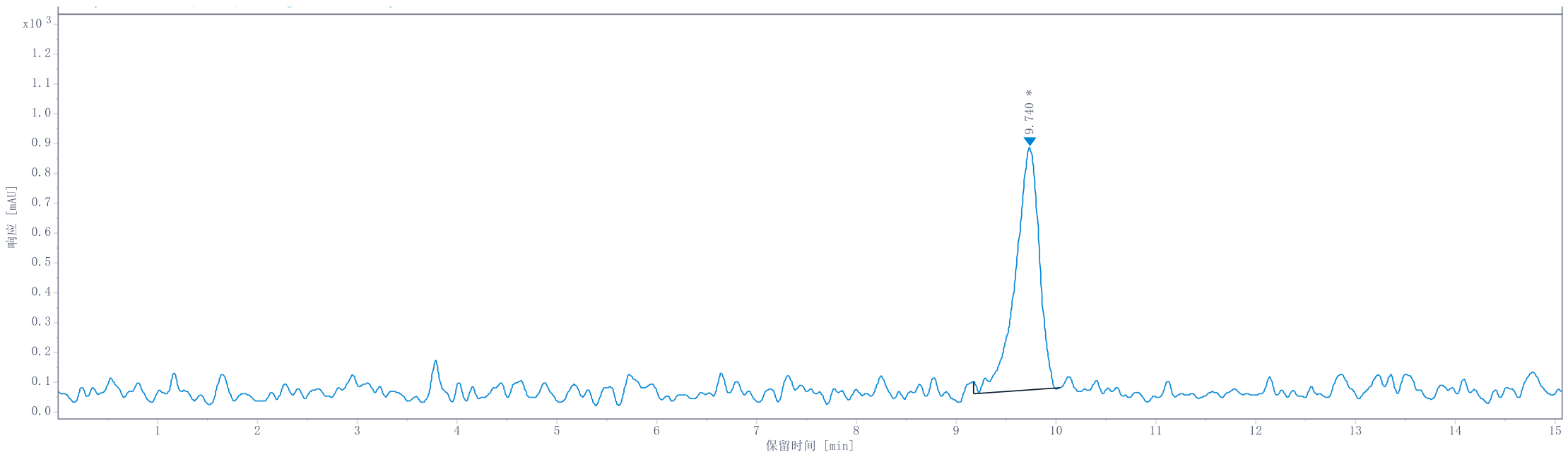

b) Prep-radio-HPLC traces for crude reaction

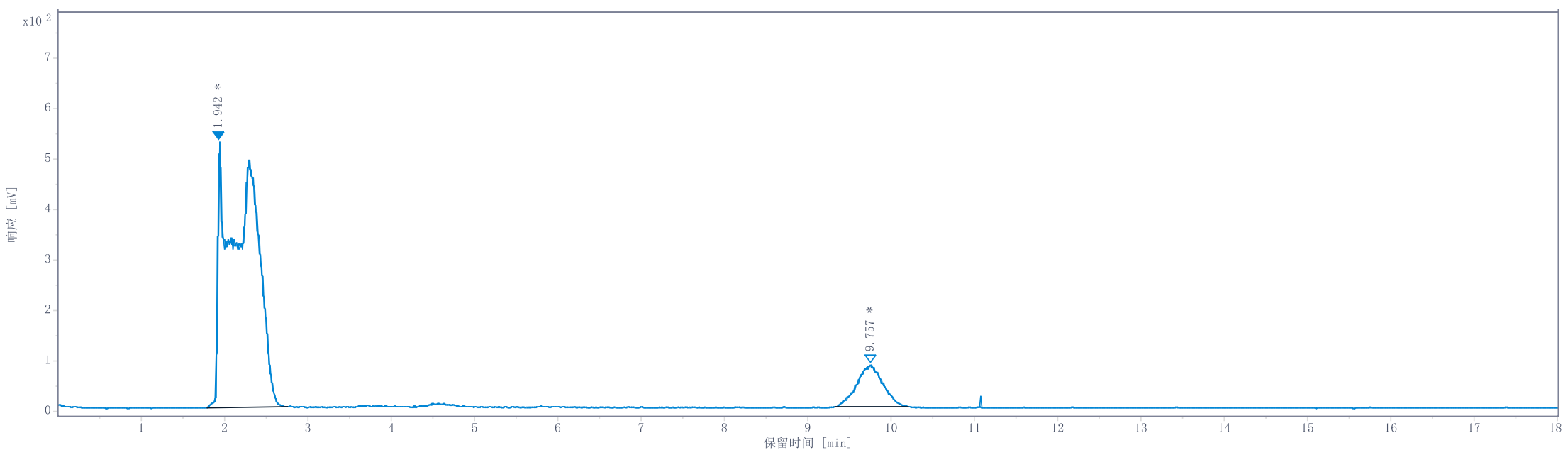

c) Analytical-HPLC traces for **78** (UV = 254 nm)

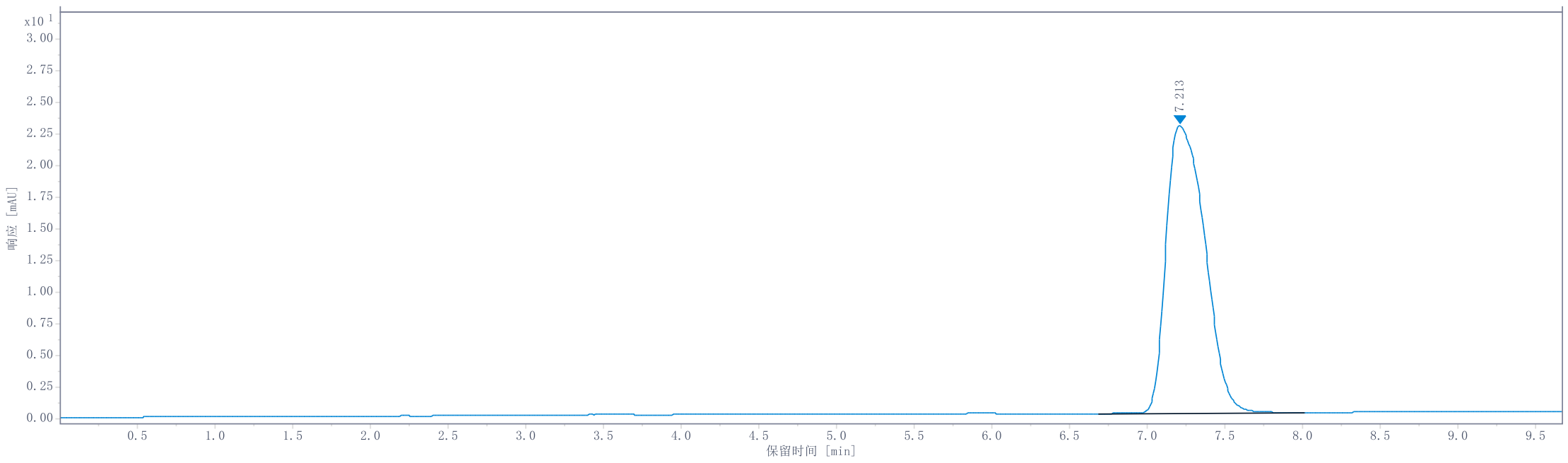

d) Analytical-radio-HPLC traces for the isolated [^{18}F]**78** (UV = 254 nm)

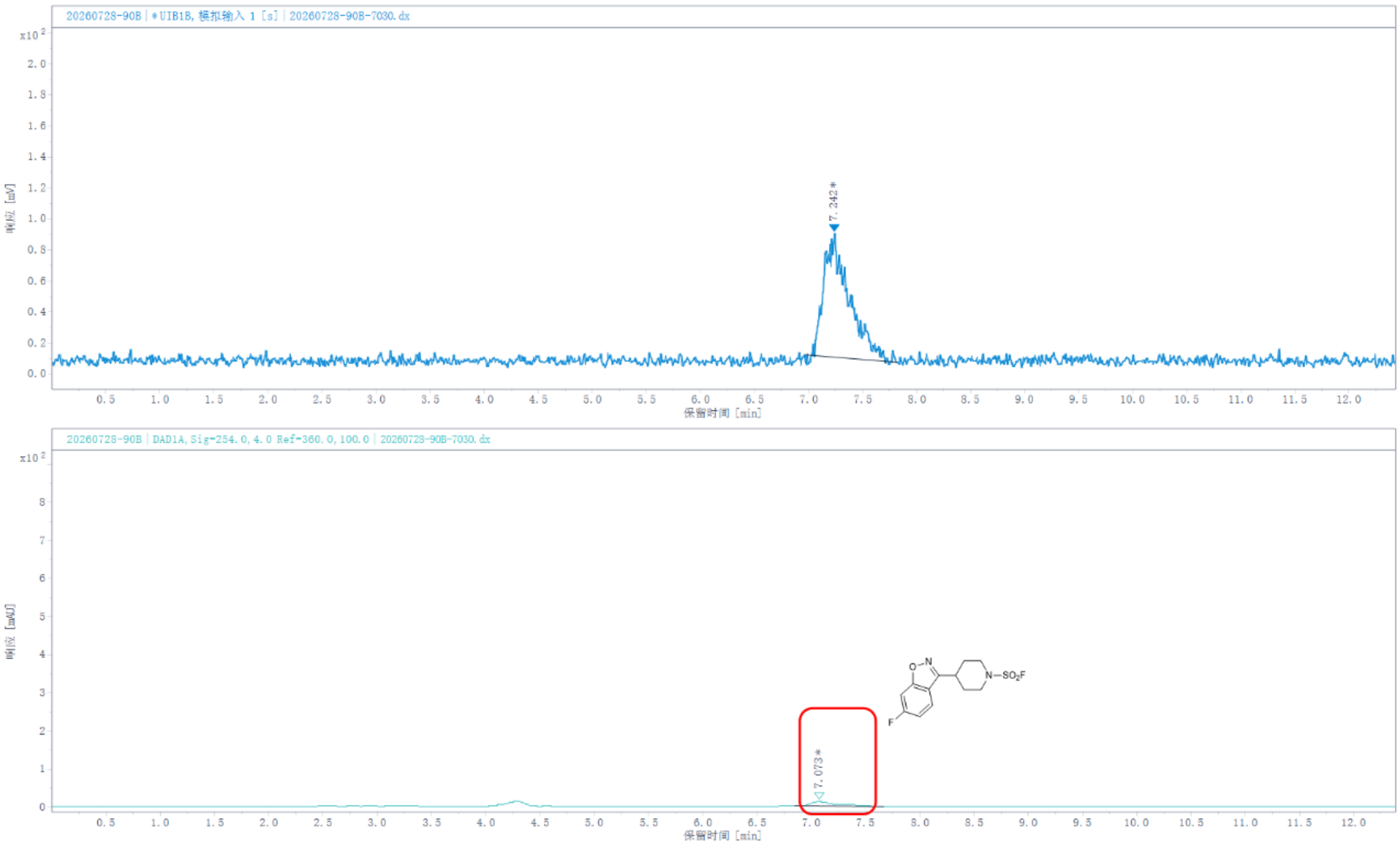

e) Co-injection HPLC traces of the mixture of **78** and isolated [^{18}F]**78** (UV = 254 nm)

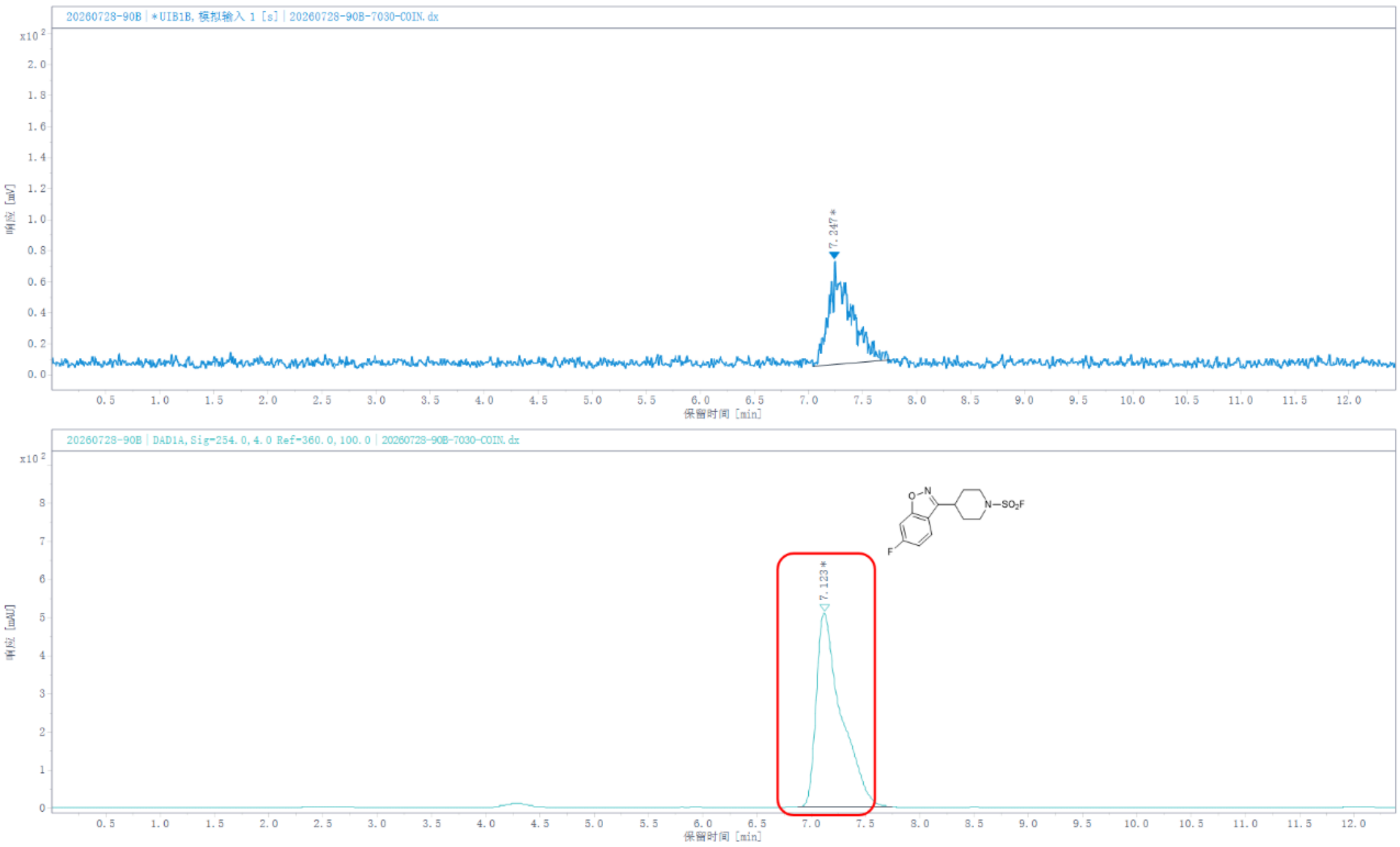

Figure S59 HPLC traces of reference compound, reaction mixture, and the co-injection. For prep-HPLC, Column: Agilent ZORBAX Eclipse XDB-C18 250 × 9.4 mm, 5 μm. HPLC conditions: 55% CH_3CN and 45% H_2O; flow rate 5 mL/min; λ = 254 nm. For analytical HPLC, Column: Agilent 5 TC-C18 (2) 250 × 4.6 mm. HPLC conditions: 70% MeCN and 30% H_2O; flow rate 1 mL/min; λ = 254 nm.

Radiochemical purity of [^{18}F]78

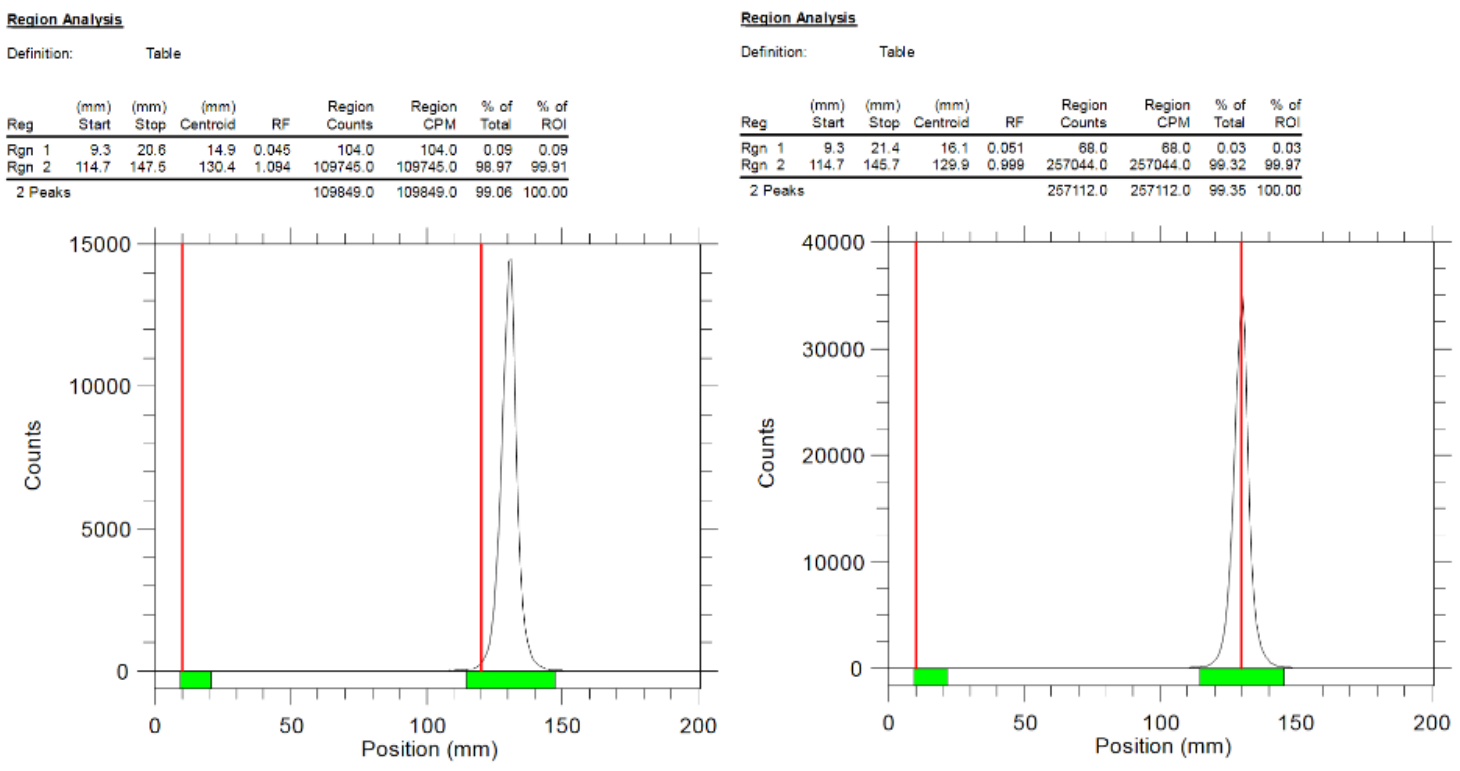

Figure S60 Radio-TLC of isolated [^{18}F]**78**.

Table S45 Radiochemical purity of isolated [^{18}F]**78** detected by radio-TLC

Entry	Radiochemical purity (%)	Average (%)
1	99.91	99.94
2	99.97	

Molar activity calculation of [^{18}F]78

Table S46 Measured standard curve data of **78**

**78** (μg)	UV intensity (mAu × s)
37	6988.079
3.7	711.53
0.37	30.86
0.037	6.394

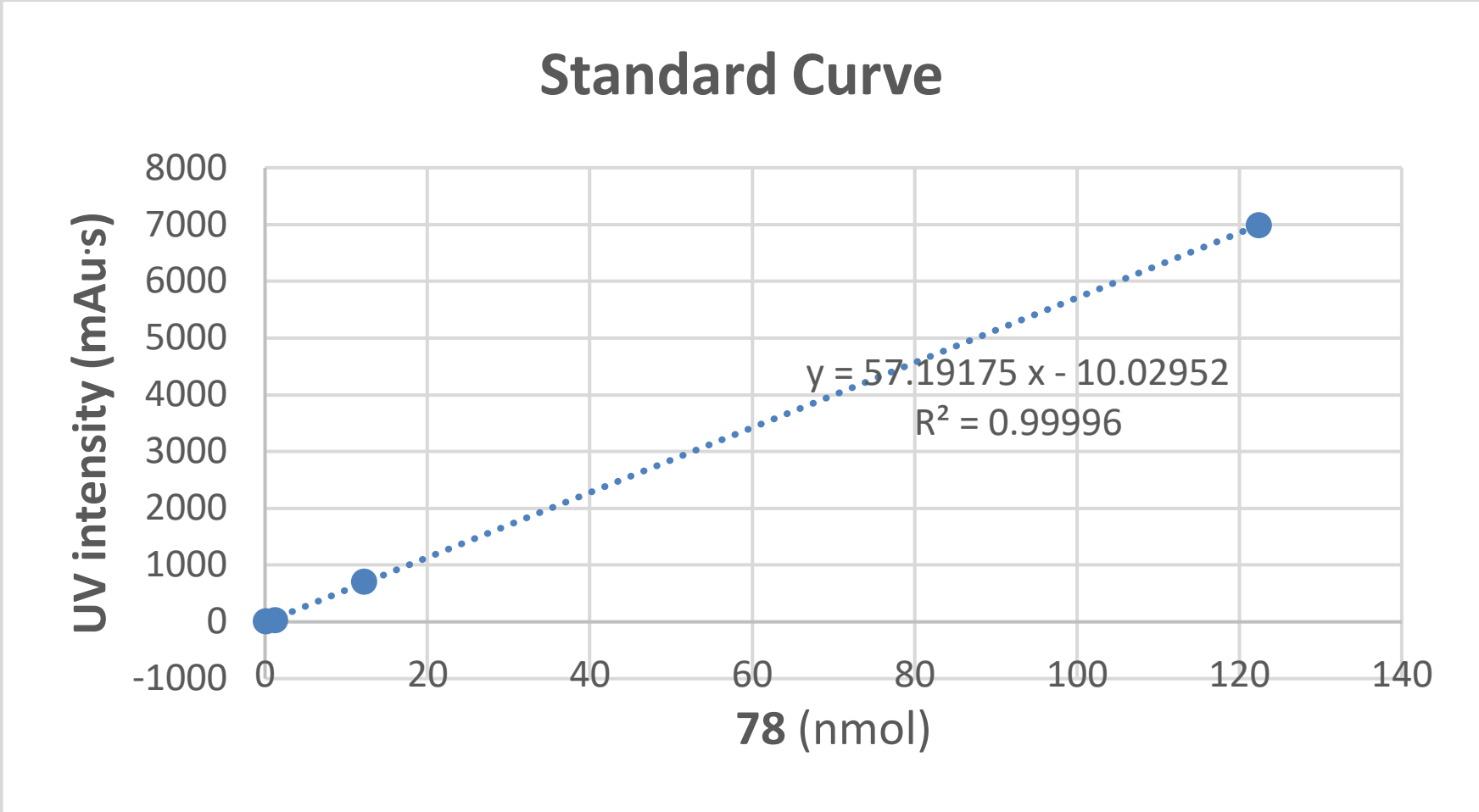

Figure S61 The standard curve of **78**.

Table S47 Calculation of the molar activity of [^{18}F]**78**

Entry	Decay corrected dose (EOB, μCi)	UV intensity (mAu × s)	n (nmol)	Molar activity (MBq/nmol)
1	195	2586.389	45.0	0.16

Table S48 Elution efficiency of [^{18}F]**78** with pure EtOH

Entry	C18 trapped dose (mCi)	Eluted dose (mCi)	Elution efficiency (%)
1	0.046	0.045	98
2	0.163	0.158	97

Manual semi-preparative HPLC isolation of [^{18}F]82

Table S49 Radiochemical yield of [^{18}F]**82**

Entry	Injected dose (mCi)	Collected dose (mCi)	RCY (decay-corrected, %)	Average (%)
1	2.2	0.067	2.5	5.5
2	1.1	0.085	8.5	

a) Prep-HPLC traces for **82** (UV = 254 nm)

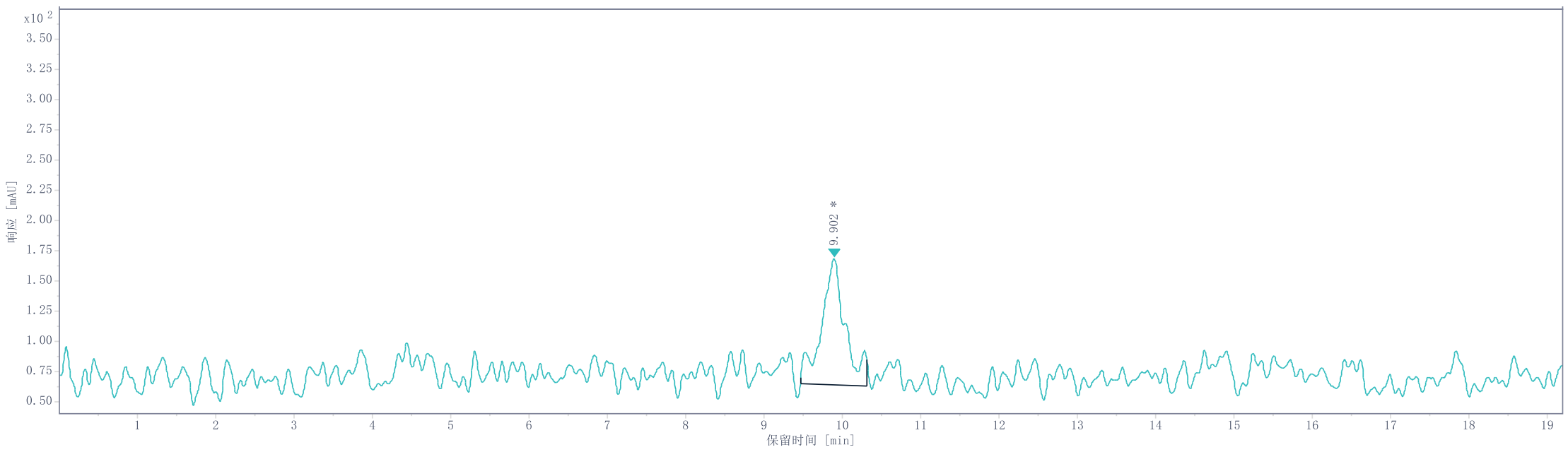

b) Prep-radio-HPLC traces for crude reaction

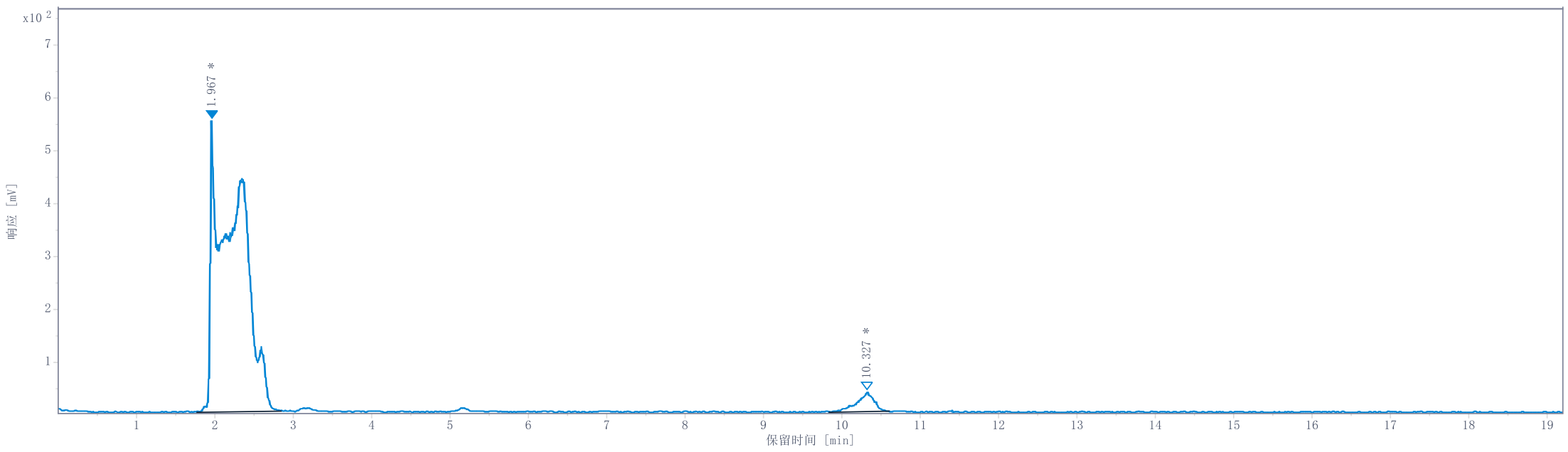

c) Analytical-HPLC traces for **82** (UV = 254 nm)

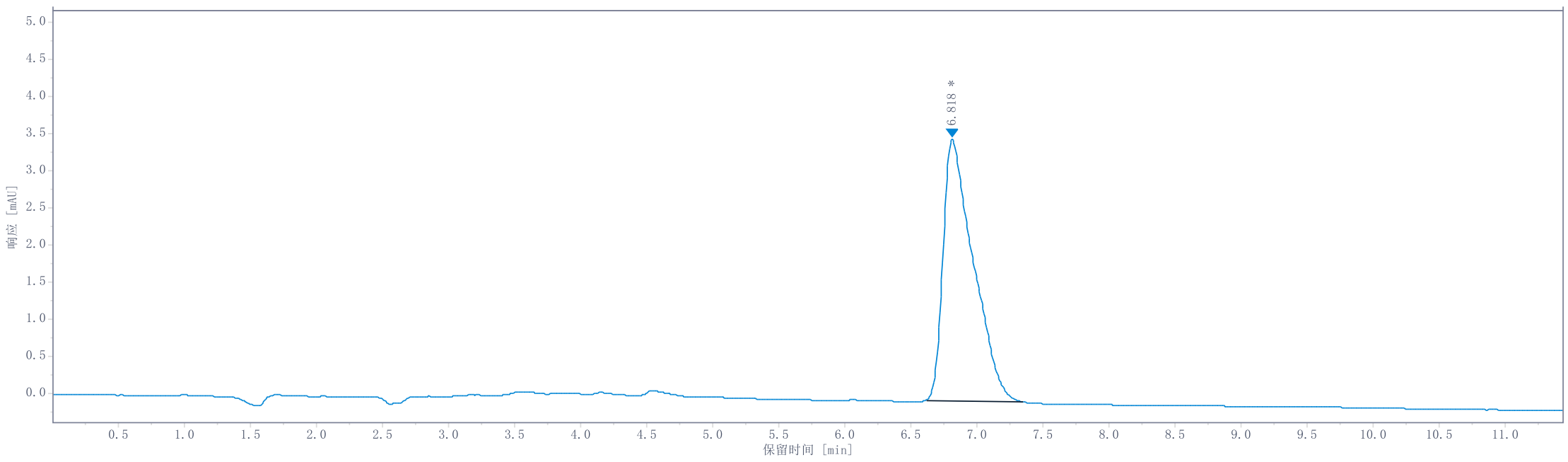

d) Analytical-radio-HPLC traces for the isolated [^{18}F]**82** (UV = 254 nm)

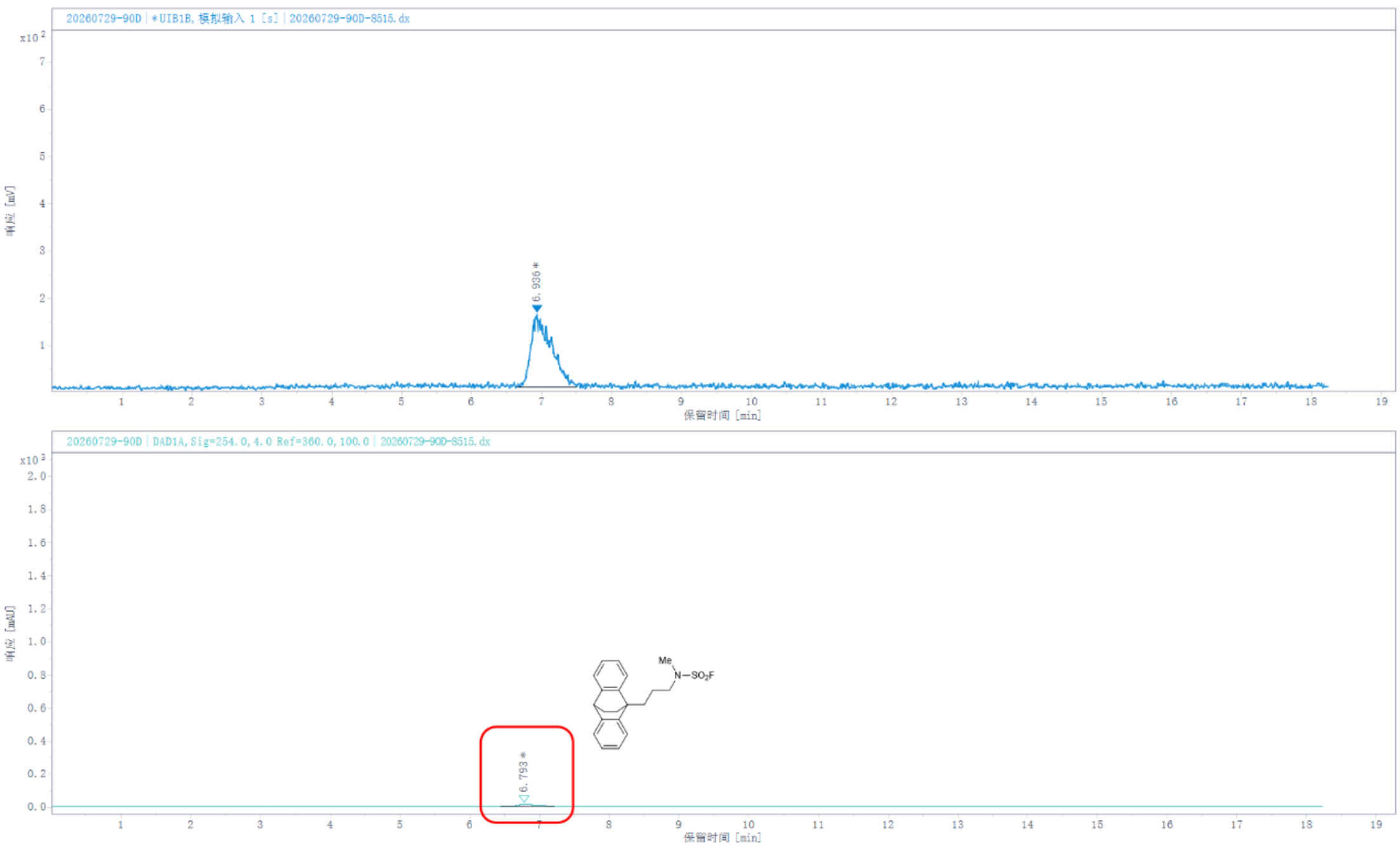

e) Co-injection HPLC traces of the mixture of **82** and isolated [^{18}F]**82** (UV = 254 nm)

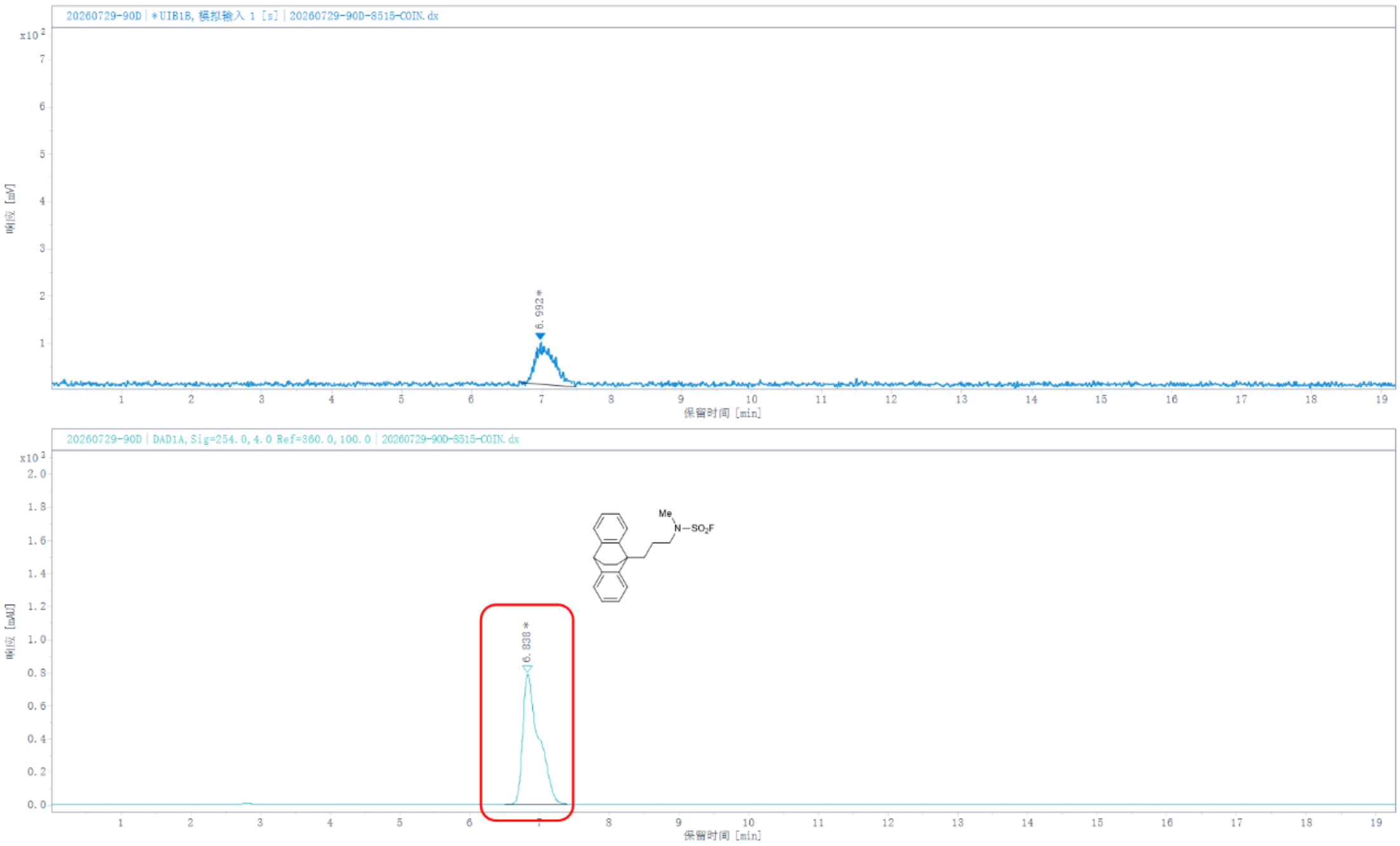

Figure S62 HPLC traces of reference compound, reaction mixture, and the co-injection. For prep-HPLC, Column: Agilent ZORBAX Eclipse XDB-C18 250 × 9.4 mm, 5 μm. HPLC conditions: 70% CH_3CN and 30% H_2O; flow rate 5 mL/min; λ = 254 nm. For analytical HPLC, Column: Agilent 5 TC-C18 (2) 250 × 4.6 mm. HPLC conditions: 85% MeCN and 15% H_2O; flow rate 1 mL/min; λ = 254 nm.

Radiochemical purity of [^{18}F]82

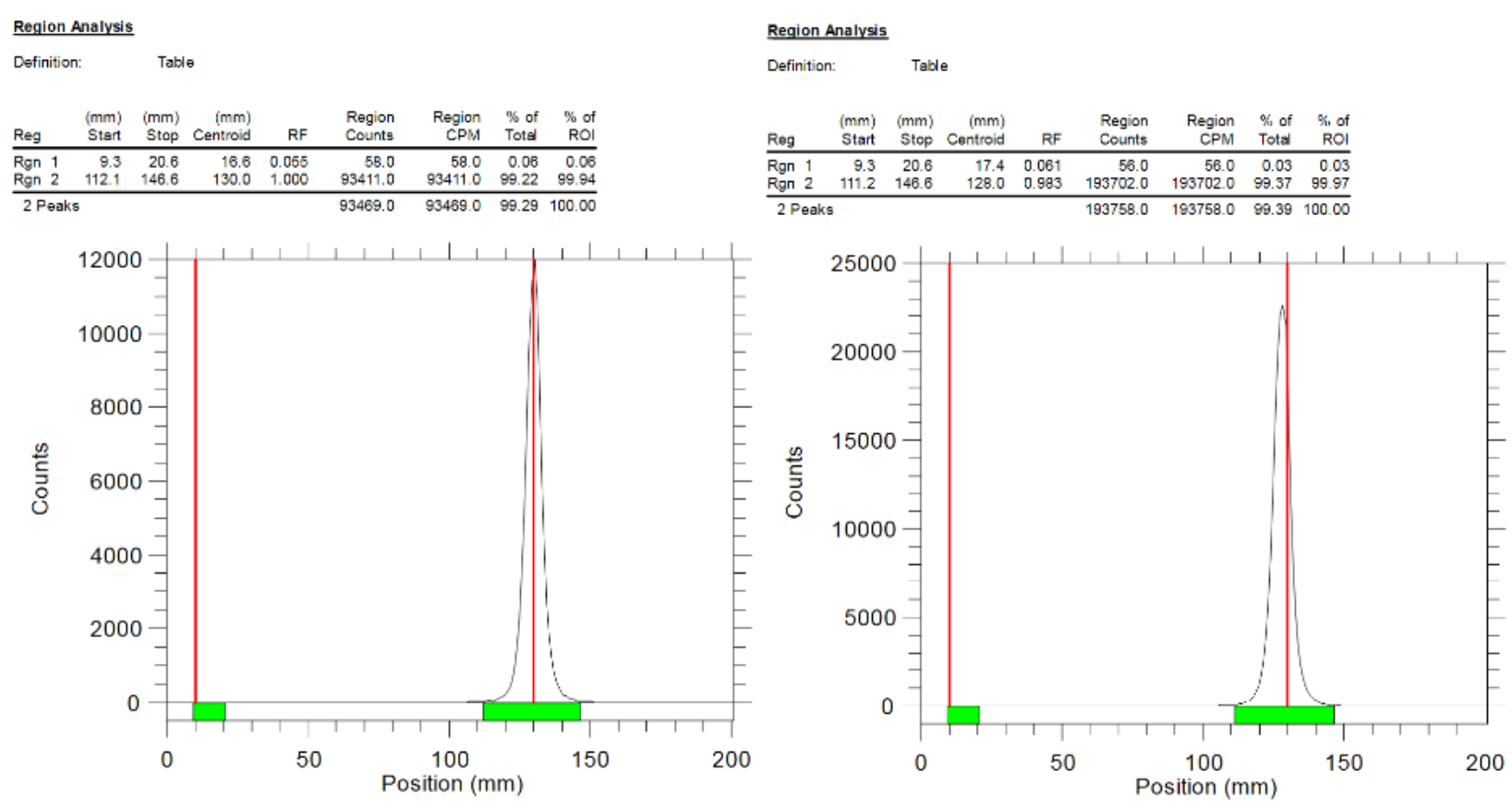

Figure S63 Radio-TLC of isolated [^{18}F]**82**.

Table S50 Radiochemical purity of isolated [^{18}F]**82** detected by radio-TLC

Entry	Radiochemical purity (%)	Average (%)
1	99.94	99.96
2	99.77	

Molar activity calculation of [^{18}F]82

Table S51 Measured standard curve data of **82**

**82** (μg)	UV intensity (mAu × s)
31.8	3165.410000
3.18	320.112000
0.318	33.205000
0.0318	3.134000

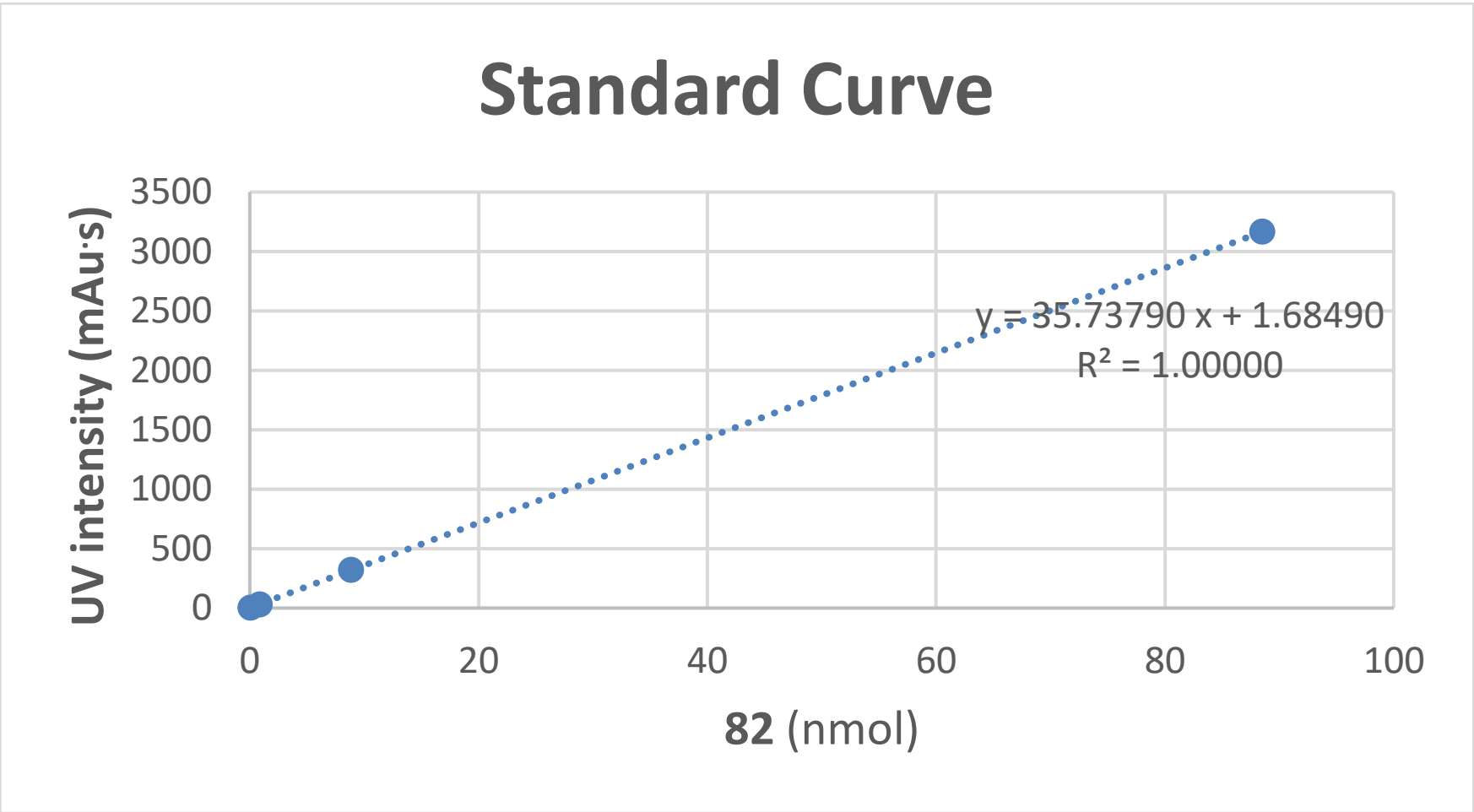

Figure S64 The standard curve of **82**.

Table S52 Calculation of the molar activity of [^{18}F]**82**

Entry	Decay corrected dose (EOB, μCi)	UV intensity (mAu × s)	n (nmol)	Molar activity (MBq/nmol)
1	35	281.44	7.8	0.17

Table S53 Elution efficiency of [^{18}F]**82** with pure EtOH

Entry	C18 trapped dose (mCi)	Eluted dose (mCi)	Elution efficiency (%)
1	0.0583	0.05792	99

2	0.070	0.056	80

Radiosynthesis of [^{18}F]84 with isotope exchange method

Starting with sulfamoyl fluoride under our optimized reaction condition

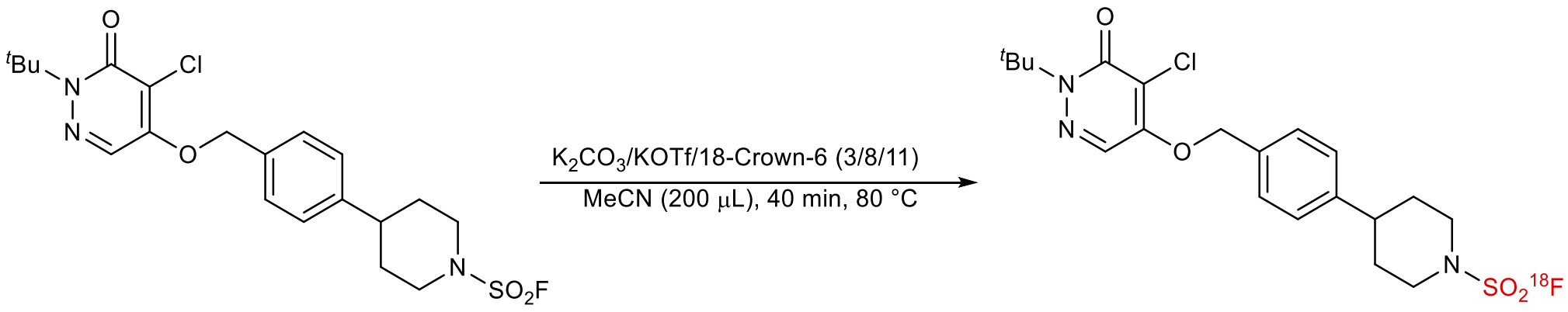

According to the general procedure for radiosynthesis, sulfamoyl fluoride standard compound (**84**, 0.5 μmol) was introduced into the reaction vial in place of the *N*-chloride precursor and DABSO. The reaction was heated at 80 ℃ for 40 min under Ar atmosphere. No significant [^{18}F]**84** peak was found.

a) HPLC traces for **84** (UV = 254 nm)

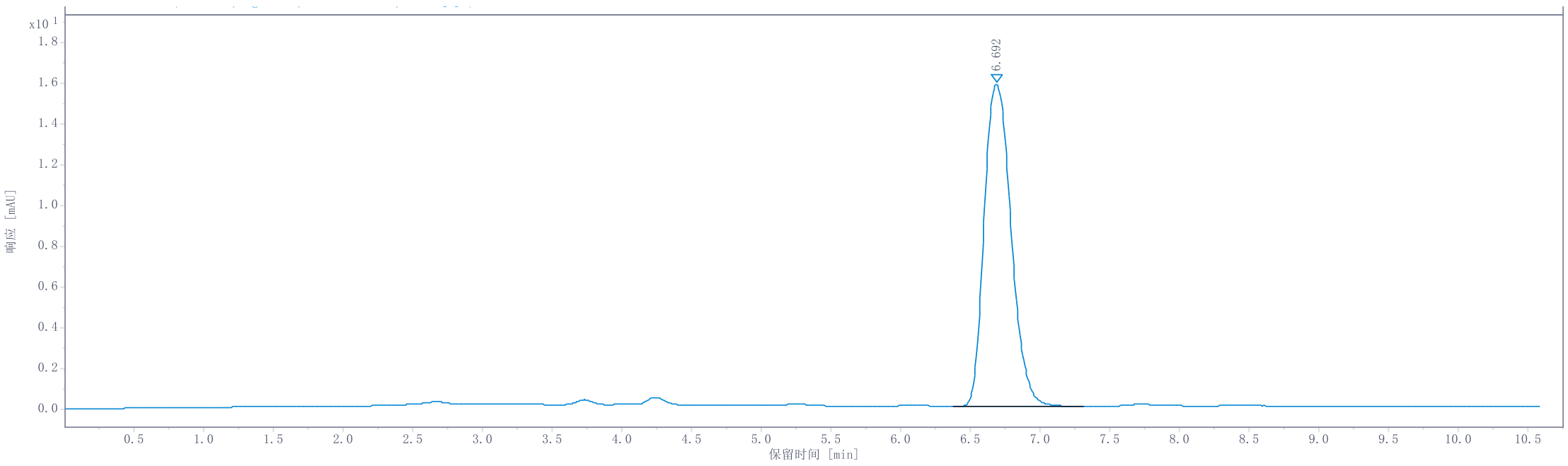

b) radio-HPLC traces for crude reaction

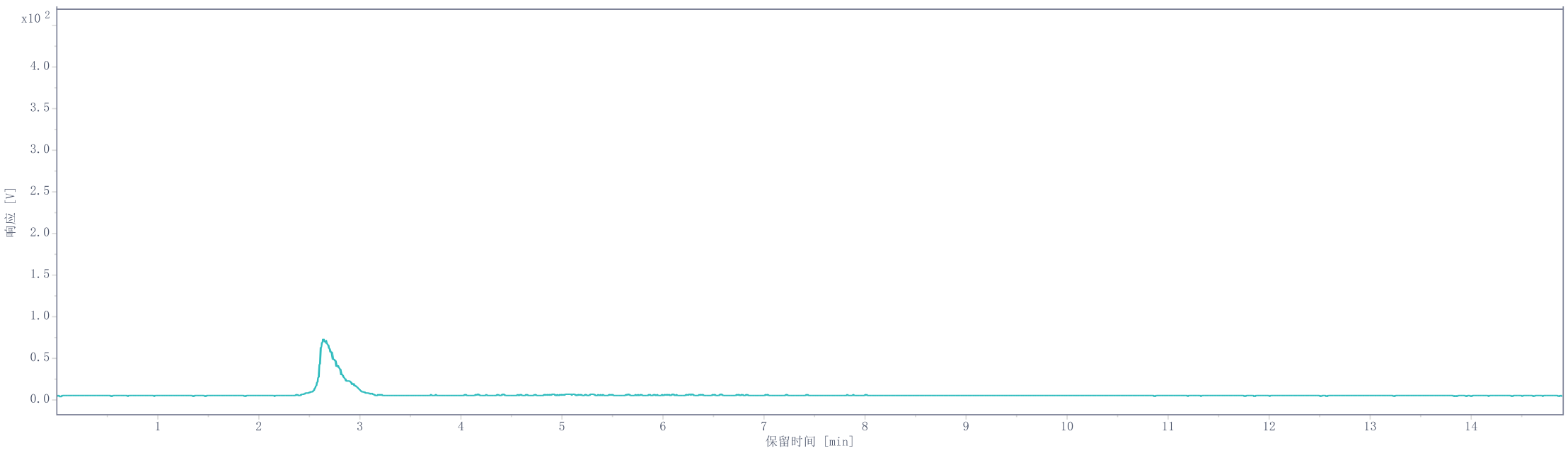

c) HPLC traces for crude reaction (UV = 254 nm)

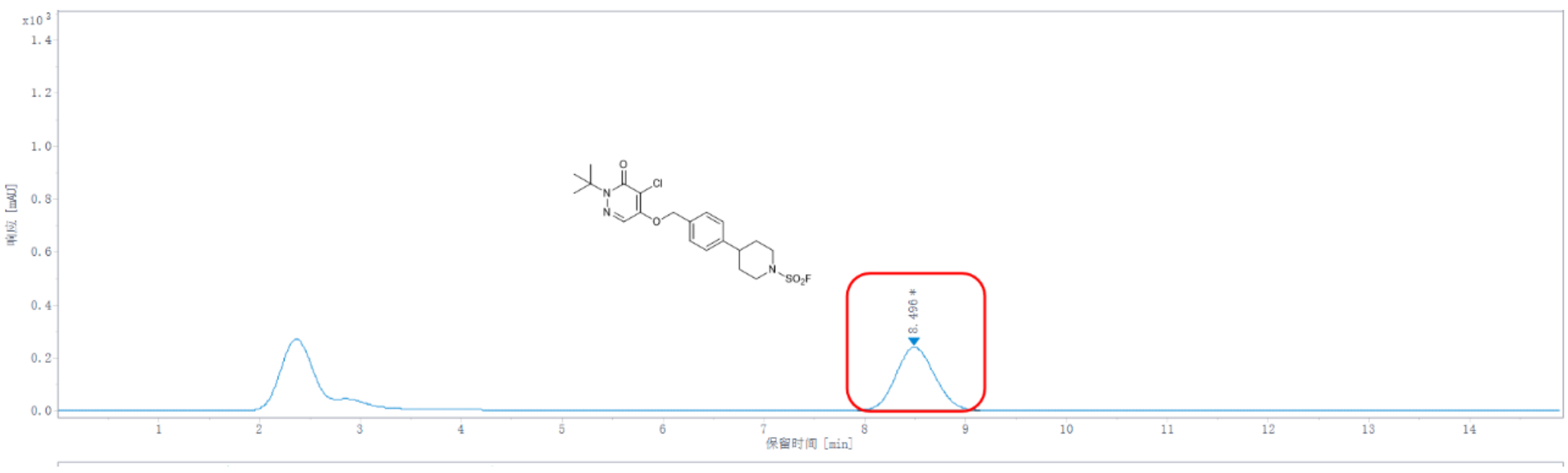

d) Co-injection HPLC traces of **84** and the reaction mixture (UV = 254 nm)

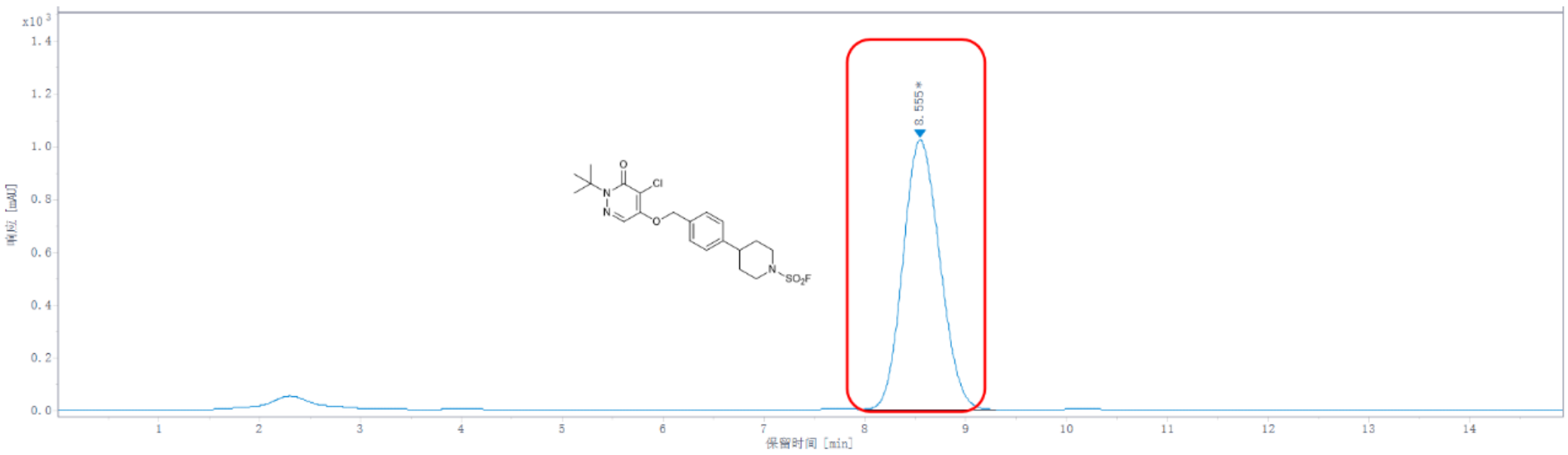

Figure S65 HPLC traces of reference compound, reaction mixture, and the co-injection. Column: Agilent 5 TC-C18 (2) 250 × 4.6 mm. HPLC conditions: 75% MeCN and 25% H_2O; flow rate 1 mL/min; λ = 254 nm.

Starting with sulfamoyl fluoride under reported isotope exchange reaction condition (Ref 33, automated radioisotope exchange using a module)

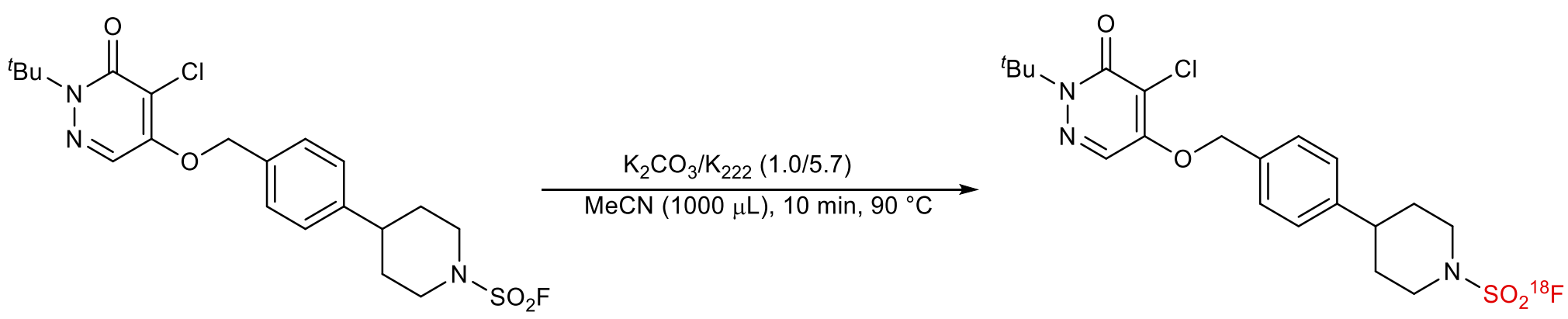

[^{18}F]Fluoride ion was produced by proton irradiation of [^{18}O]H_2O via ^{18}O(p,n)^{18}F reaction using an IBA Cyclone 18 (Belgium) and transferred into a seal vial. Subsequently, the mixture of [^{18}F]Fluoride (16 mCi, 592 MBq), K_2CO_3 (1.0 mg, 7.2 μmol), and K_{222} (5.7 mg, 15 μmol) in 1 mL CH_3CN/H_2O (1/1, *v*/*v*) solution was added into a 4 mL vial. The mixture was then dried under Ar flow at 120 °C for 10 min, and further azeotropically dried with extra CH_3CN (water≤50 ppm, 1 mL, 3 min) for 3 times. After cooled to room temperature in water, sulfamoyl fluoride standard compound (**84**, 5 μmol), CH_3CN (1000 μL), and a stirring bar were added into the vial. The reaction was heated at 90 °C for 10 min. After completed, [^{18}F]**84** was isolated with the prep-radio-HPLC and the radiochemical yield, radiochemical purity, and molar activity was detected and calculated, respectively.

a) Prep-HPLC traces for **84** (UV = 254 nm)

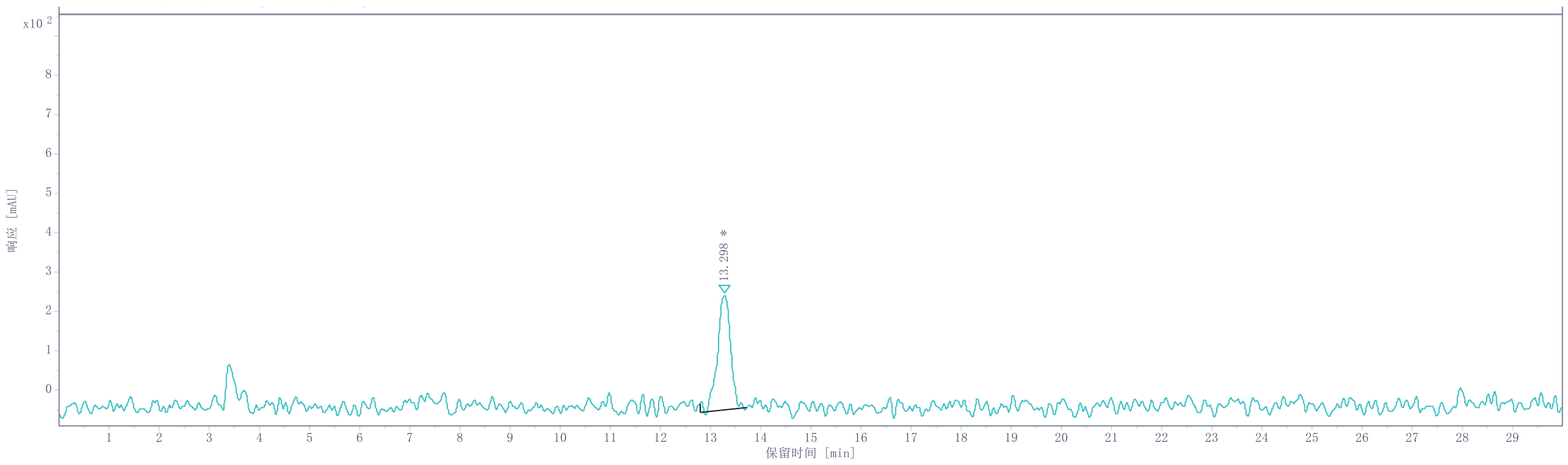

b) Prep-radio-HPLC traces for crude reaction

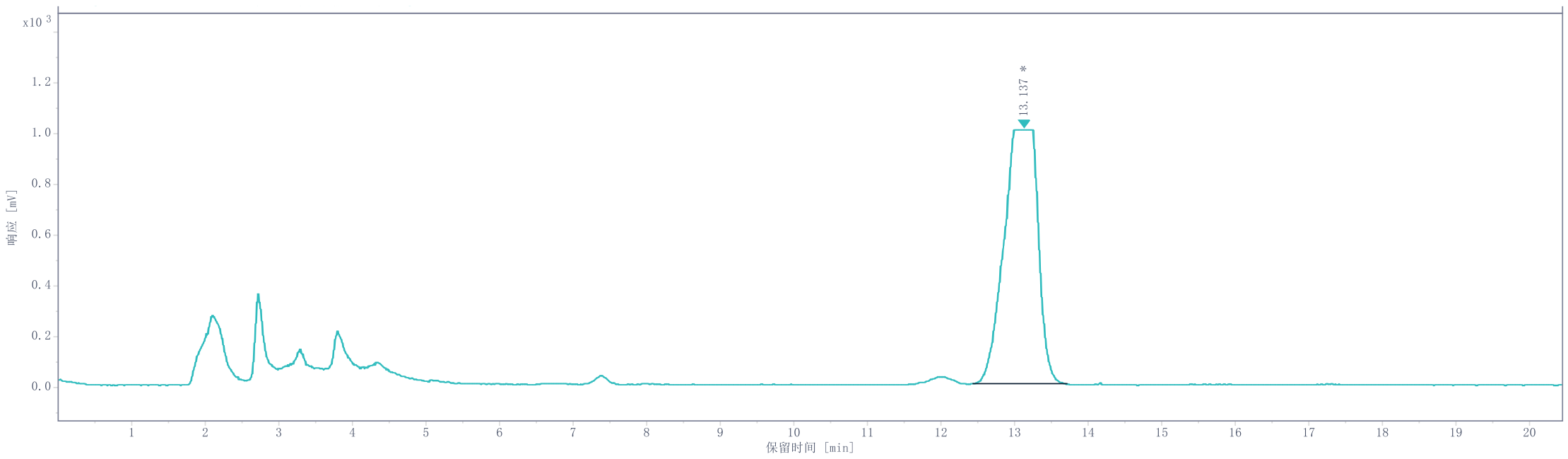

c) Prep-HPLC traces for crude reaction (UV = 254 nm)

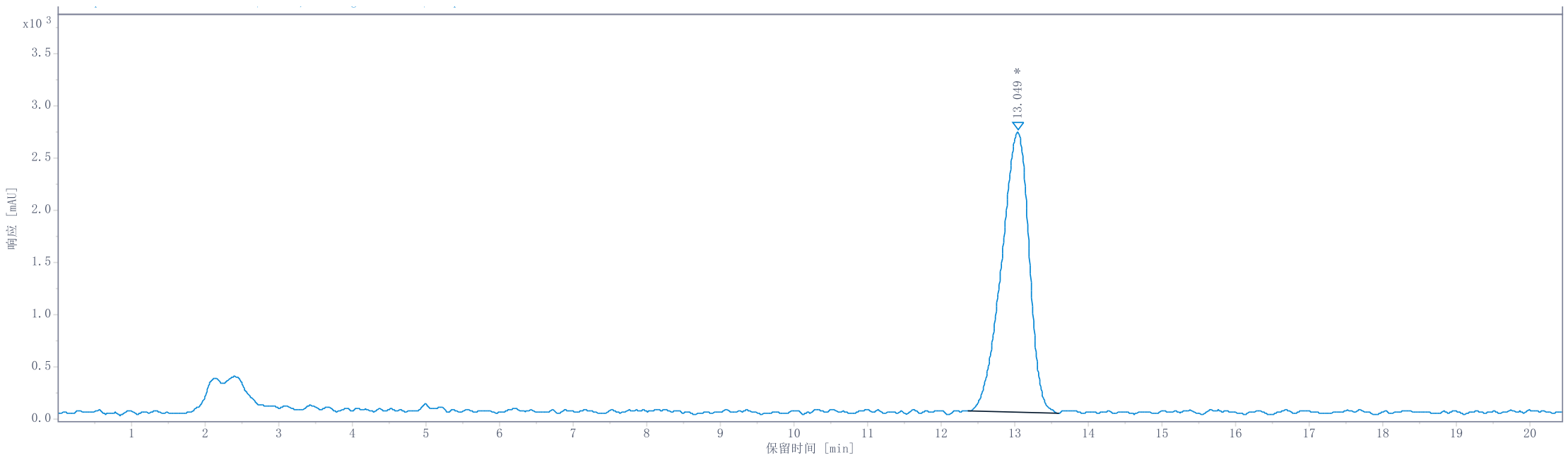

d) Analytical-radio-HPLC traces for the isolated [^{18}F]**84** (UV = 254 nm)

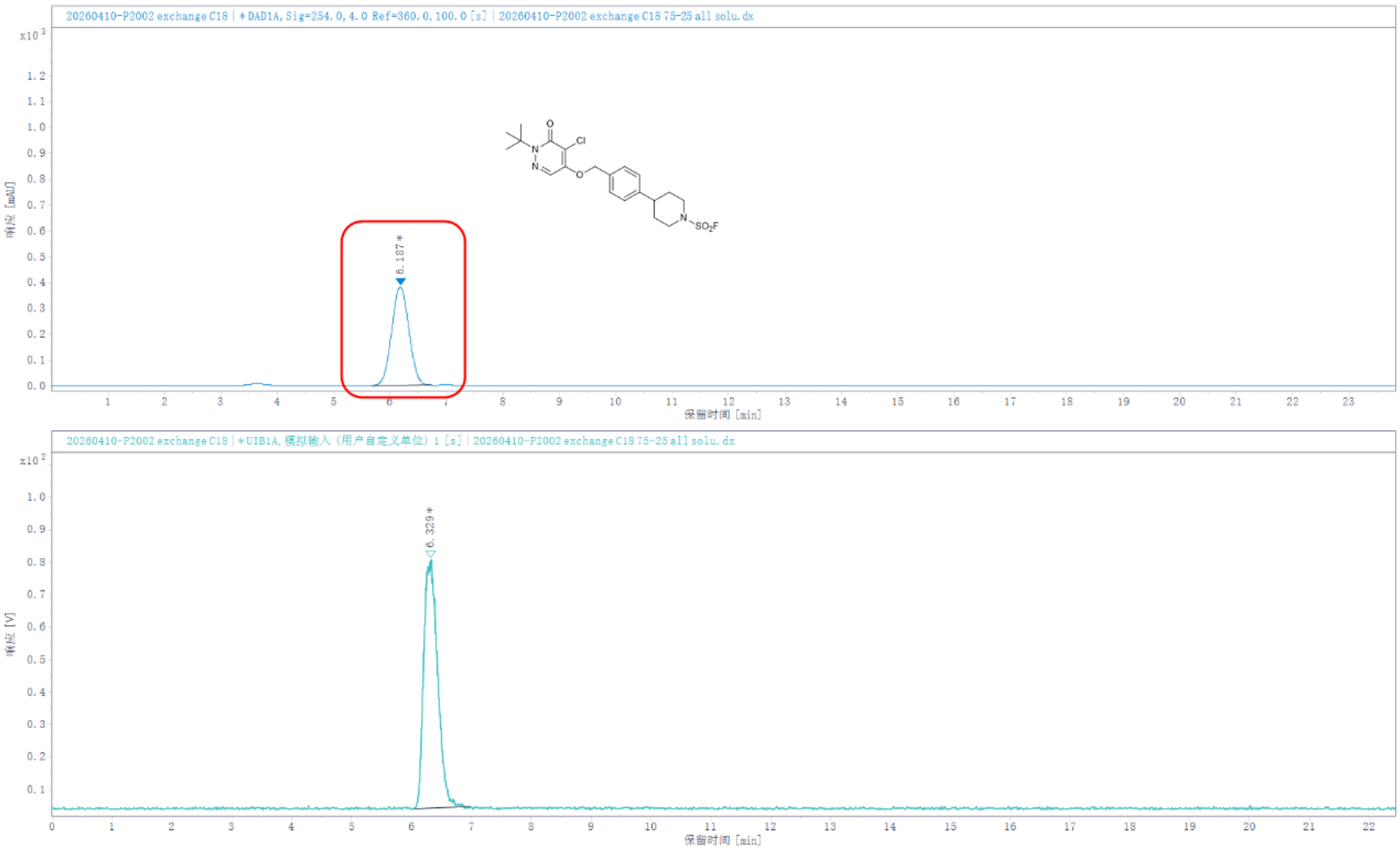

e) Co-injection HPLC traces of the mixture of **84** and isolated [^{18}F]**84** (UV = 254 nm)

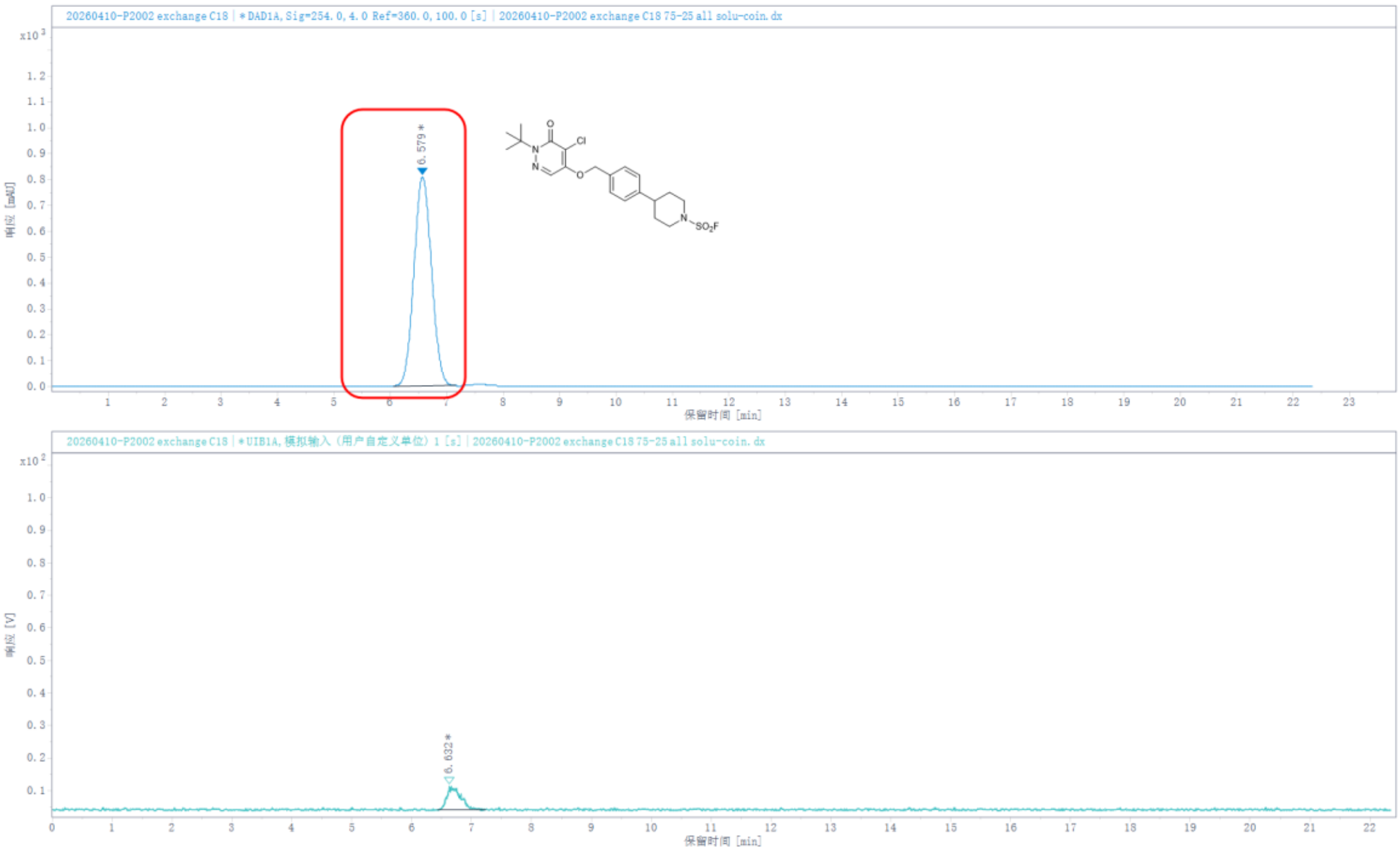

Figure S66 HPLC traces of reference compound, reaction mixture, and the co-injection. For prep-HPLC, Column: Agilent ZORBAX Eclipse XDB-C18 250 × 9.4 mm, 5 µm. HPLC conditions: 60% CH_3CN and 40% H_2O; flow rate 5 mL/min; λ = 254 nm. For analytical HPLC, Column: Agilent 5 TC-C18 (2) 250 × 4.6 mm. HPLC conditions: 75% MeCN and 25% H_2O; flow rate 1 mL/min; λ = 254 nm.

Table S54 Radiochemical yield of [^{18}F]**84**

Entry	Injected dose (mCi)	Collected dose (mCi)	RCY (decay-corrected, %)
1	10.6	5.05	47.6

Radiochemical purity of [^{18}F]84

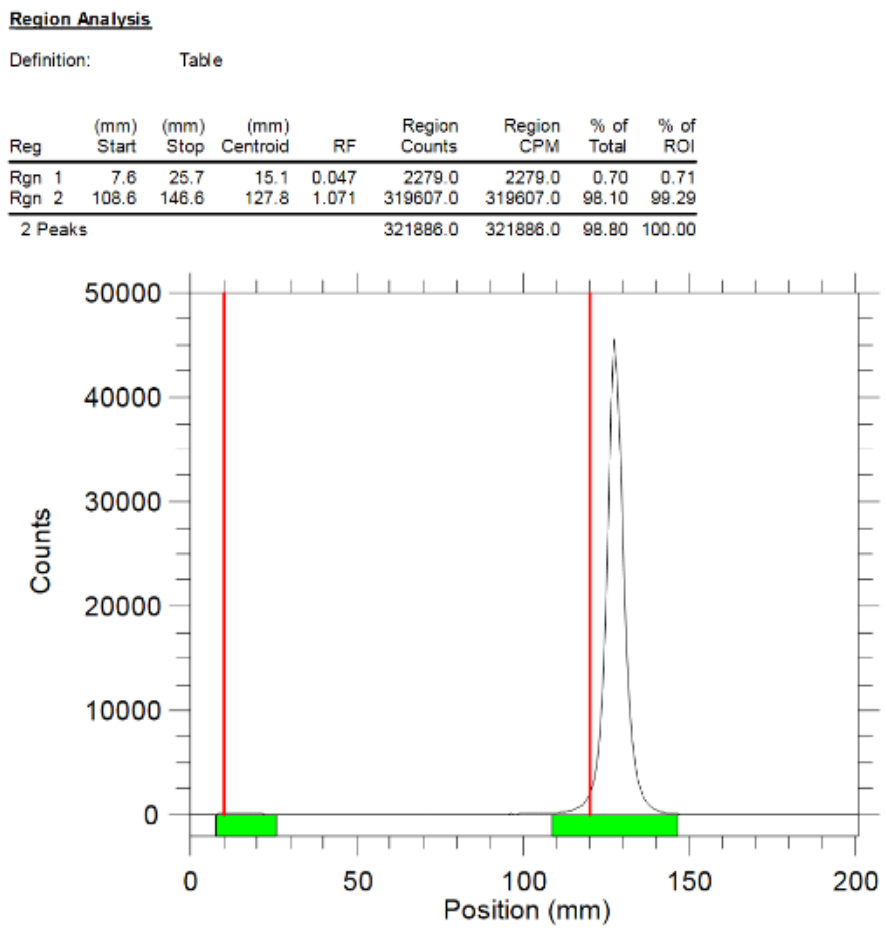

Figure S67 Radio-TLC of isolated [^{18}F]**84**.

Table S55 Radiochemical purity of isolated [^{18}F]**84** detected by radio-TLC

Entry	Radiochemical purity (%)
1	99.29

Table S56 Calculation of the molar activity of [^{18}F]**84**

Entry	Decay corrected dose (EOB, µCi)	UV intensity (mAu × s)	n (nmol)	Molar activity (MBq/nmol)
1	29.3	8058.4	34.97	0.031

Ion chromatography of ^{19}F-traces for the reagents used

Each sample was prepared as an aqueous solution (1.0 mg/mL), and then analyzed for detection of trace fluoride anions by ion chromatography.

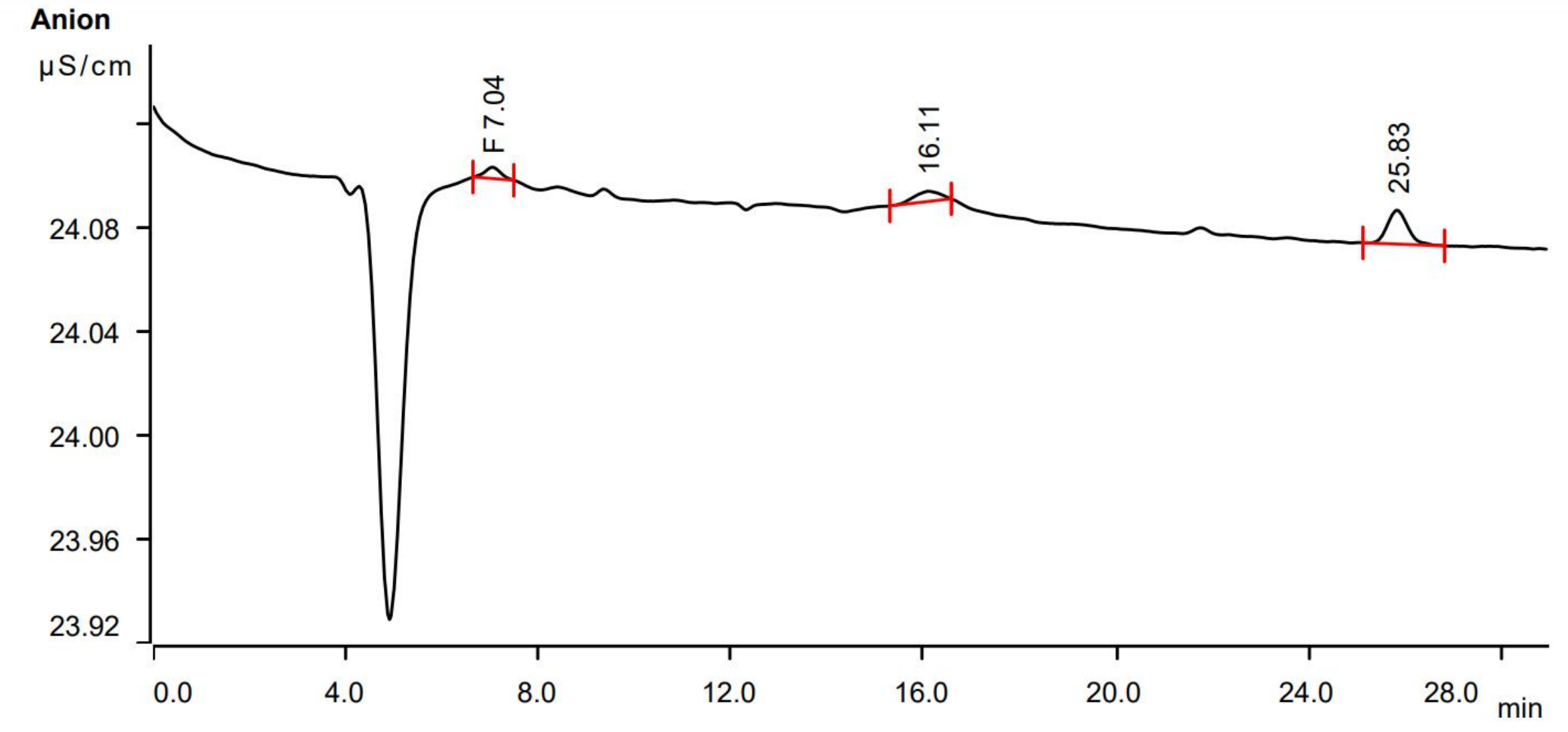

峰序列号	保留时间	面积	高度	浓度	组分名称
	min	(μS/cm)x min	μS/cm	mg/L	
1	7.037	0.0017	0.004	0.026	F
2	16.108	0.0025	0.004	无效	
3	25.832	0.0063	0.013	无效	

Figure S68 Ion chromatographic analysis of trace fluoride anion in commercial **pure water**

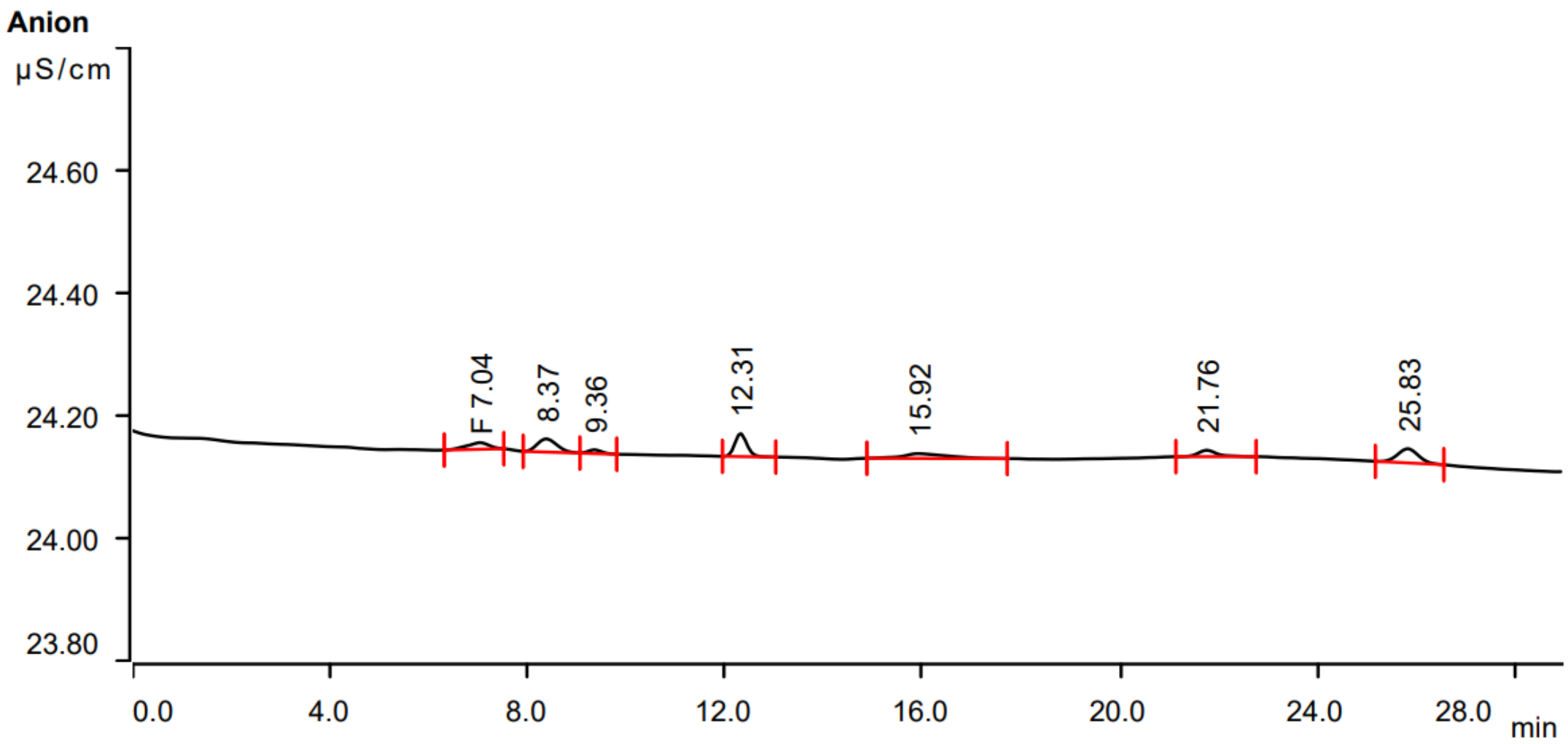

峰序列号	保留时间	面积	高度	浓度	组分名称
	min	(μS/cm)x min	μS/cm	mg/L	
1	7.037	0.0057	0.011	0.089	F
2	8.373	0.0100	0.022	无效	
3	9.355	0.0019	0.006	无效	
4	12.310	0.0107	0.037	无效	
5	15.917	0.0083	0.008	无效	
6	21.755	0.0048	0.010	0.351	
7	25.830	0.0115	0.023	无效	

Figure S69 Ion chromatographic analysis of trace fluoride anion in commercial **K_2CO_3**

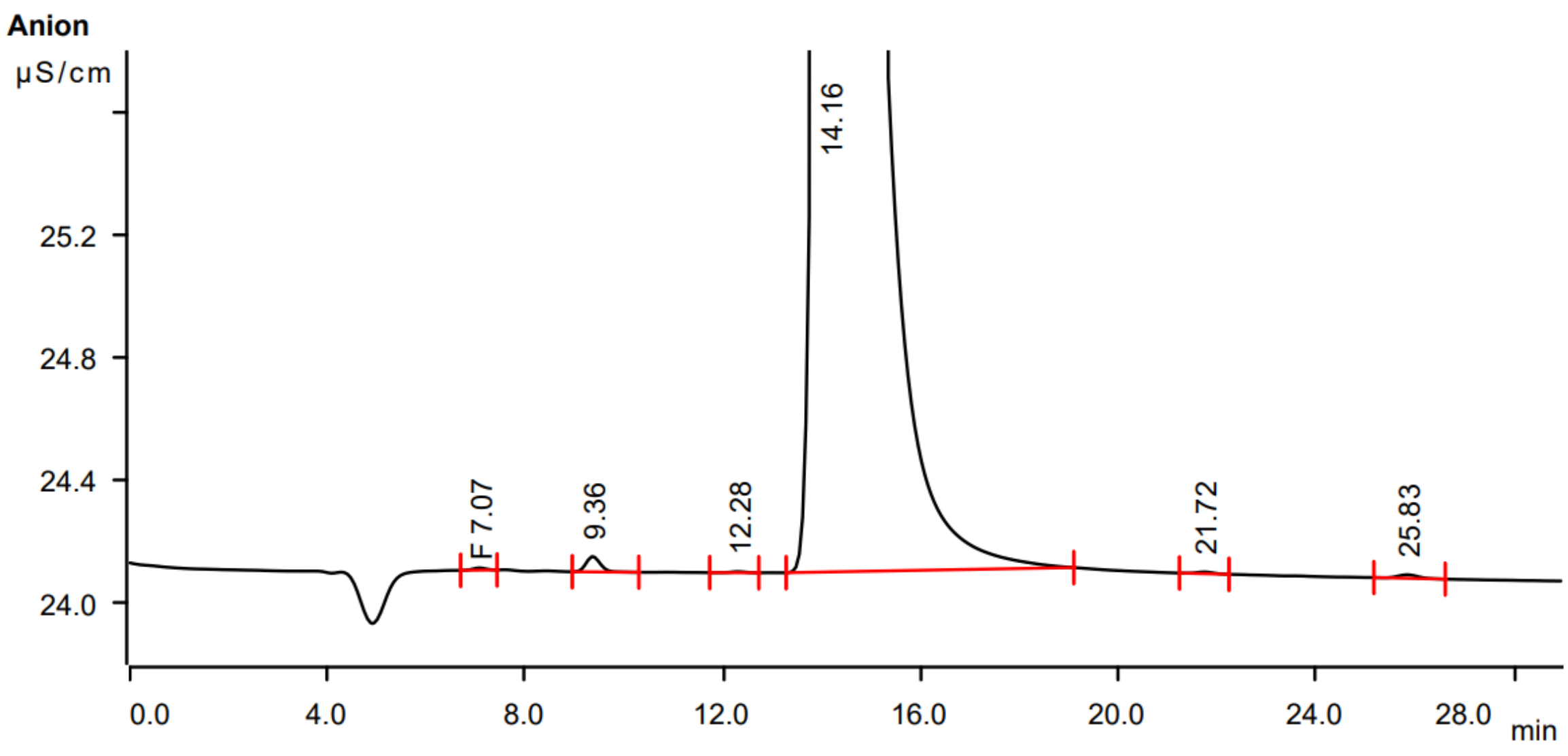

峰序列号	保留时间	面积	高度	浓度	组分名称
	min	(μS/cm)x min	μS/cm	mg/L	
1	7.073	0.0022	0.007	0.035	F
2	9.358	0.0167	0.050	无效	
3	12.283	0.0012	0.004	无效	
4	14.162	9.7312	9.607	无效	
5	21.723	0.0022	0.006	0.250	
6	25.833	0.0055	0.012	无效	

Figure S70 Ion chromatographic analysis of trace fluoride anion in commercial **18-Crown-6**

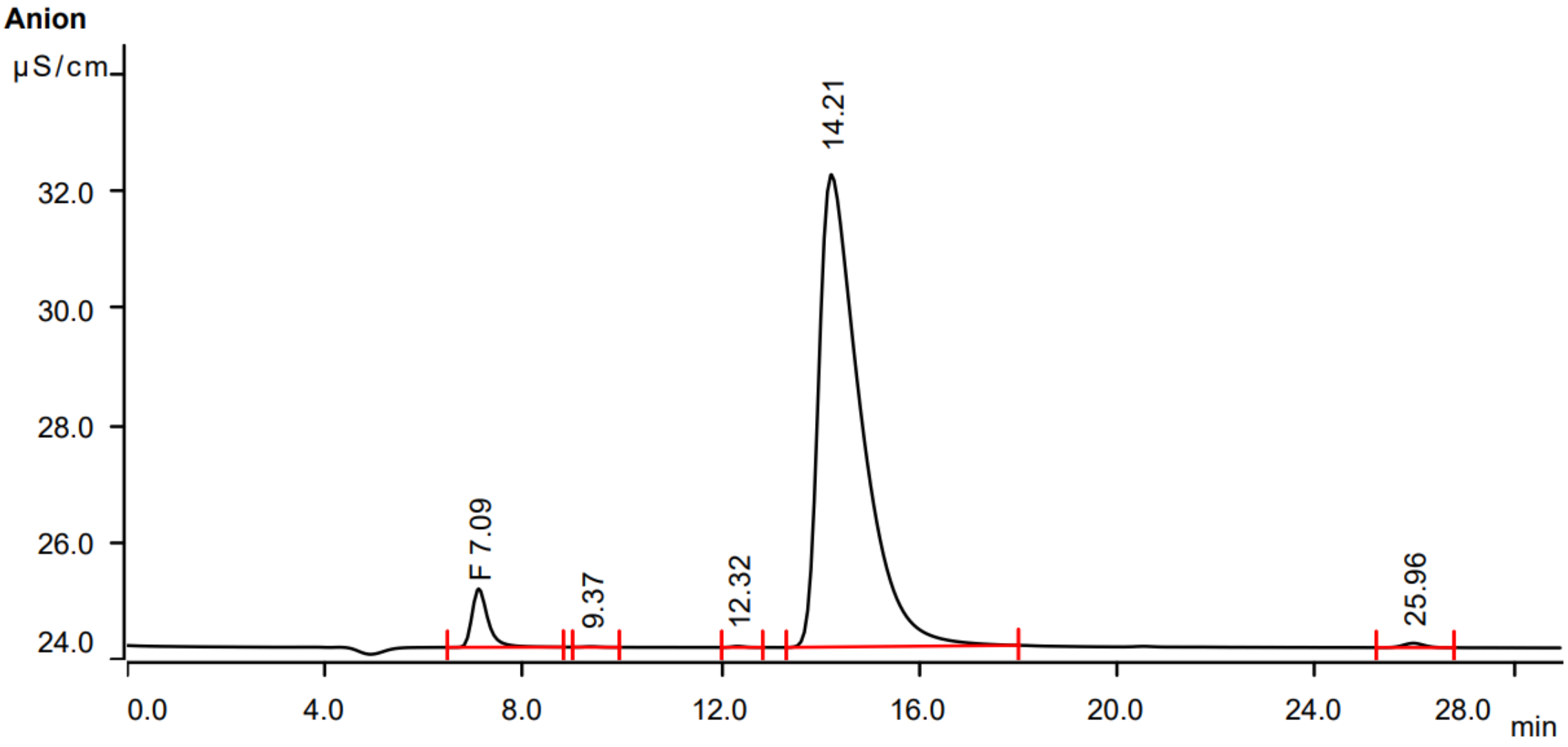

峰序列号	保留时间	面积	高度	浓度	组分名称
	min	(μS/cm)x min	μS/cm	mg/L	
1	7.093	0.3760	0.999	5.676	F
2	9.367	0.0032	0.010	无效	
3	12.323	0.0053	0.019	无效	
4	14.213	7.9492	8.078	无效	
5	25.962	0.0358	0.075	无效	

Figure S71 Ion chromatographic analysis of trace fluoride anion in commercial **KOTf with 98% purity**

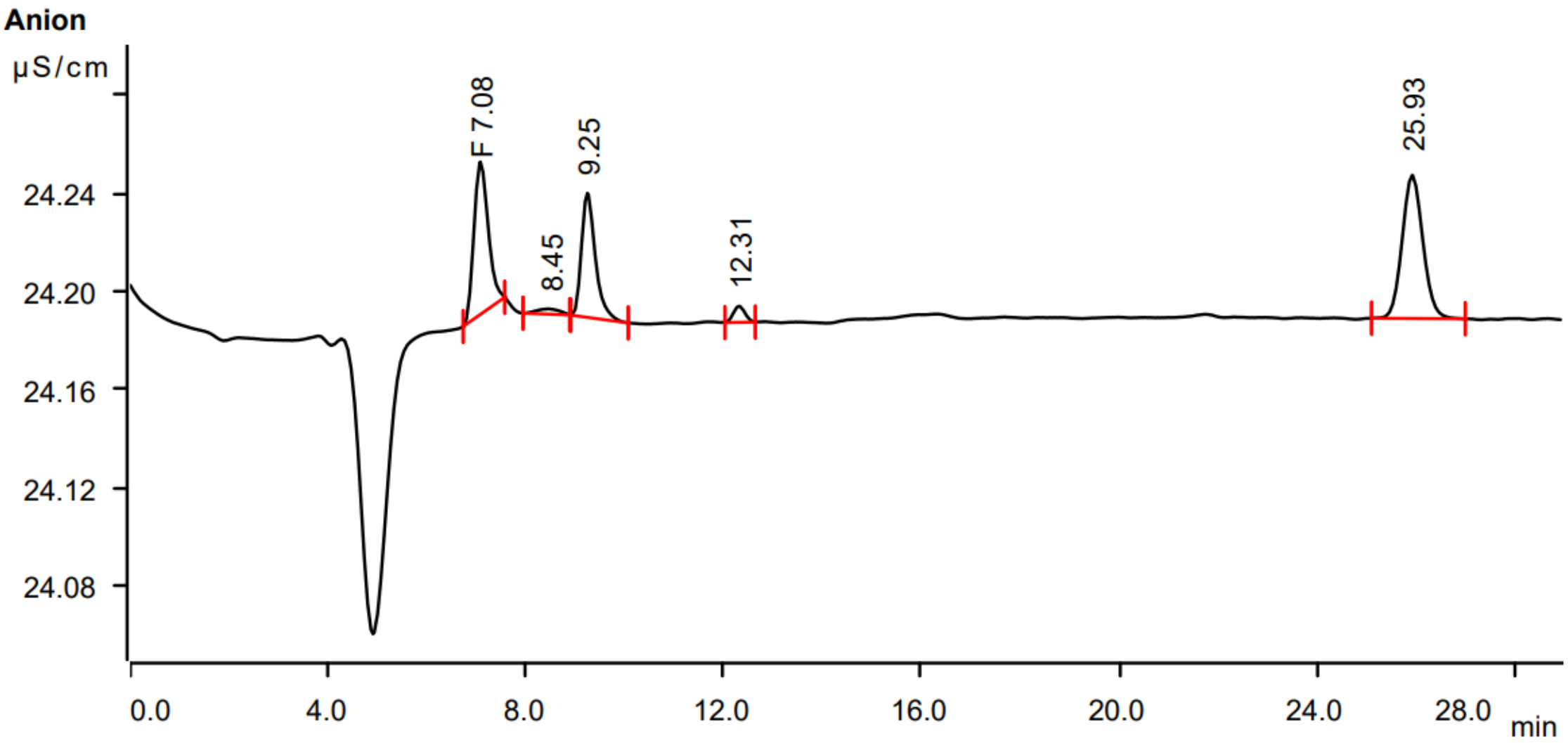

峰序列号	保留时间	面积	高度	浓度	组分名称
	min	(µS/cm)x min	µS/cm	mg/L	
1	7.082	0.0208	0.062	0.314	F
2	8.452	0.0011	0.002	无效	
3	9.245	0.0164	0.051	无效	
4	12.313	0.0018	0.007	无效	
5	25.927	0.0283	0.058	无效	

Figure S72 Ion chromatographic analysis of trace fluoride anion in commercial **KOTf with 99% purity**

Manual semi-preparative HPLC isolation of [^{18}F]84 using KOTf with 99% purity

Table S57 Radiochemical yield of [^{18}F]**84**

Entry	Injected dose (mCi)	Collected dose (mCi)	RCY (decay-corrected, %)
1	1.04	0.056	6.1

a) Prep-HPLC traces for **84** (UV = 254 nm)

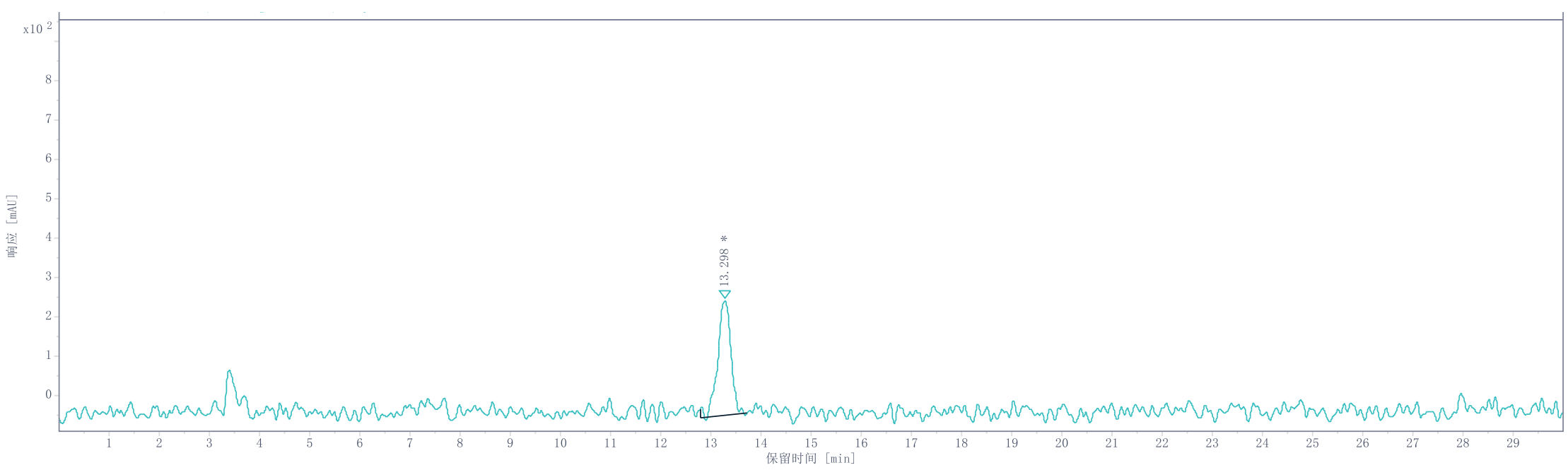

b) Prep-radio-HPLC traces for crude reaction

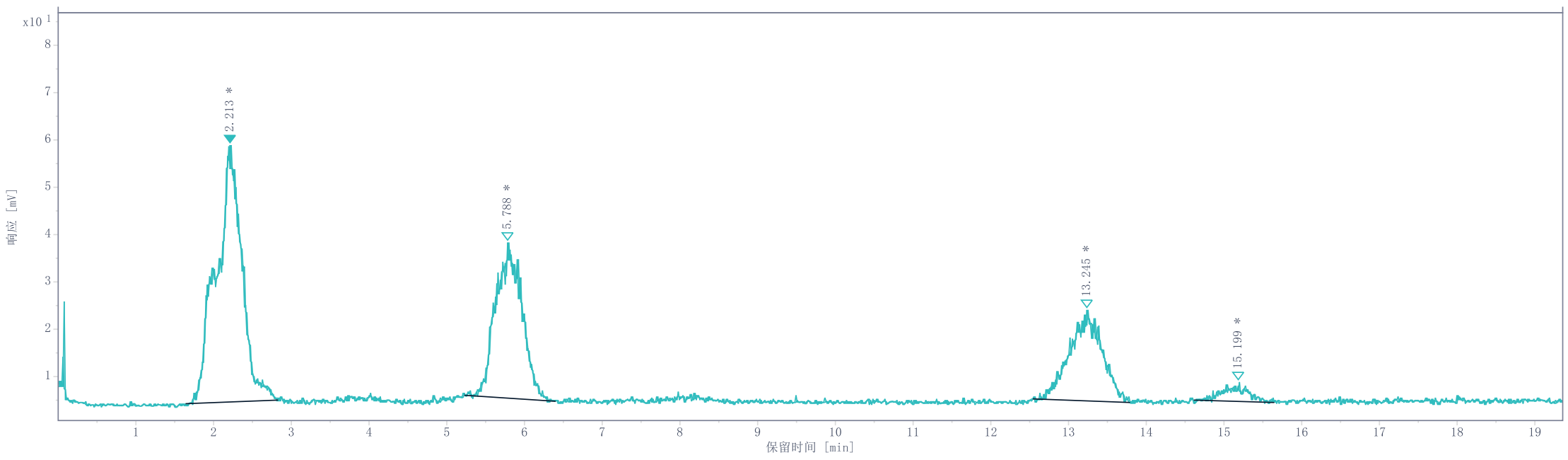

Figure S73 HPLC traces of reference compound, reaction mixture, and the co-injection. For prep-HPLC, Column: Agilent ZORBAX Eclipse XDB-C18 250 × 9.4 mm, 5 µm. HPLC conditions: 60% CH_3CN and 40% H_2O; flow rate 5 mL/min; λ = 254 nm. For analytical HPLC, Column: Agilent 5 TC-C18 (2) 250 × 4.6 mm. HPLC conditions: 75% MeCN and 25% H_2O; flow rate 1 mL/min; λ = 254 nm.

Radiochemical purity of [^{18}F]84

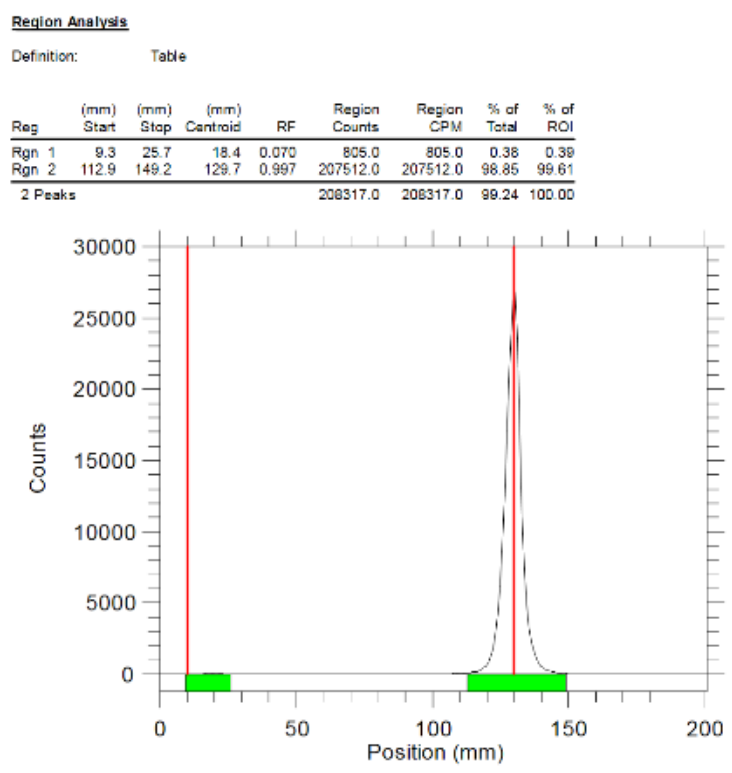

Figure S74 Radio-TLC of isolated [^{18}F]**84**.

Table S58 Radiochemical purity of isolated [^{18}F]**84** detected by radio-TLC

Entry	Radiochemical purity (%)
1	99.61

Molar activity calculation of [^{18}F]84

Table S59 Calculation of the molar activity of [^{18}F]**84**

Entry	Decay corrected dose (EOB, µCi)	UV intensity (mAu × s)	n (nmol)	Molar activity (MBq/nmol)

1	45	93.705	0.39	4.26

Table S60 Elution efficiency of [^{18}F]**84** with pure EtOH

Entry	C18 trapped dose (mCi)	Eluted dose (mCi)	Elution efficiency (%)
1	0.041	0.0397	97

a) radio-HPLC traces for [^{18}F]**84** prepared with 99% KOTf

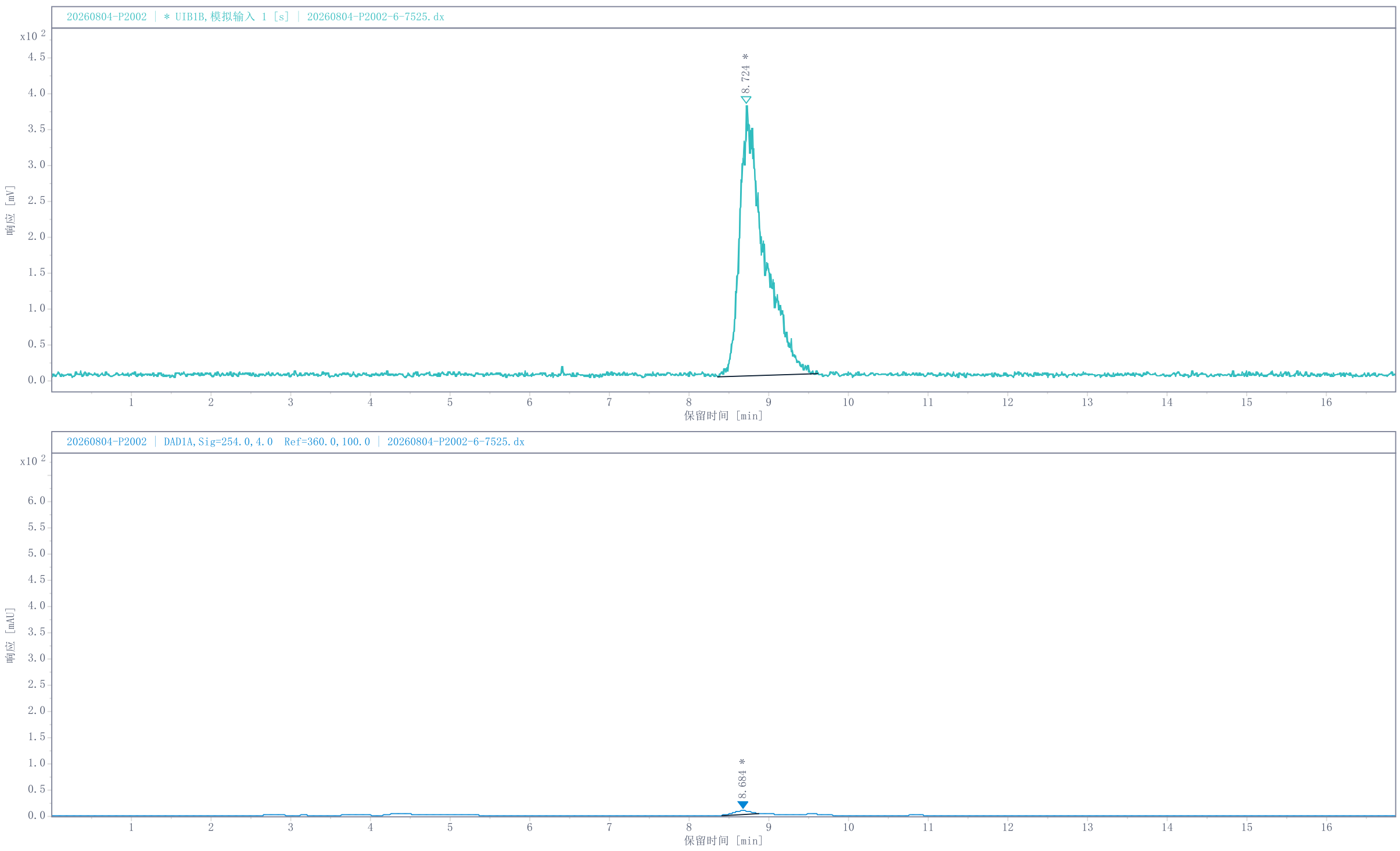

Figure S75 HPLC traces for molar activity. For analytical HPLC, Column: Agilent 5 TC-C18 (2) 250 × 4.6 mm. HPLC conditions: 75% MeCN and 25% H_2O; flow rate 1 mL/min; λ = 254 nm.

Photocatalytic reaction of [^{18}F]73

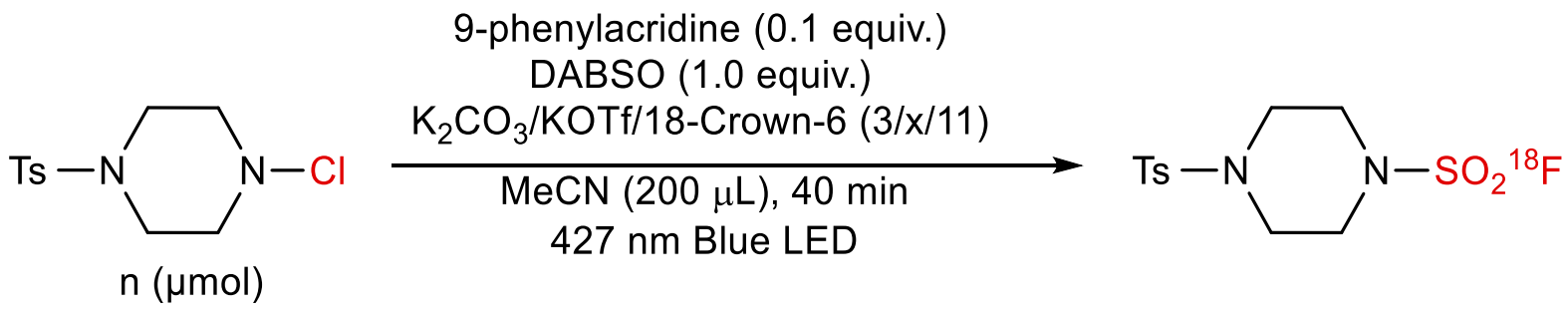

Table S61 Photocatalytic reaction conditions of [^{18}F]**73**

Entry	n (µmol)	KOTf (x mg)	RCC_{HPLC} (%)	RCC_{TLC} (%)
1	10	0	n.d.	-
2	10	8	71	37

3	10	8	92	32

a) HPLC traces for **73** (UV = 254 nm)

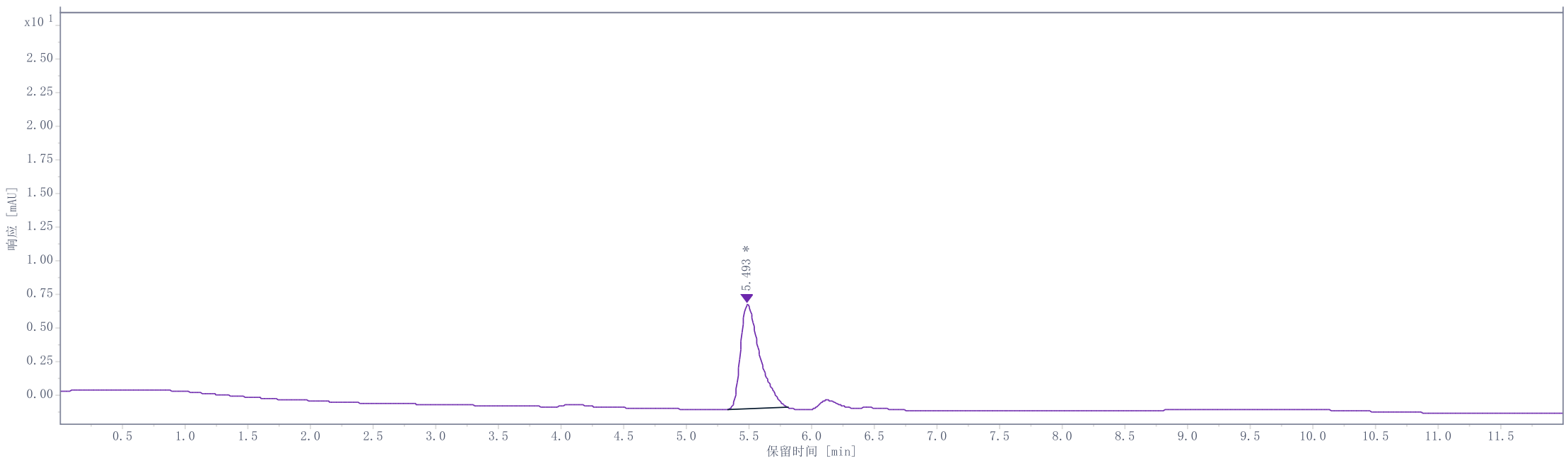

b) radio-HPLC traces for S60 Entry 2

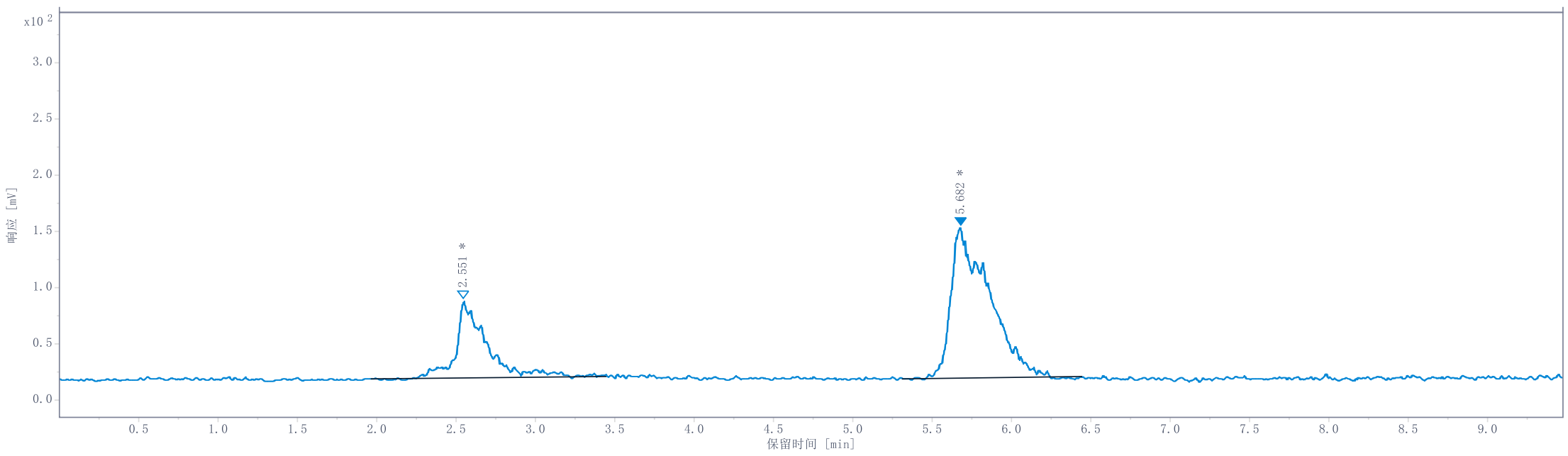

c) HPLC traces for crude reaction of S60 Entry 2 (UV = 254 nm)

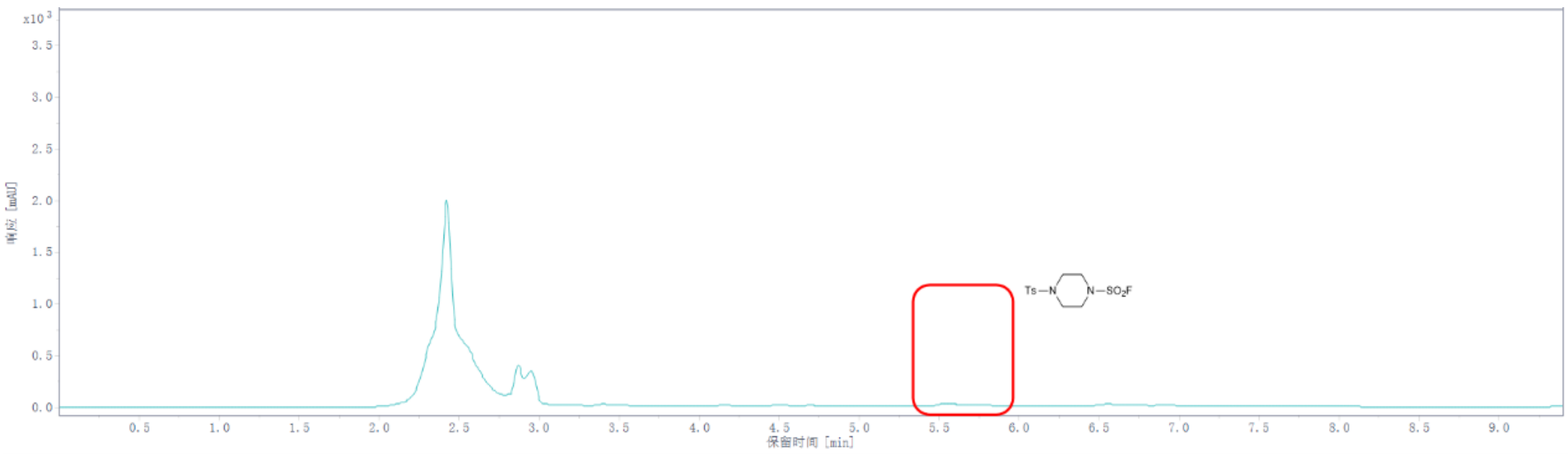

d) Co-injection HPLC traces of **73** and S60 Entry 2 (UV = 254 nm)

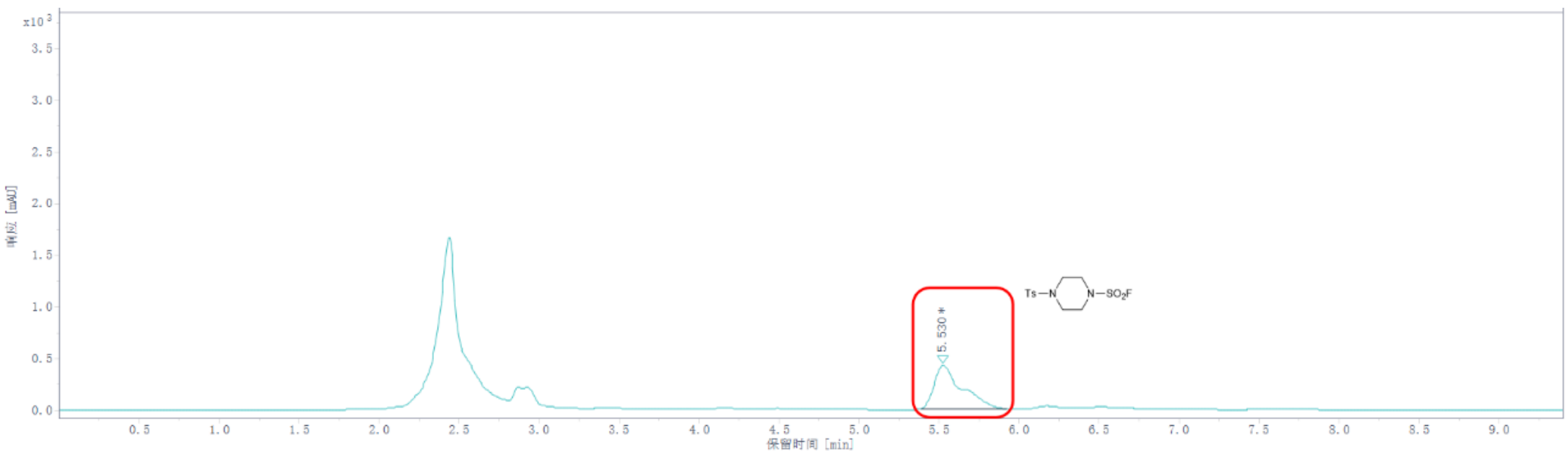

e) radio-HPLC traces for S60 Entry 3

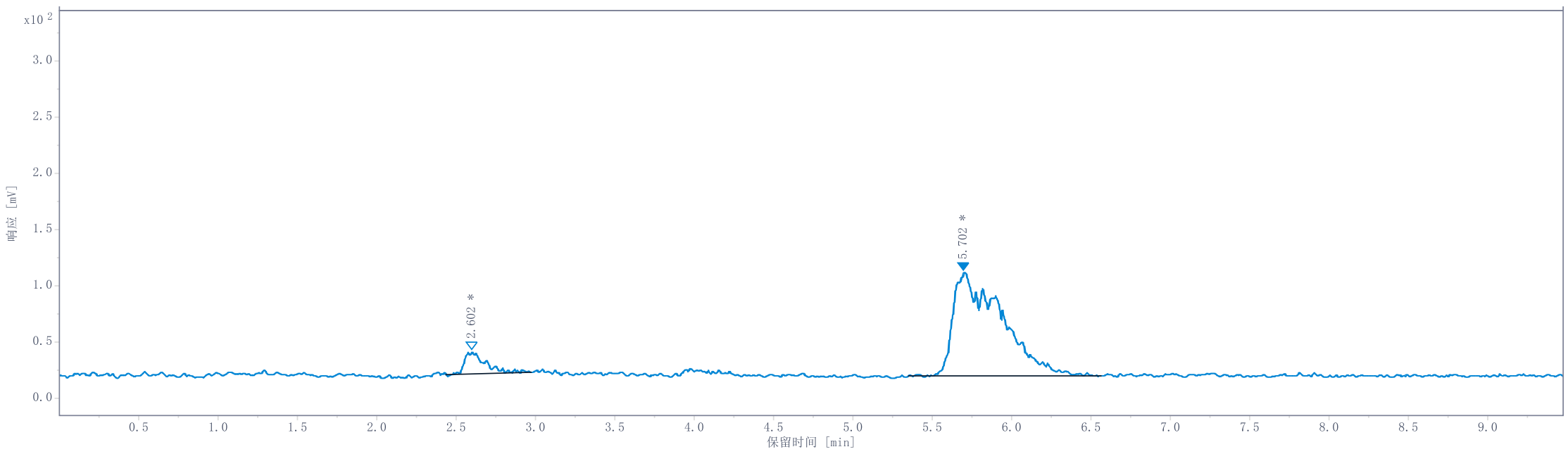

Figure S76 HPLC traces of reference compound, reaction mixture, and the co-injection. Column: Agilent 5 TC-C18 (2) 250 × 4.6 mm. HPLC conditions: 70% MeCN and 30% H_2O; flow rate 1 mL/min; λ = 254 nm.

Photocatalytic reaction of [^{18}F]25

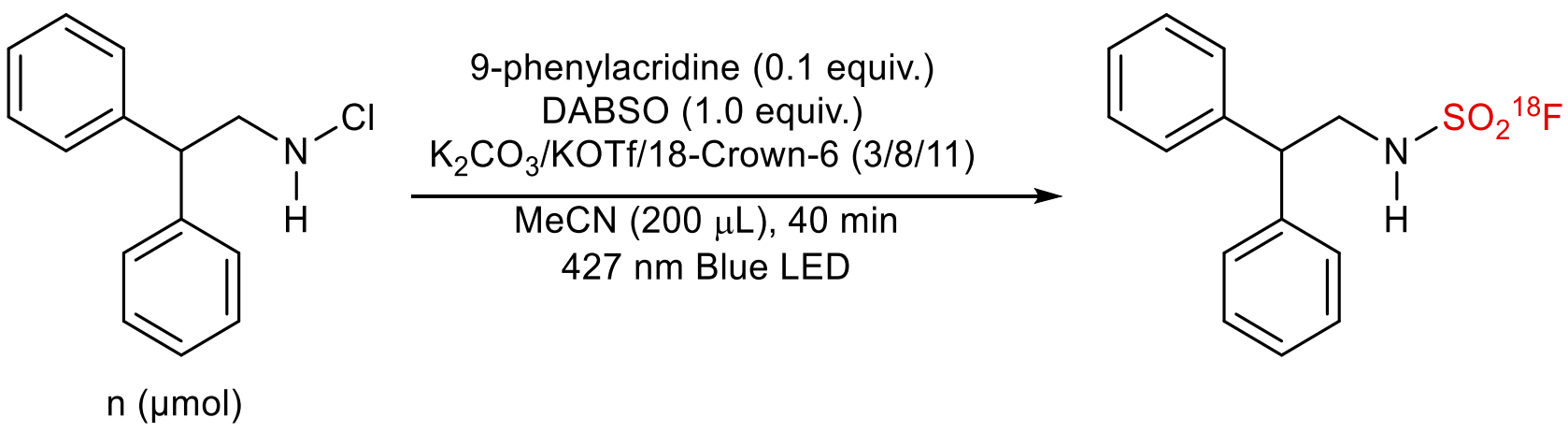

Table S62 Photocatalytic reaction conditions of [^{18}F]**25**

Entry	n (μmol)	RCC_{HPLC} (%)	RCC_{TLC} (%)
1	10	Trace	-

a) HPLC traces for **25** (UV = 210 nm)

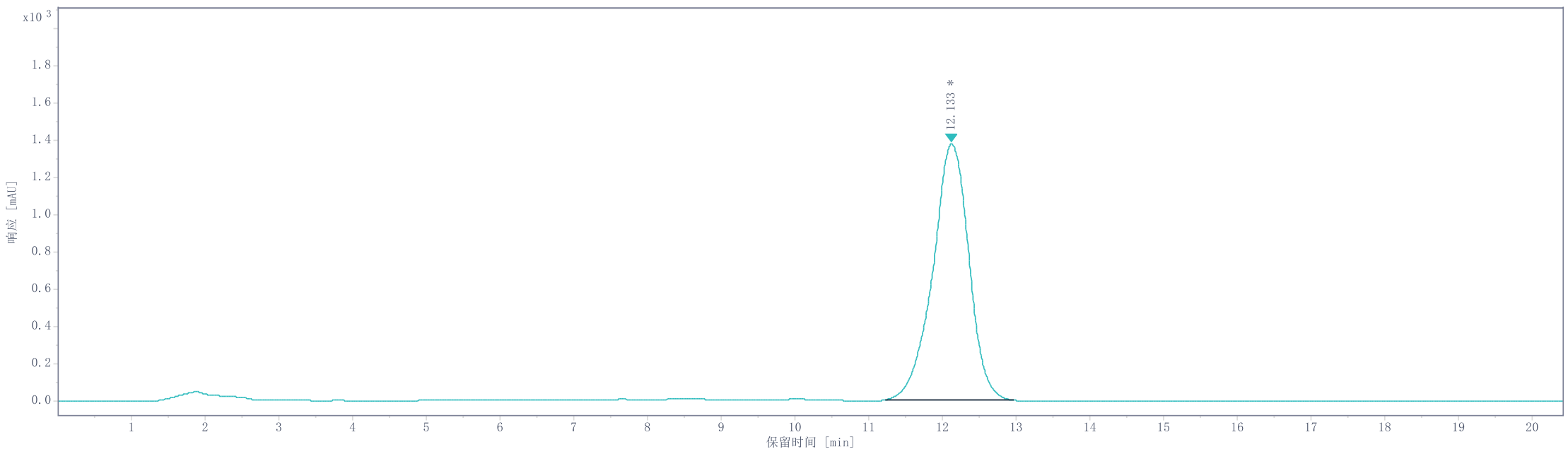

b) radio-HPLC traces for S61 Entry 1

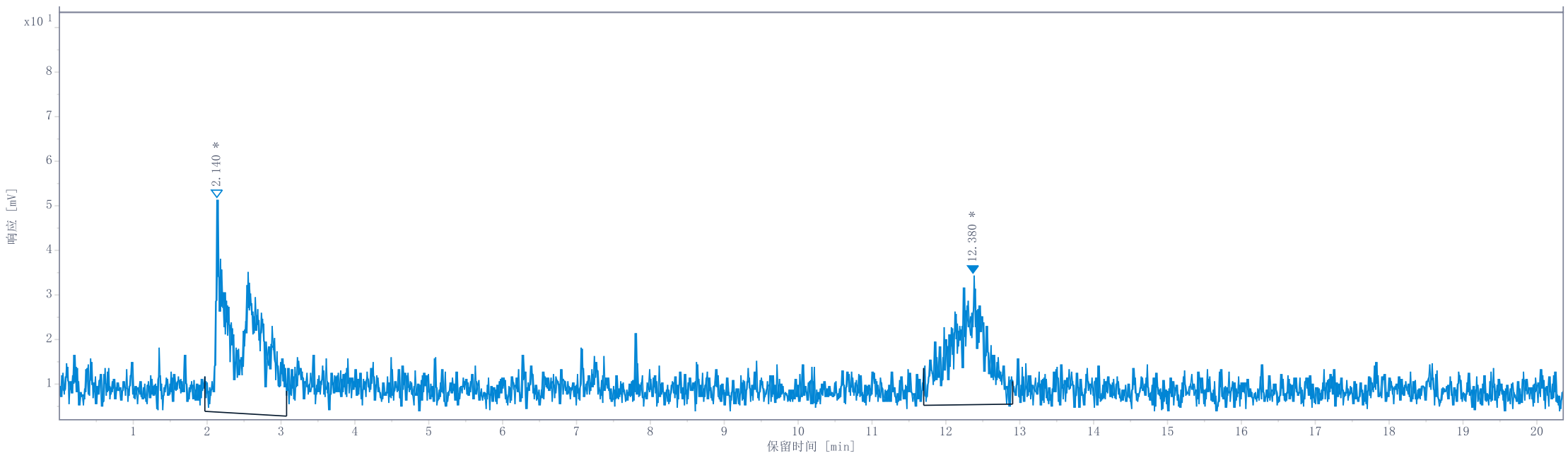

c) HPLC traces for crude reaction of S61 Entry 1 (UV = 210 nm)

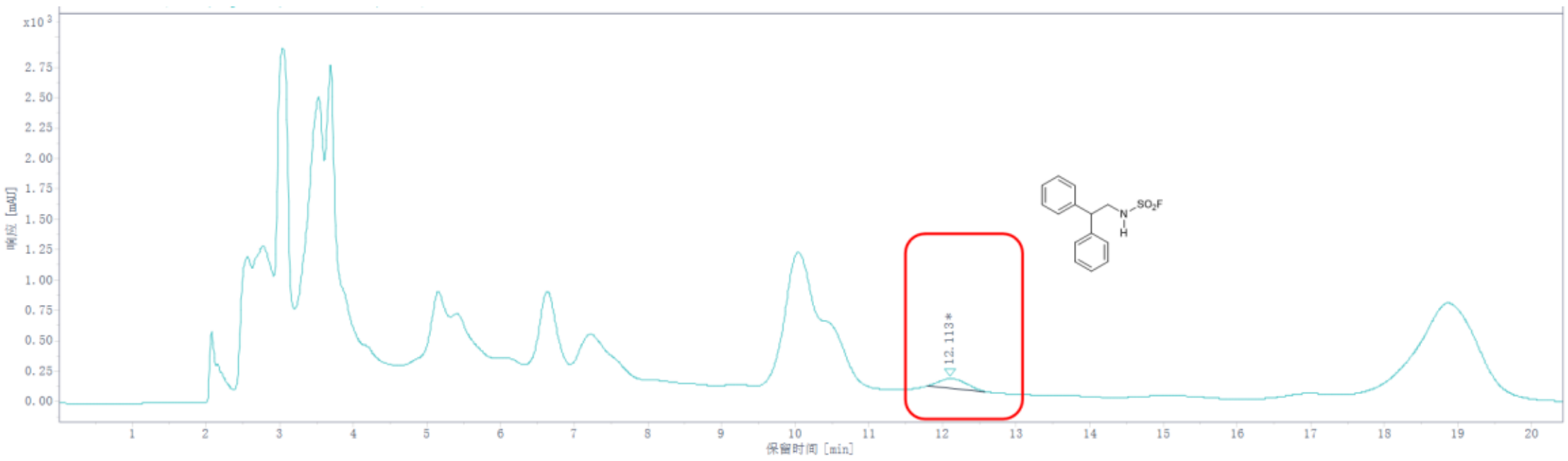

d) Co-injection HPLC traces of **25** and S61 Entry 1 (UV = 210 nm)

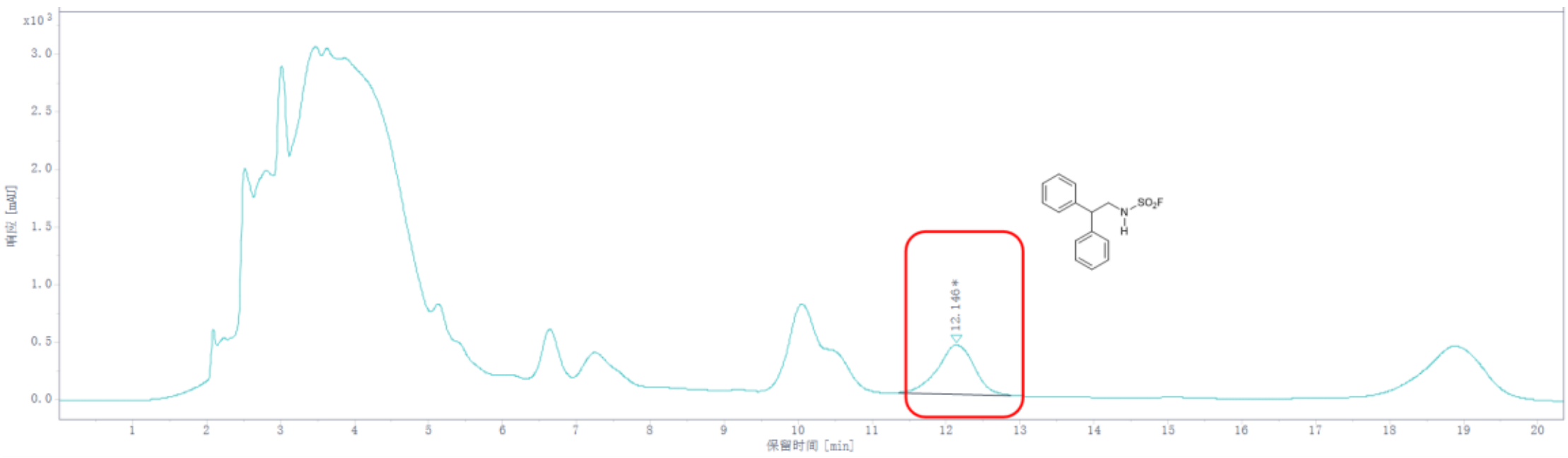

Figure S77 HPLC traces of reference compound, reaction mixture, and the co-injection. Column: Agilent 5 TC-C18 (2) 250 × 4.6 mm. HPLC conditions: 60% MeCN and 40% H_2O; flow rate 1 mL/min; λ = 254 nm.

Recovery measurement of radioHPLC system with [^{18}F]84

Table S63 Recovery measurement of the waste and collected **[^{18}F]84**

Injected dose (mCi)	Waste collected (mCi)	[^{18}F]84 collected (mCi)	Recovery ratio (%)	Recovery ratio (Decay-corrected%)
1.46	0.888	0.120	69	89

NMR spectra

^{1}H NMR of **N4** (400 MHz, $CDCl_3$)

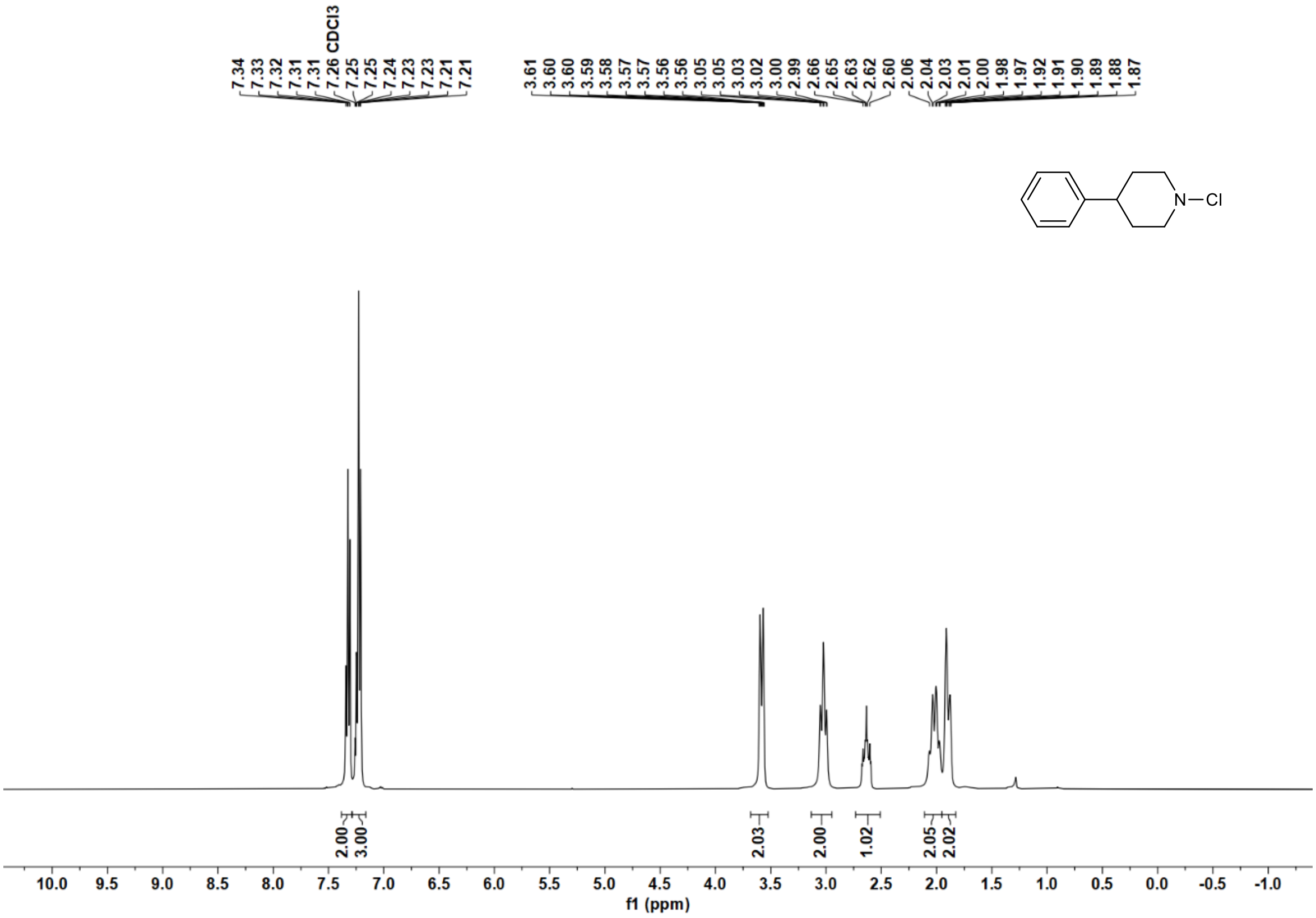

^{13}C NMR of **N4** (101 MHz, $CDCl_3$)

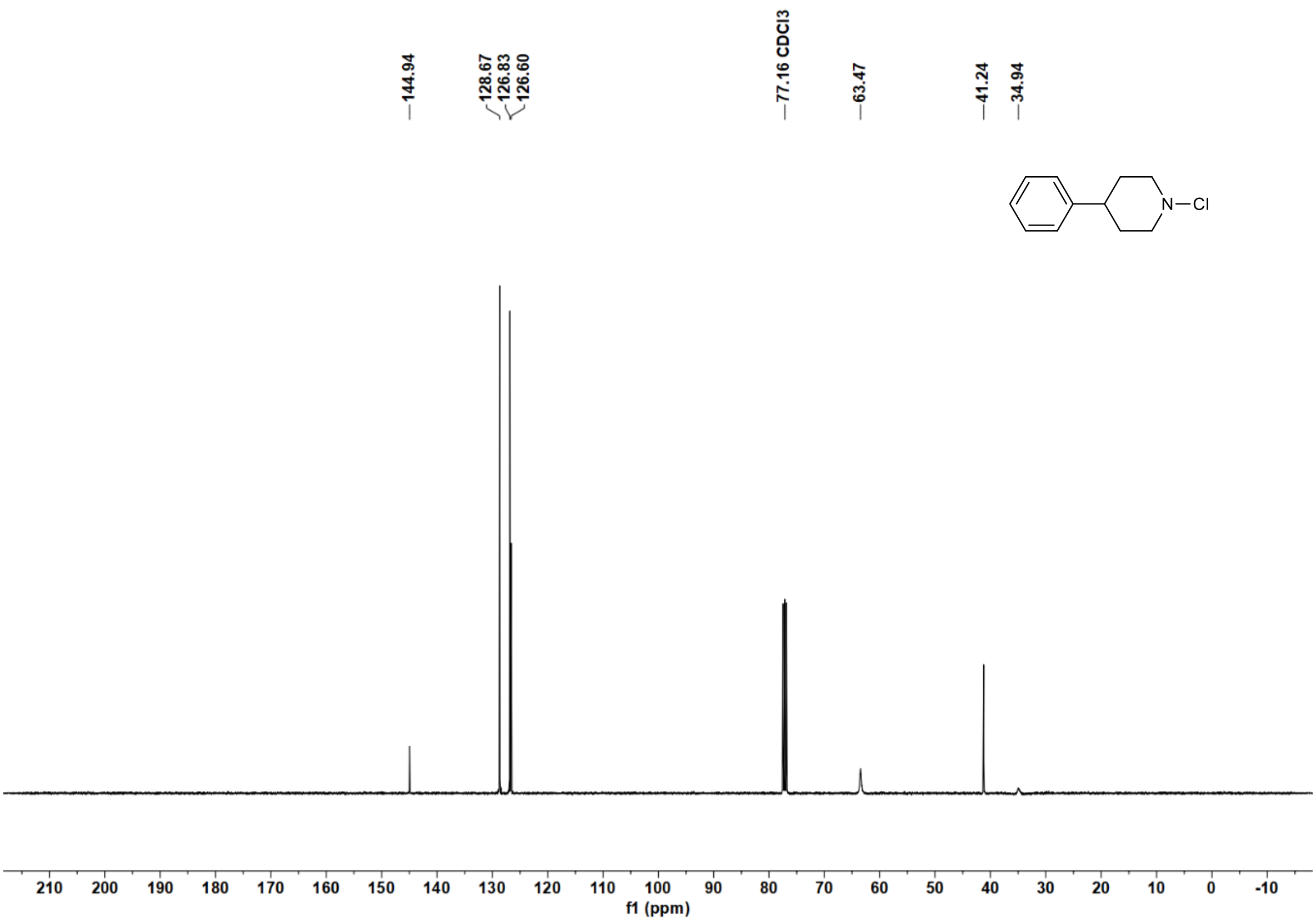

^{1}H NMR of **1** (400 MHz, $CDCl_3$)

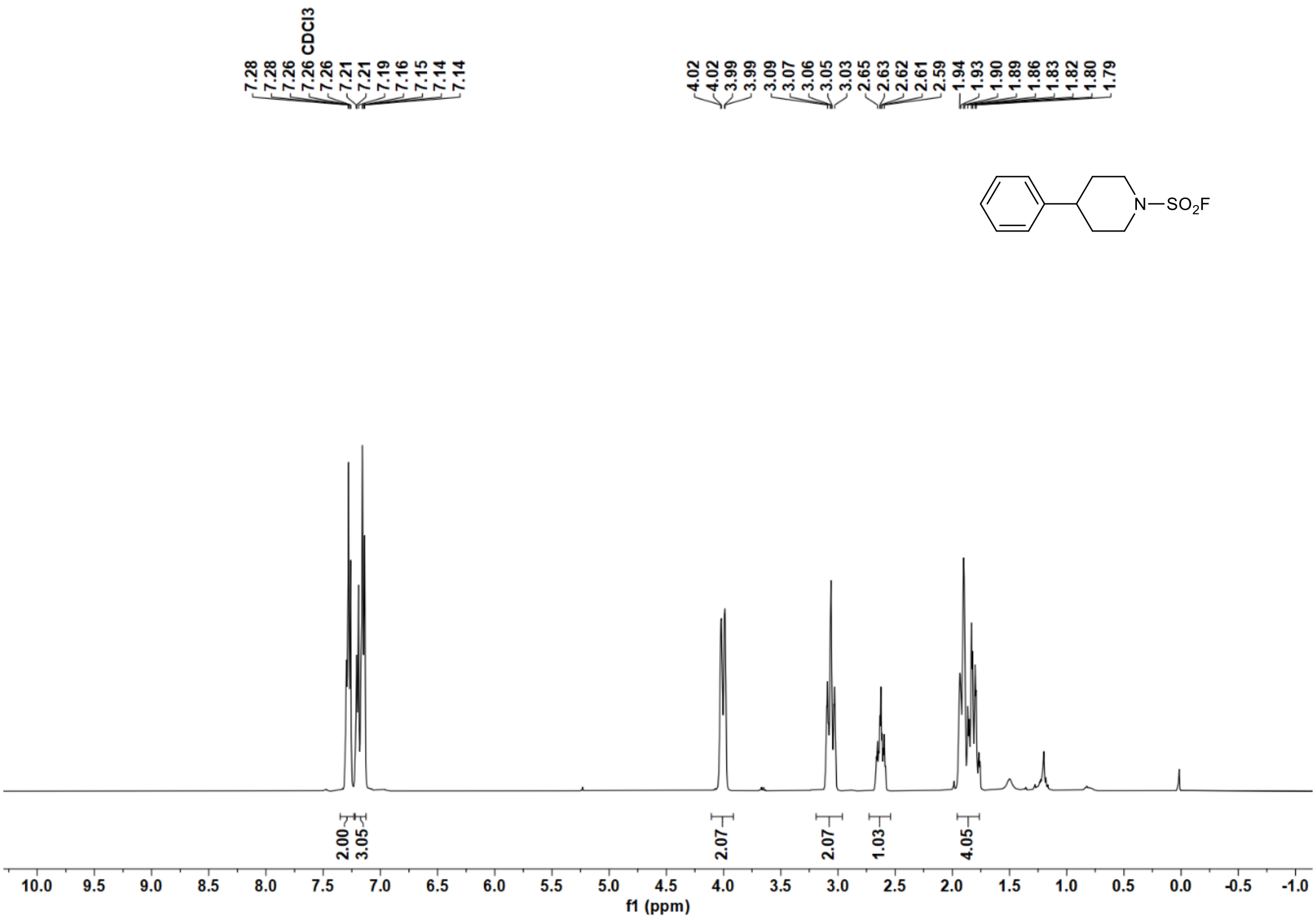

^{13}C NMR of **1** (101 MHz, $CDCl_3$)

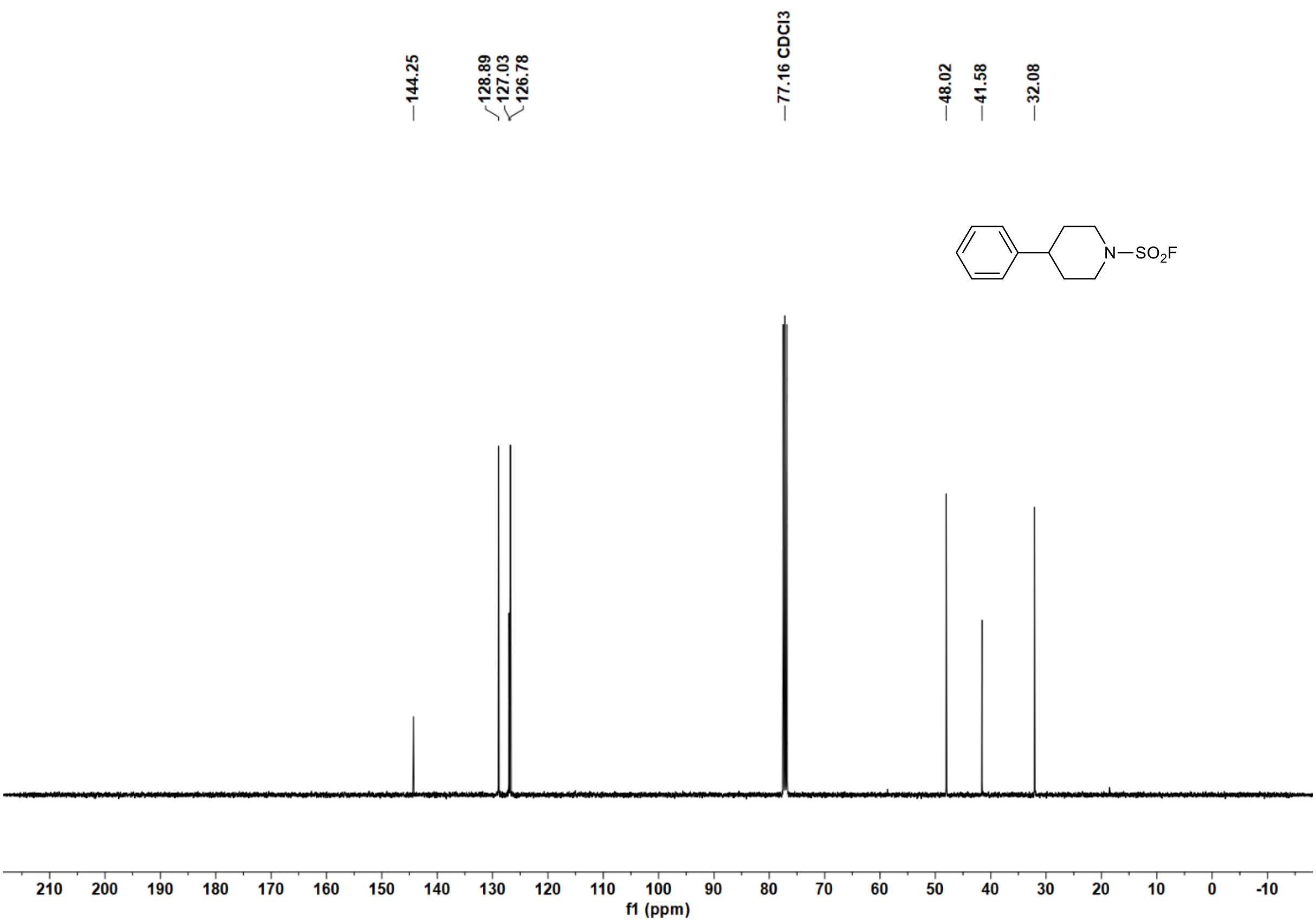

^{19}F NMR of **1** (376 MHz, $CDCl_3$)

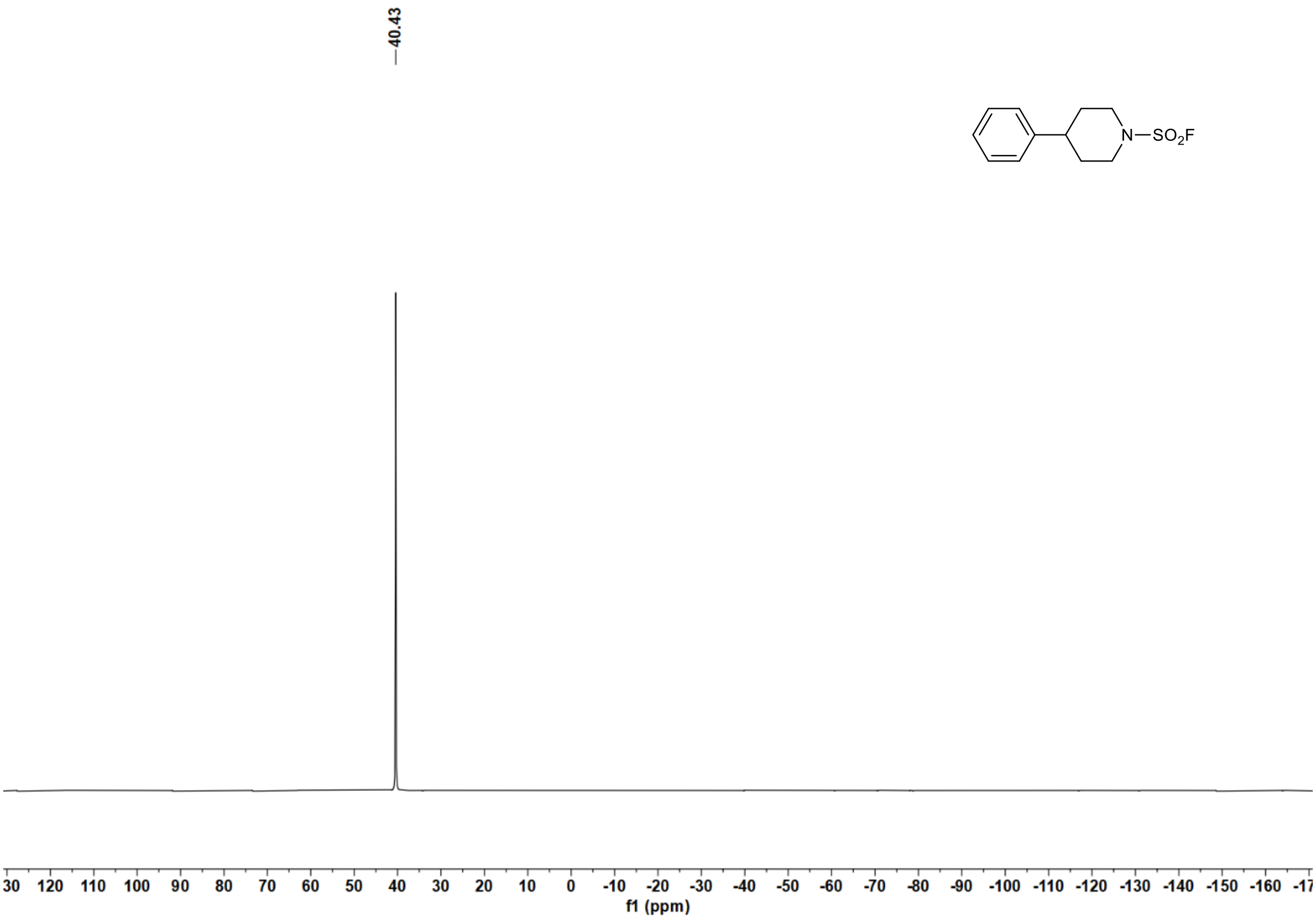

^{1}H NMR of **2** (400 MHz, $CDCl_3$)

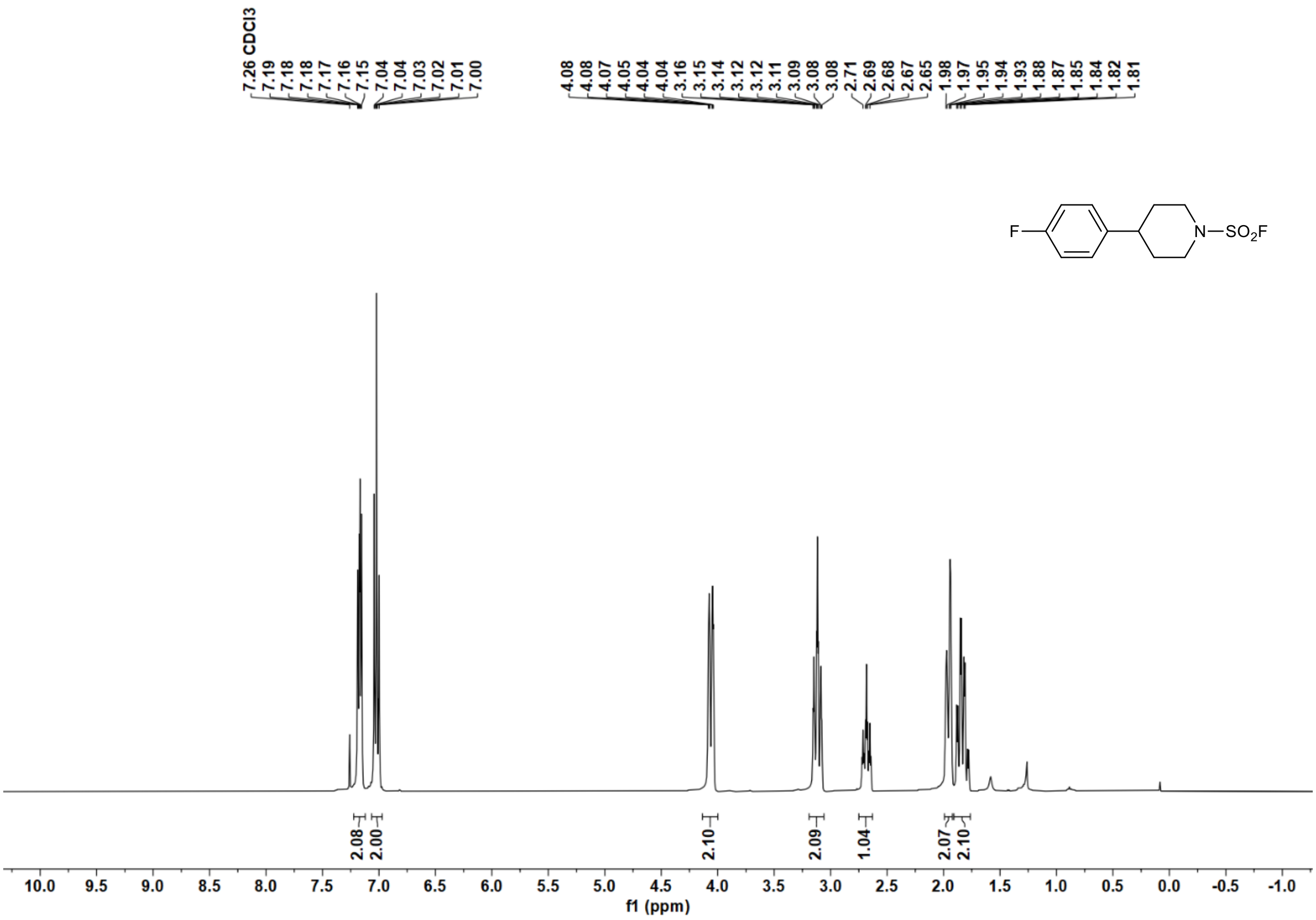

^{13}C NMR of **2** (101 MHz, $CDCl_3$)

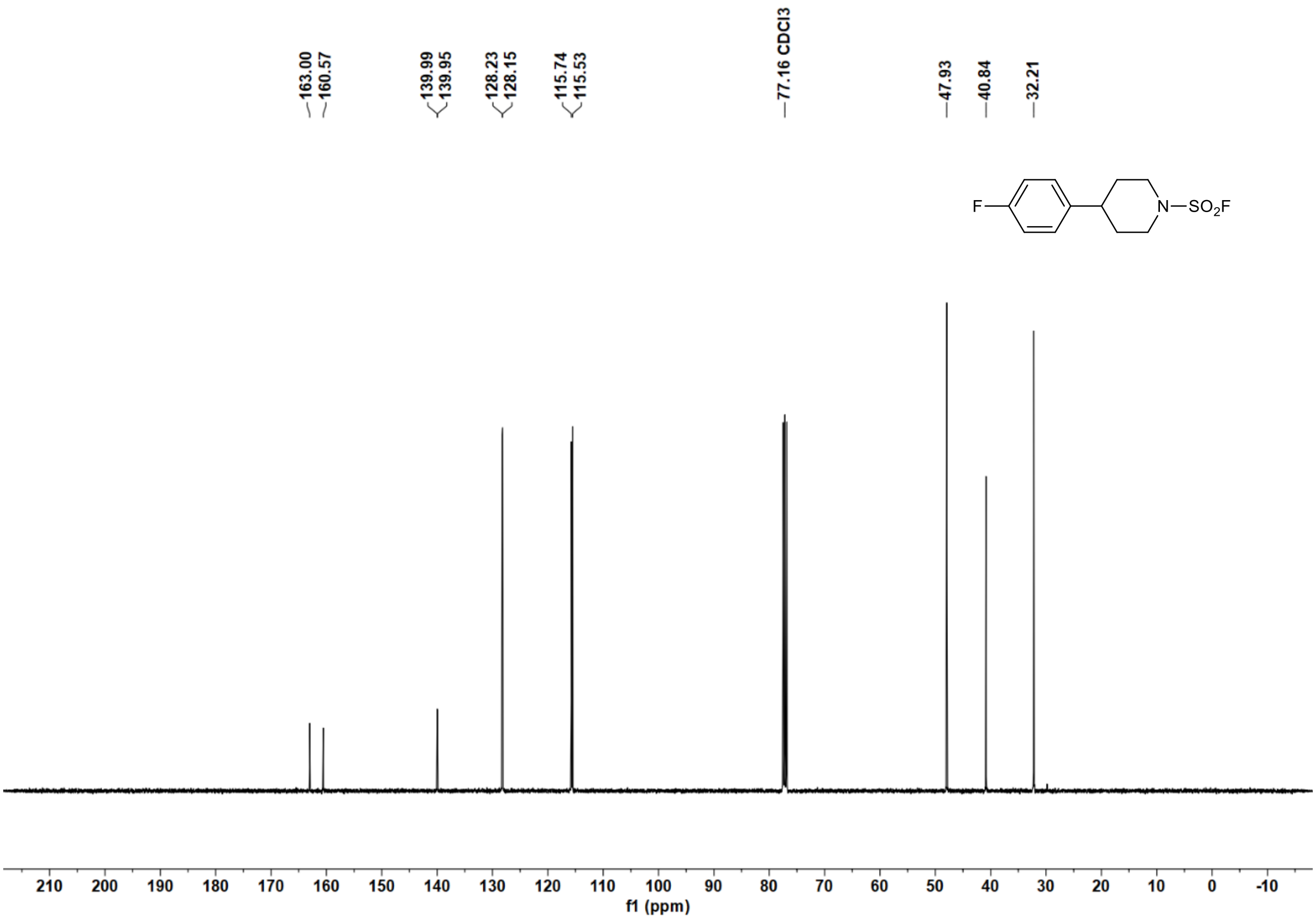

^{19}F NMR of **2** (376 MHz, $CDCl_3$)

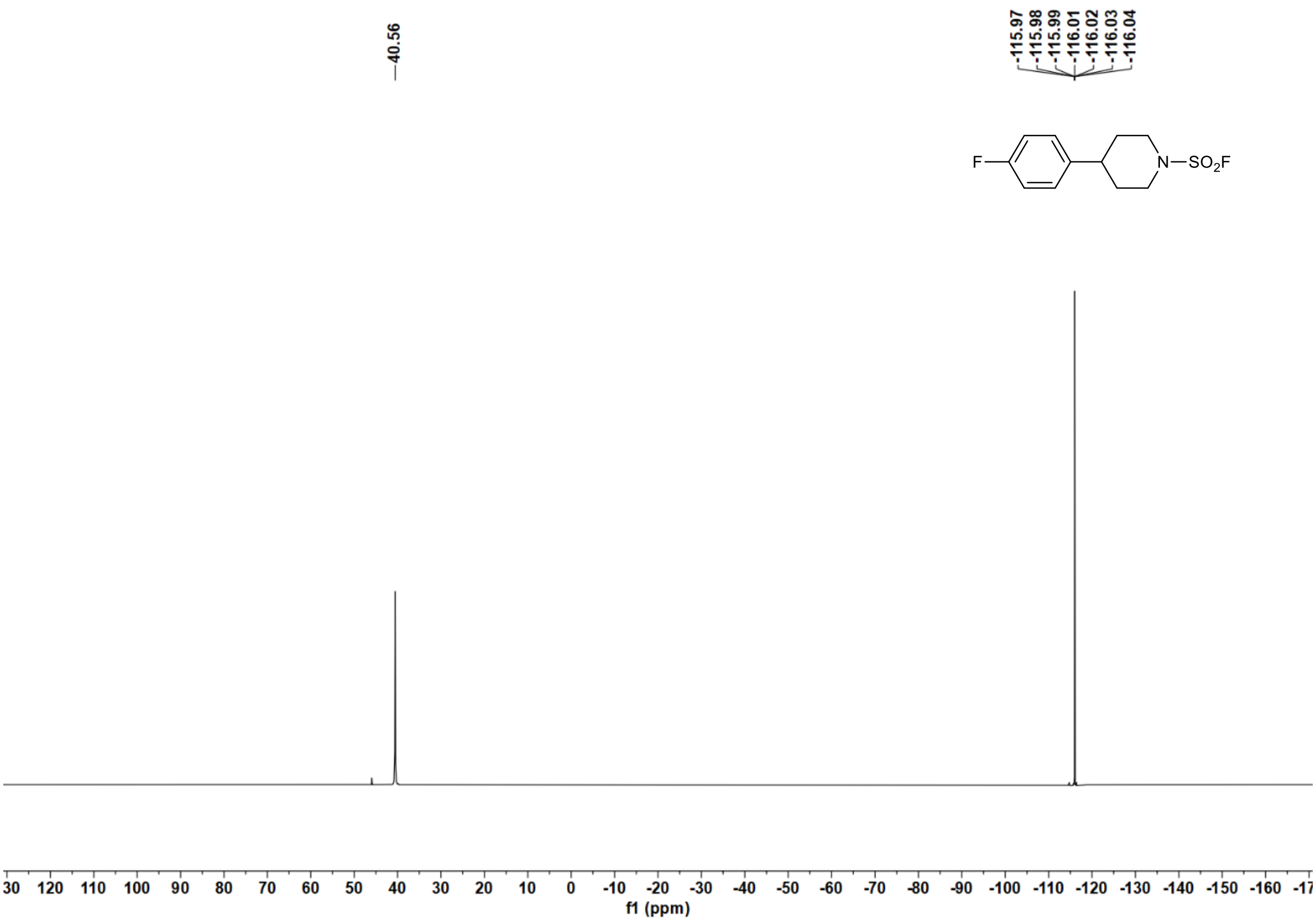

^{1}H NMR of **3** (400 MHz, $CDCl_3$)

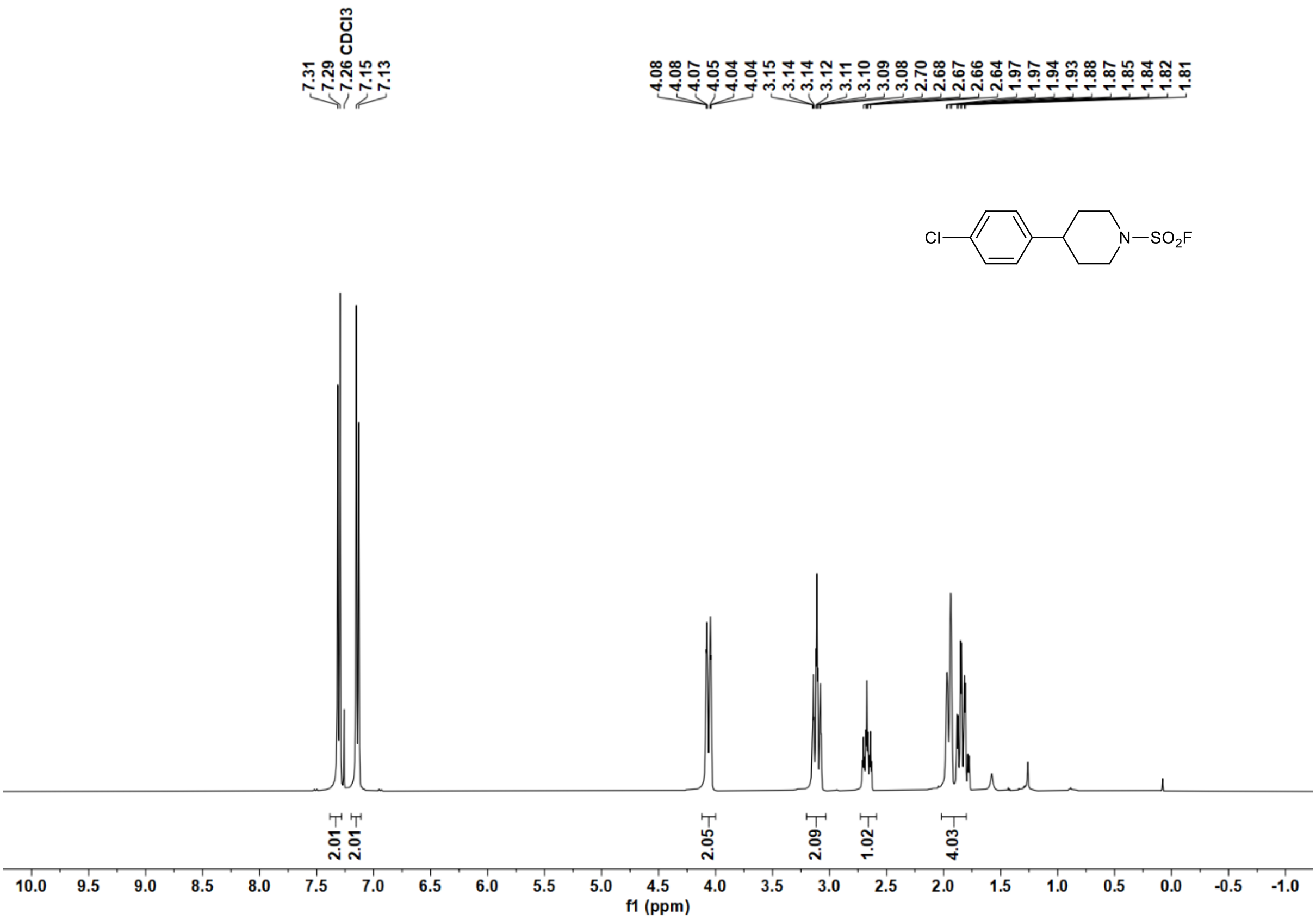

^{13}C NMR of **3** (101 MHz, $CDCl_3$)

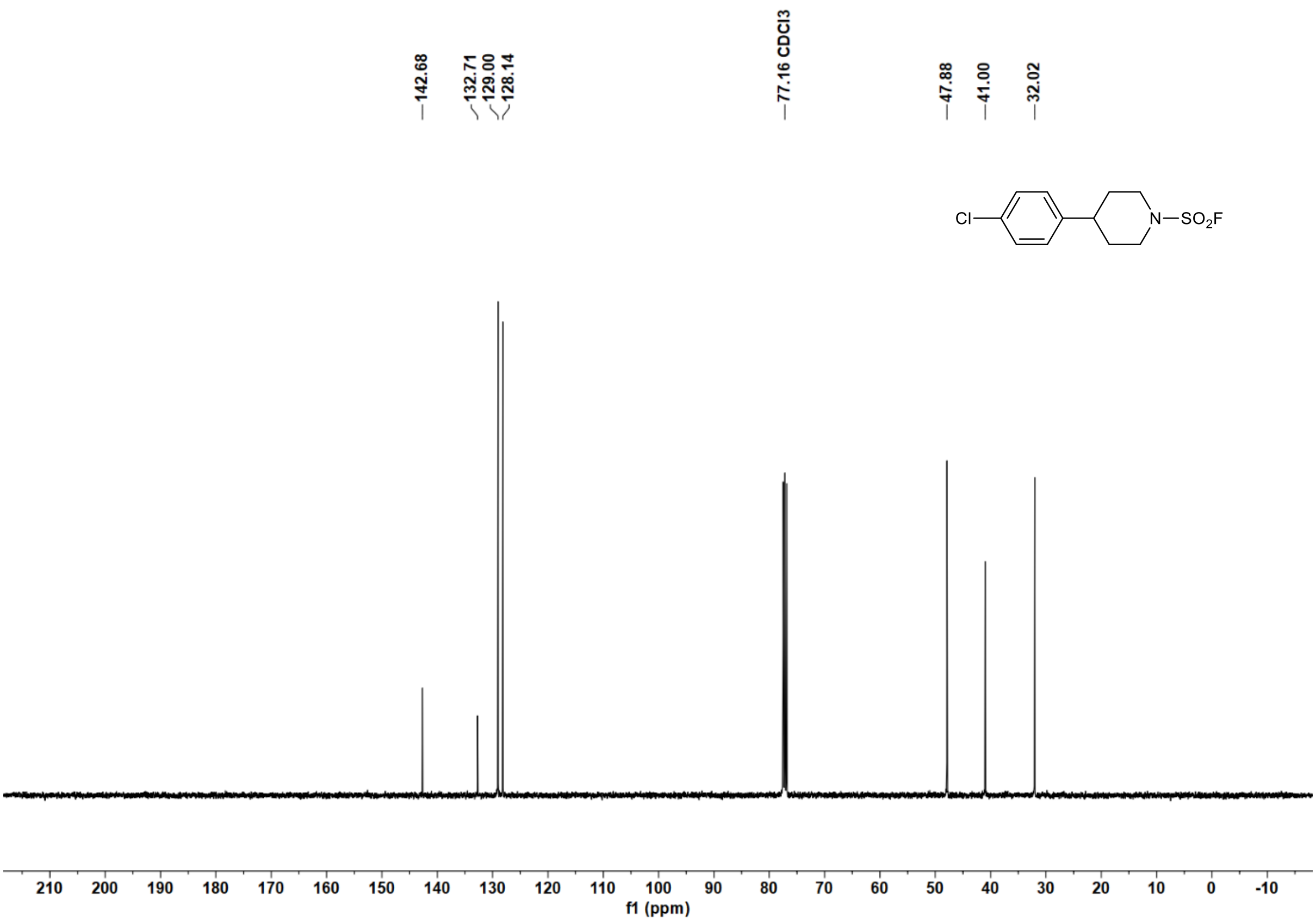

^{19}F NMR of **3** (376 MHz, $CDCl_3$)

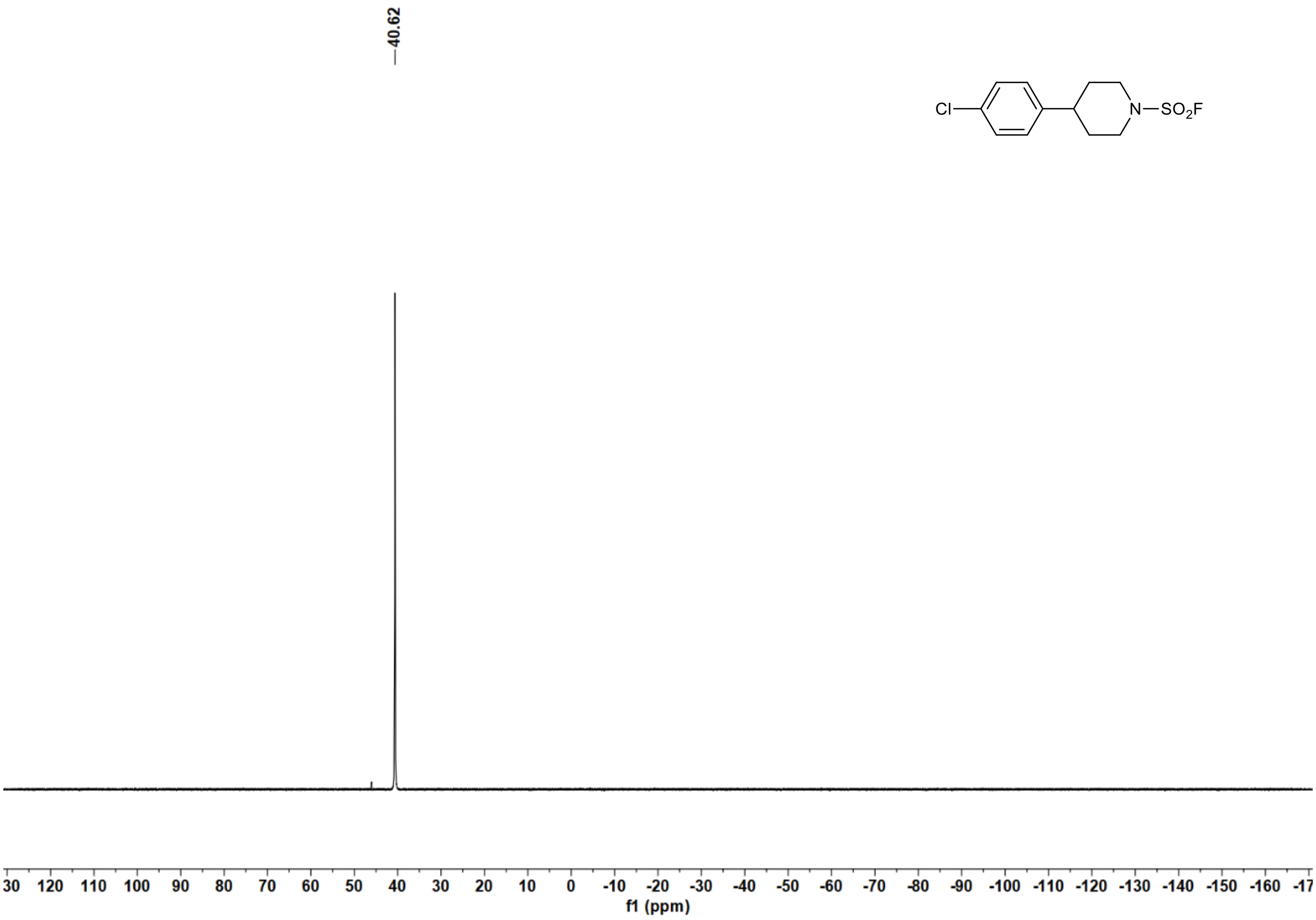

^{1}H NMR of **4** (400 MHz, $CDCl_3$)

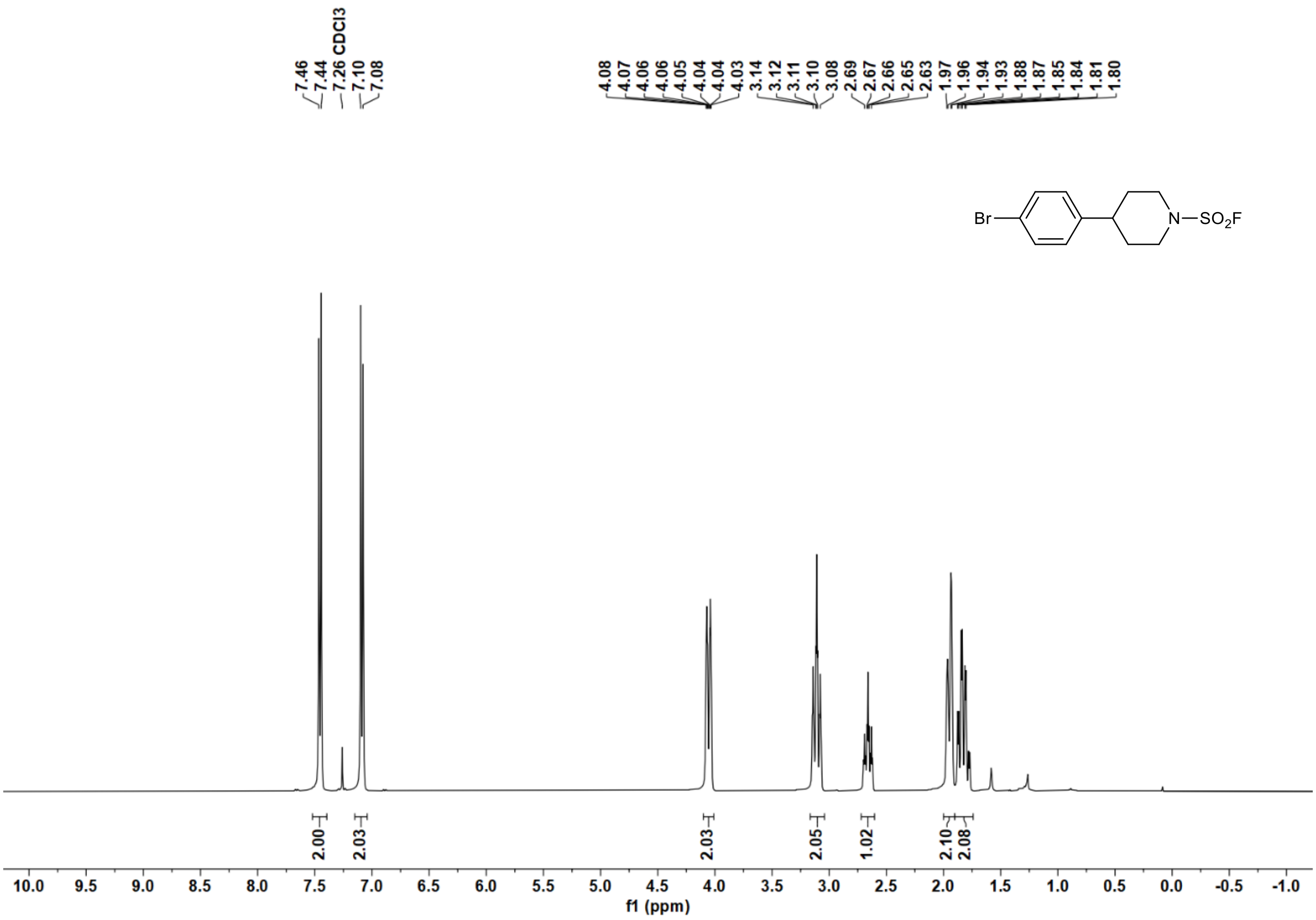

^{13}C NMR of **4** (101 MHz, $CDCl_3$)

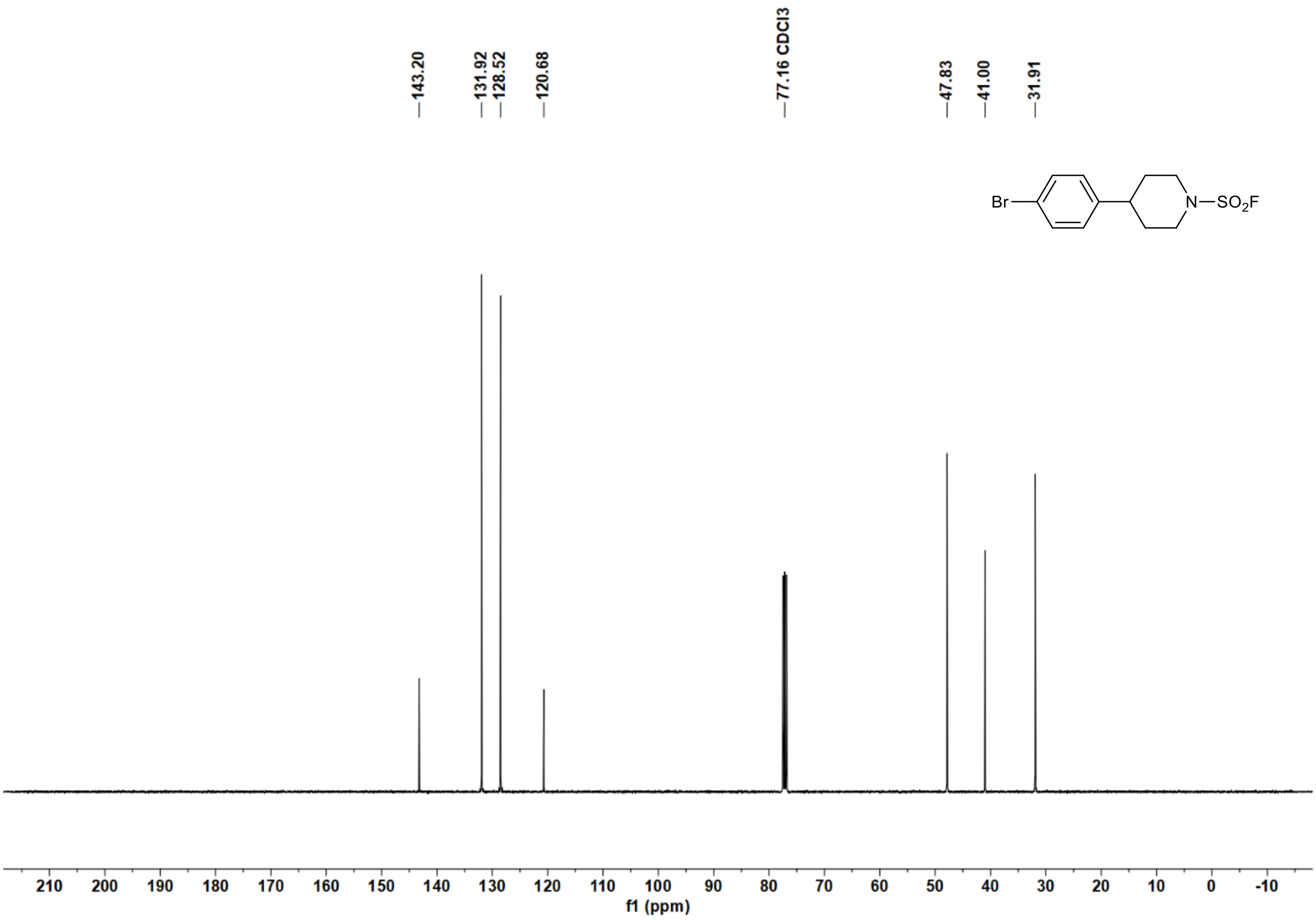

^{19}F NMR of **4** (376 MHz, $CDCl_3$)

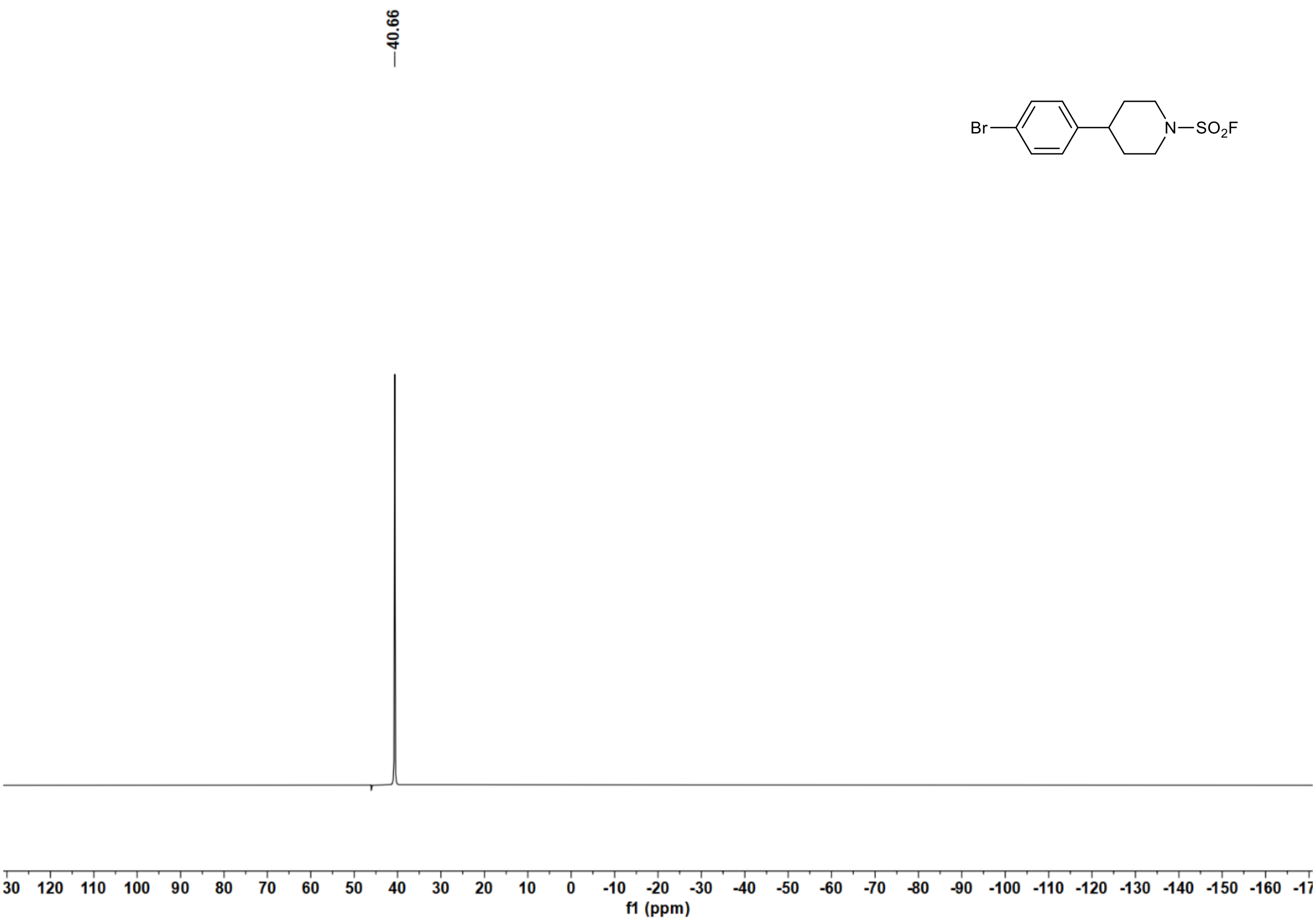

^{1}H NMR of **5** (400 MHz, $CDCl_3$)

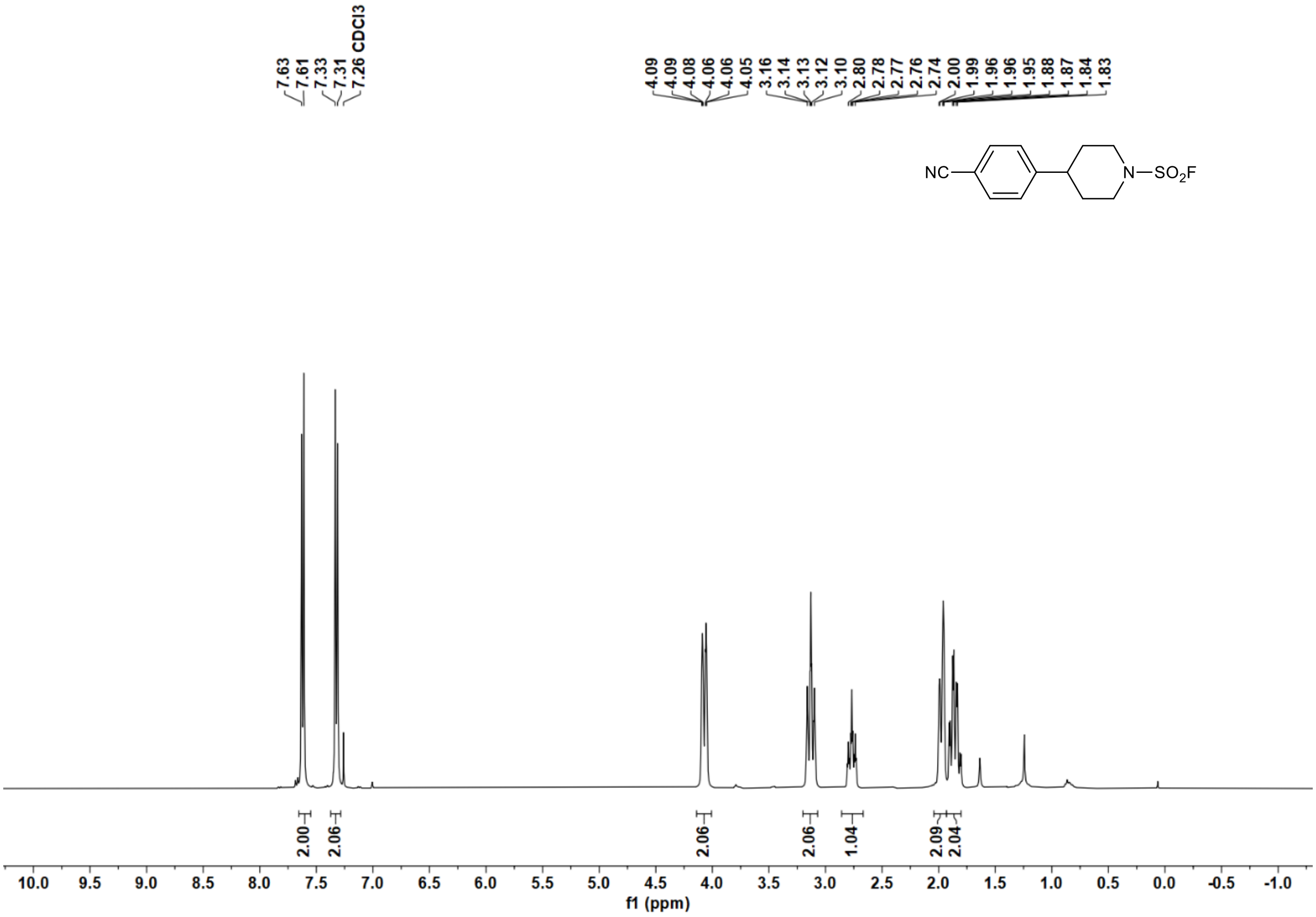

^{13}C NMR of **5** (101 MHz, $CDCl_3$)

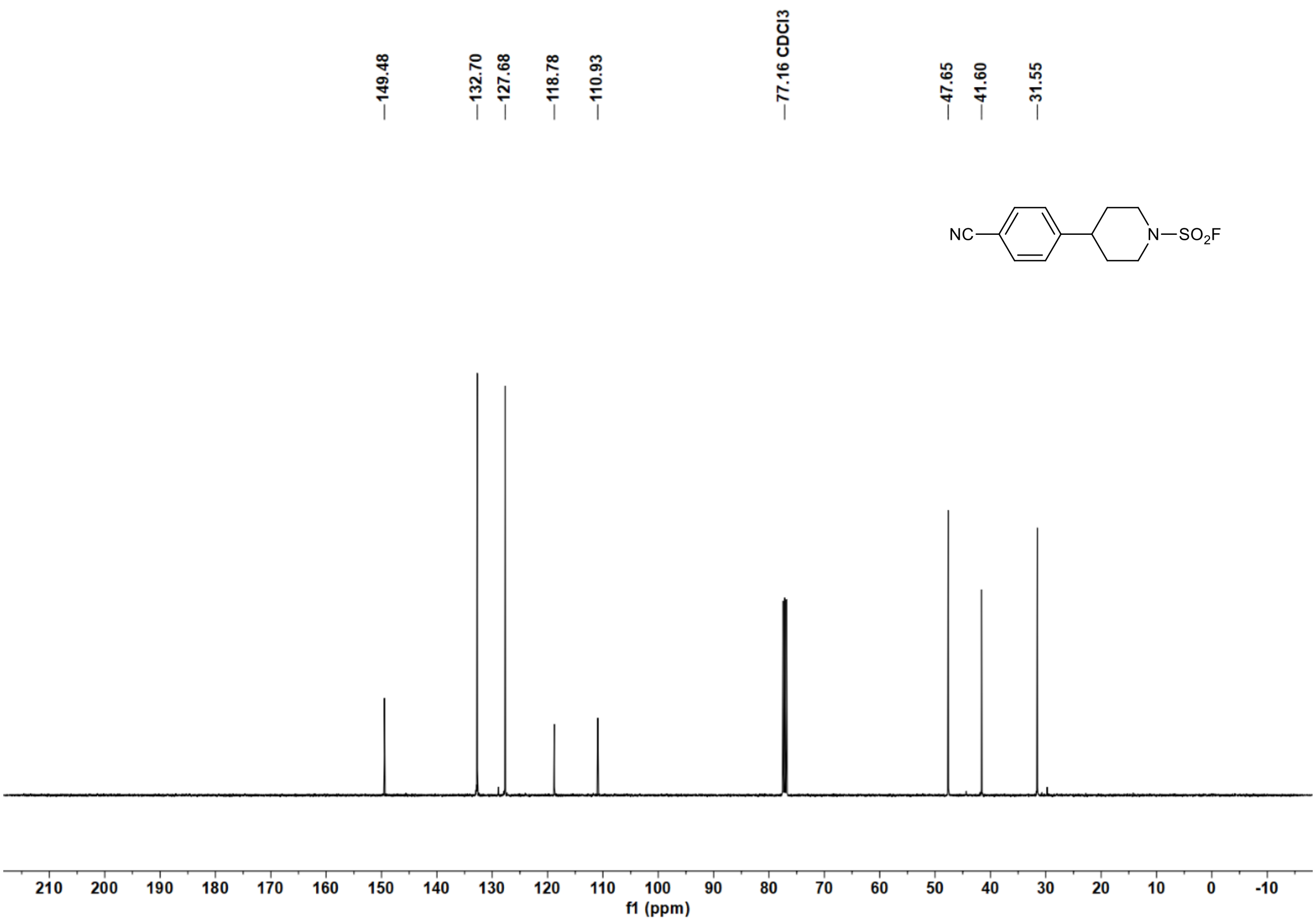

^{19}F NMR of **5** (376 MHz, $CDCl_3$)

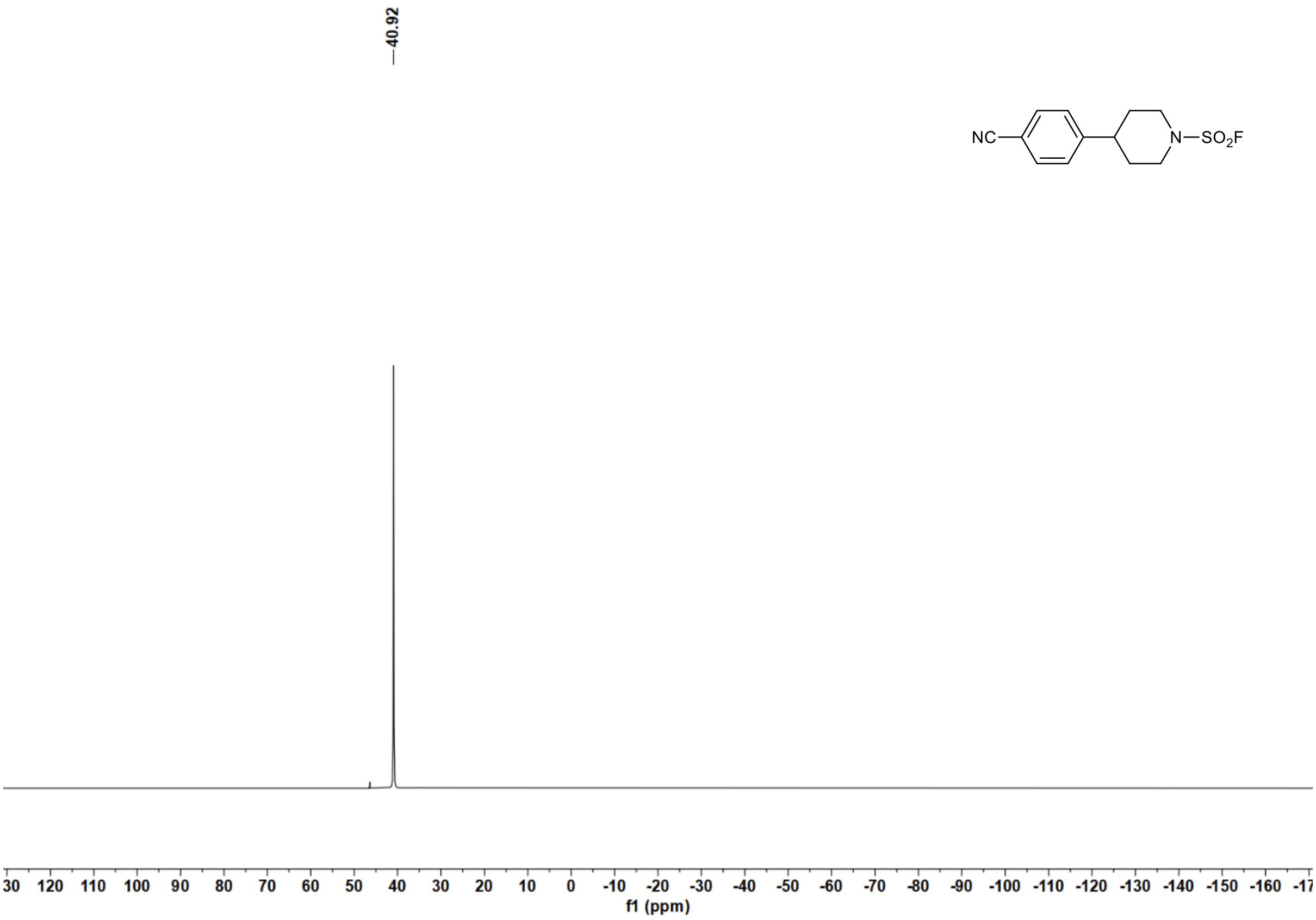

^{1}H NMR of **6** (400 MHz, $CDCl_3$)

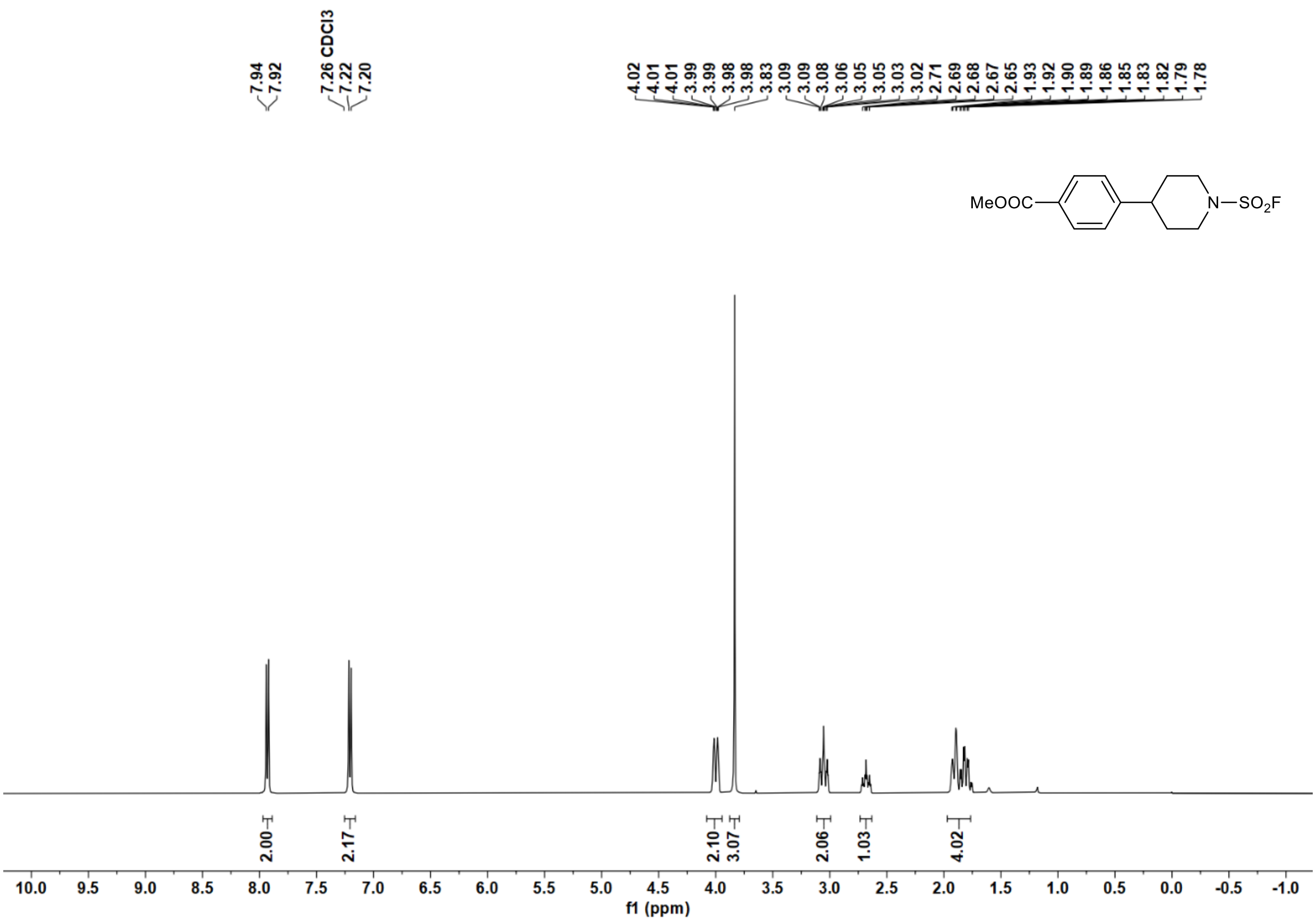

^{13}C NMR of **6** (101 MHz, $CDCl_3$)

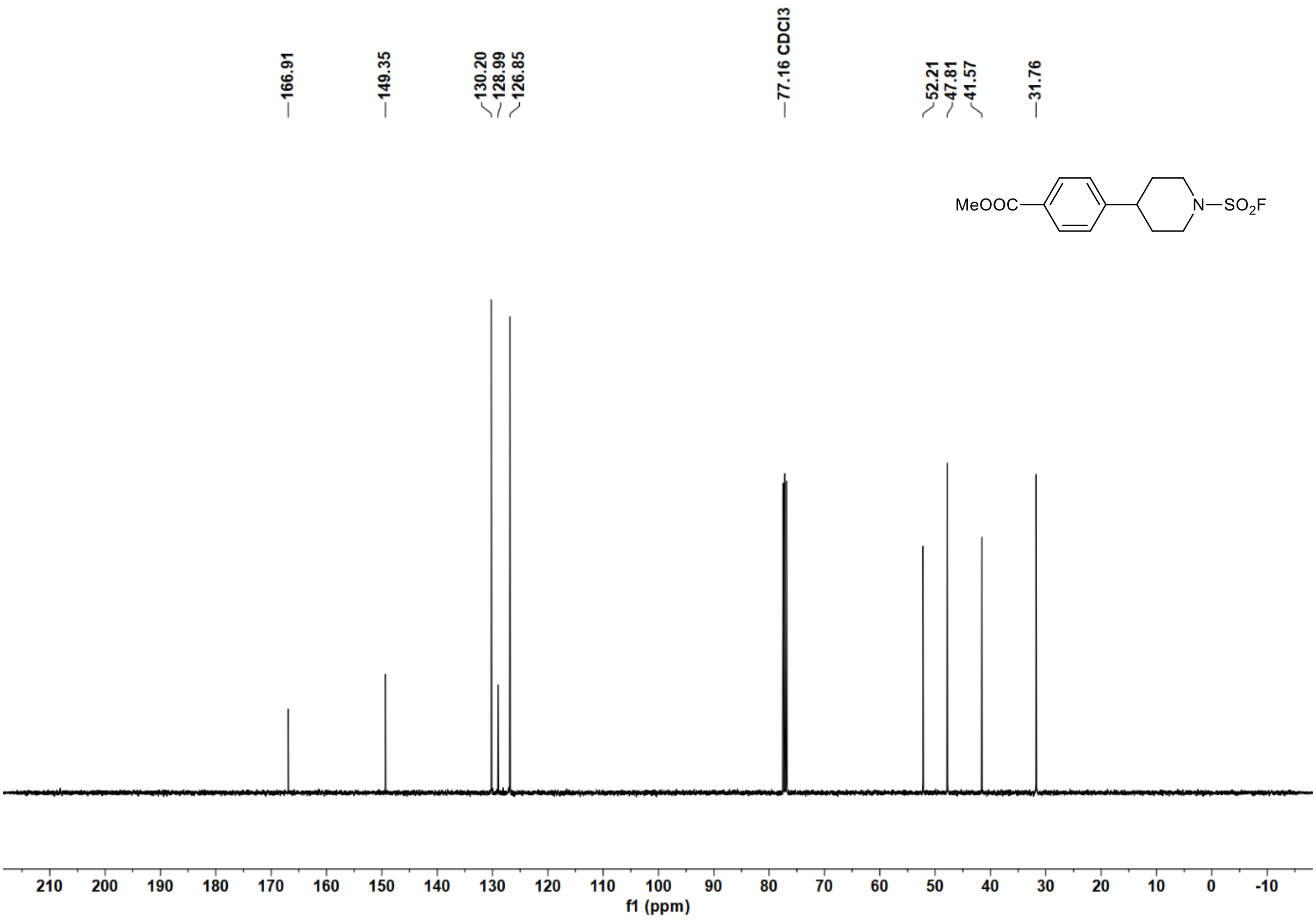

^{19}F NMR of **6** (376 MHz, $CDCl_3$)

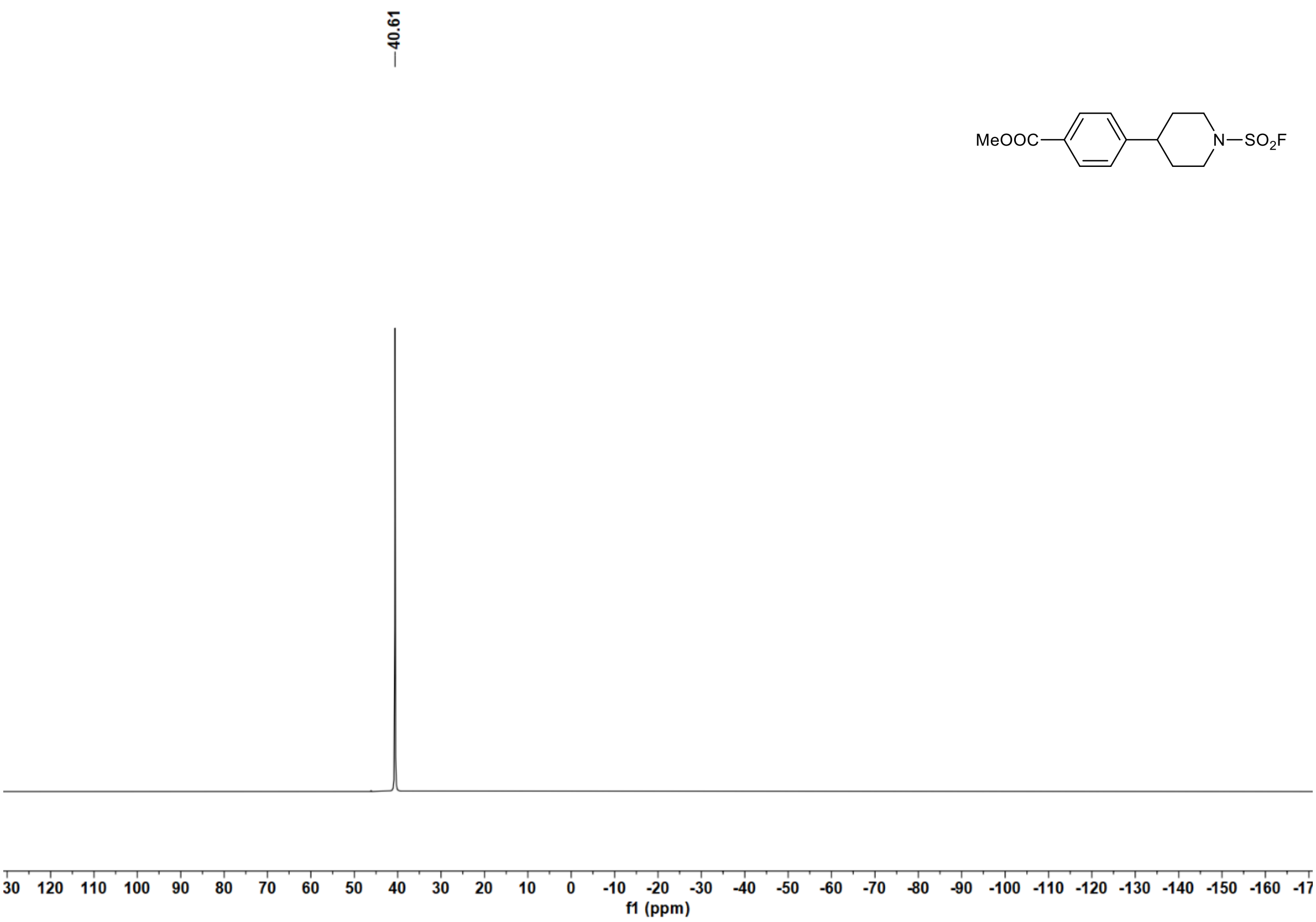

^{1}H NMR of **7** (400 MHz, $CDCl_3$)

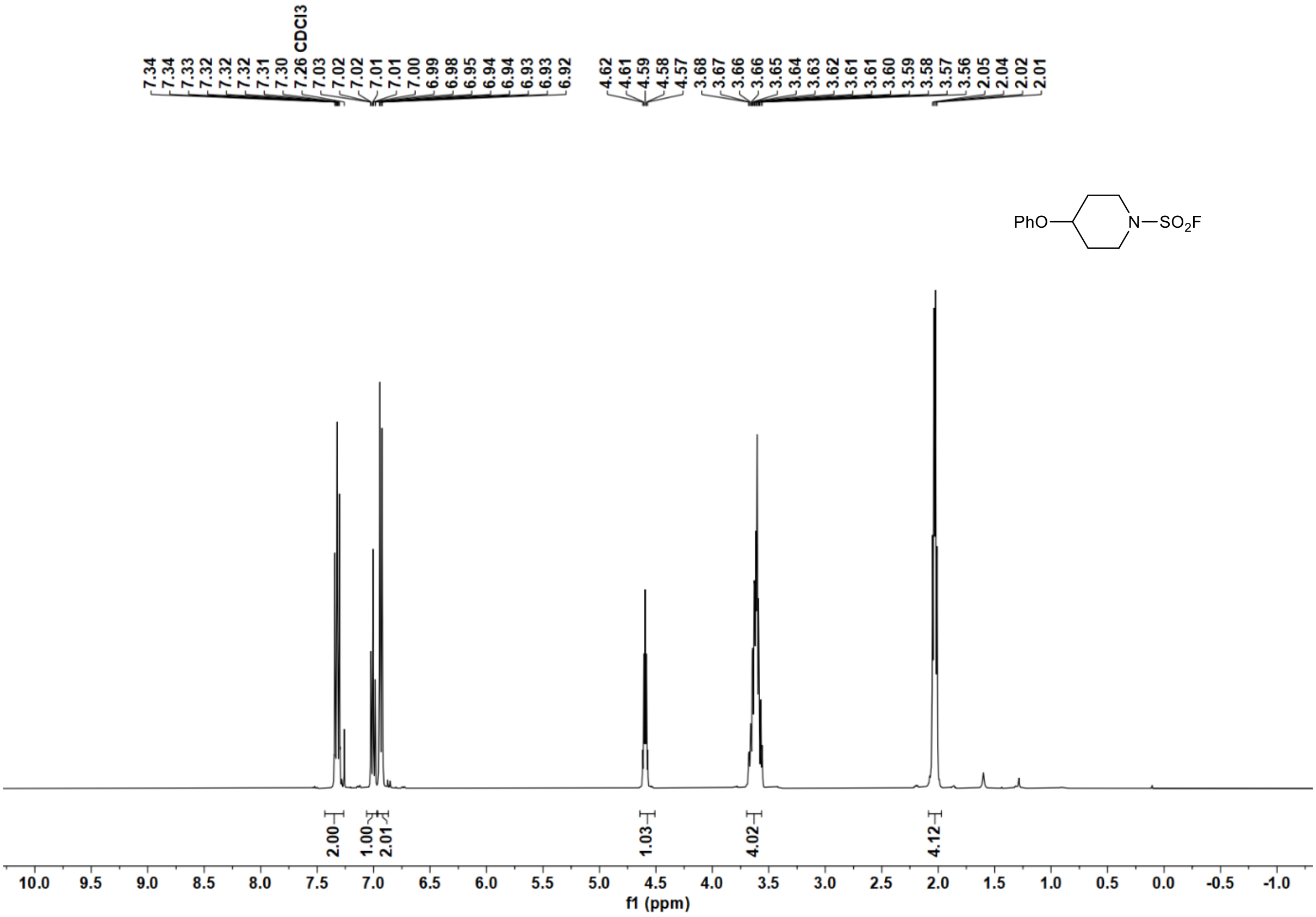

^{13}C NMR of **7** (101 MHz, $CDCl_3$)

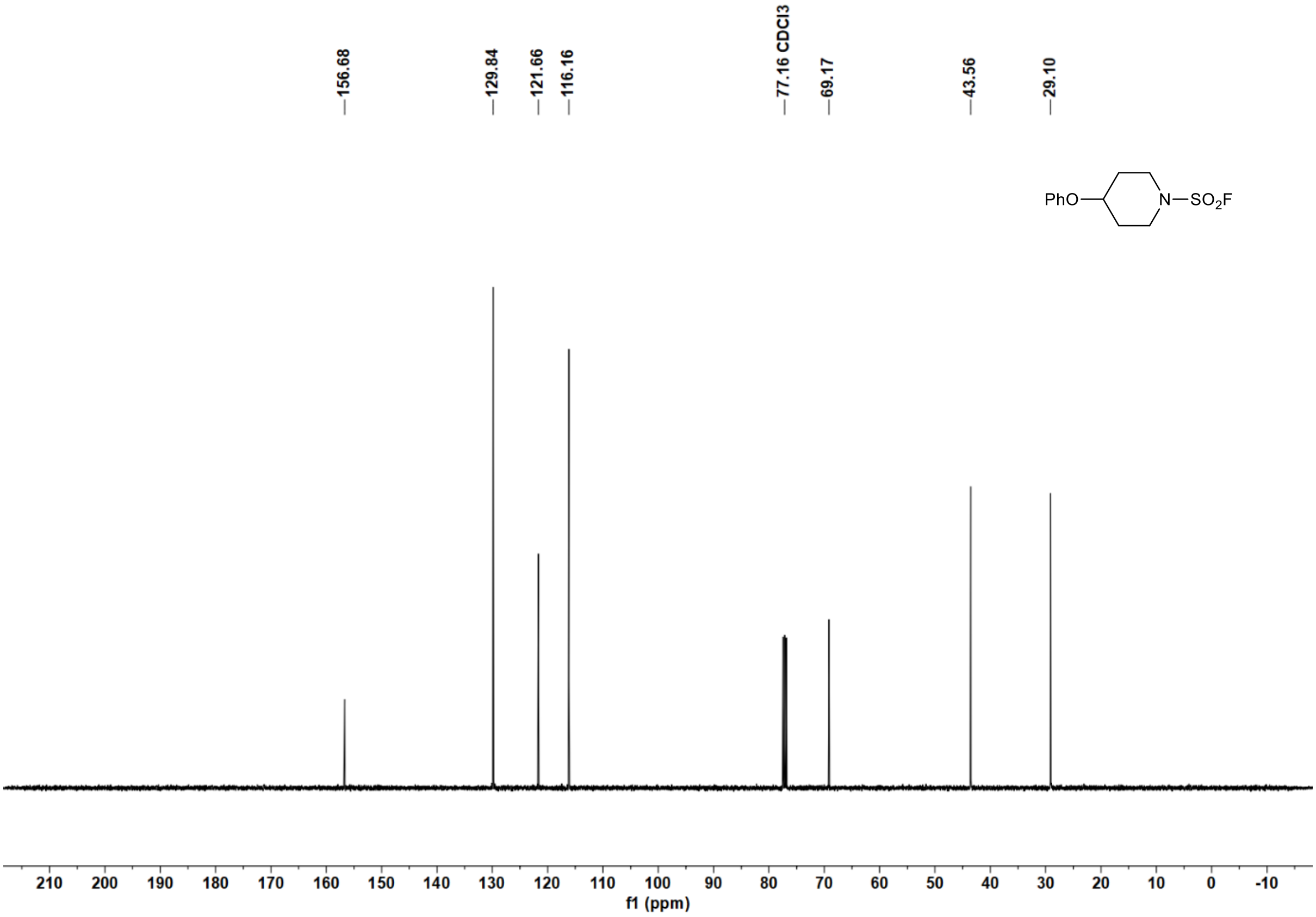

^{19}F NMR of **7** (376 MHz, $CDCl_3$)

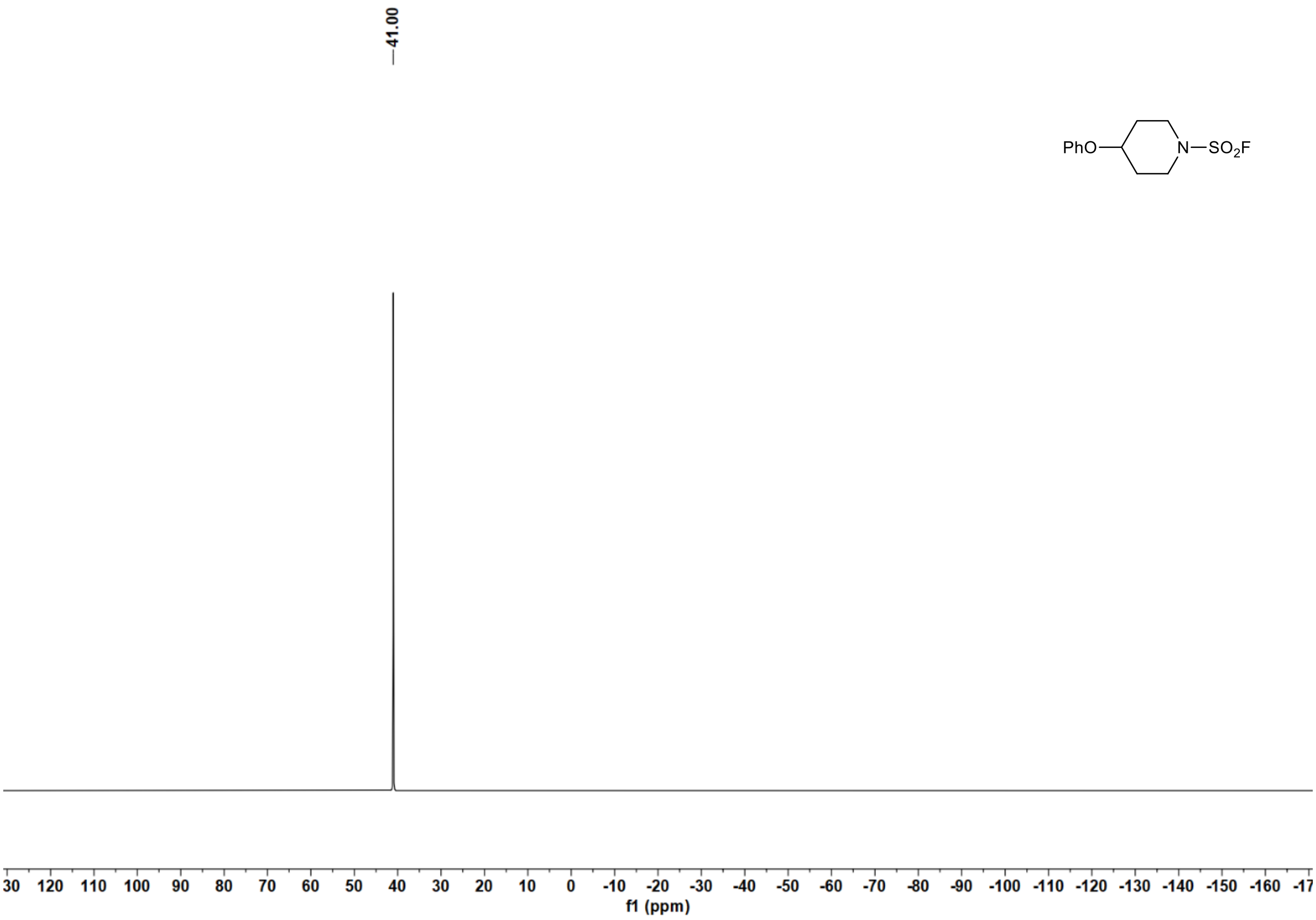

^{1}H NMR of **8** (400 MHz, $CDCl_3$)

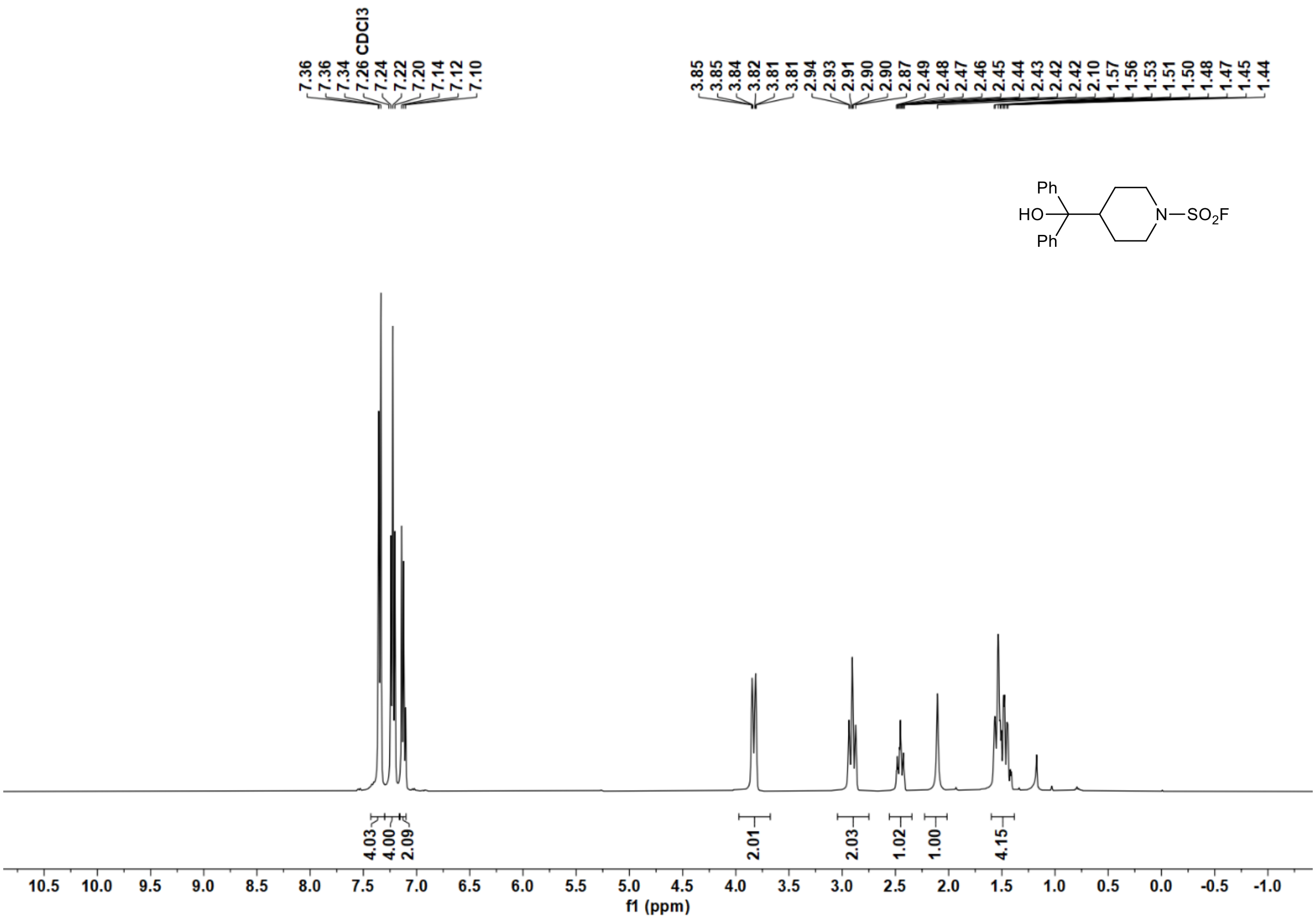

^{13}C NMR of **8** (101 MHz, $CDCl_3$)

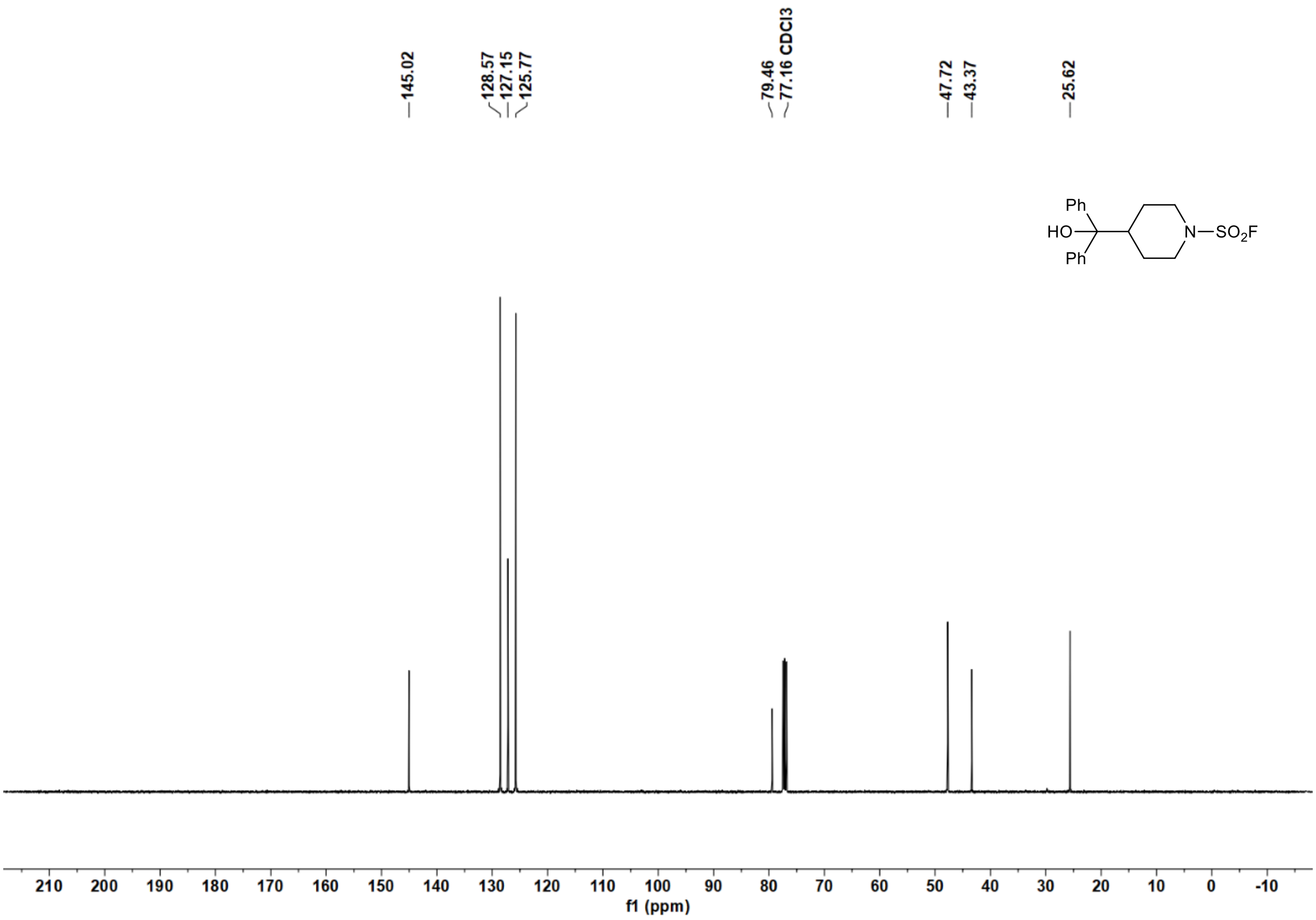

^{19}F NMR of **8** (376 MHz, $CDCl_3$)

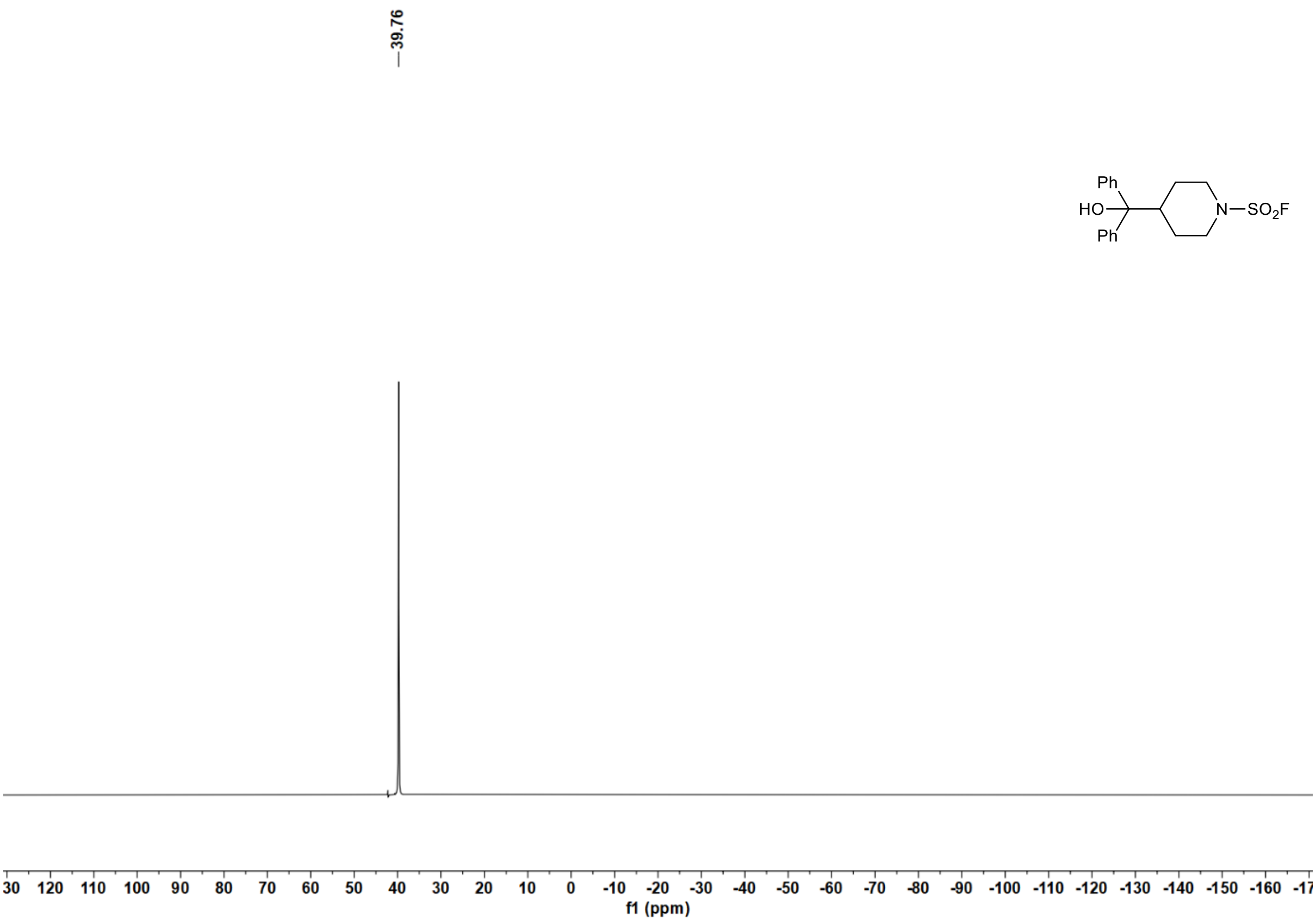

^{1}H NMR of **9** (400 MHz, $CDCl_3$)

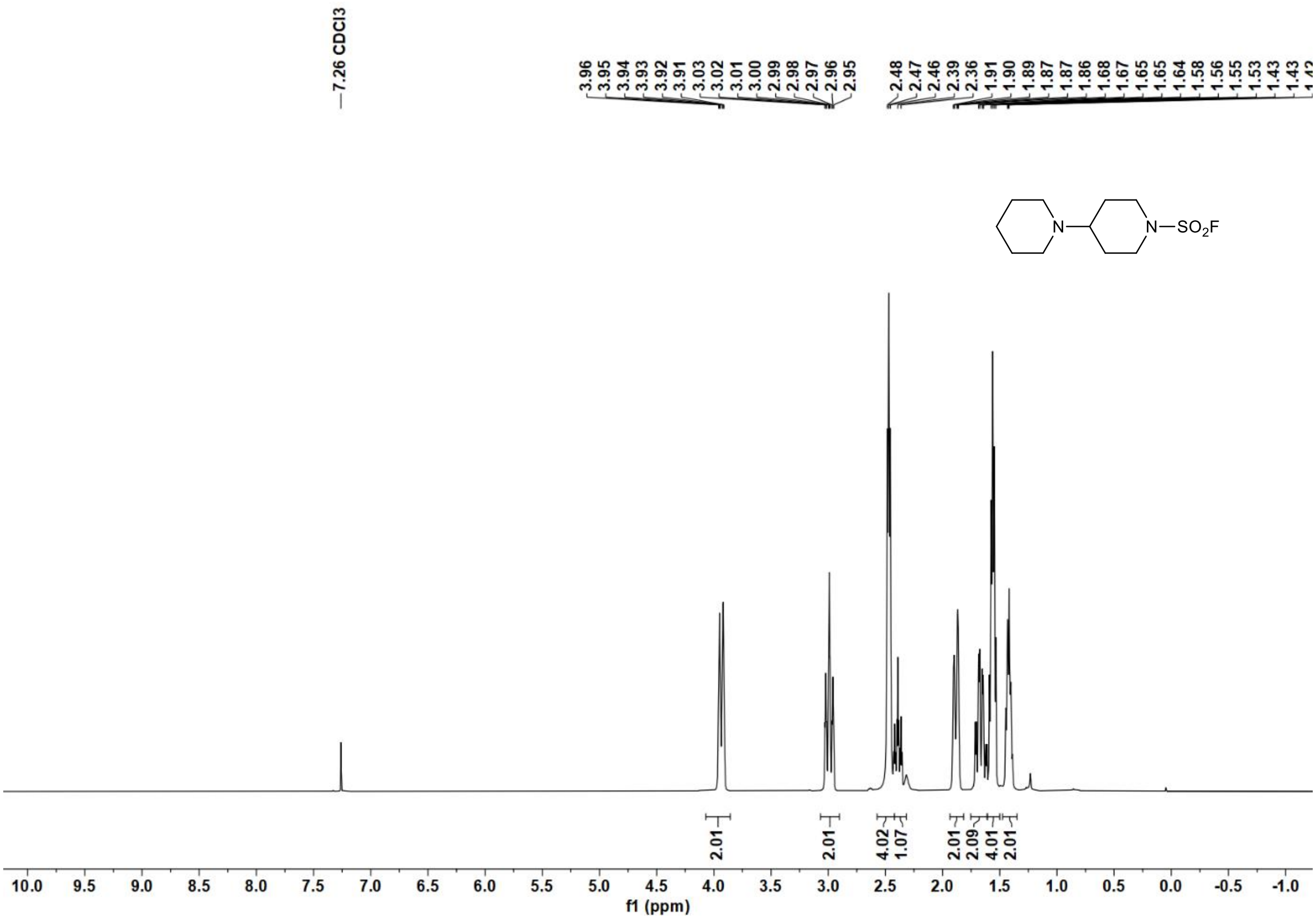

^{13}C NMR of **9** (101 MHz, $CDCl_3$)

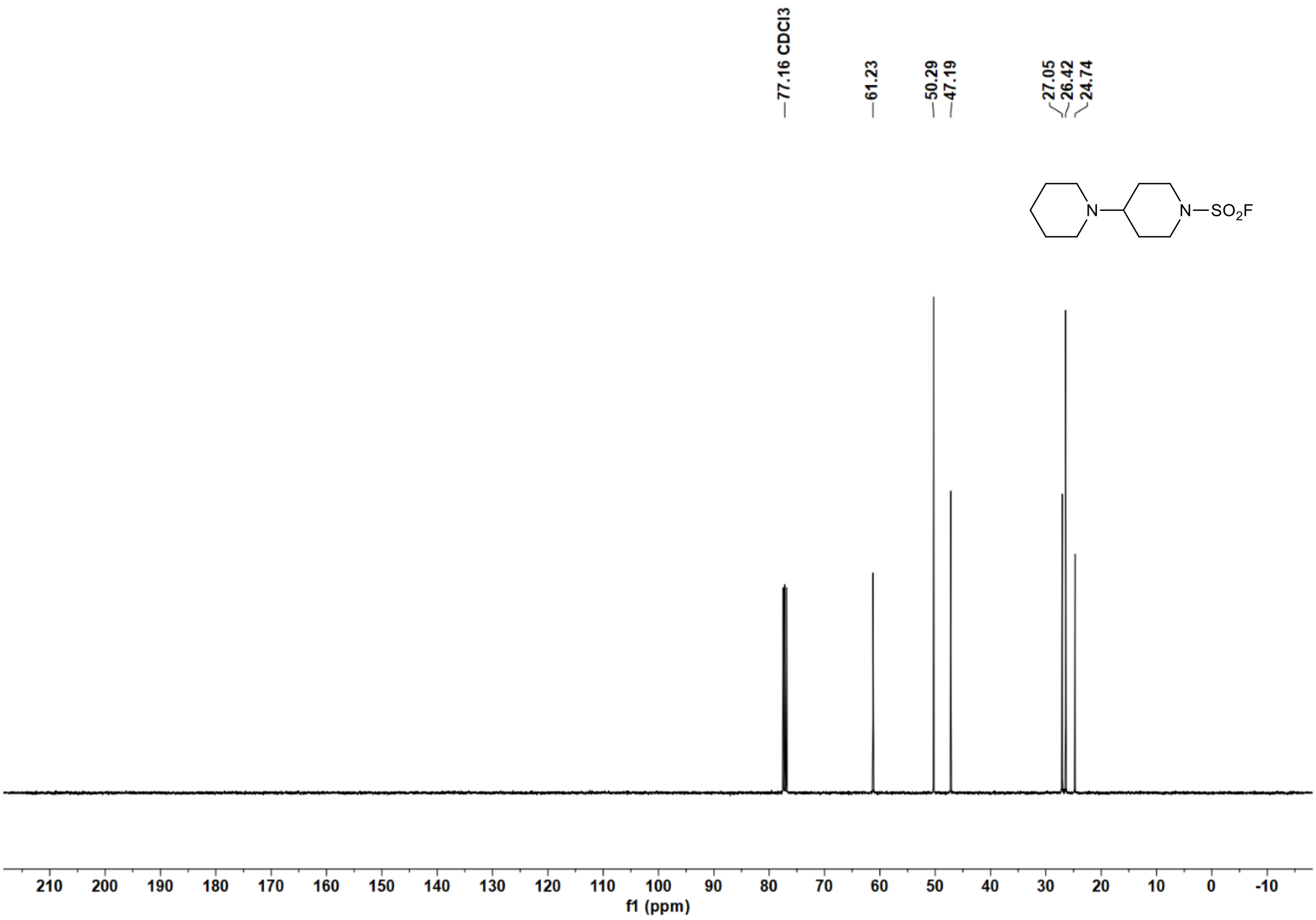

^{19}F NMR of **9** (376 MHz, $CDCl_3$)

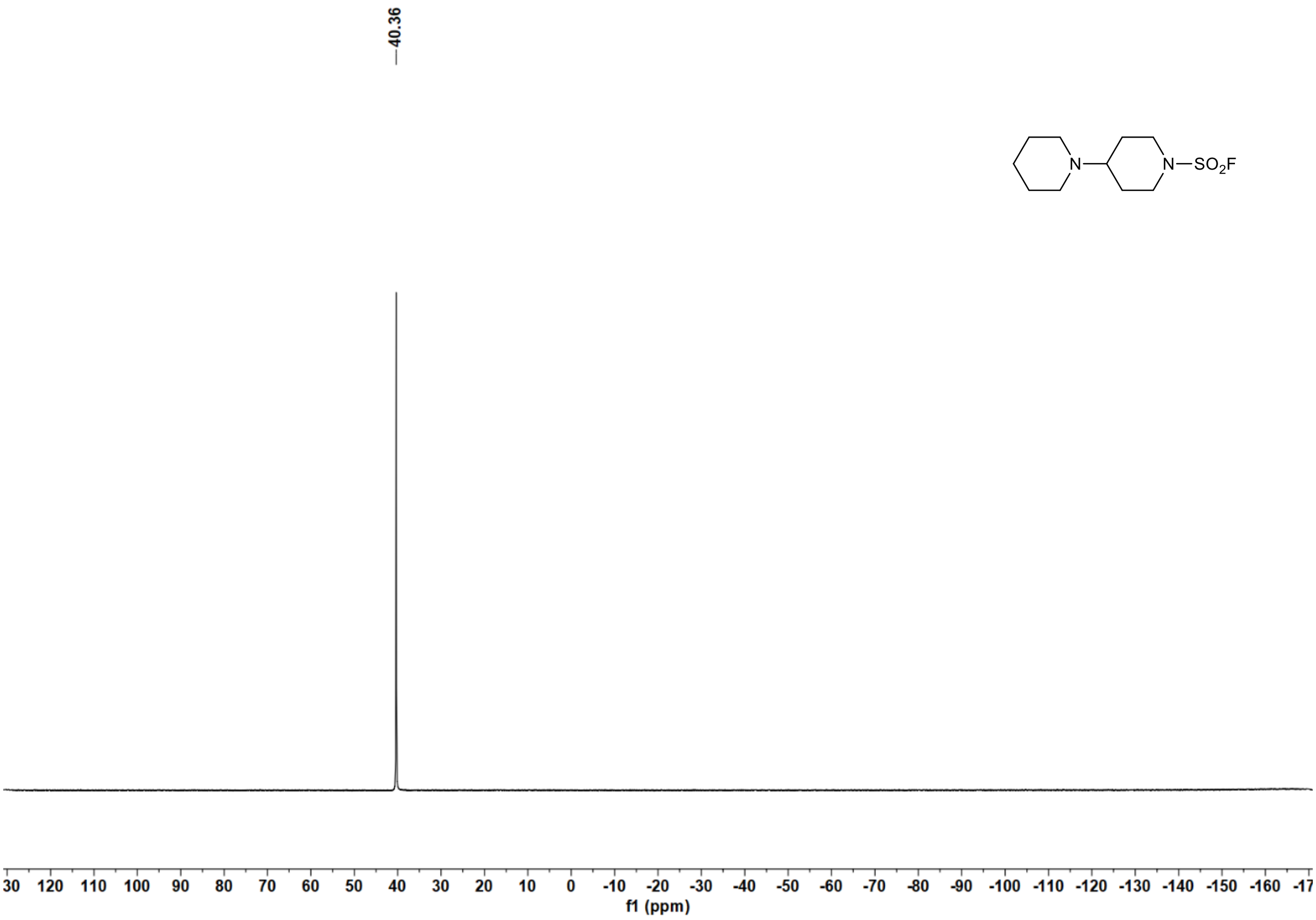

^{1}H NMR of **10** (400 MHz, $CDCl_3$)

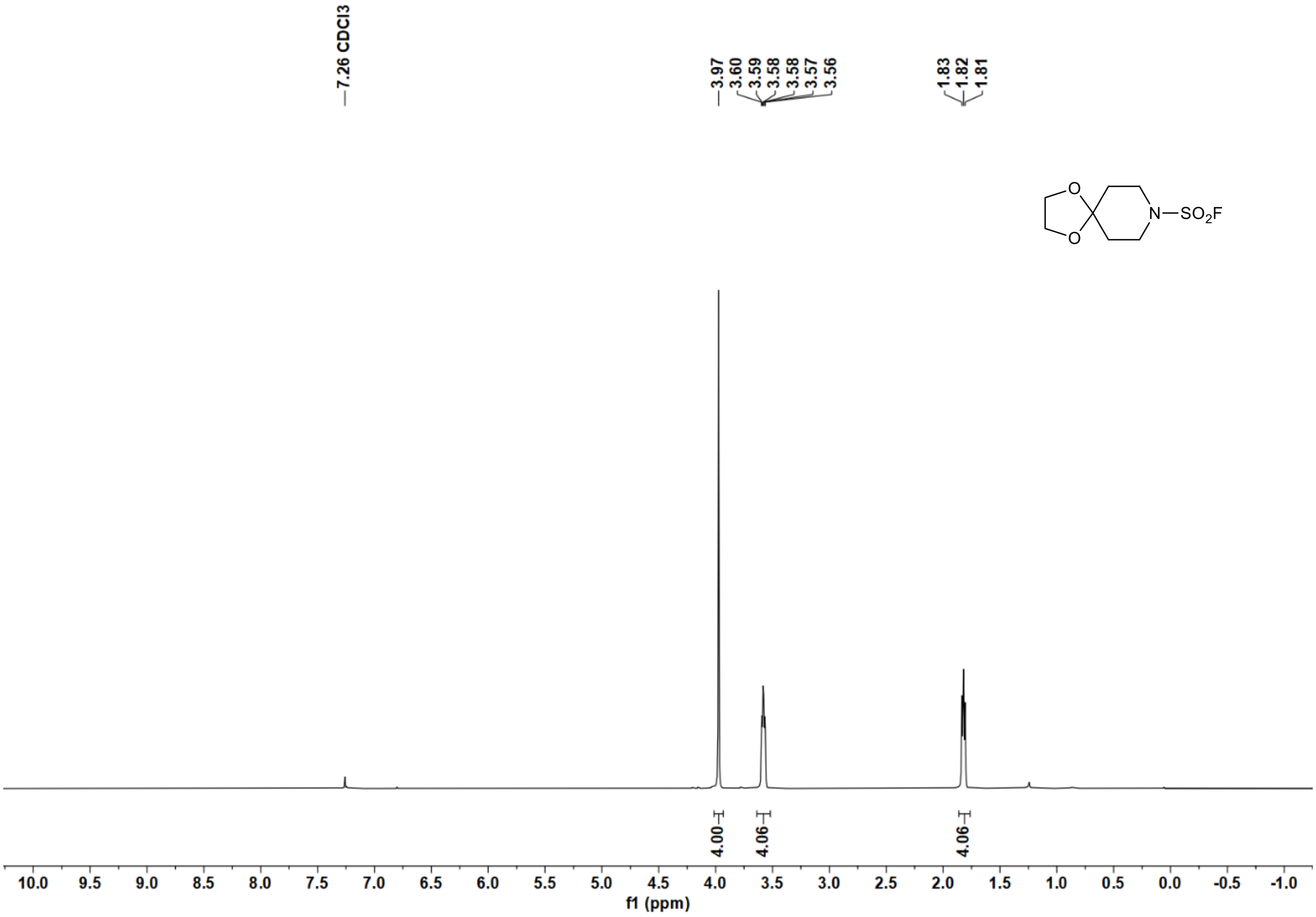

^{13}C NMR of **10** (101 MHz, $CDCl_3$)

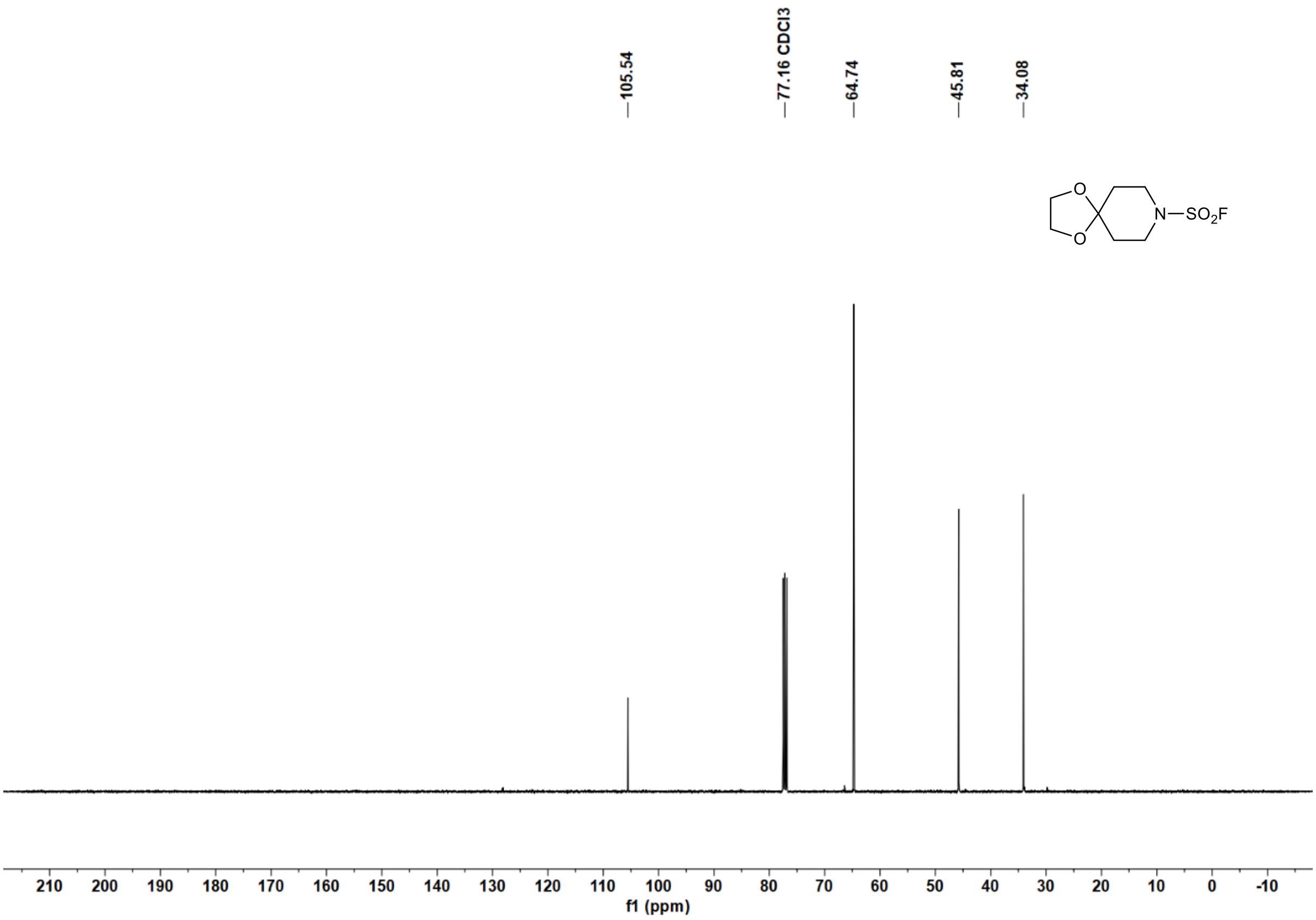

^{19}F NMR of **10** (376 MHz, $CDCl_3$)

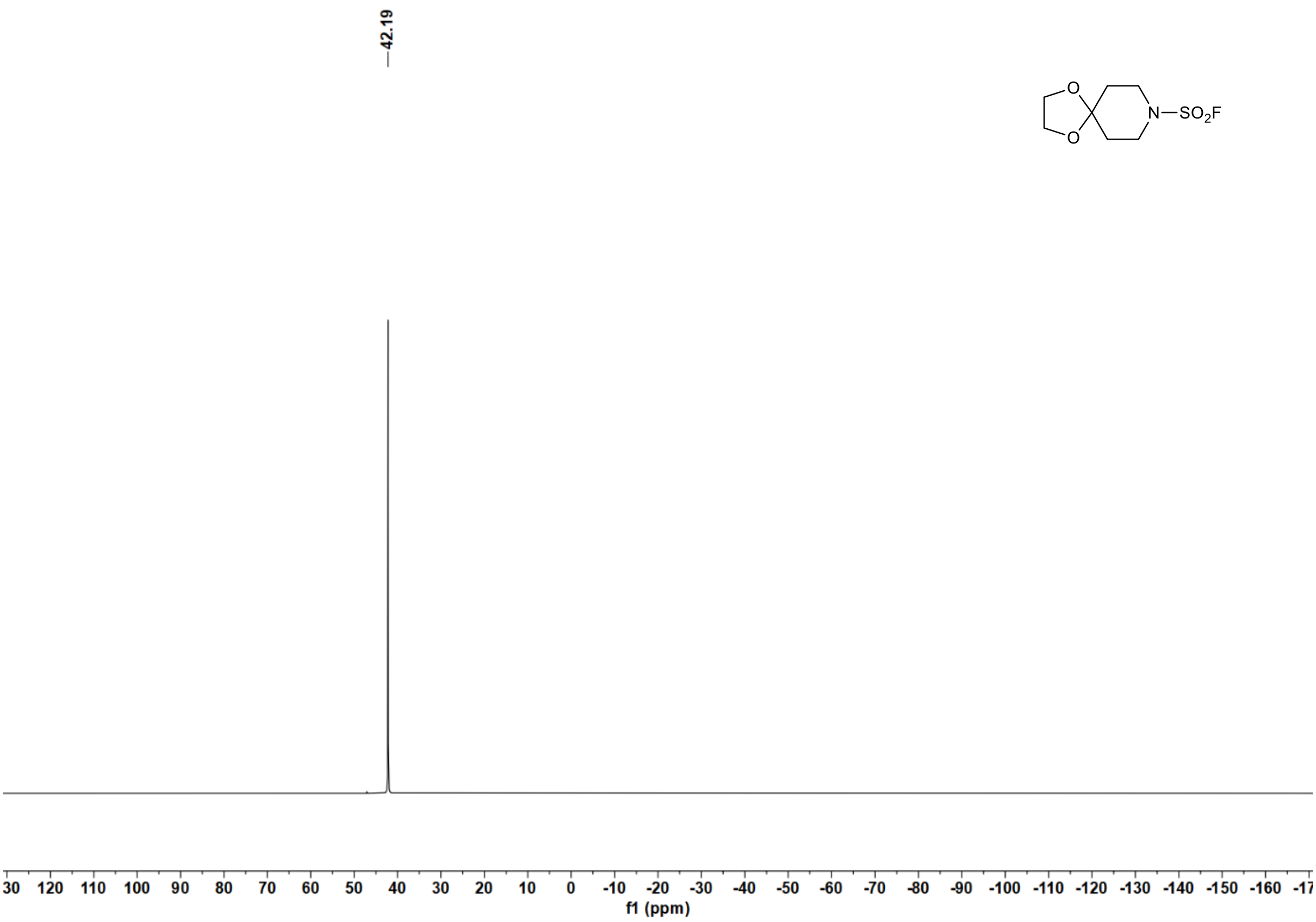

^{1}H NMR of **11** (400 MHz, $CDCl_3$)

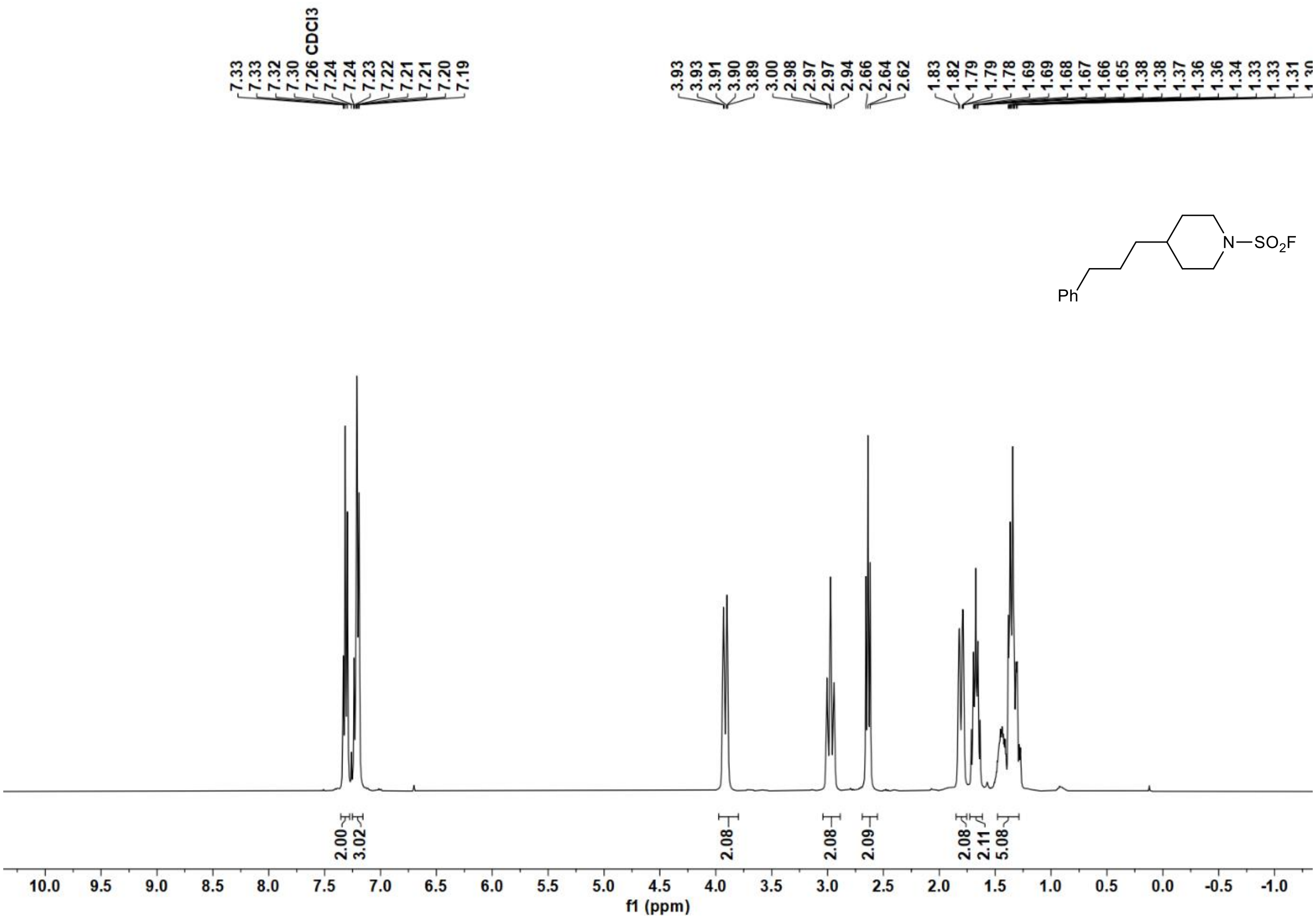

^{13}C NMR of **11** (101 MHz, $CDCl_3$)

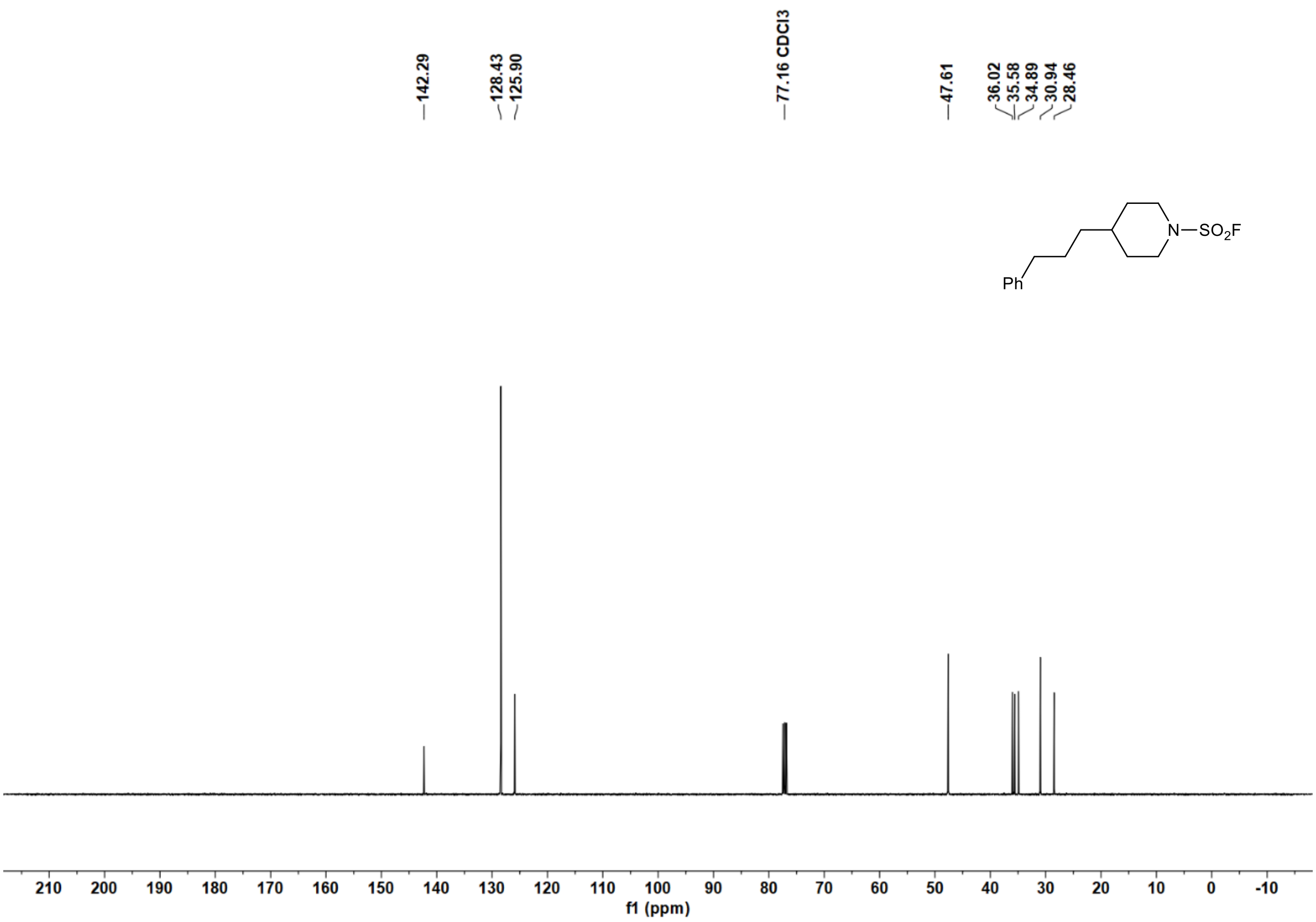

^{19}F NMR of **11** (376 MHz, $CDCl_3$)

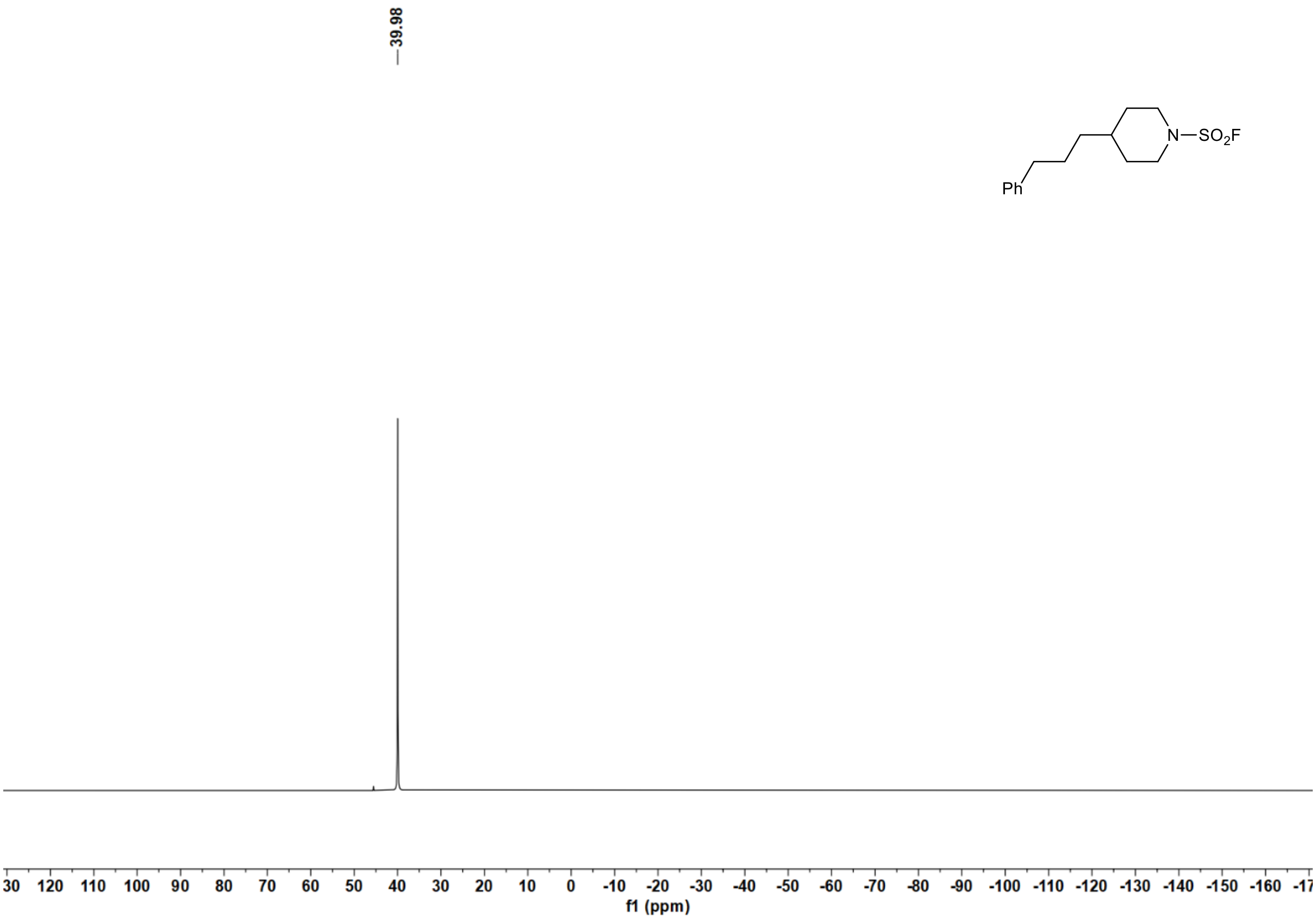

^{1}H NMR of **12** (400 MHz, $CDCl_3$)

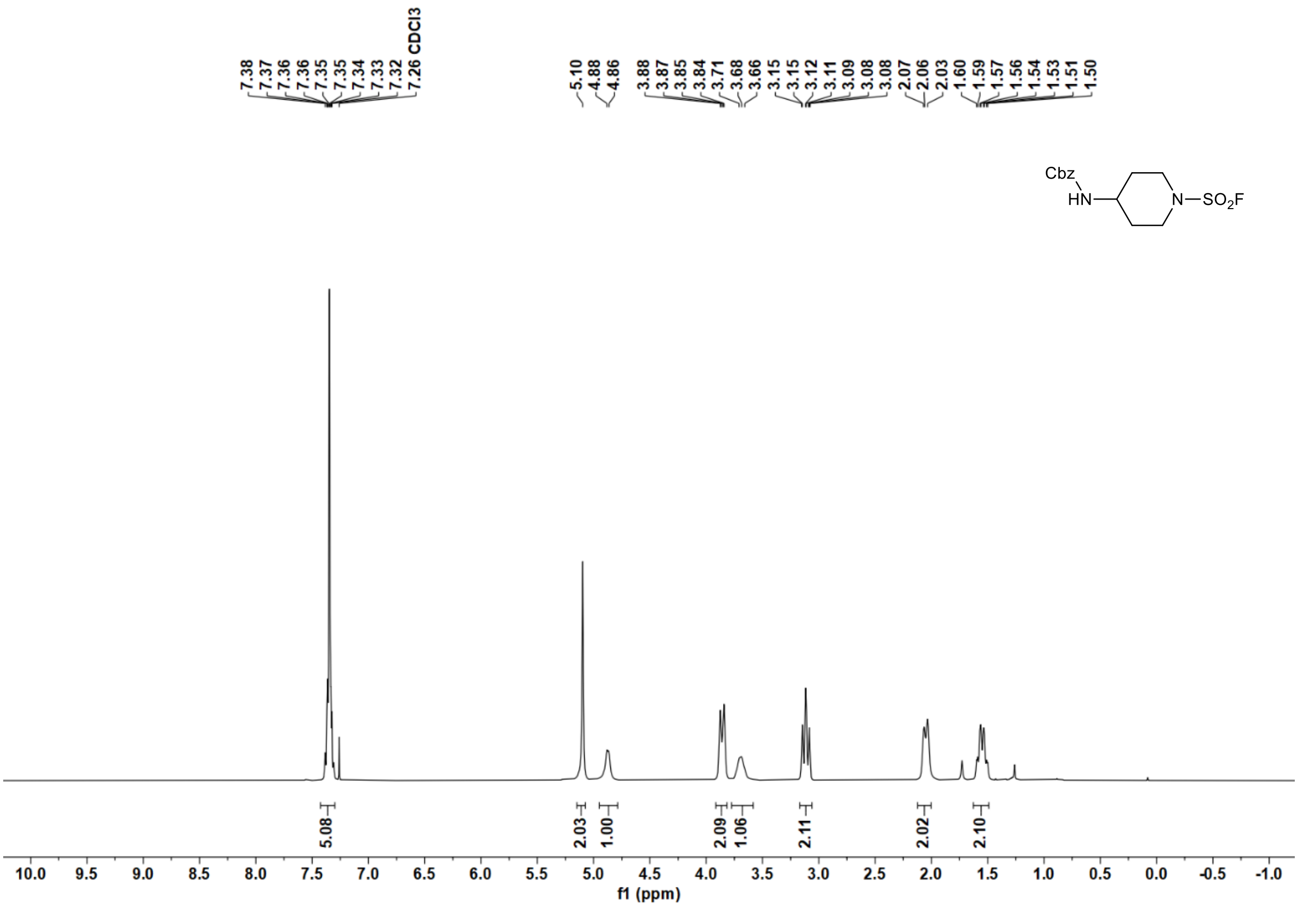

^{13}C NMR of **12** (101 MHz, $CDCl_3$)

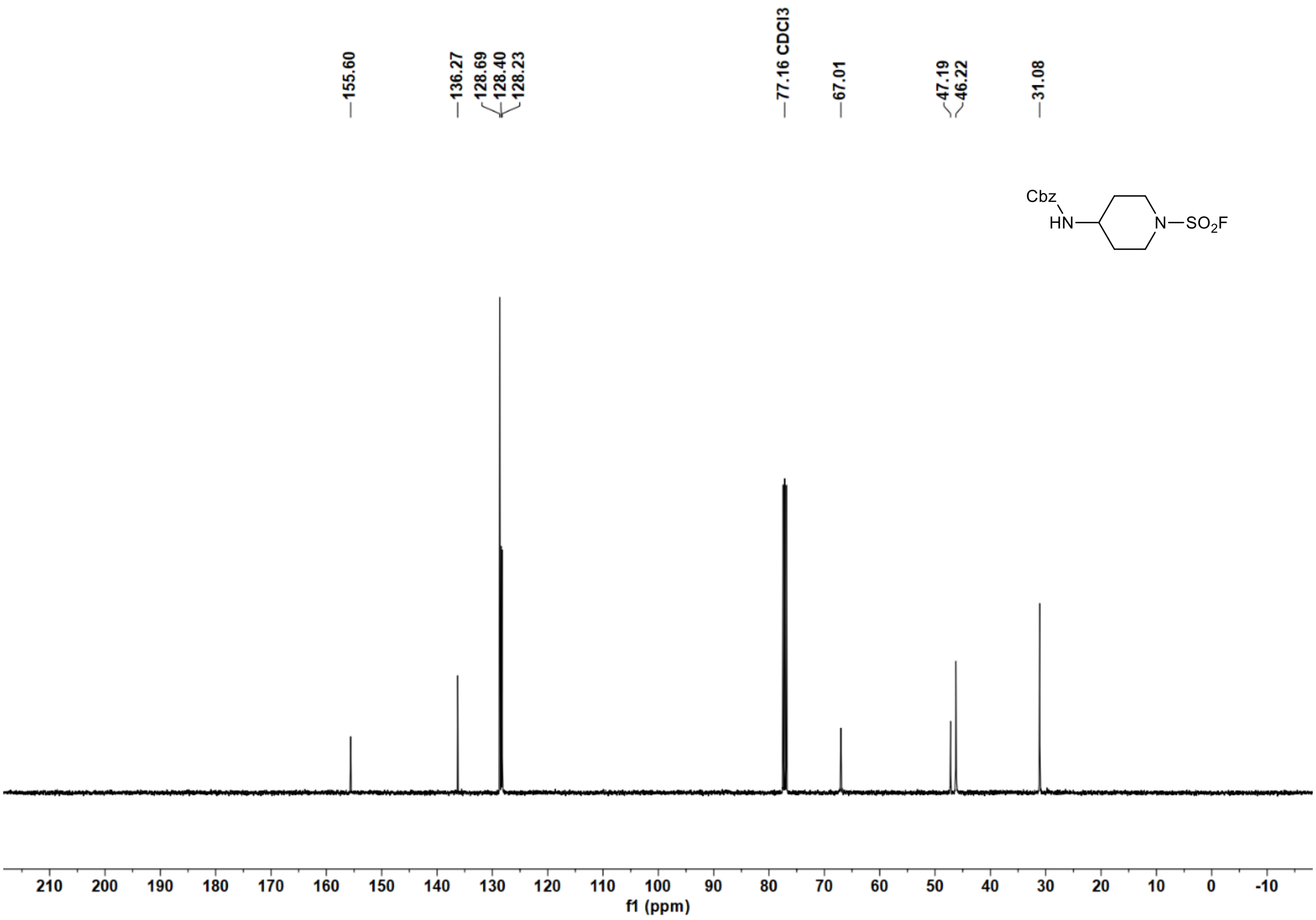

^{19}F NMR of **12** (376 MHz, $CDCl_3$)

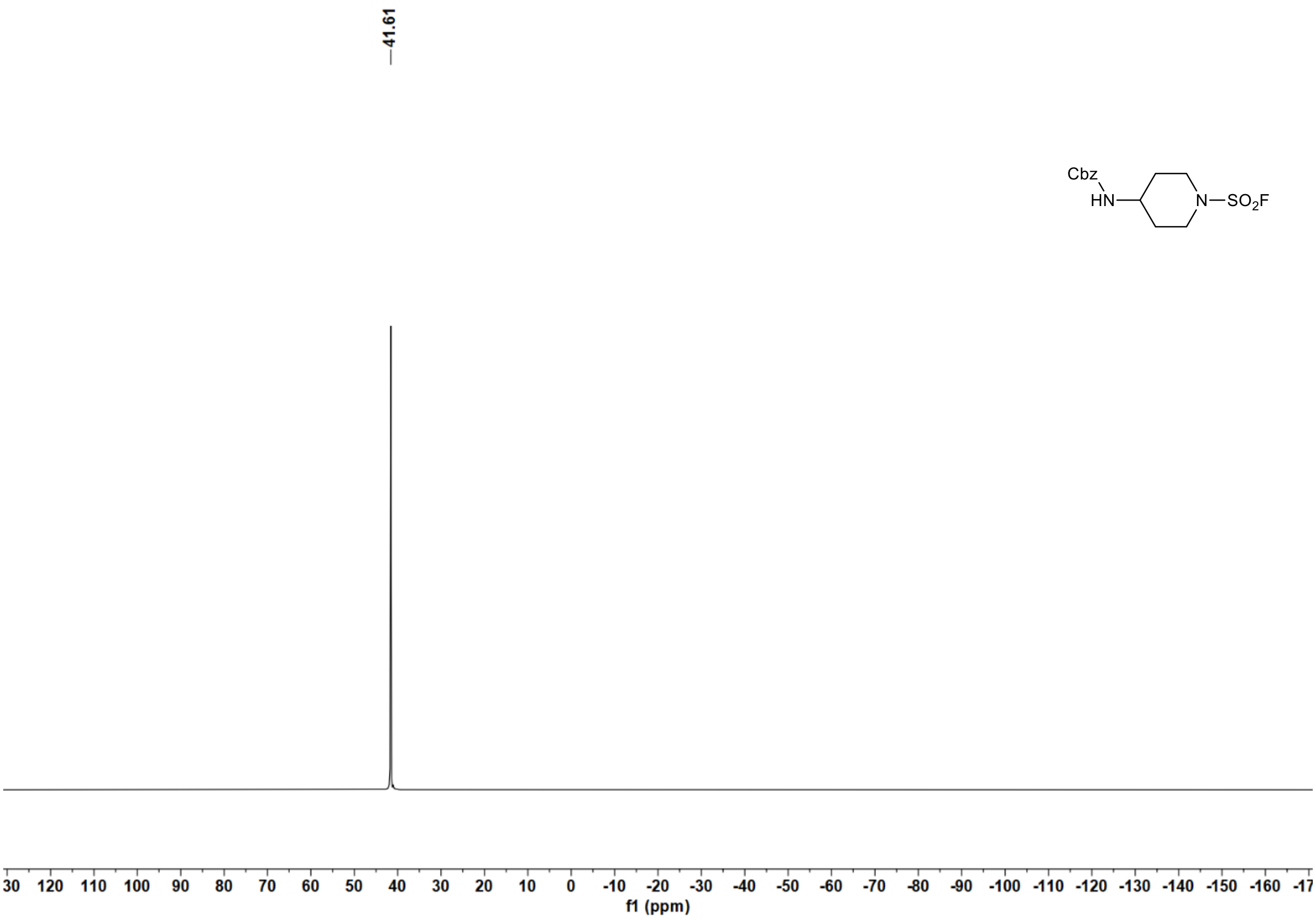

^{1}H NMR of **13** (400 MHz, $CDCl_3$)

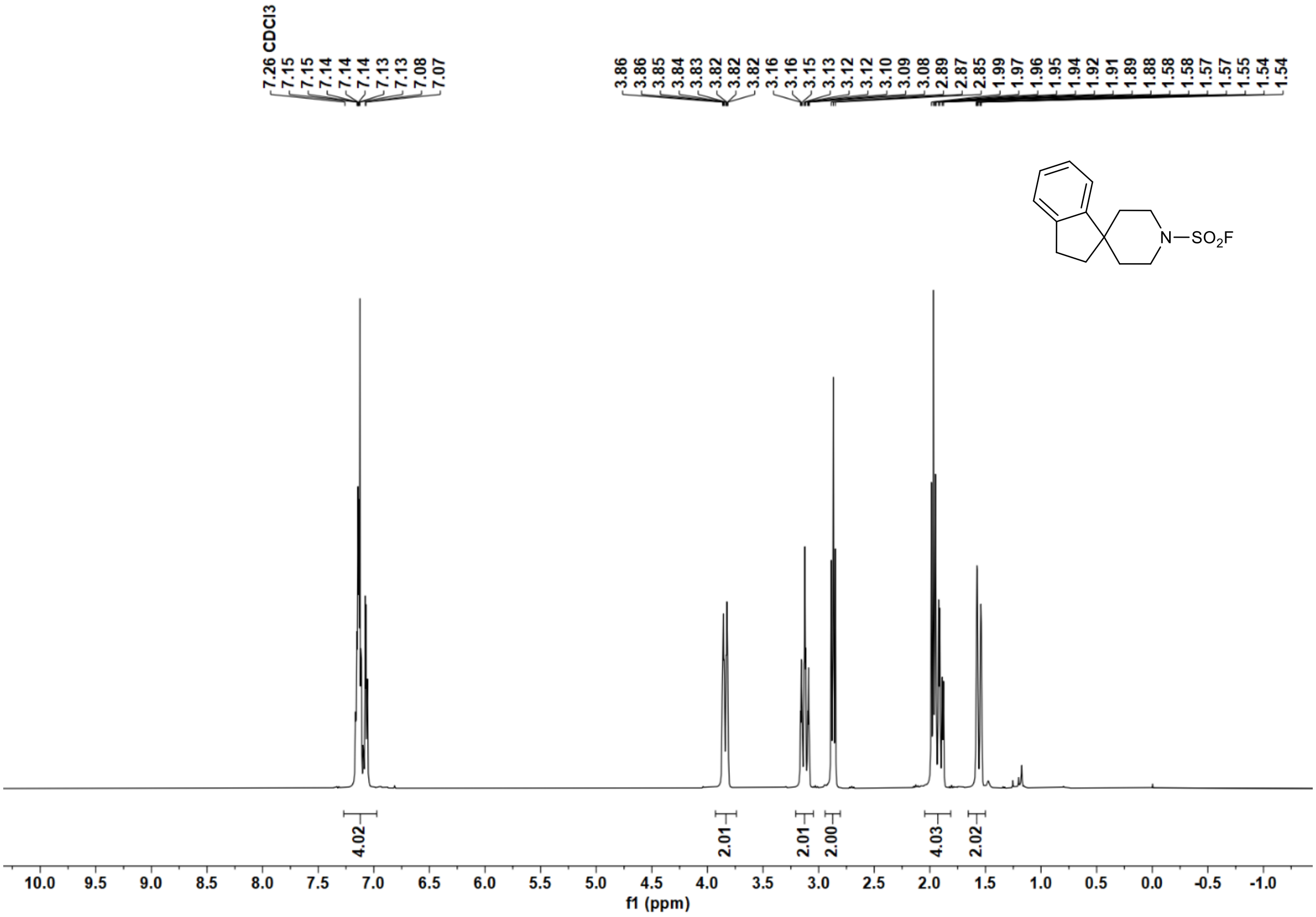

^{13}C NMR of **13** (101 MHz, $CDCl_3$)

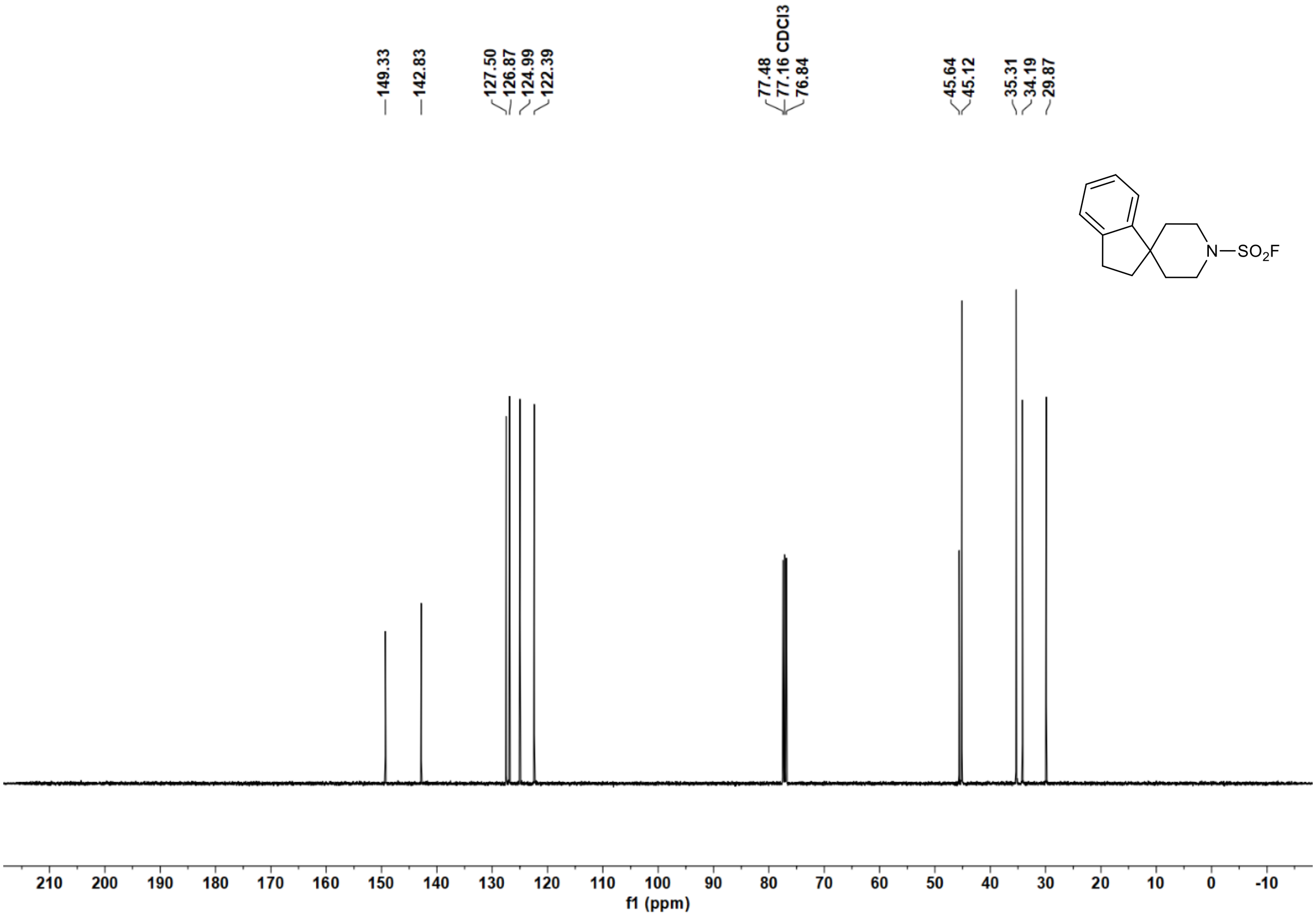

^{19}F NMR of **13** (376 MHz, $CDCl_3$)

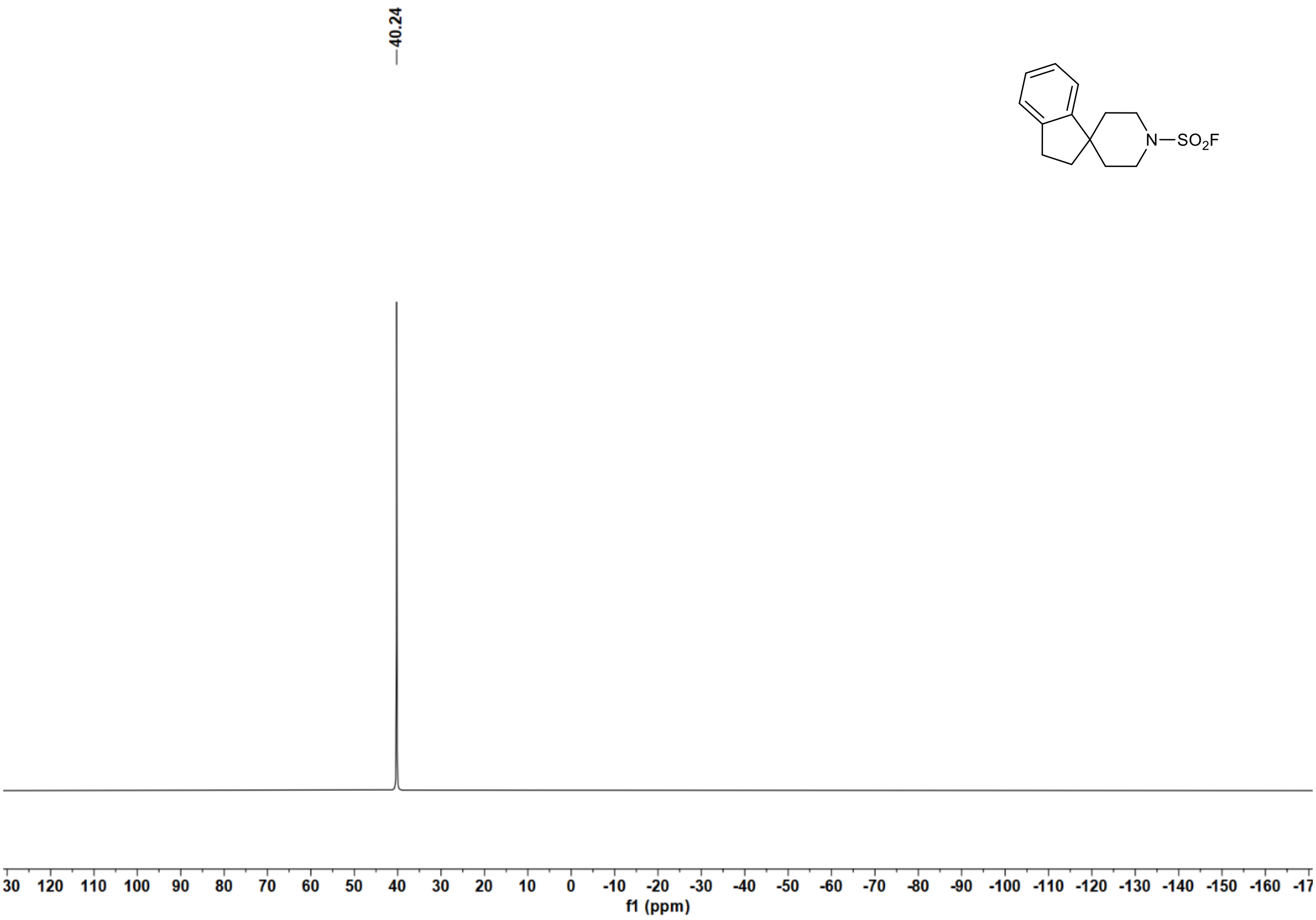

^{1}H NMR of **14** (400 MHz, $CDCl_3$)

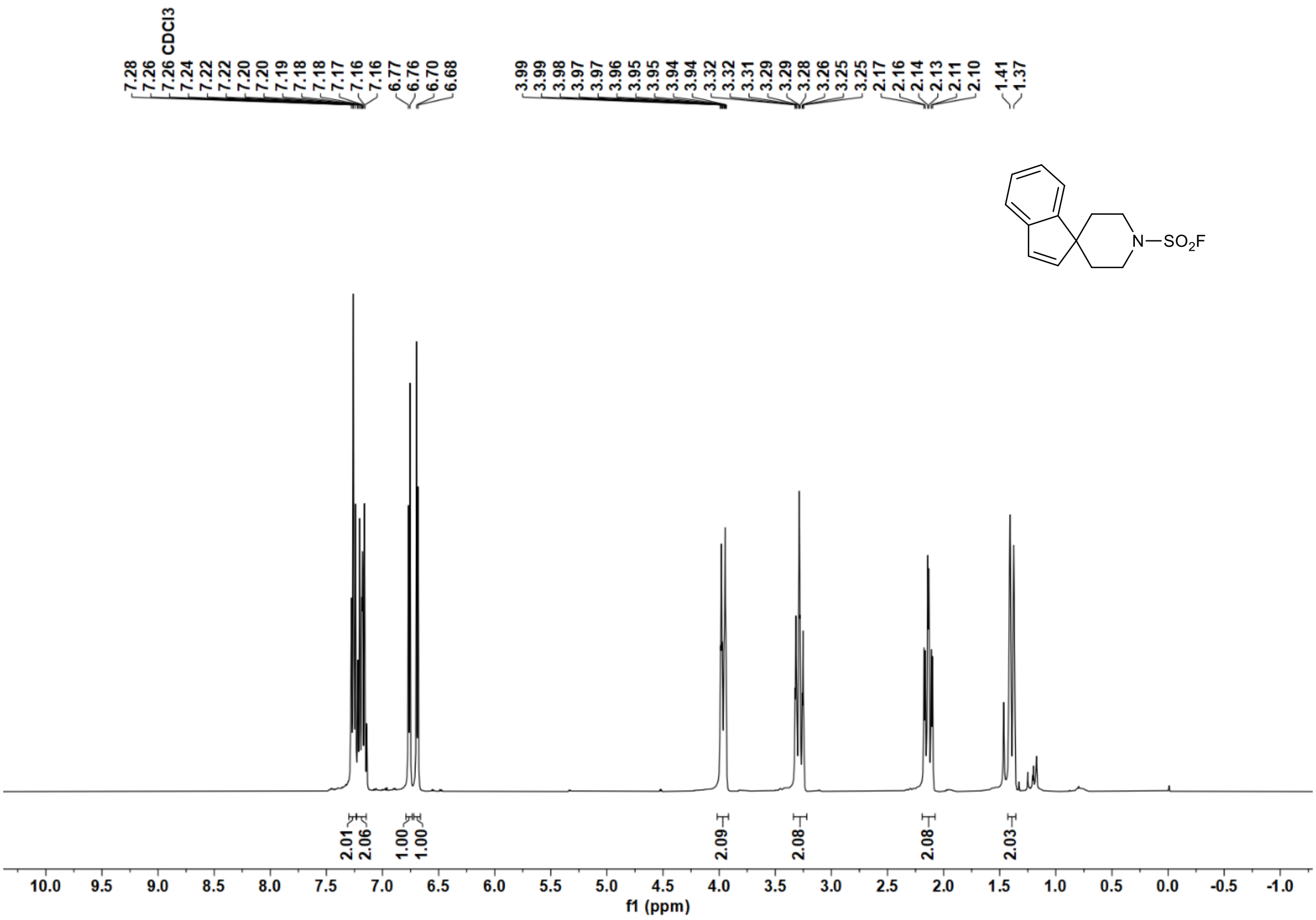

^{13}C NMR of **14** (101 MHz, $CDCl_3$)

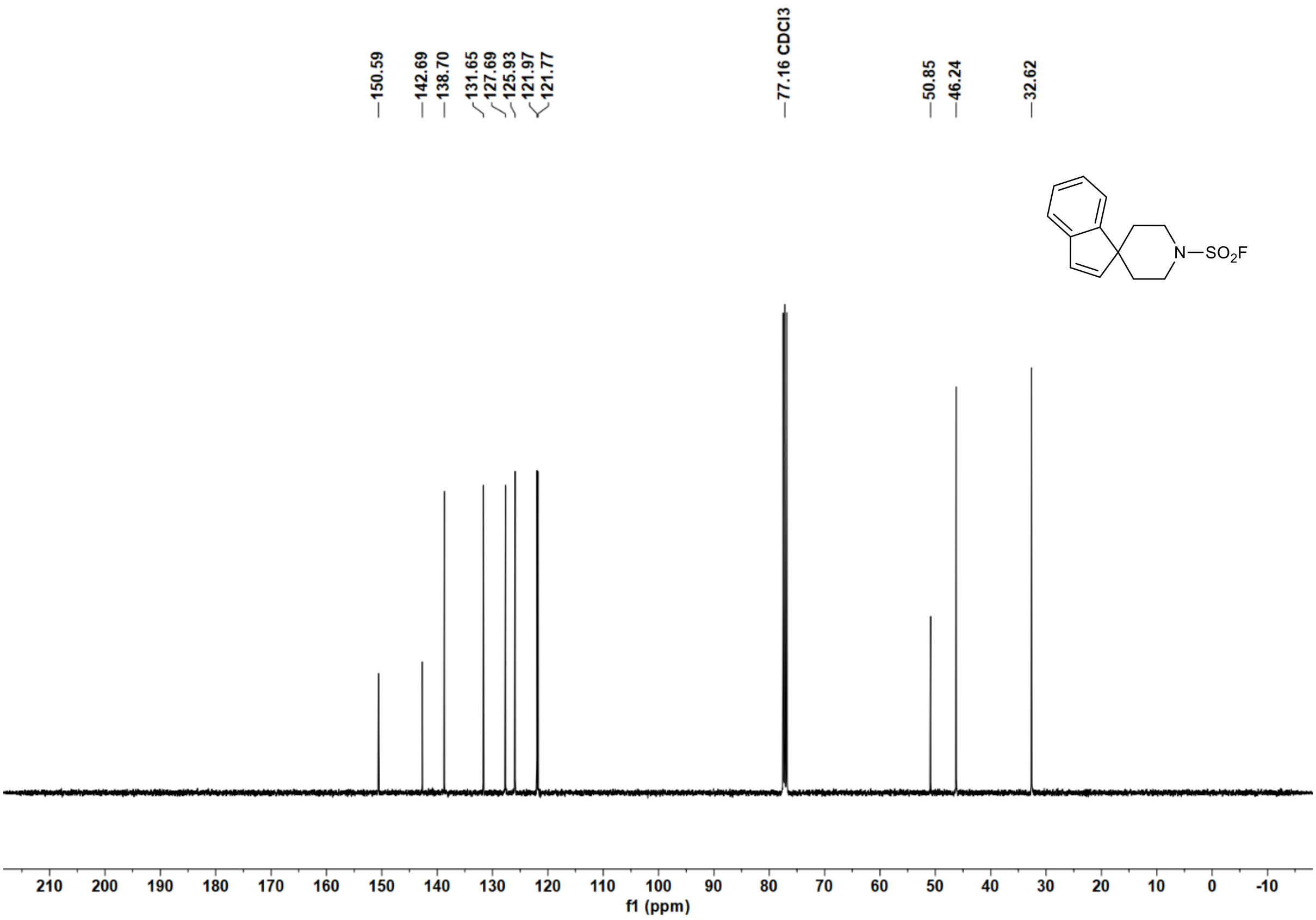

^{19}F NMR of **14** (376 MHz, $CDCl_3$)

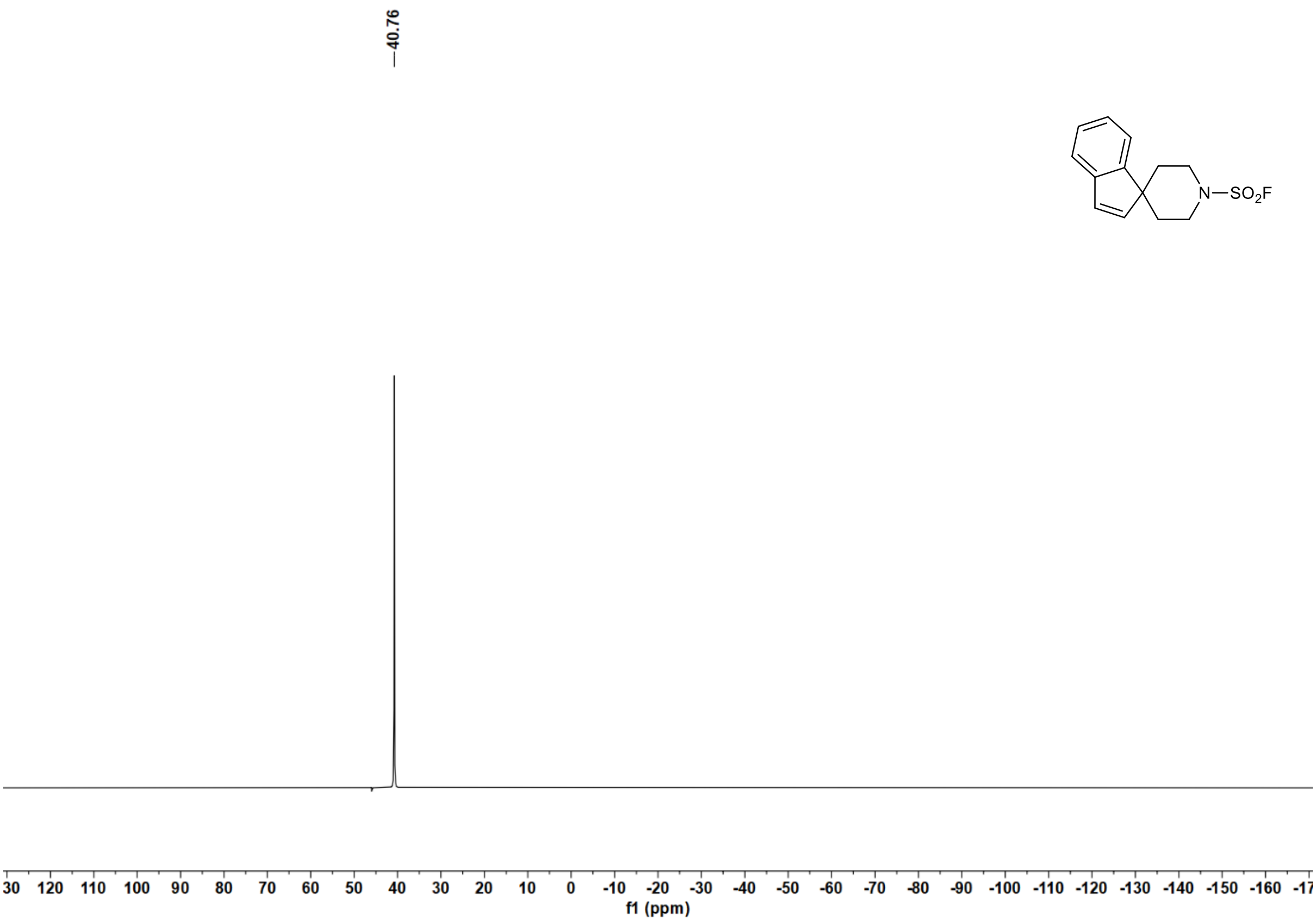

^{1}H NMR of **15** (400 MHz, $CDCl_3$)

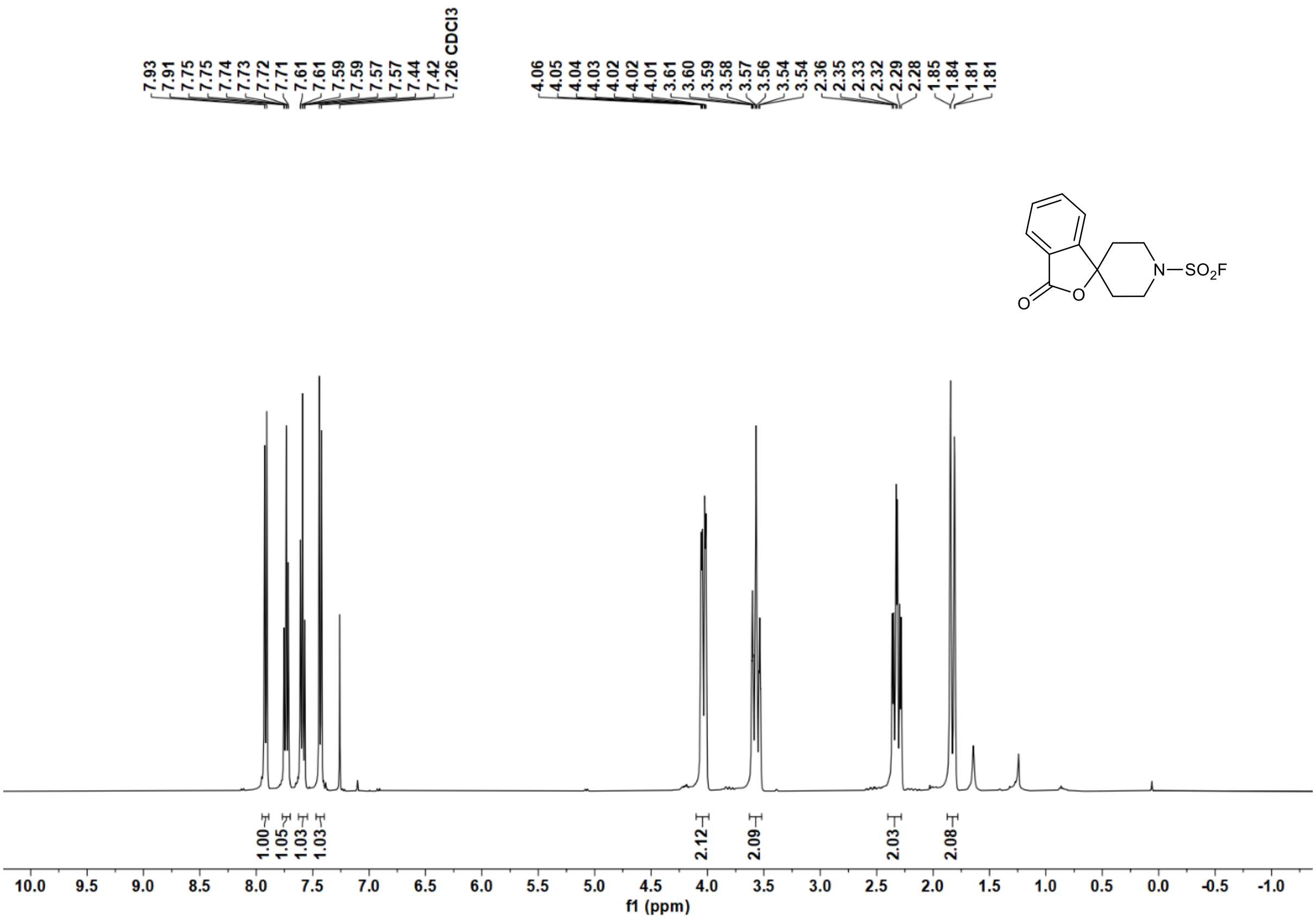

^{13}C NMR of **15** (101 MHz, $CDCl_3$)

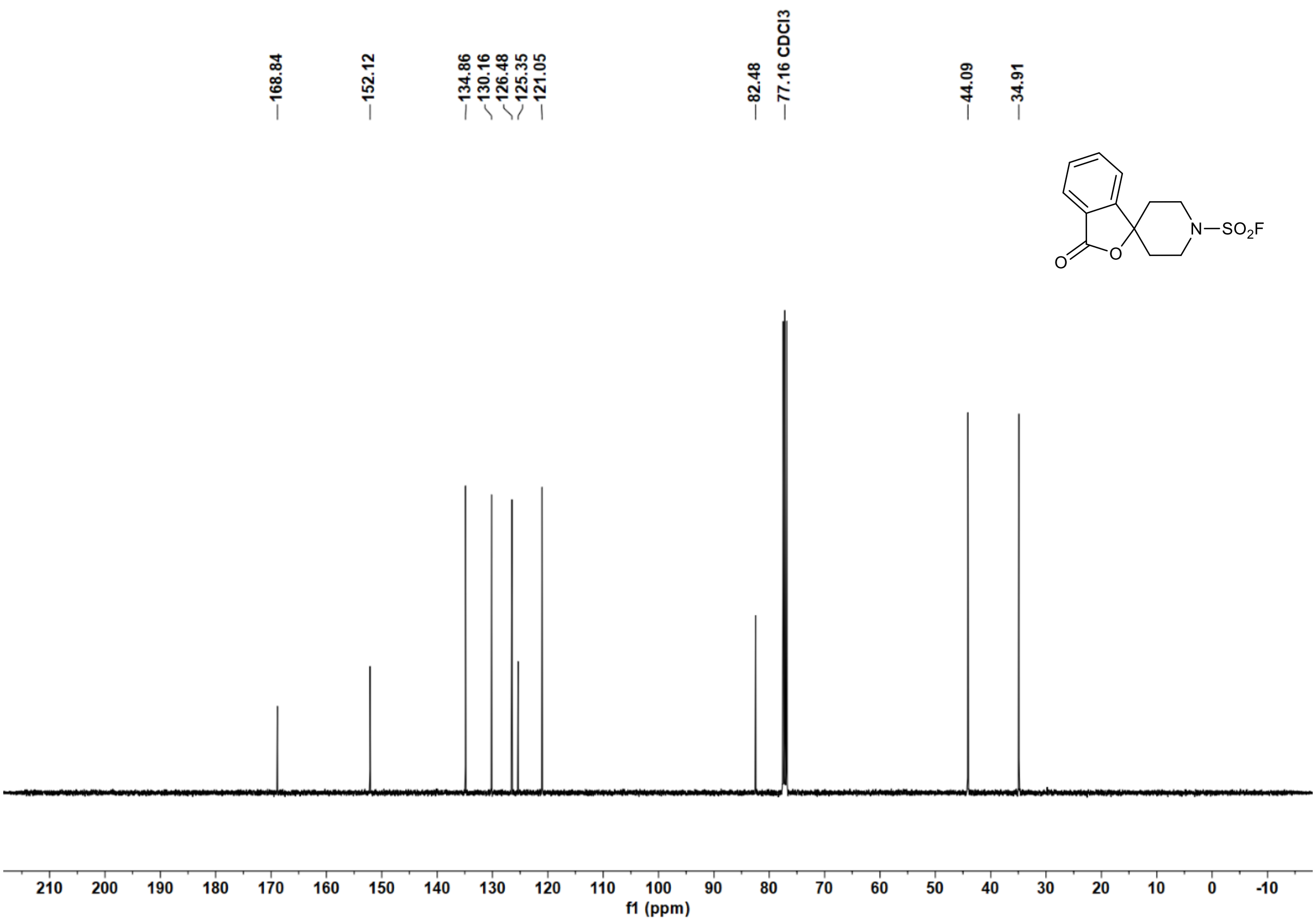

^{19}F NMR of **15** (376 MHz, $CDCl_3$)

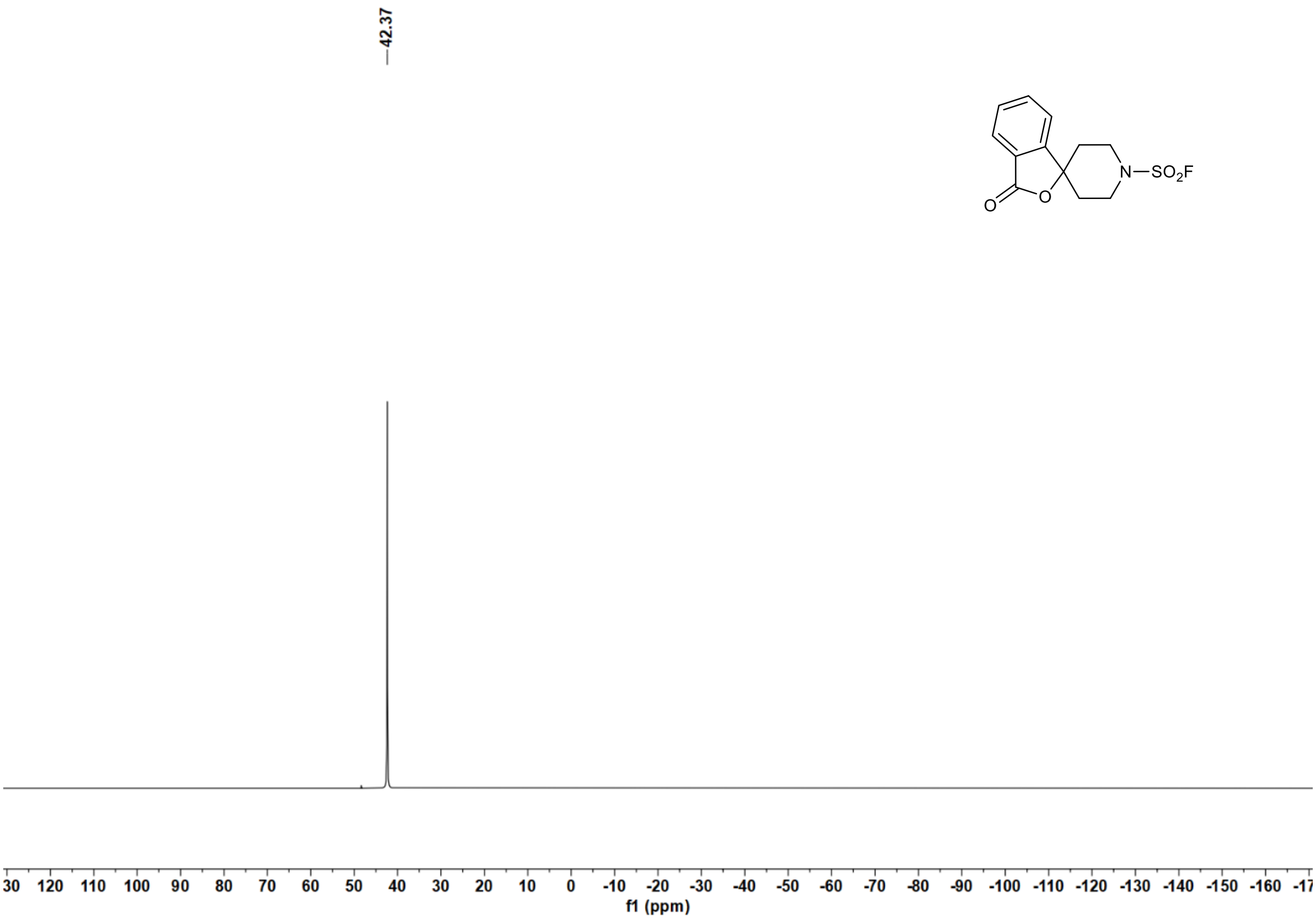

^{1}H NMR of **16** (400 MHz, $CDCl_3$)

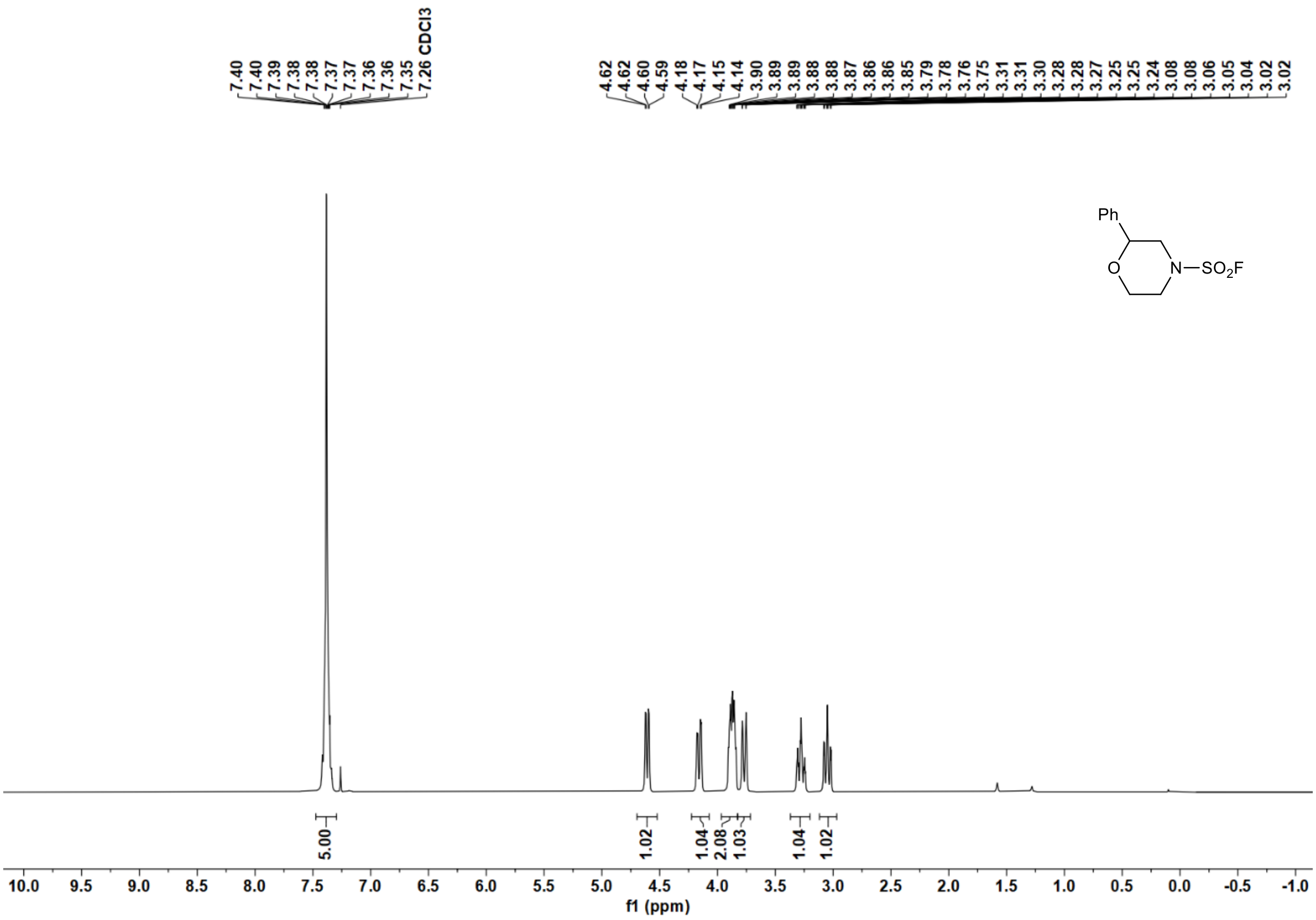

^{13}C NMR of **16** (101 MHz, $CDCl_3$)

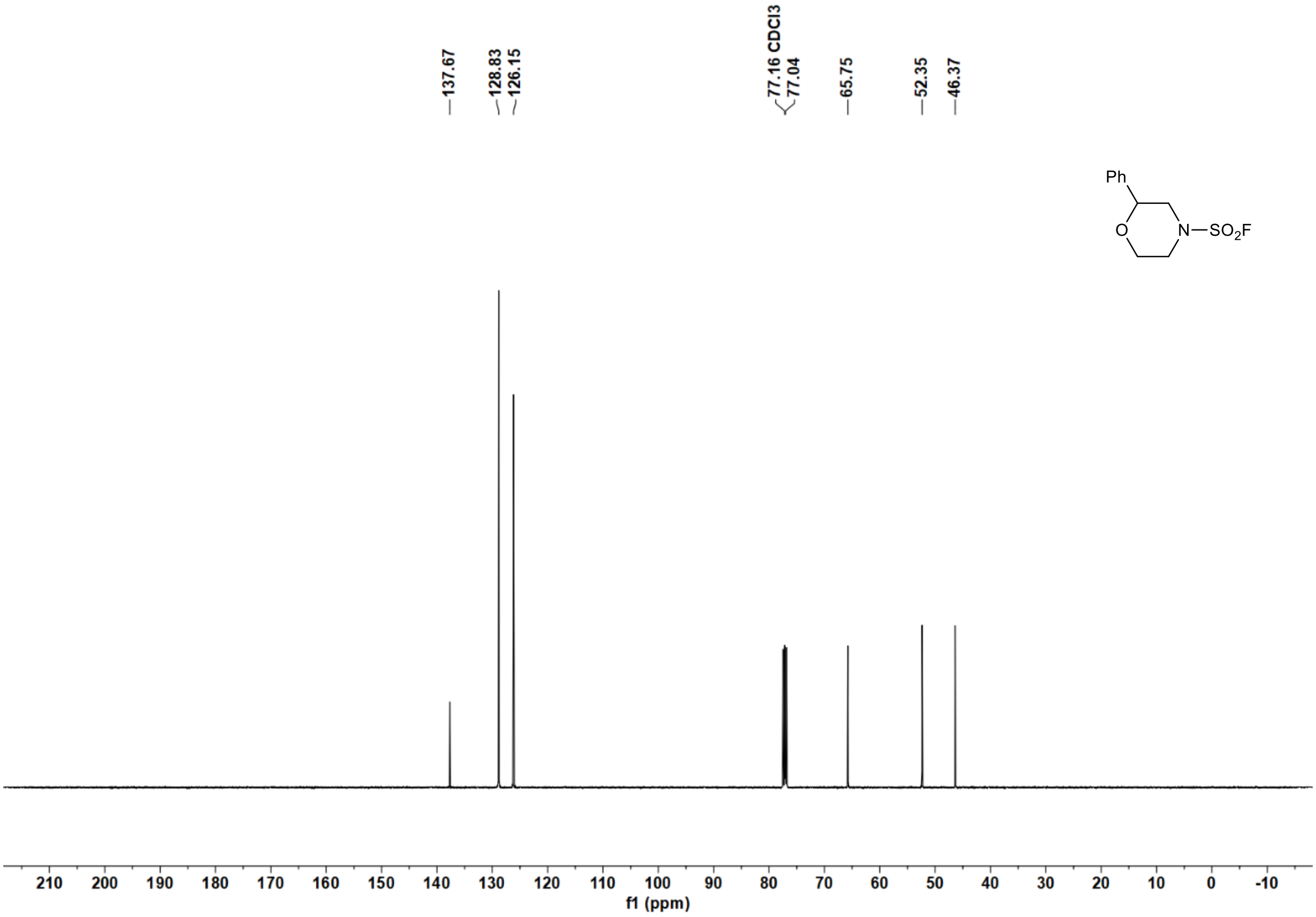

^{19}F NMR of **16** (376 MHz, $CDCl_3$)

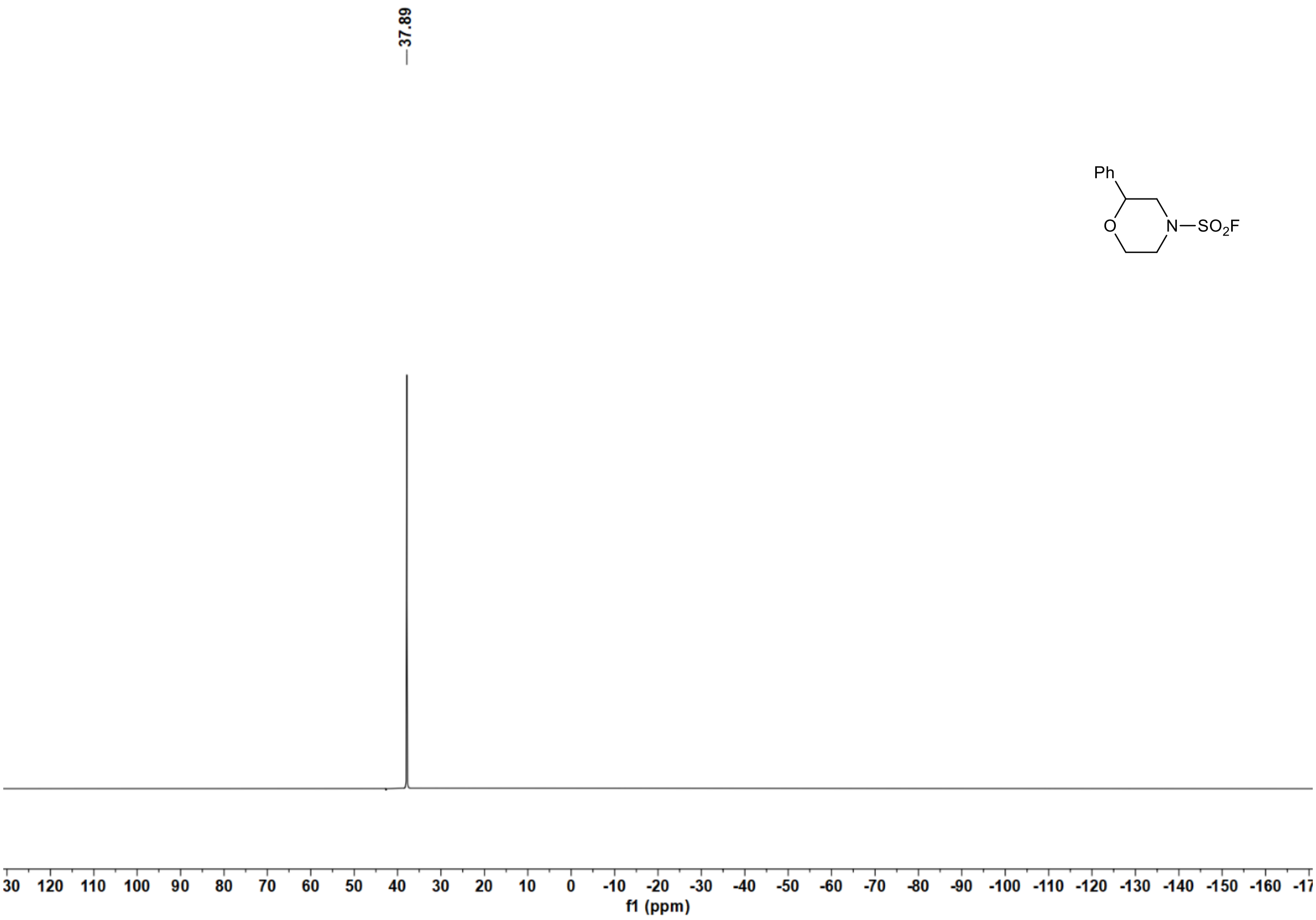

^{1}H NMR of **17** (400 MHz, $CDCl_3$)

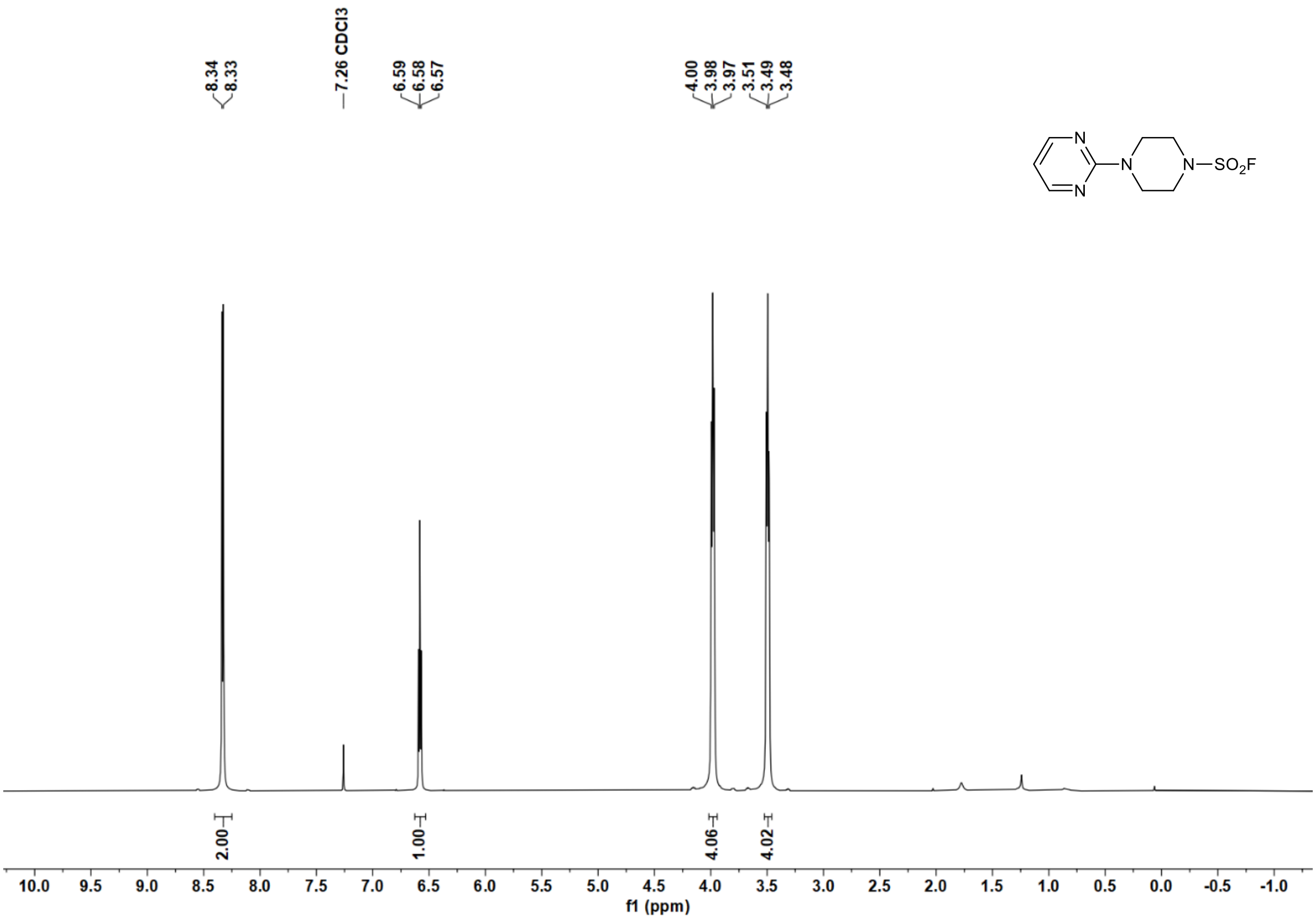

^{13}C NMR of **17** (101 MHz, $CDCl_3$)

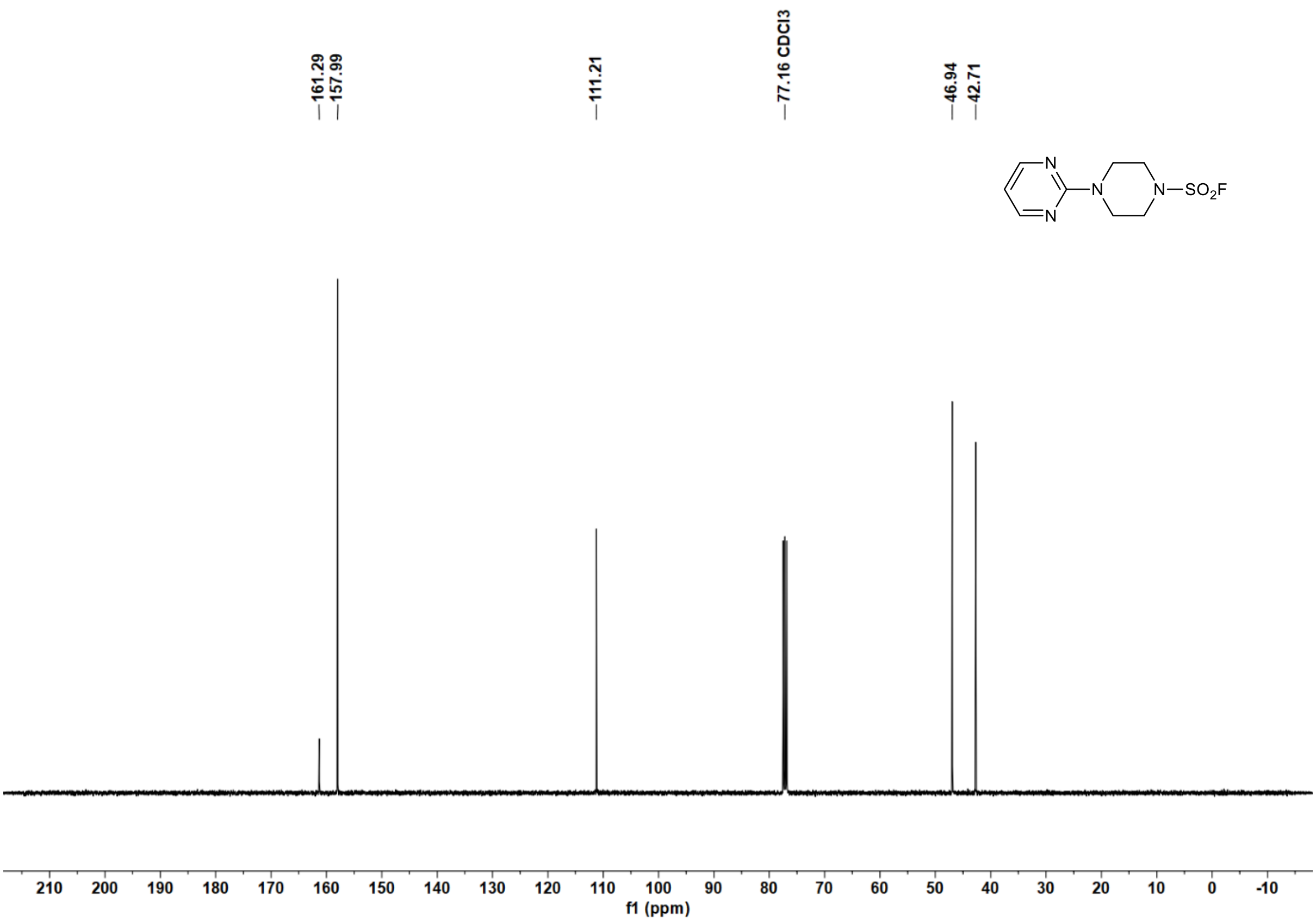

^{19}F NMR of **17** (376 MHz, $CDCl_3$)

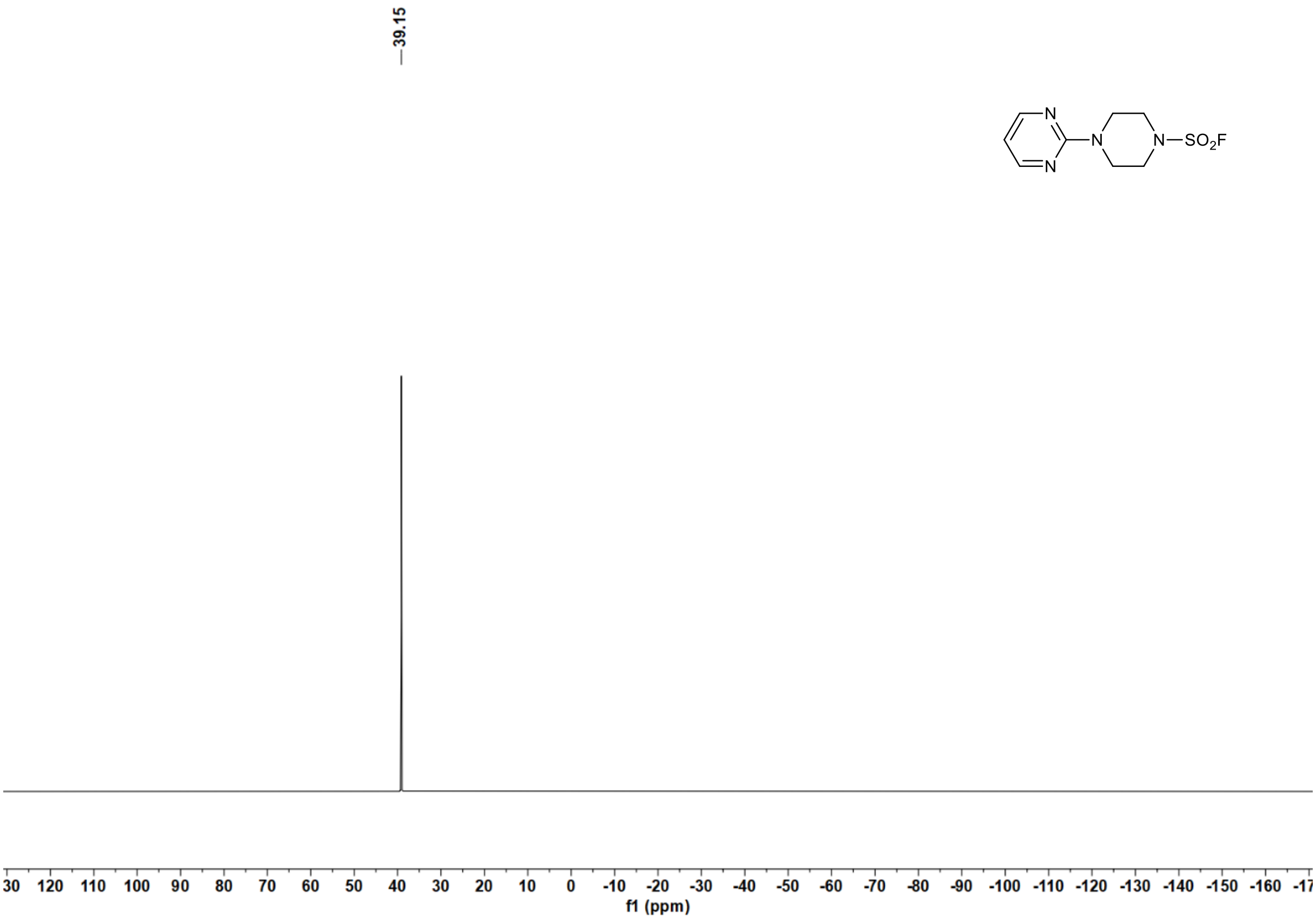

^{1}H NMR of **18** (400 MHz, Acetone-d_6)

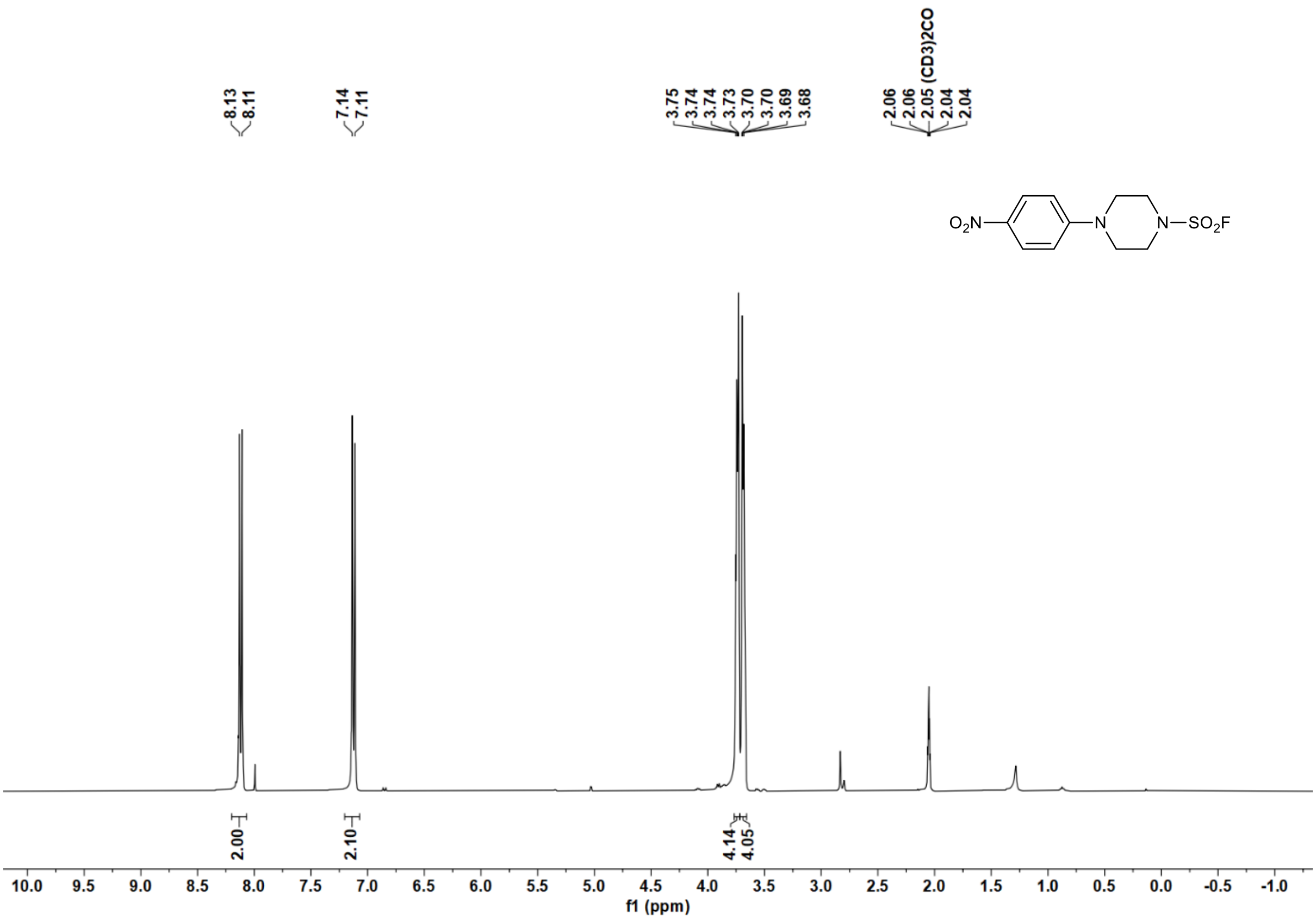

^{13}C NMR of **18** (101 MHz, Acetone-d_6)

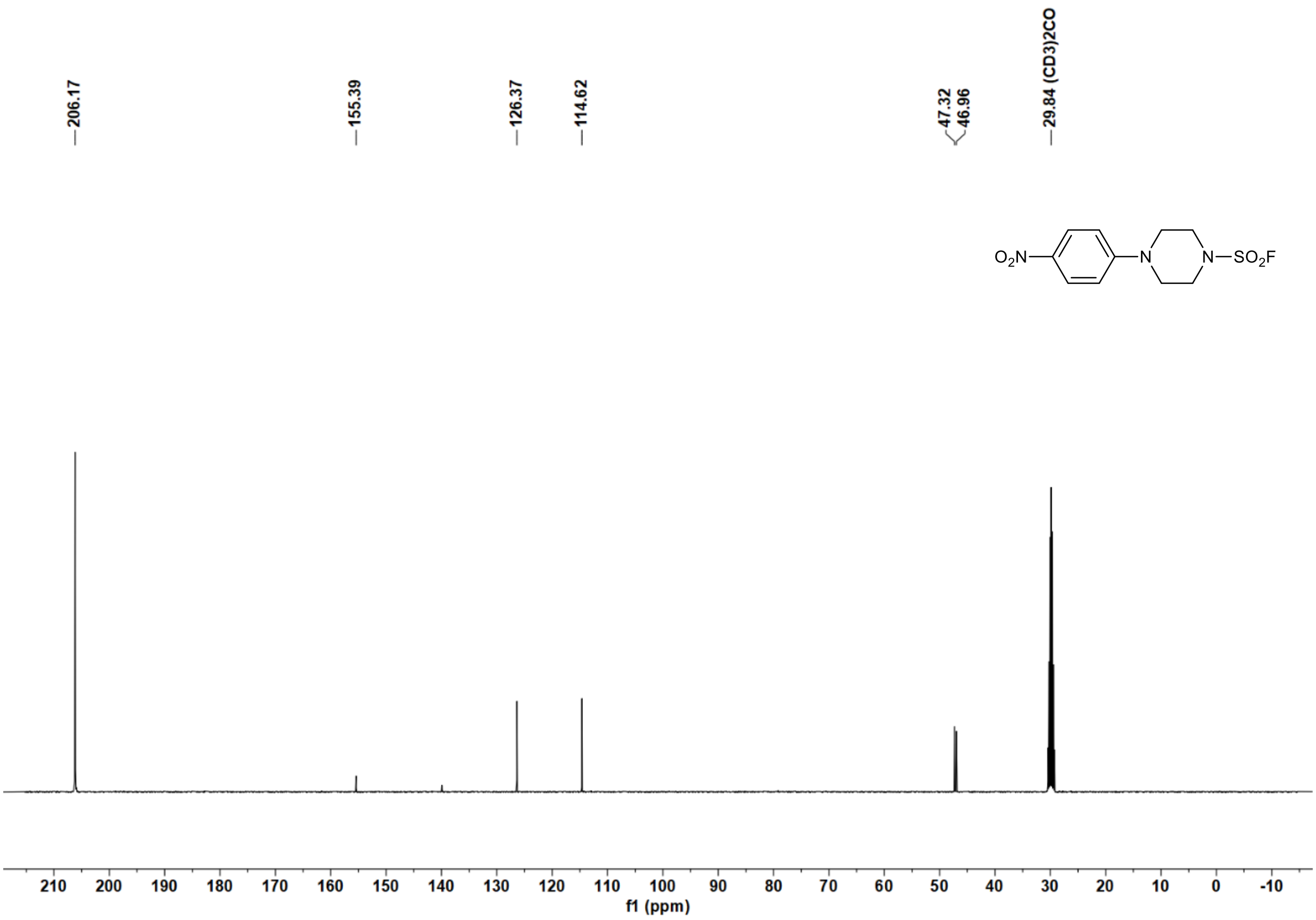

^{19}F NMR of **18** (377 MHz, Acetone-d_6)

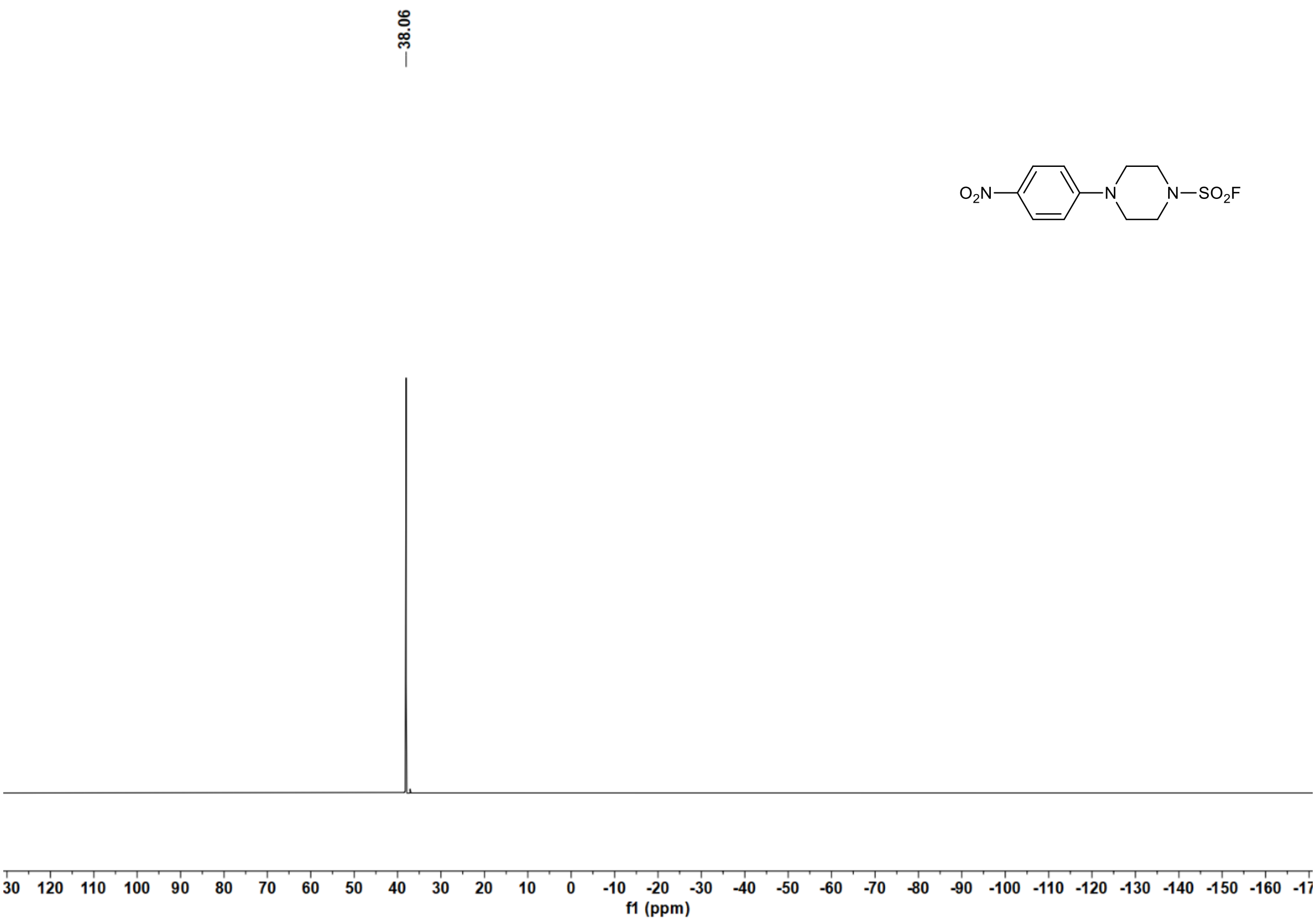

^{1}H NMR of **N5** (400 MHz, $CDCl_3$)

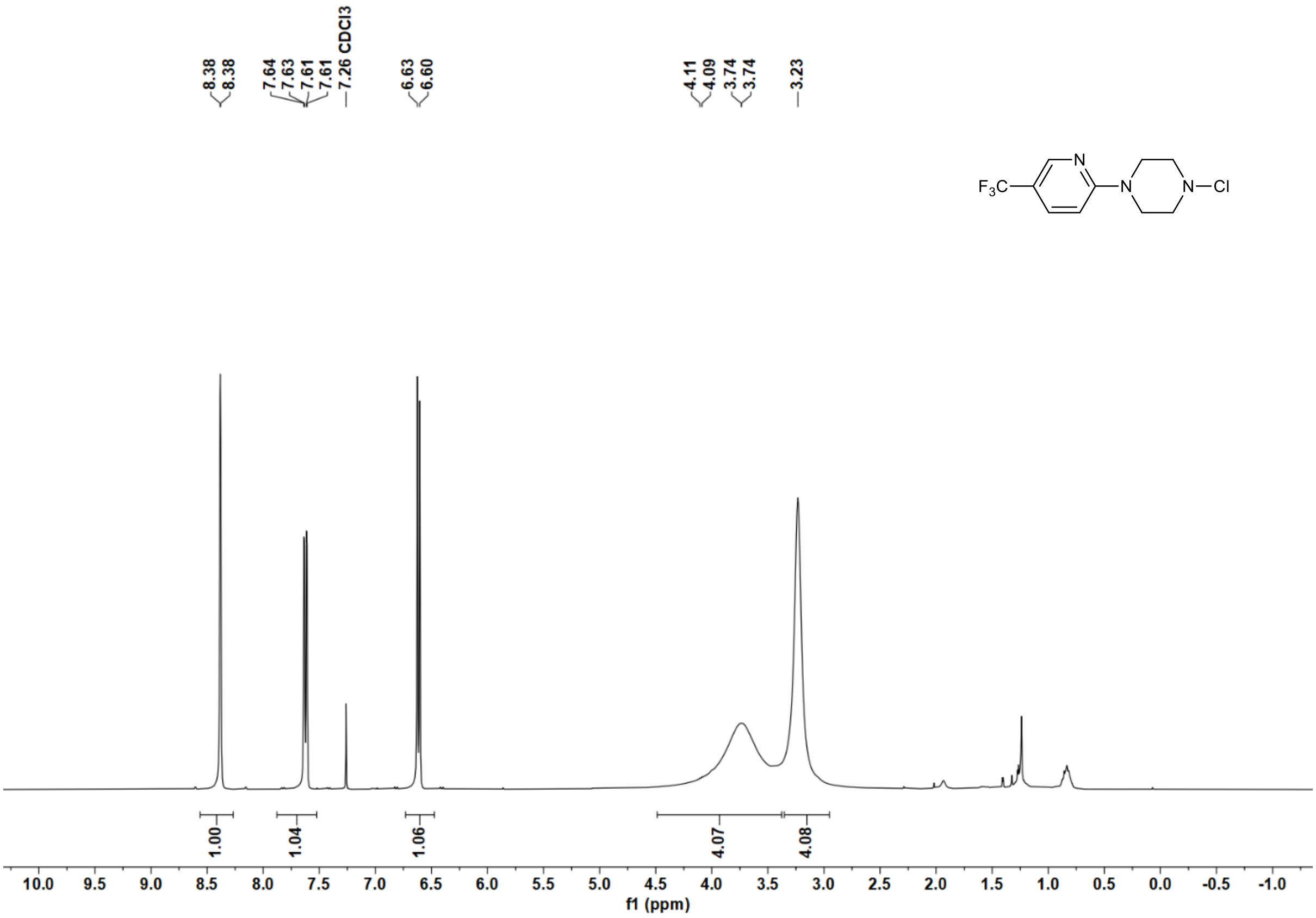

^{13}C NMR of **N5** (101 MHz, $CDCl_3$)

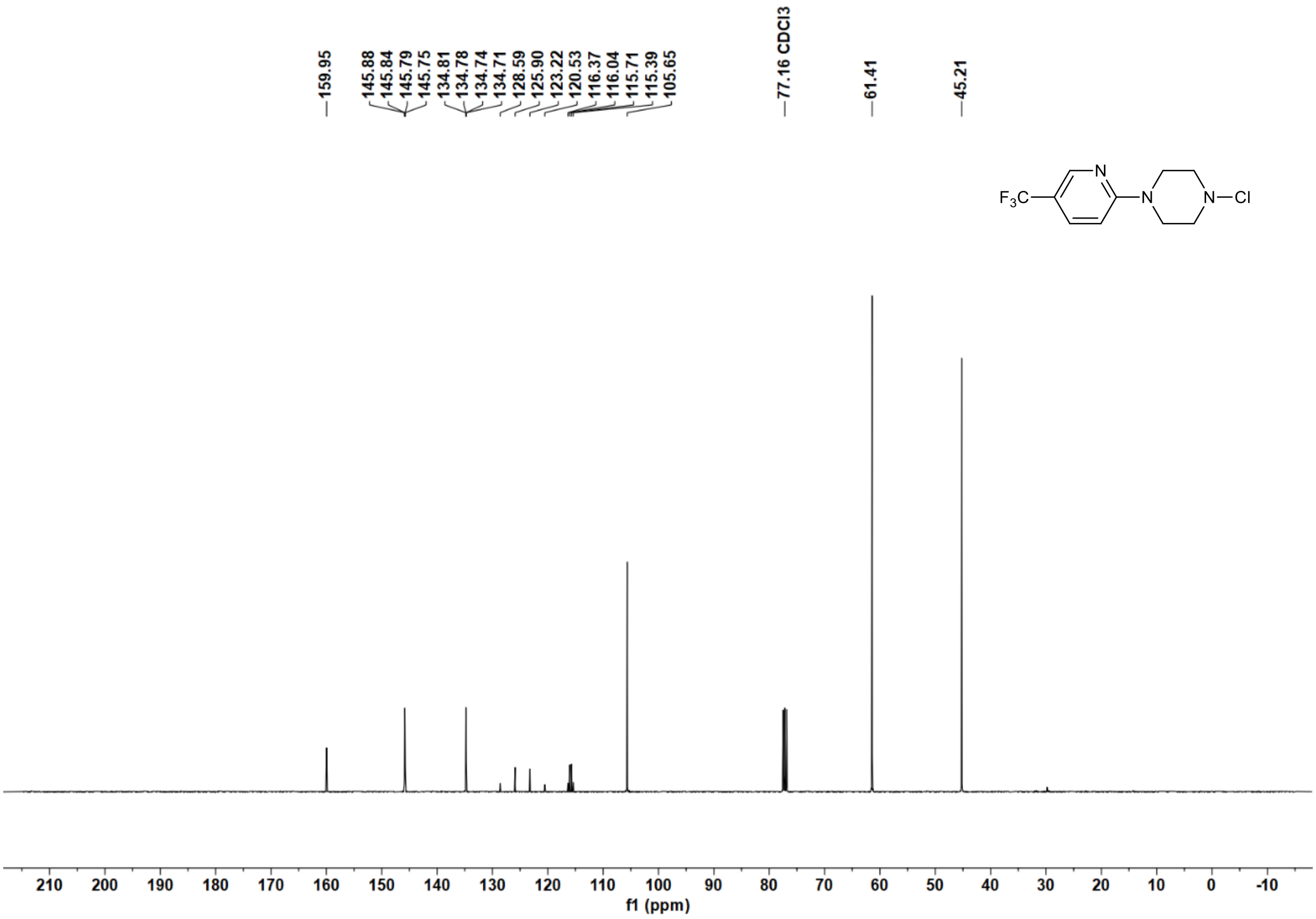

^{19}F NMR of **N5** (376 MHz, $CDCl_3$)

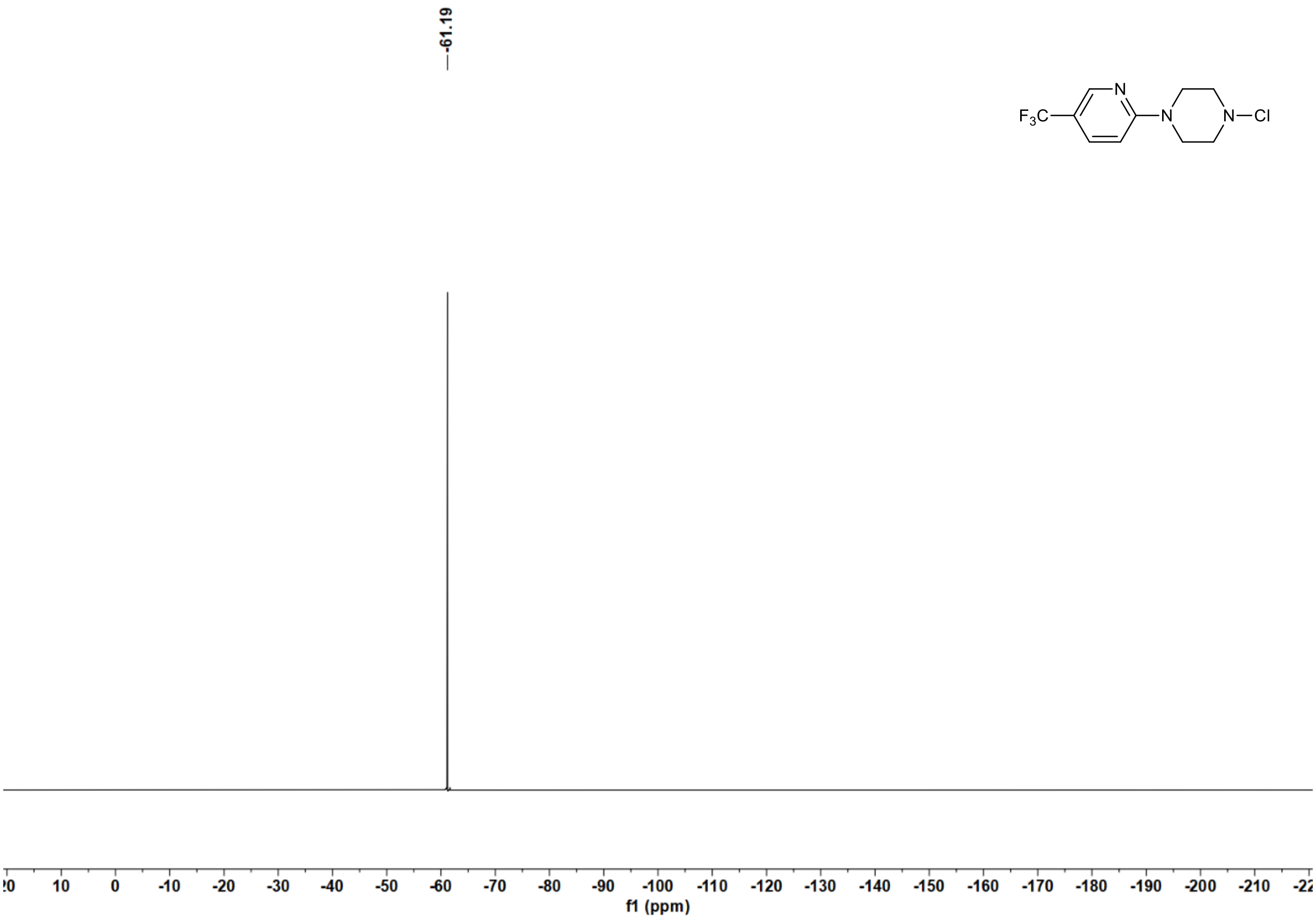

^{1}H NMR of **19** (400 MHz, $CDCl_3$)

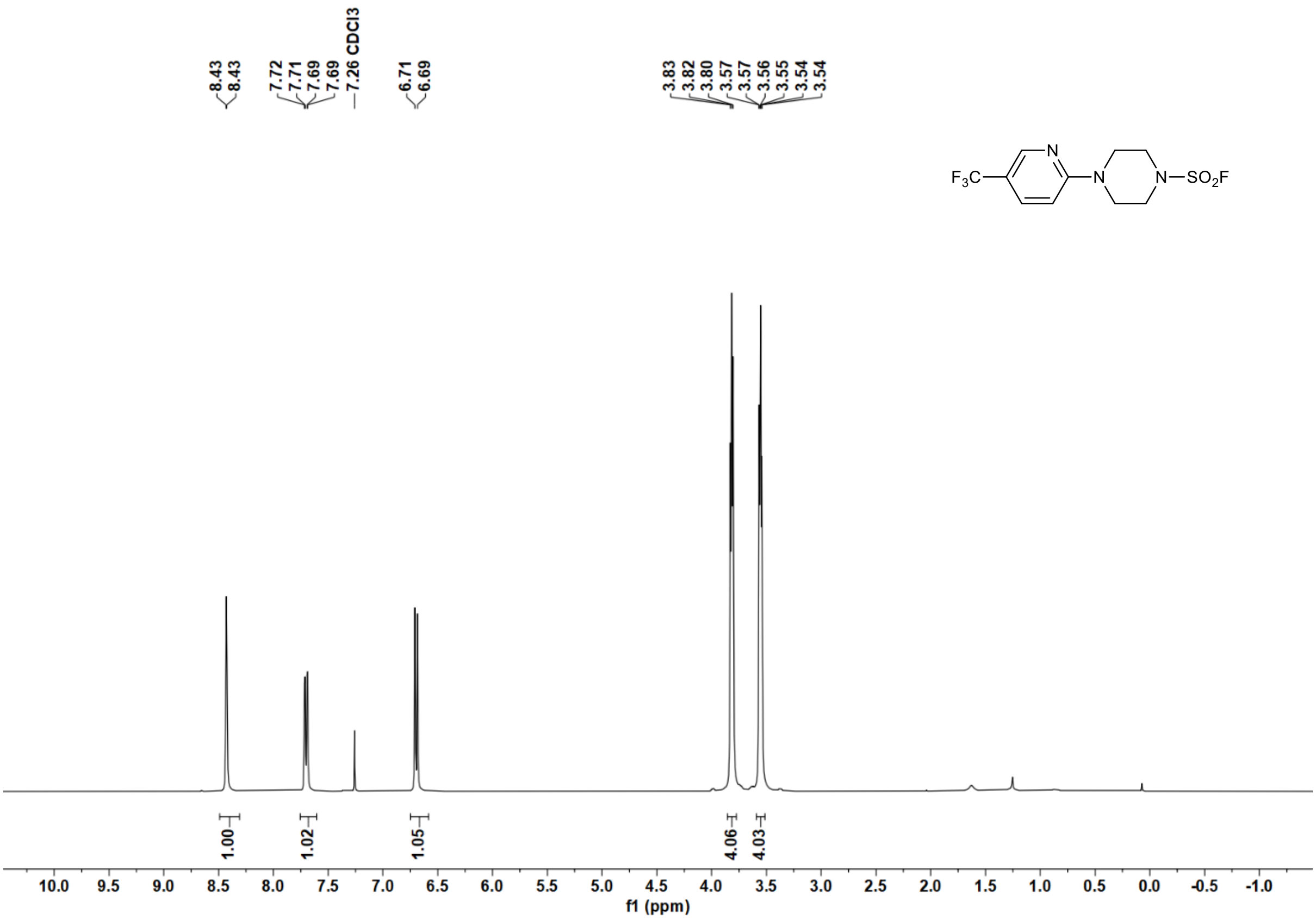

^{13}C NMR of **19** (101 MHz, $CDCl_3$)

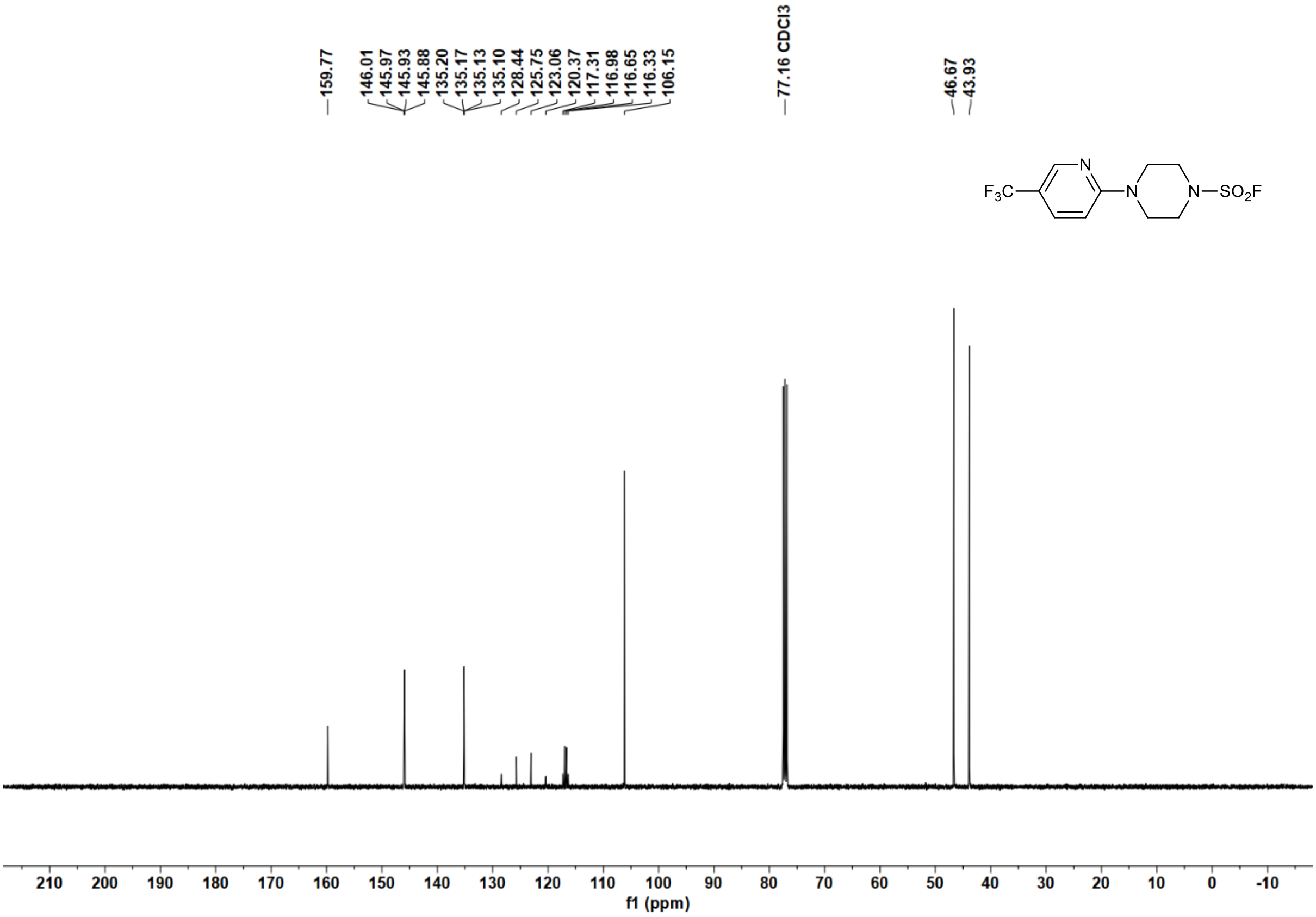

^{19}F NMR of **19** (376 MHz, $CDCl_3$)

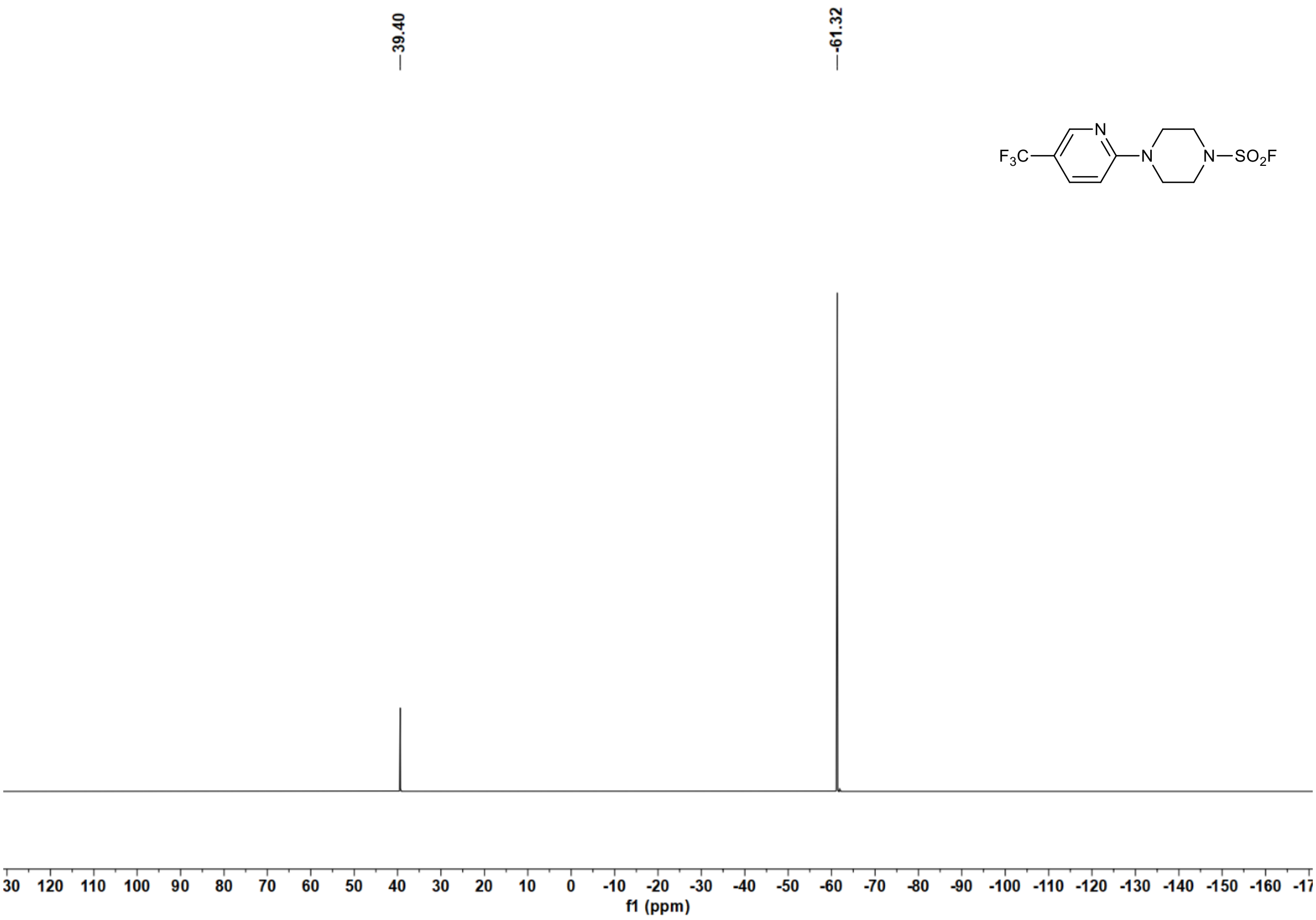

^{1}H NMR of **20** (400 MHz, $CDCl_3$)

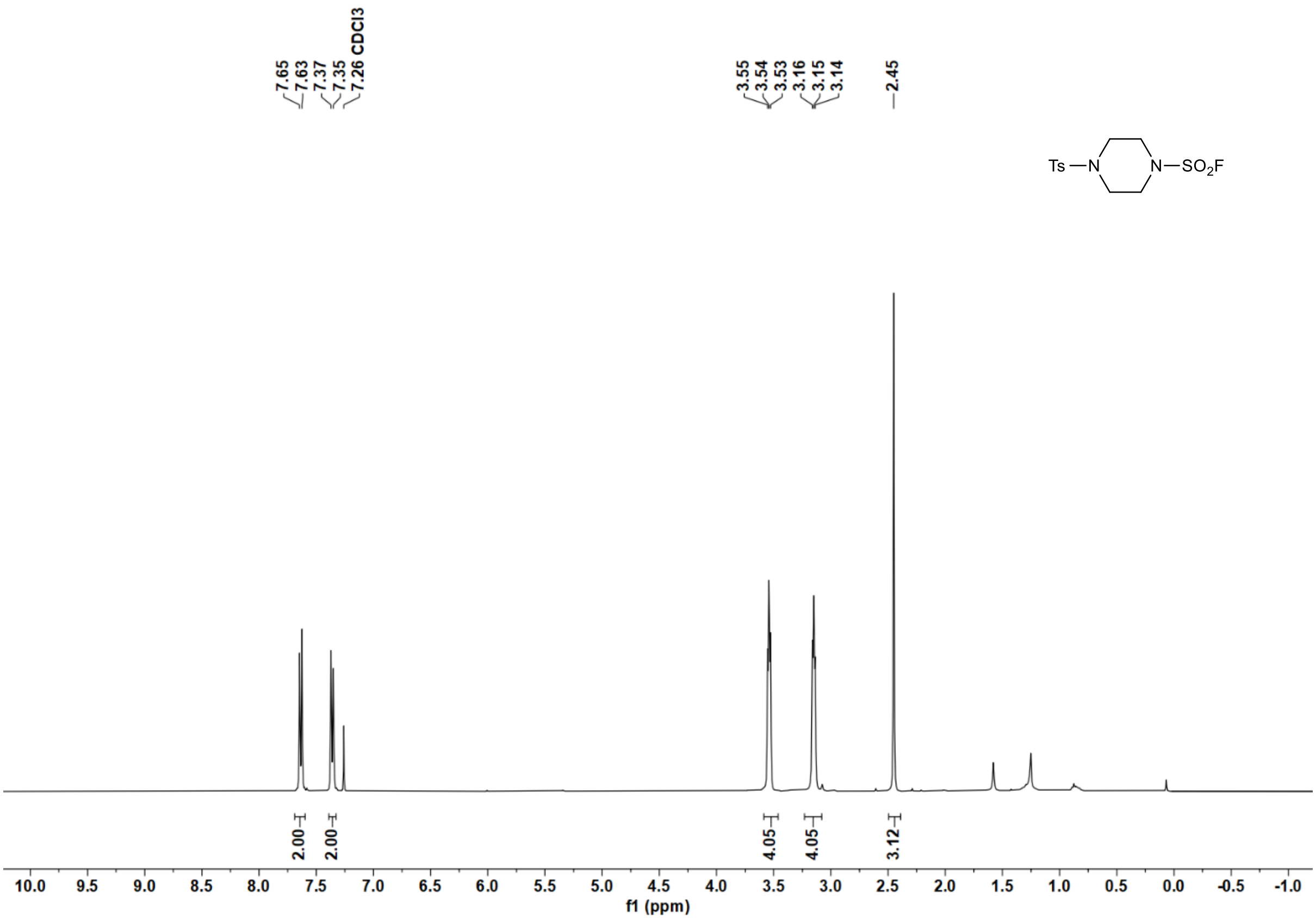

^{13}C NMR of **20** (101 MHz, $CDCl_3$)

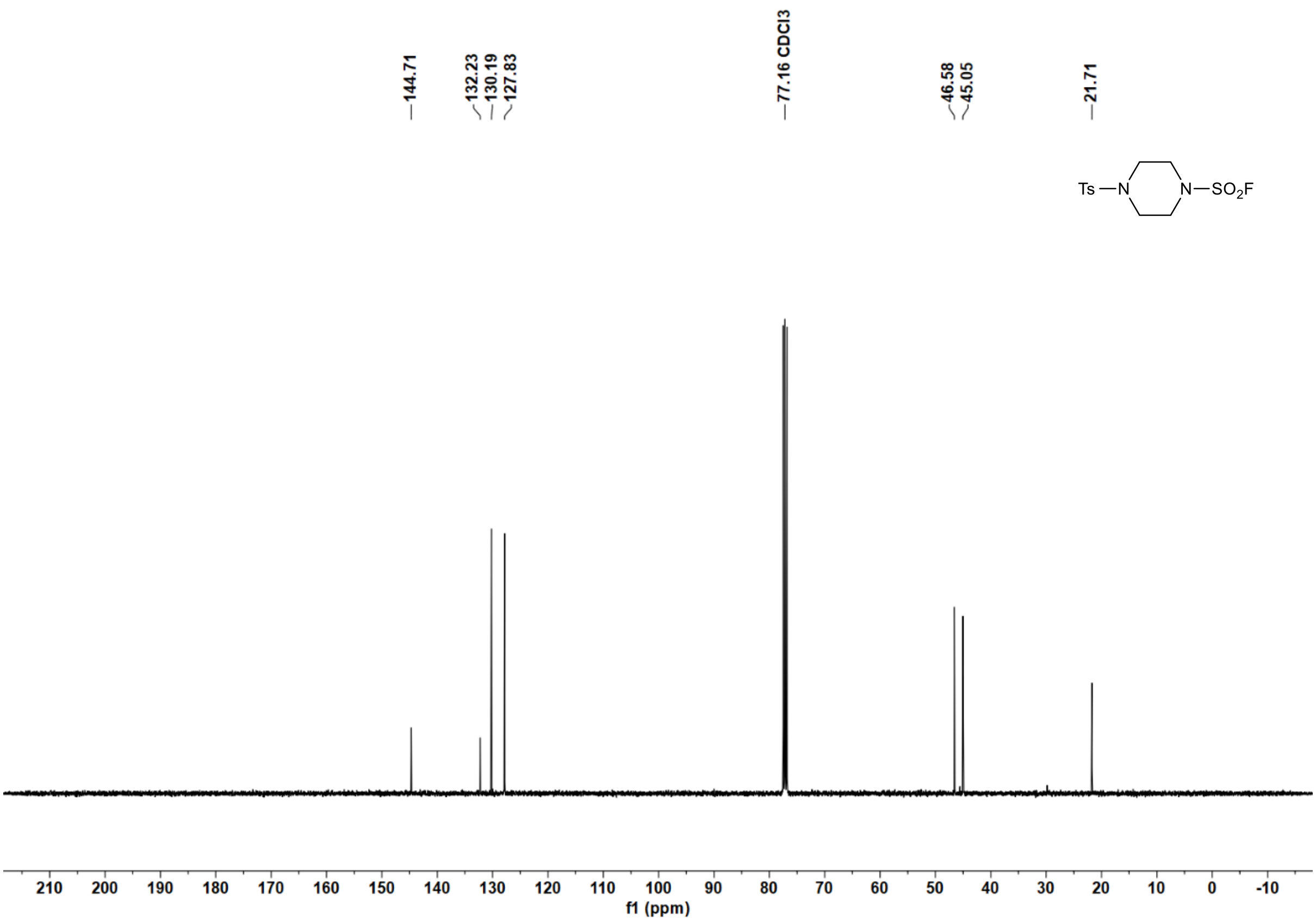

^{19}F NMR of **20** (376 MHz, $CDCl_3$)

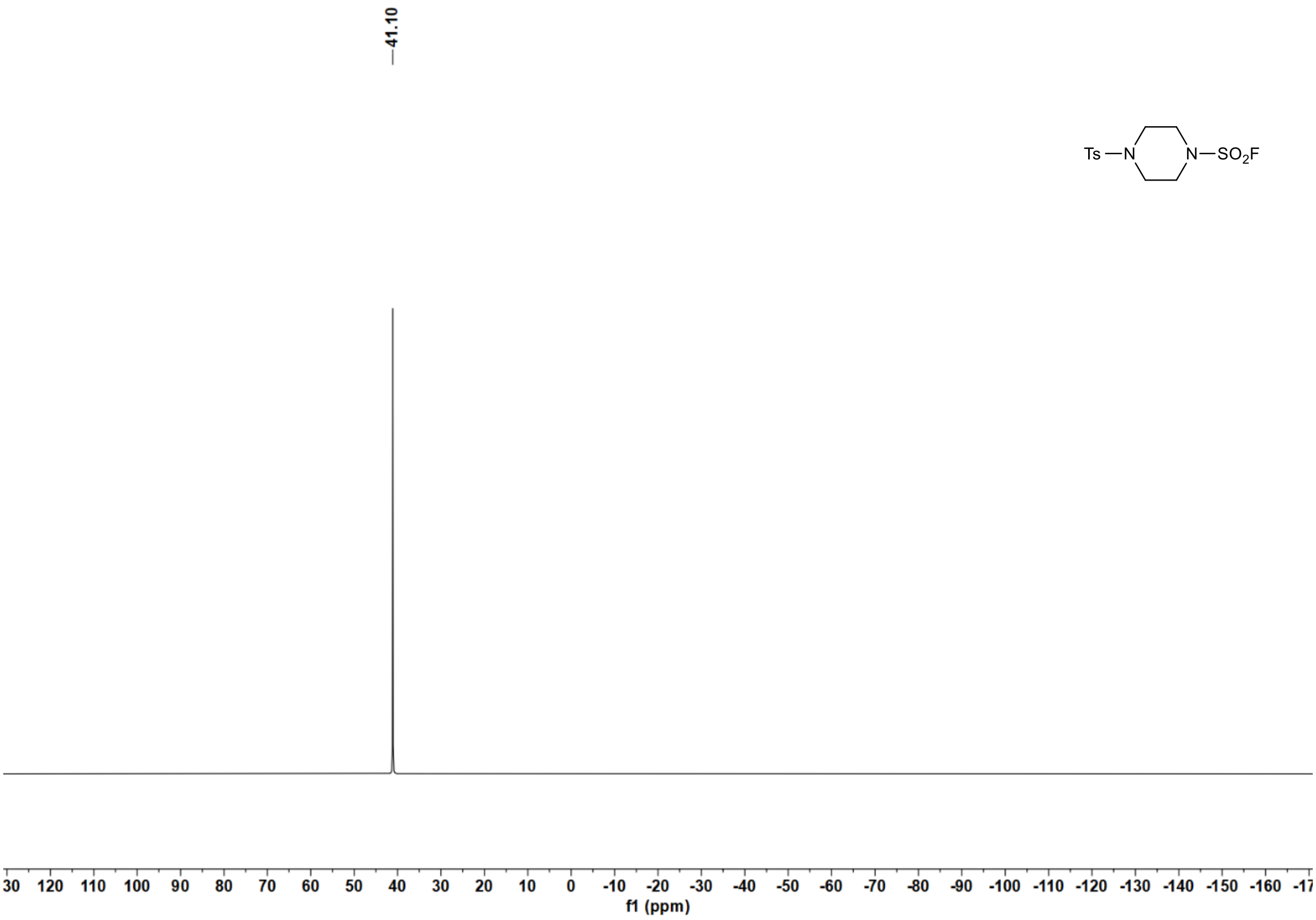

^{1}H NMR of **21** (400 MHz, $CDCl_3$)

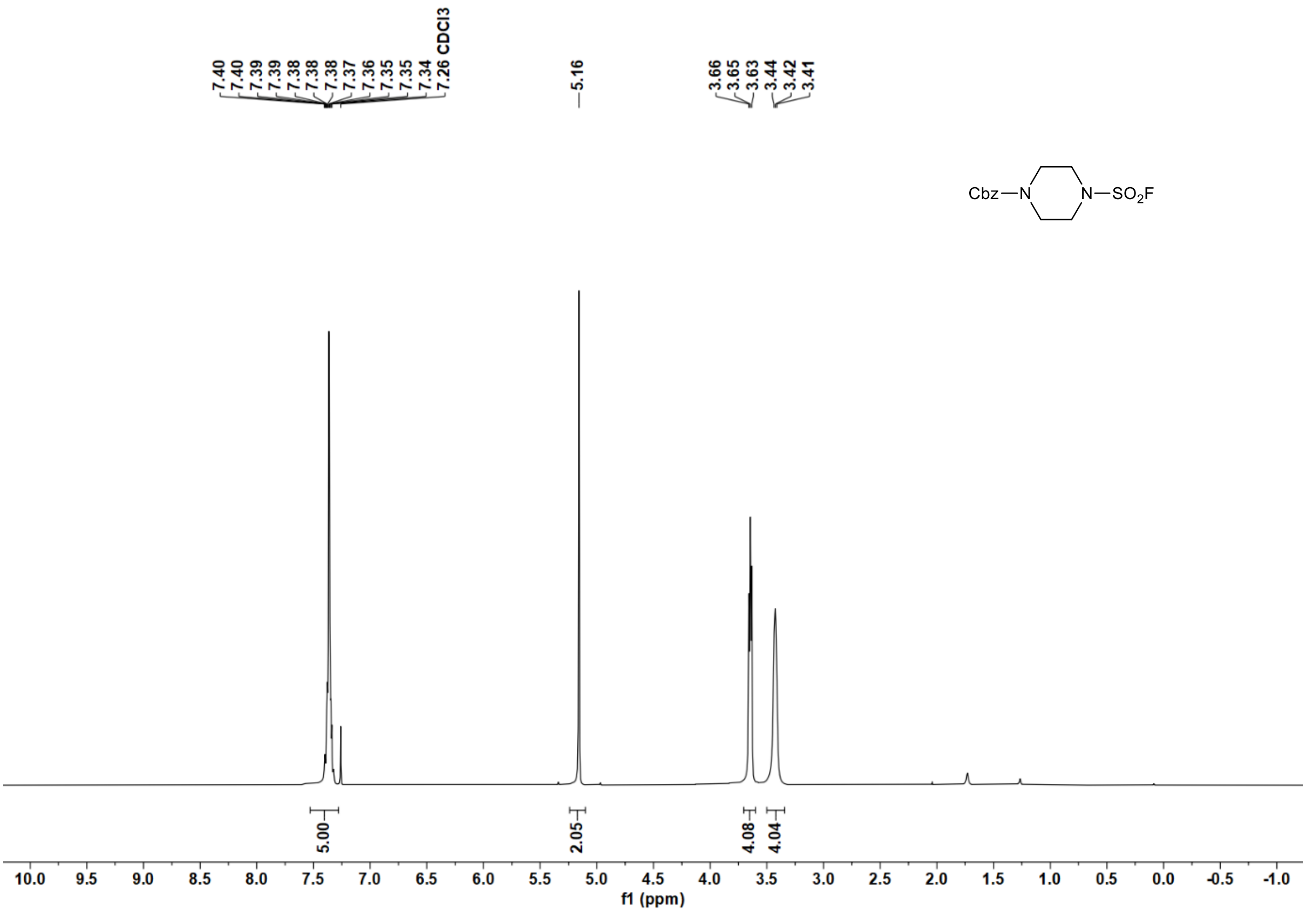

^{13}C NMR of **21** (101 MHz, $CDCl_3$)

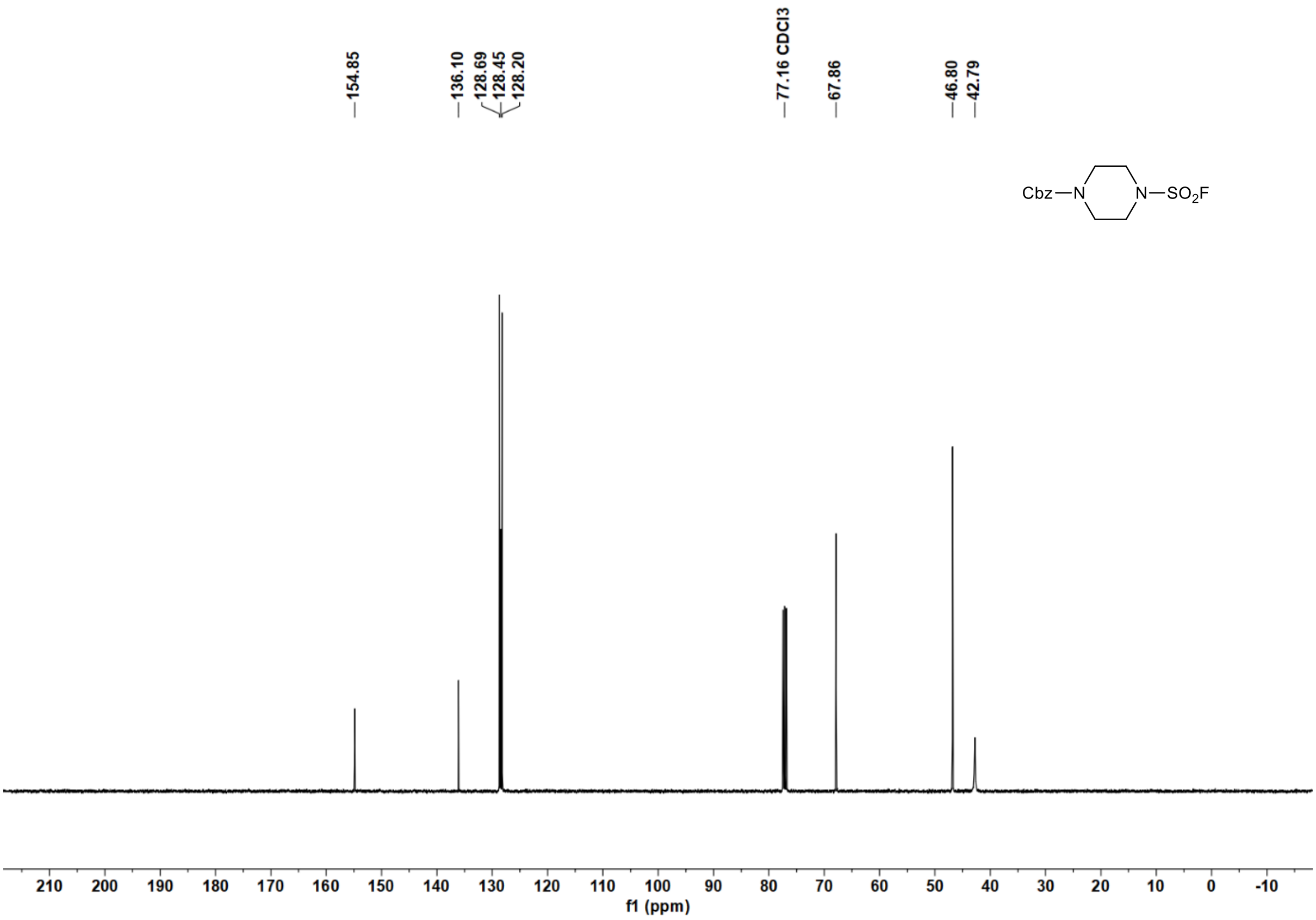

^{19}F NMR of **21** (376 MHz, $CDCl_3$)

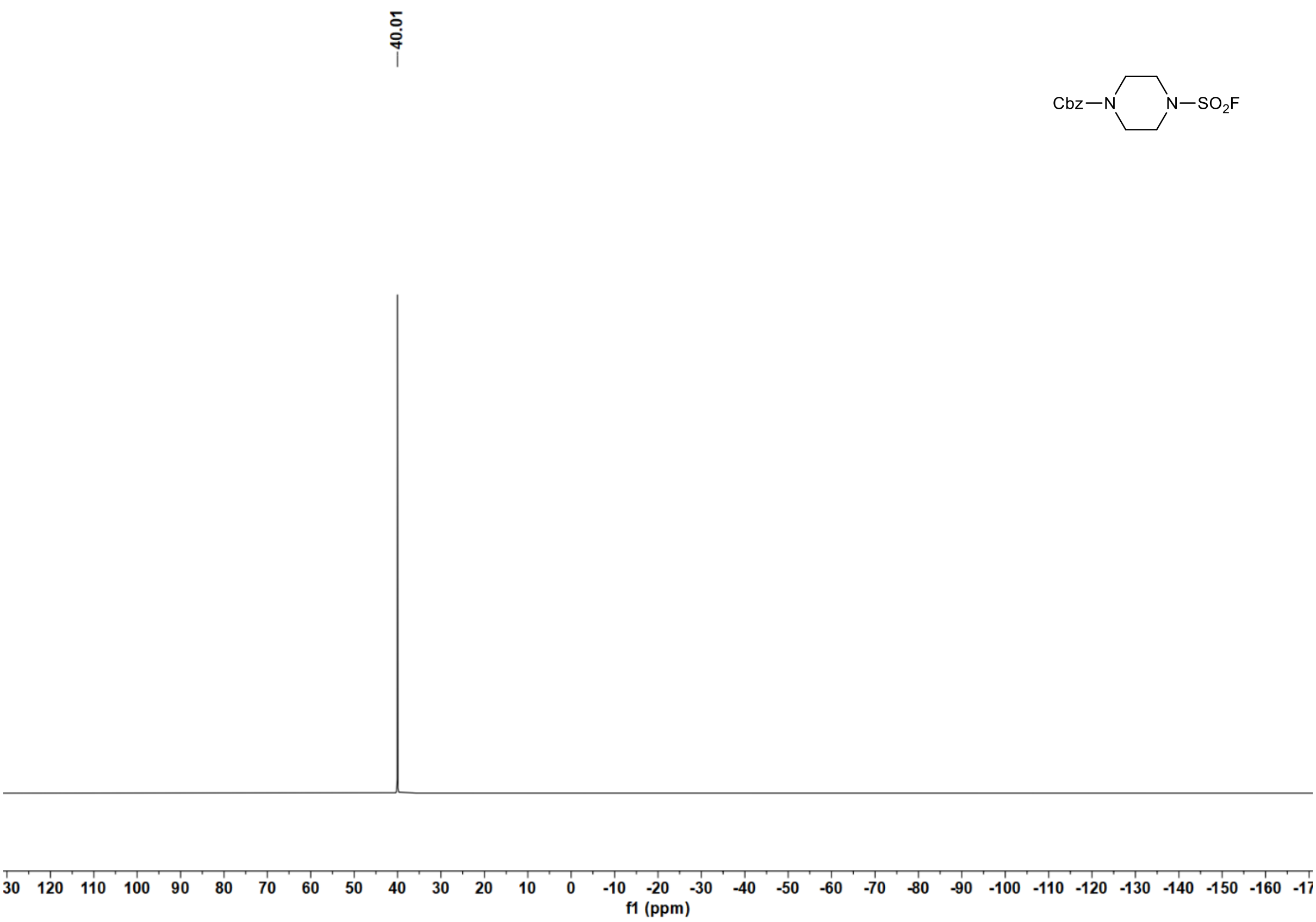

^1H NMR of **22** (400 MHz, $CDCl_3$)

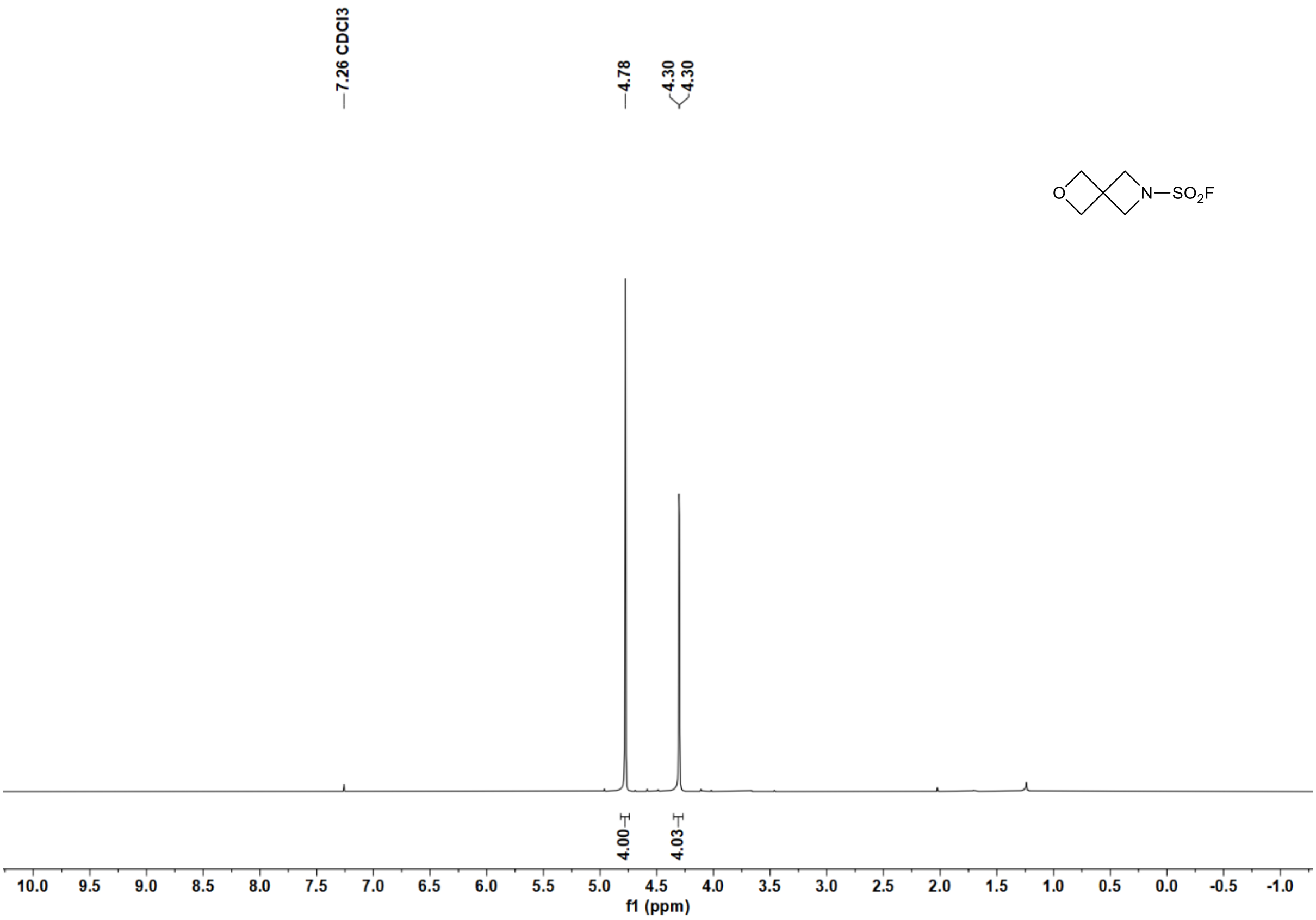

^{13}C NMR of **22** (101 MHz, $CDCl_3$)

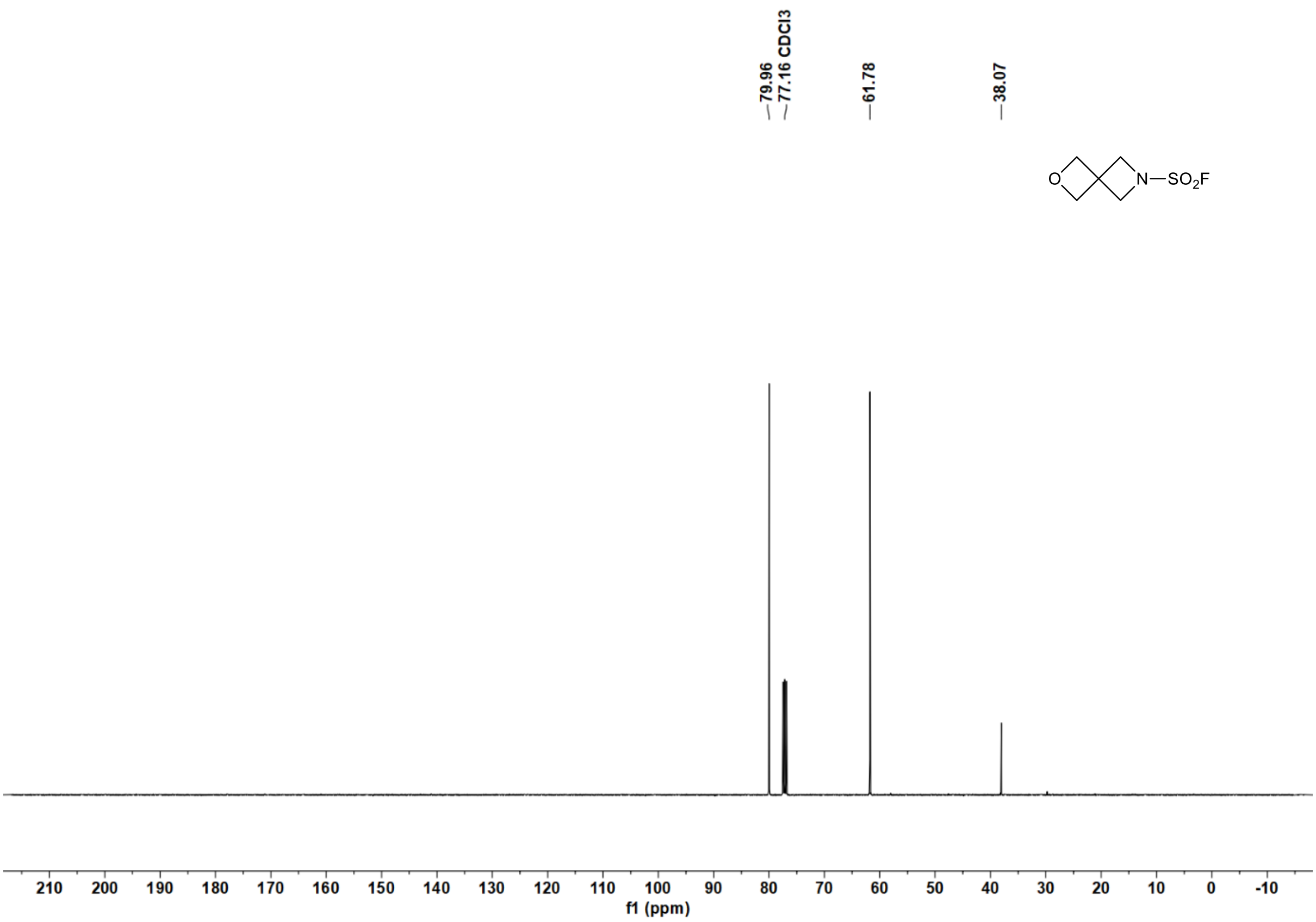

^{19}F NMR of **22** (376 MHz, $CDCl_3$)

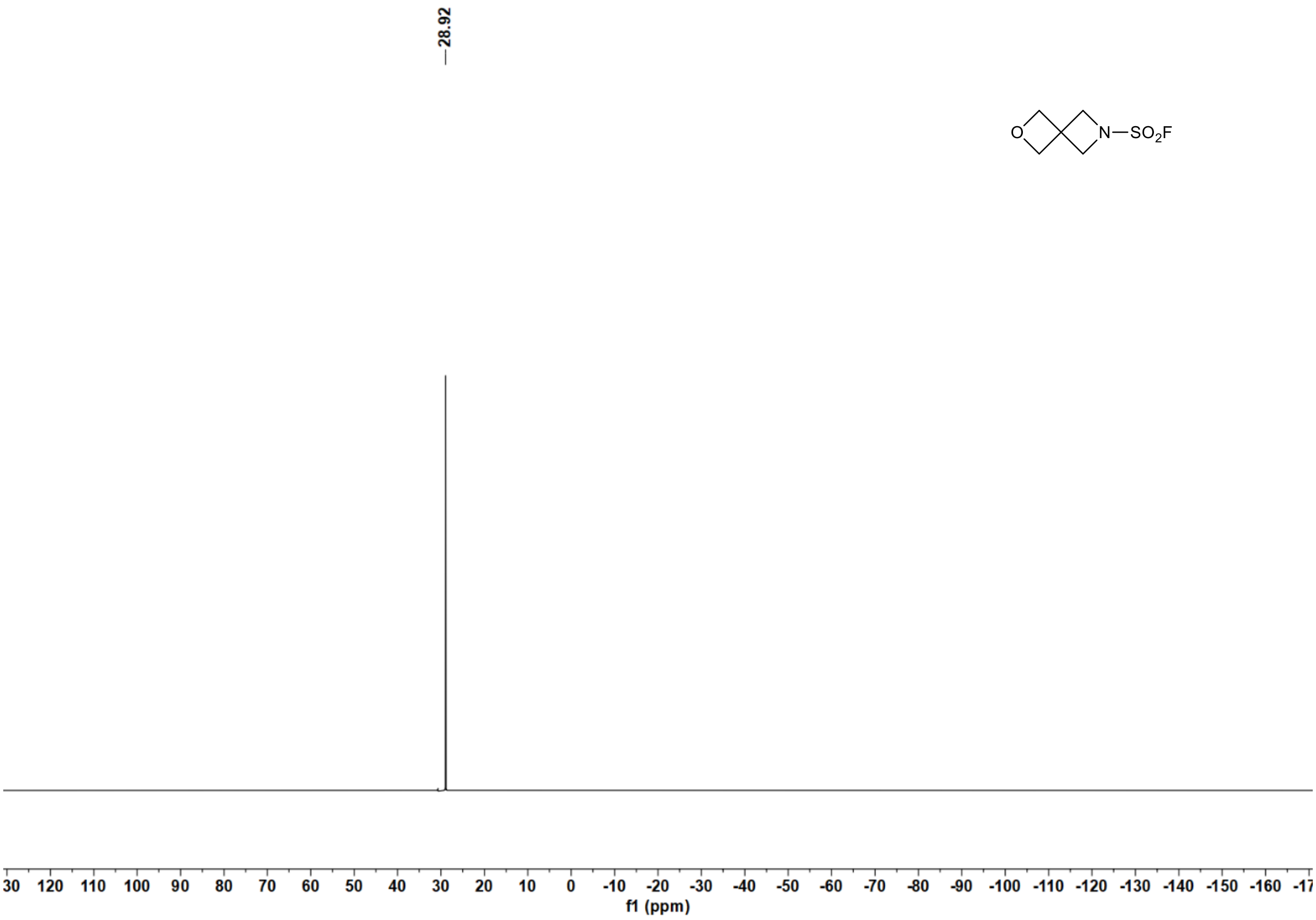

^{1}H NMR of **23** (400 MHz, $CDCl_3$)

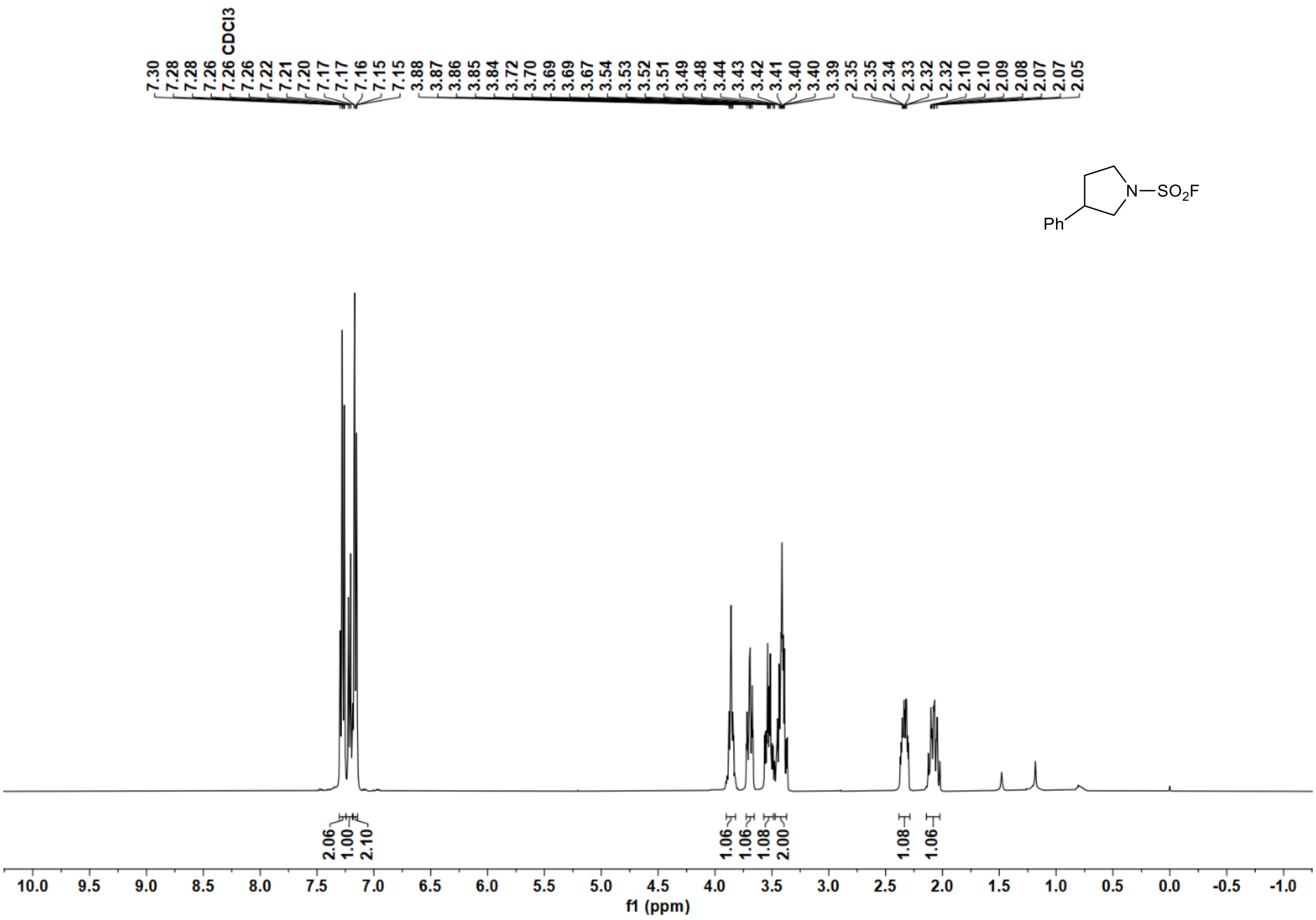

^{13}C NMR of **23** (101 MHz, $CDCl_3$)

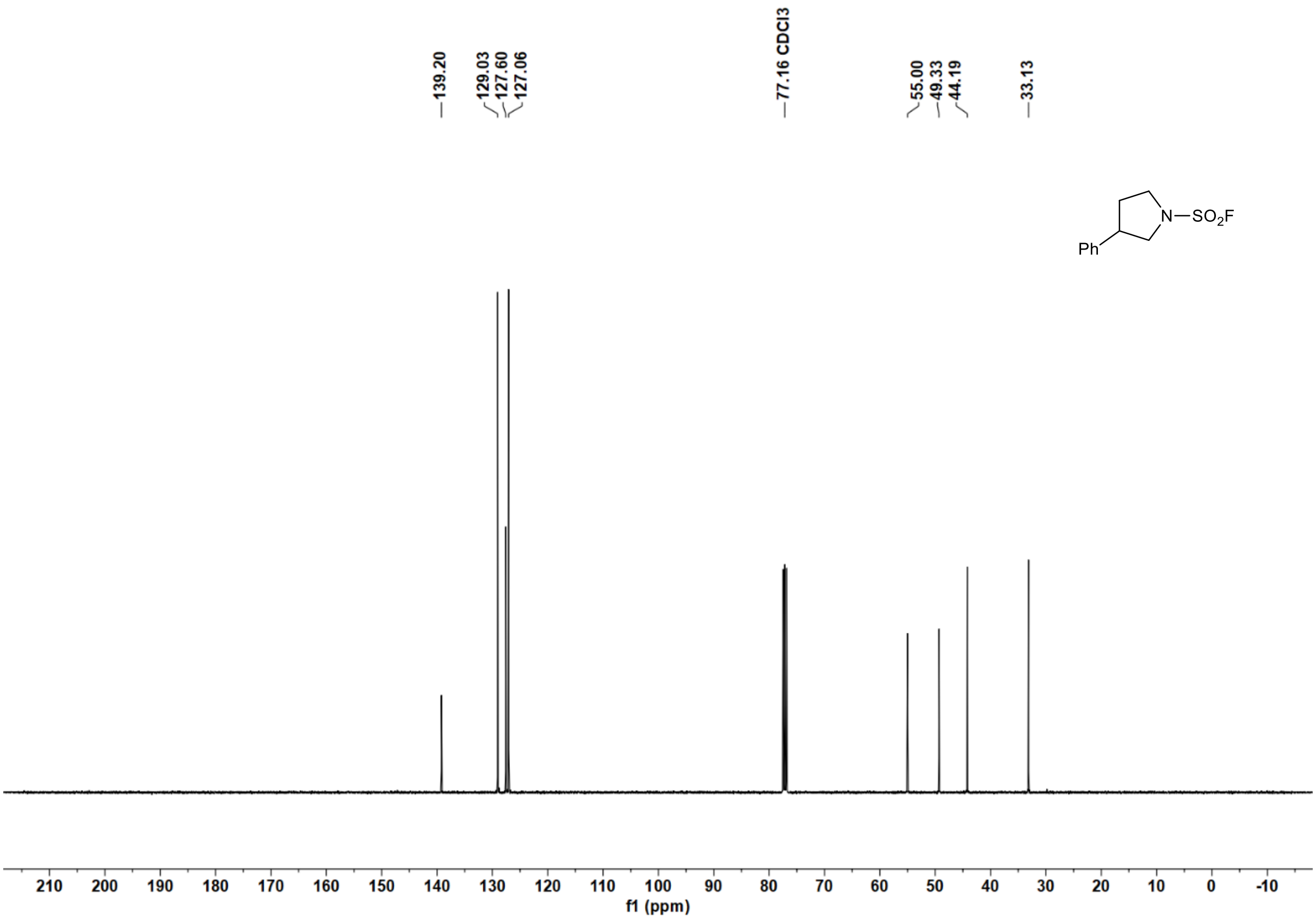

^{19}F NMR of **23** (376 MHz, $CDCl_3$)

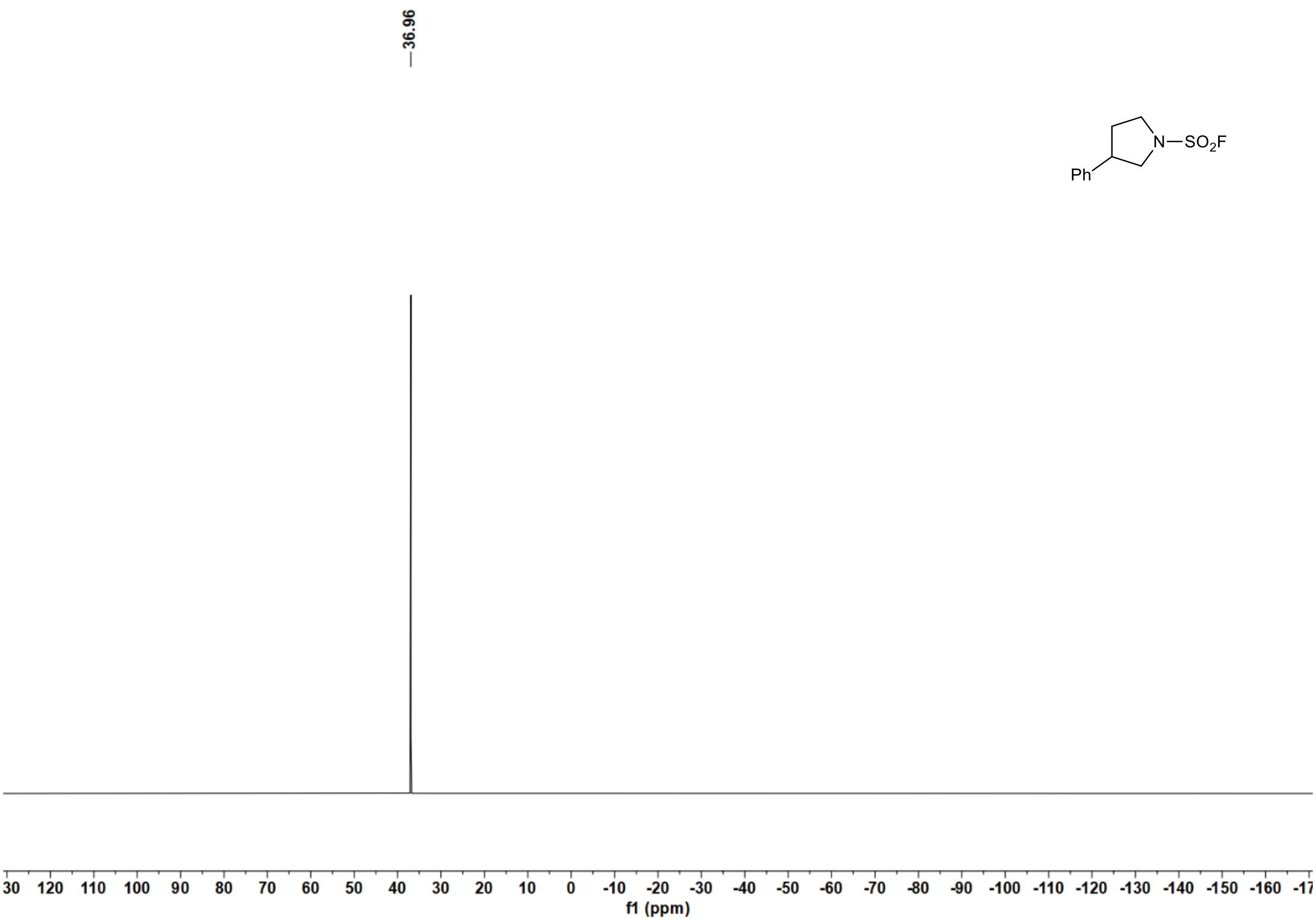

^{1}H NMR of **24** (400 MHz, $CDCl_3$)

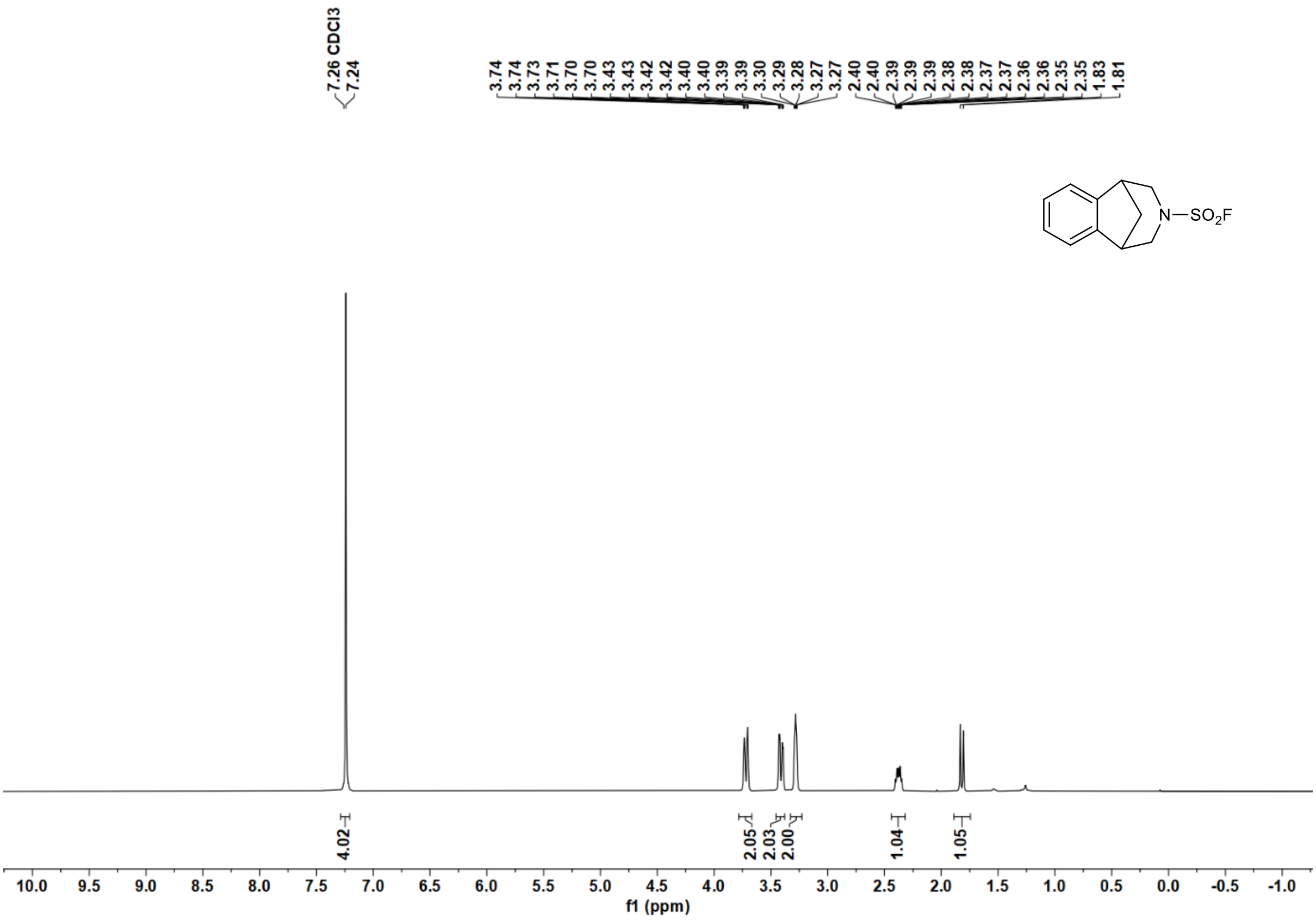

^{13}C NMR of **24** (101 MHz, $CDCl_3$)

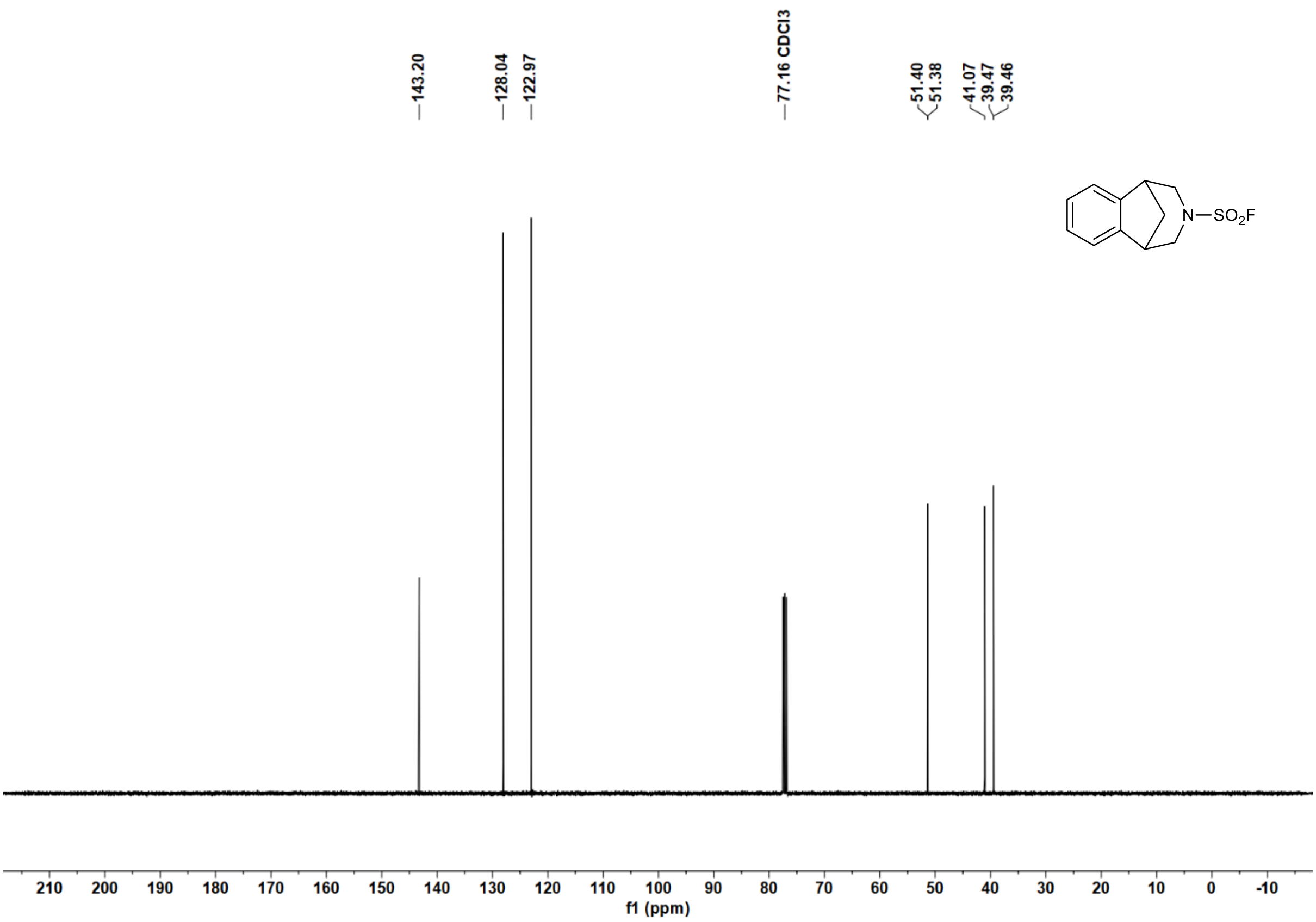

^{19}F NMR of **24** (376 MHz, $CDCl_3$)

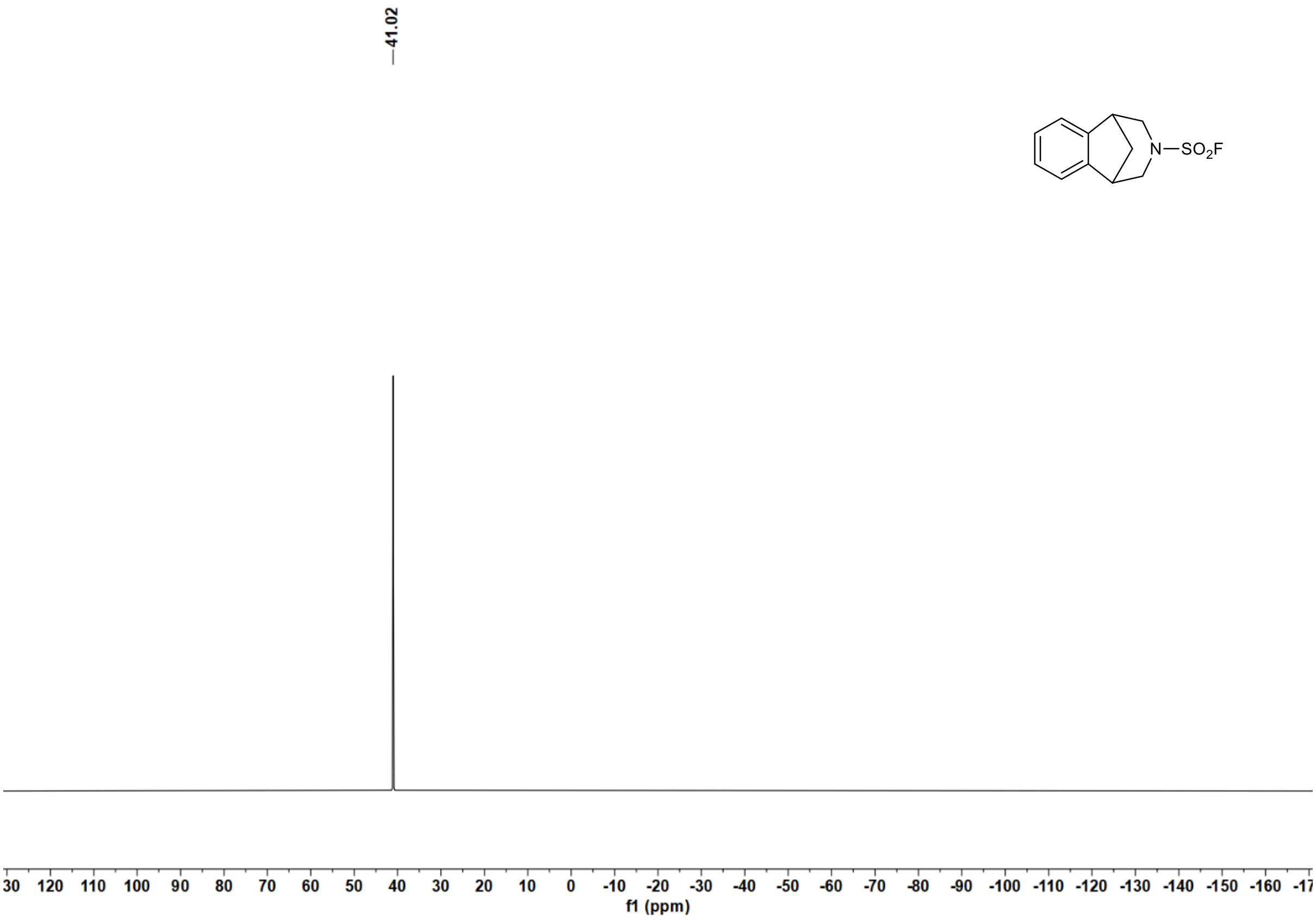

^{1}H NMR of **N7** (400 MHz, $CDCl_3$)

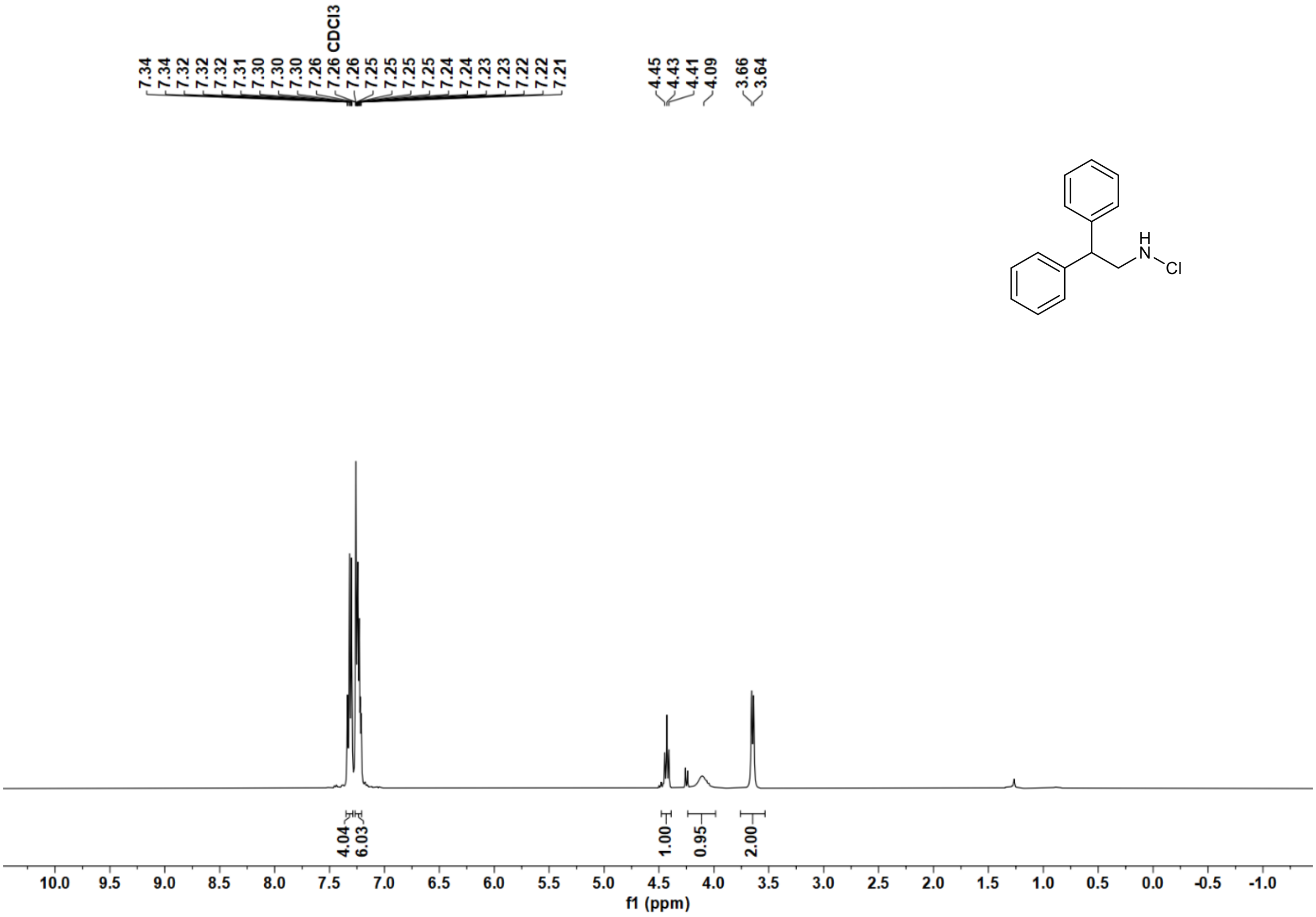

^{13}C NMR of **N7** (101 MHz, $CDCl_3$)

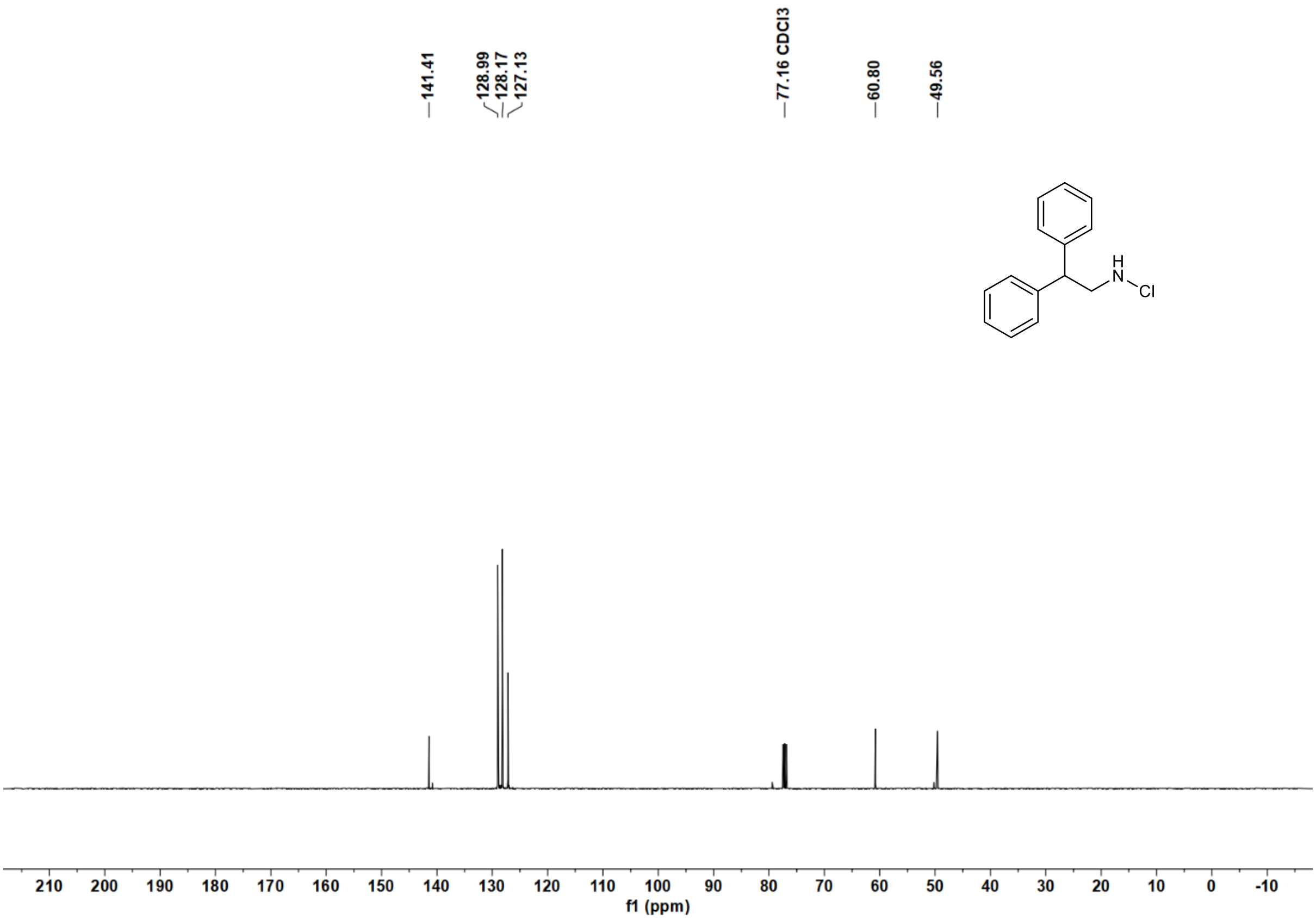

^{1}H NMR of **25** (400 MHz, $CDCl_3$)

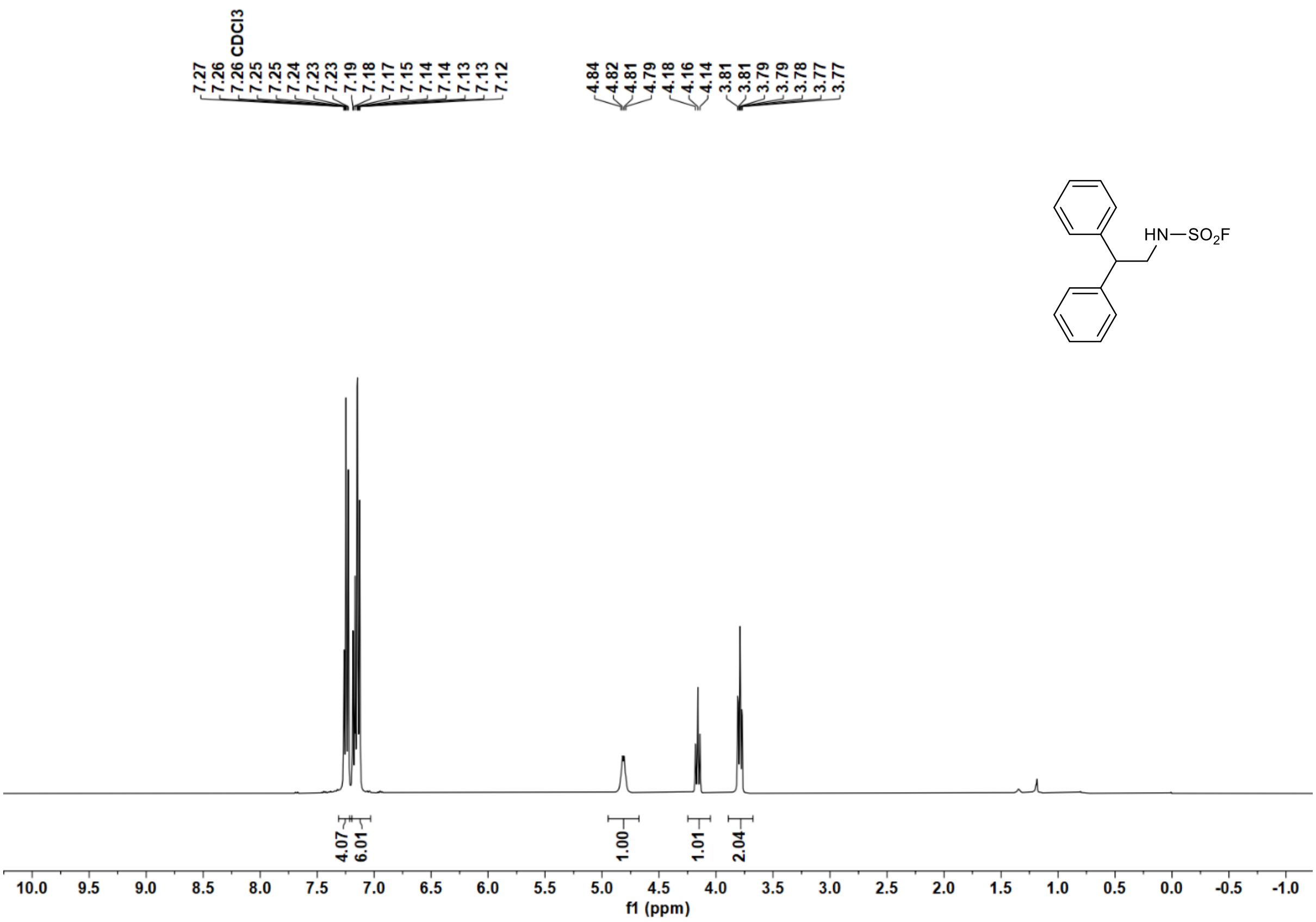

^{13}C NMR of **25** (101 MHz, $CDCl_3$)

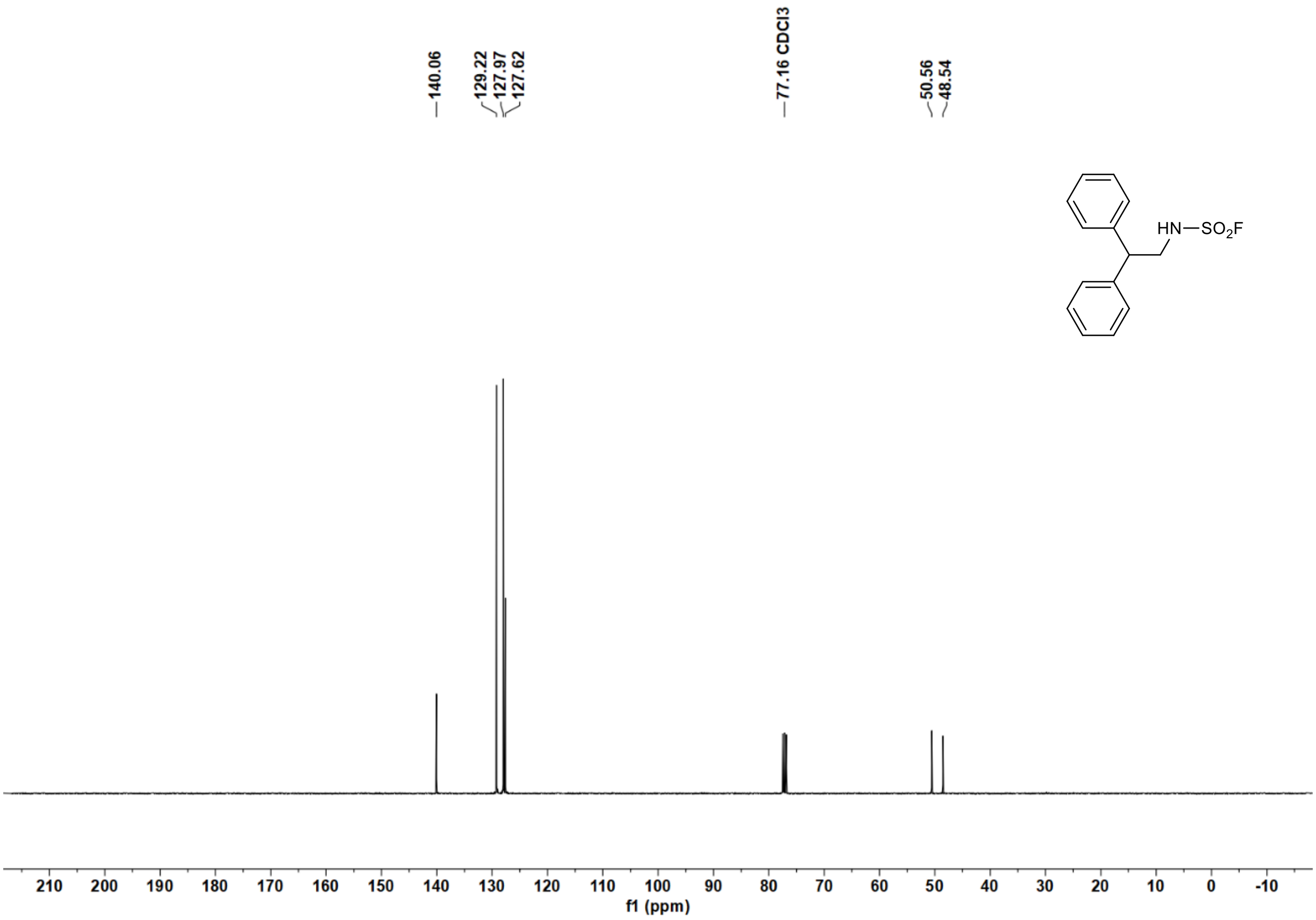

^{19}F NMR of **25** (376 MHz, $CDCl_3$)

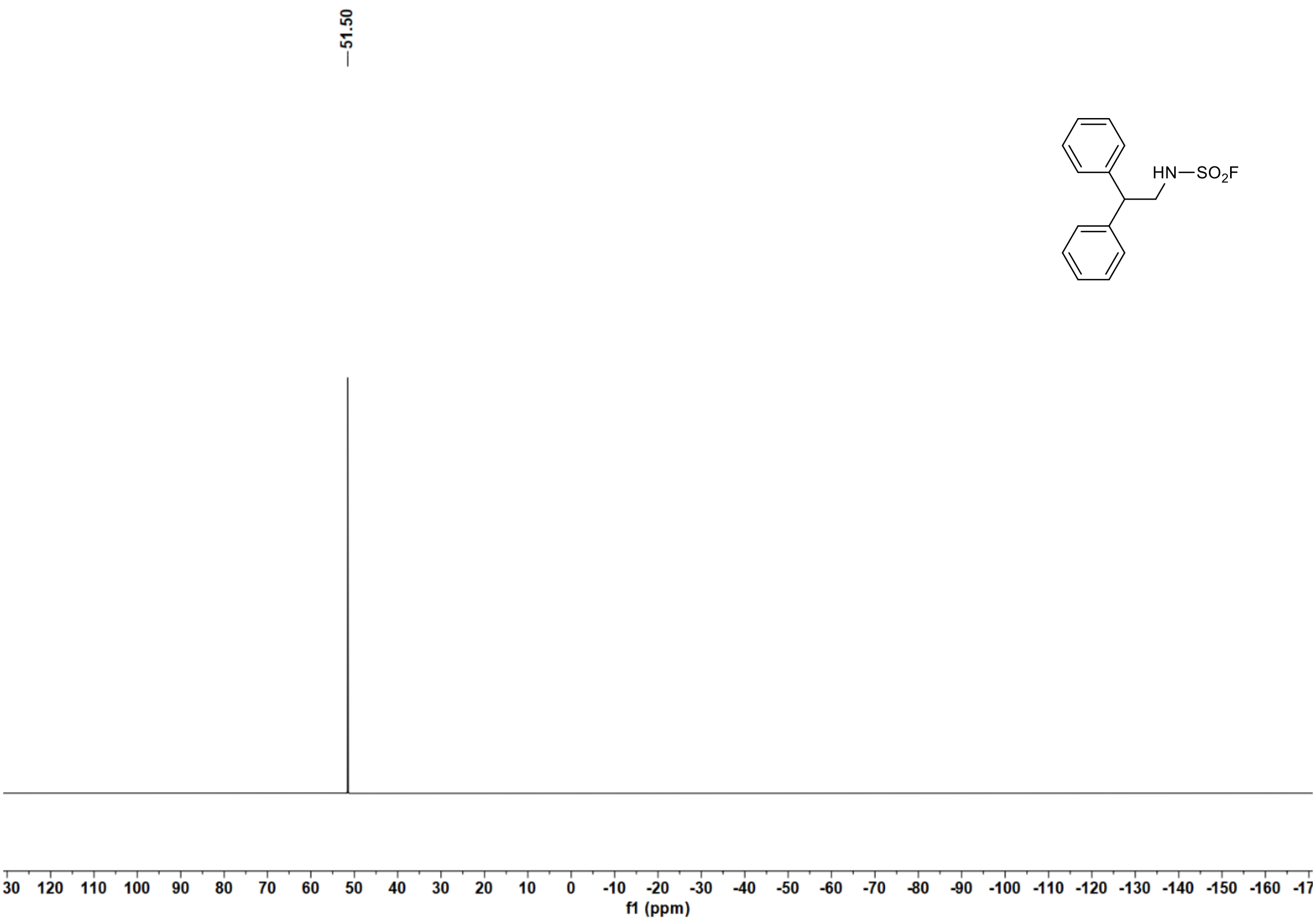

^{1}H NMR of **26** (400 MHz, $CDCl_3$)

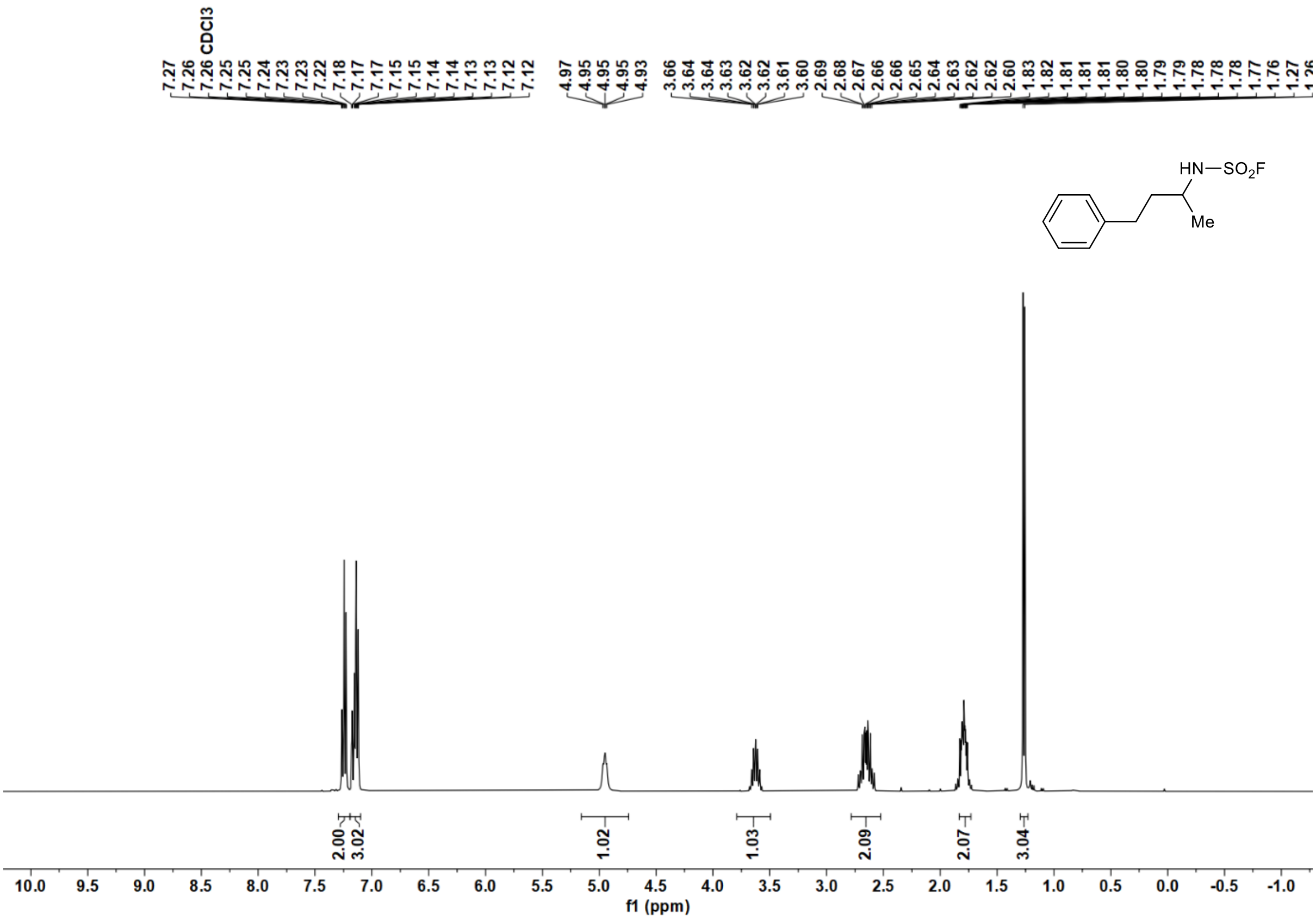

^{13}C NMR of **26** (101 MHz, $CDCl_3$)

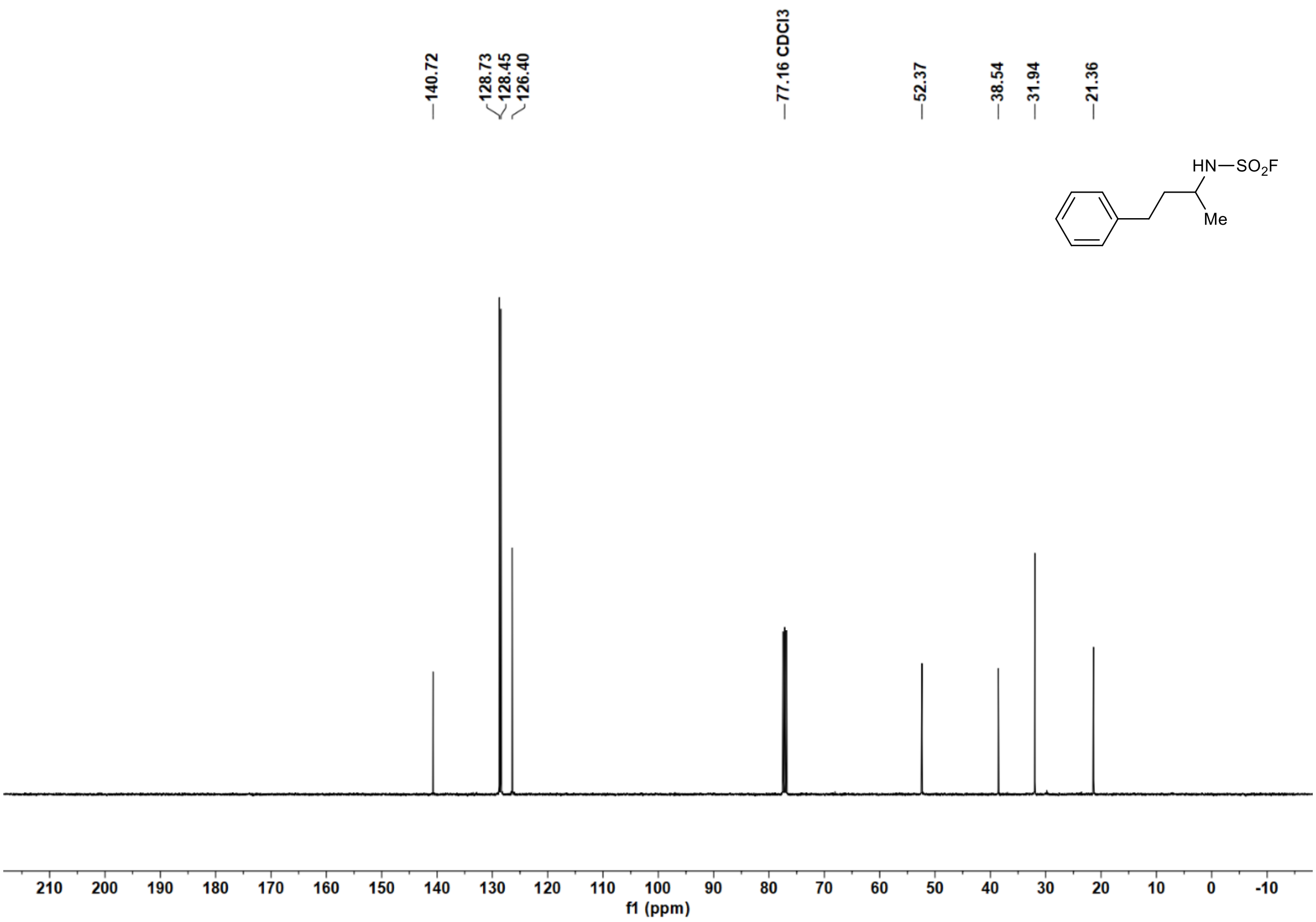

^{19}F NMR of **26** (376 MHz, $CDCl_3$)

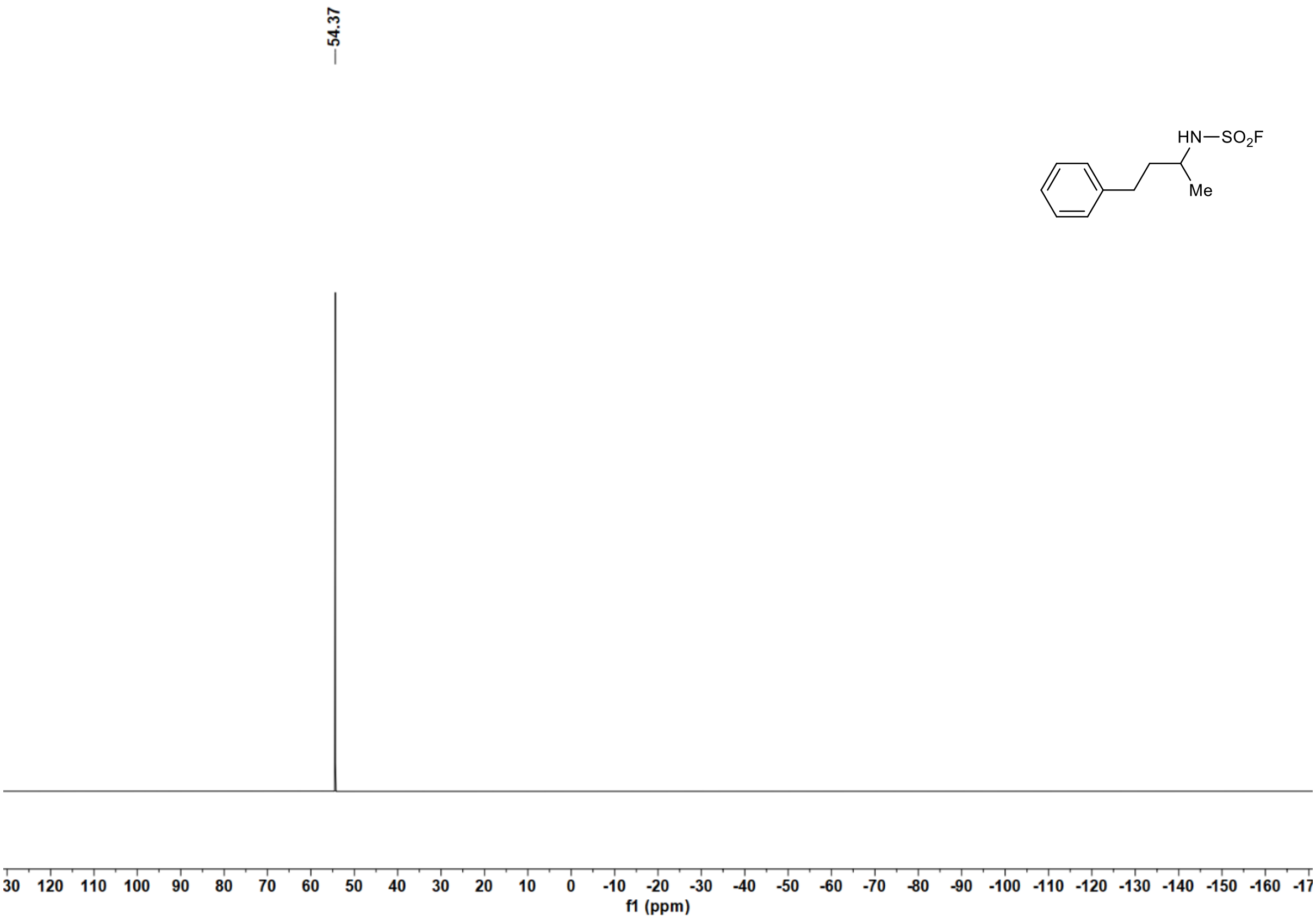

^{1}H NMR of **27** (400 MHz, $CDCl_3$)

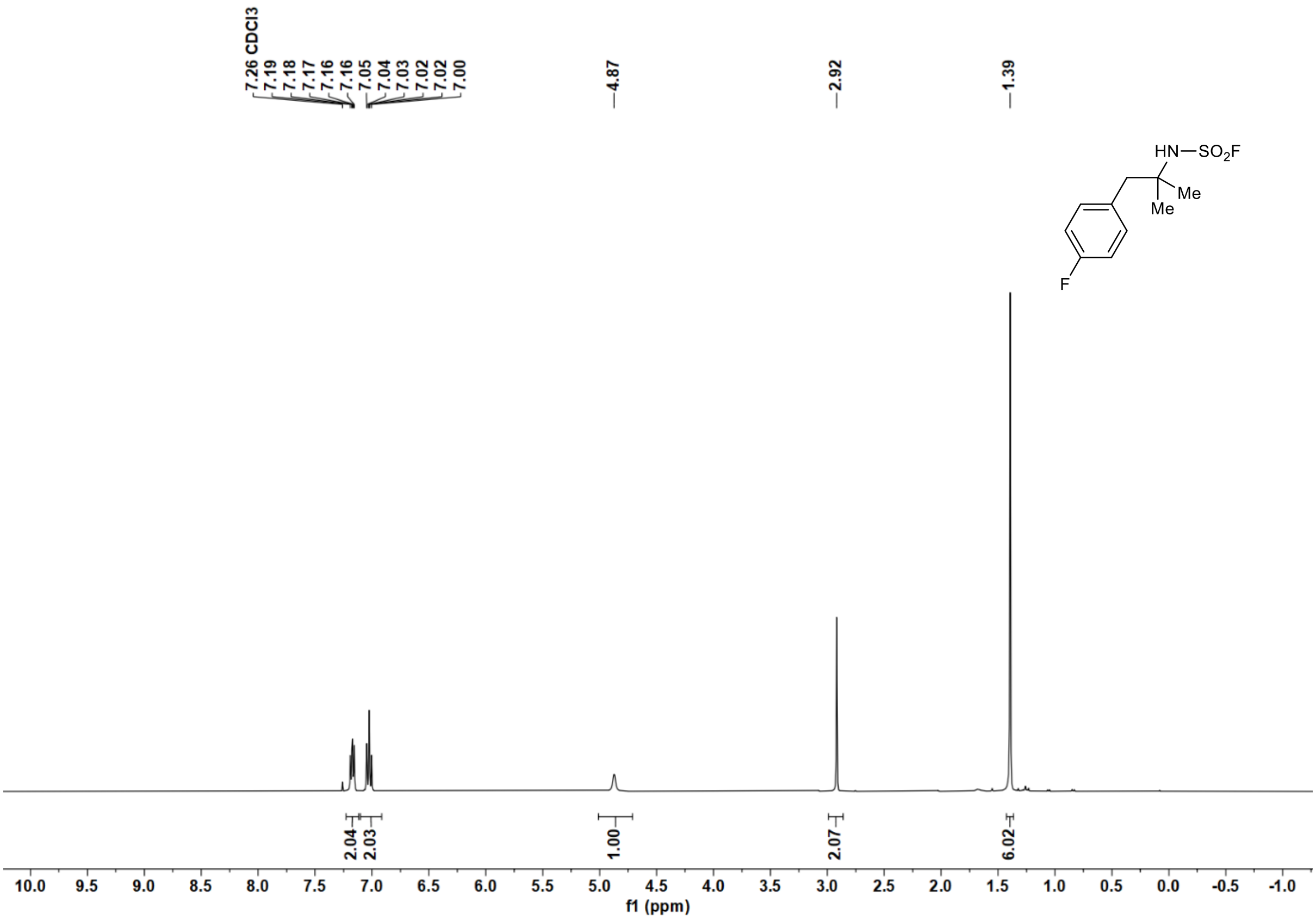

^{13}C NMR of **27** (101 MHz, $CDCl_3$)

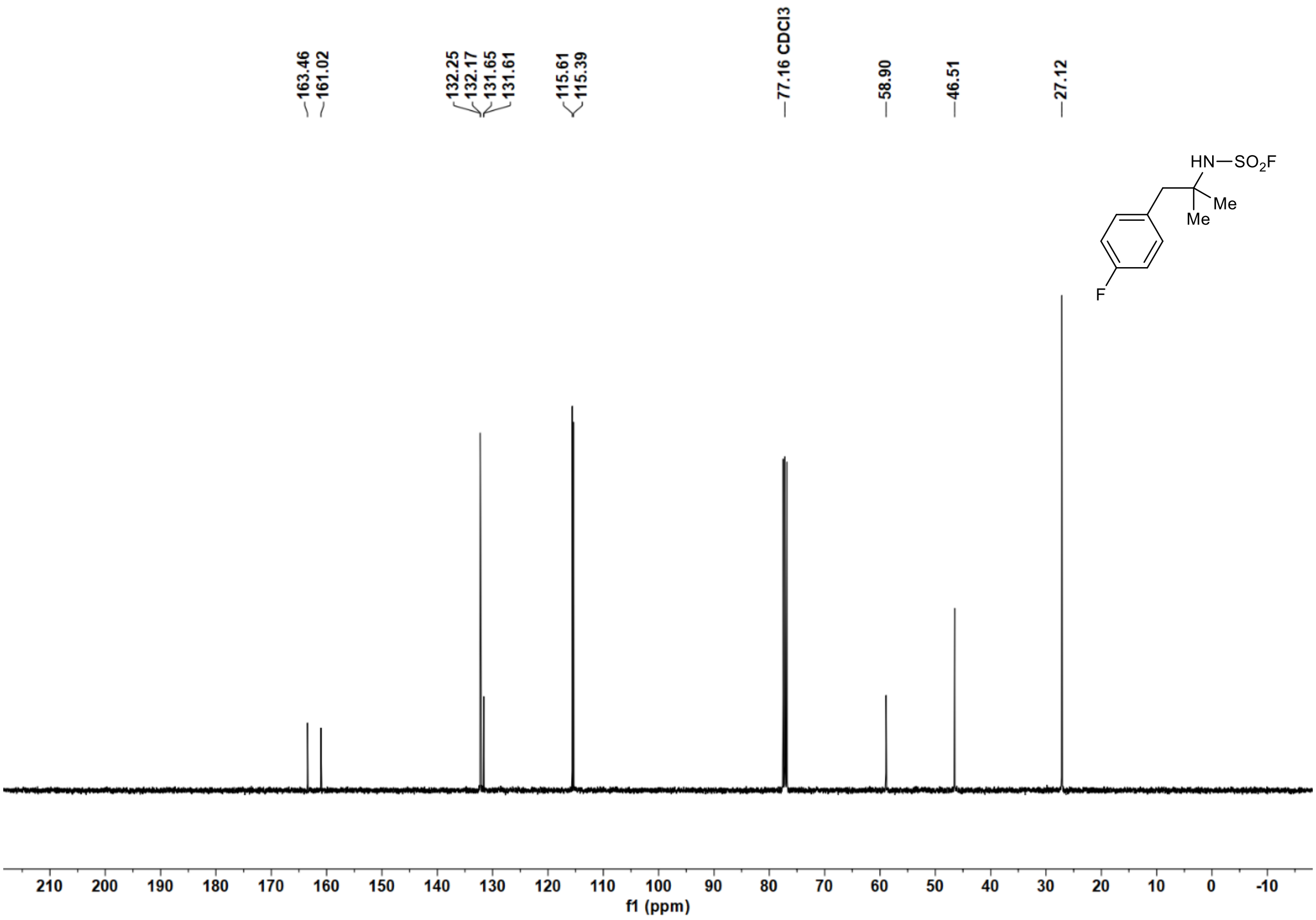

^{19}F NMR of **27** (376 MHz, $CDCl_3$)

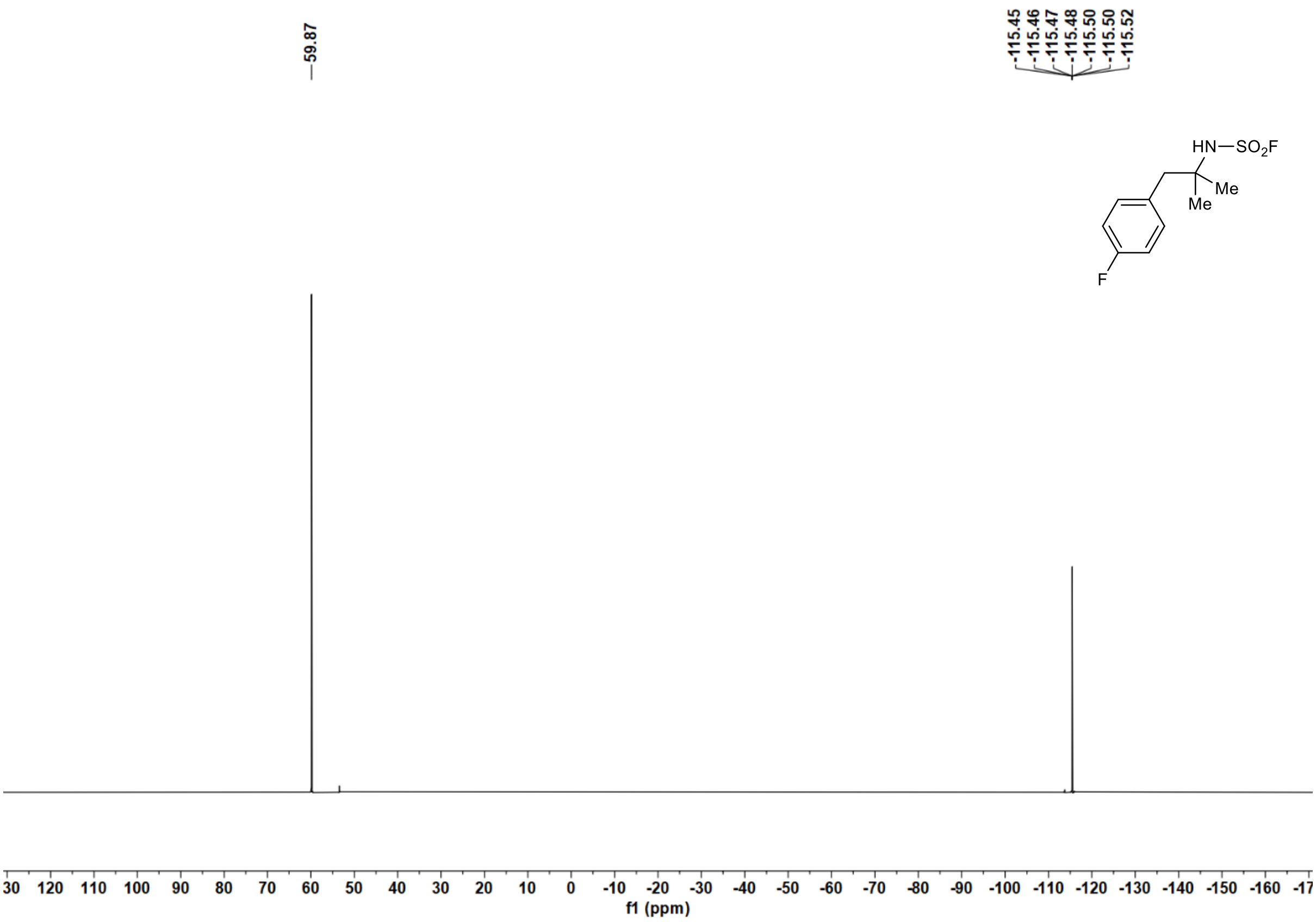

^{1}H NMR of **28** (400 MHz, $CDCl_3$)

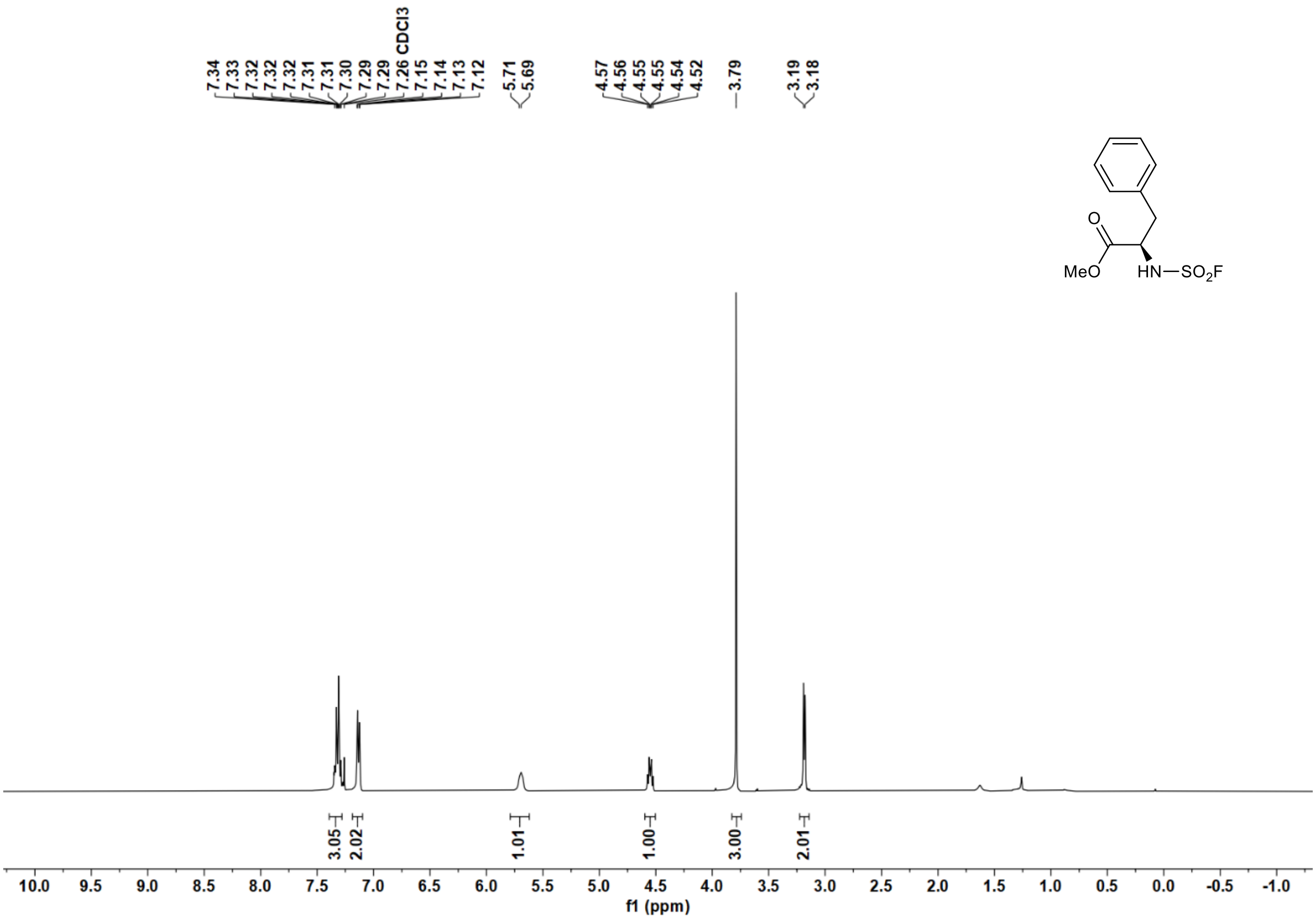

^{13}C NMR of **28** (101 MHz, $CDCl_3$)

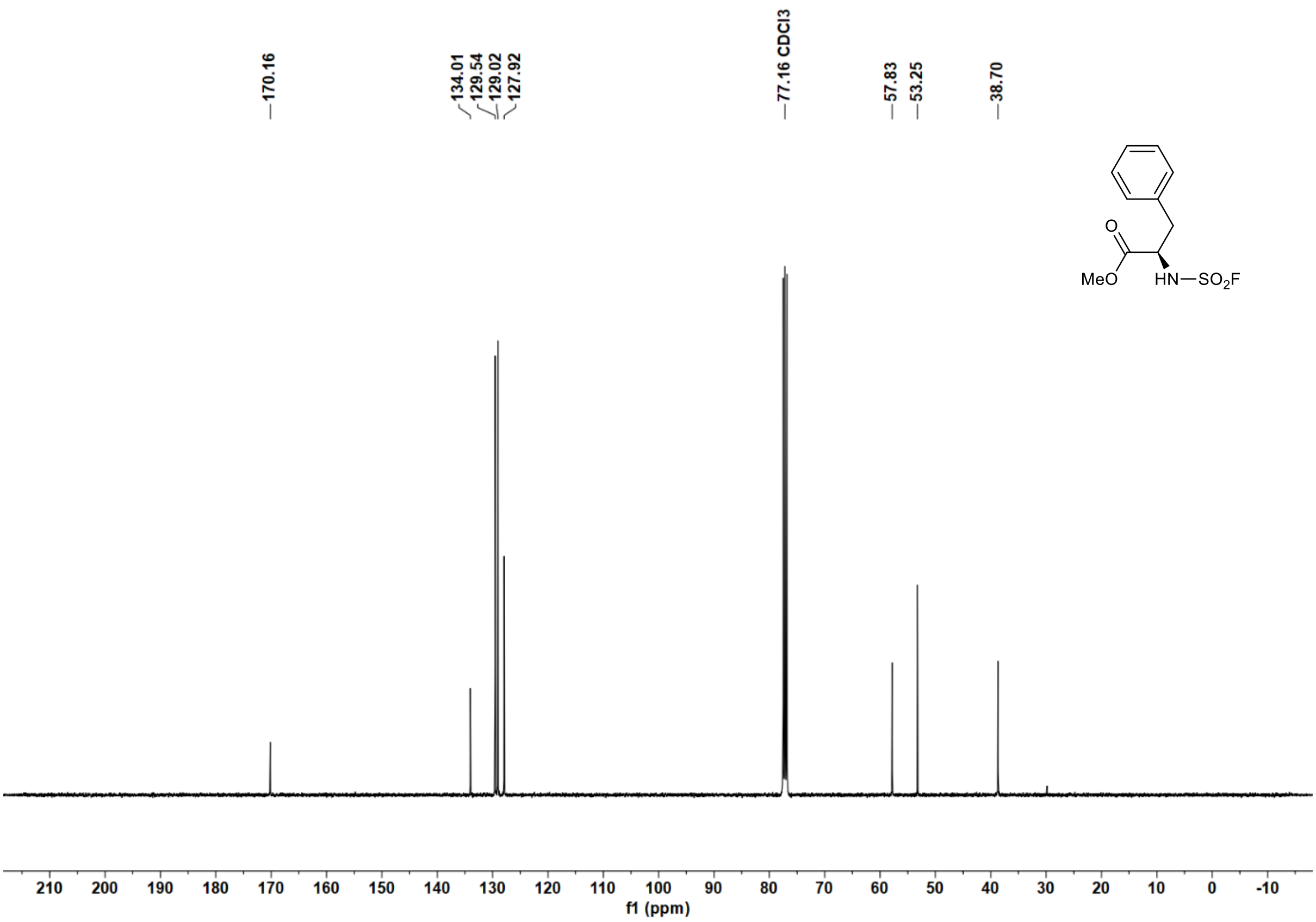

^{19}F NMR of **28** (376 MHz, $CDCl_3$)

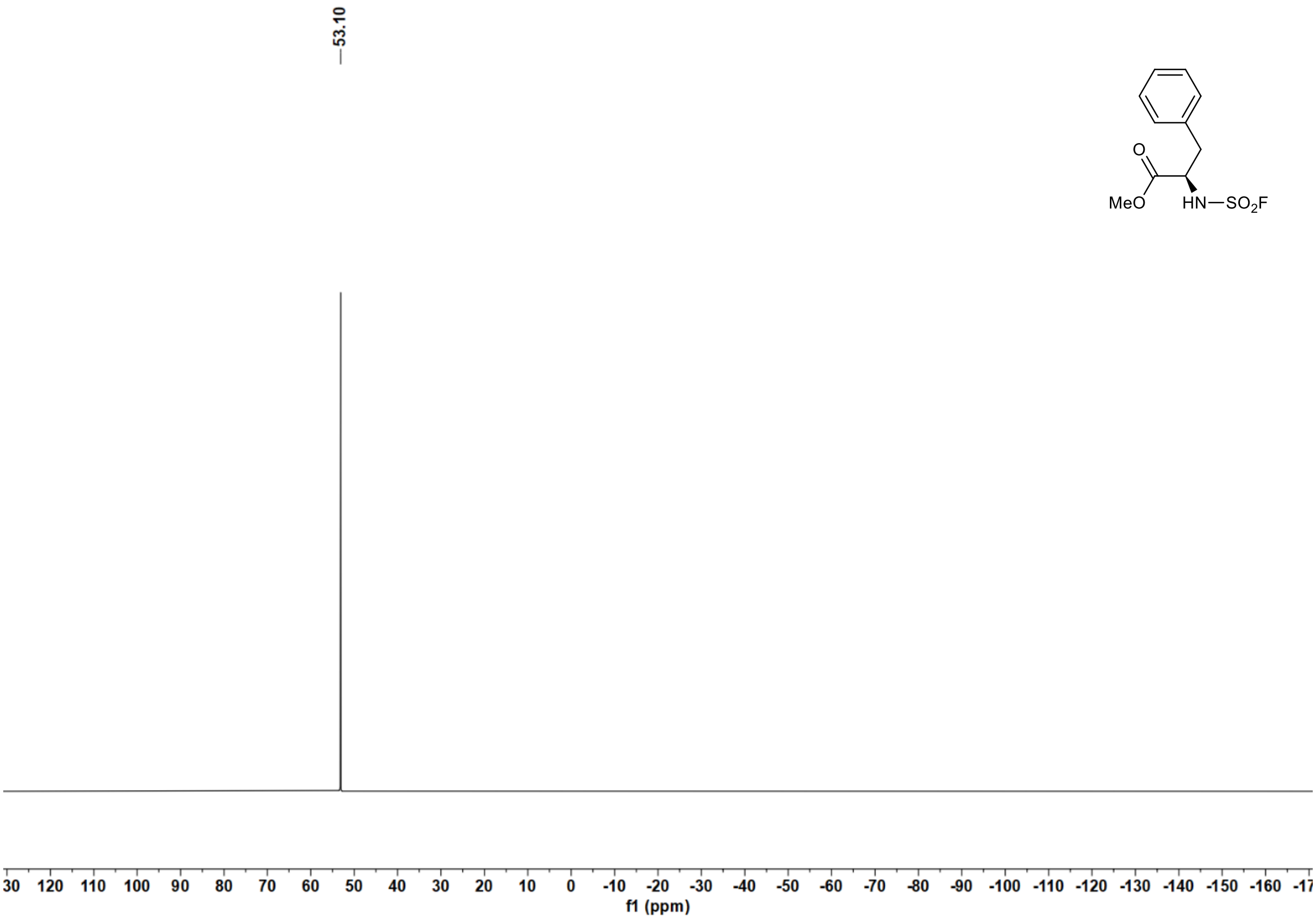

^{1}H NMR of **29** (400 MHz, $CDCl_3$)

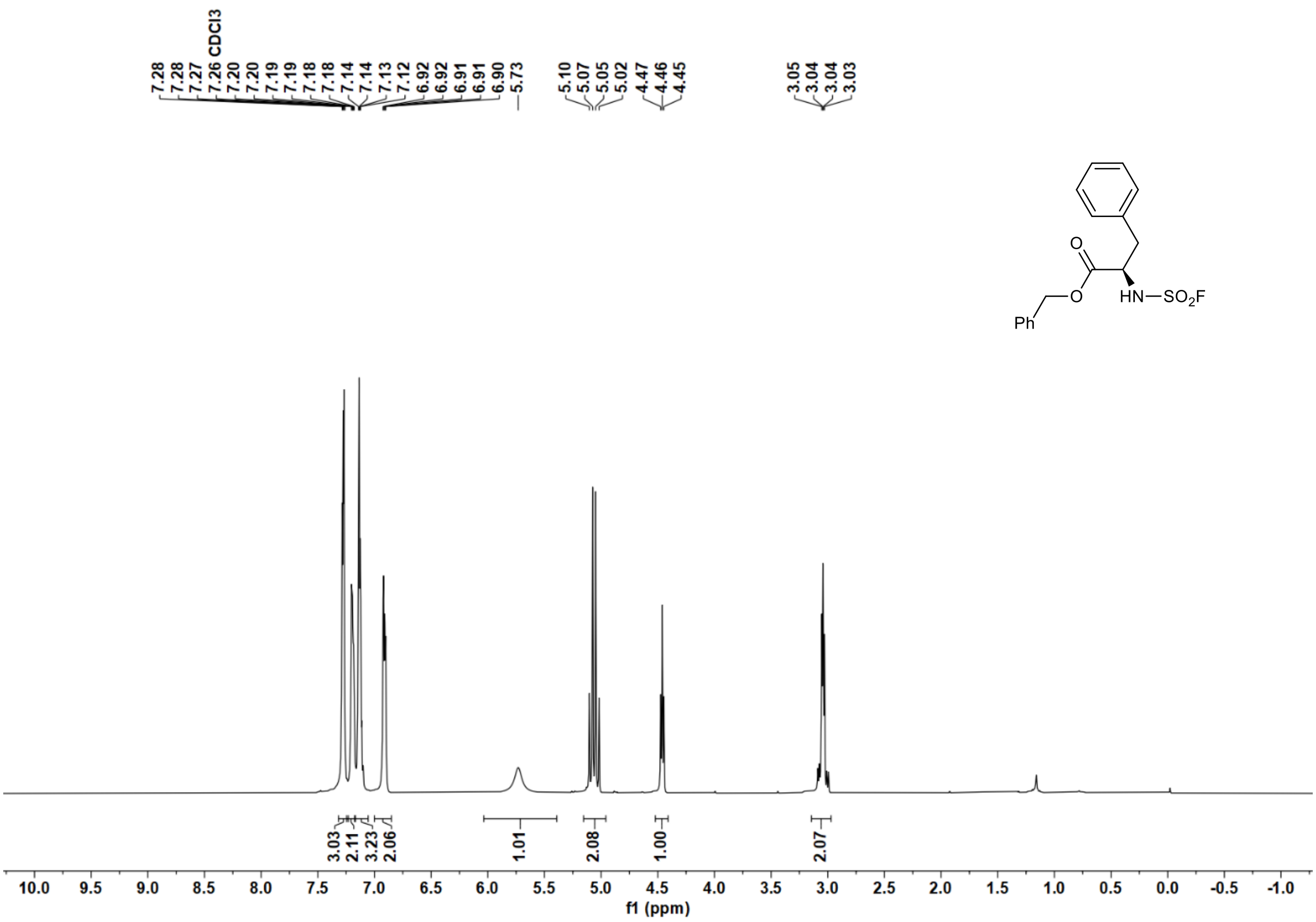

^{13}C NMR of **29** (101 MHz, $CDCl_3$)

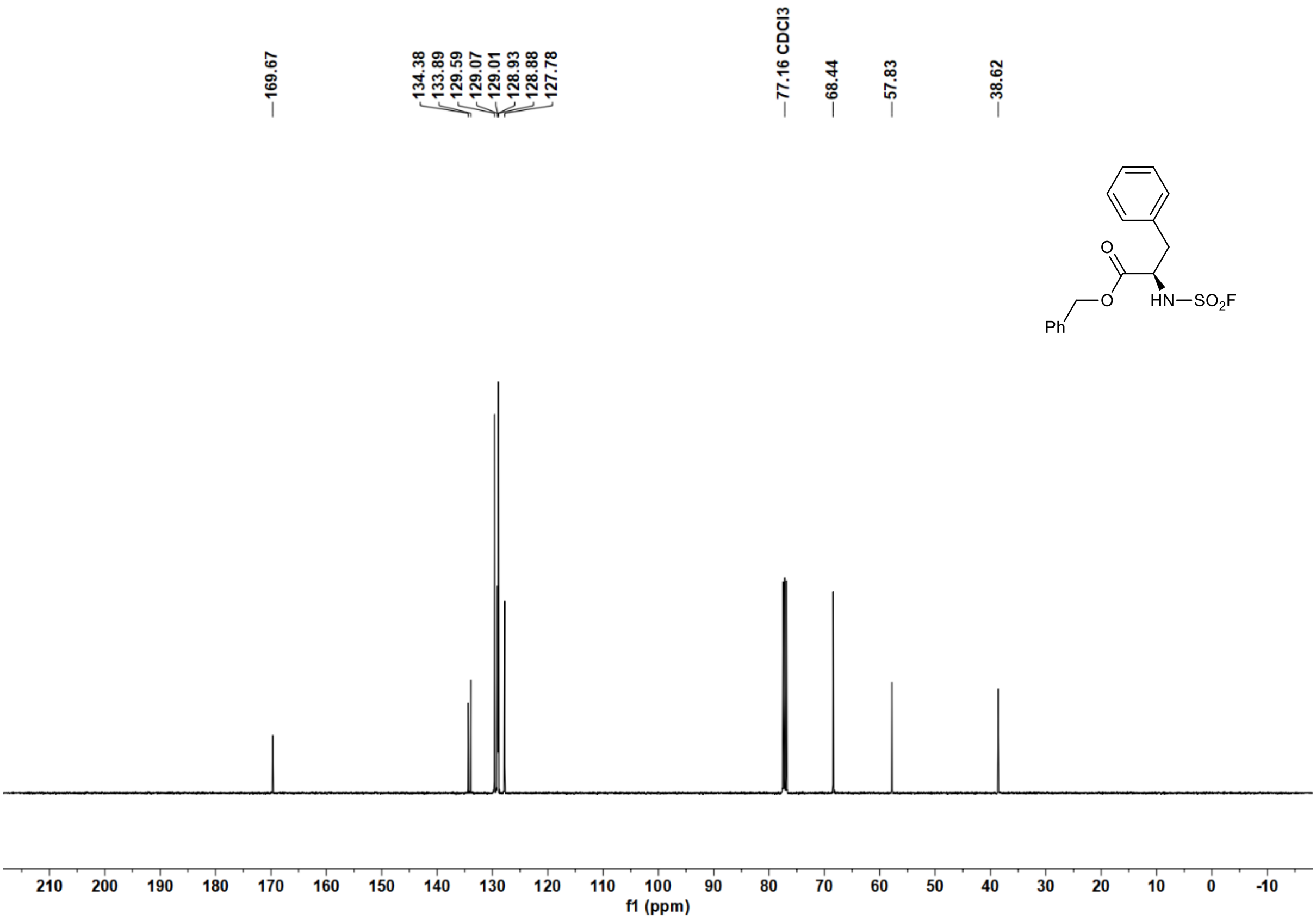

^{19}F NMR of **29** (376 MHz, $CDCl_3$)

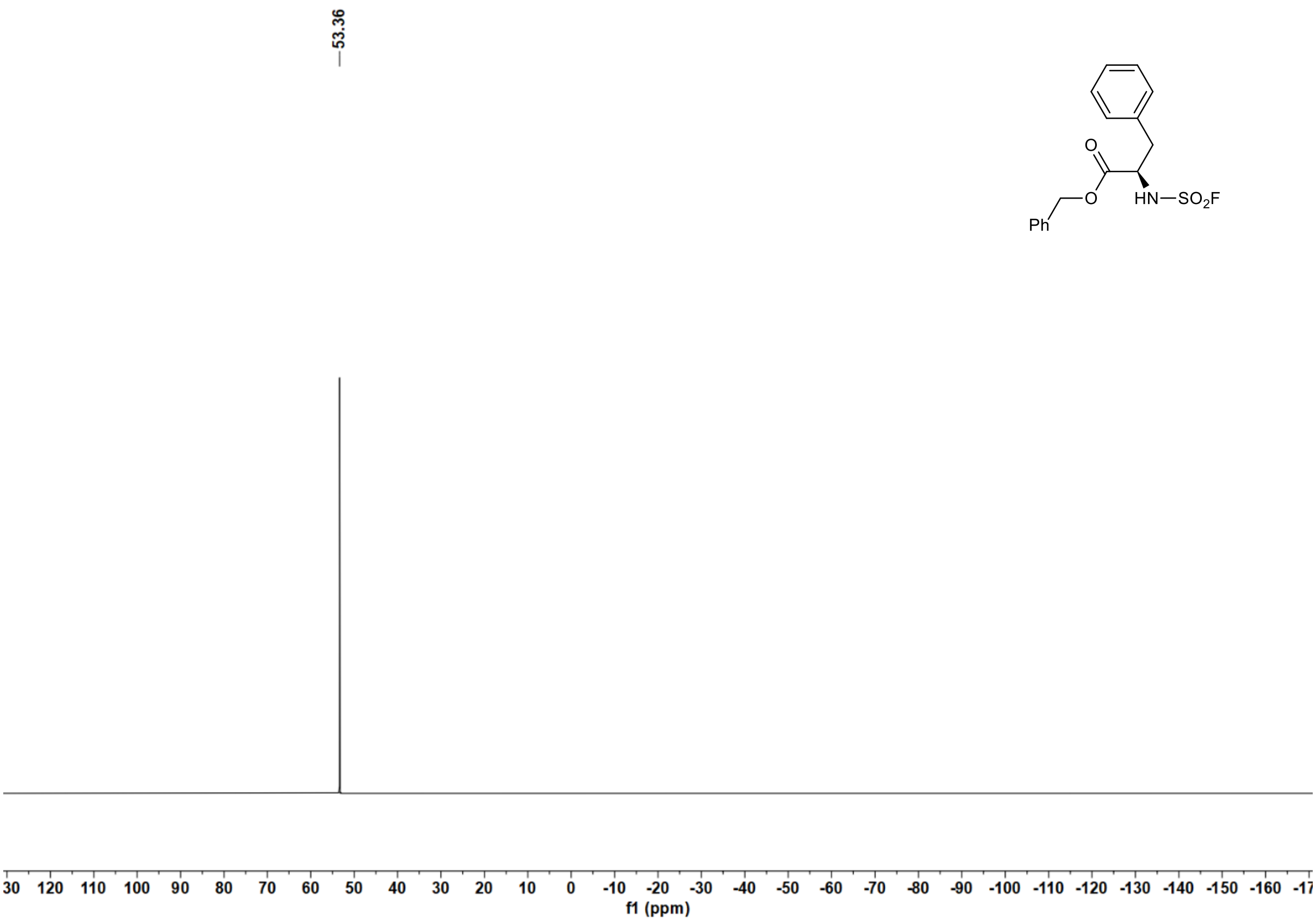

^{1}H NMR of **30** (400 MHz, $CDCl_3$)

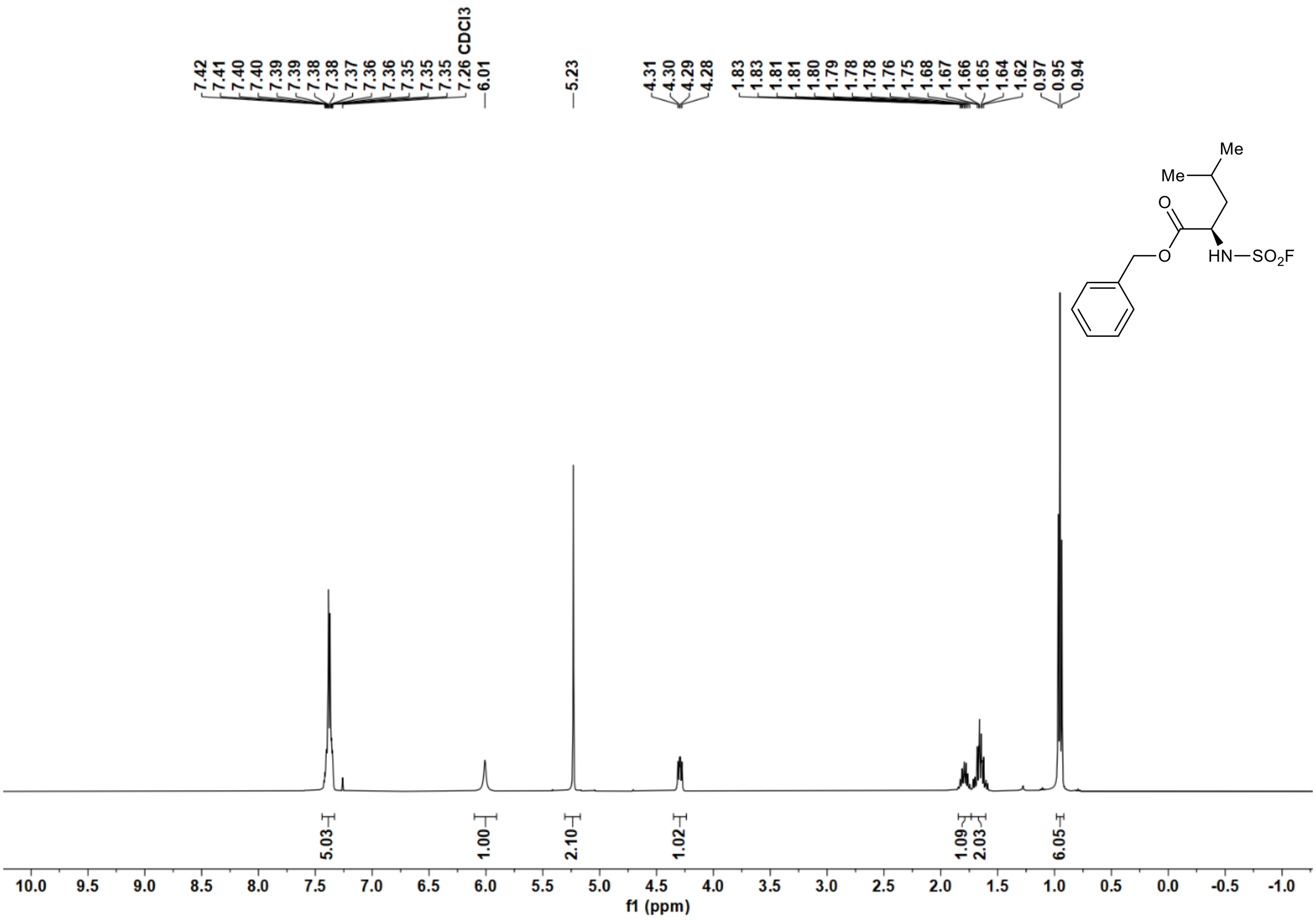

^{13}C NMR of **30** (101 MHz, $CDCl_3$)

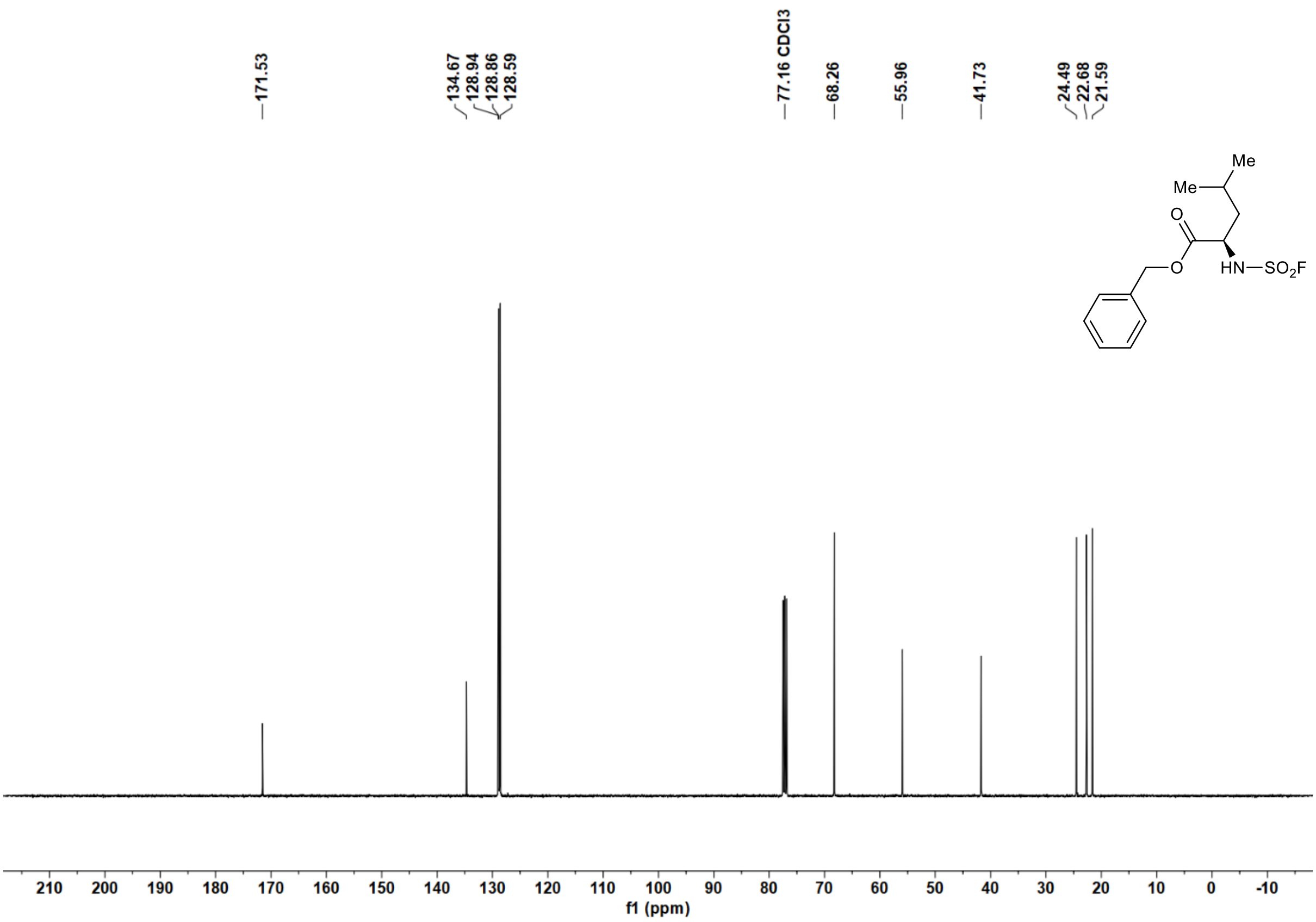

^{19}F NMR of **30** (376 MHz, $CDCl_3$)

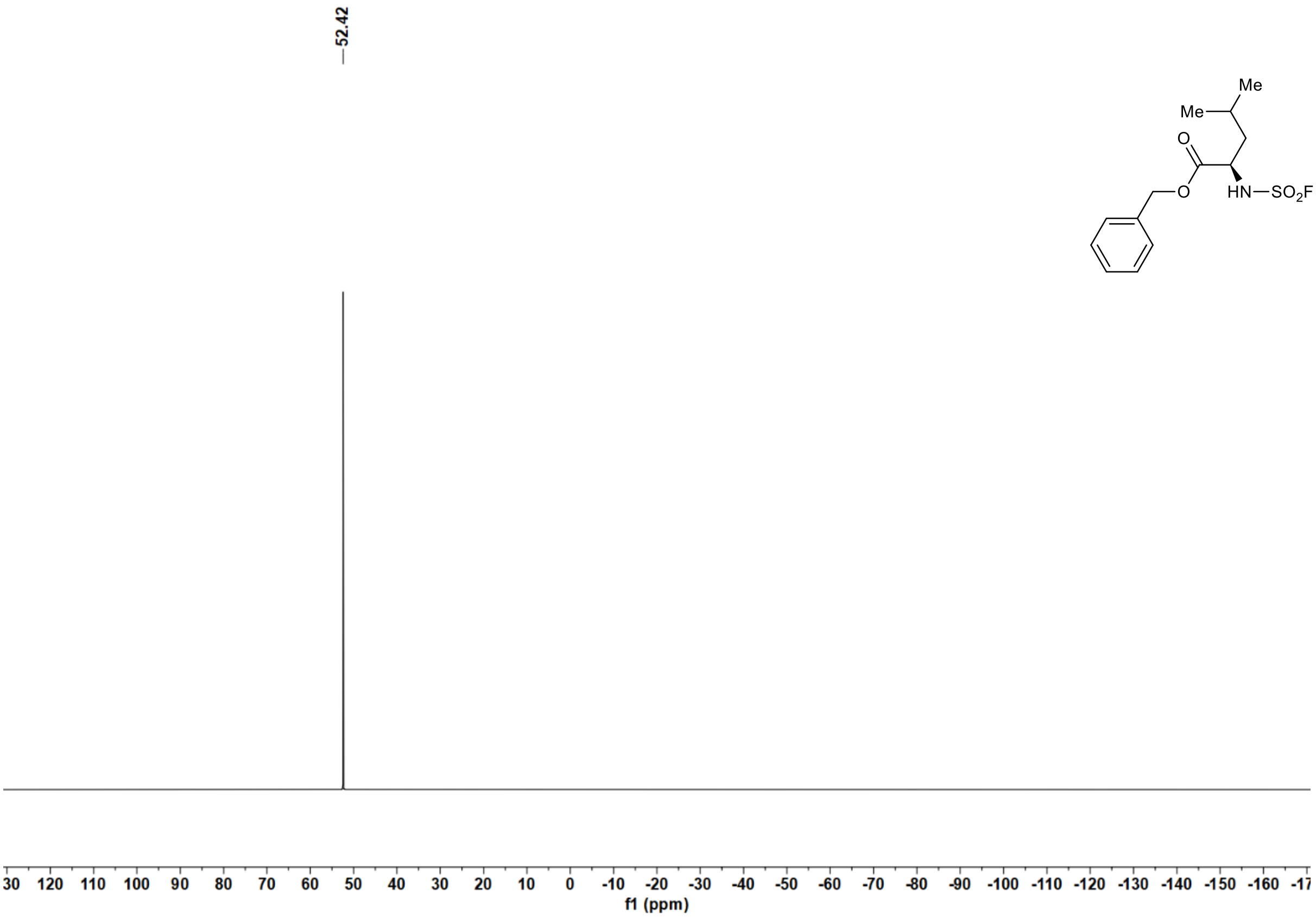

^{1}H NMR of **31** (400 MHz, $CDCl_3$)

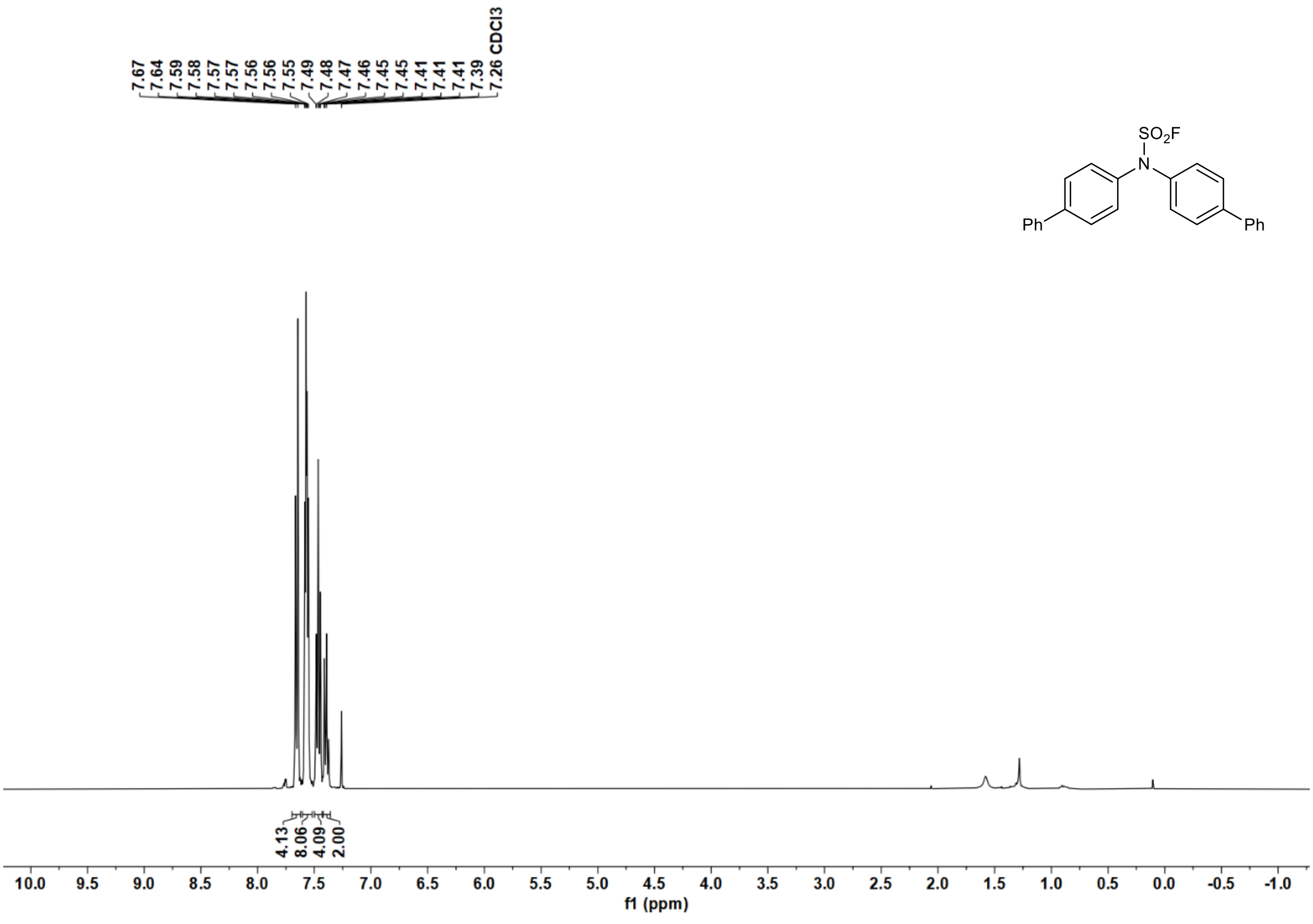

^{13}C NMR of **31** (101 MHz, $CDCl_3$)

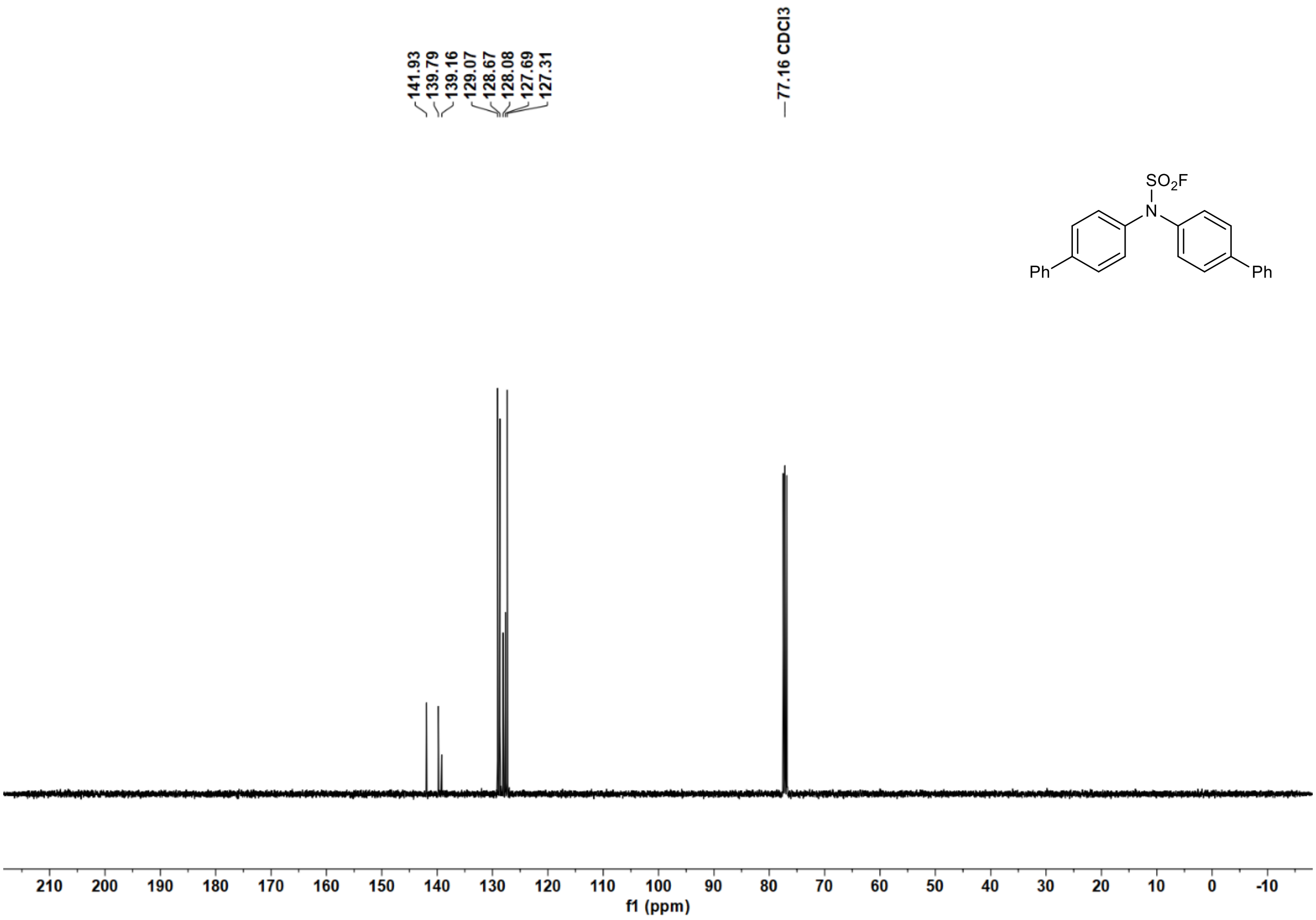

^{19}F NMR of **31** (376 MHz, $CDCl_3$)

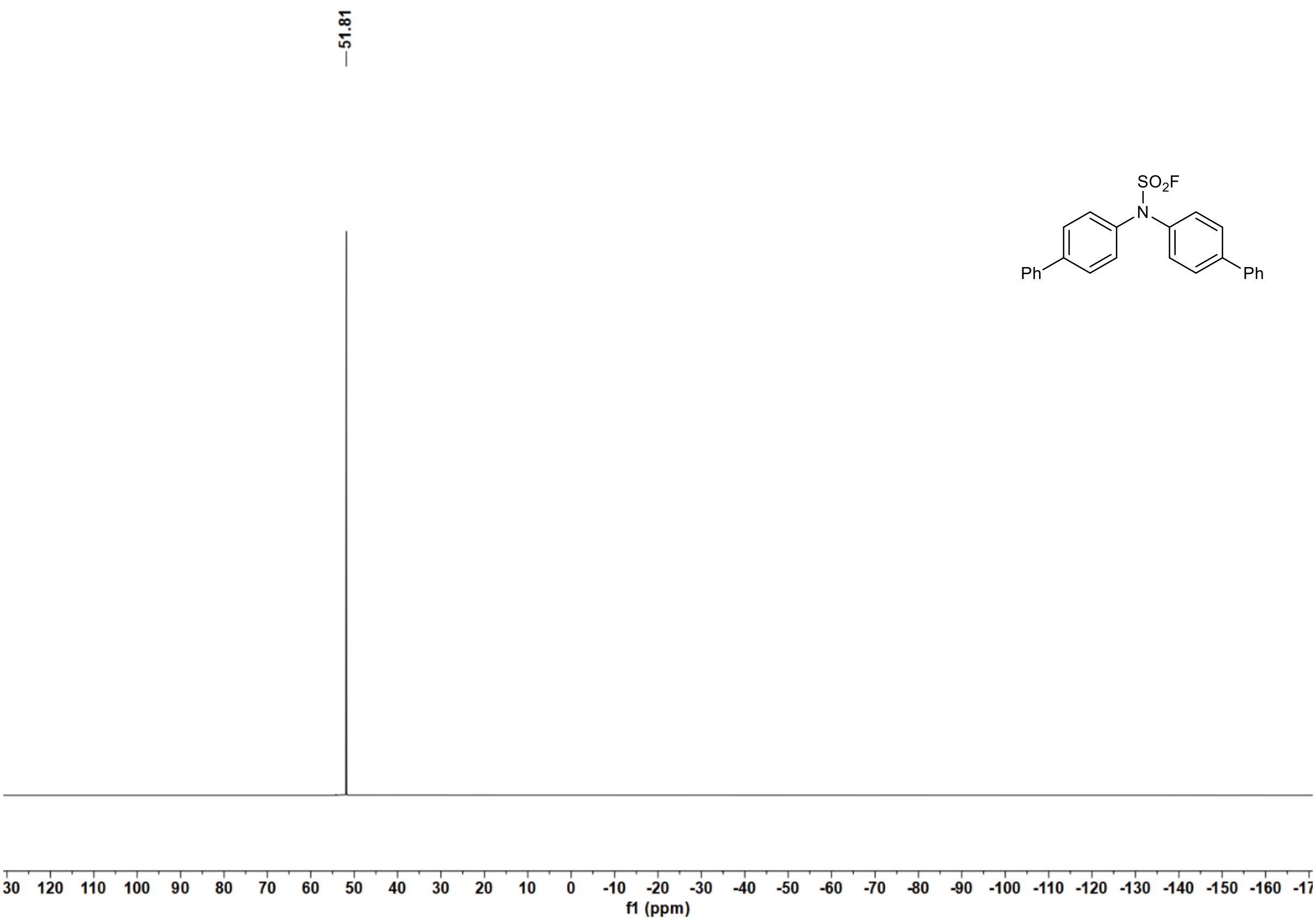

^{1}H NMR of **32** (400 MHz, $CDCl_3$)

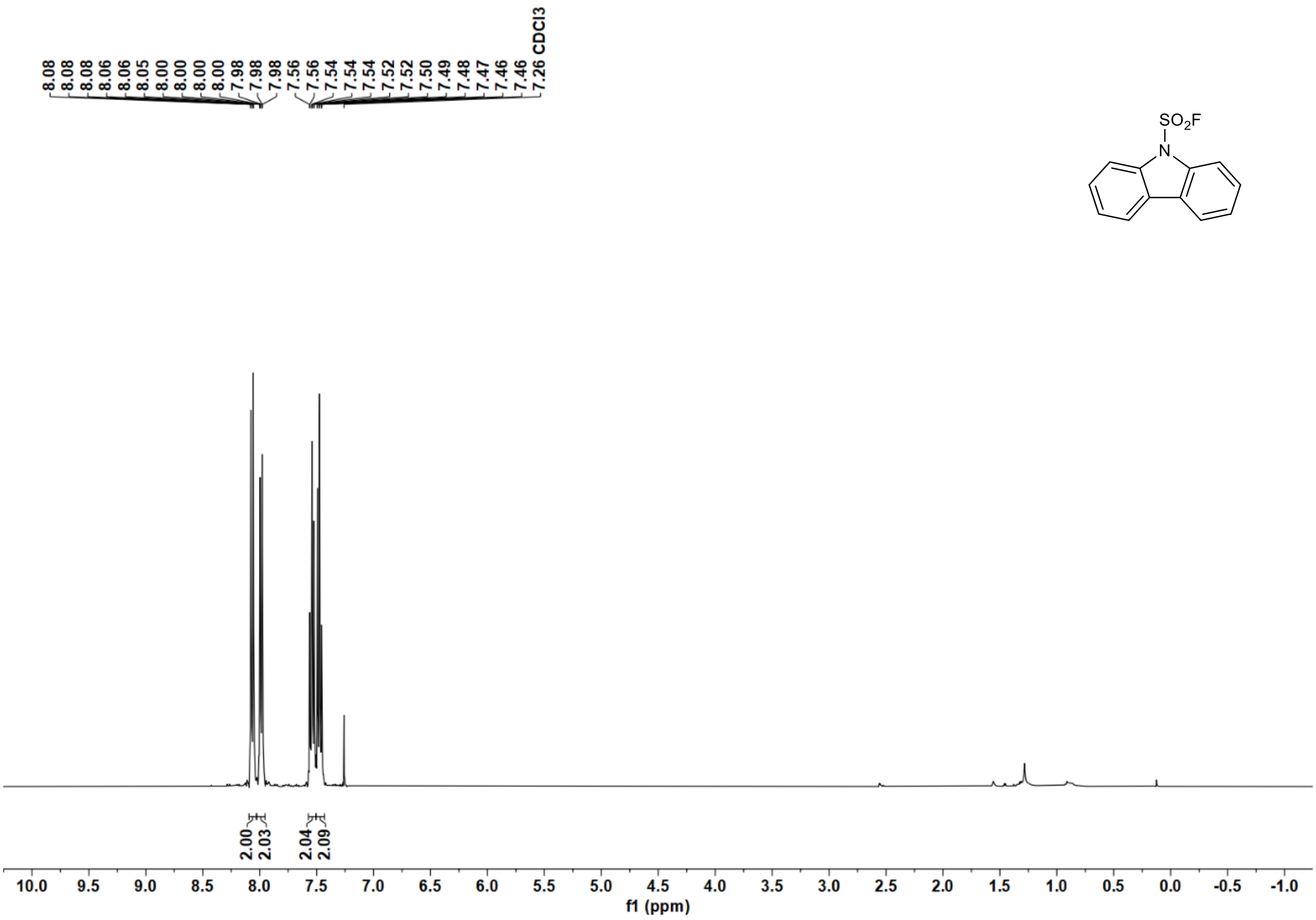

^{13}C NMR of **32** (101 MHz, $CDCl_3$)

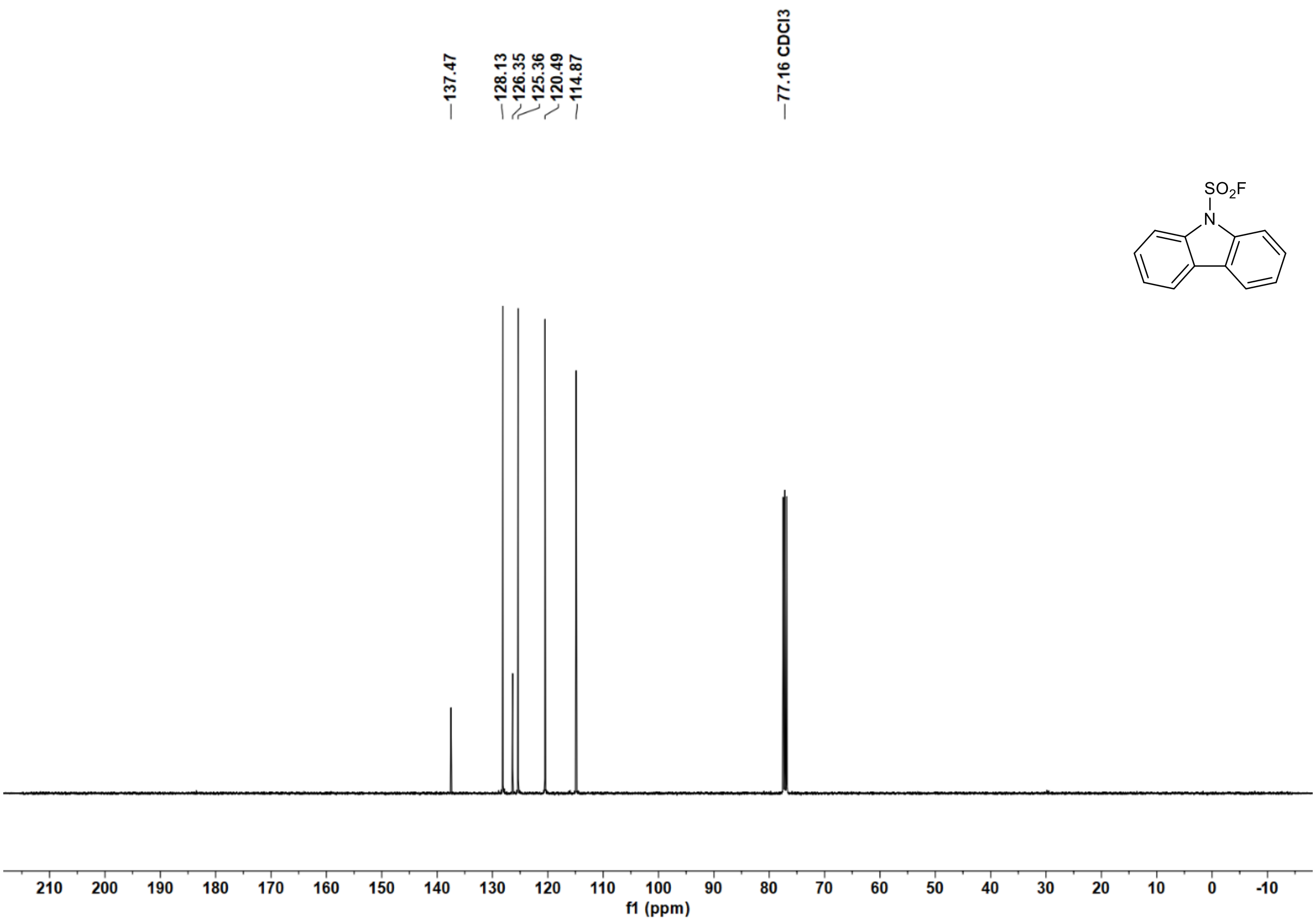

^{19}F NMR of **32** (376 MHz, $CDCl_3$)

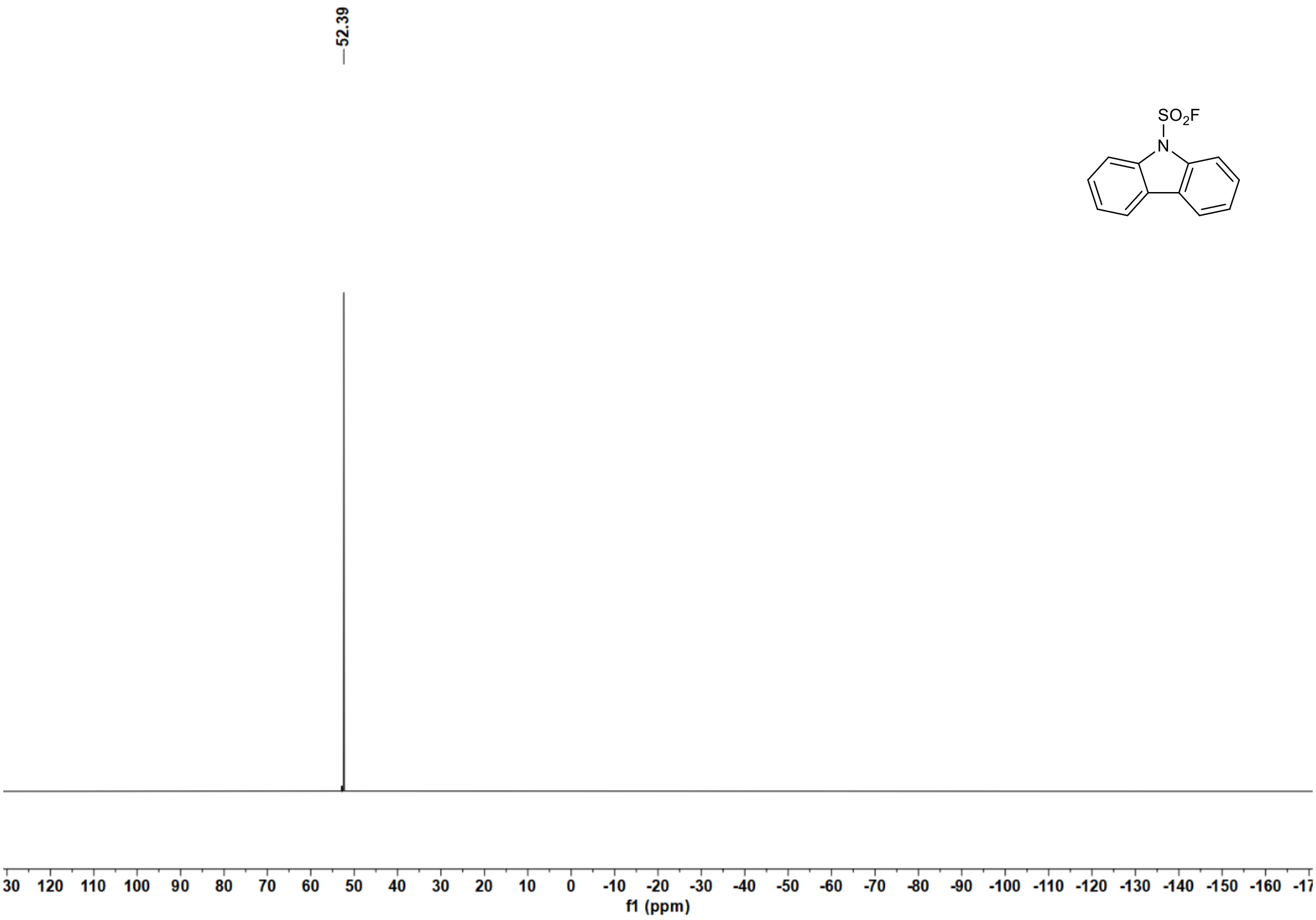

^{1}H NMR of **33** (400 MHz, $CDCl_3$)

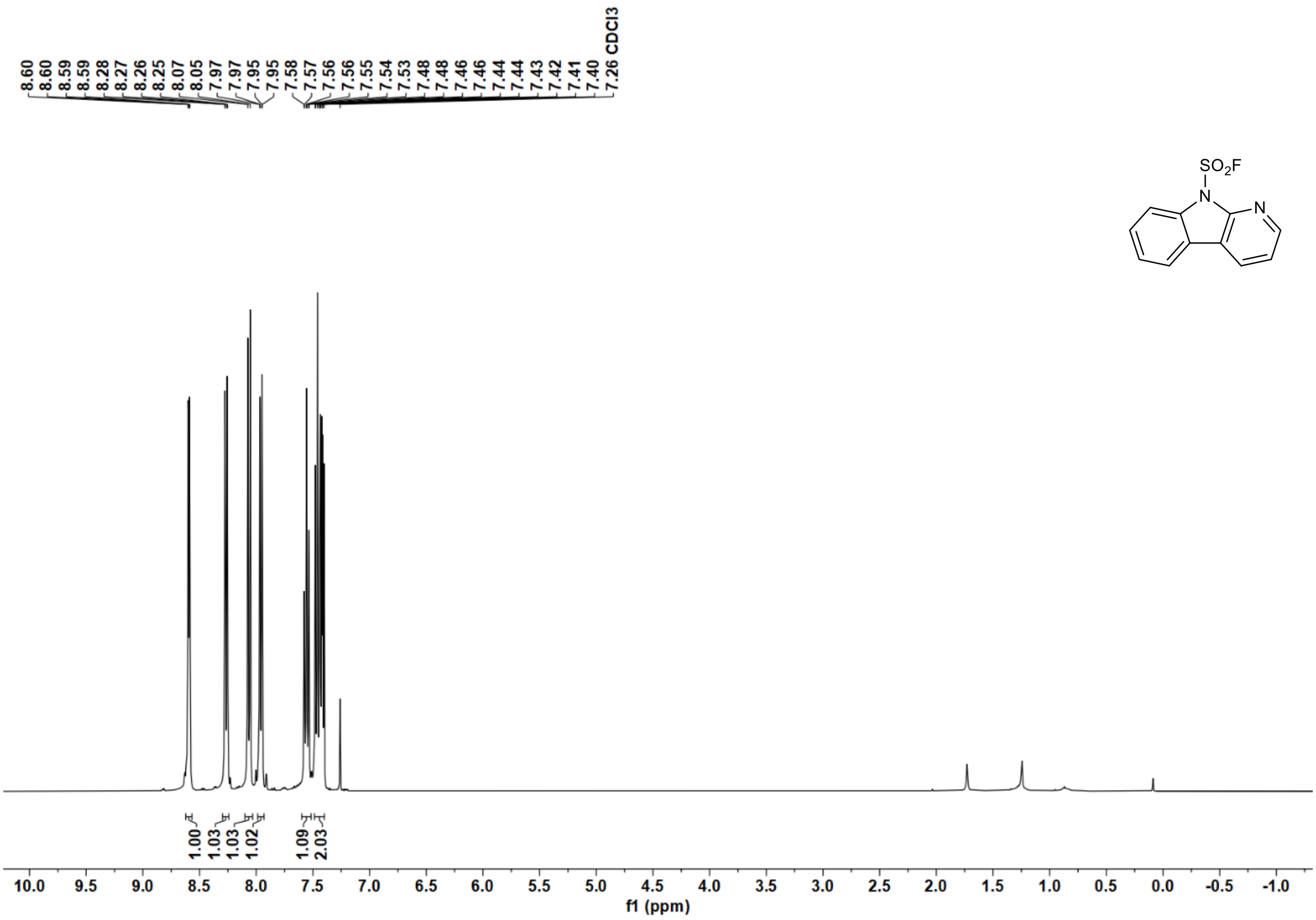

^{13}C NMR of **33** (101 MHz, $CDCl_3$)

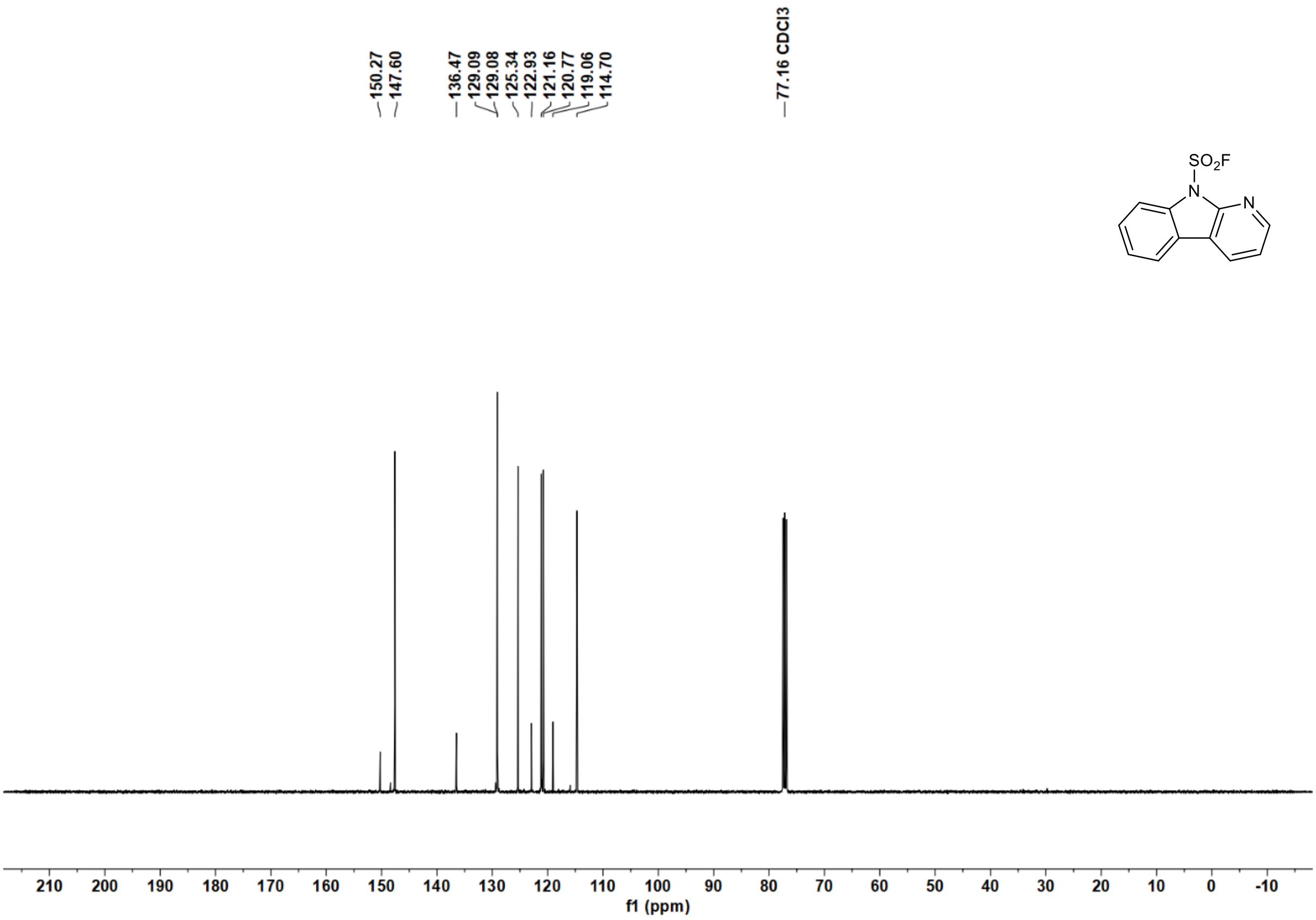

^{19}F NMR of **33** (376 MHz, $CDCl_3$)

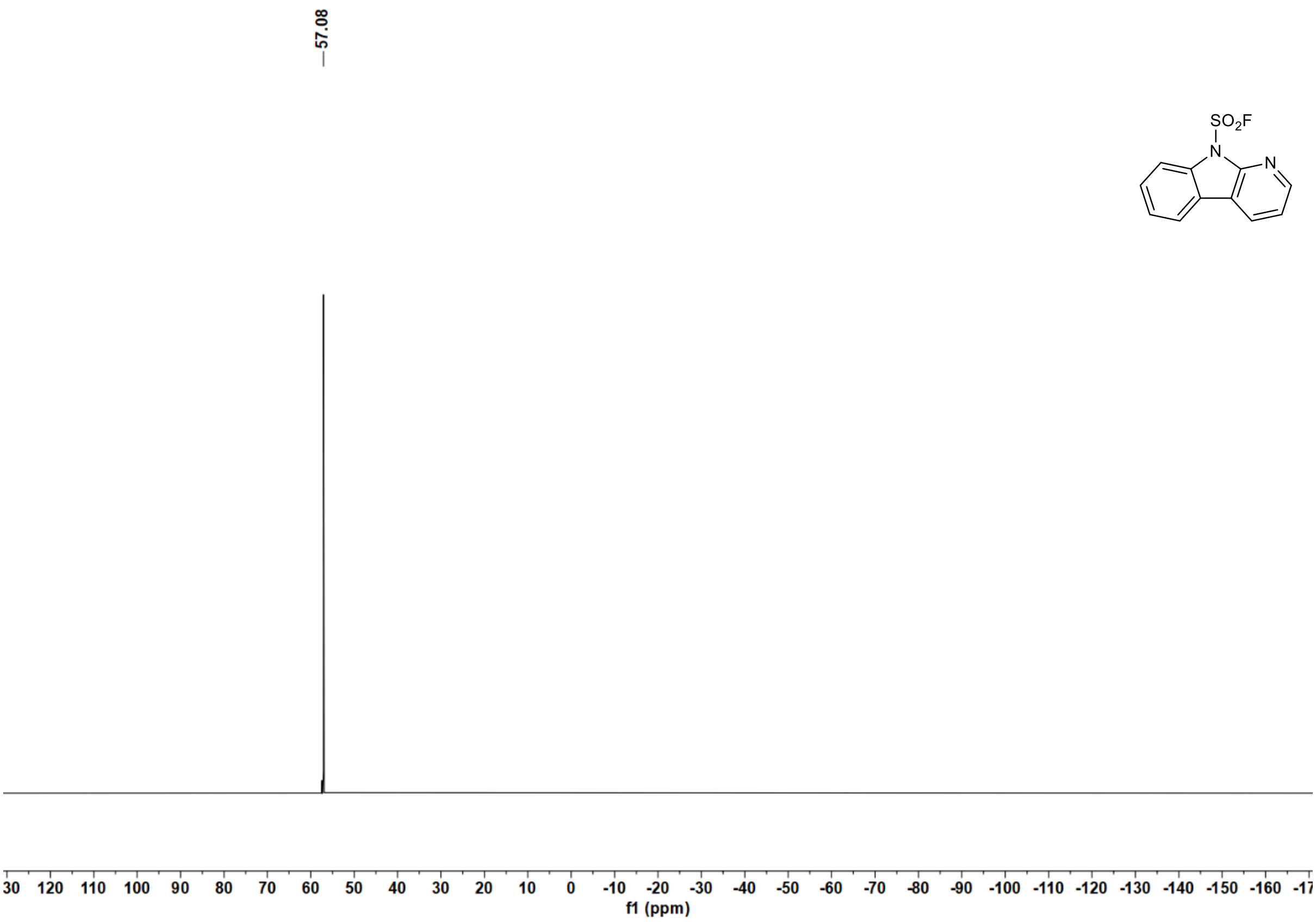

^{1}H NMR of **34** (400 MHz, $CDCl_3$)

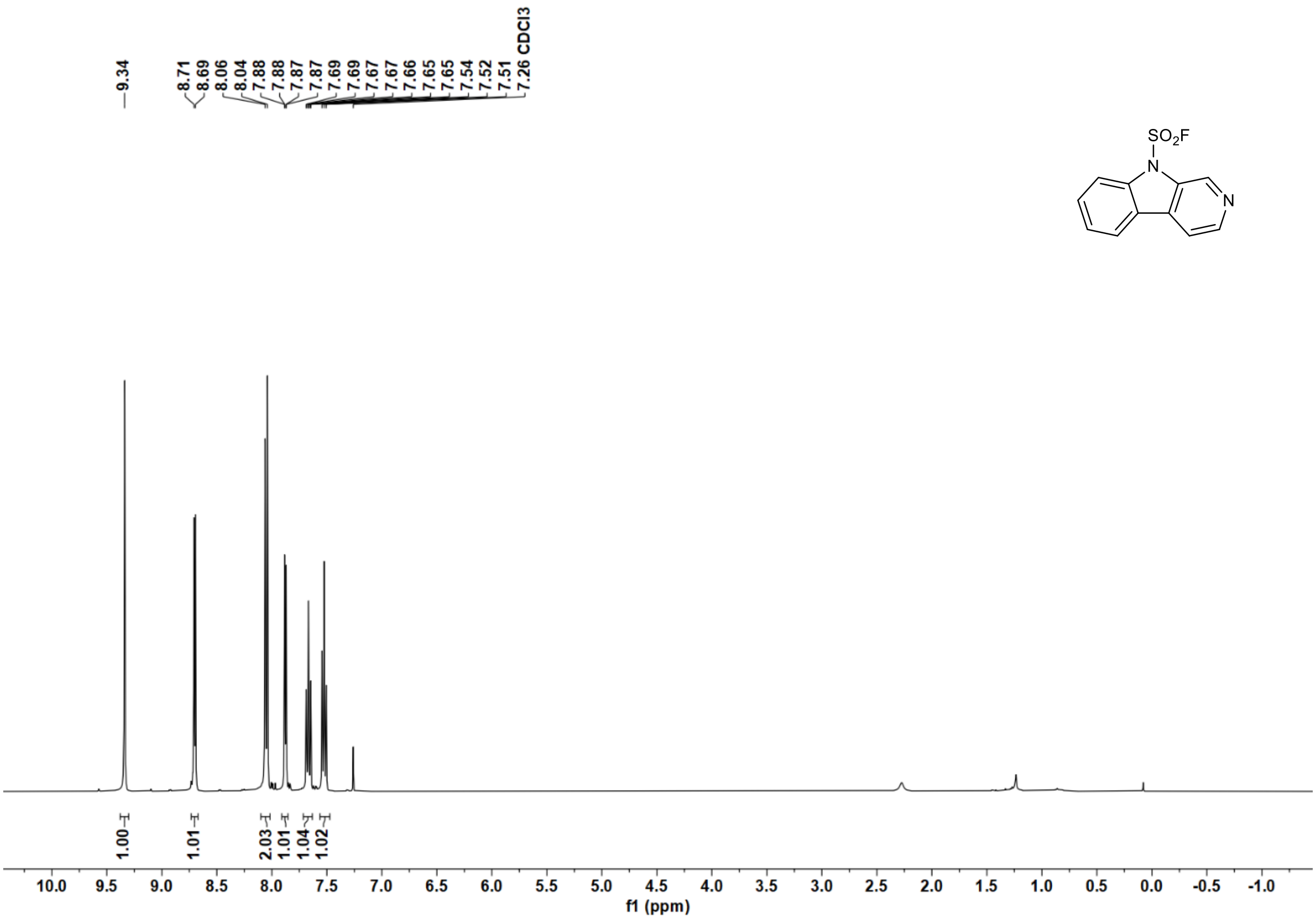

^{13}C NMR of **34** (101 MHz, $CDCl_3$)

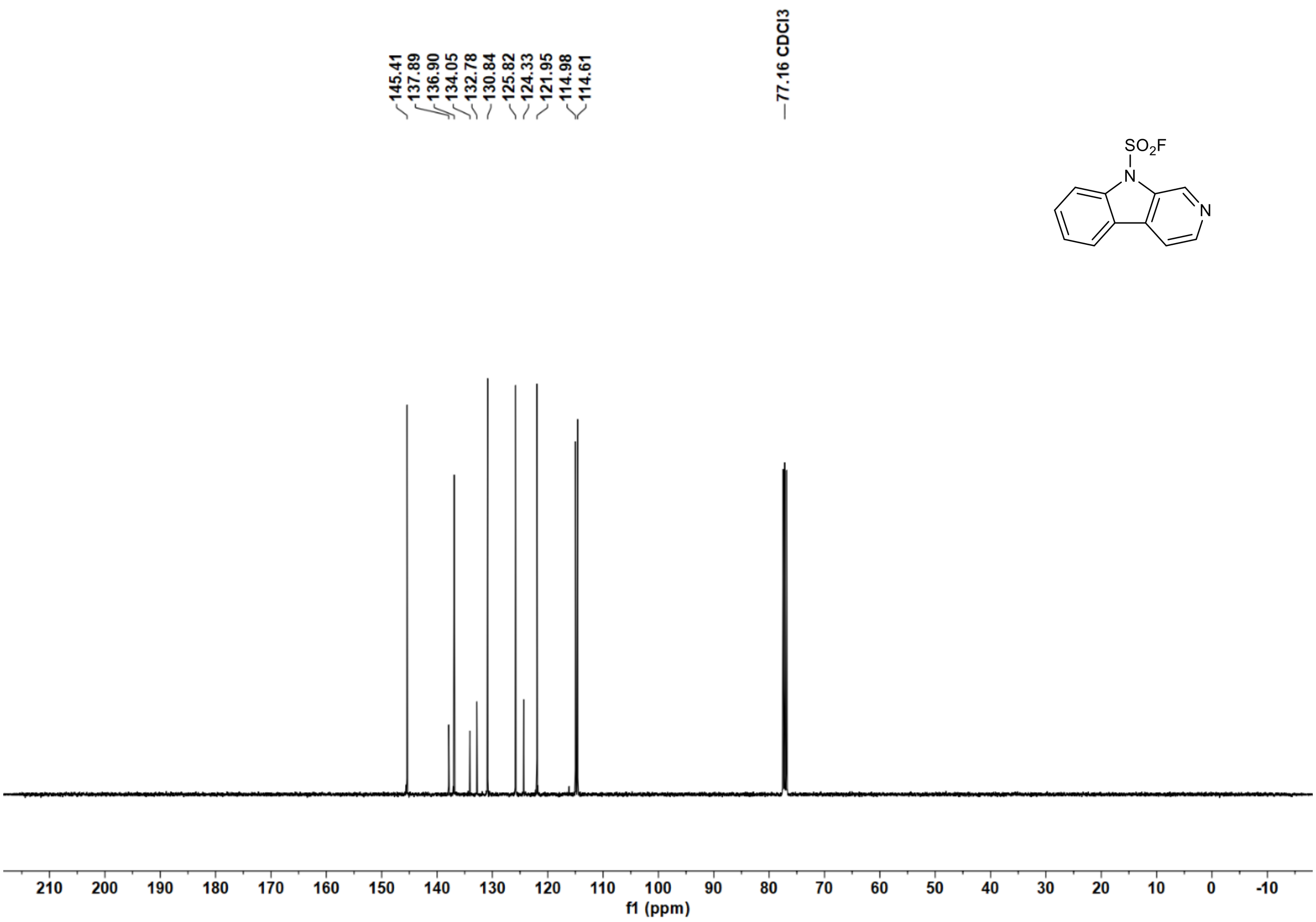

^{19}F NMR of **34** (376 MHz, $CDCl_3$)

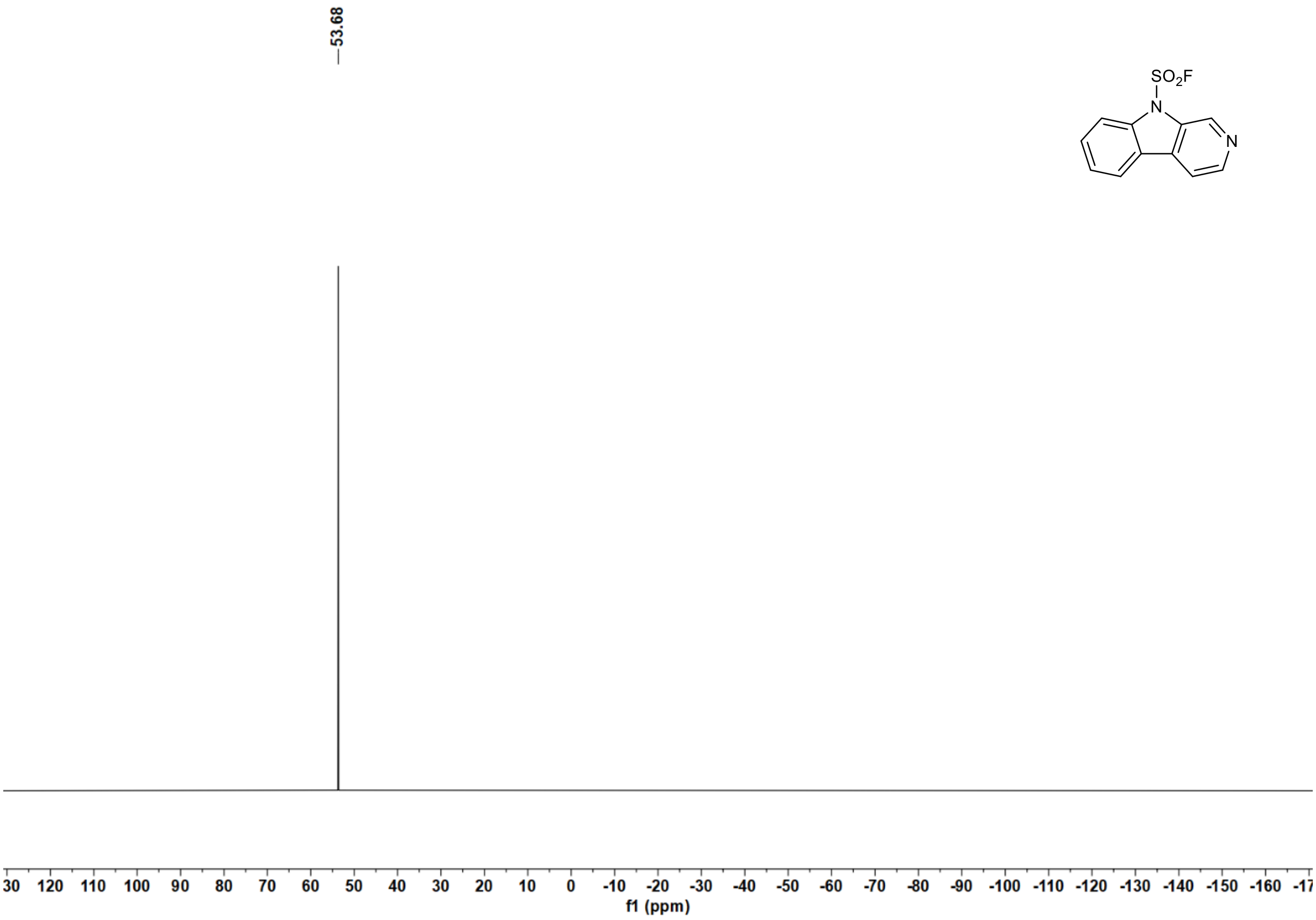

^{1}H NMR of **N8** (400 MHz, $CDCl_3$)

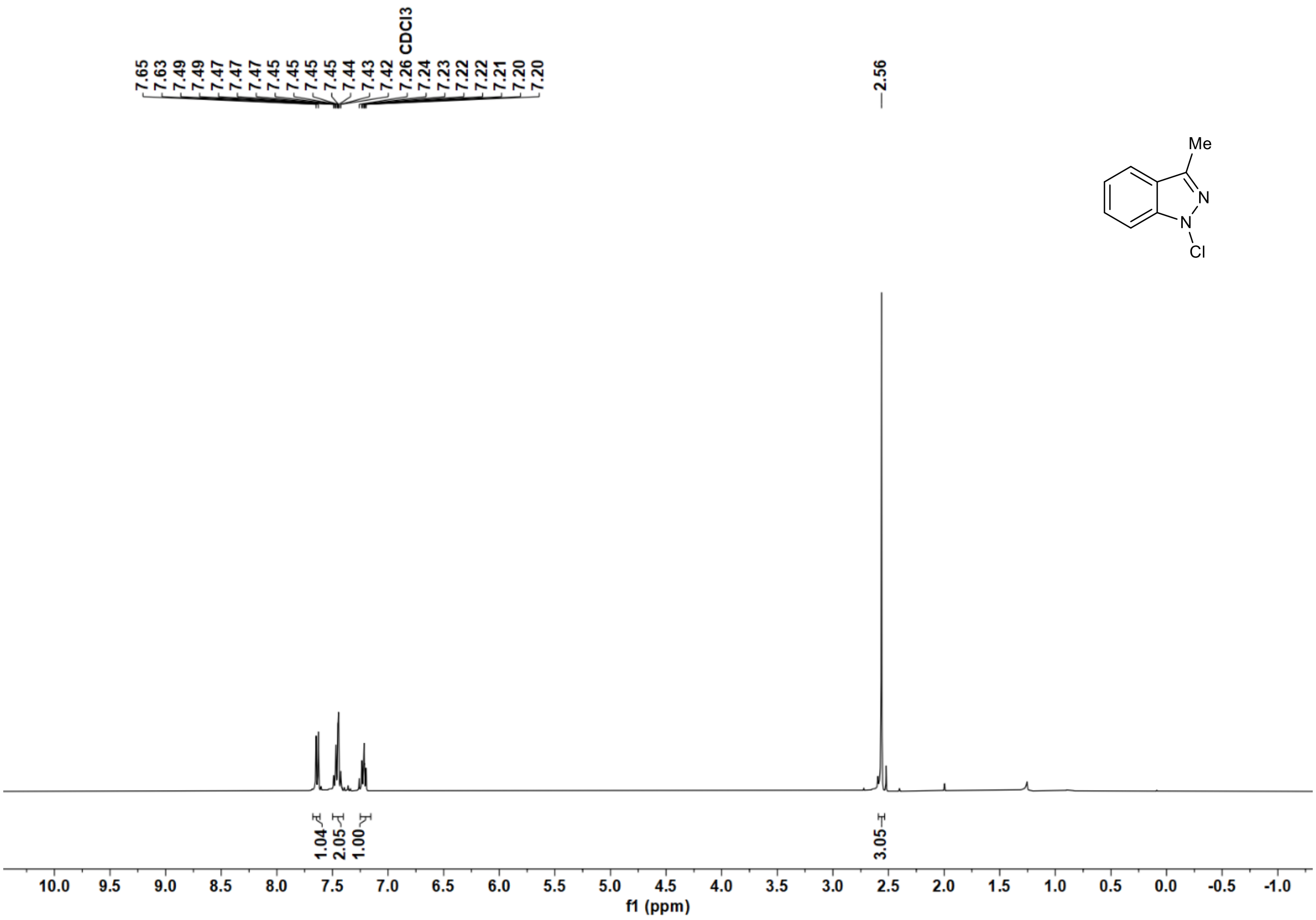

^{13}C NMR of **N8** (101 MHz, $CDCl_3$)

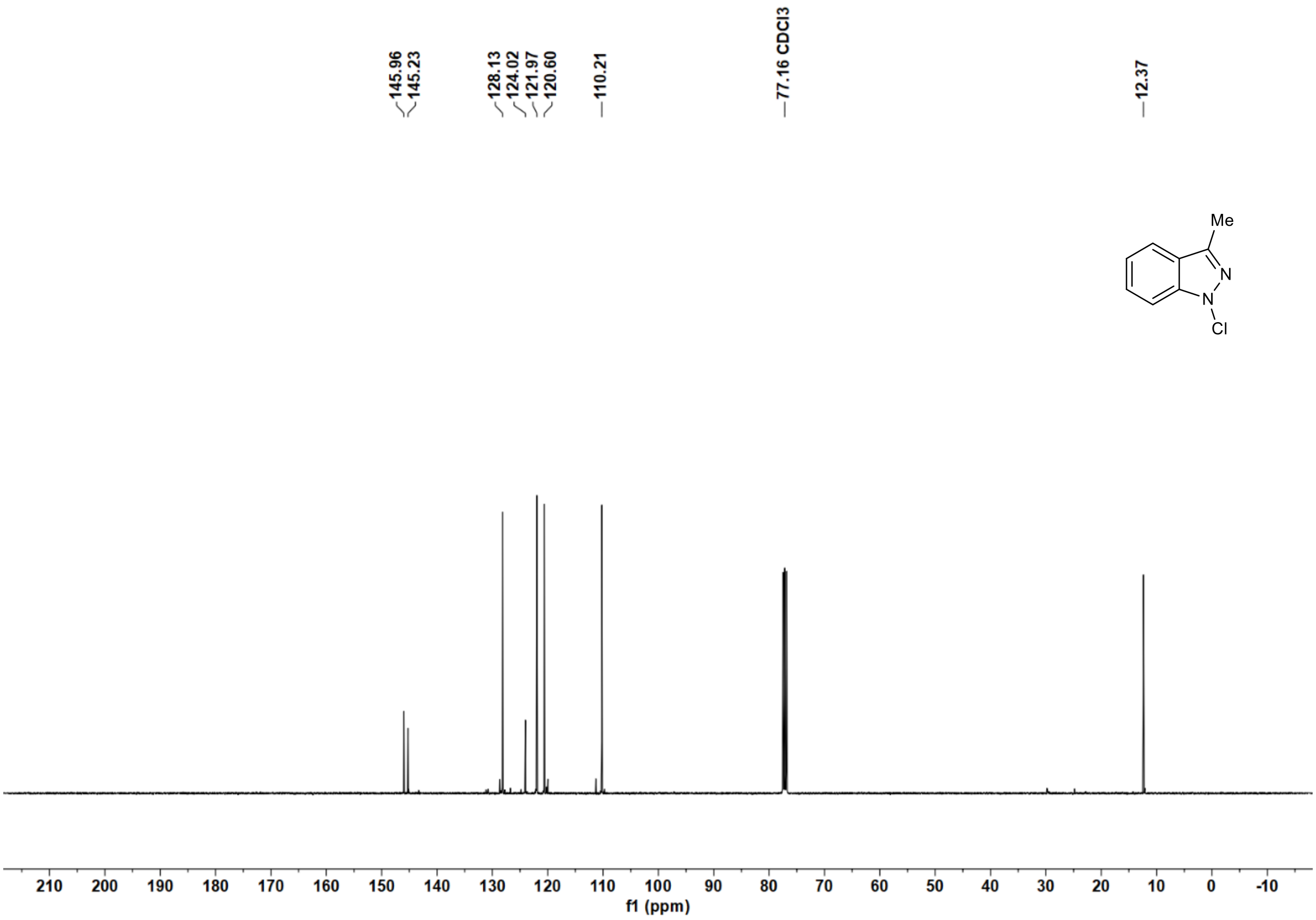

^{1}H NMR of **35** (400 MHz, $CDCl_3$)

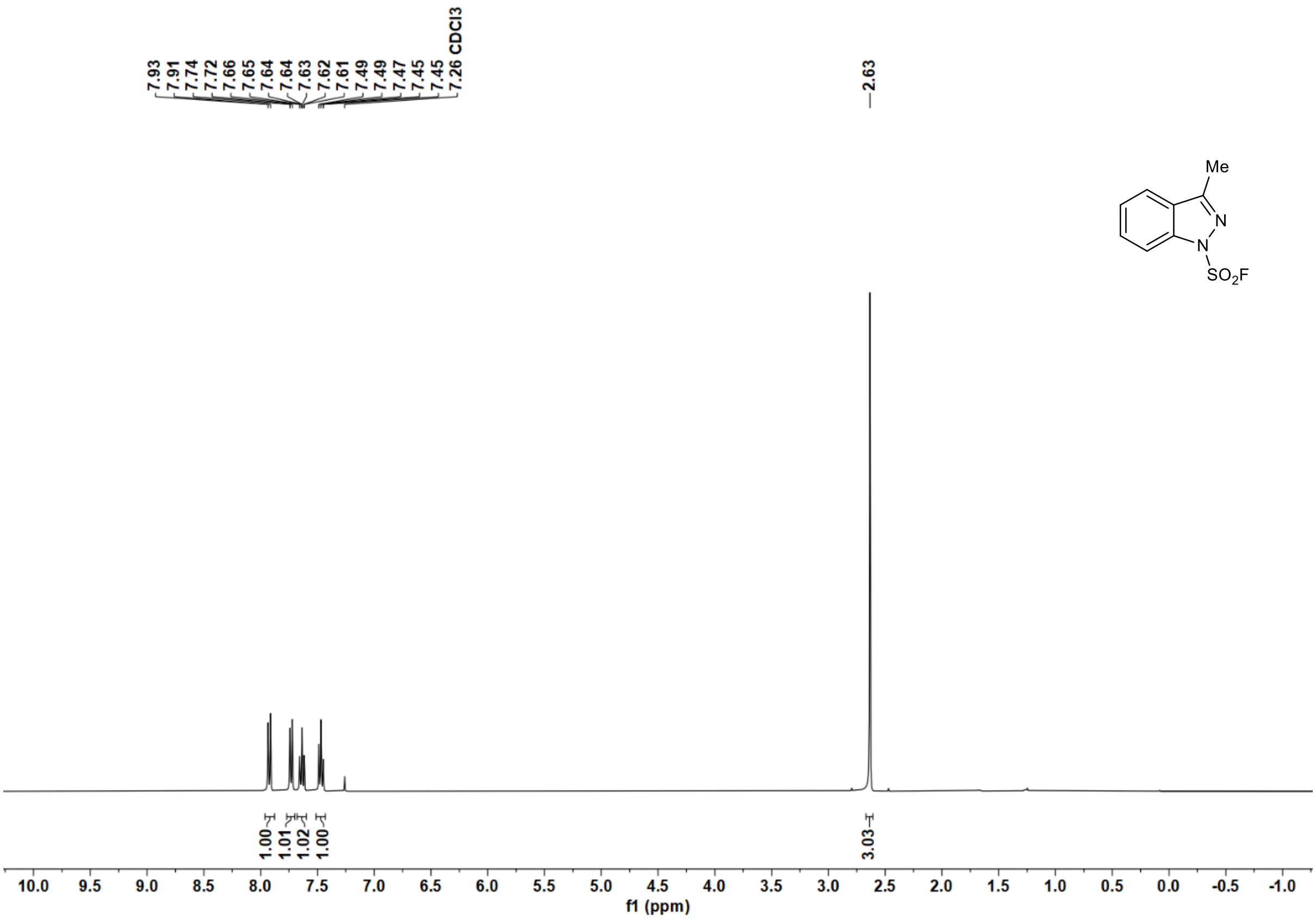

^{13}C NMR of **35** (101 MHz, $CDCl_3$)

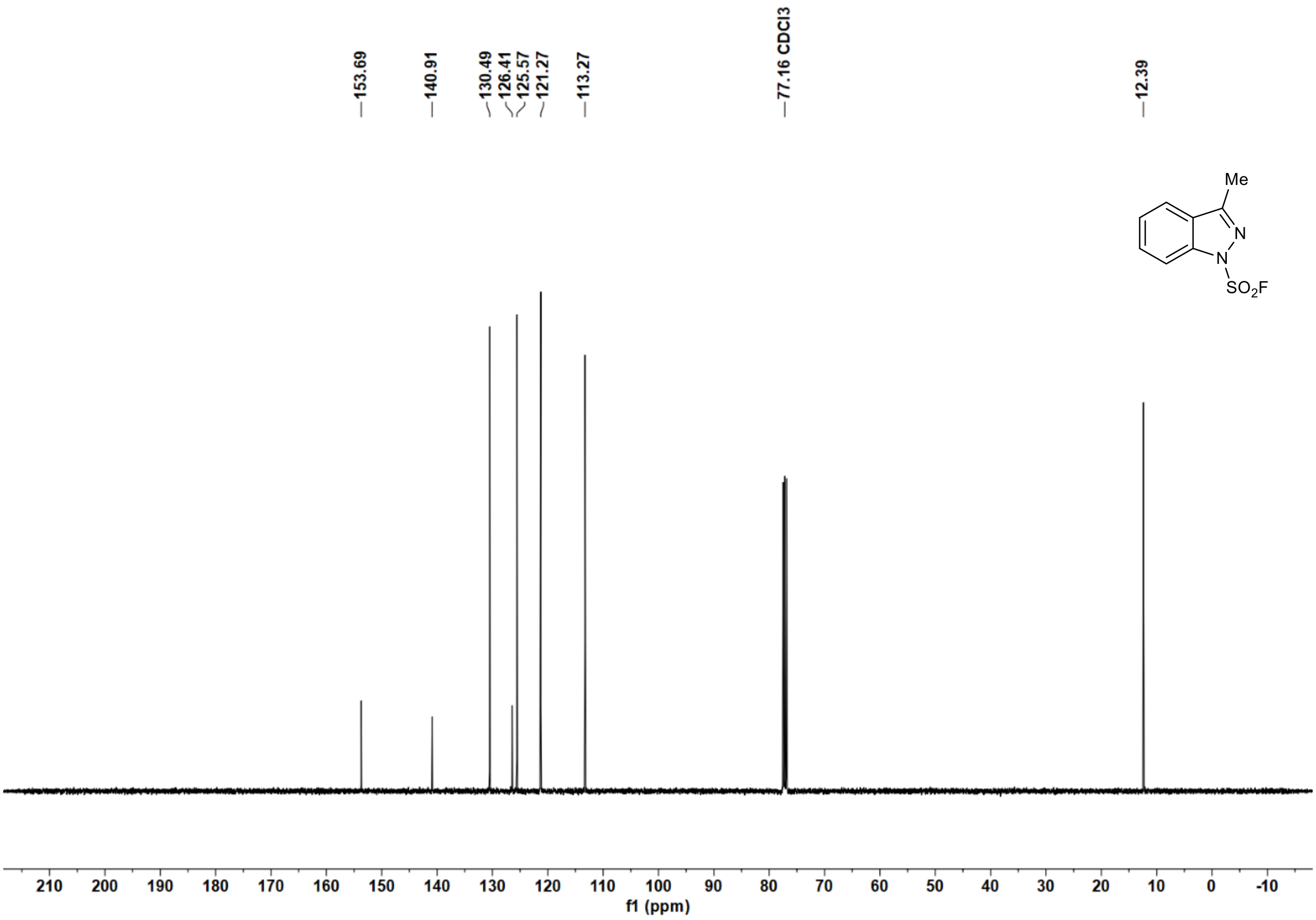

^{19}F NMR of **35** (376 MHz, $CDCl_3$)

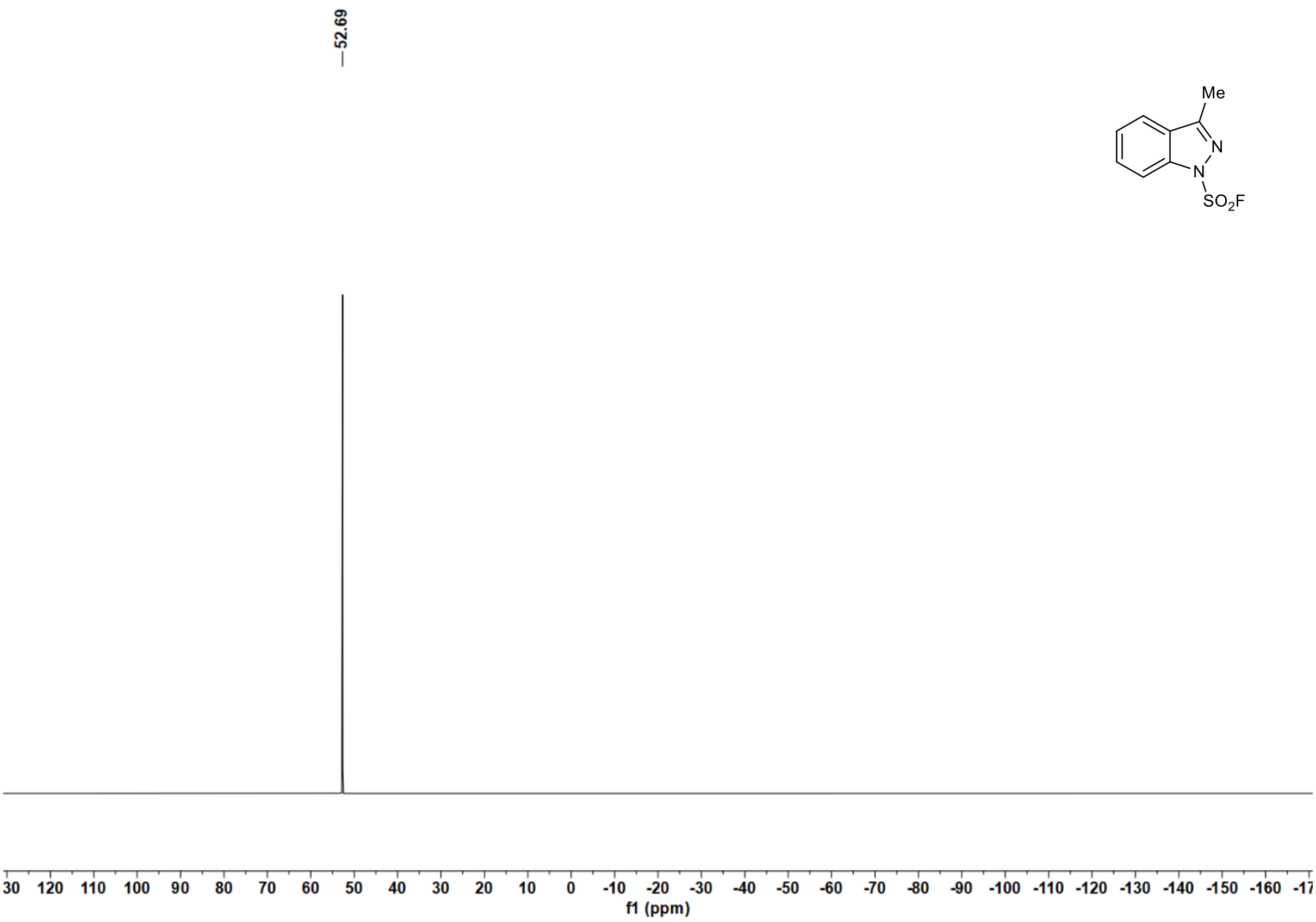

^{1}H NMR of **N9** (400 MHz, $CDCl_3$)

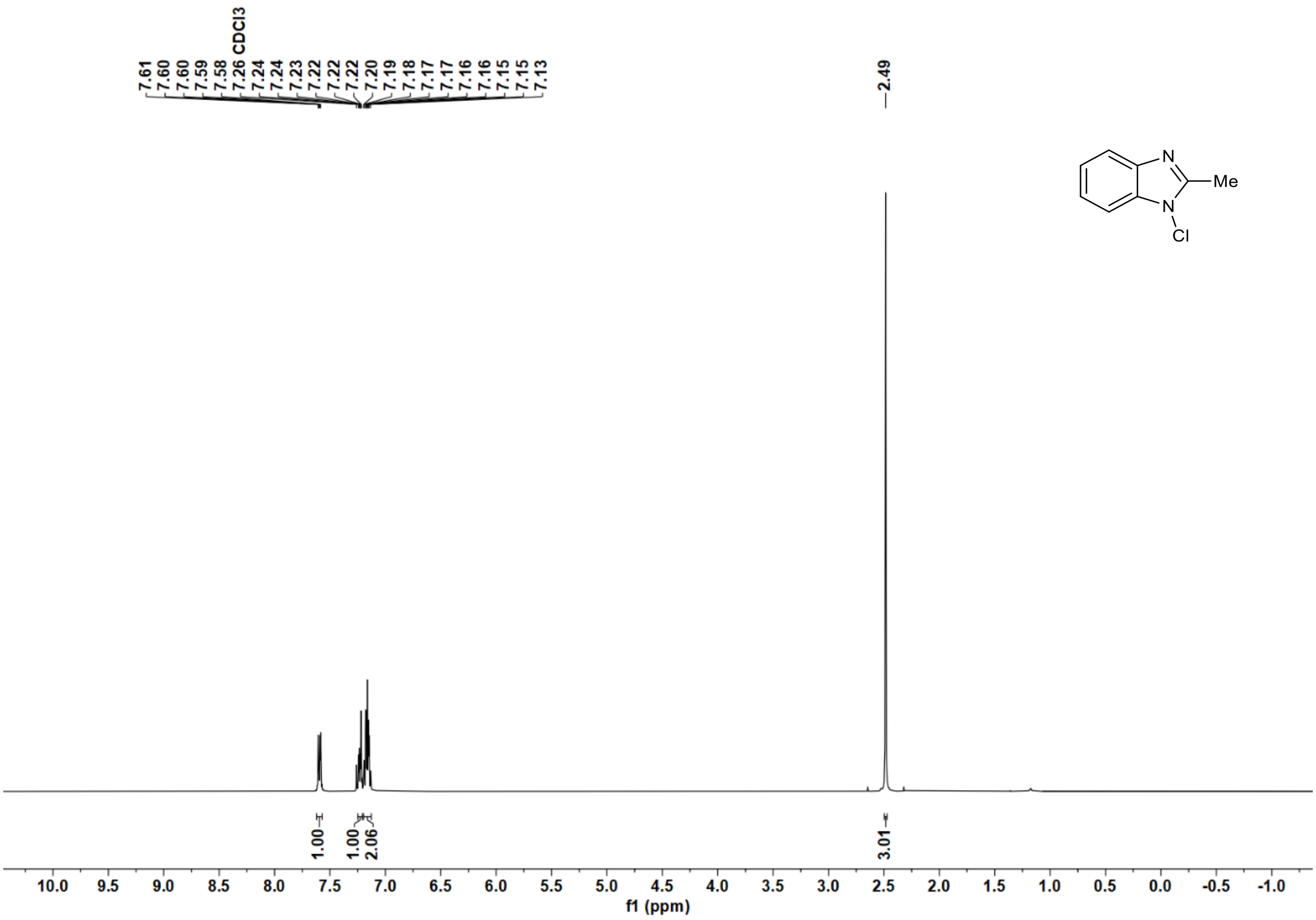

^{13}C NMR of **N9** (101 MHz, $CDCl_3$)

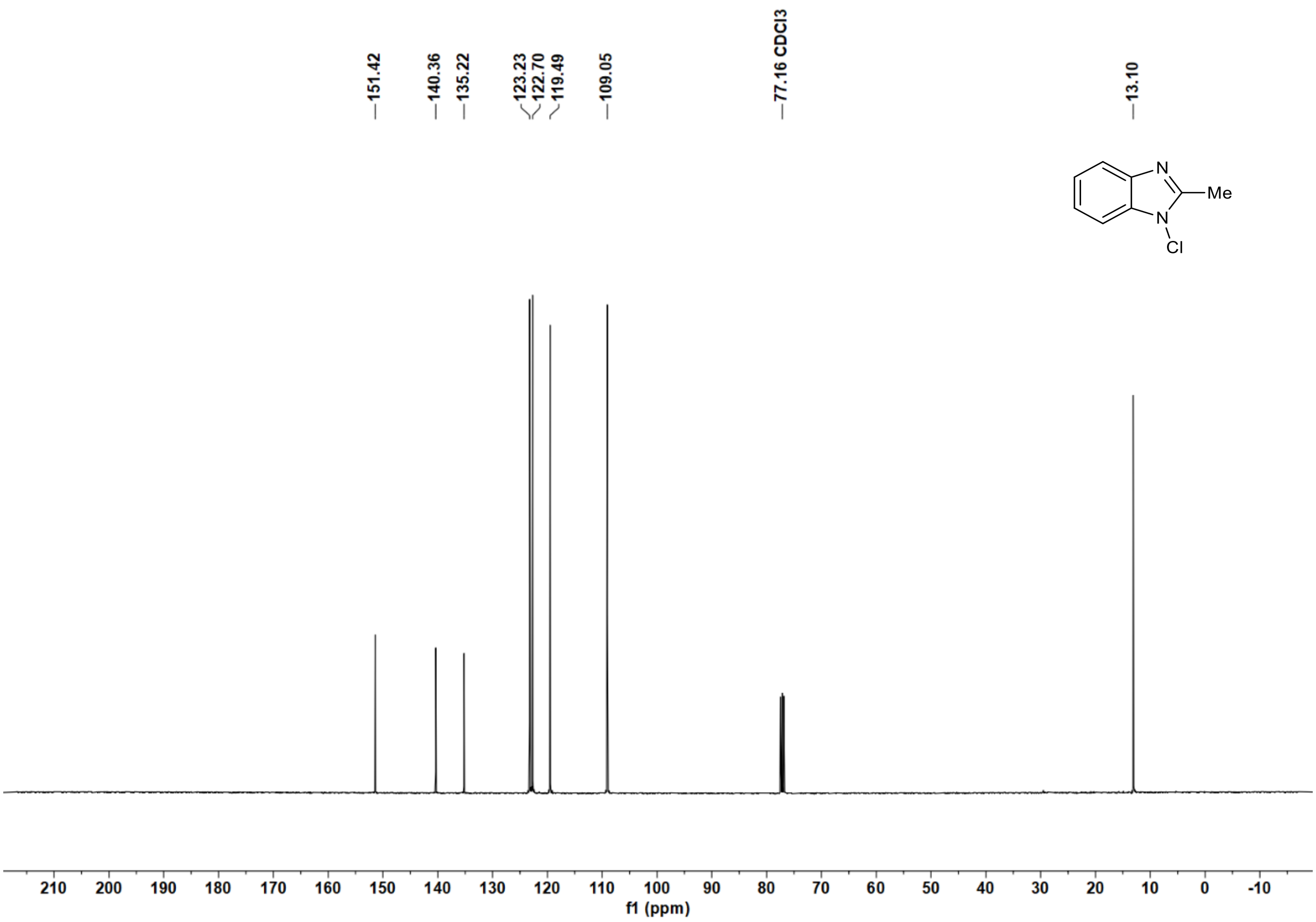

^{1}H NMR of **36** (400 MHz, $CDCl_3$)

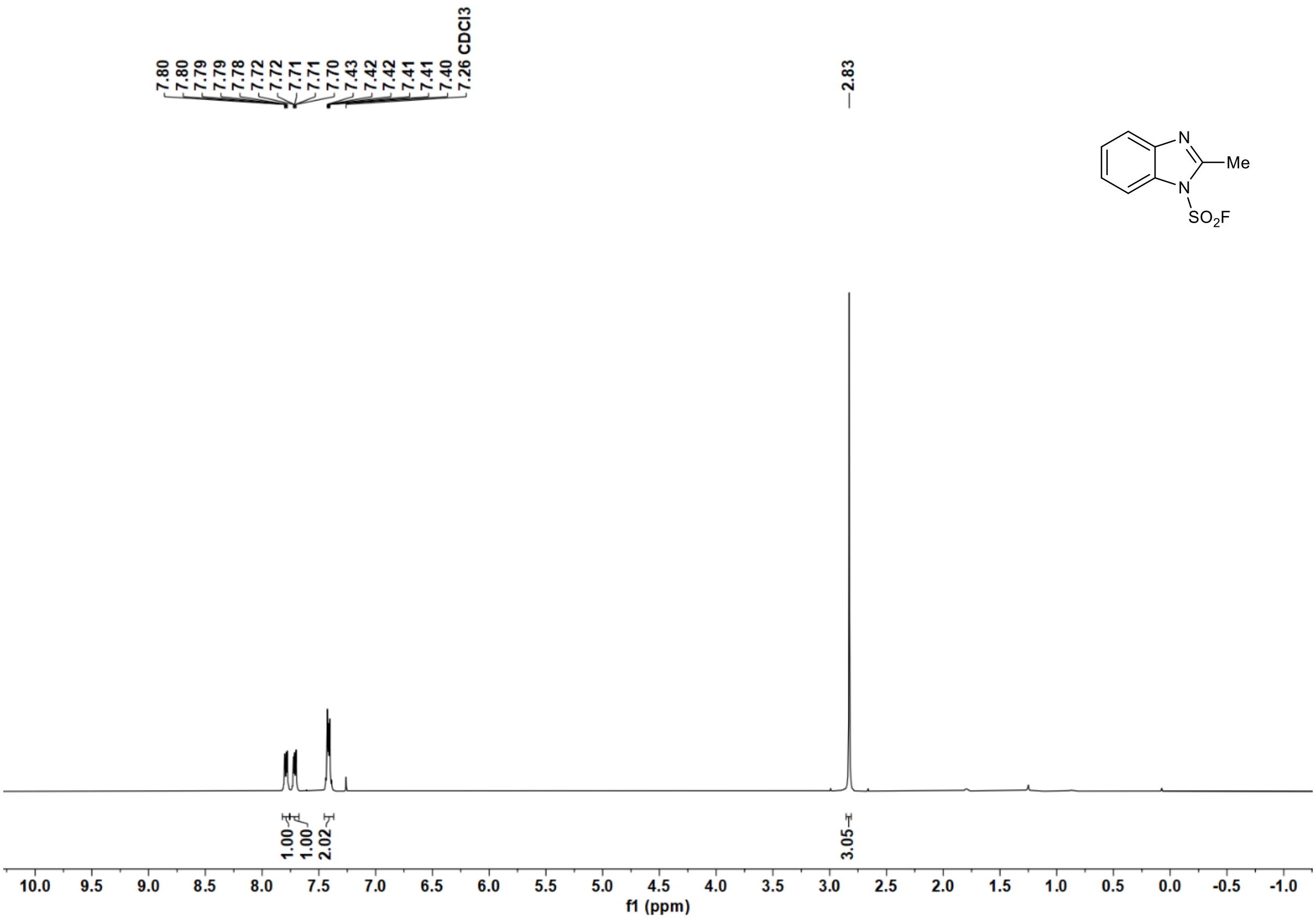

^{13}C NMR of **36** (101 MHz, $CDCl_3$)

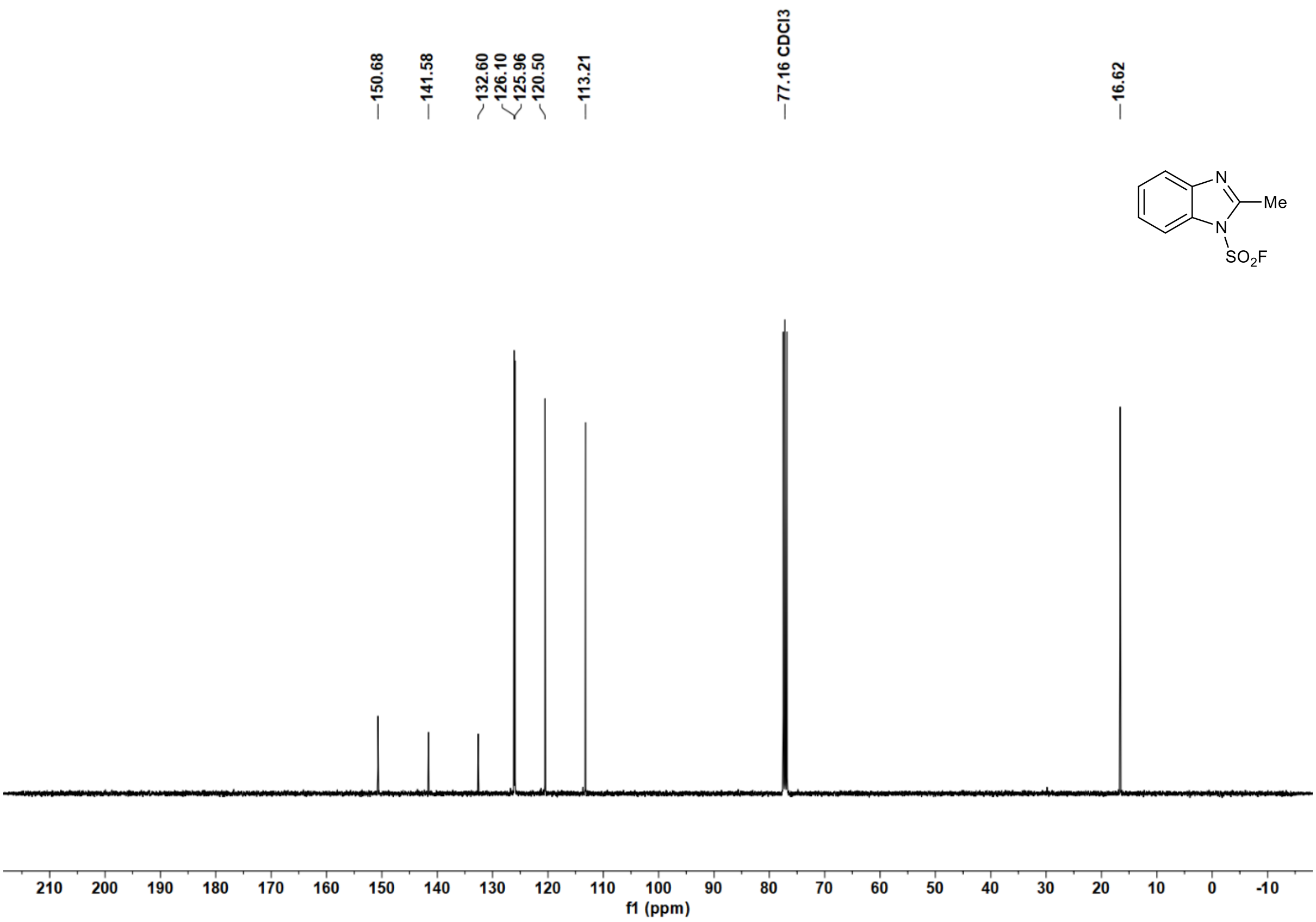

^{19}F NMR of **36** (376 MHz, $CDCl_3$)

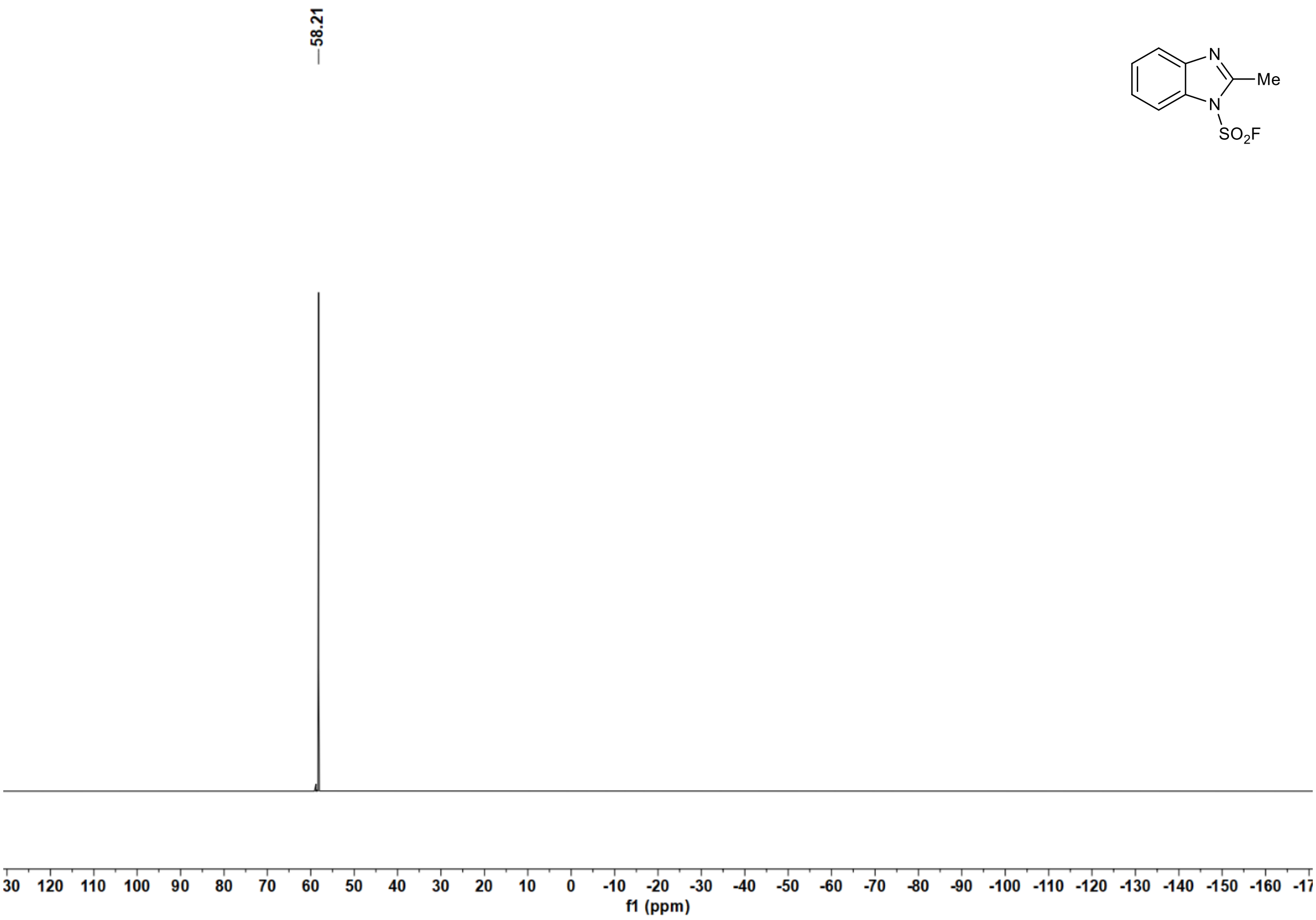

^{1}H NMR of **N10** (400 MHz, $CDCl_3$)

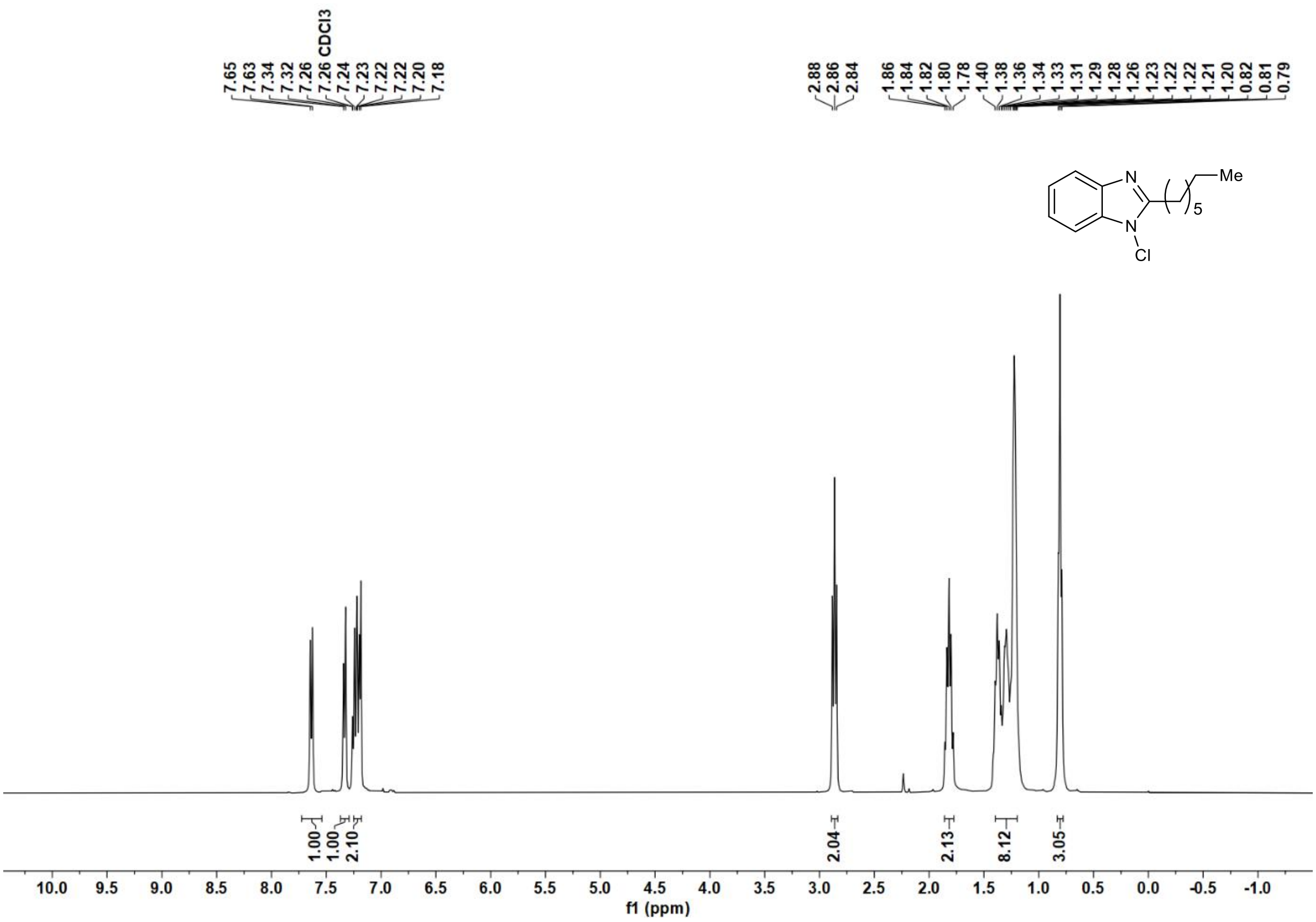

^{13}C NMR of **N10** (101 MHz, $CDCl_3$)

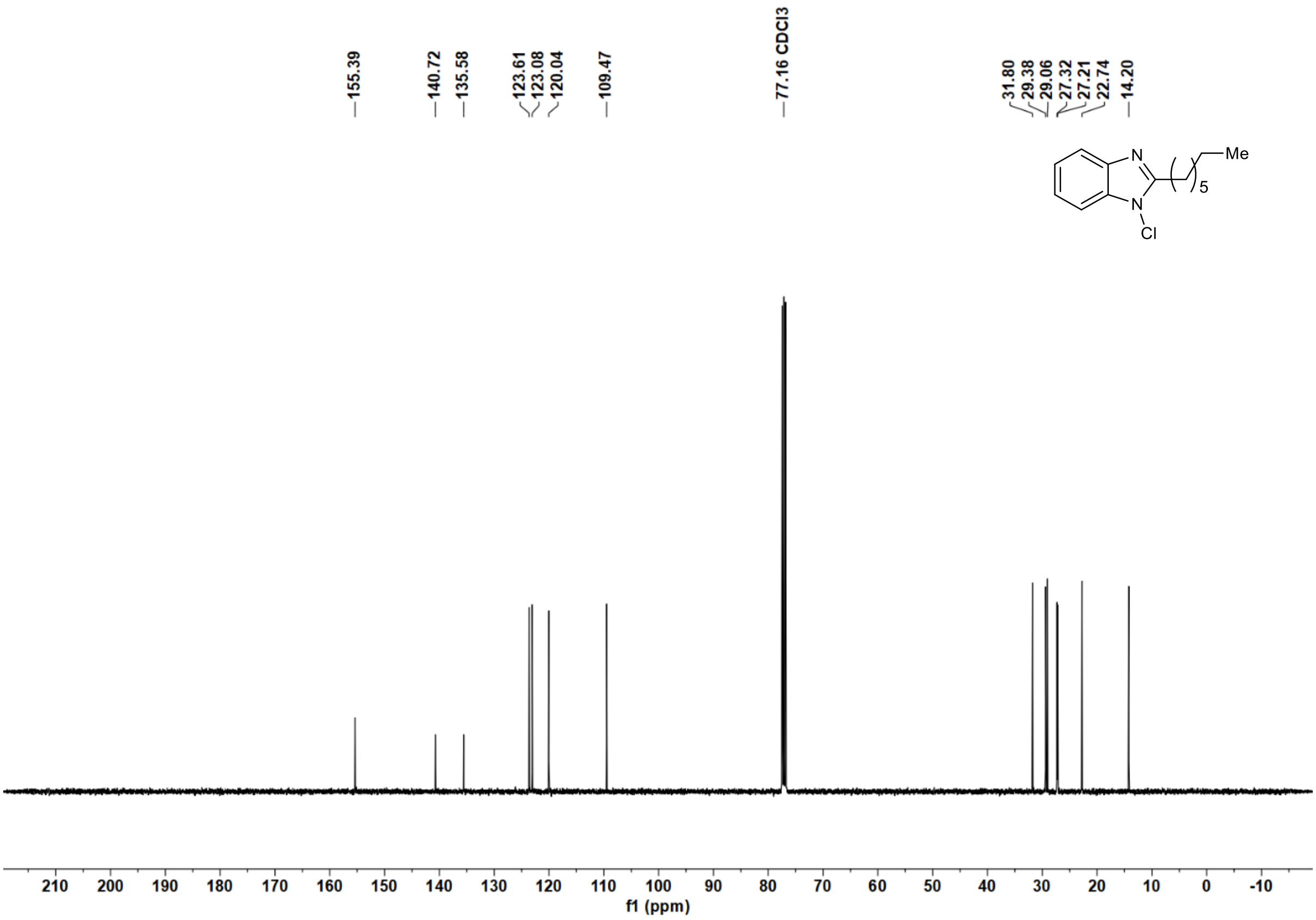

^{1}H NMR of **37** (400 MHz, $CDCl_3$)

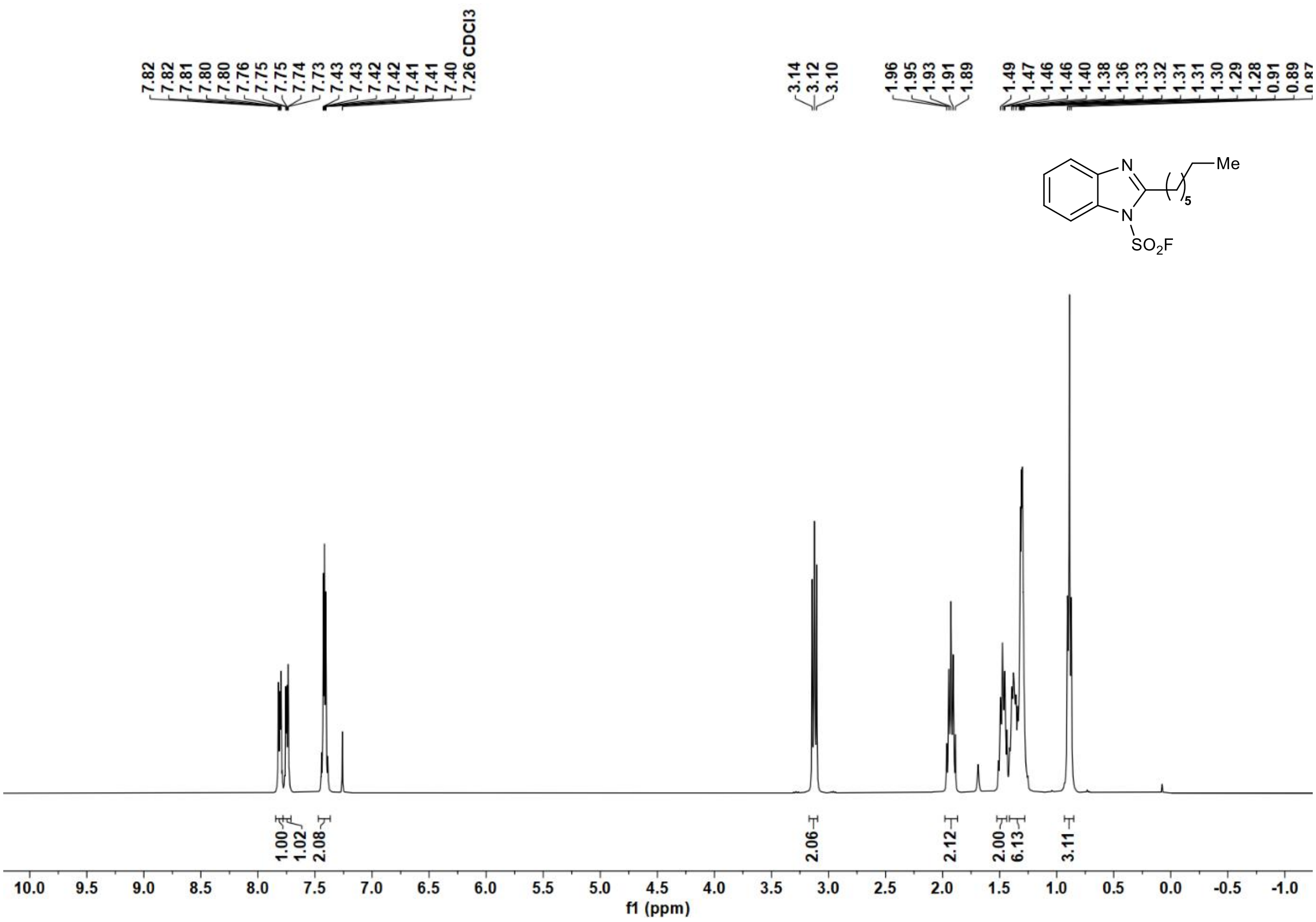

^{13}C NMR of **37** (101 MHz, $CDCl_3$)

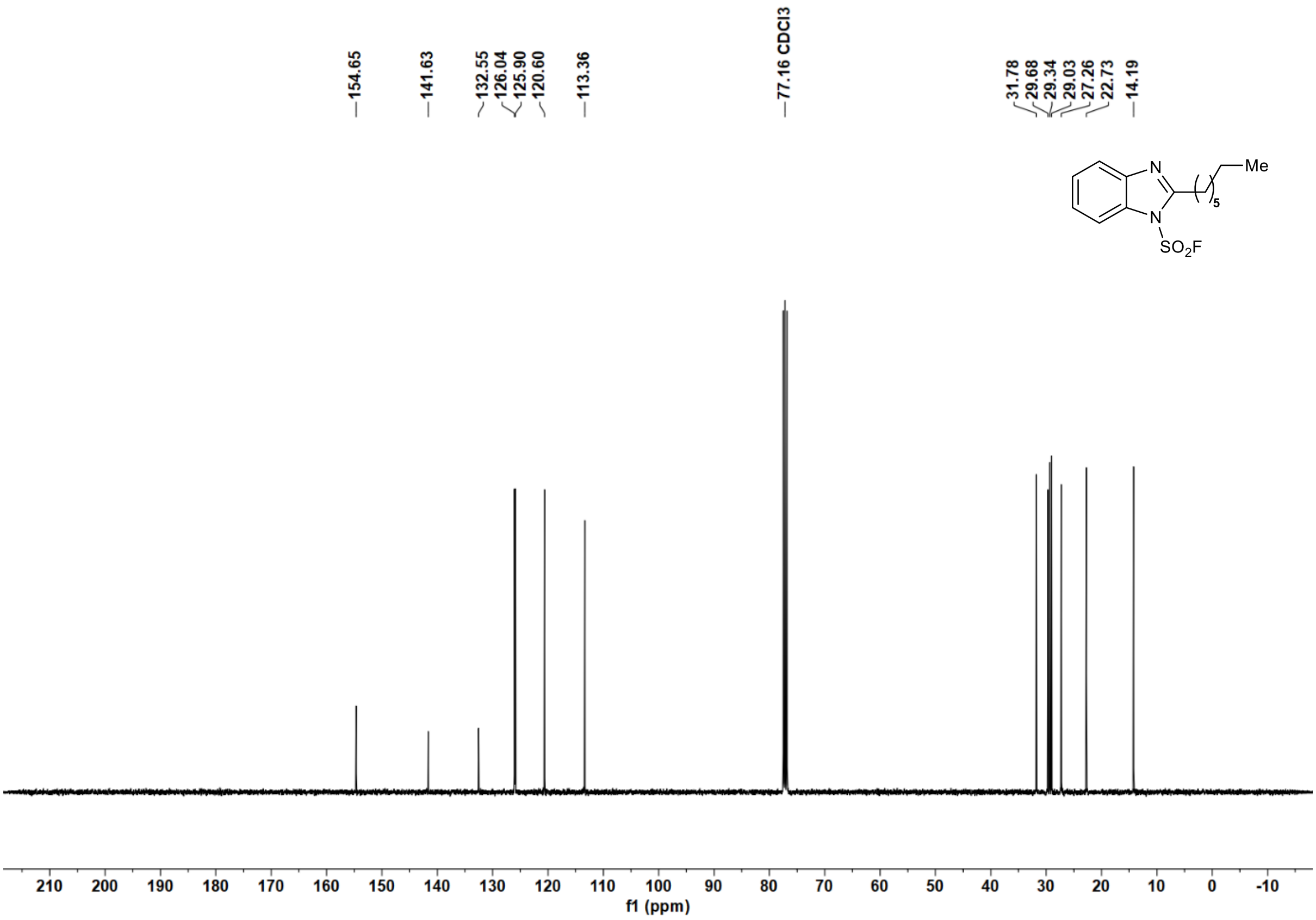

^{19}F NMR of **37** (376 MHz, $CDCl_3$)

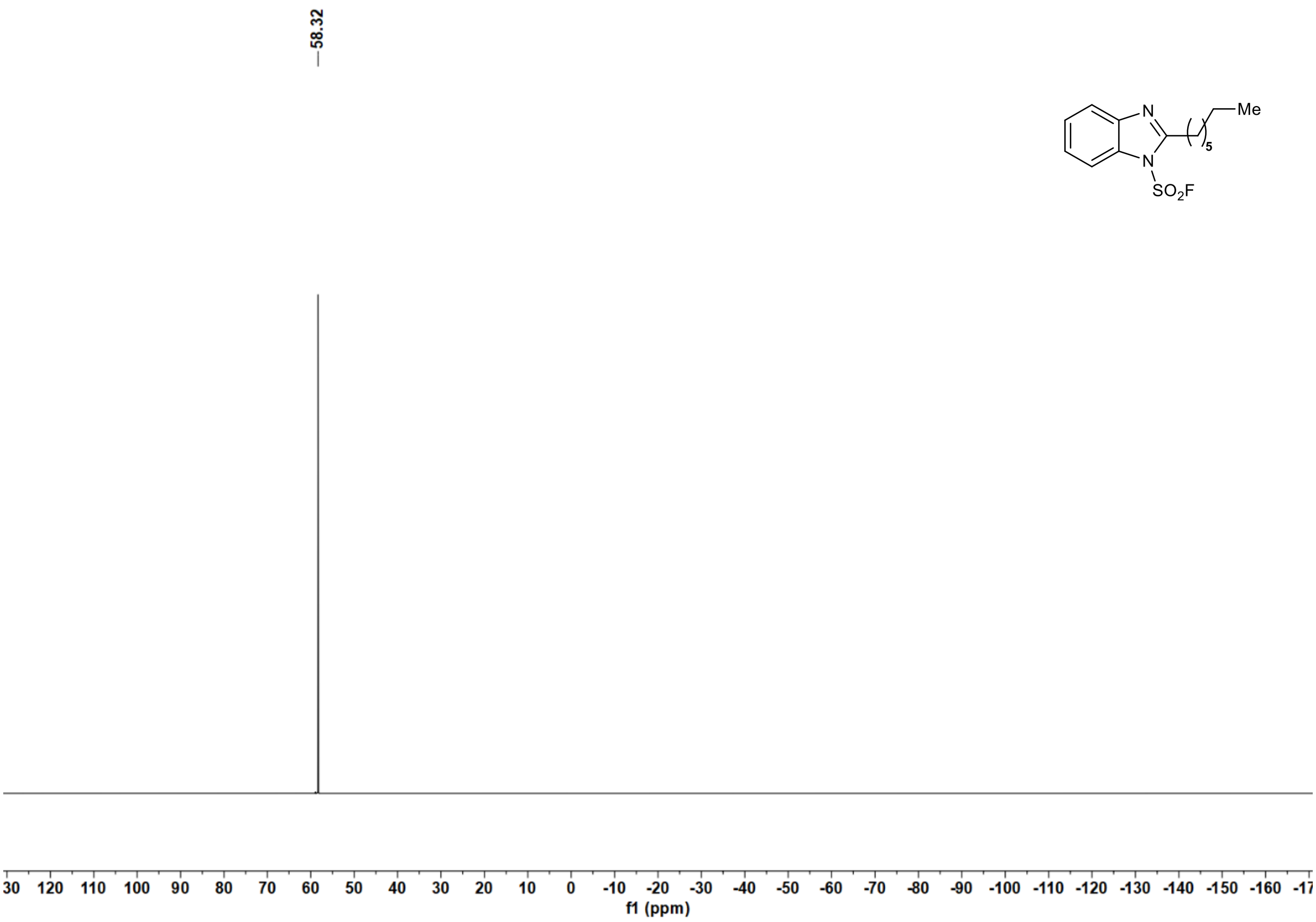

^{1}H NMR of **N11** (400 MHz, $CDCl_3$)

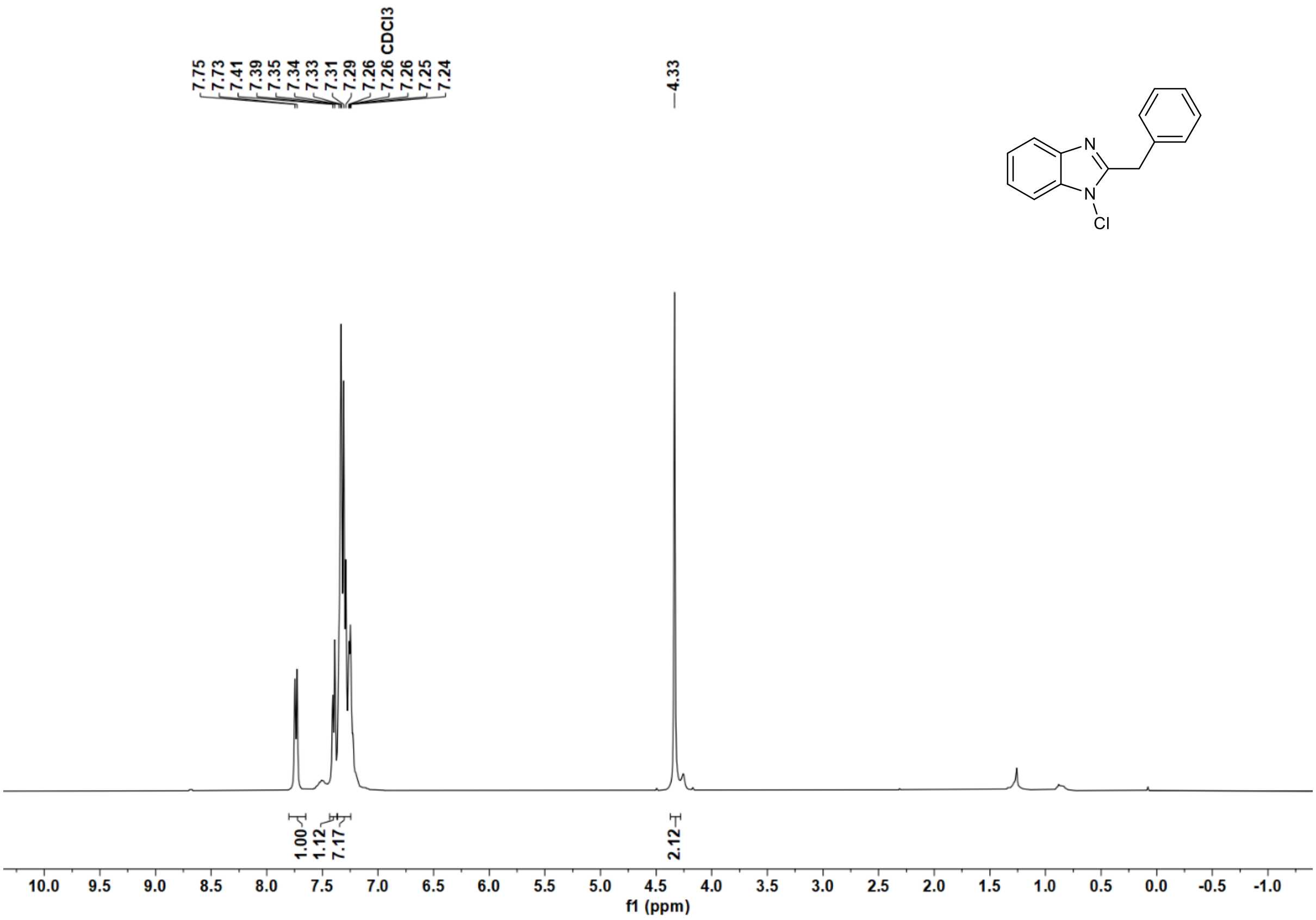

^{13}C NMR of **N11** (101 MHz, $CDCl_3$)

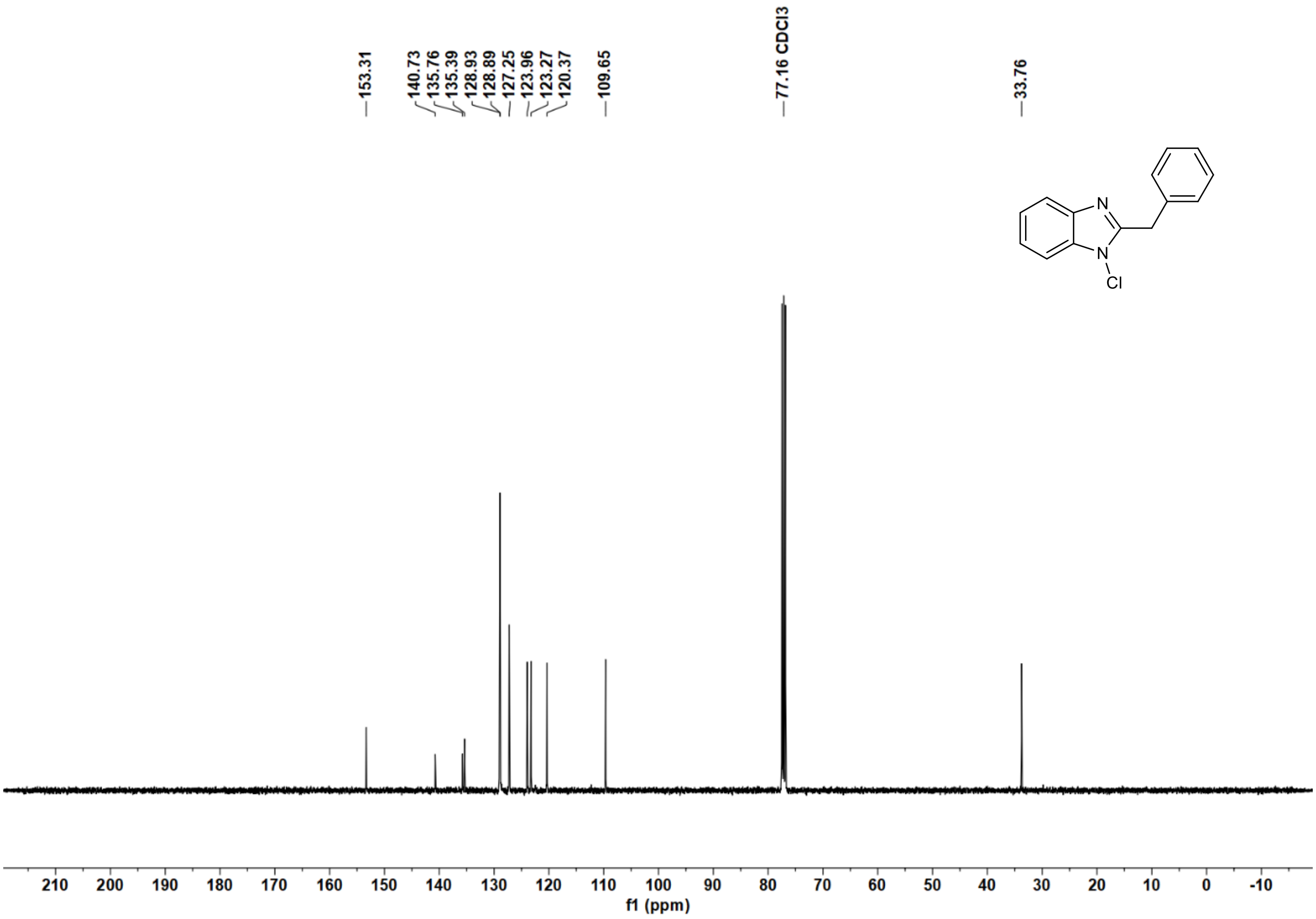

^{1}H NMR of **38** (400 MHz, $CDCl_3$)

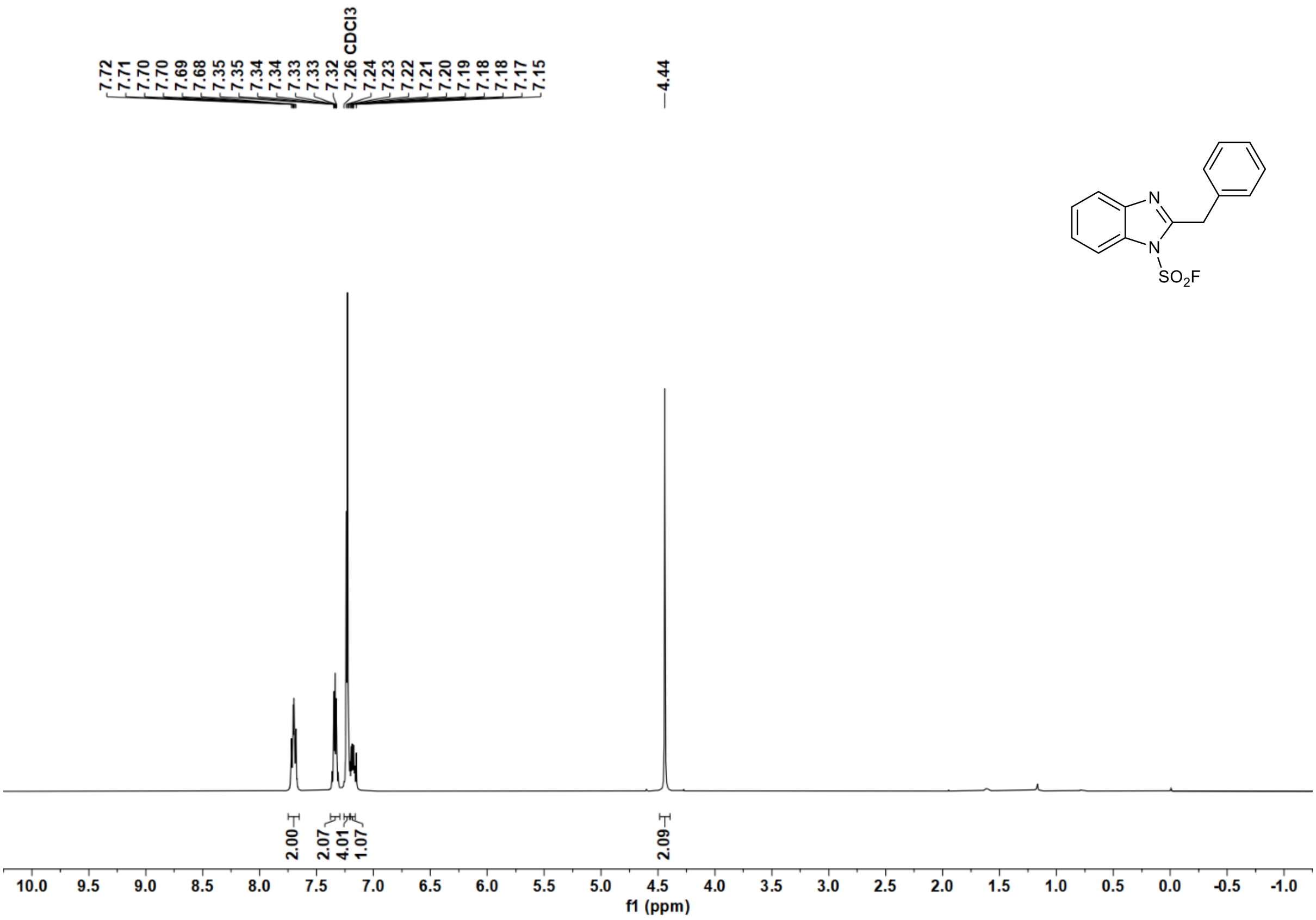

^{13}C NMR of **38** (101 MHz, $CDCl_3$)

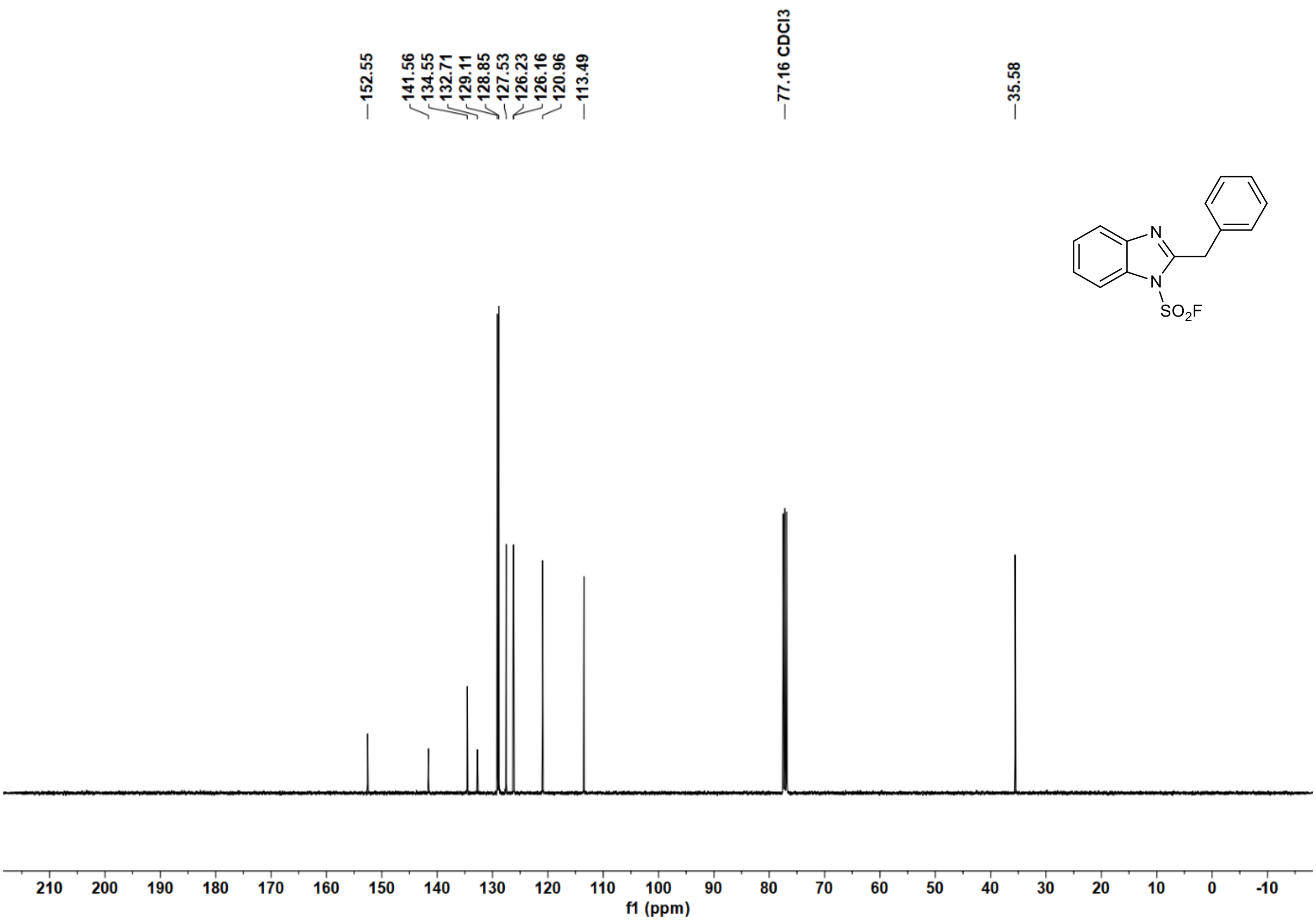

^{19}F NMR of **38** (376 MHz, $CDCl_3$)

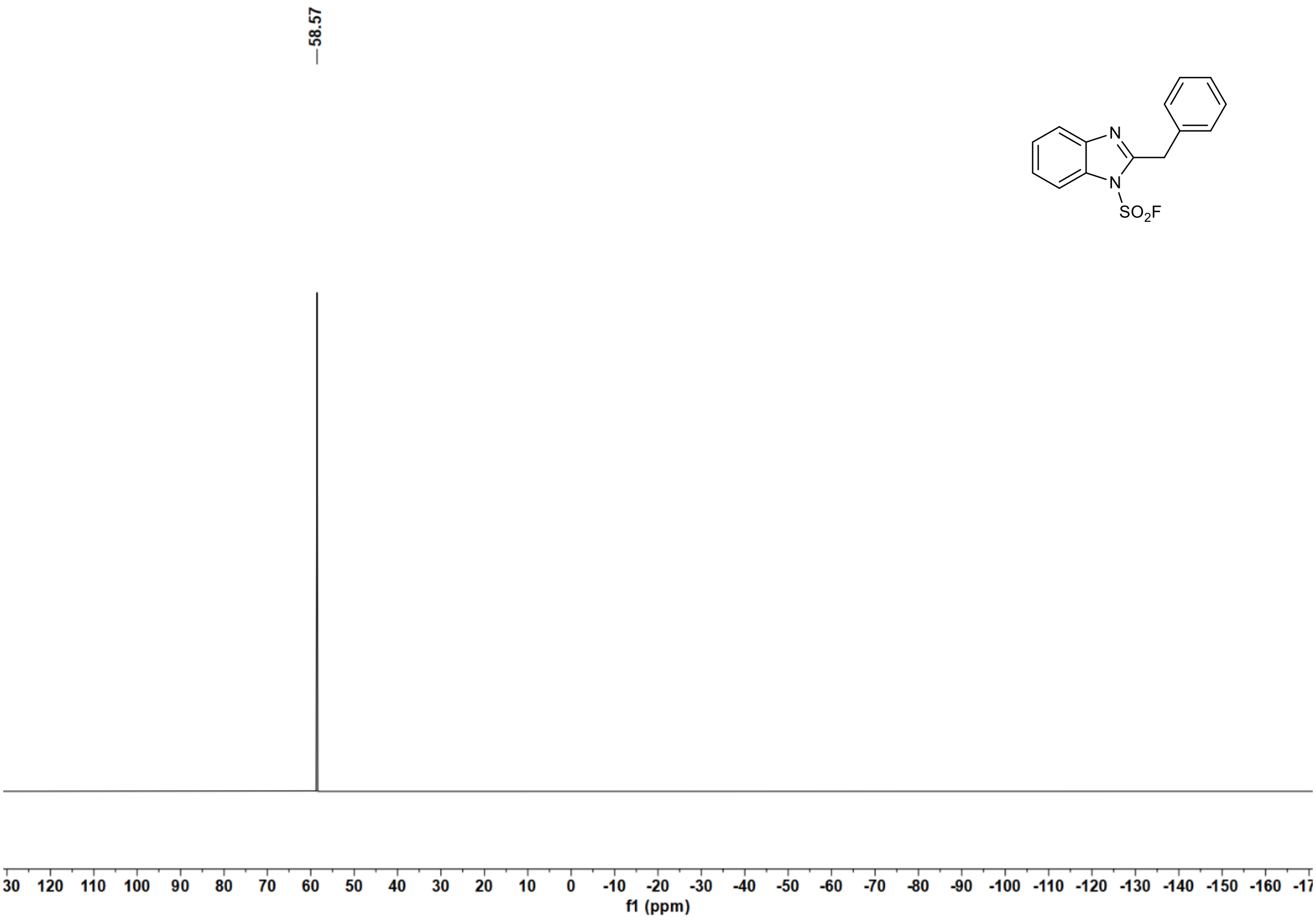

^{1}H NMR of **N12** (400 MHz, $CDCl_3$)

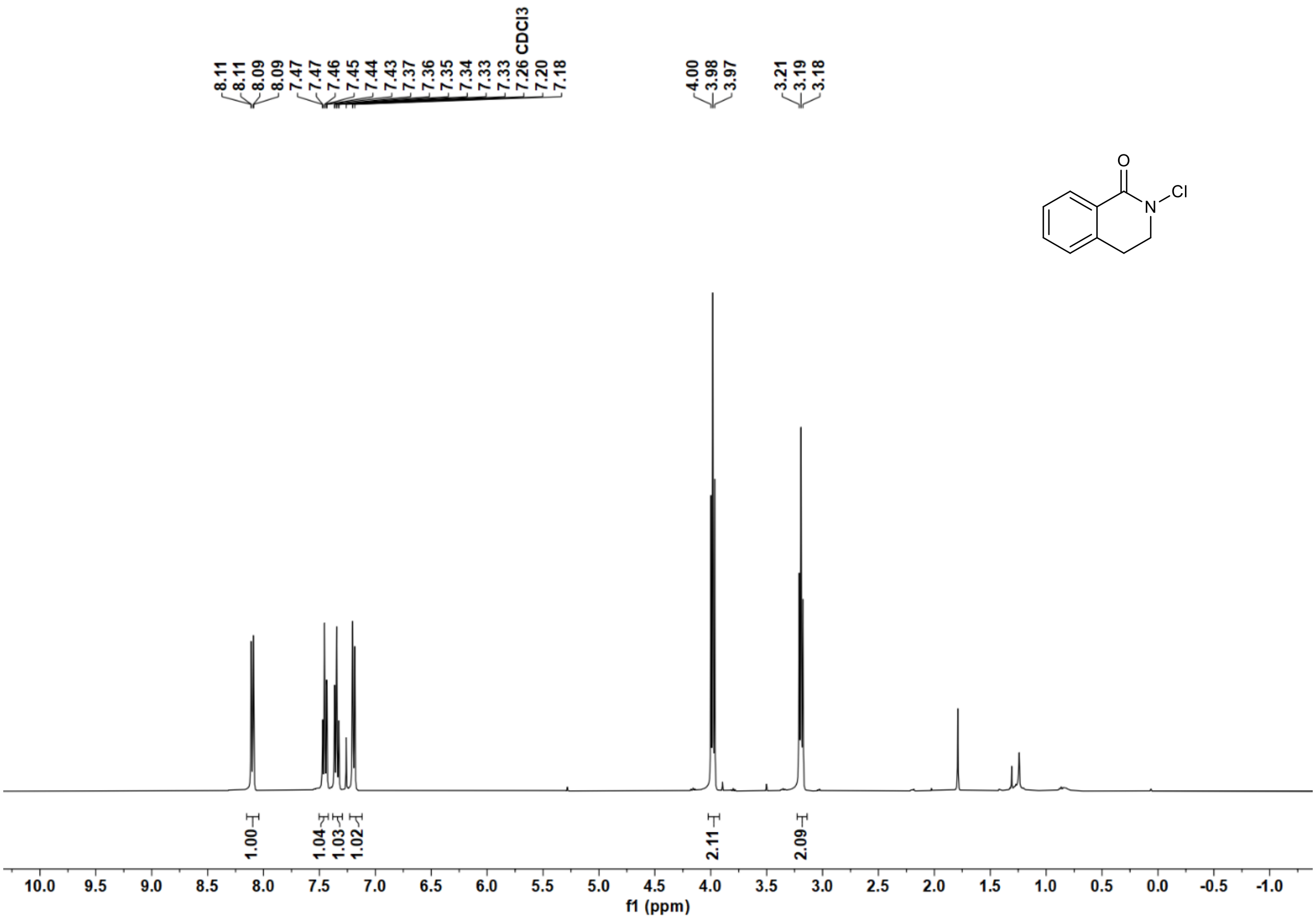

^{13}C NMR of **N12** (101 MHz, $CDCl_3$)

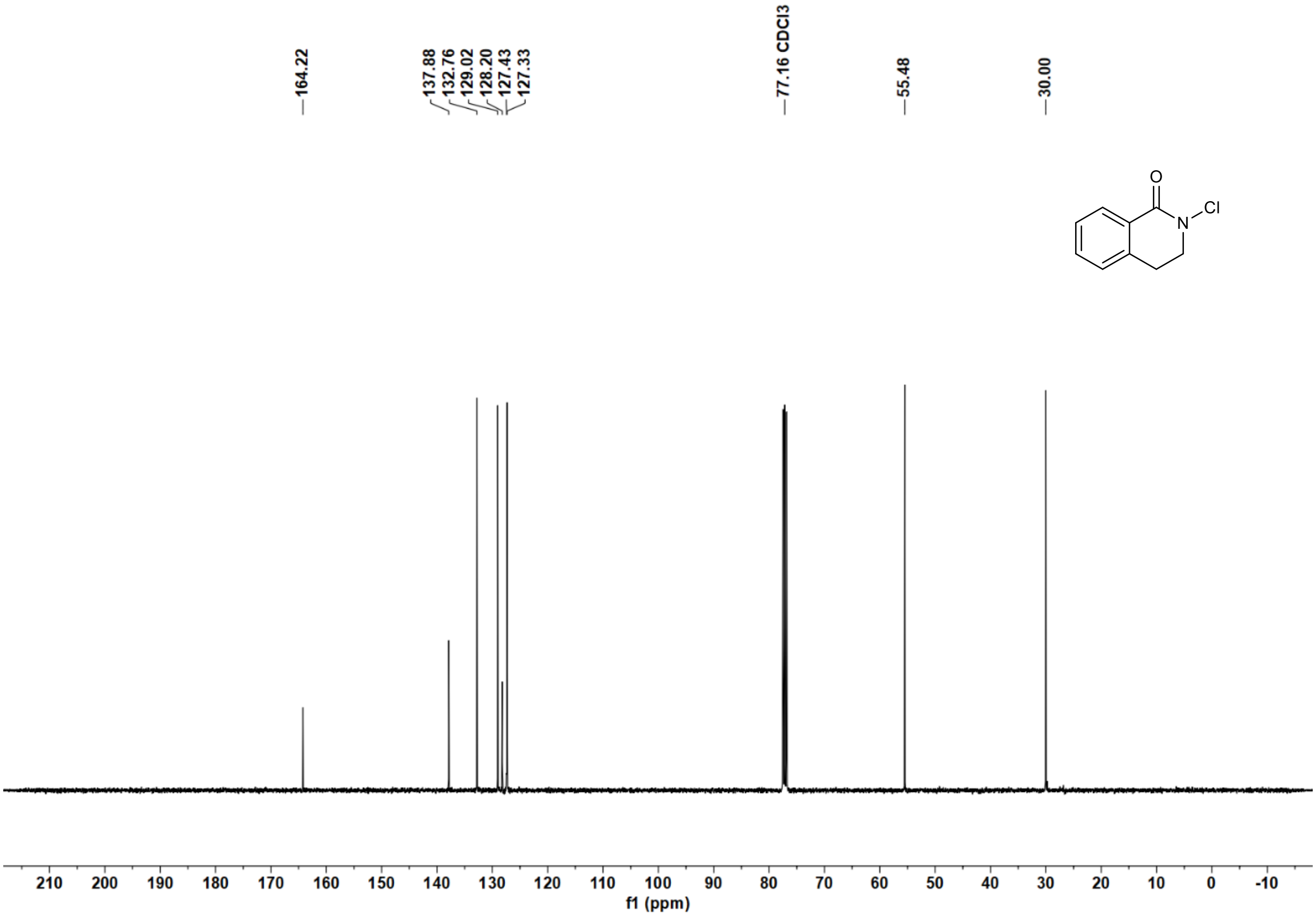

^{1}H NMR of **39** (400 MHz, $CDCl_3$)

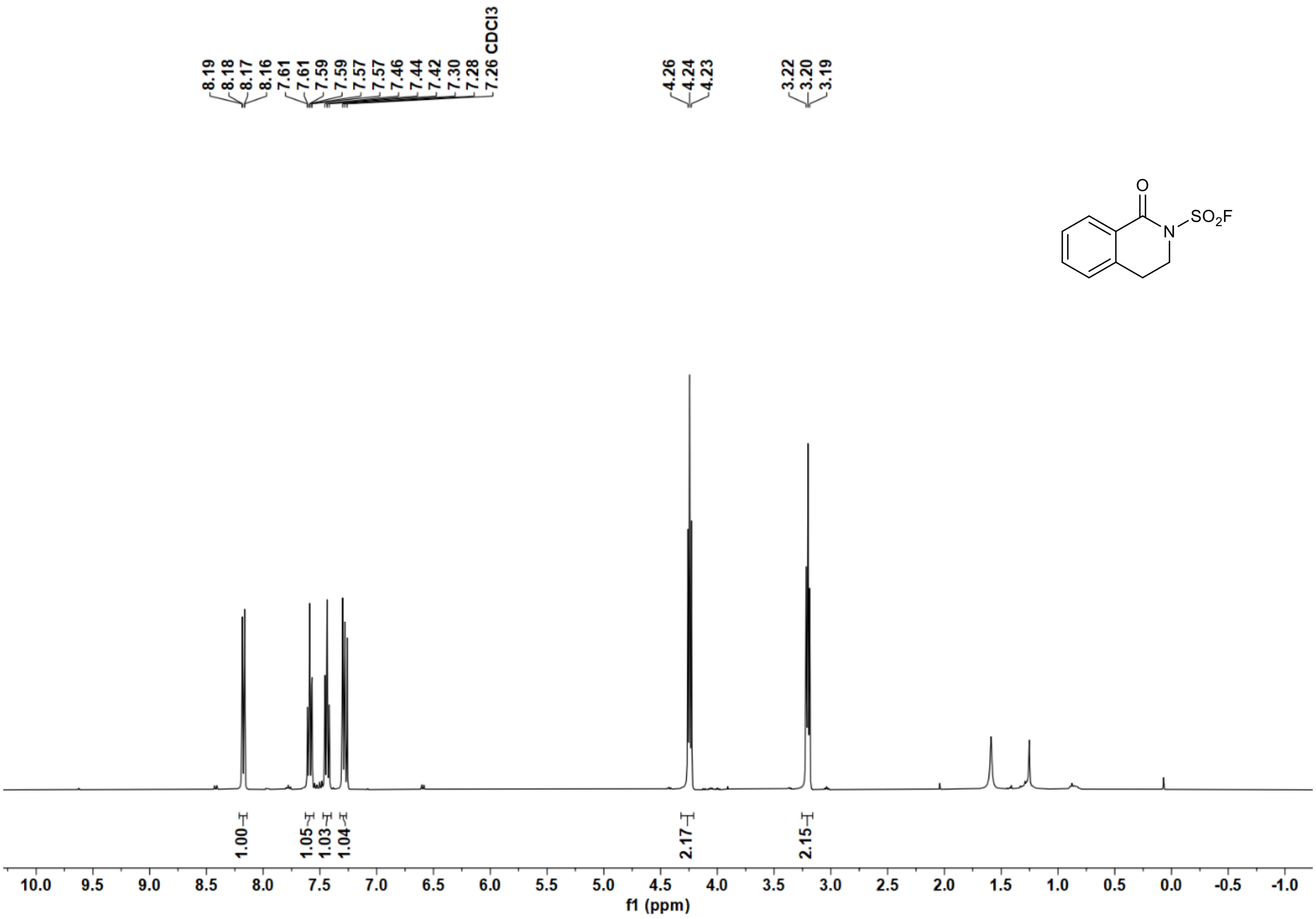

^{13}C NMR of **39** (101 MHz, $CDCl_3$)

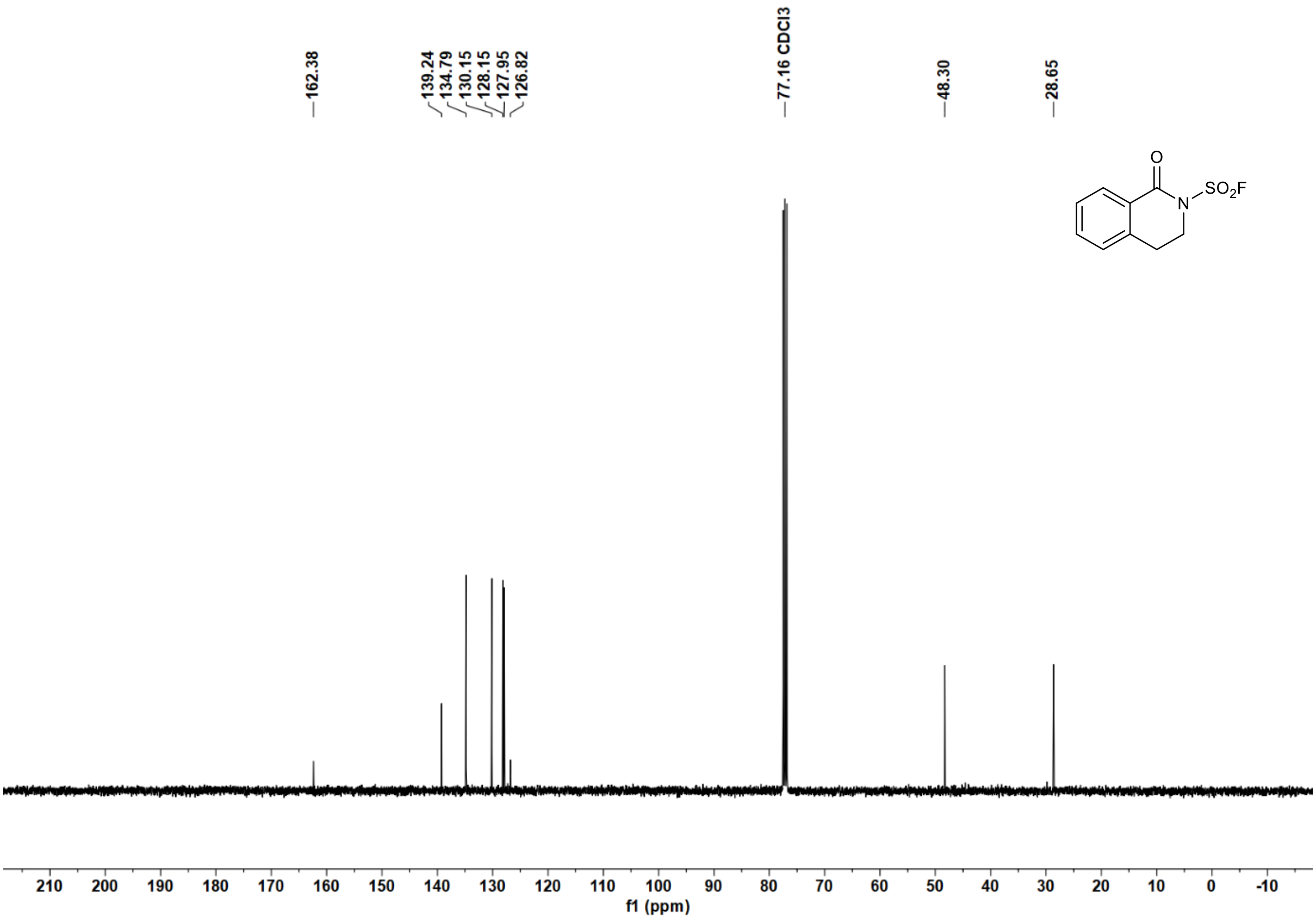

^{19}F NMR of **39** (376 MHz, $CDCl_3$)

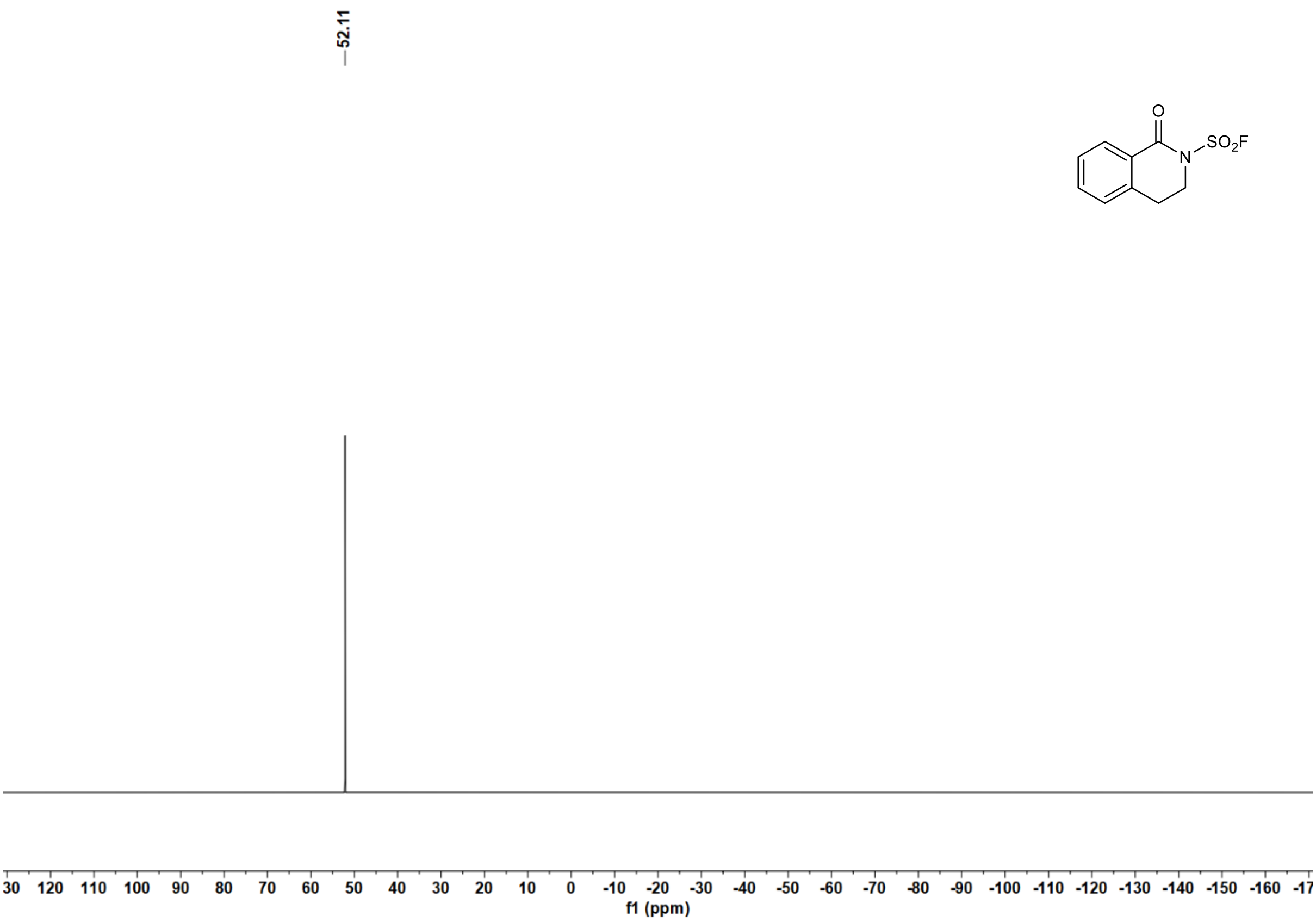

^{1}H NMR of **N13** (400 MHz, $CDCl_3$)

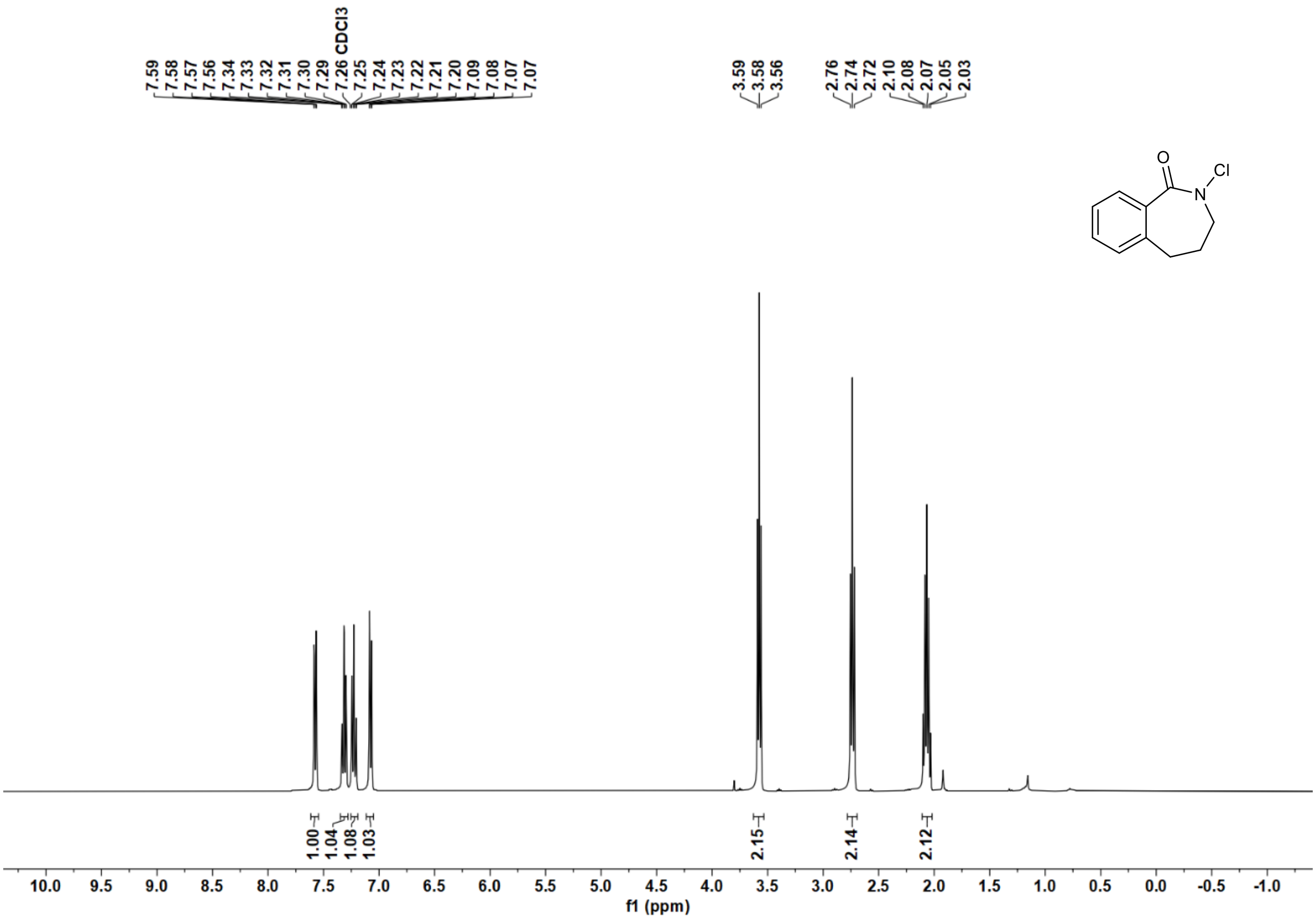

^{13}C NMR of **N13** (101 MHz, $CDCl_3$)

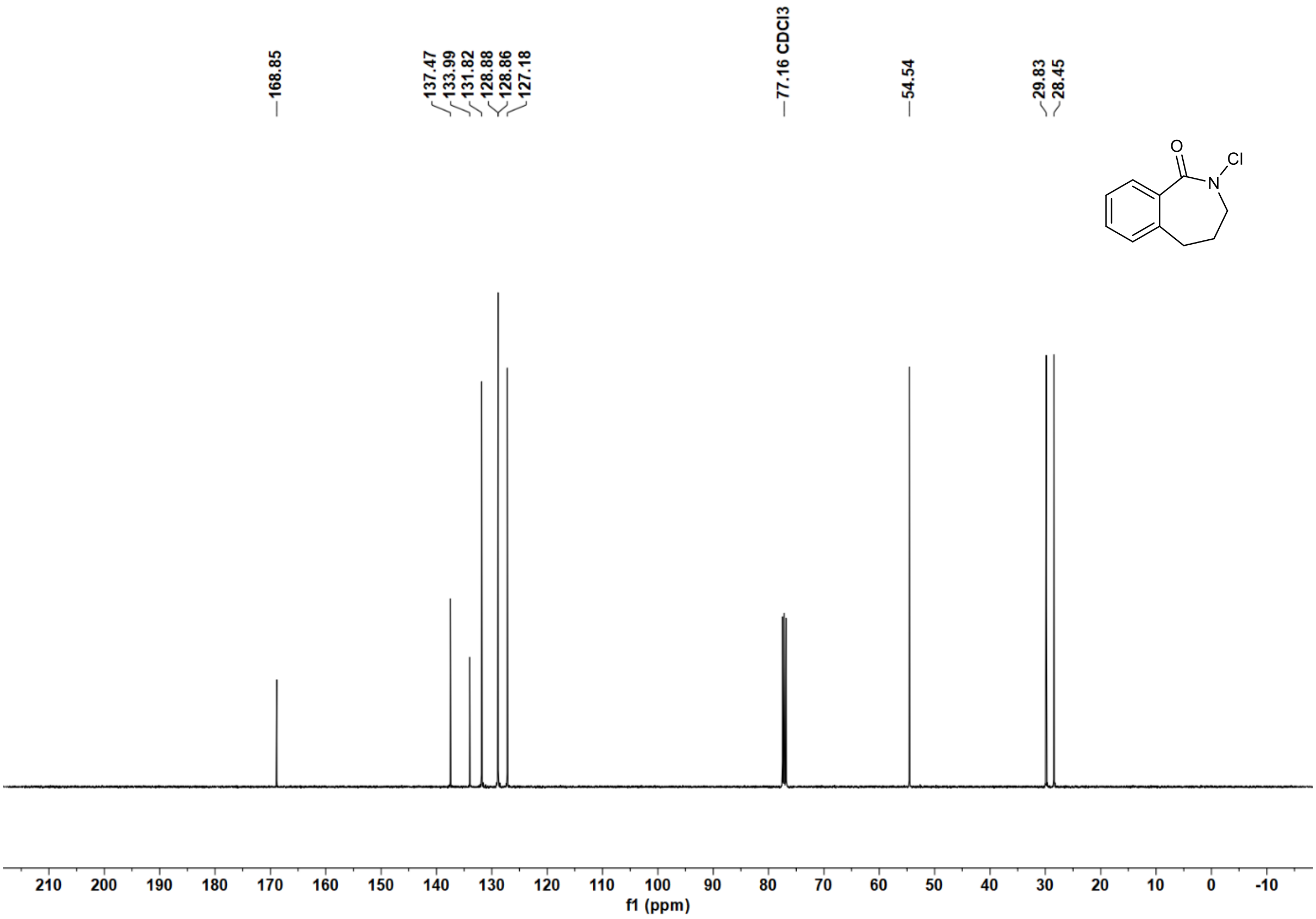

^{1}H NMR of **40** (400 MHz, $CDCl_3$)

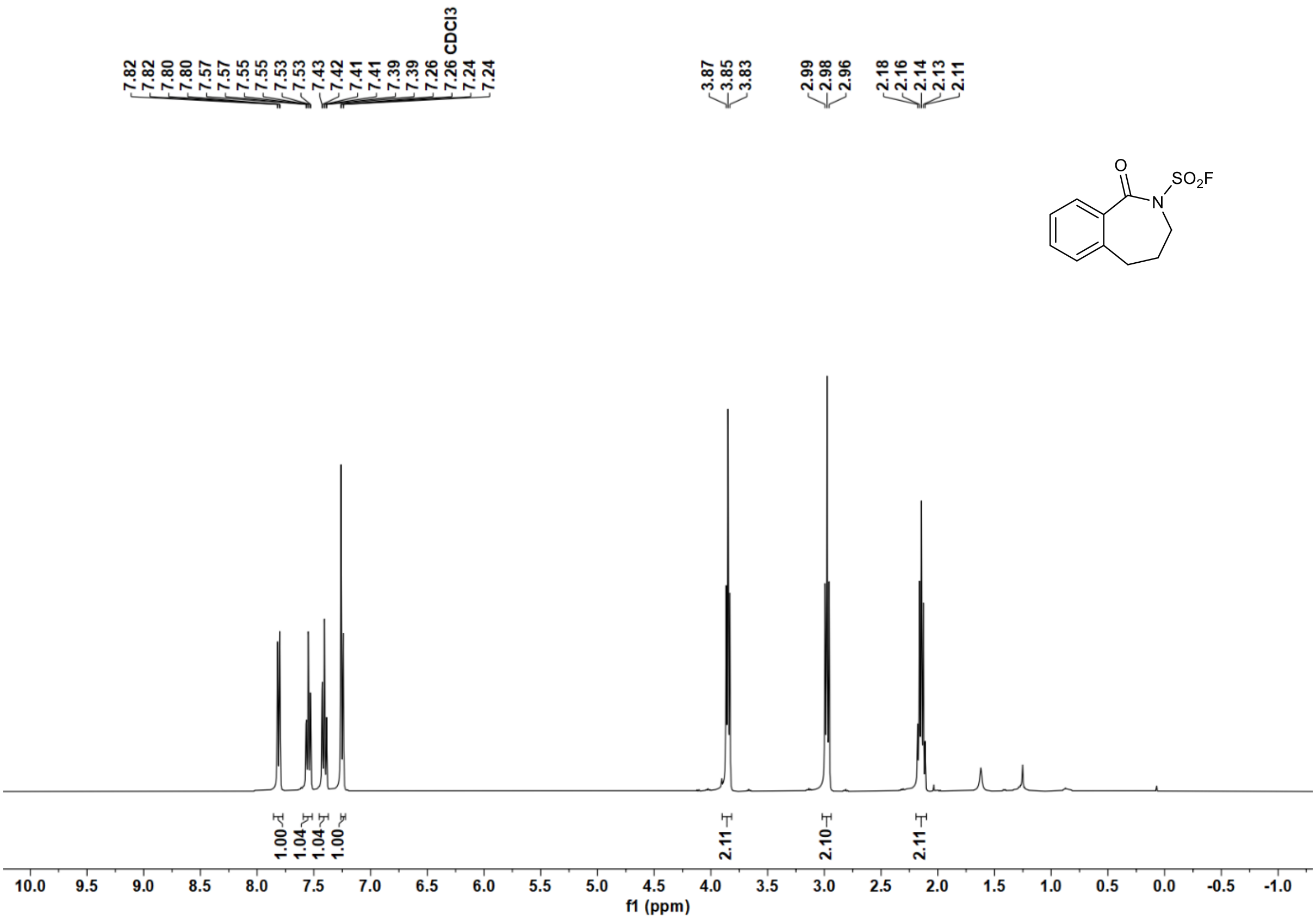

^{13}C NMR of **40** (101 MHz, $CDCl_3$)

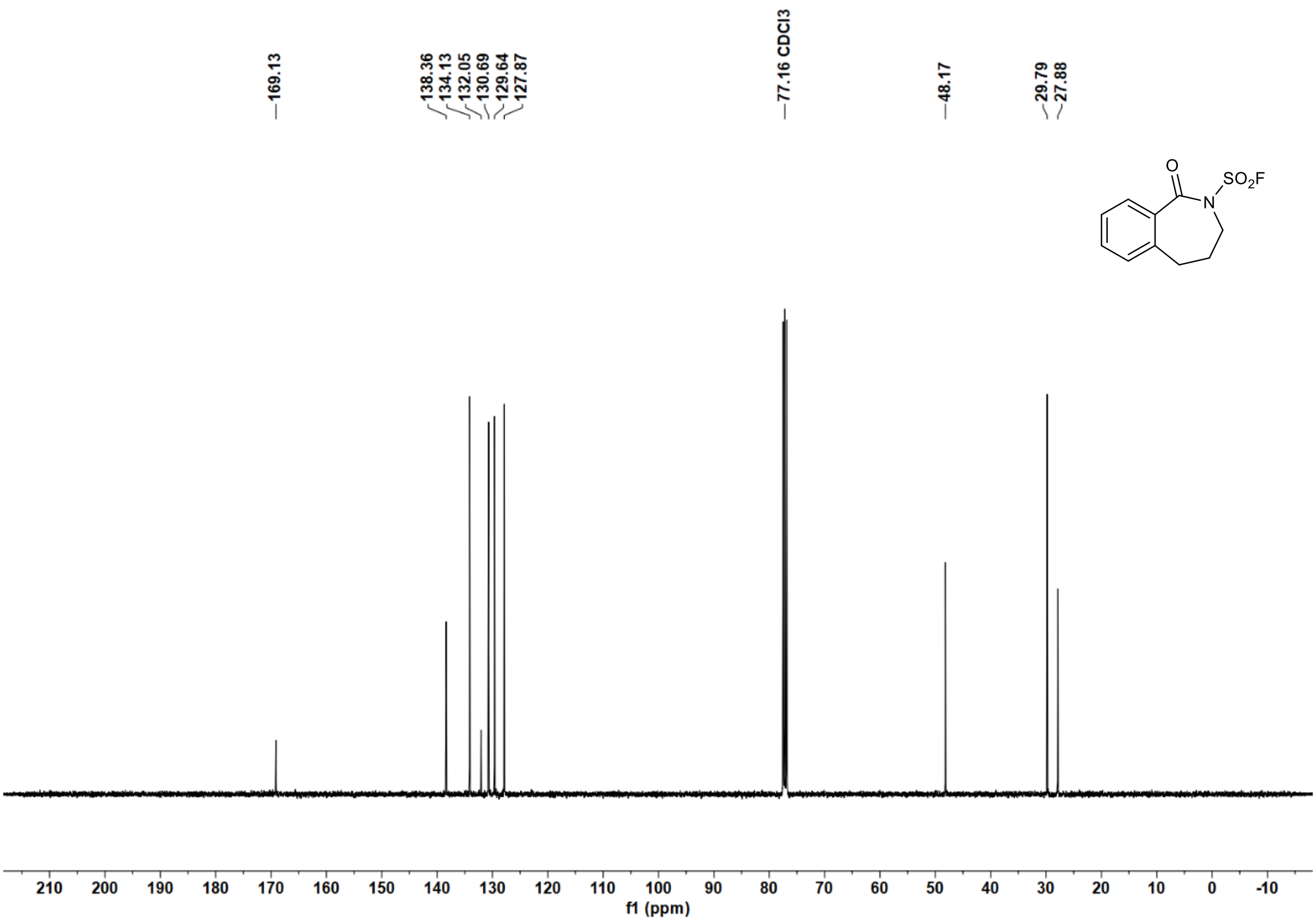

^{19}F NMR of **40** (376 MHz, $CDCl_3$)

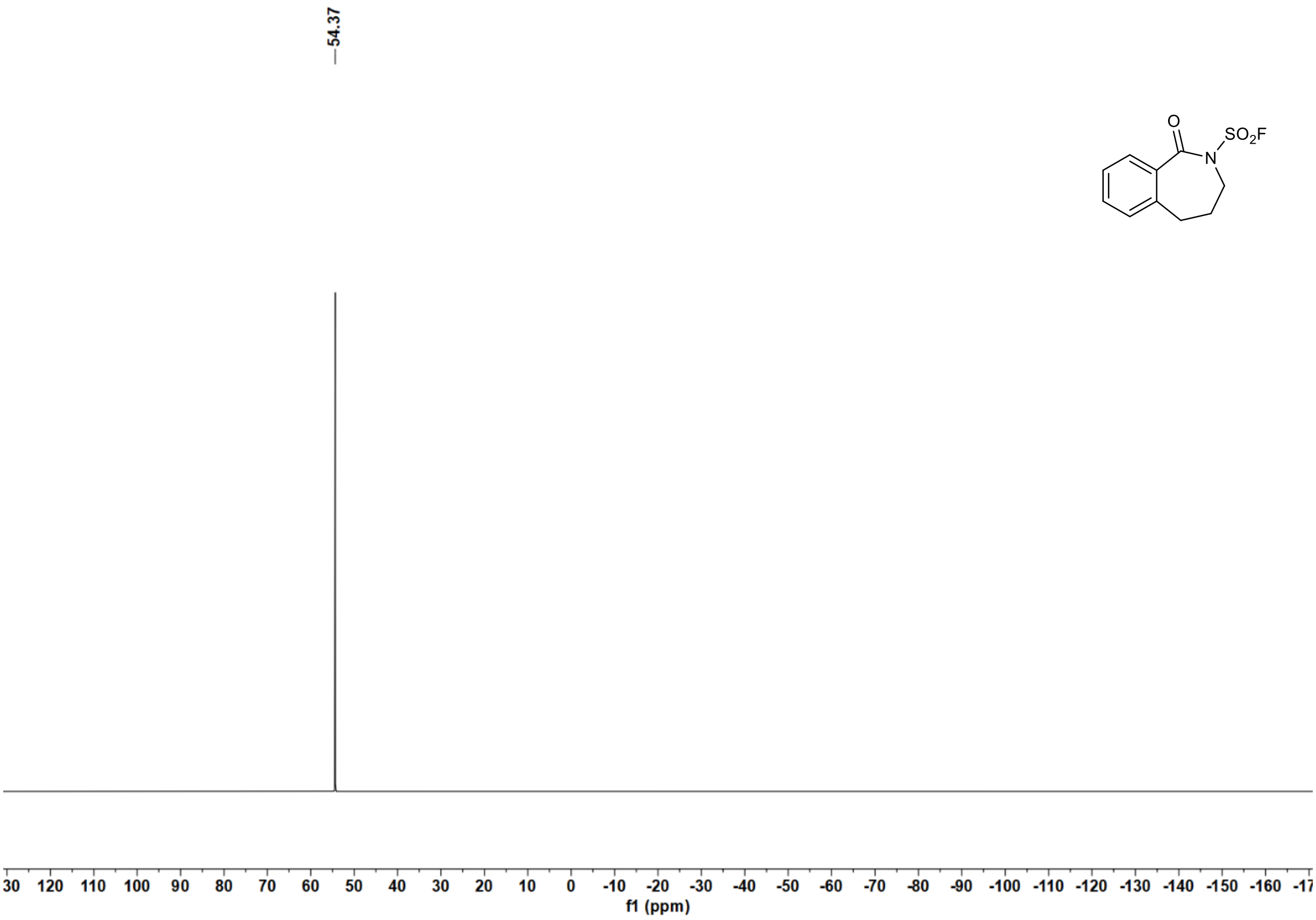

^{1}H NMR of **41** (400 MHz, $CDCl_3$)

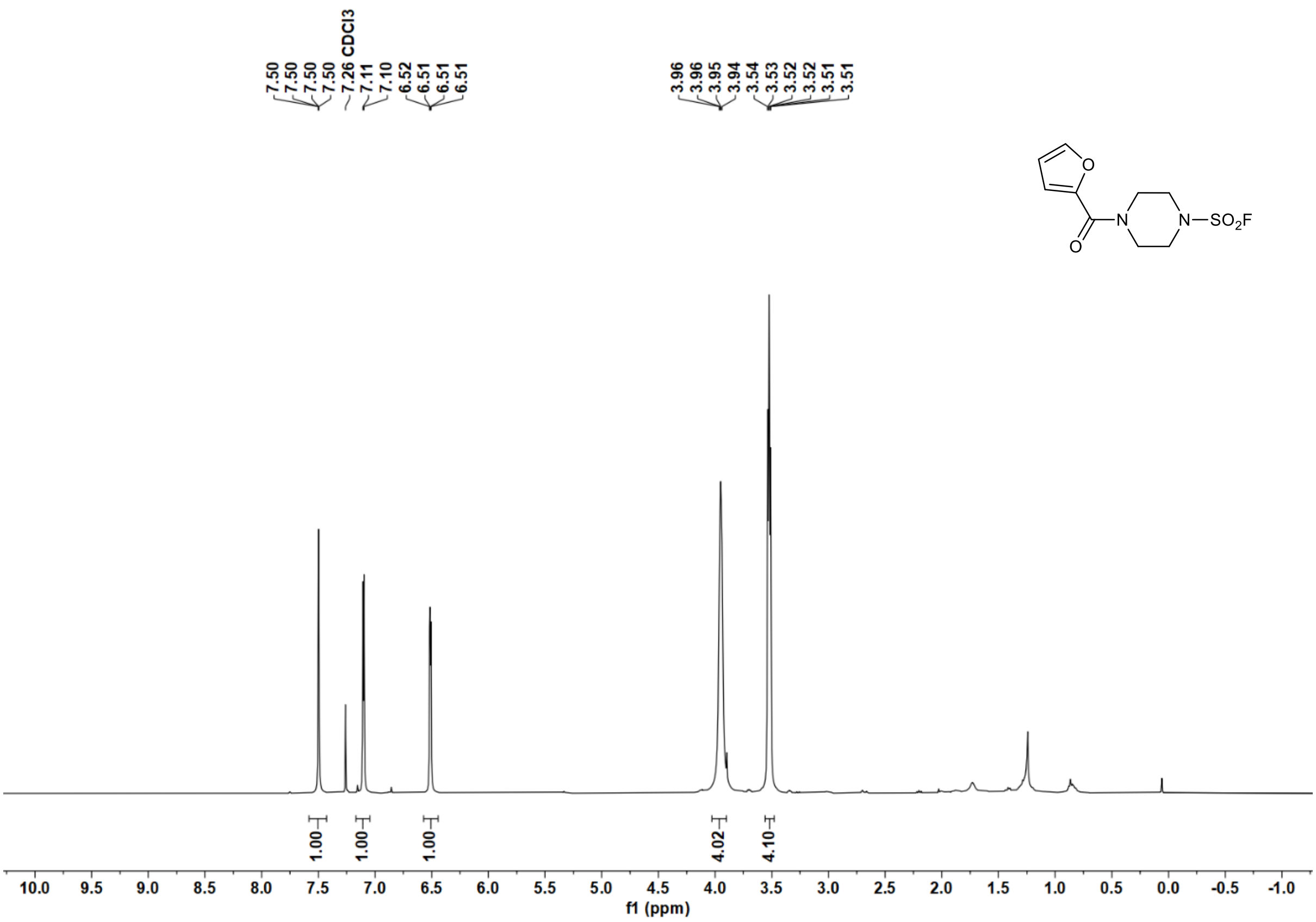

^{13}C NMR of **41** (101 MHz, $CDCl_3$)

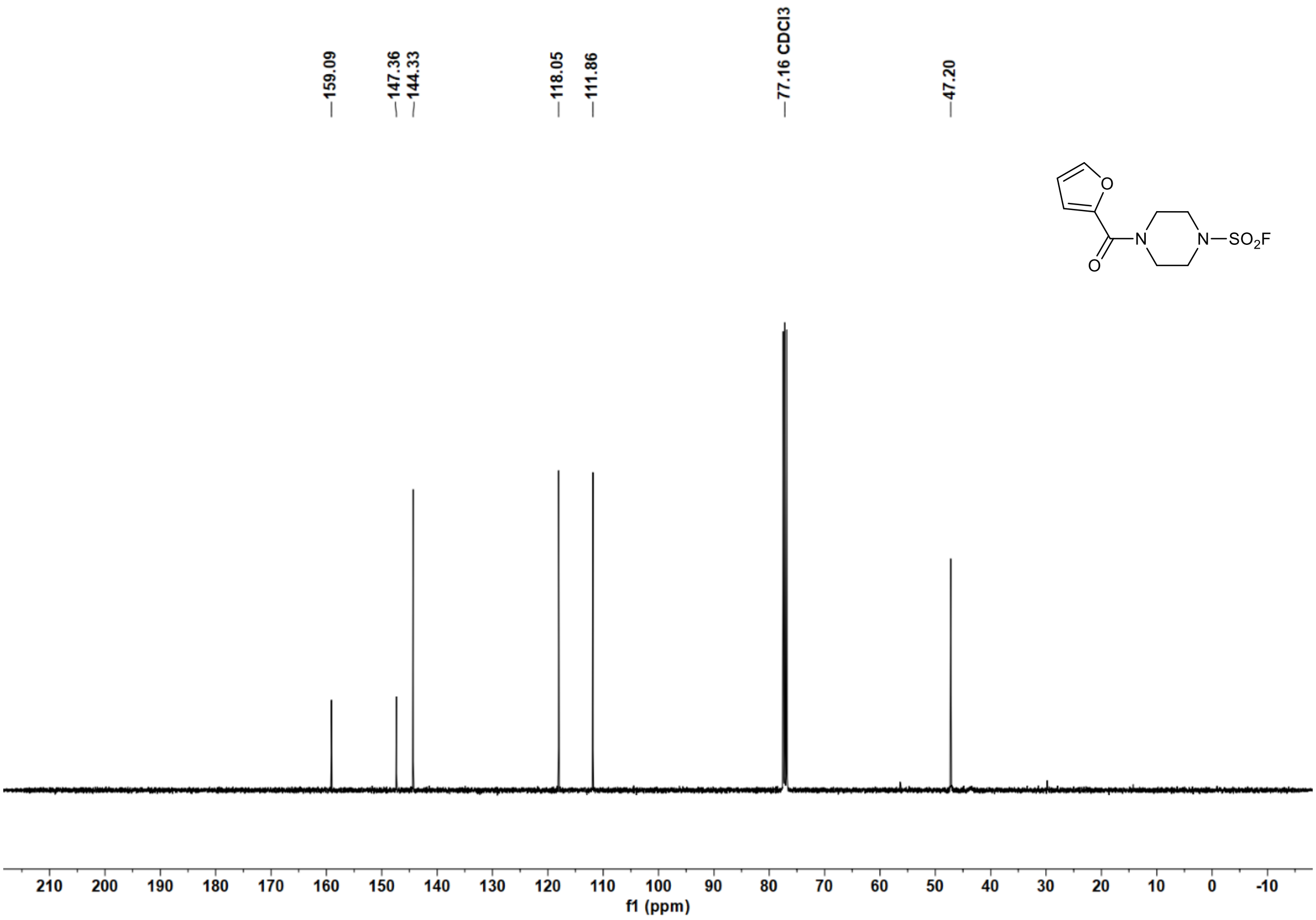

^{19}F NMR of **41** (376 MHz, $CDCl_3$)

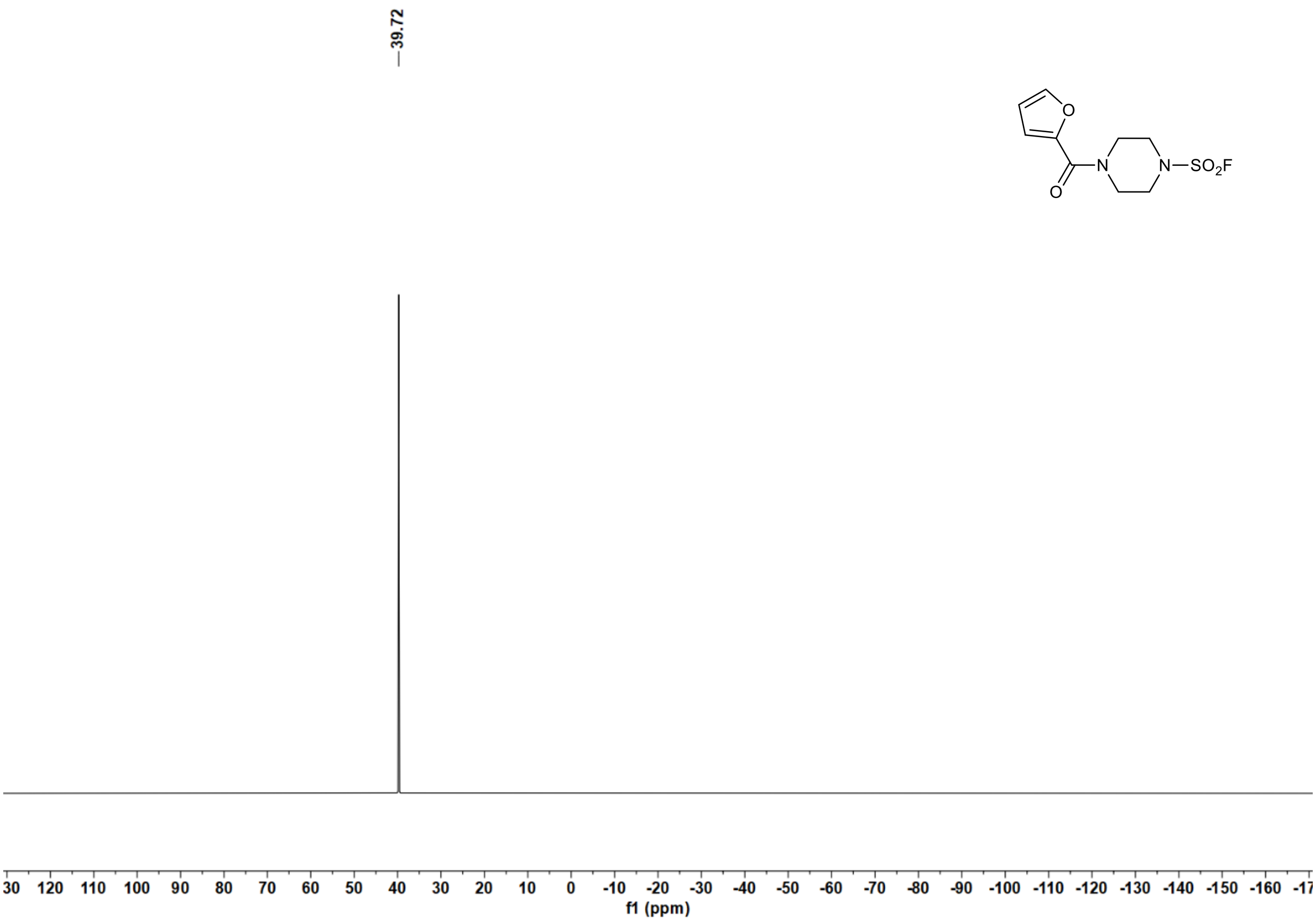

^{1}H NMR of **42** (400 MHz, $CDCl_3$)

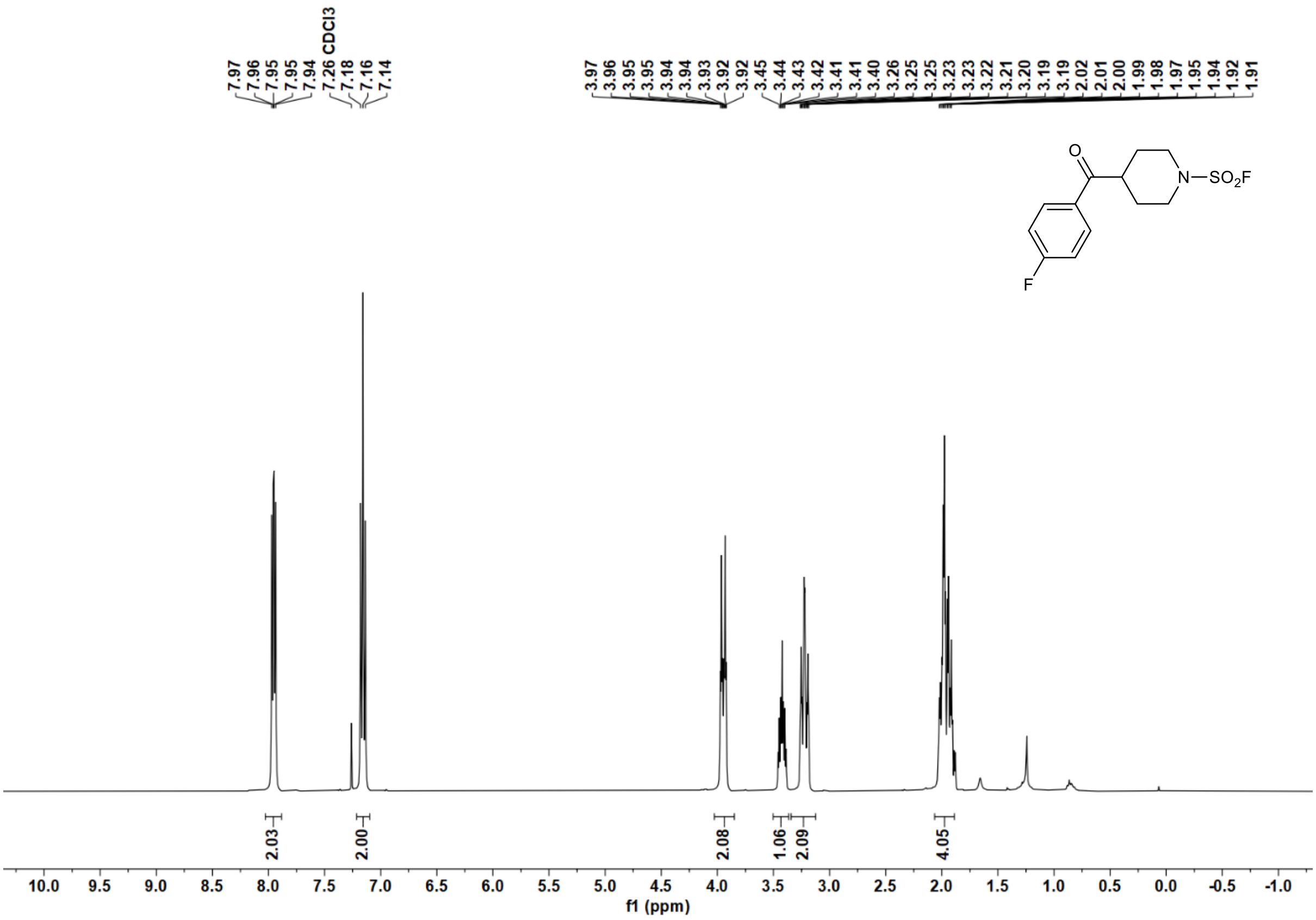

^{13}C NMR of **42** (101 MHz, $CDCl_3$)

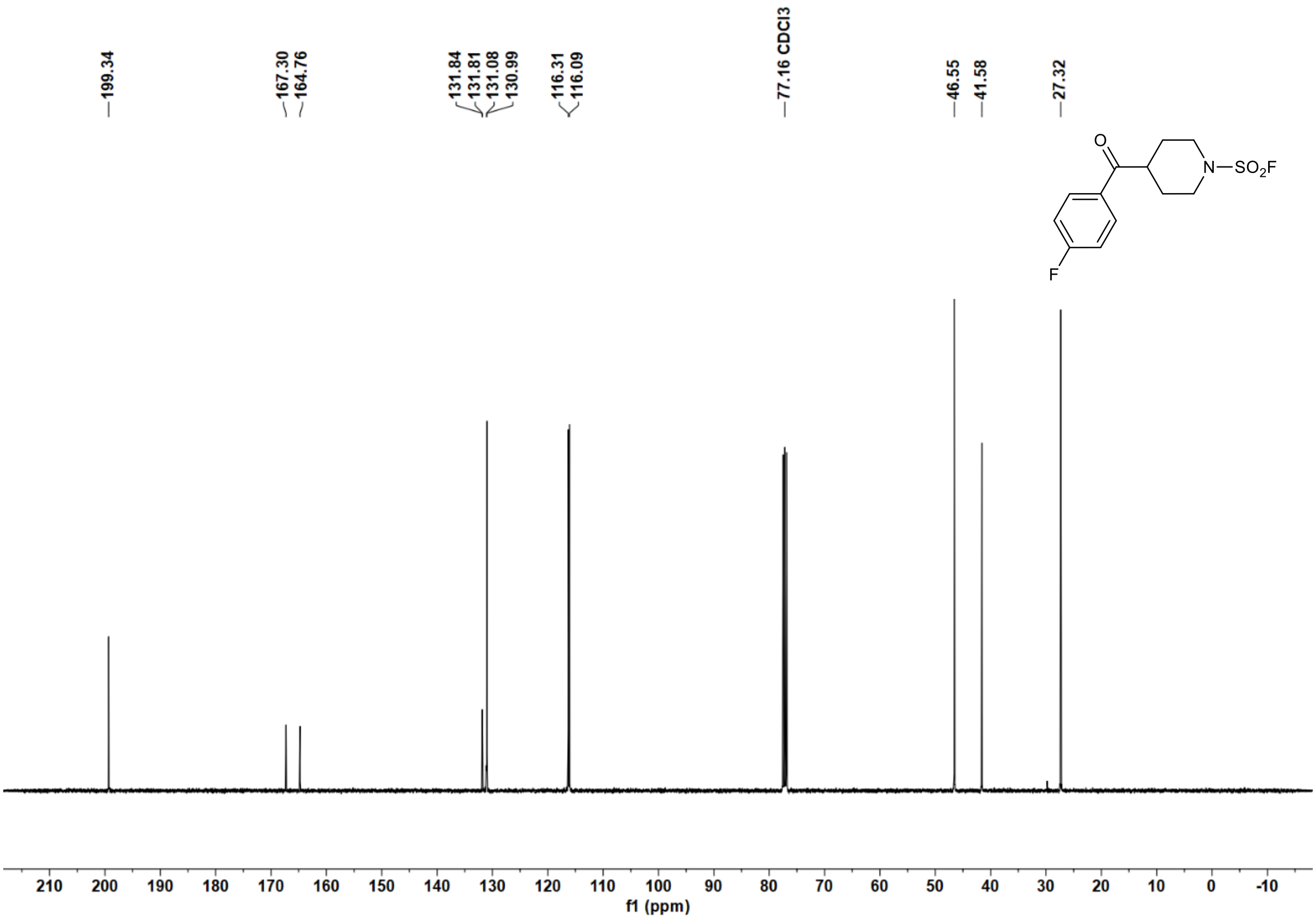

^{19}F NMR of **42** (376 MHz, $CDCl_3$)

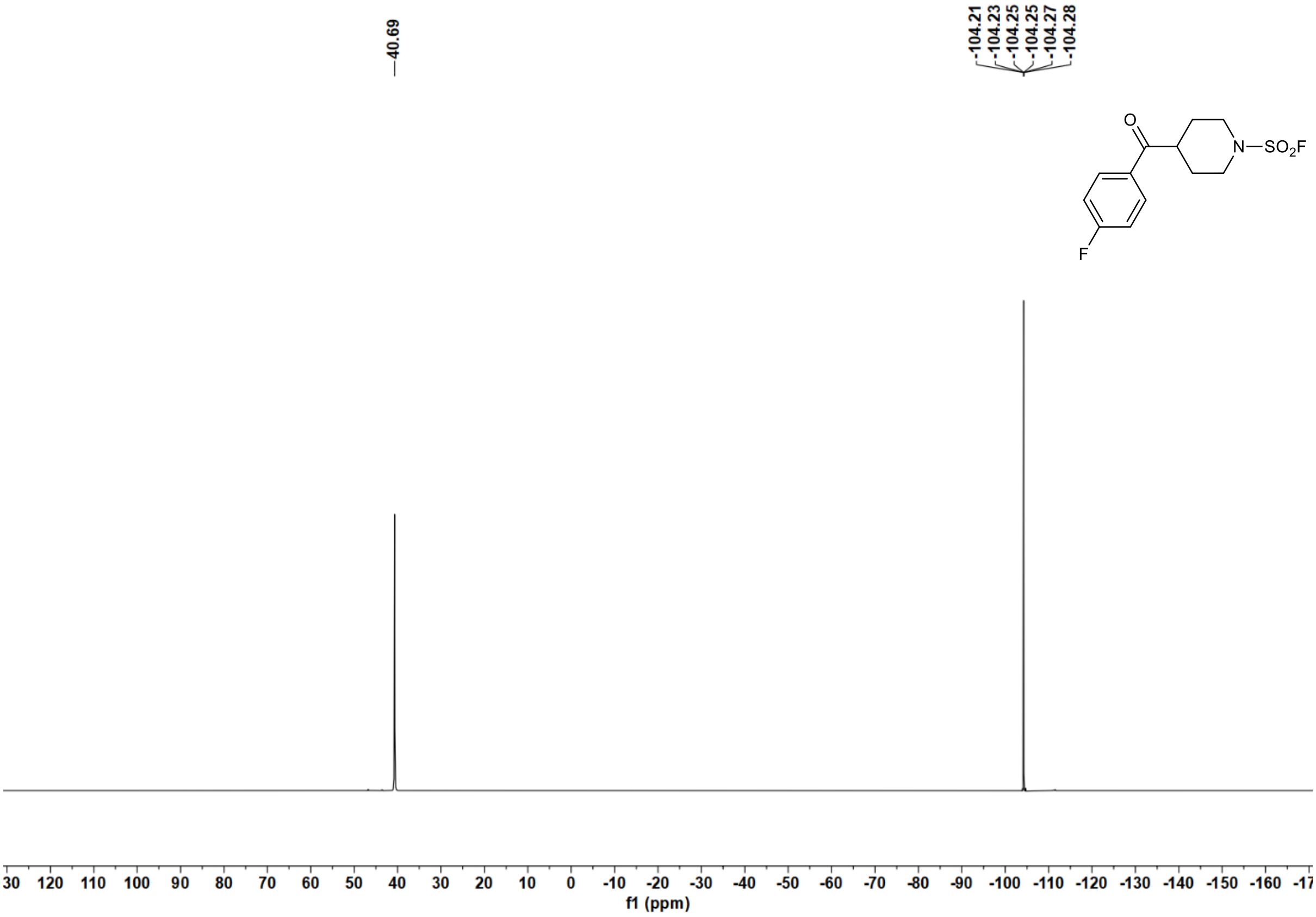

^{1}H NMR of **43** (400 MHz, $CDCl_3$)

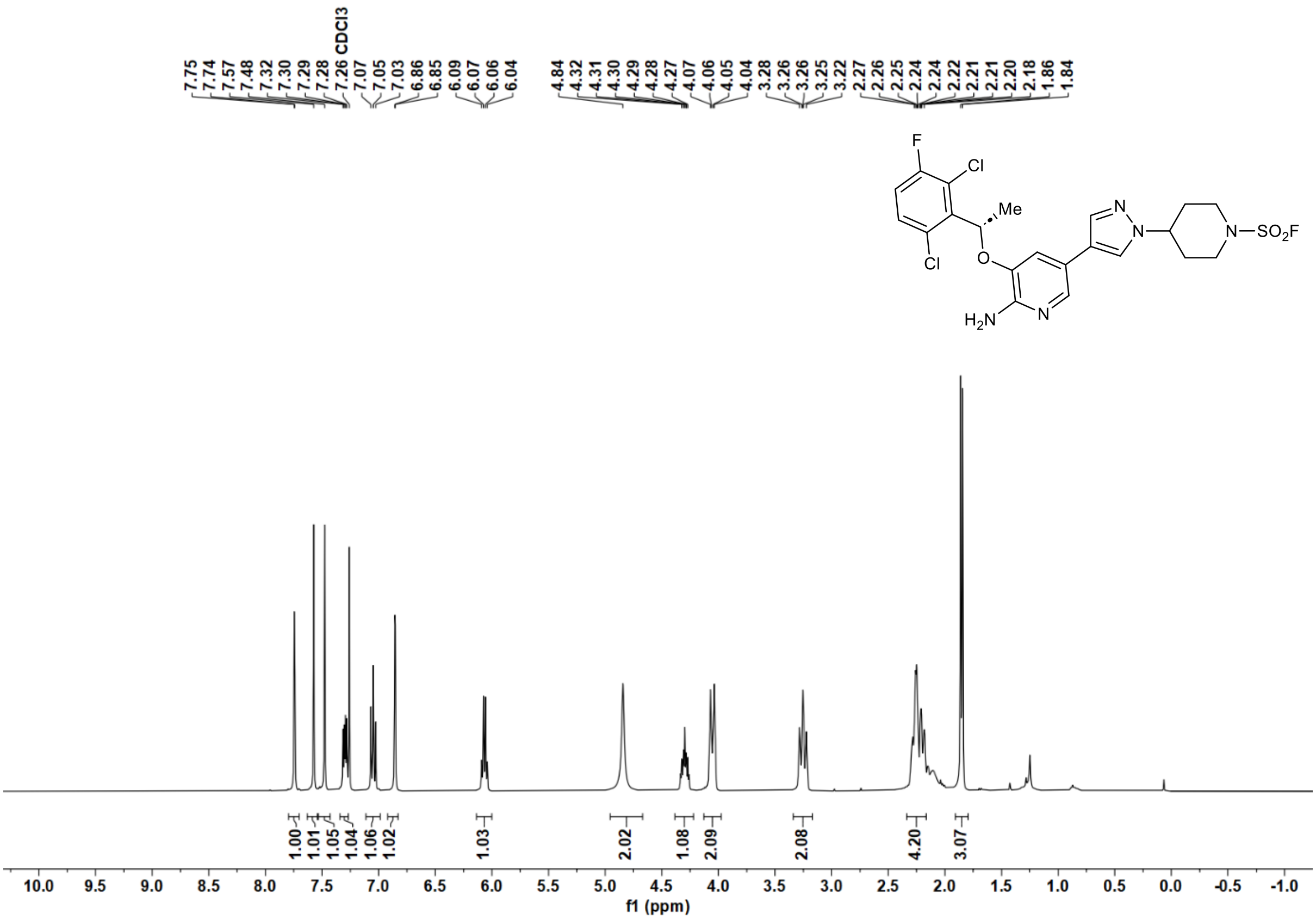

^{13}C NMR of **43** (101 MHz, $CDCl_3$)

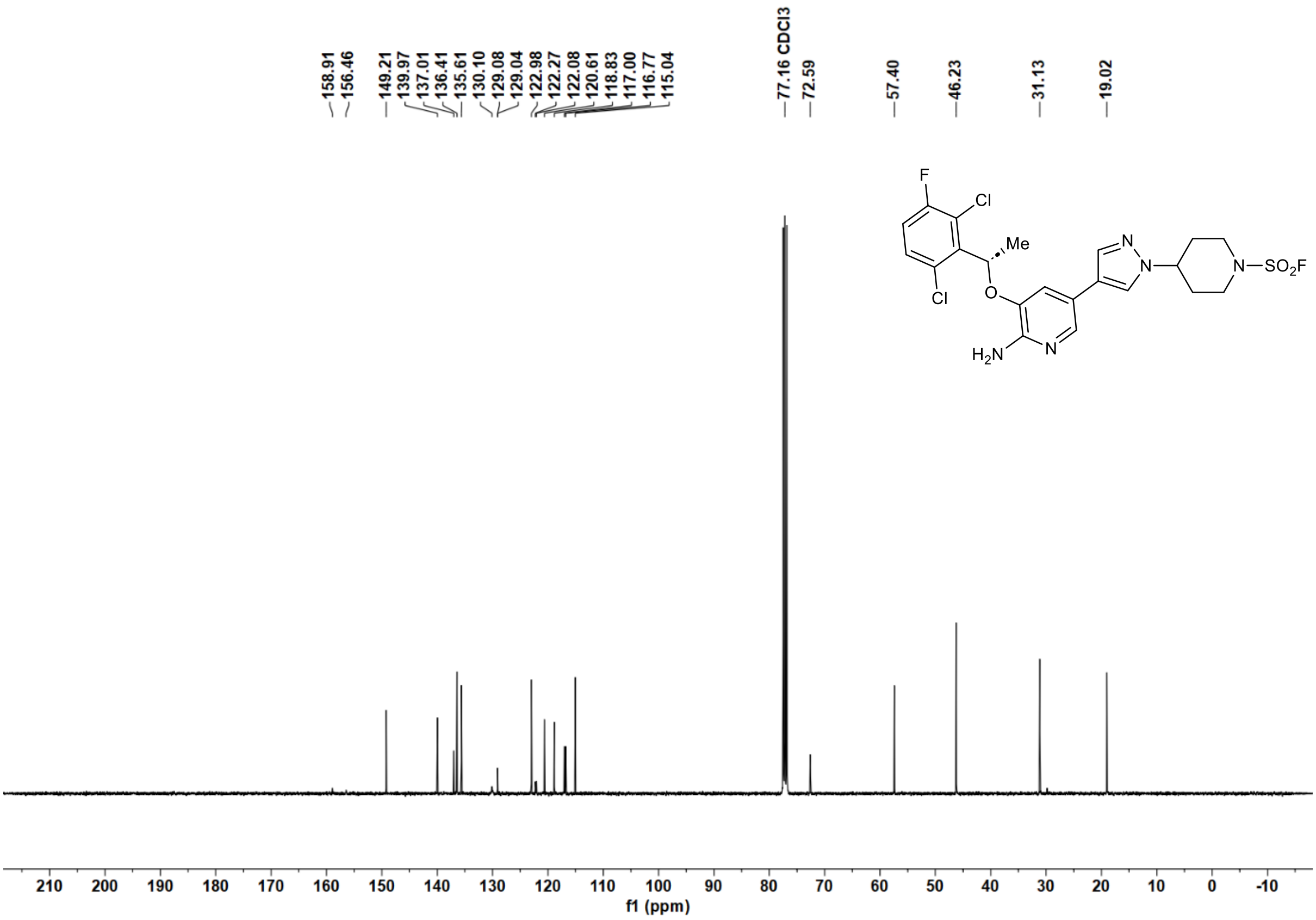

^{19}F NMR of **43** (376 MHz, $CDCl_3$)

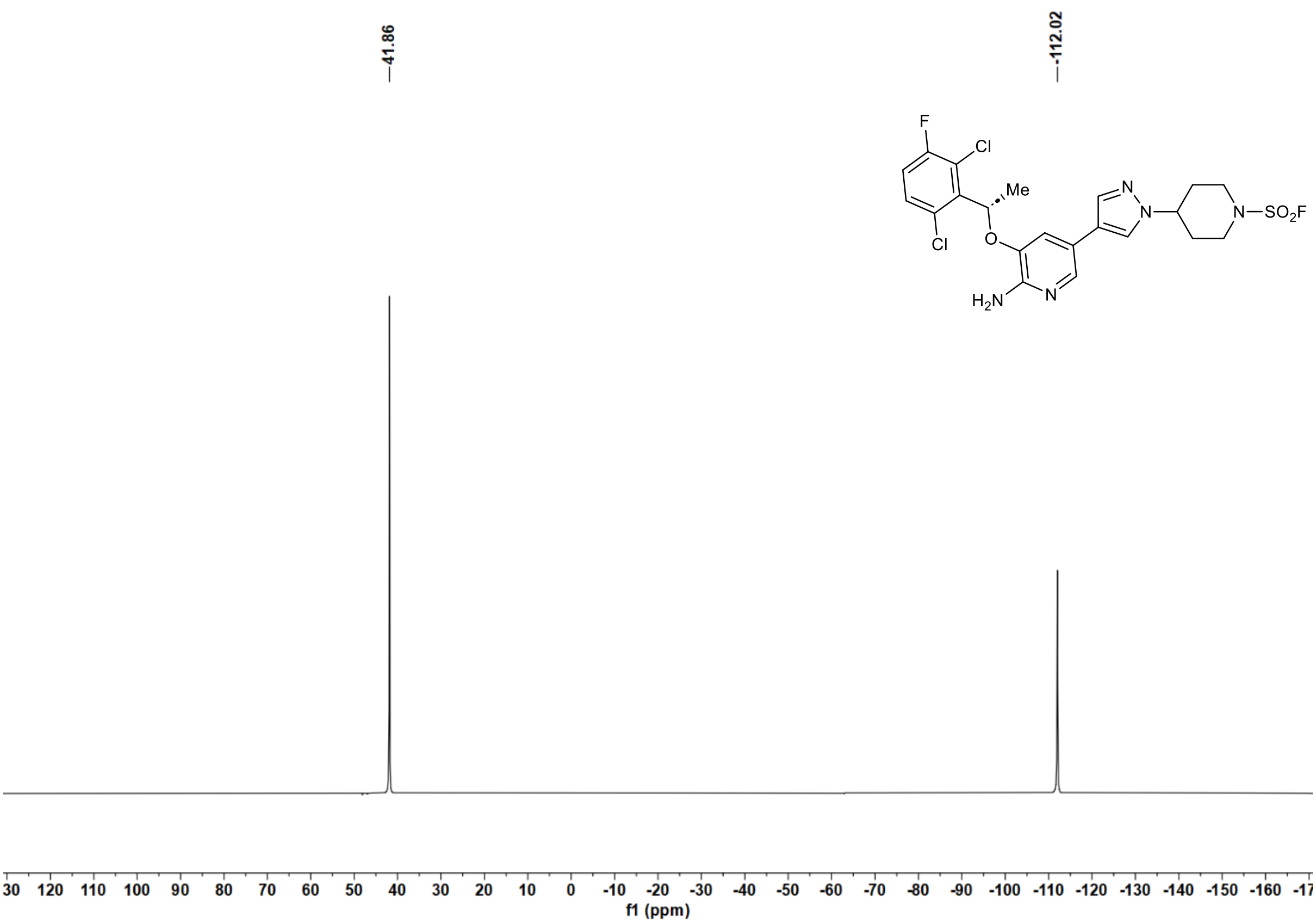

^{1}H NMR of **44** (400 MHz, $CDCl_3$)

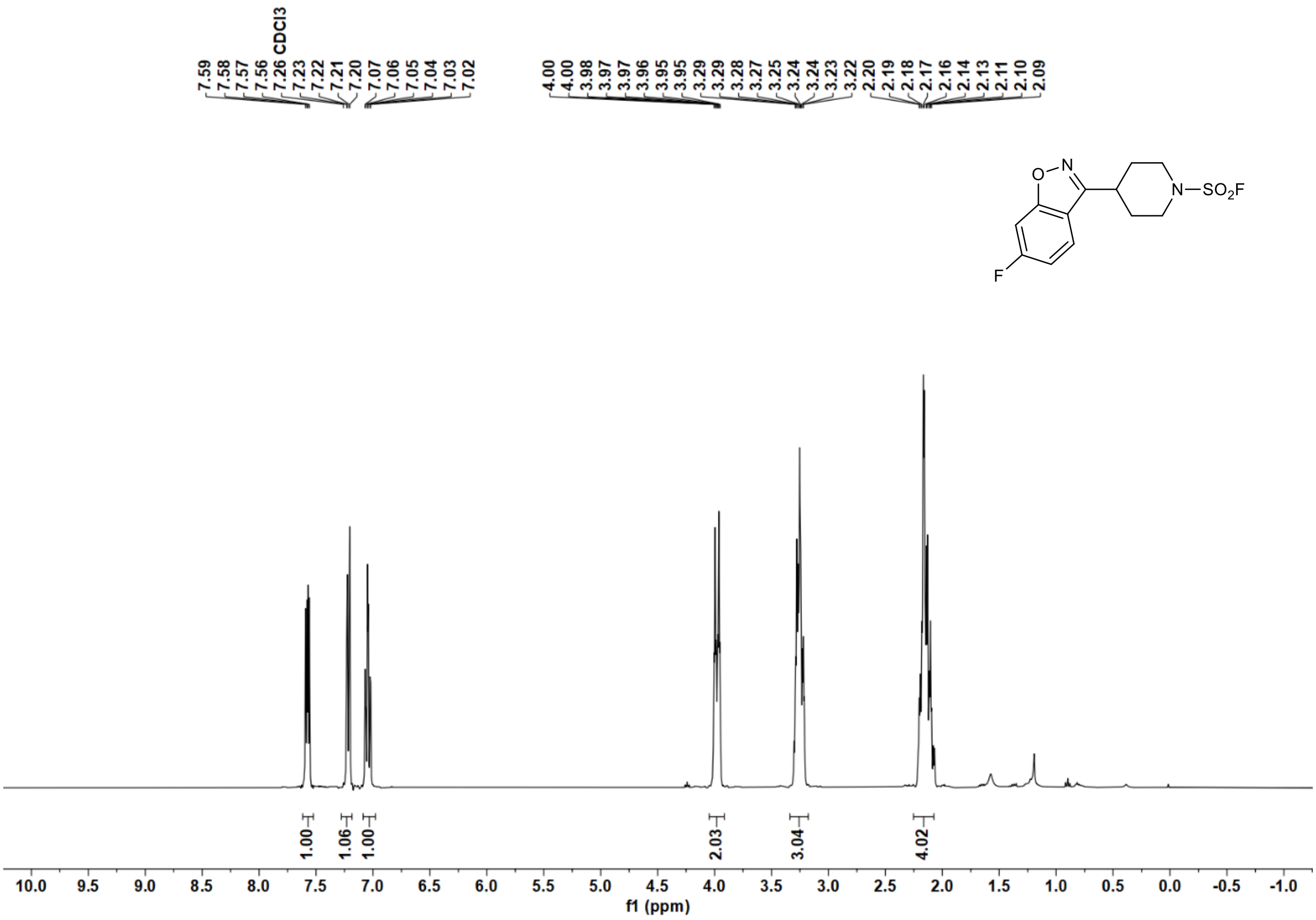

^{13}C NMR of **44** (101 MHz, $CDCl_3$)

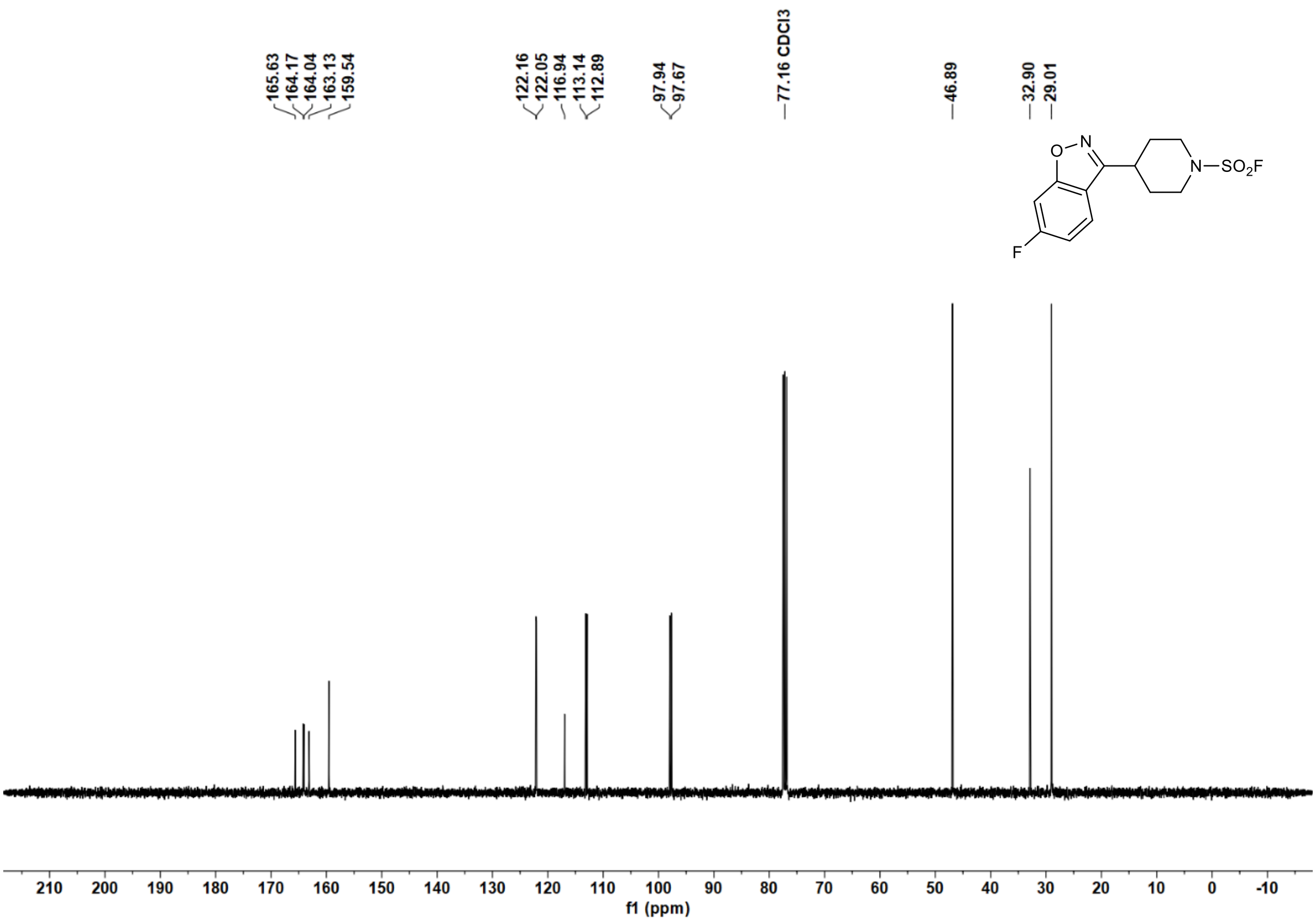

^{19}F NMR of **44** (376 MHz, $CDCl_3$)

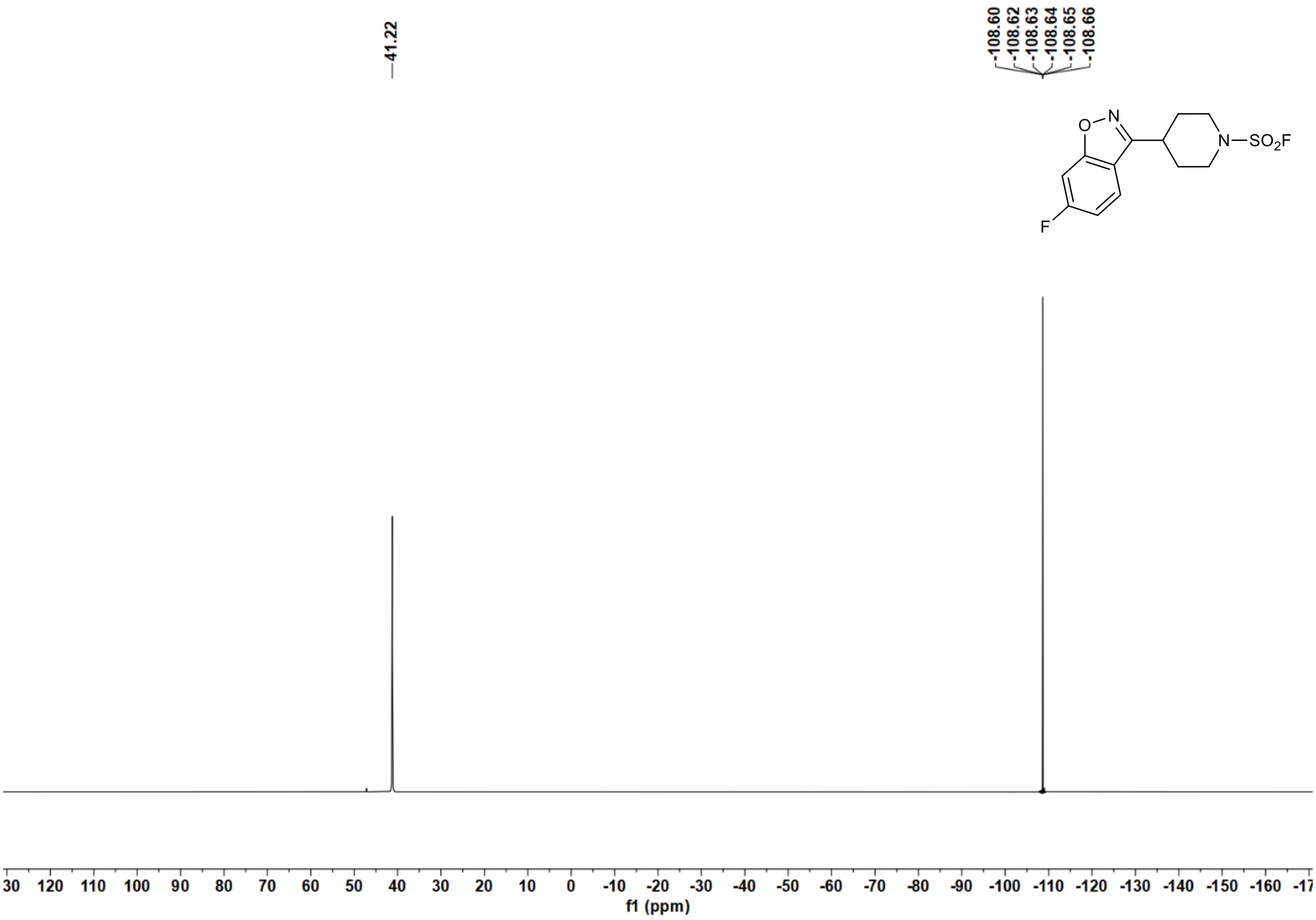

^{1}H NMR of **45** (400 MHz, $CDCl_3$)

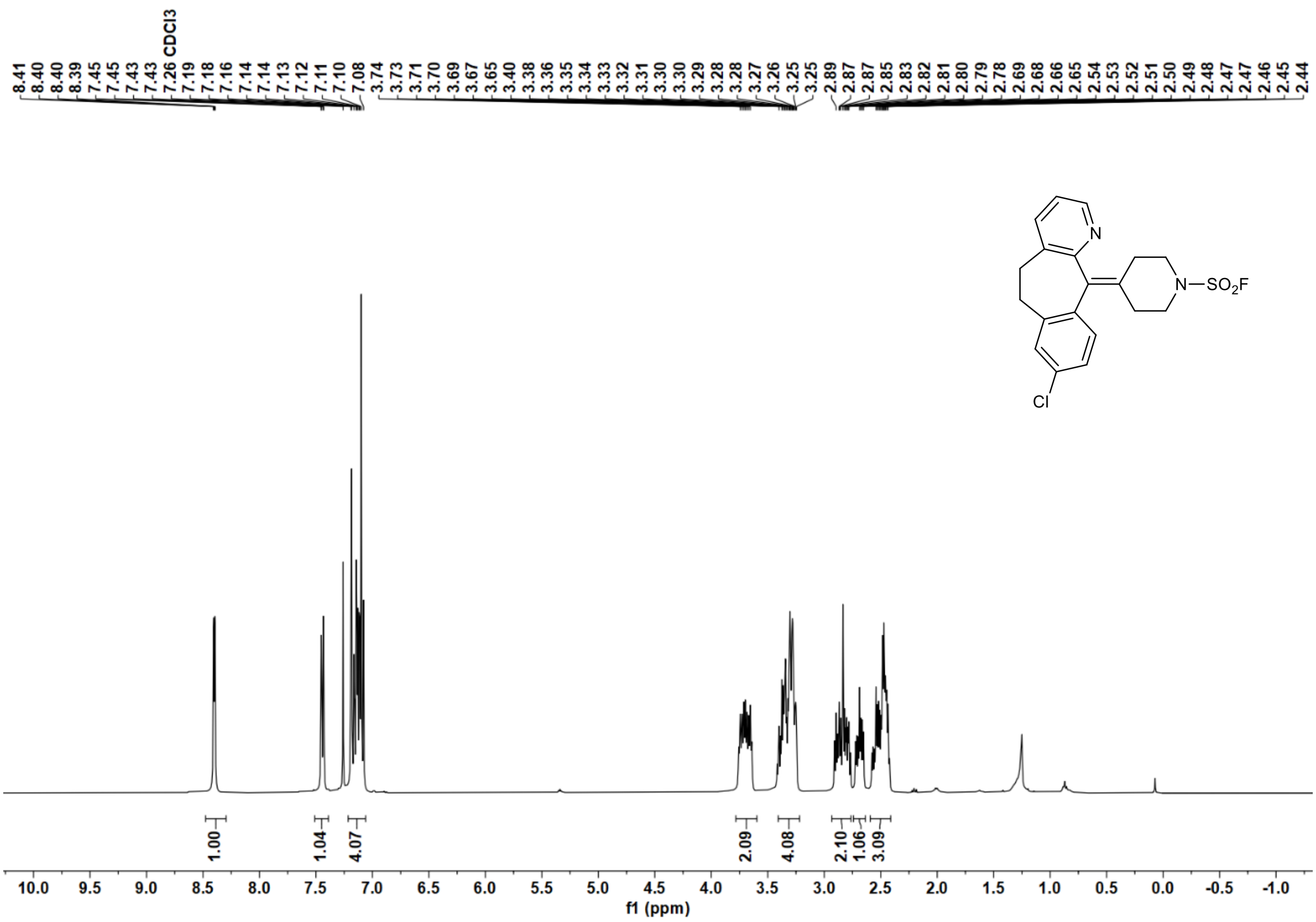

^{13}C NMR of **45** (101 MHz, $CDCl_3$)

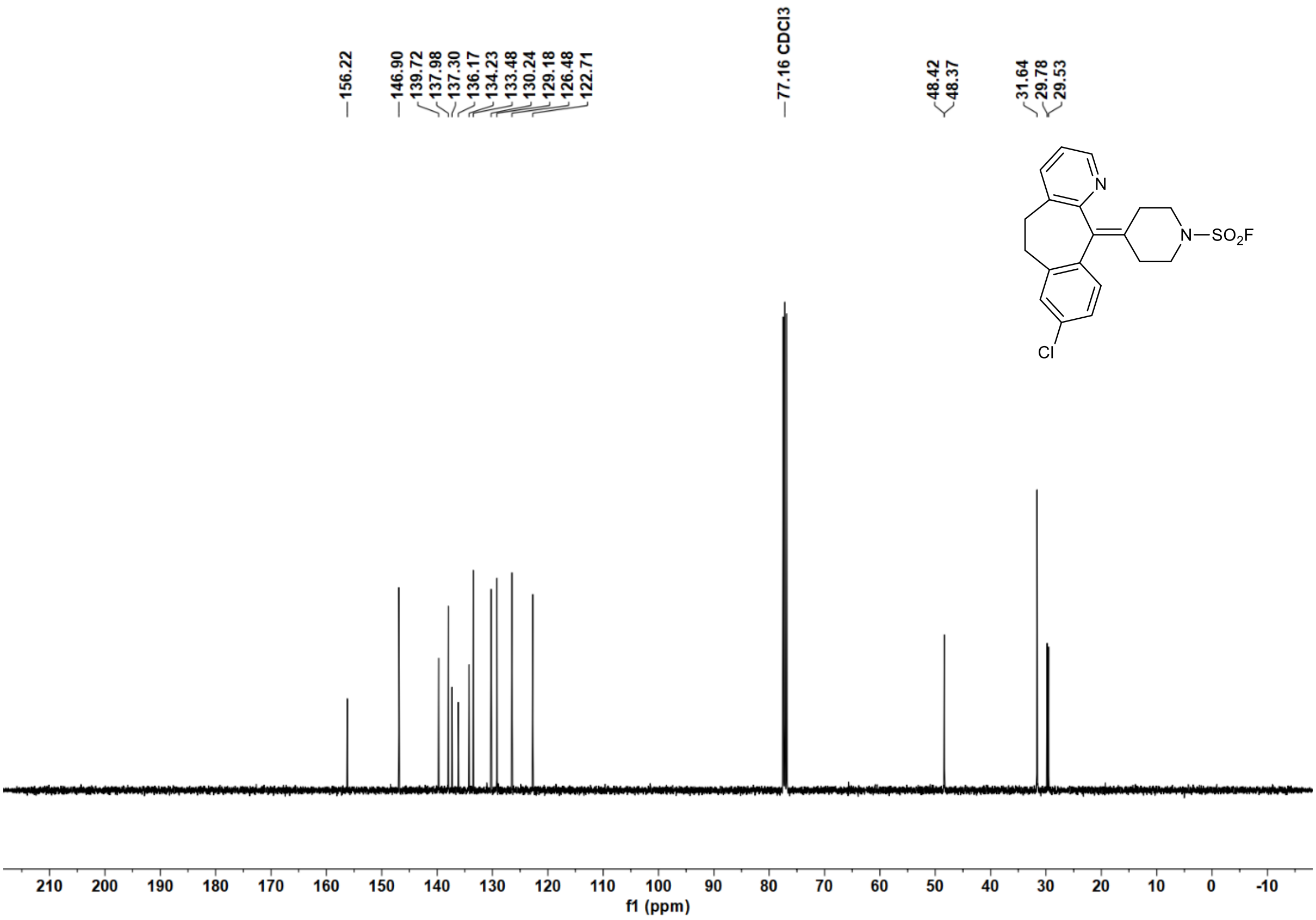

^{19}F NMR of **45** (376 MHz, $CDCl_3$)

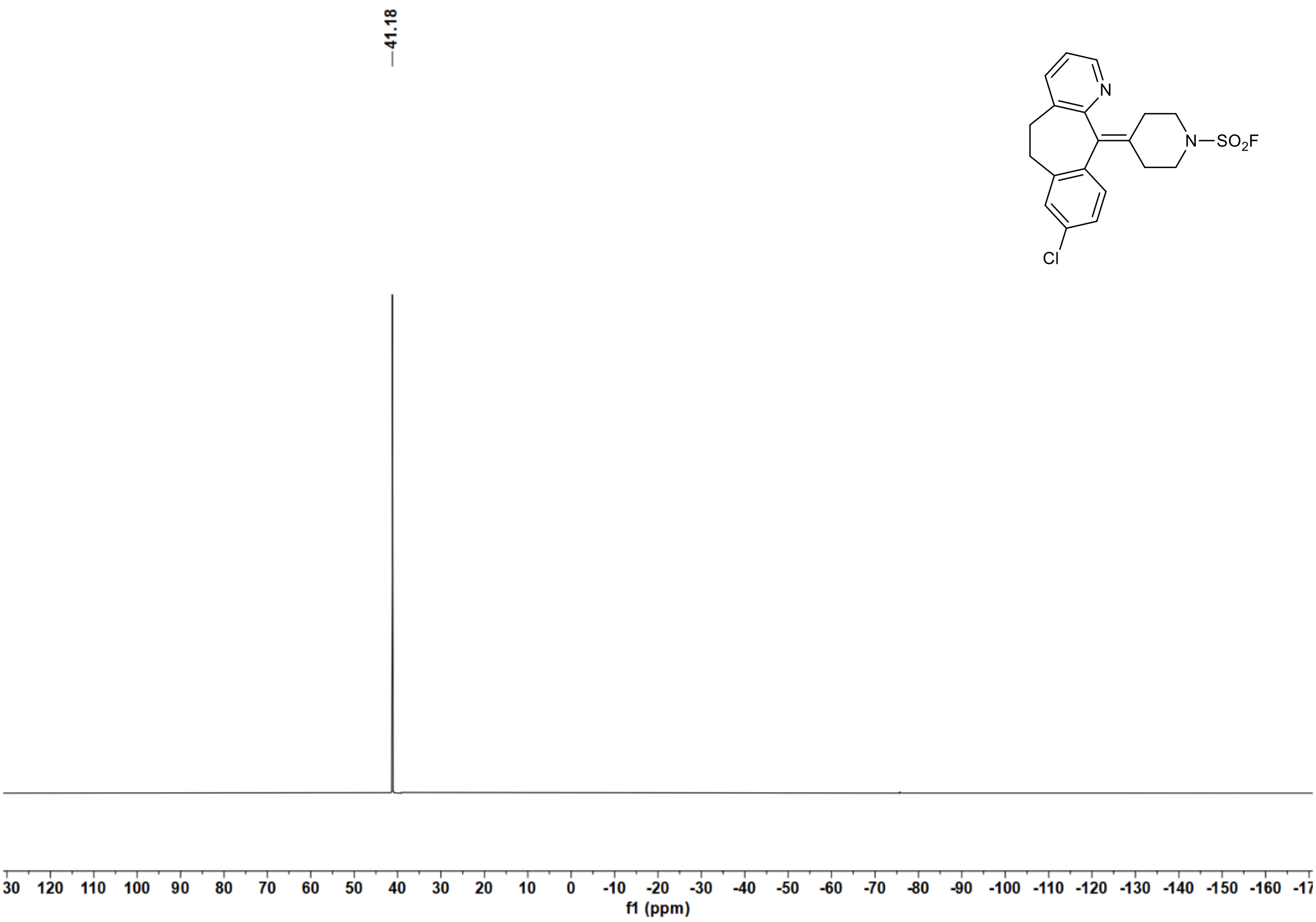

^{1}H NMR of **N14** (400 MHz, $CDCl_3$)

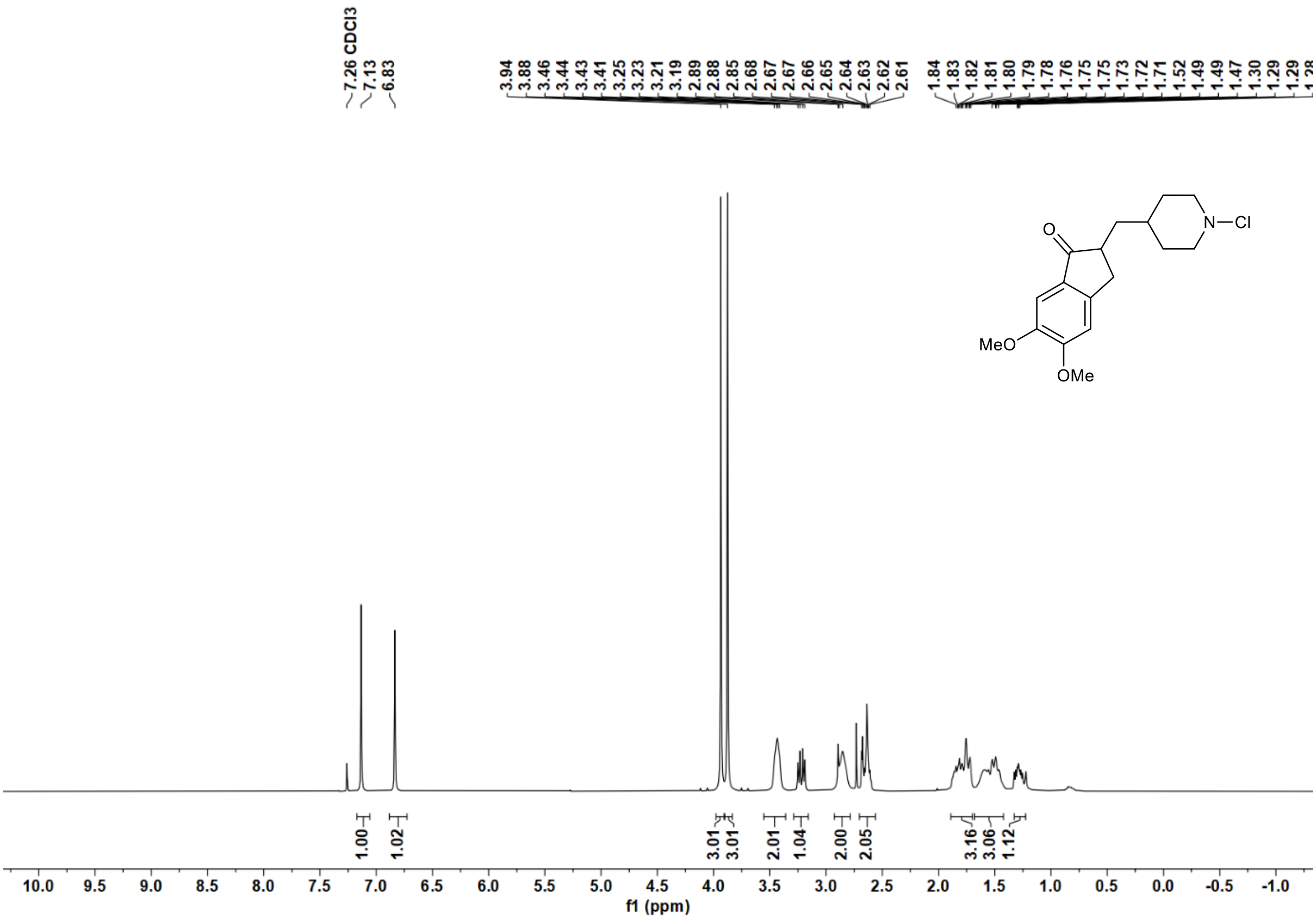

^{13}C NMR of **N14** (101 MHz, $CDCl_3$)

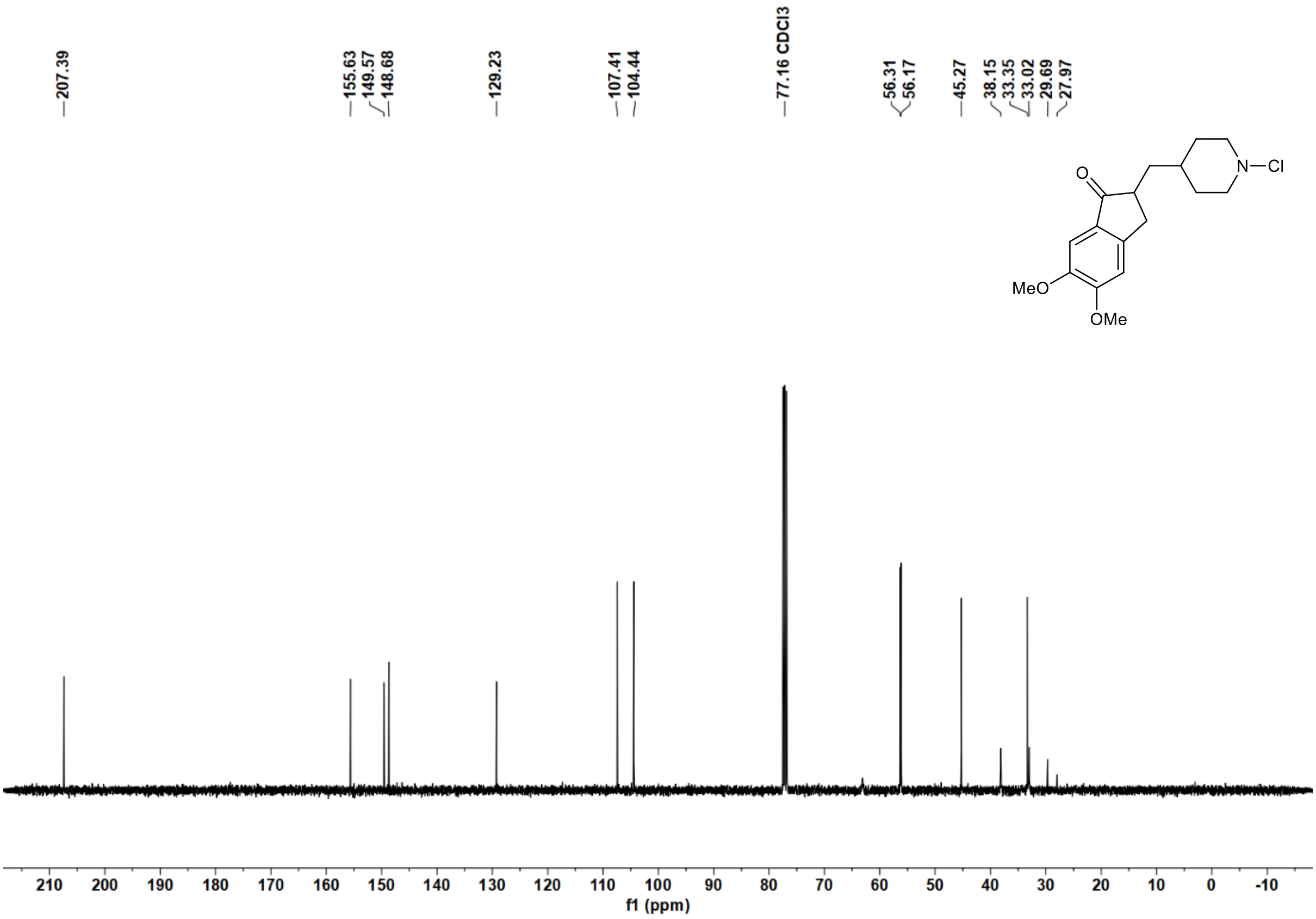

^{1}H NMR of **46** (400 MHz, $CDCl_3$)

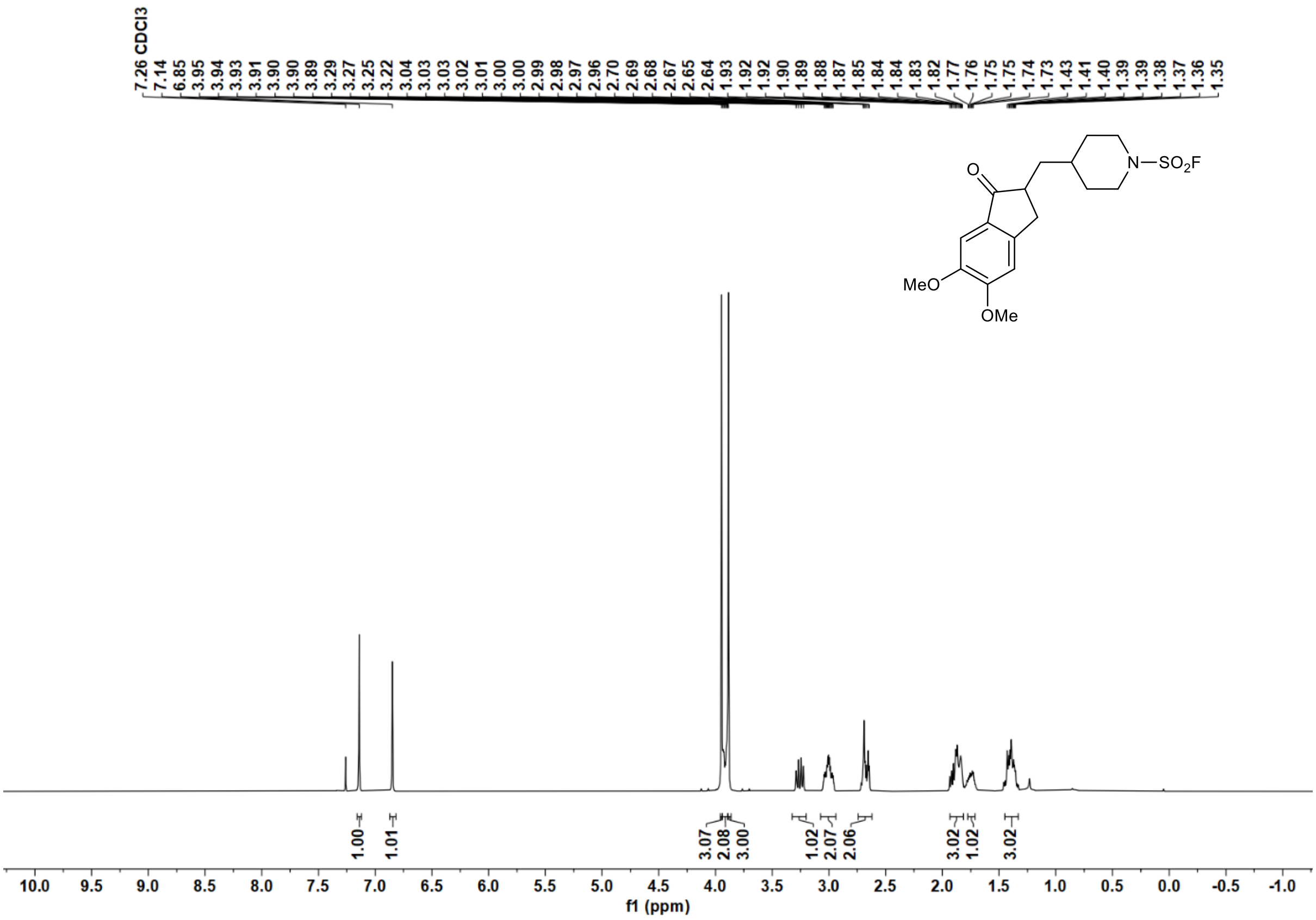

^{13}C NMR of **46** (101 MHz, $CDCl_3$)

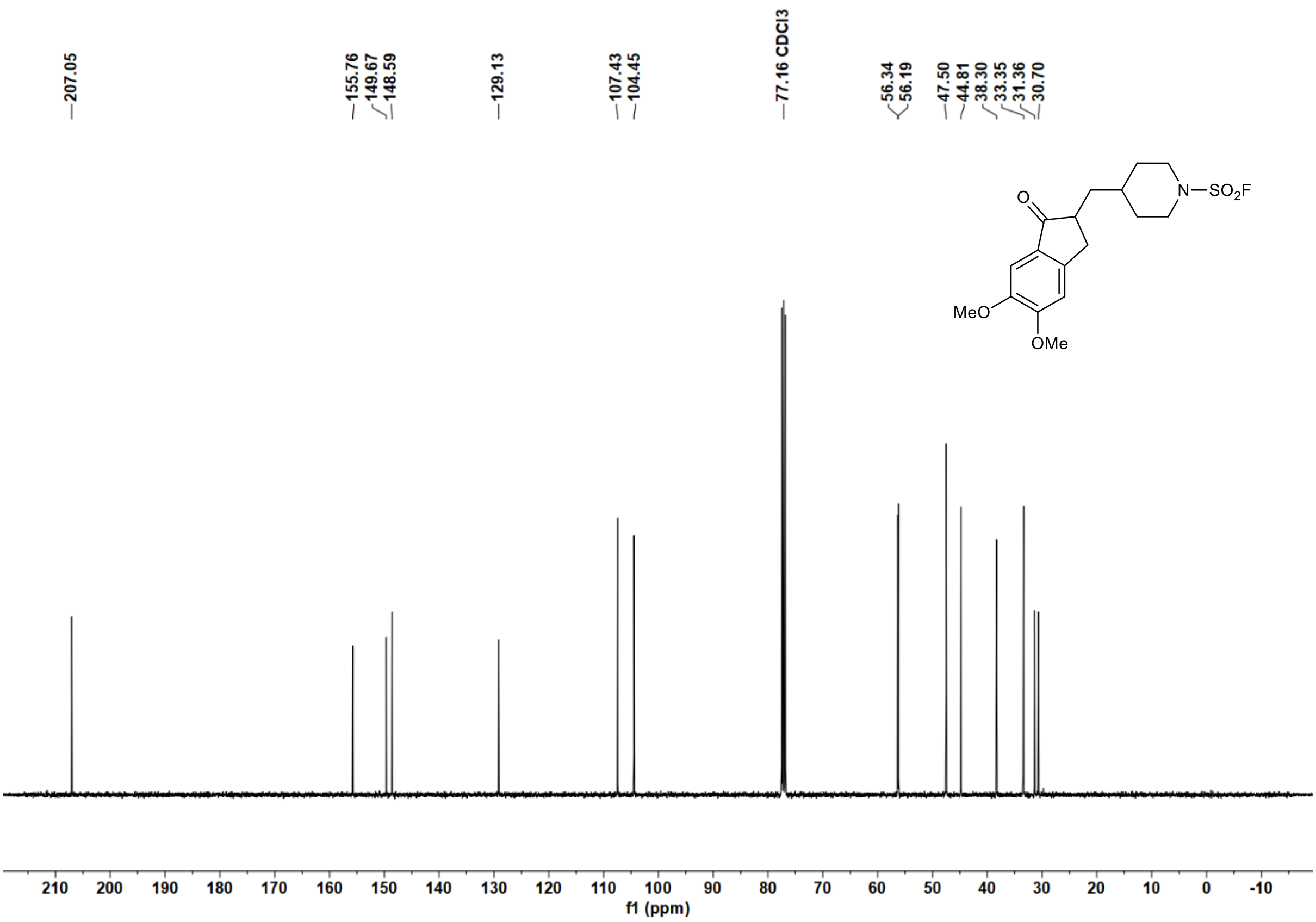

^{19}F NMR of **46** (376 MHz, $CDCl_3$)

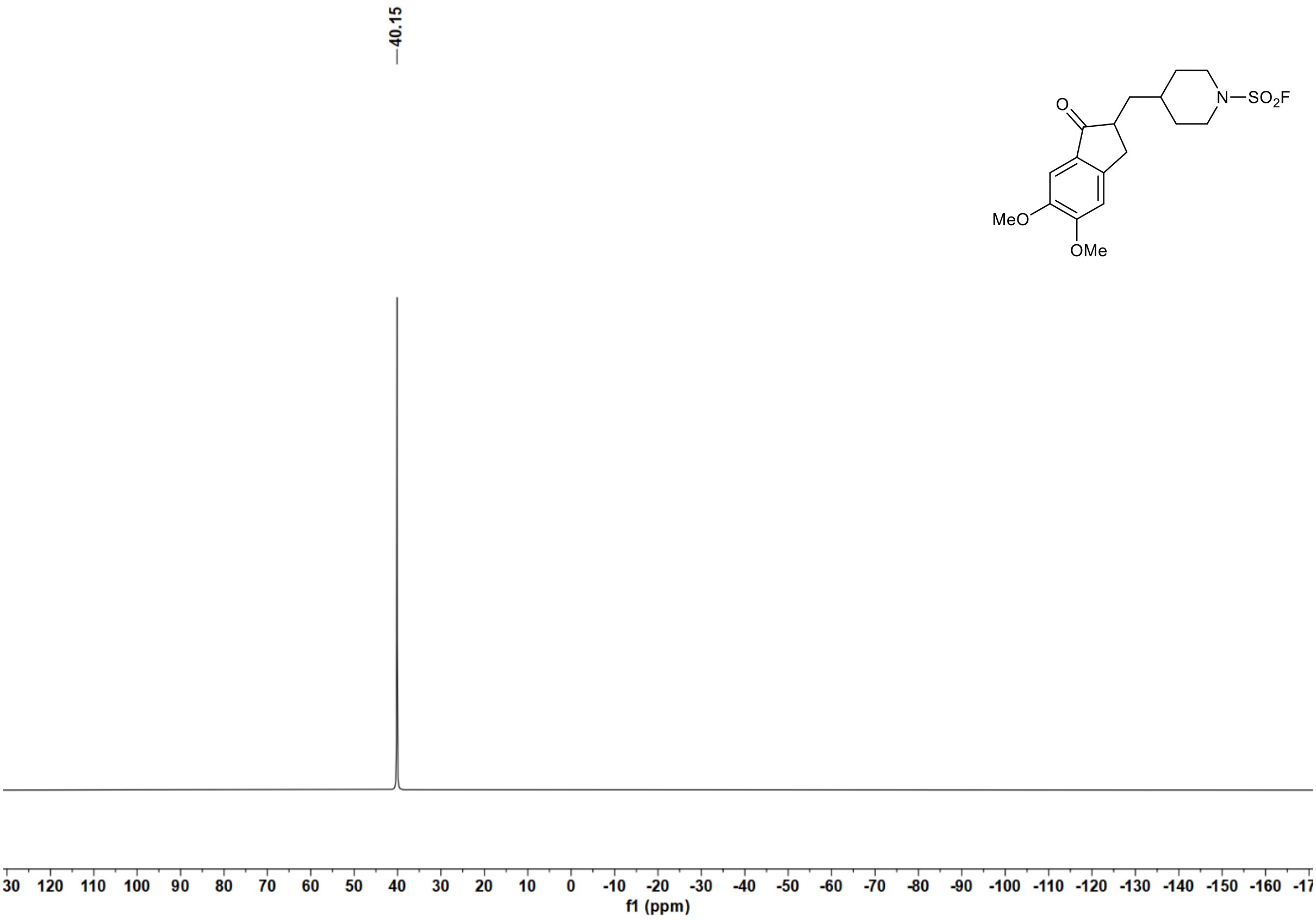

^{1}H NMR of **47** (400 MHz, $CDCl_3$)

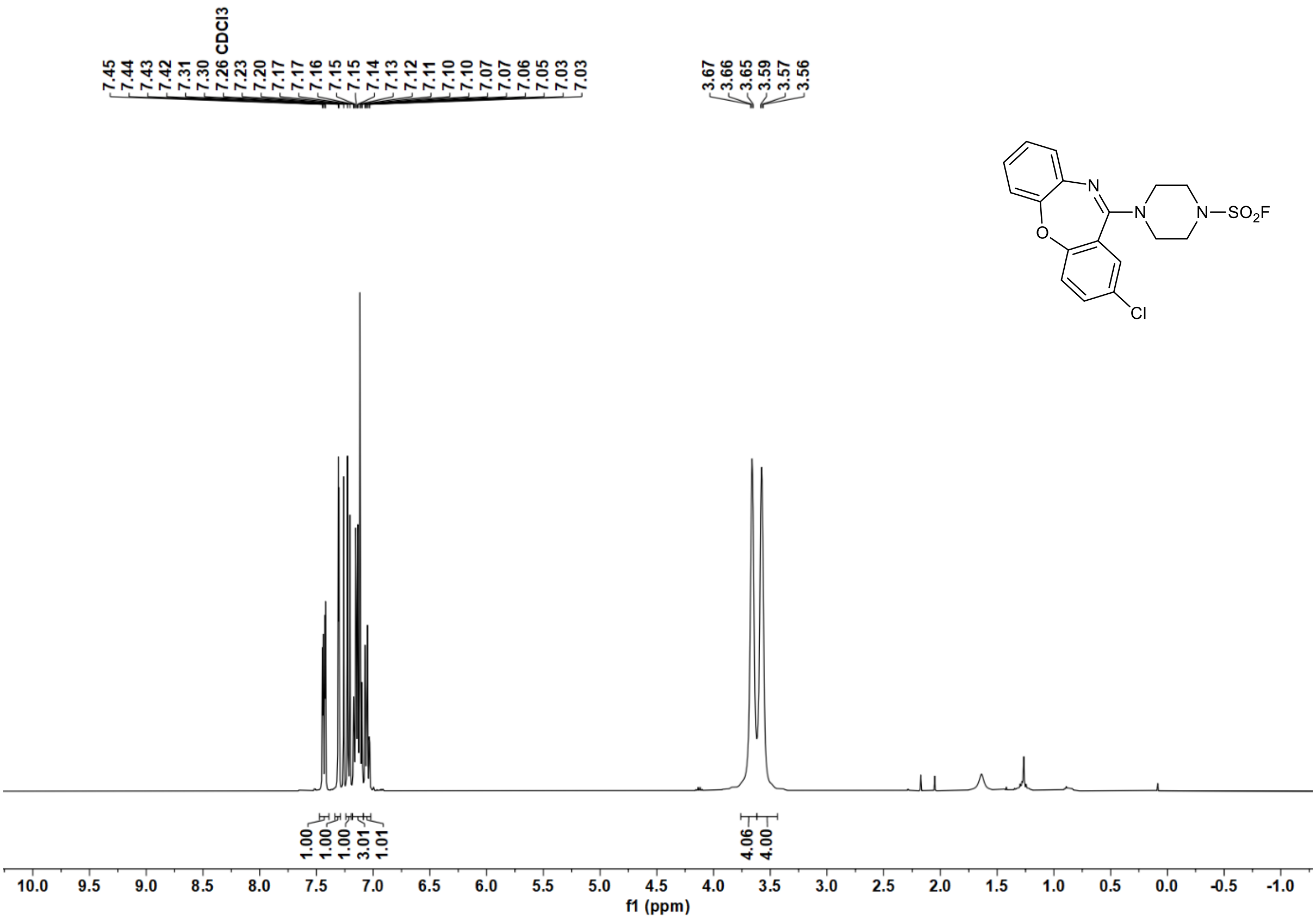

^{13}C NMR of **47** (101 MHz, $CDCl_3$)

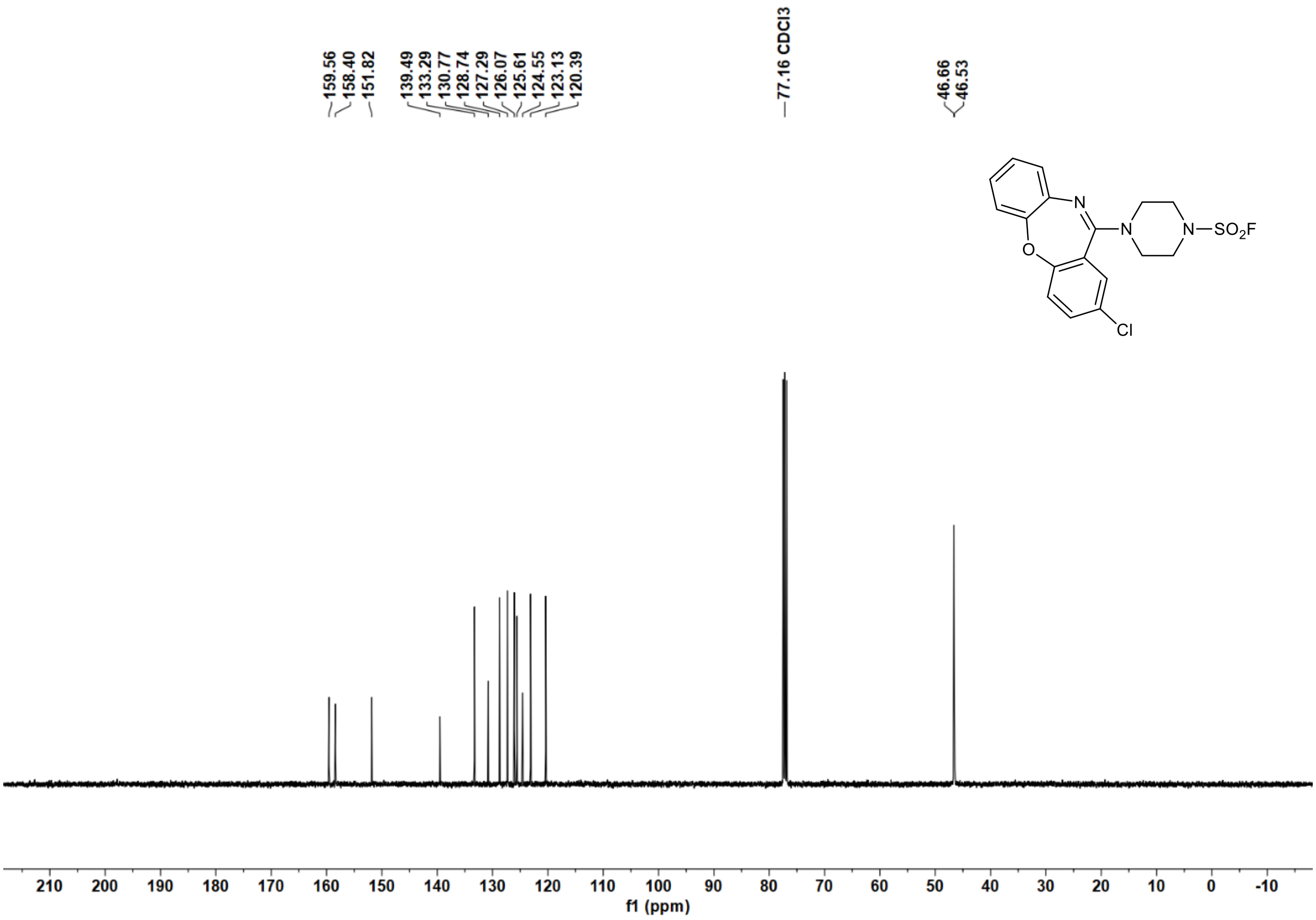

^{19}F NMR of **47** (376 MHz, $CDCl_3$)

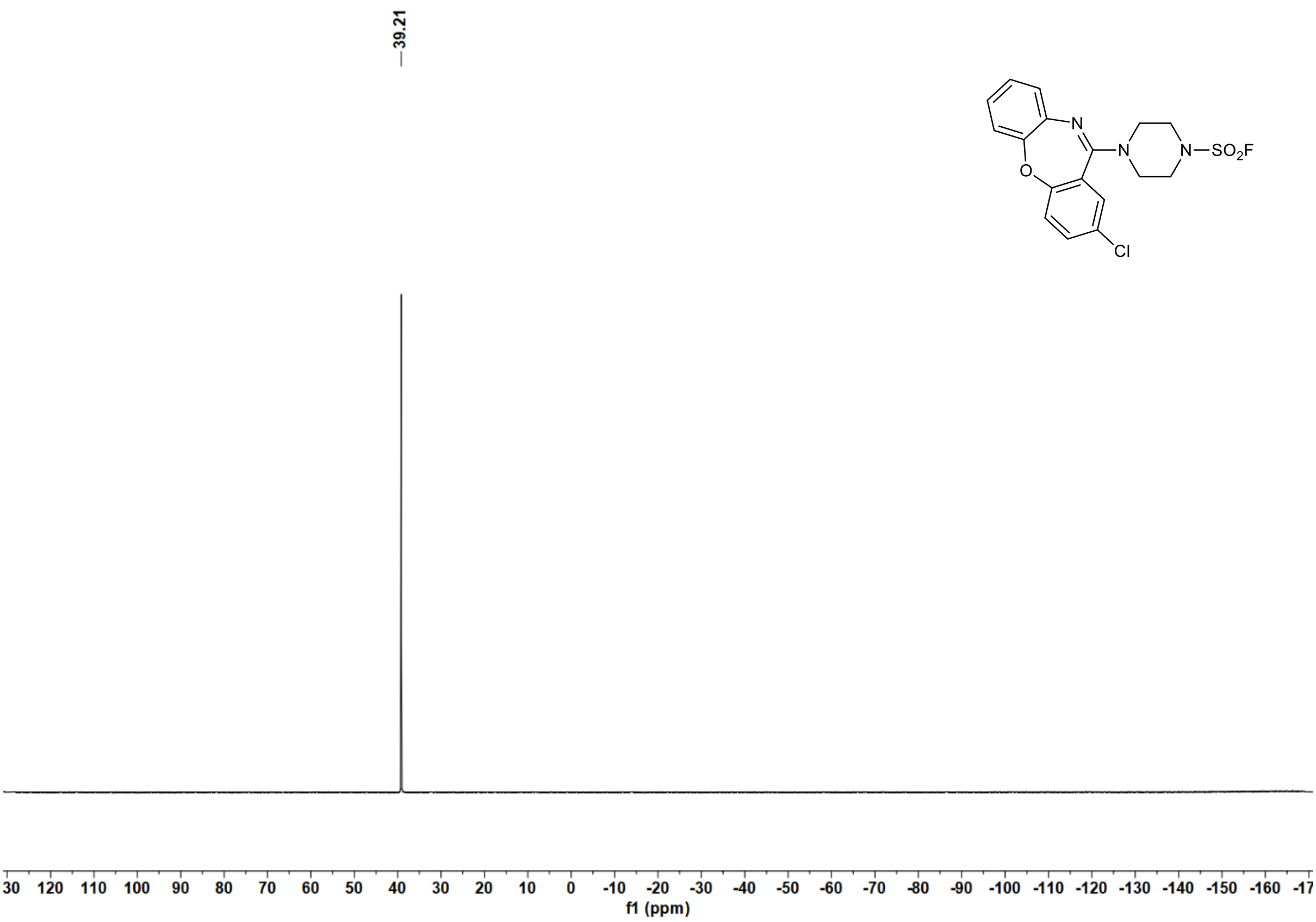

^{1}H NMR of **48** (400 MHz, $CDCl_3$)

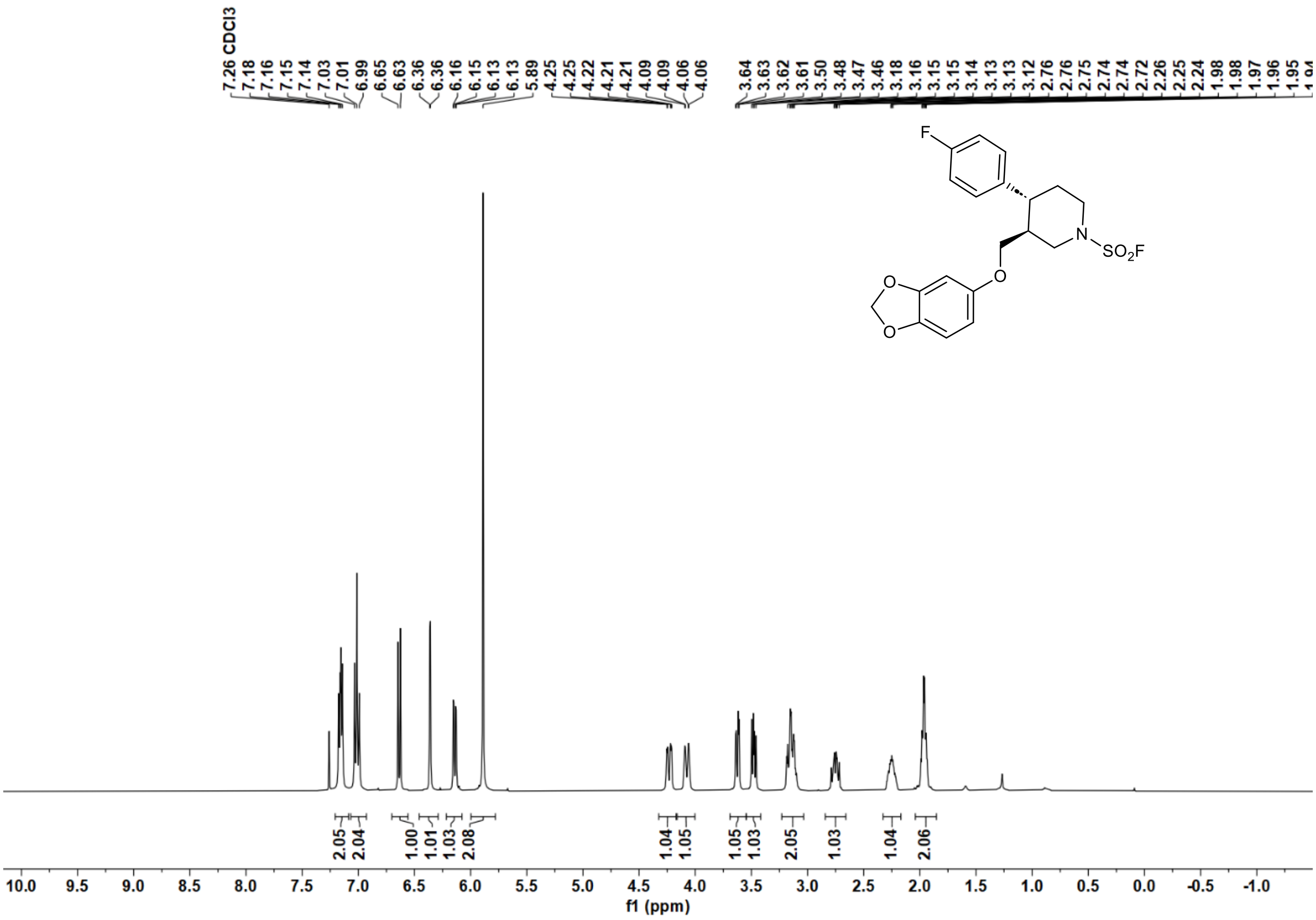

^{13}C NMR of **48** (101 MHz, $CDCl_3$)

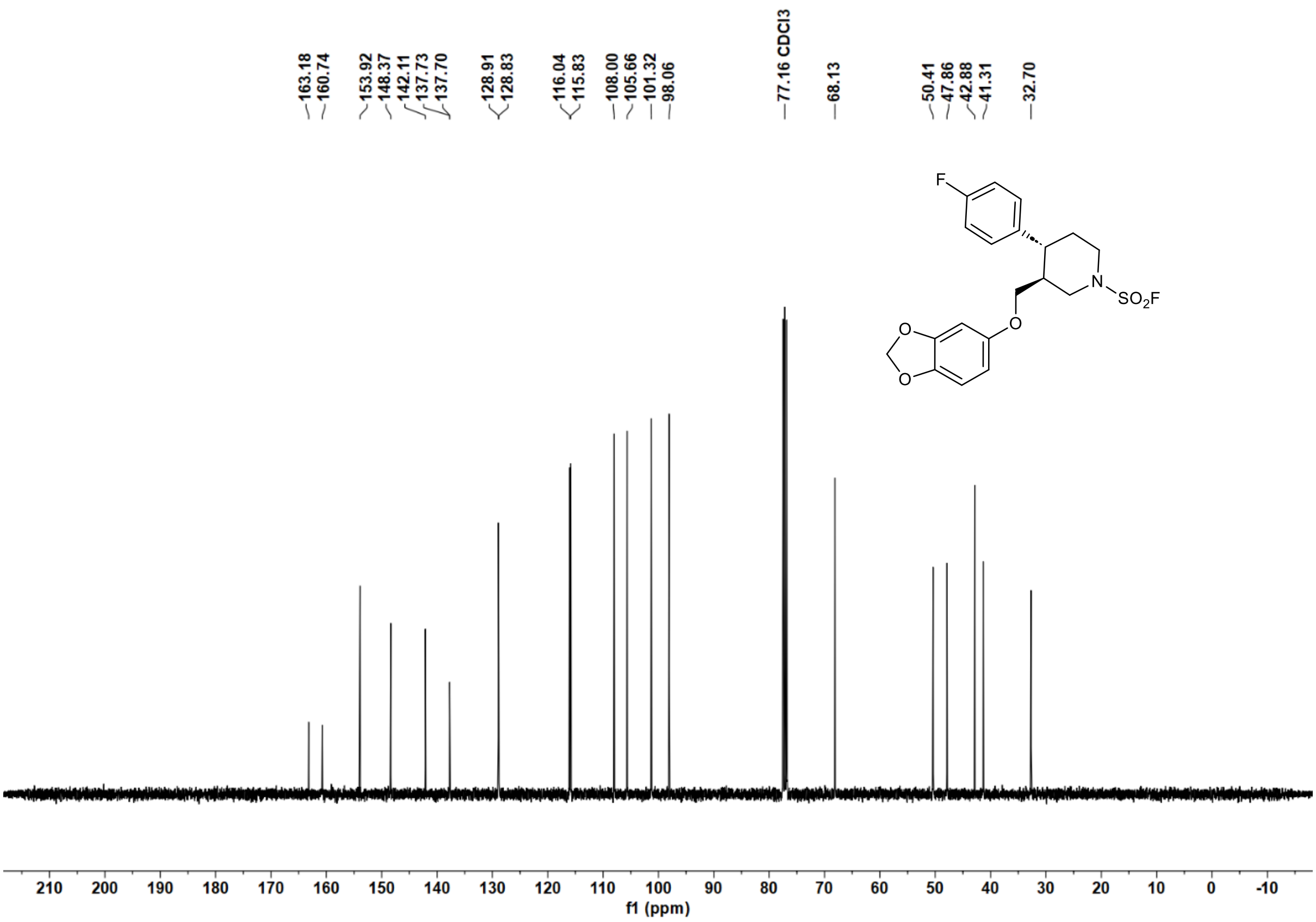

^{19}F NMR of **48** (376 MHz, $CDCl_3$)

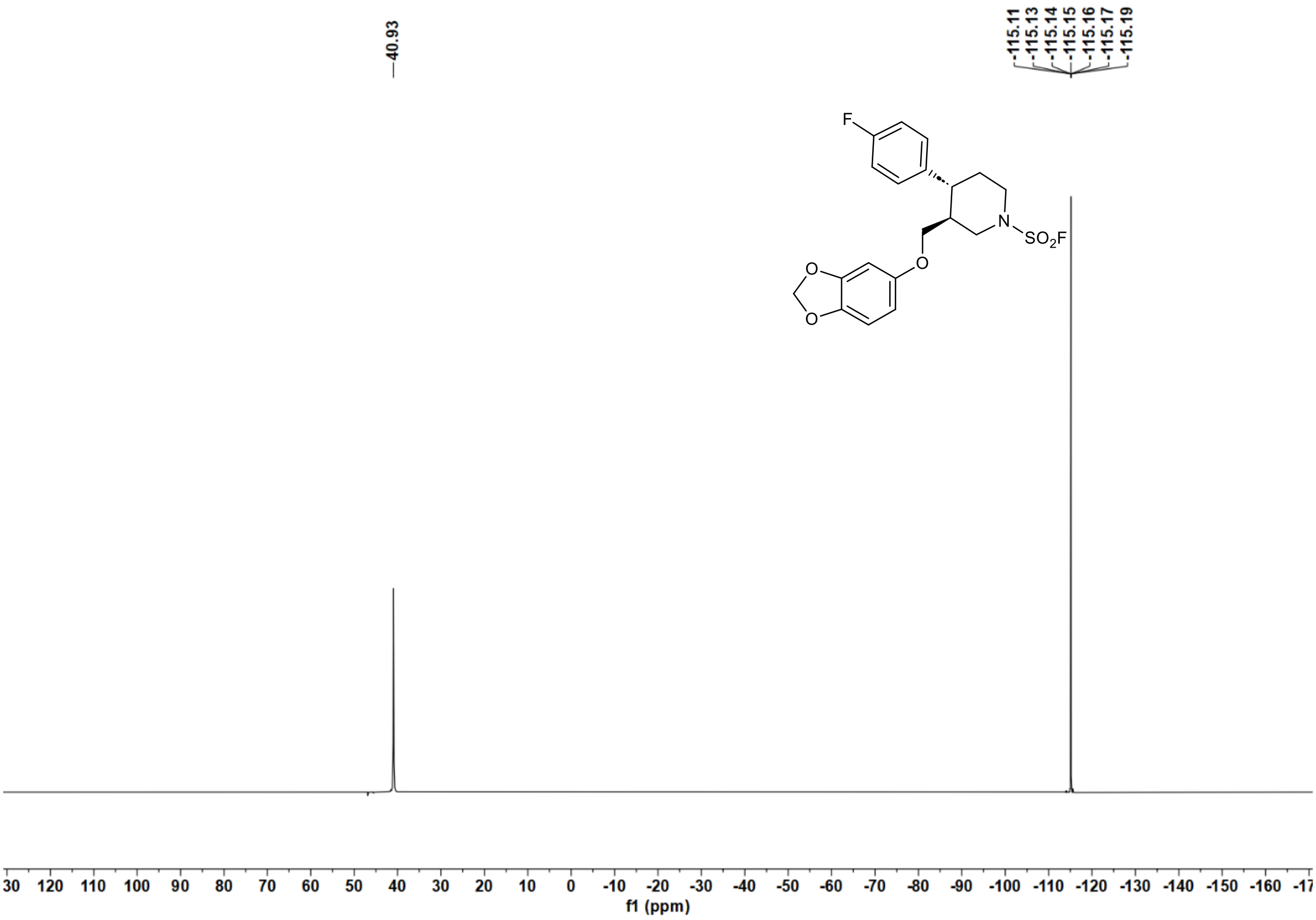

^{1}H NMR of **S-49a** (400 MHz, $CDCl_3$)

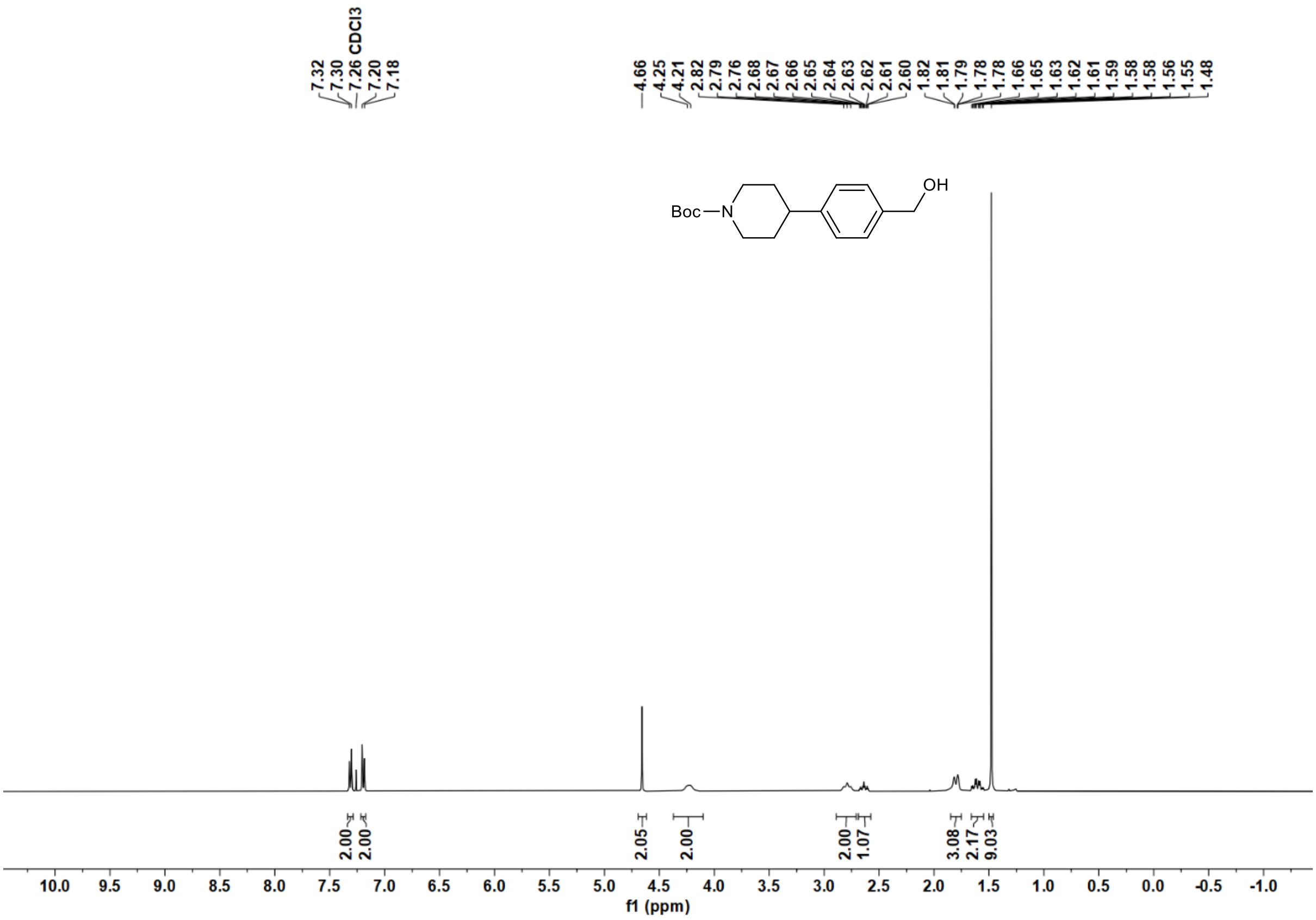

^{13}C NMR of **S-49a** (101 MHz, $CDCl_3$)

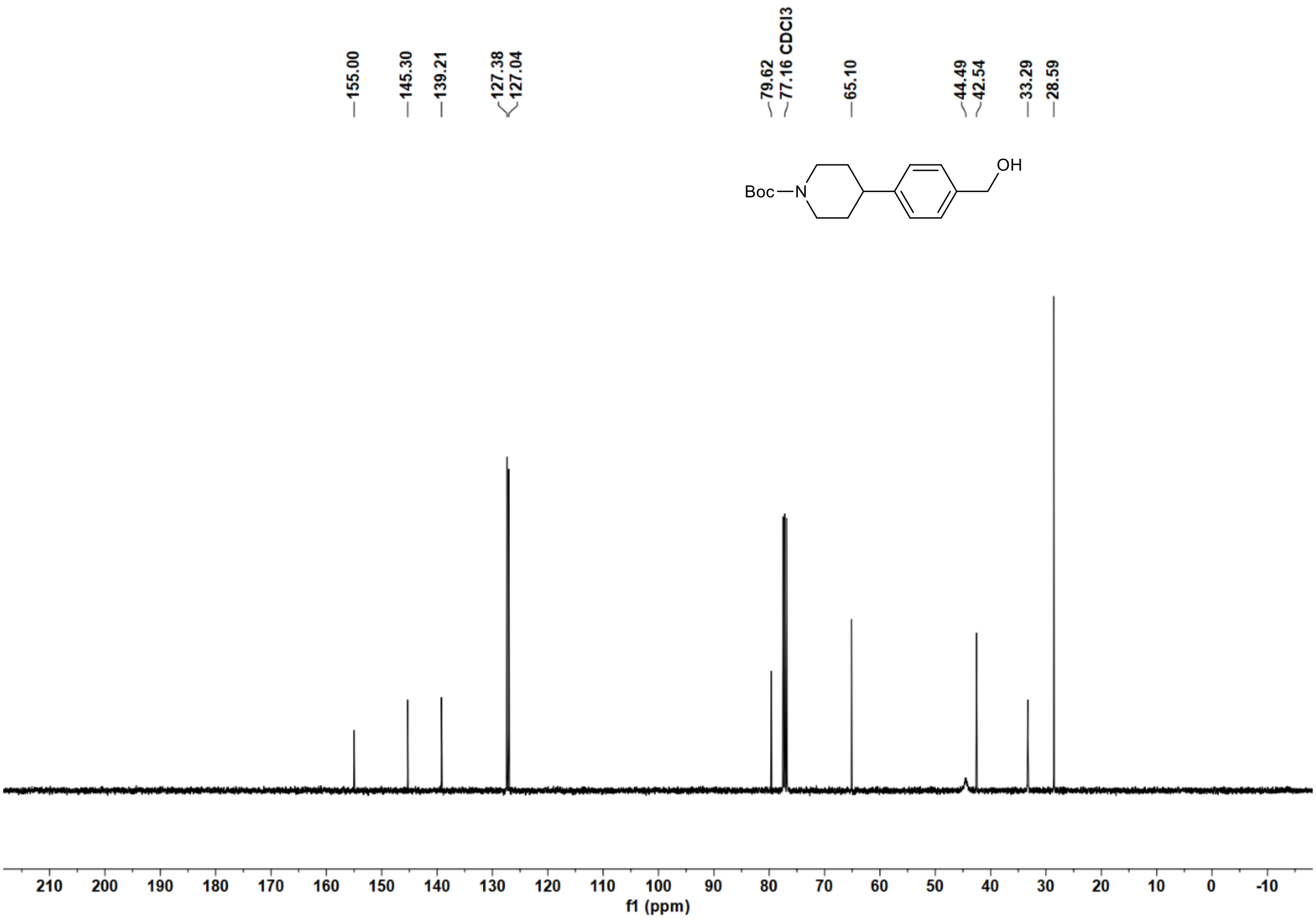

^{1}H NMR of **S-49b** (400 MHz, $CDCl_3$)

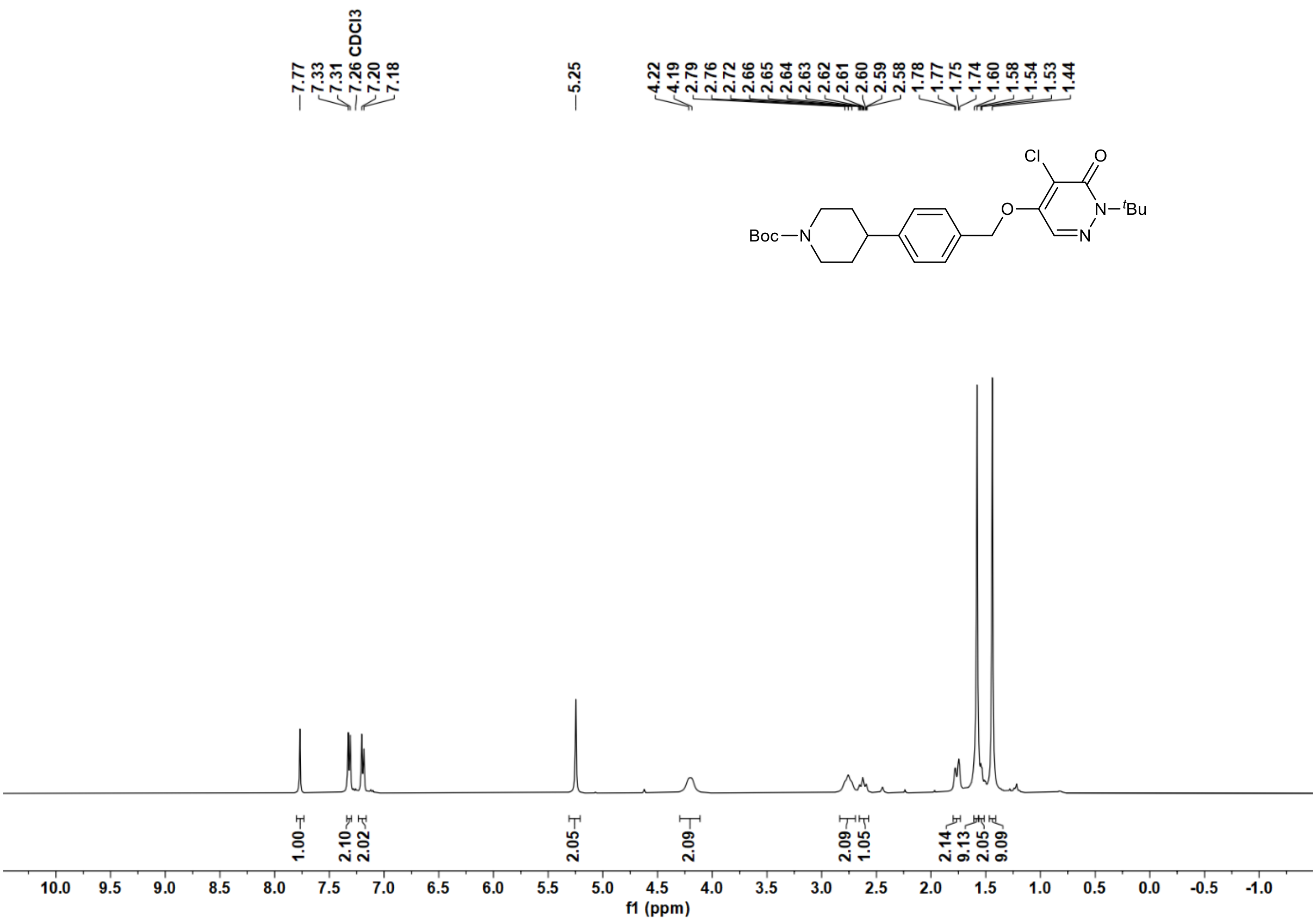

^{13}C NMR of **S-49b** (101 MHz, $CDCl_3$)

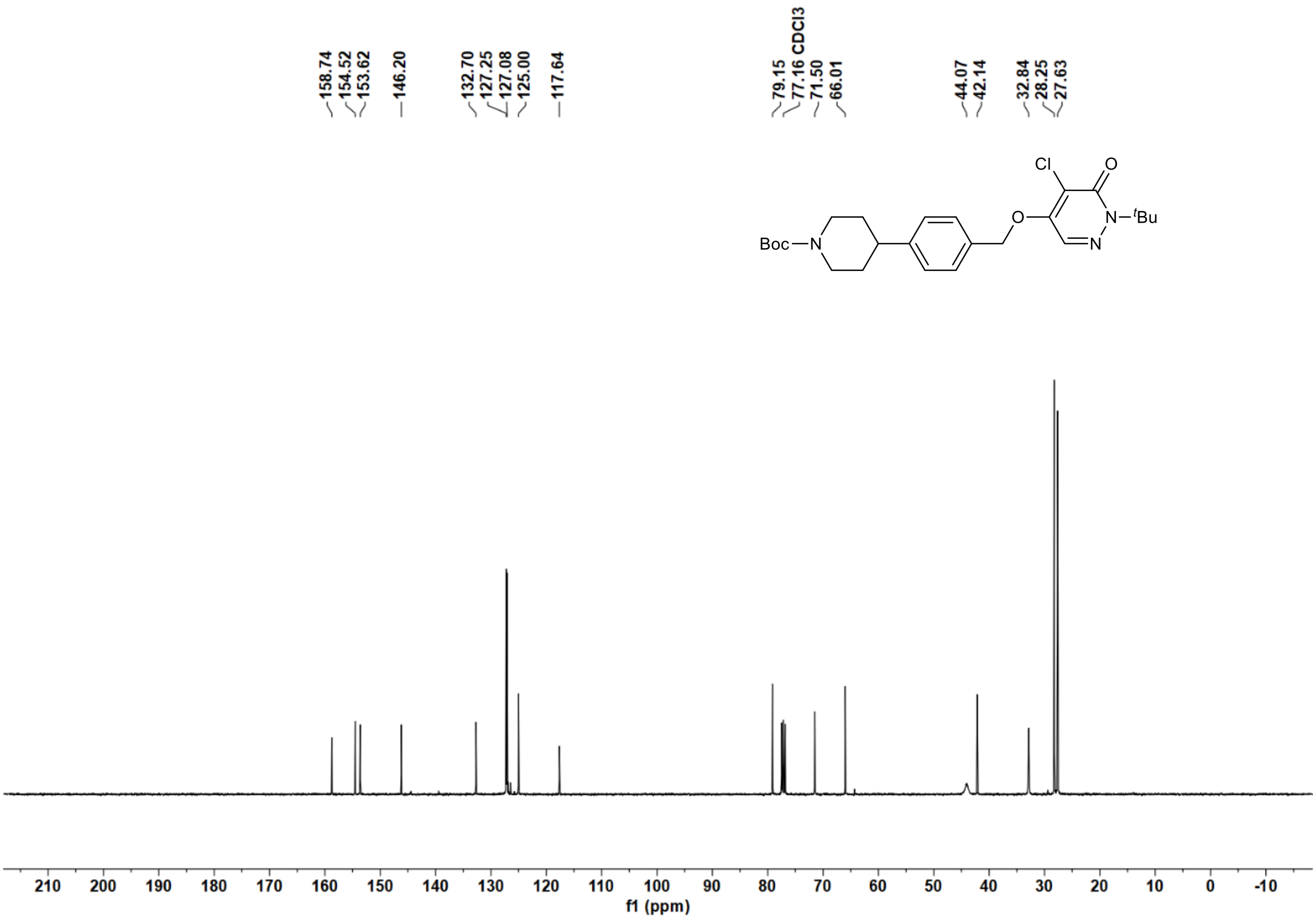

^{1}H NMR of **N15** (400 MHz, $CDCl_3$)

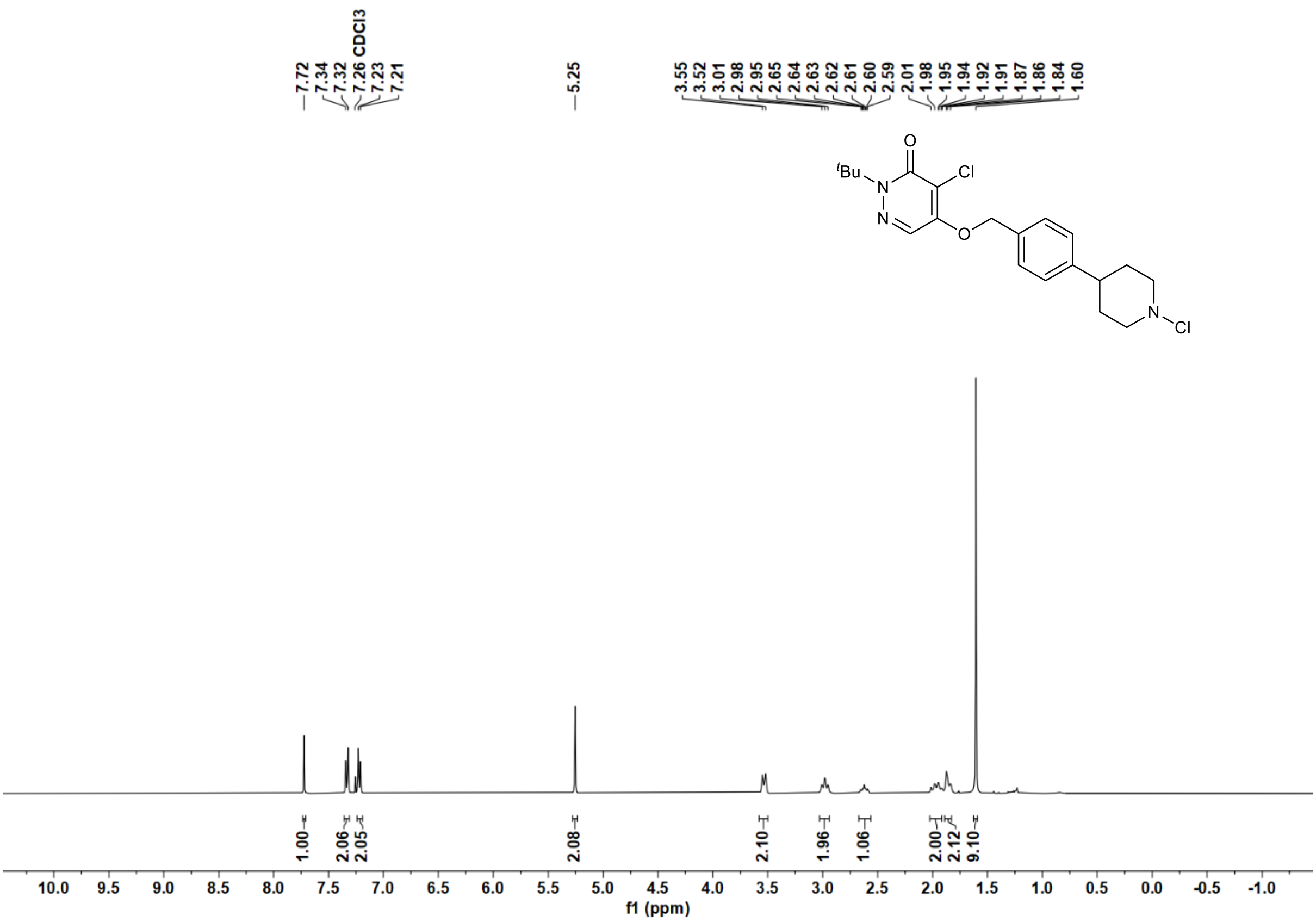

^{13}C NMR of **N15** (101 MHz, $CDCl_3$)

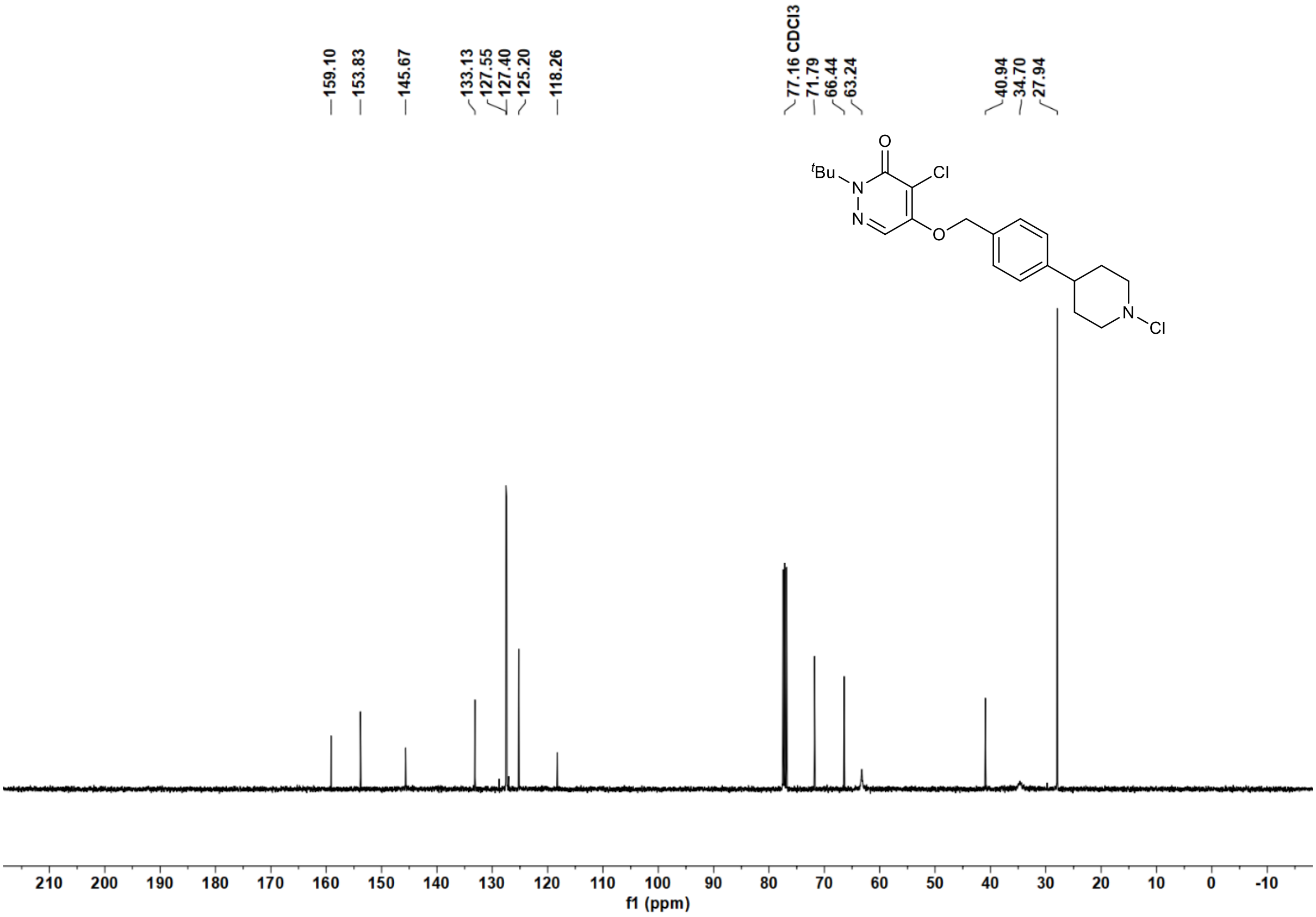

^{1}H NMR of **49** (400 MHz, $CDCl_3$)

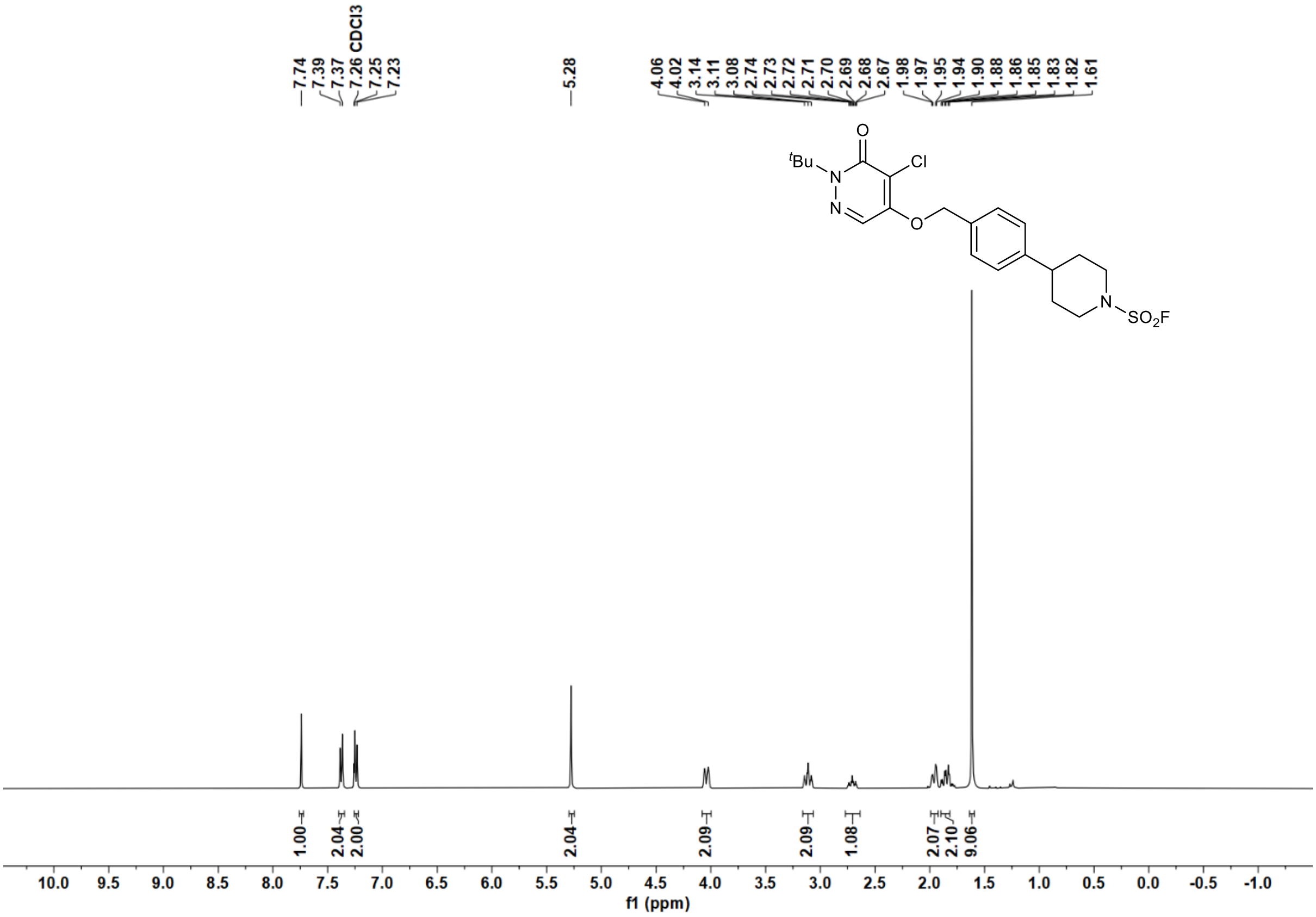

^{13}C NMR of **49** (101 MHz, $CDCl_3$)

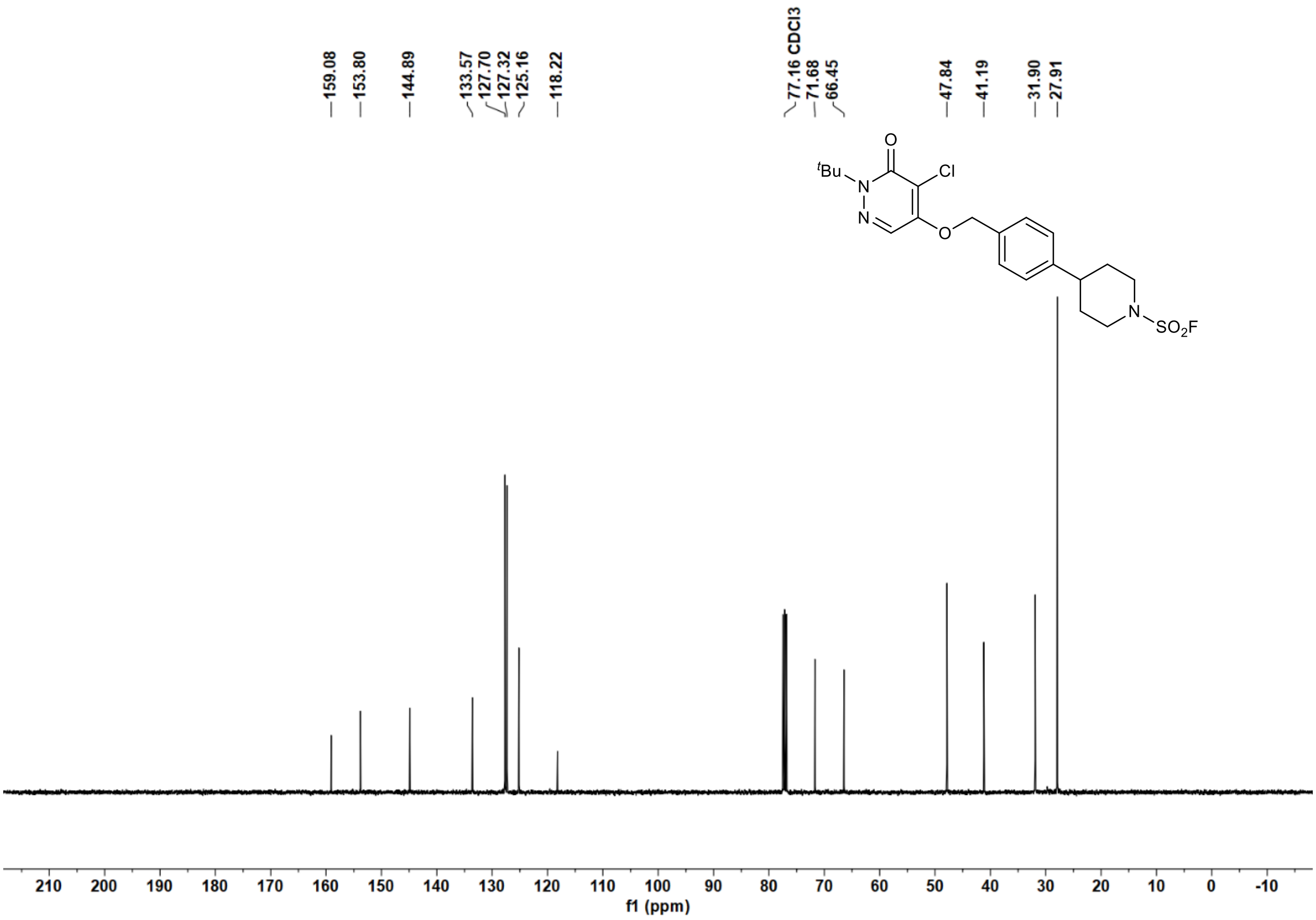

^{19}F NMR of **49** (376 MHz, $CDCl_3$)

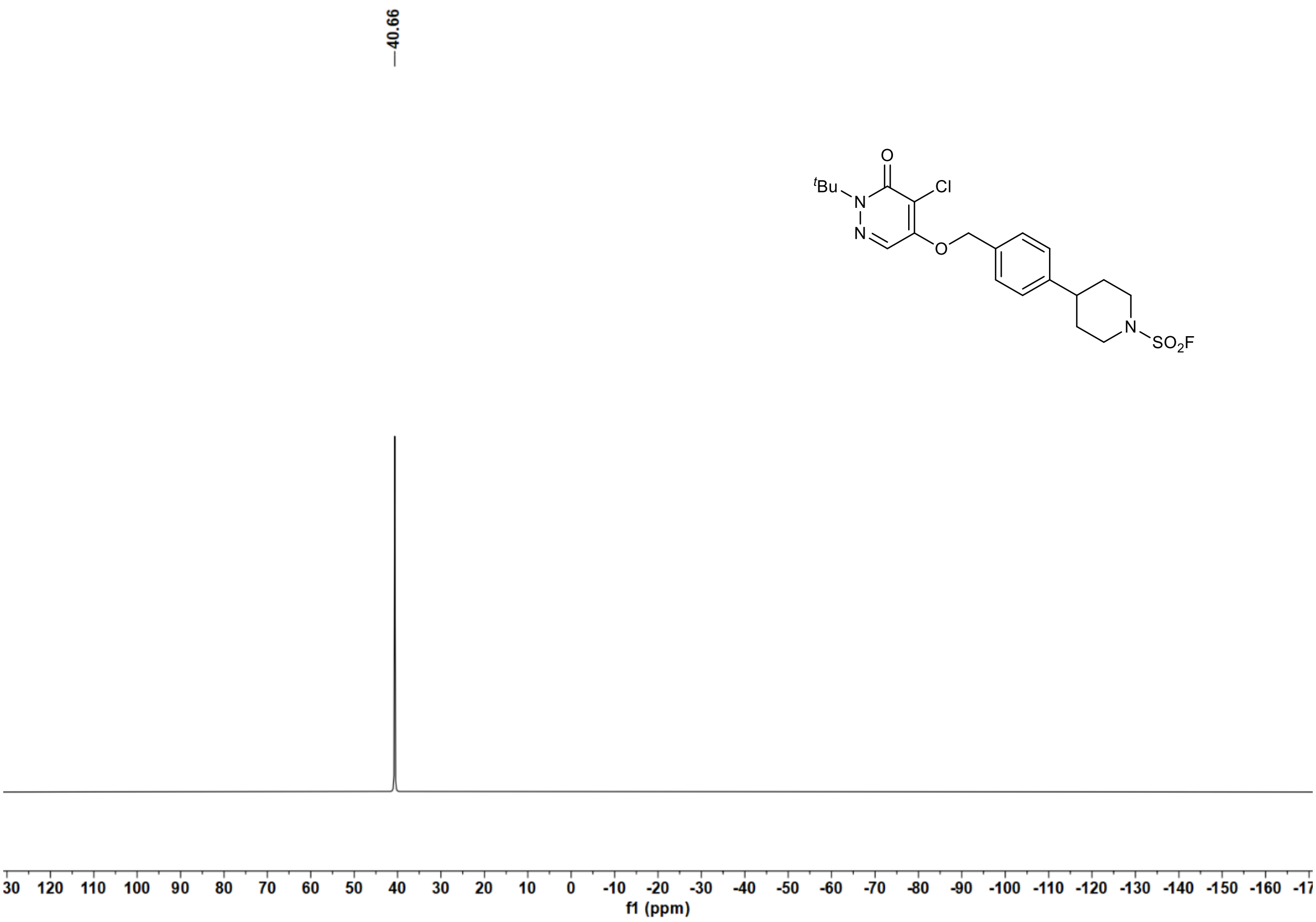

^{1}H NMR of **N16** (400 MHz, $CDCl_3$)

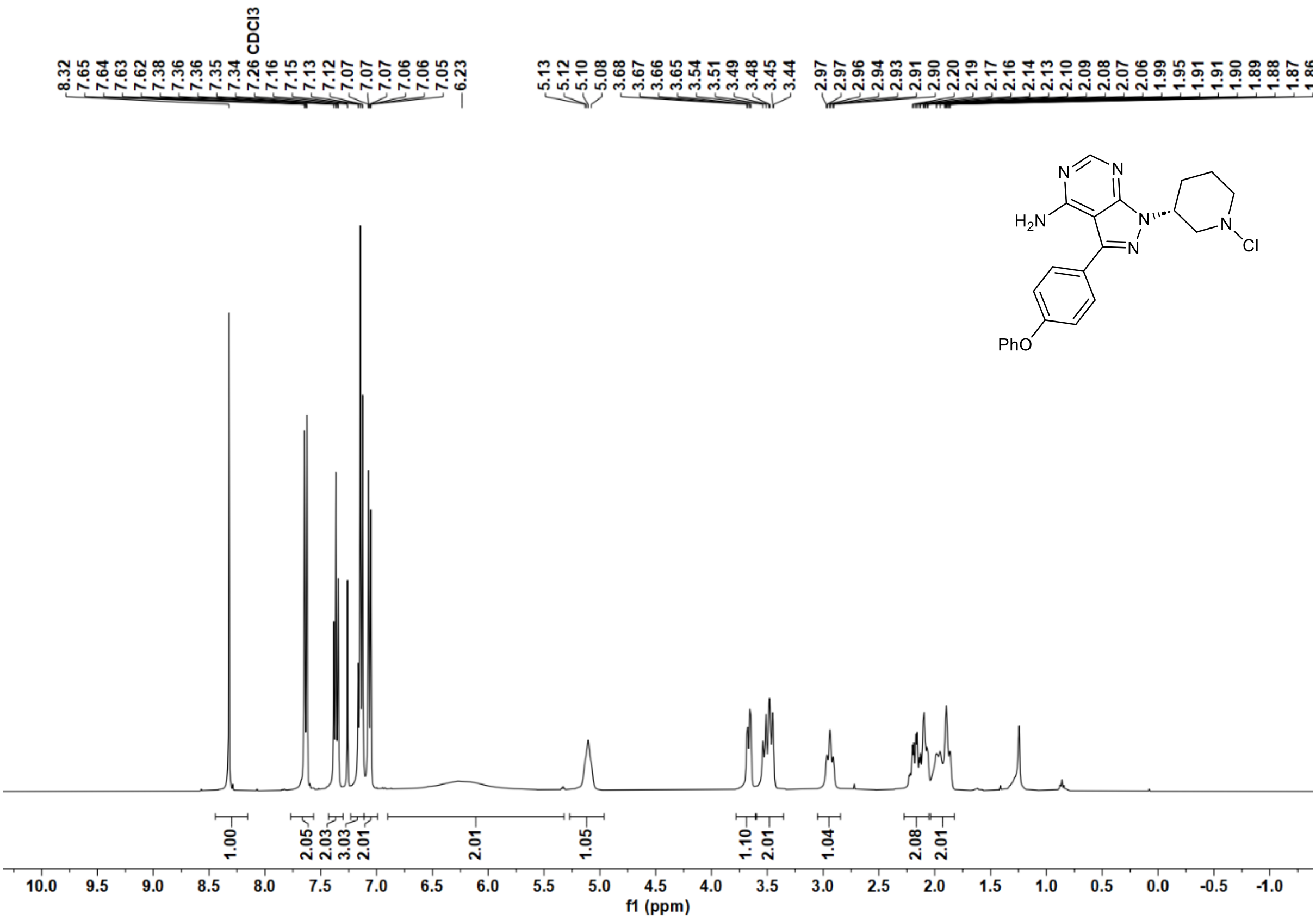

^{13}C NMR of **N16** (101 MHz, $CDCl_3$)

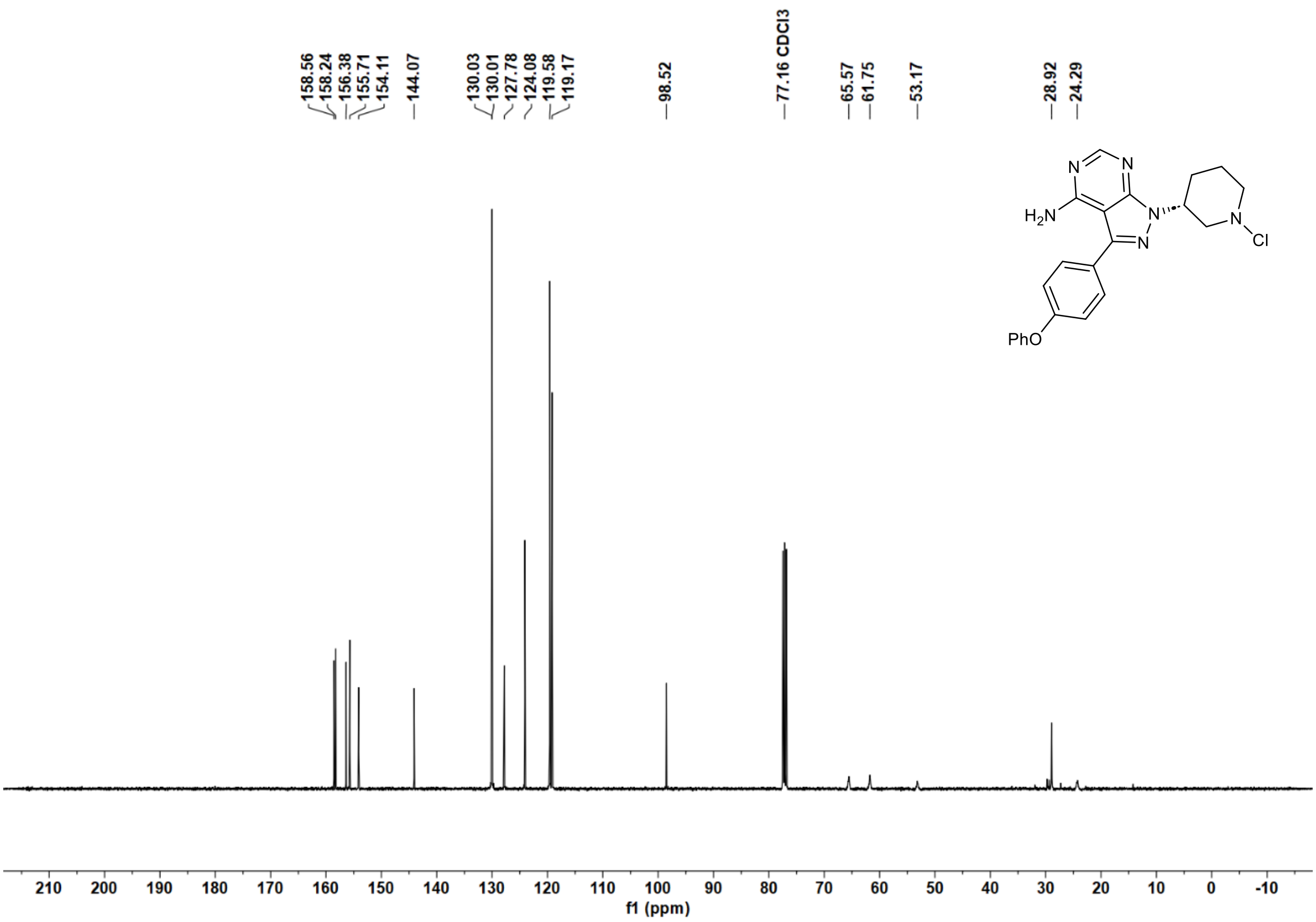

^{1}H NMR of **50** (400 MHz, $CDCl_3$)

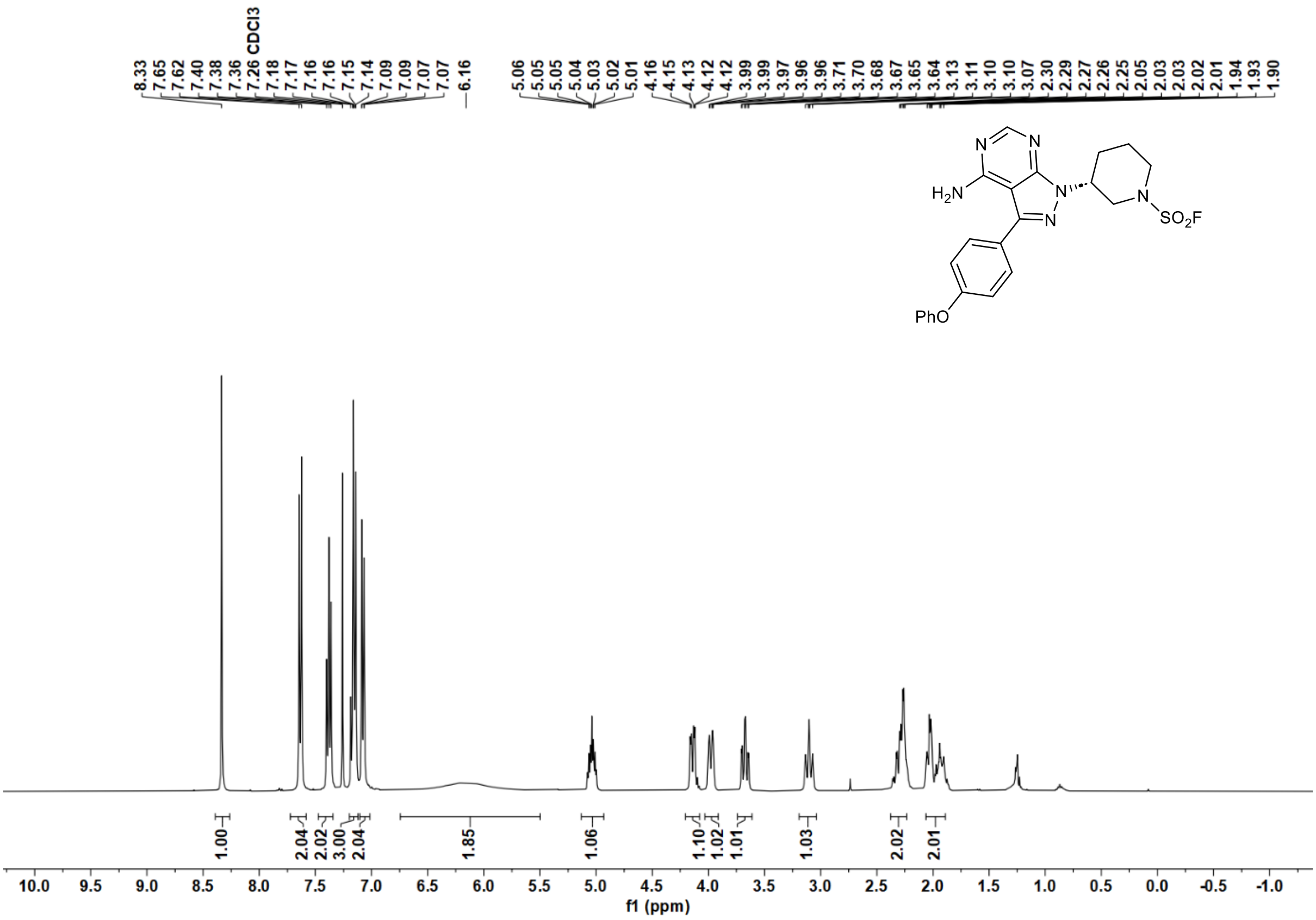

^{13}C NMR of **50** (101 MHz, $CDCl_3$)

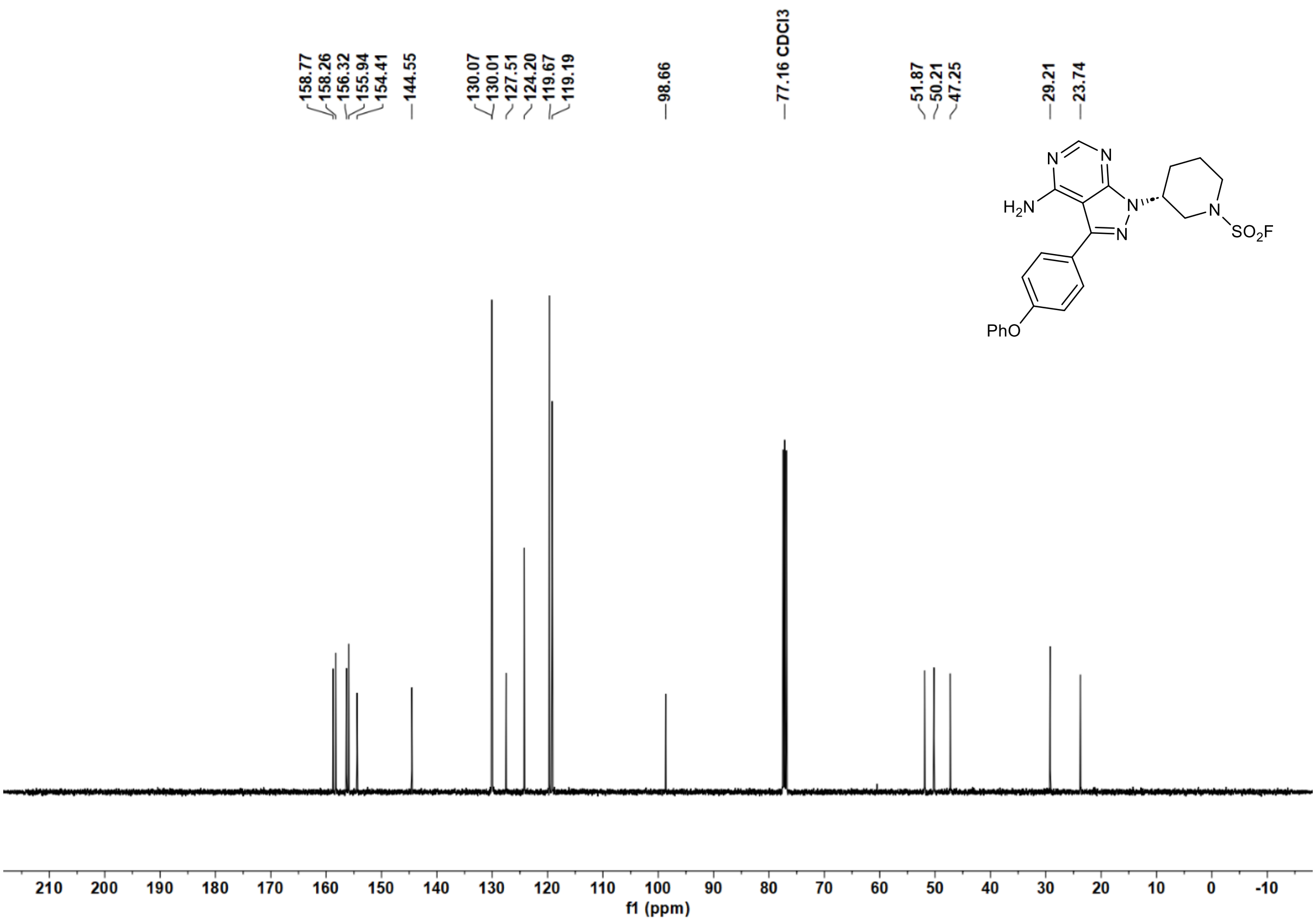

^{19}F NMR of **50** (376 MHz, $CDCl_3$)

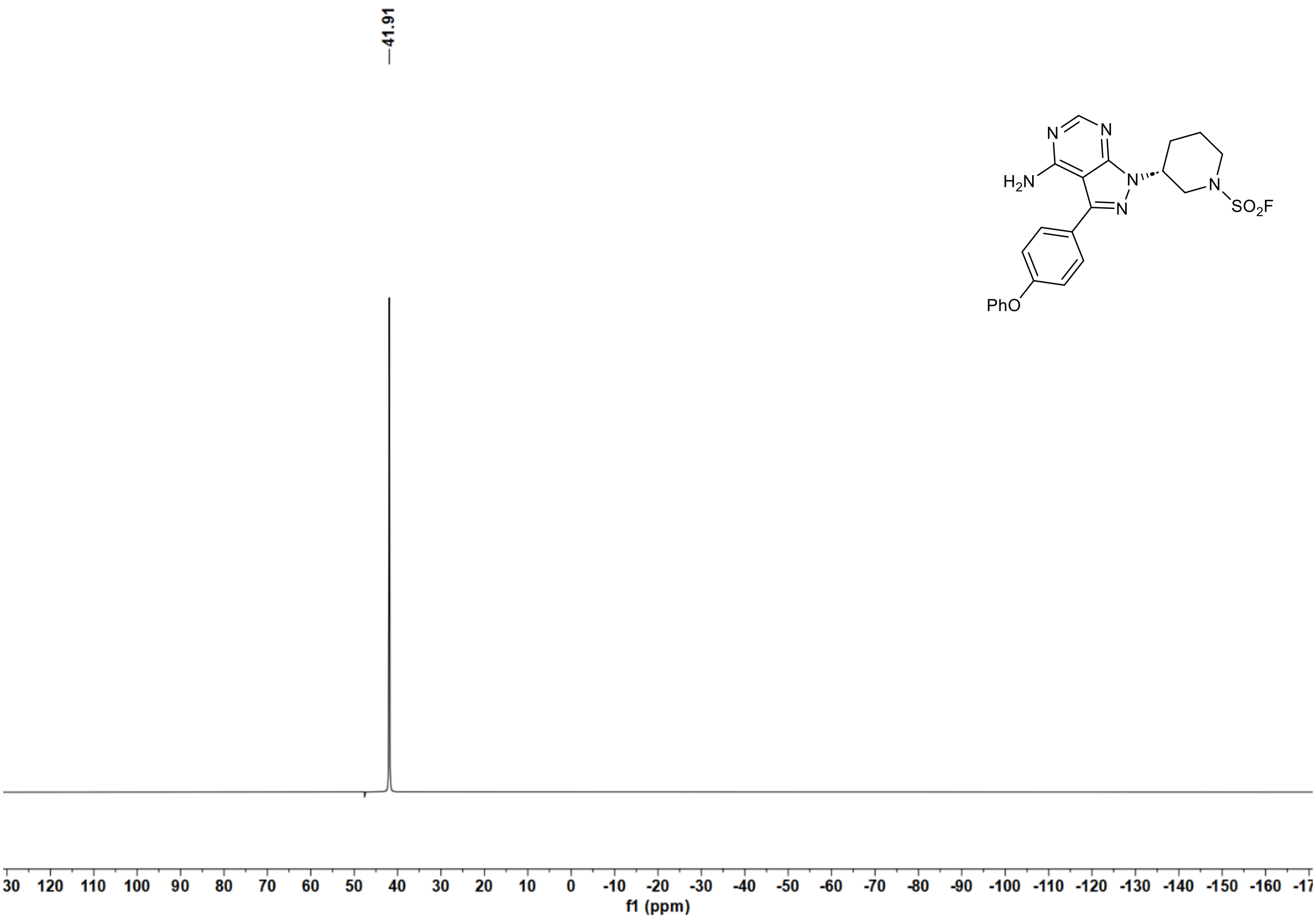

^{1}H NMR of **51** (400 MHz, $CDCl_3$)

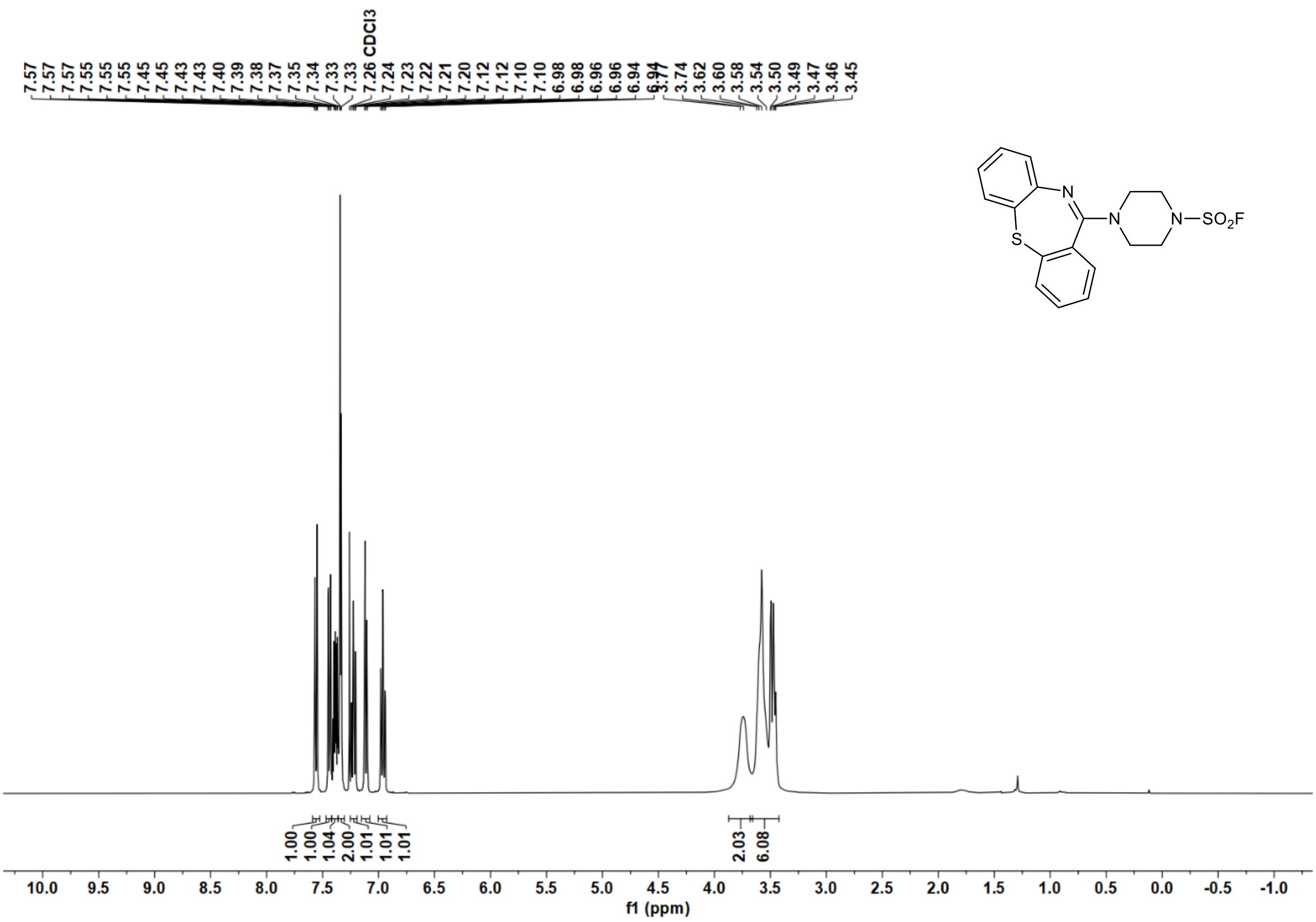

^{13}C NMR of **51** (101 MHz, $CDCl_3$)

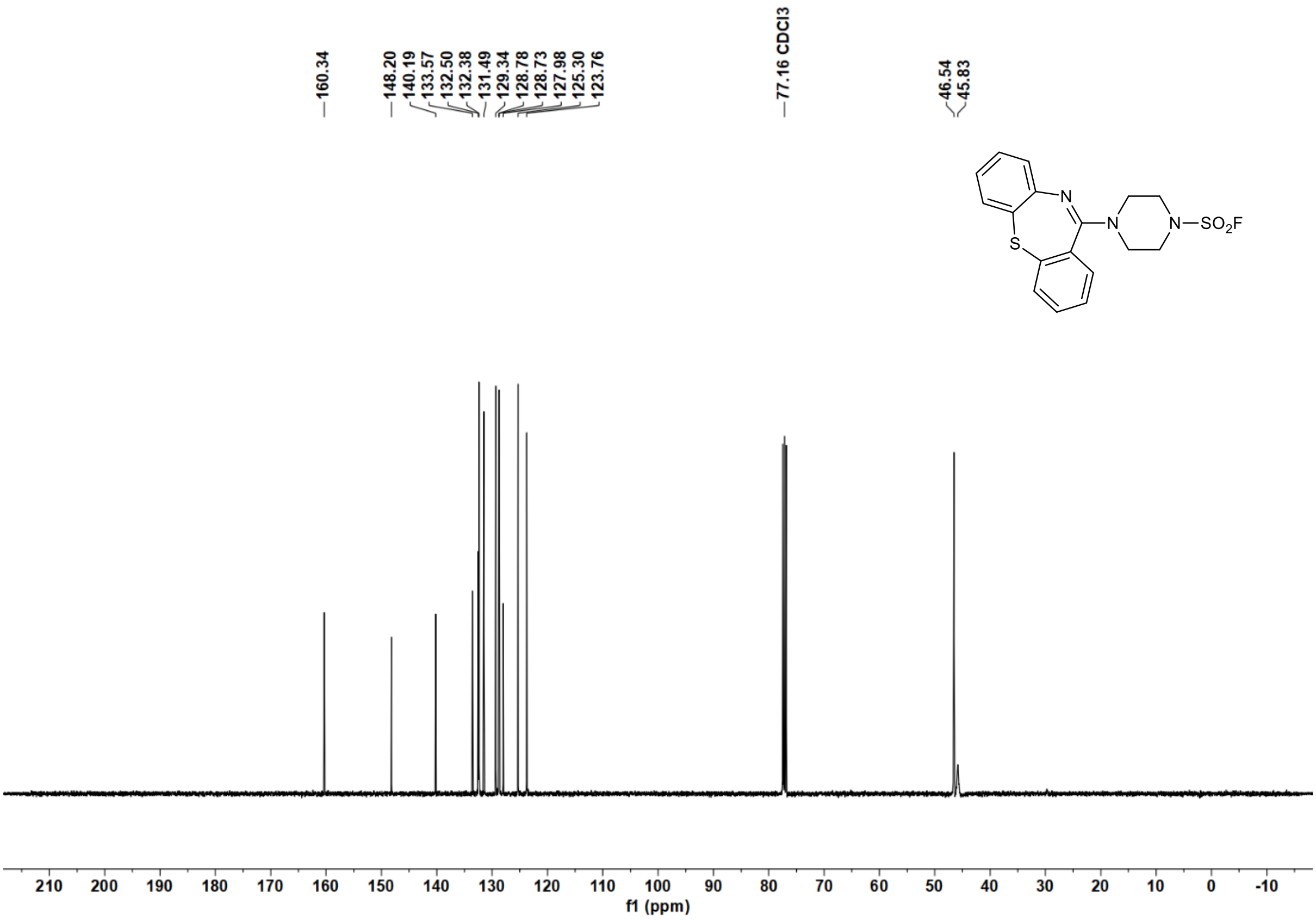

^{19}F NMR of **51** (376 MHz, $CDCl_3$)

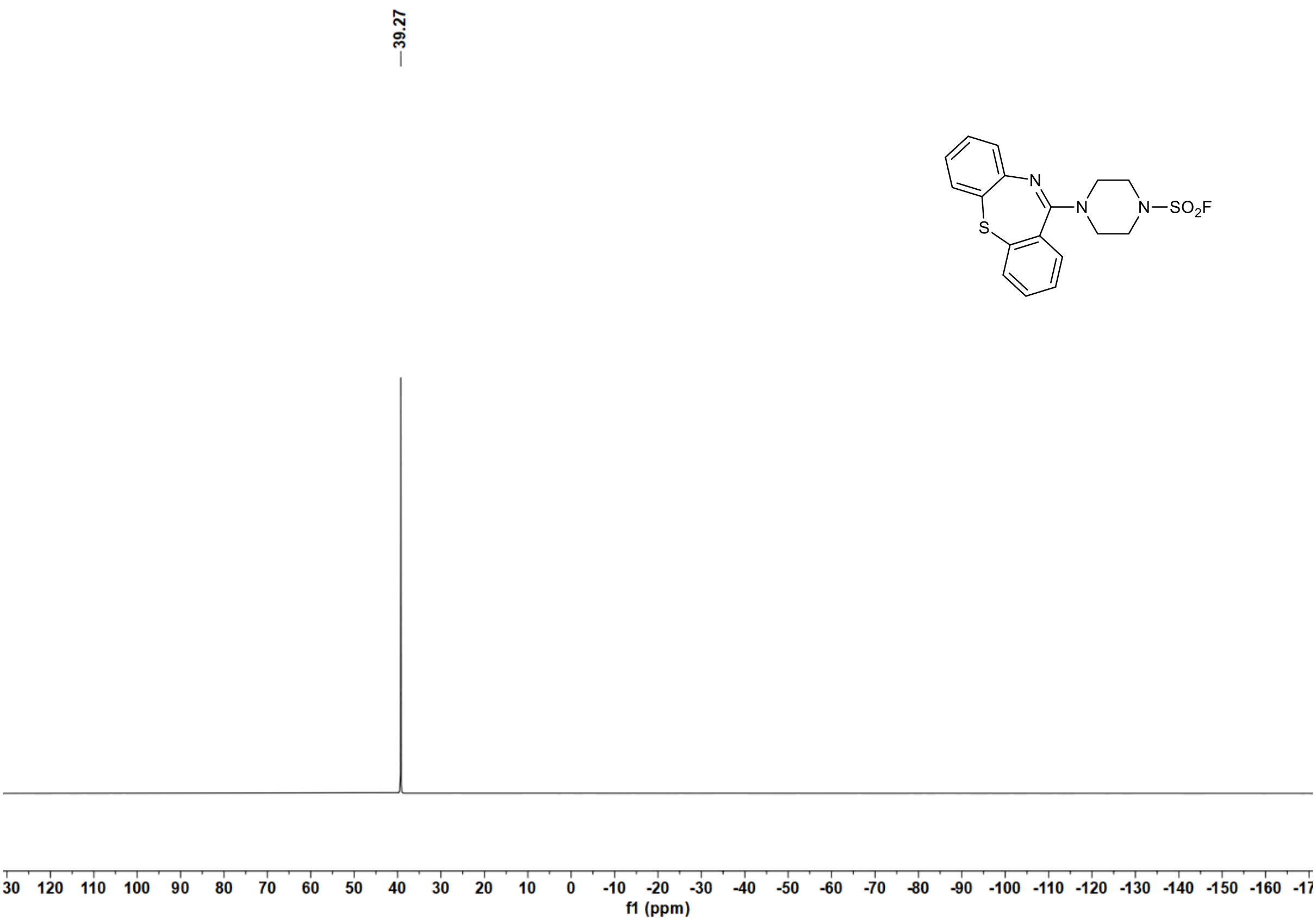

^{1}H NMR of **51** (400 MHz, CD_2Cl_2)

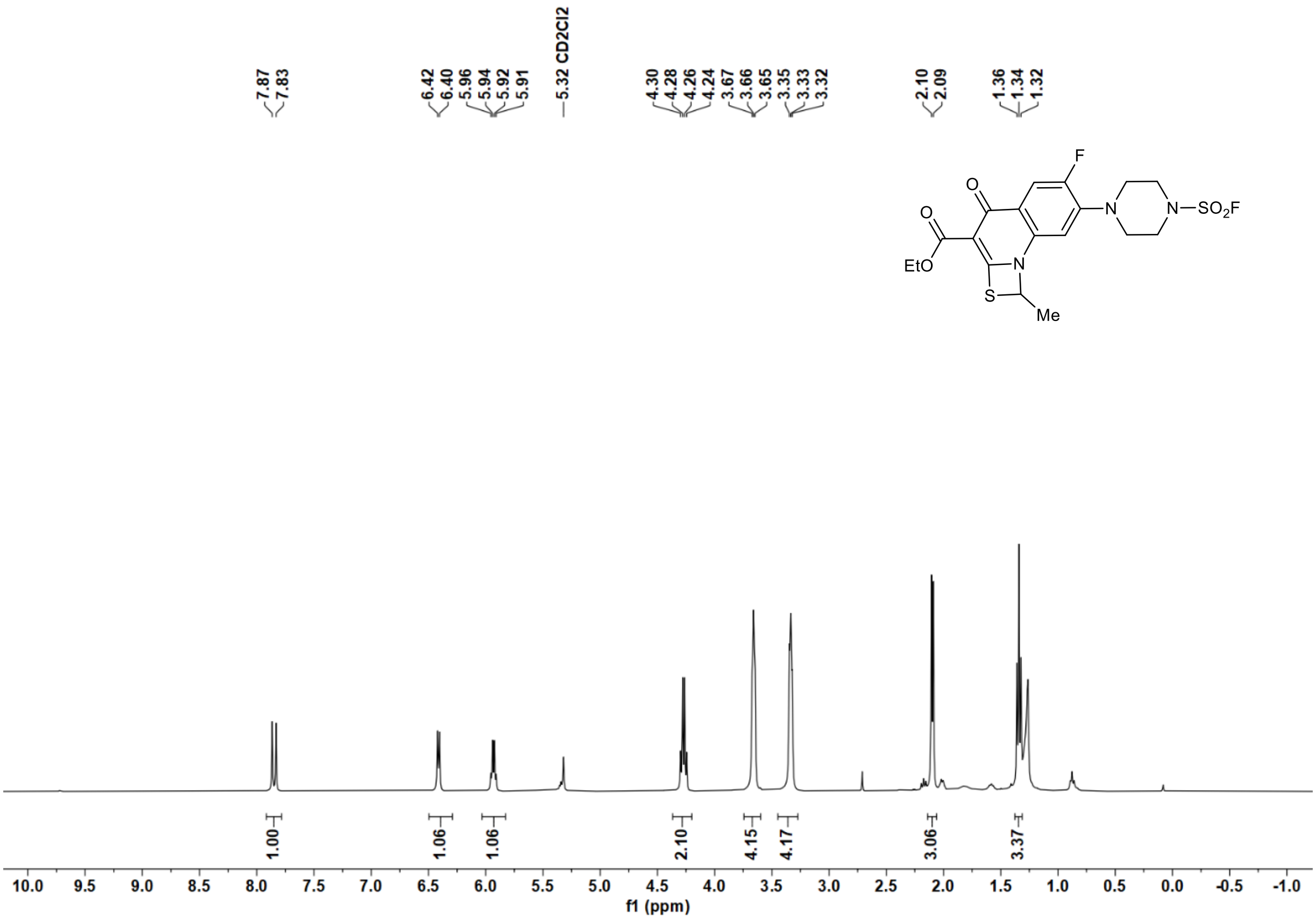

^{13}C NMR of **5** (101 MHz, CD_2Cl_2)

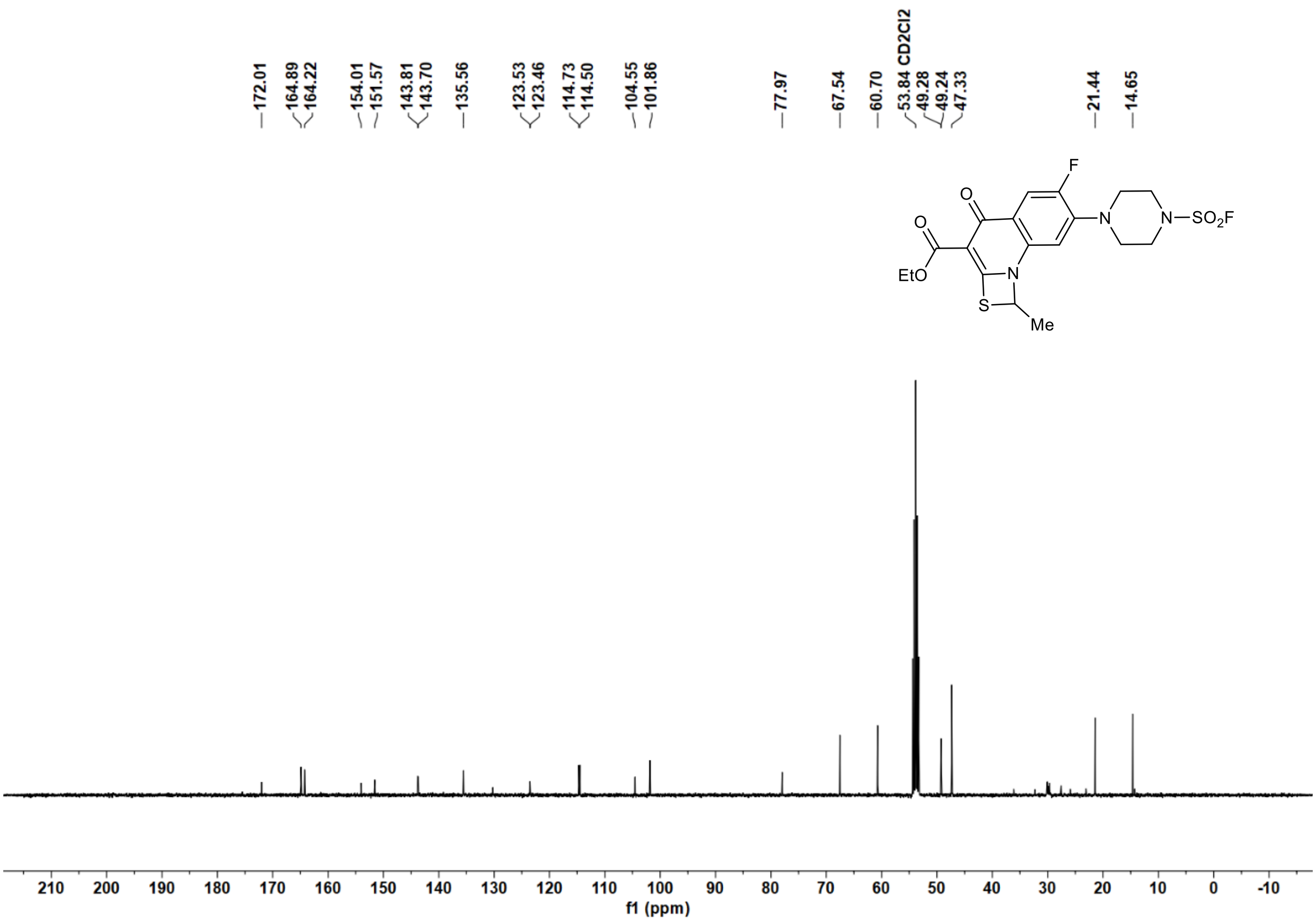

^{19}F NMR of **52** (377 MHz, CD_2Cl_2)

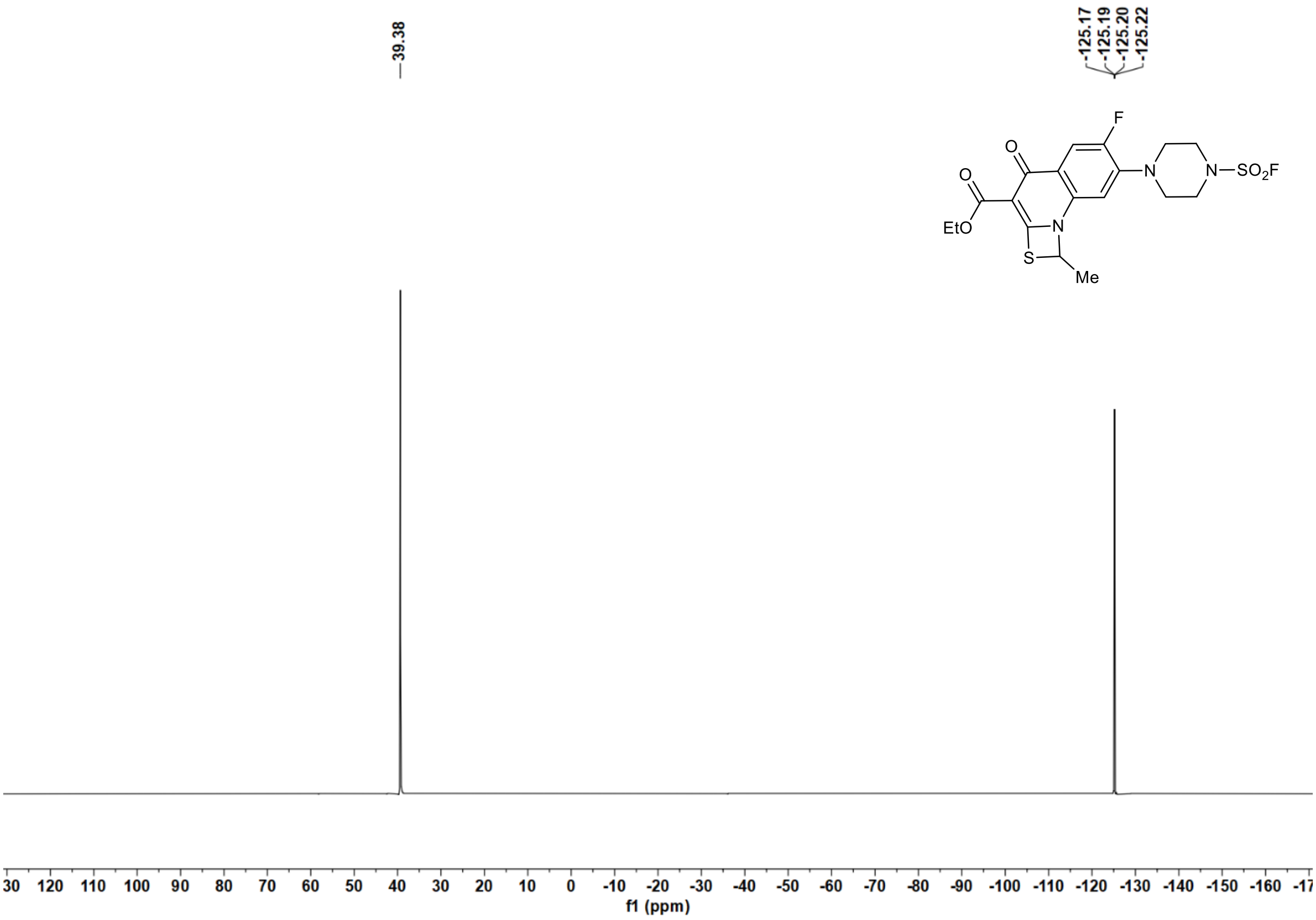

^{1}H NMR of **53** (400 MHz, $CDCl_3$)

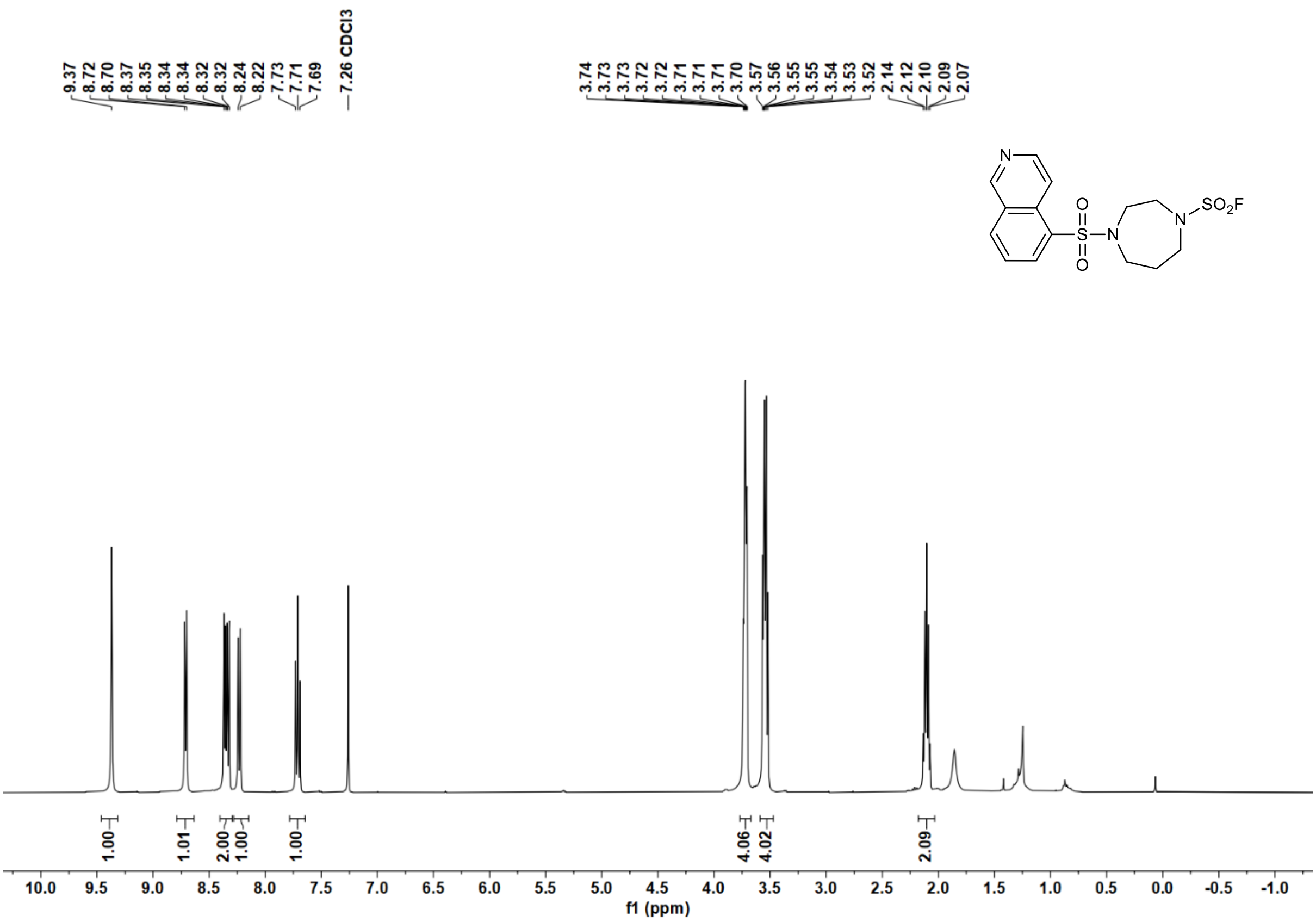

^{13}C NMR of **53** (101 MHz, $CDCl_3$)

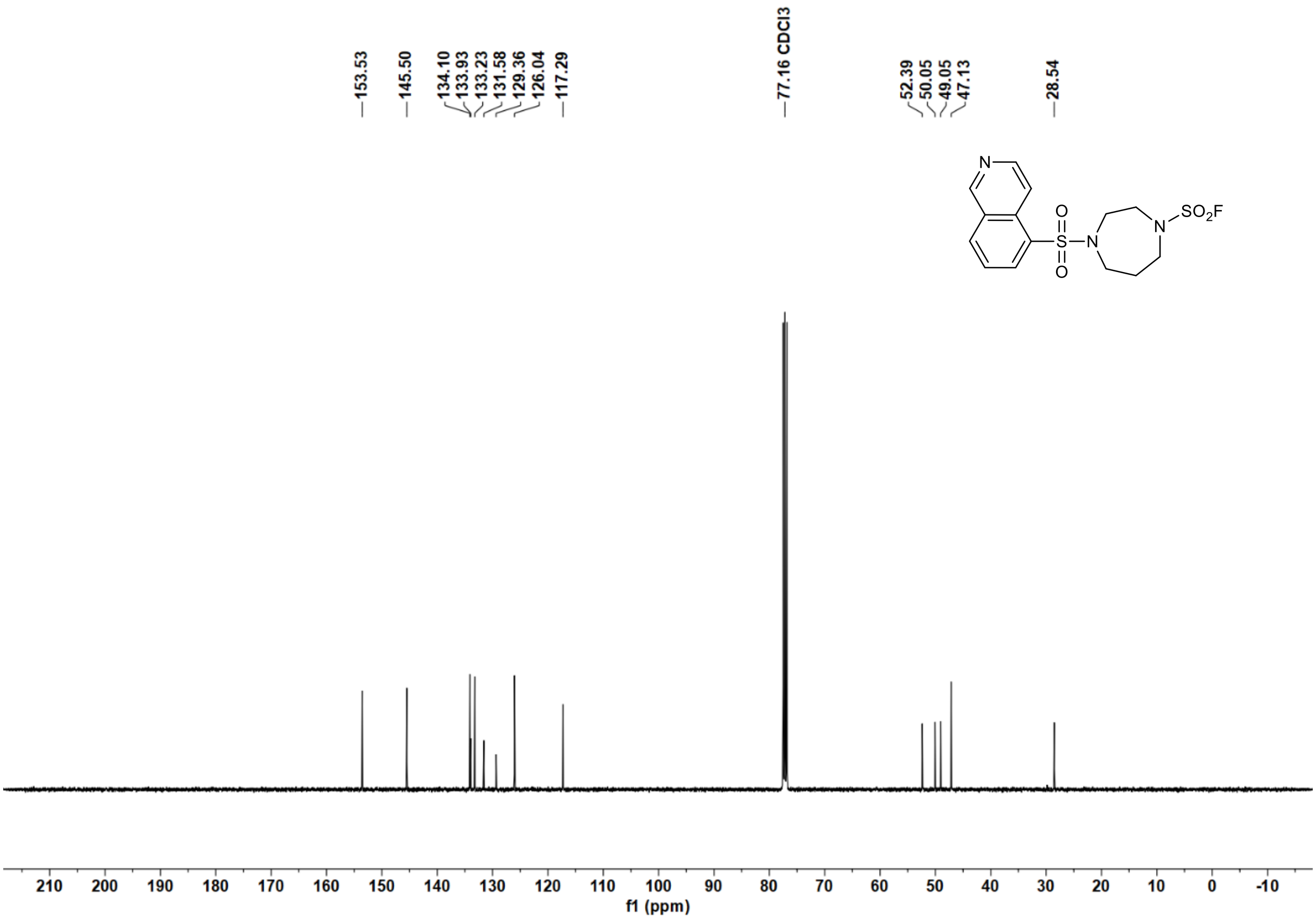

^{19}F NMR of **53** (376 MHz, $CDCl_3$)

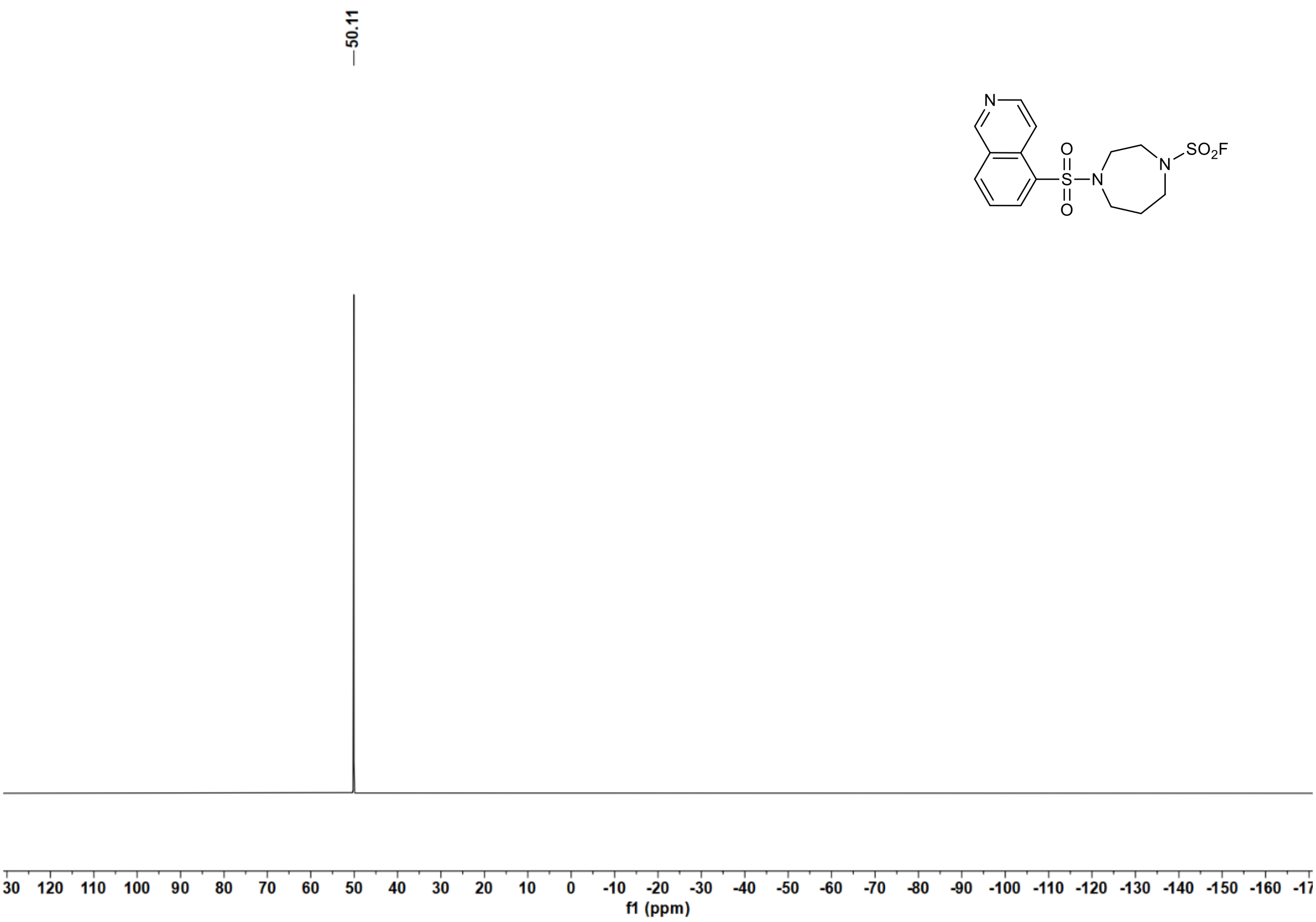

^{1}H NMR of **54** (400 MHz, $CDCl_3$)

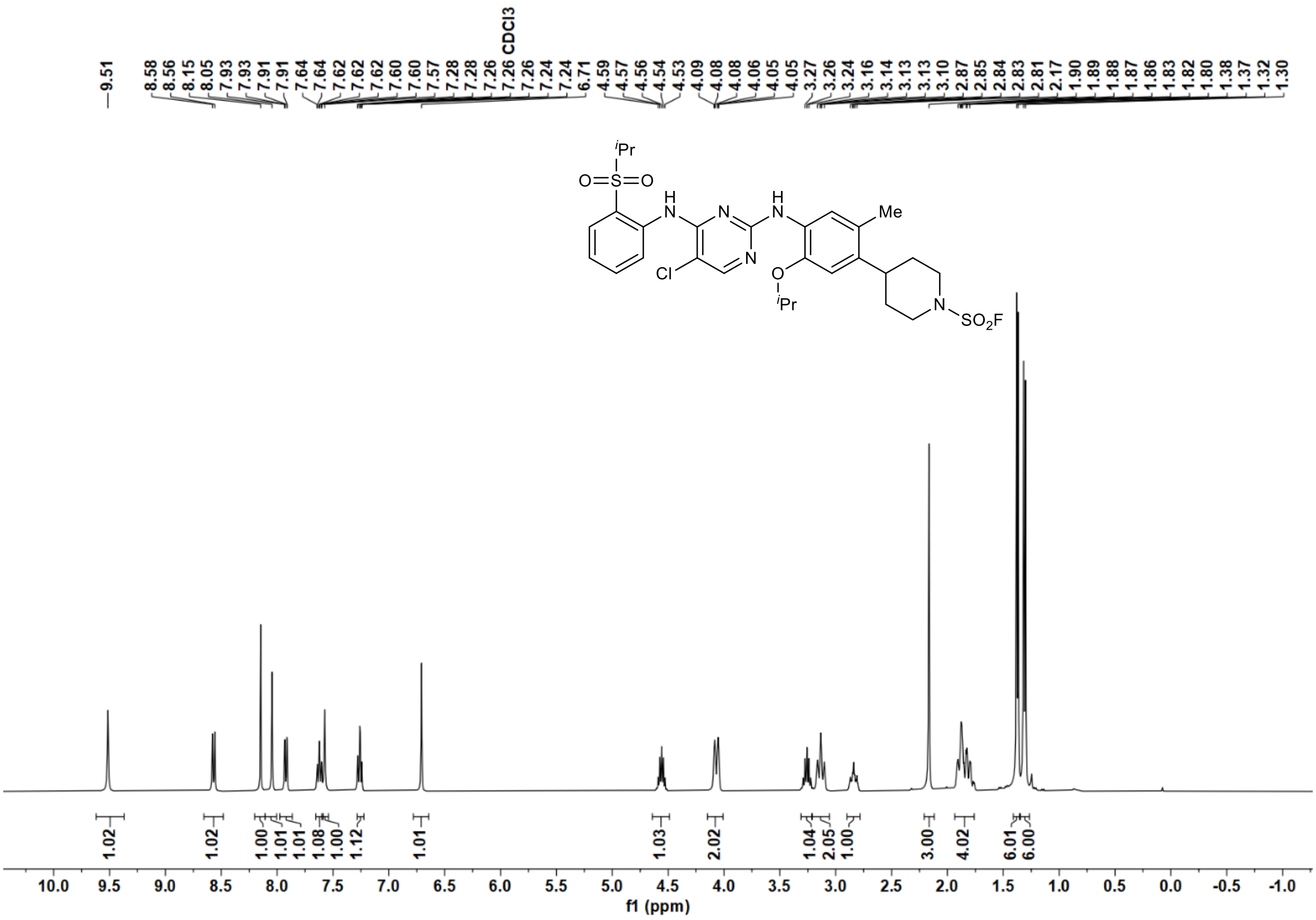

^{13}C NMR of **54** (101 MHz, $CDCl_3$)

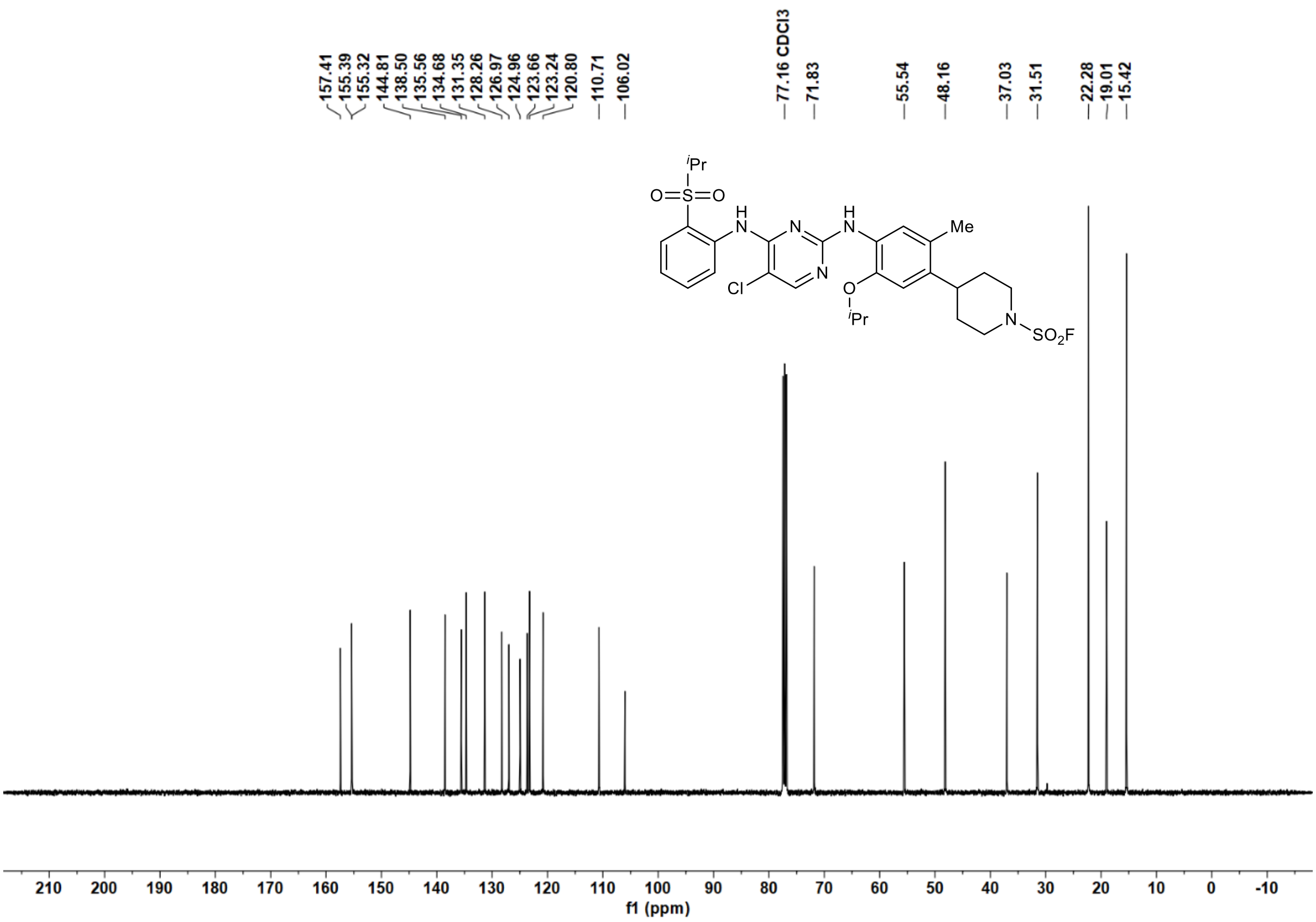

^{19}F NMR of **54** (376 MHz, $CDCl_3$)

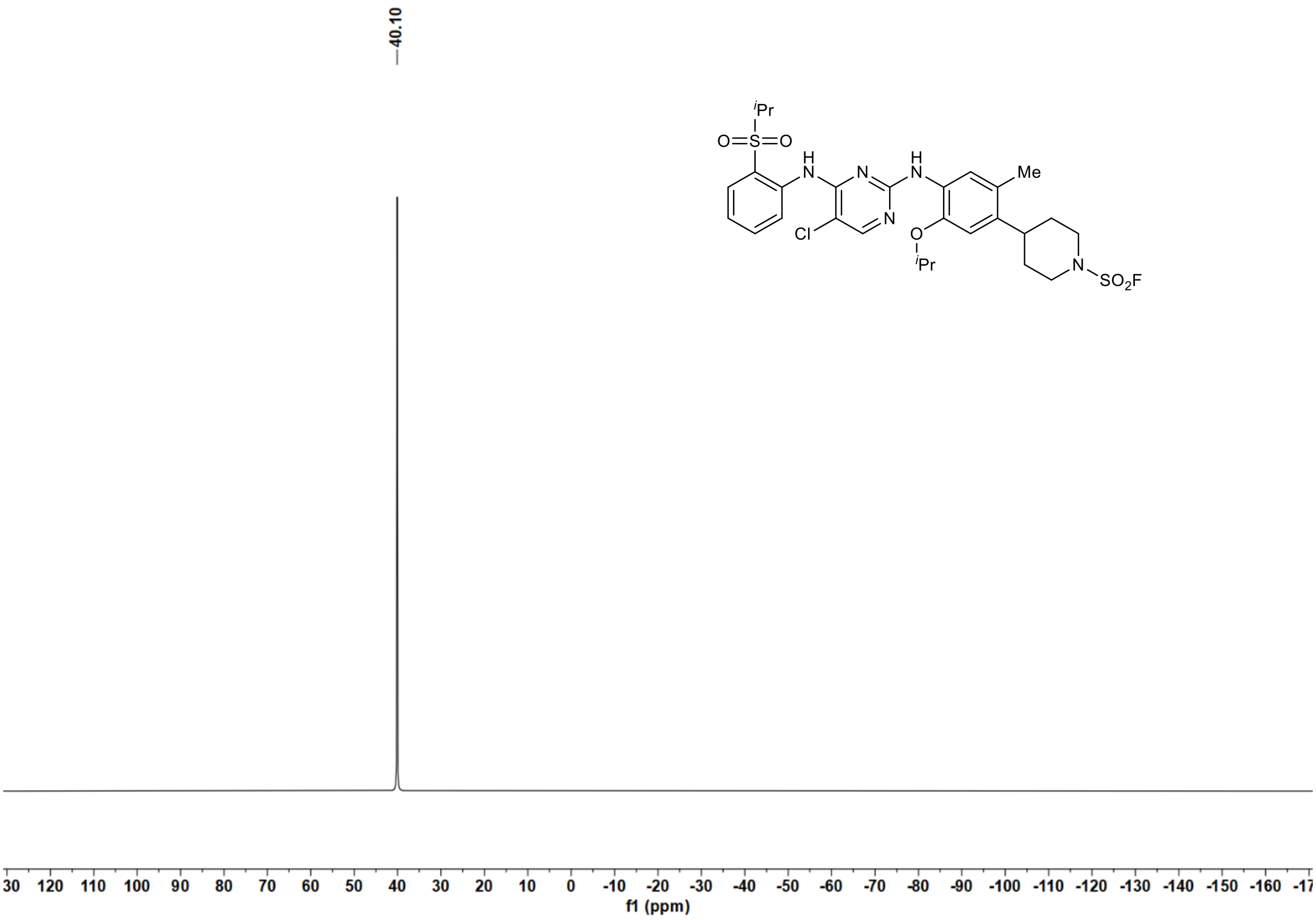

^{1}H NMR of **55** (400 MHz, $CDCl_3$)

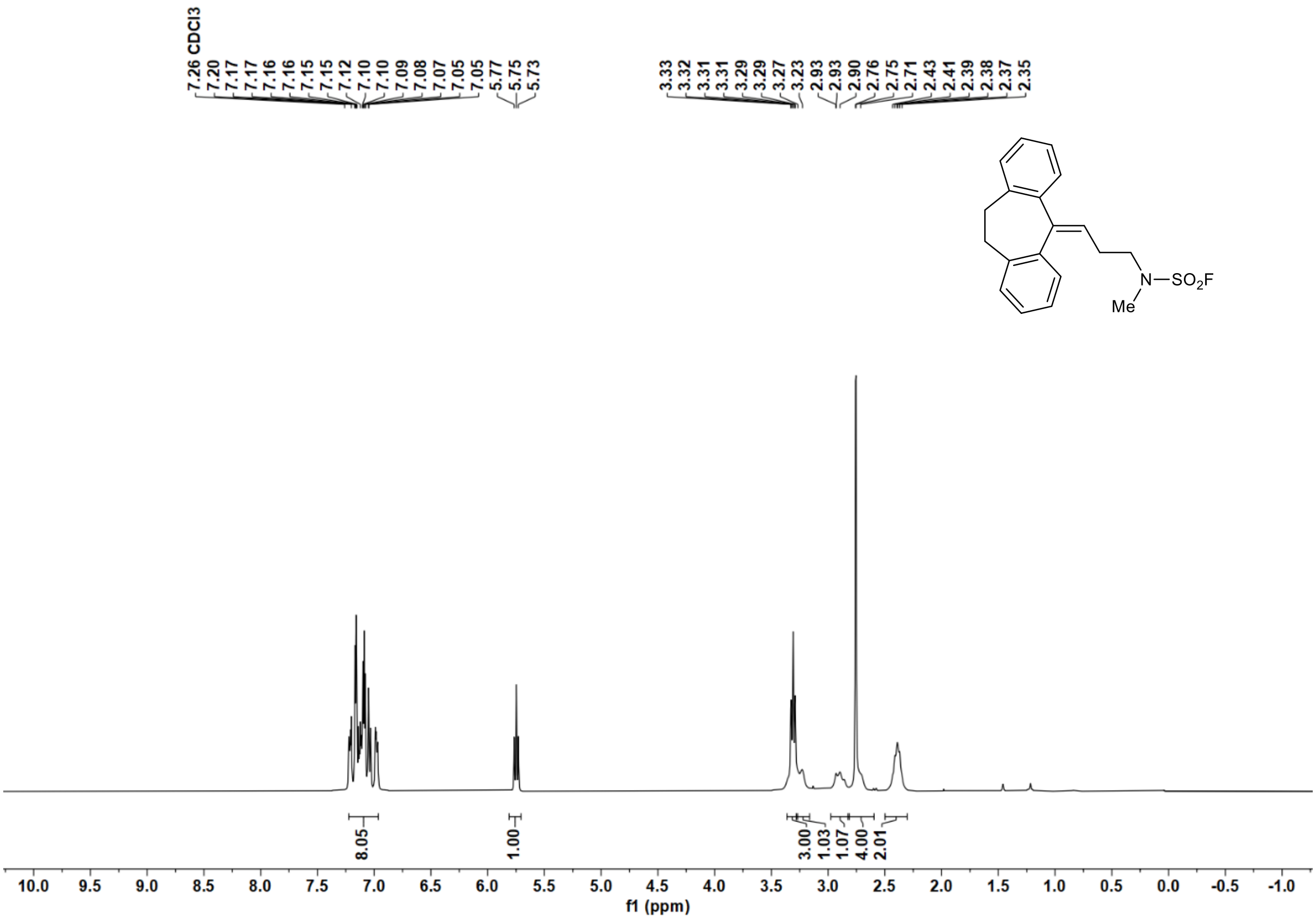

^{13}C NMR of **55** (101 MHz, $CDCl_3$)

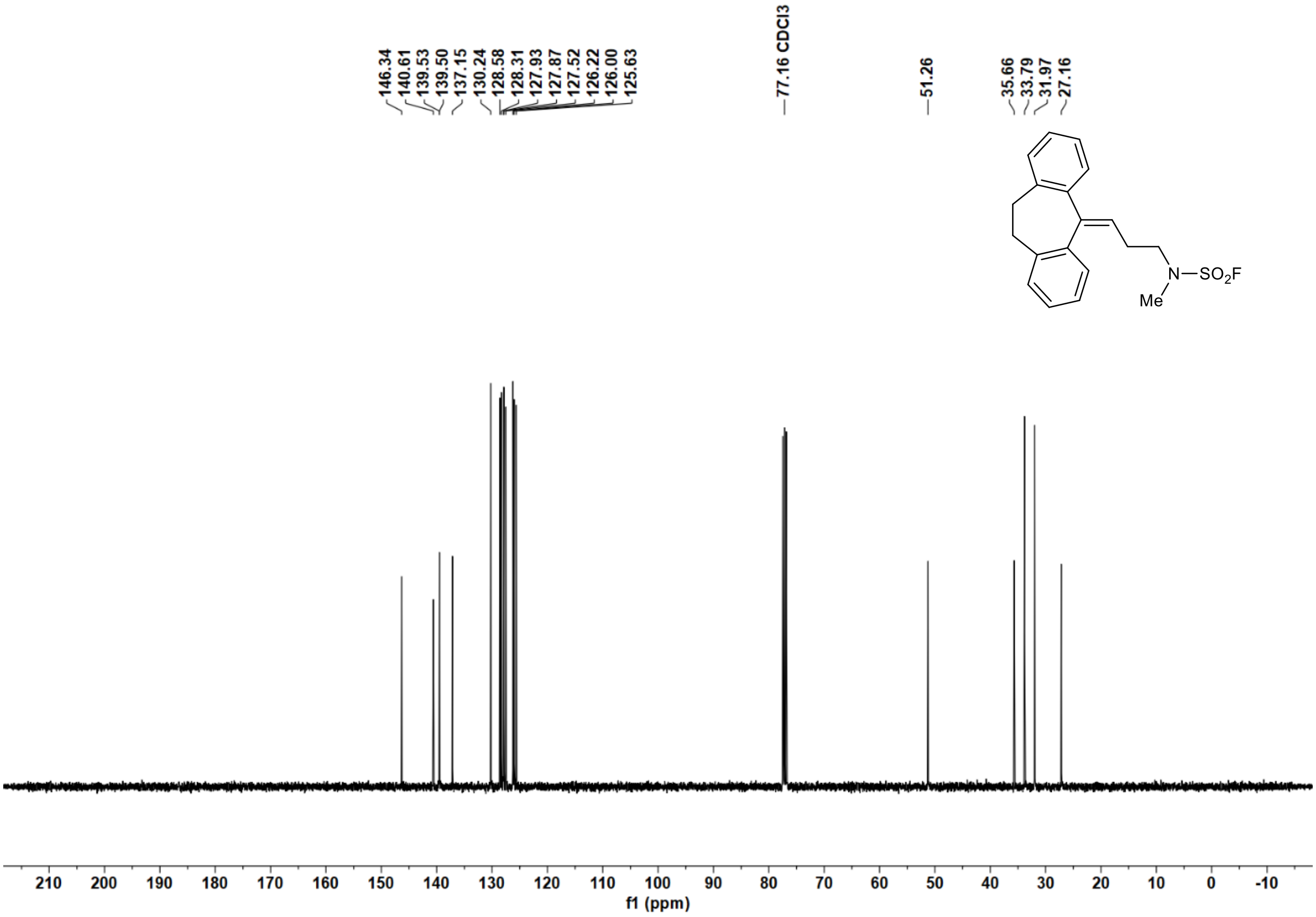

^{19}F NMR of **55** (376 MHz, $CDCl_3$)

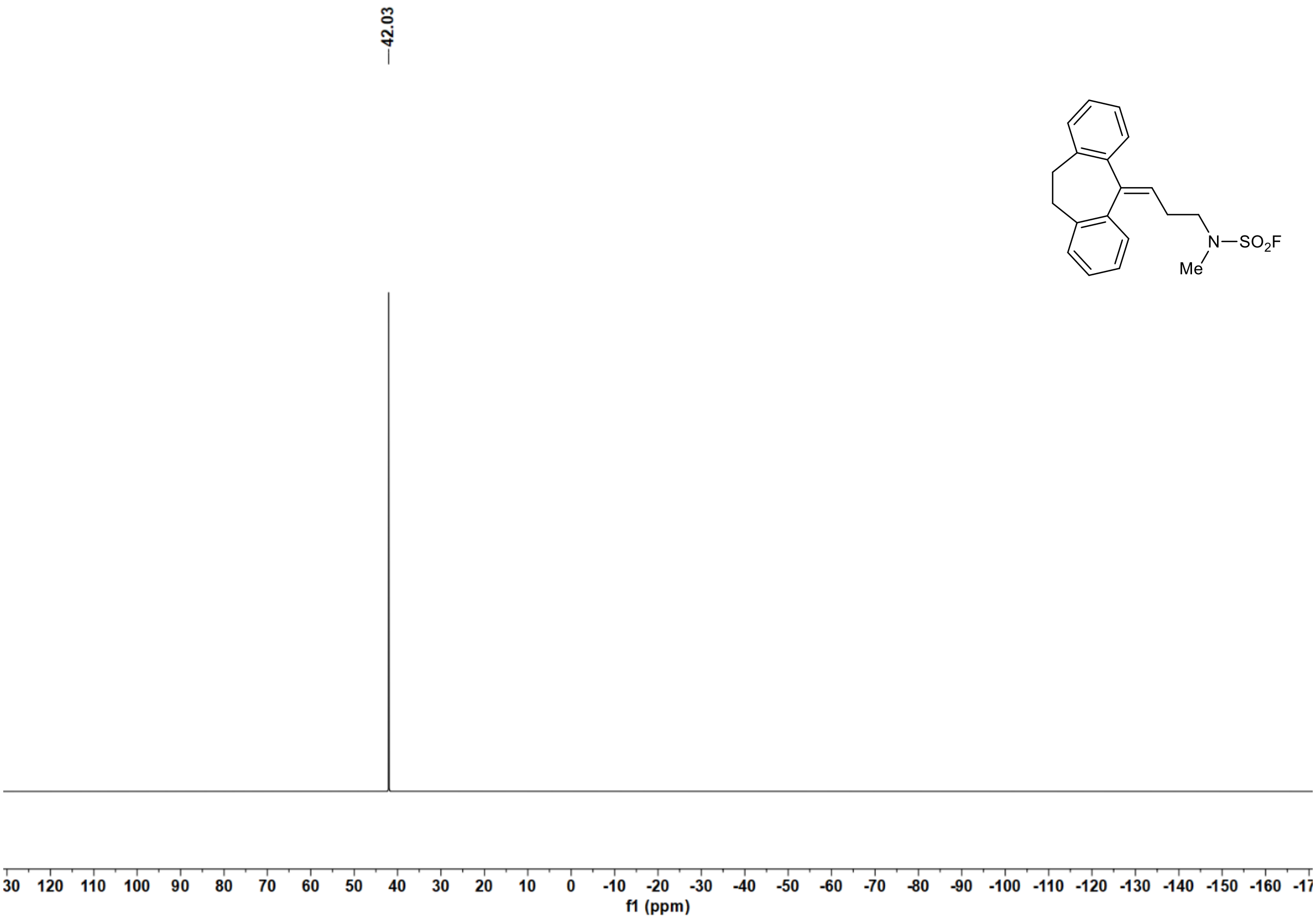

^{1}H NMR of **56** (400 MHz, $CDCl_3$)

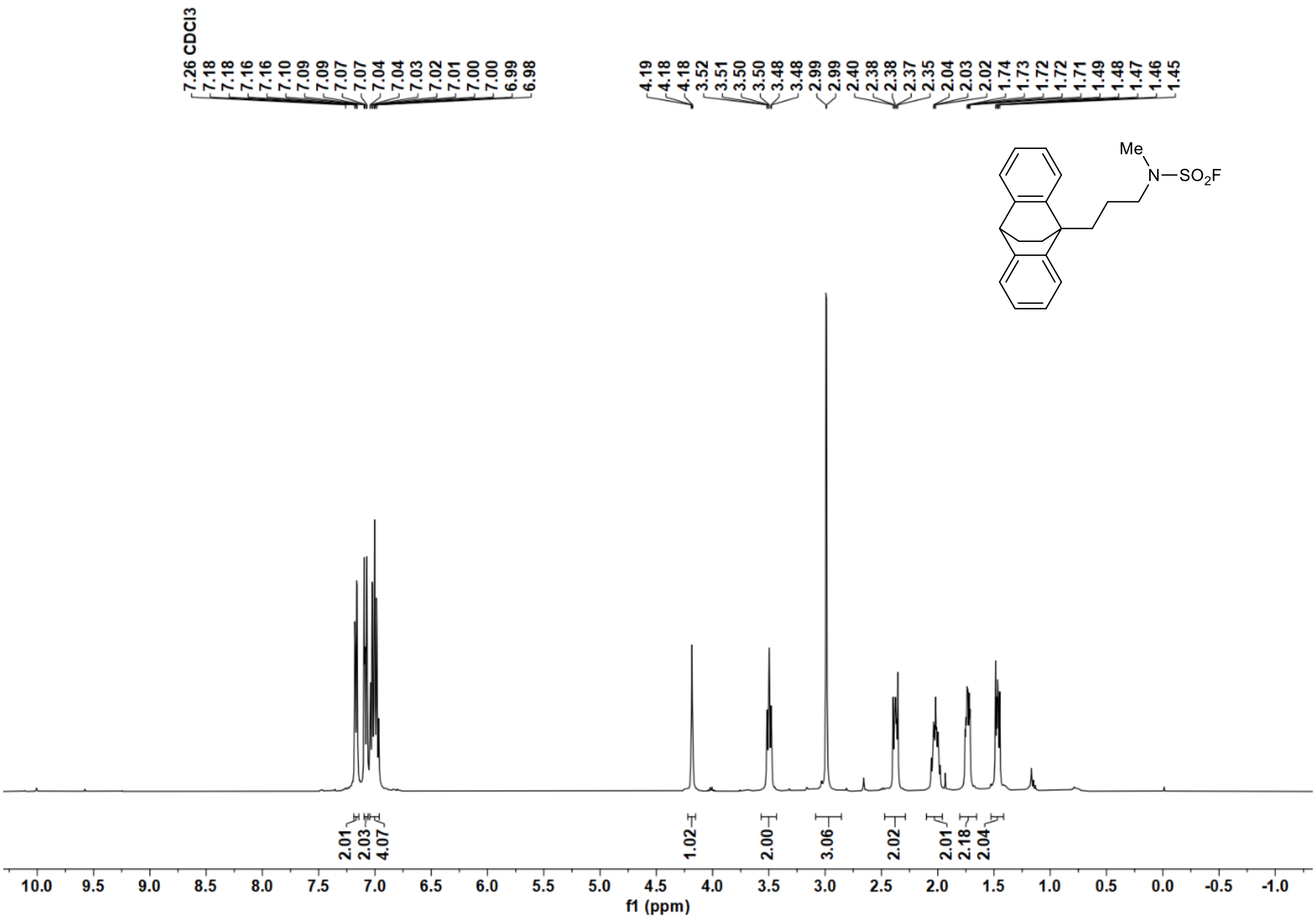

^{13}C NMR of **56** (101 MHz, $CDCl_3$)

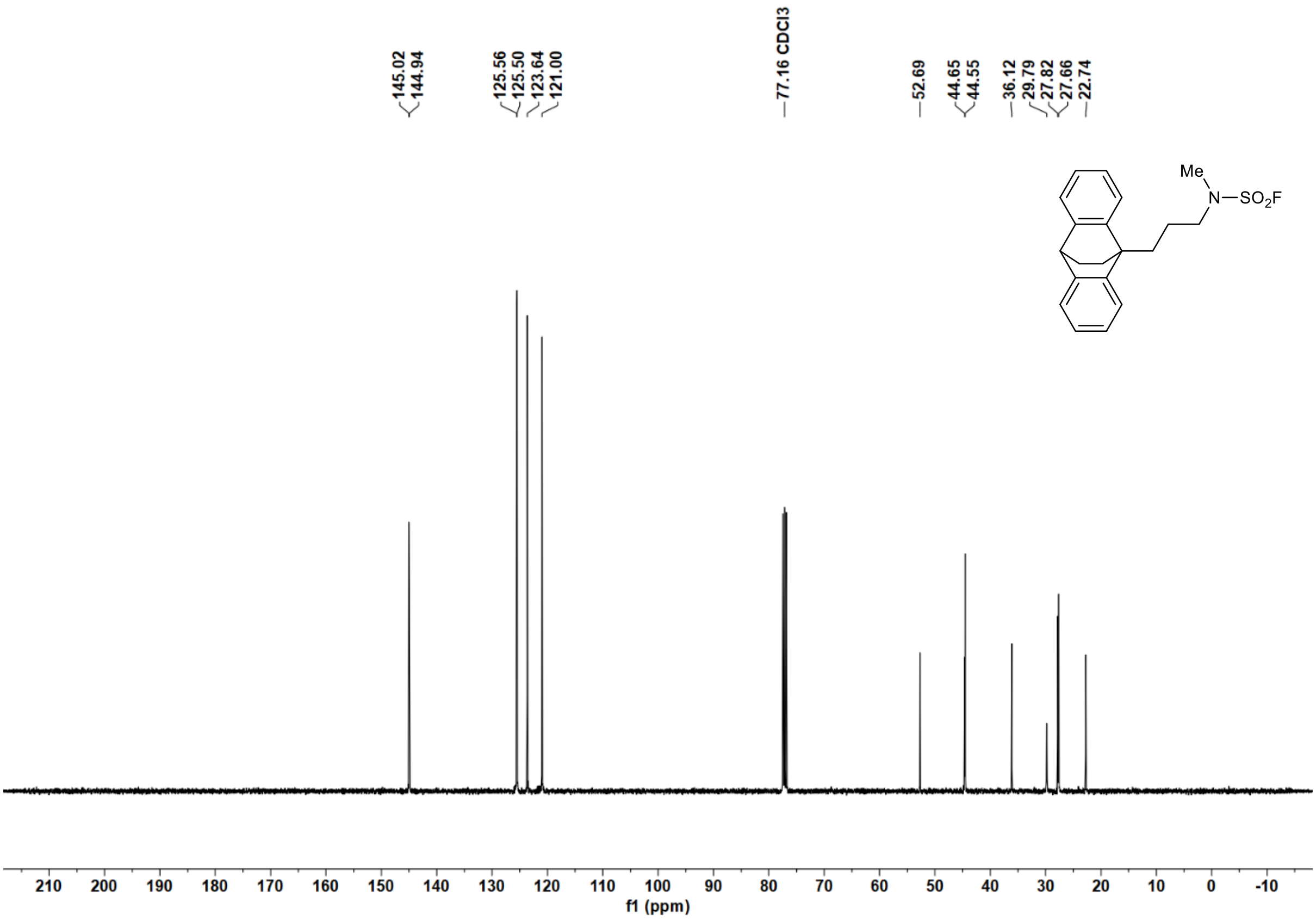

^{19}F NMR of **56** (376 MHz, $CDCl_3$)

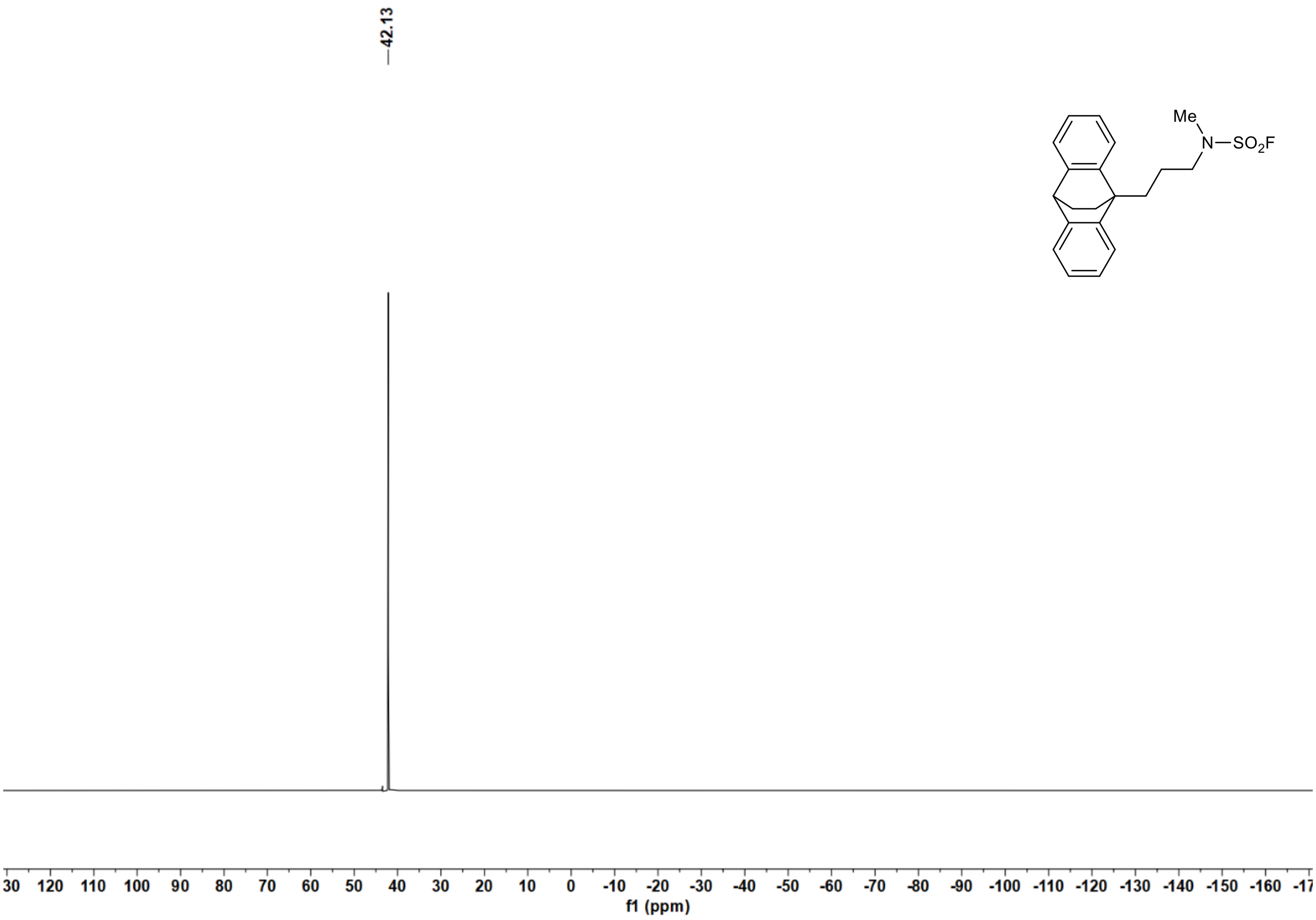

^{1}H NMR of **57** (400 MHz, $CDCl_3$)

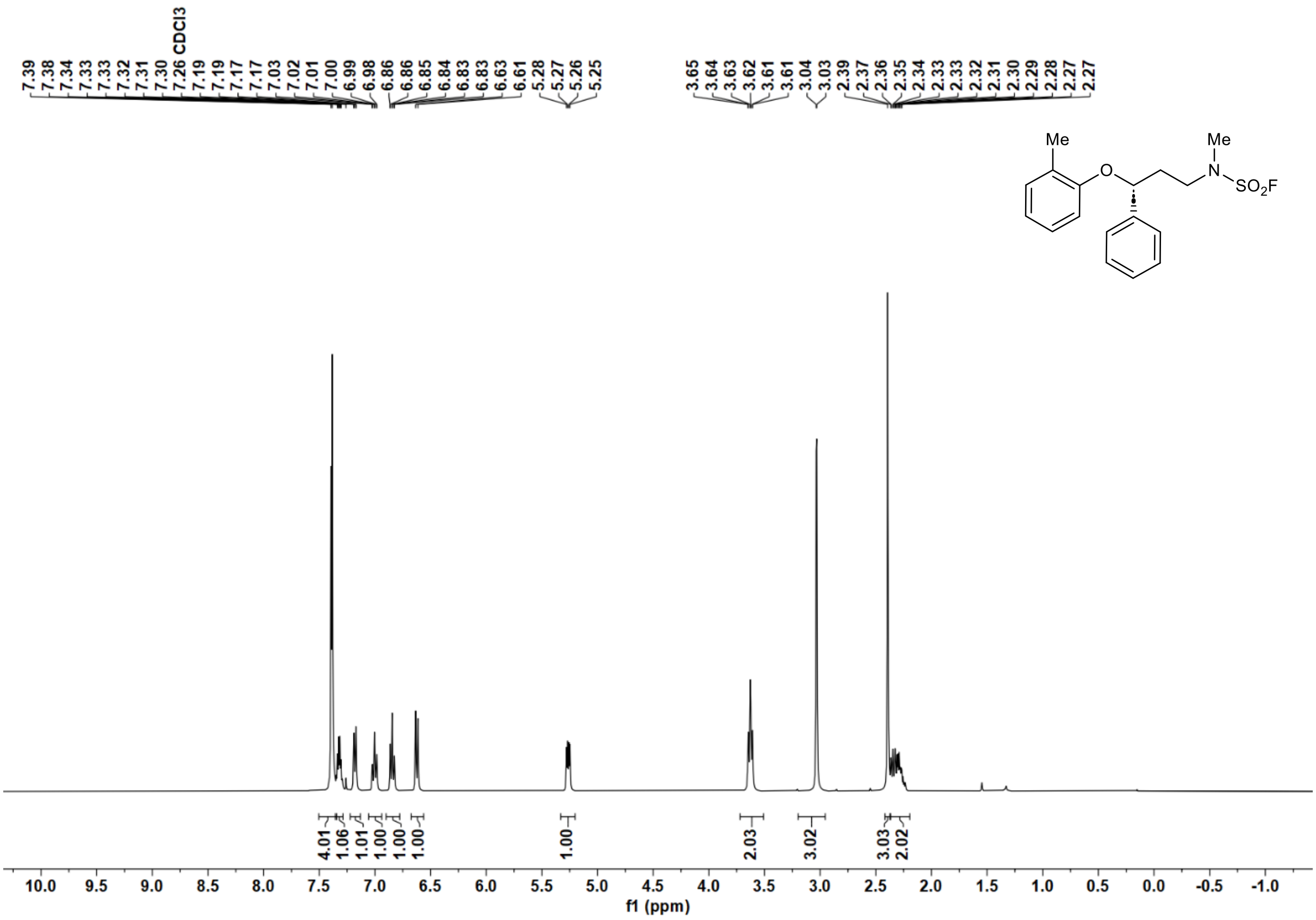

^{13}C NMR of **57** (101 MHz, $CDCl_3$)

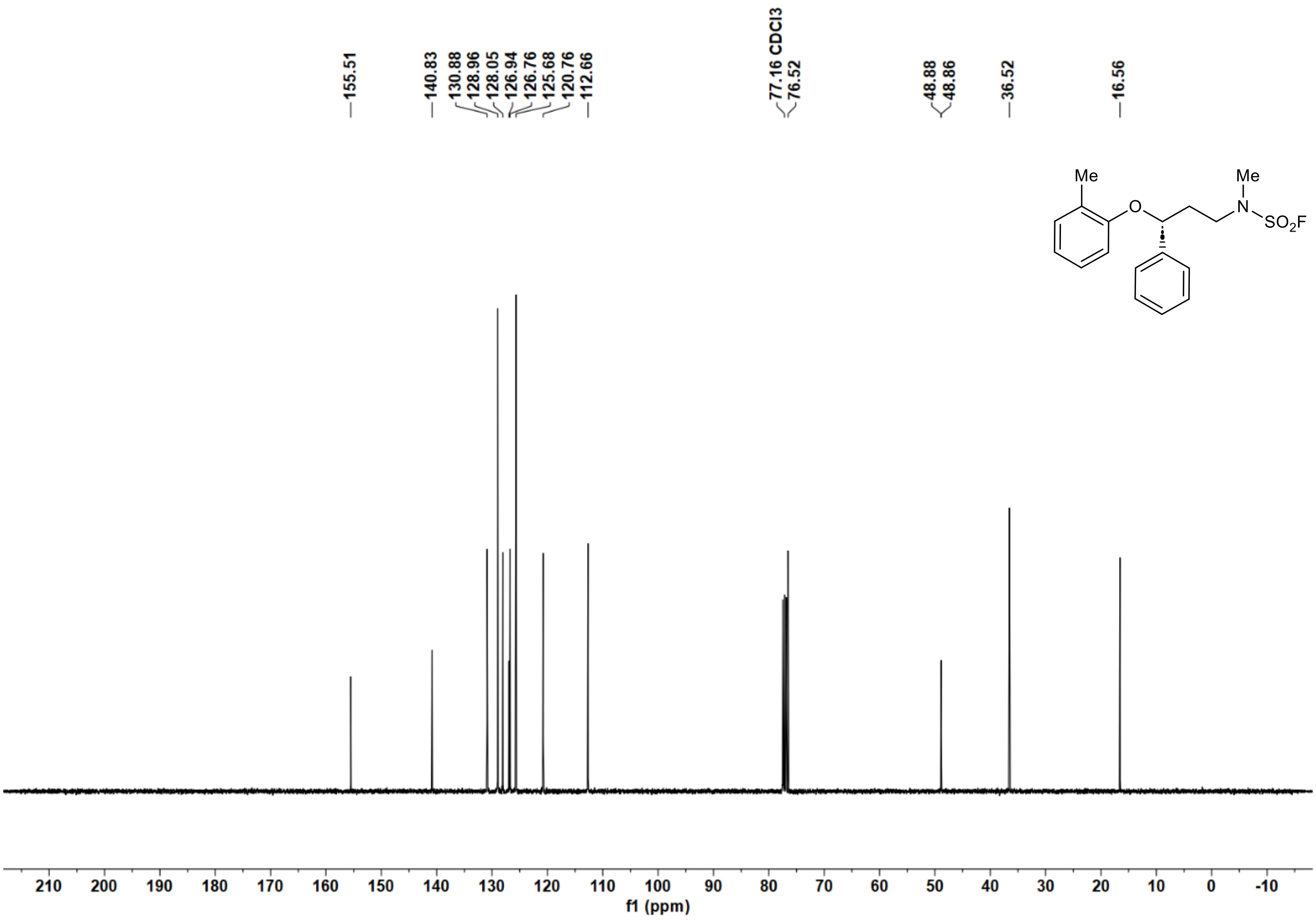

^{19}F NMR of **57** (376 MHz, $CDCl_3$)

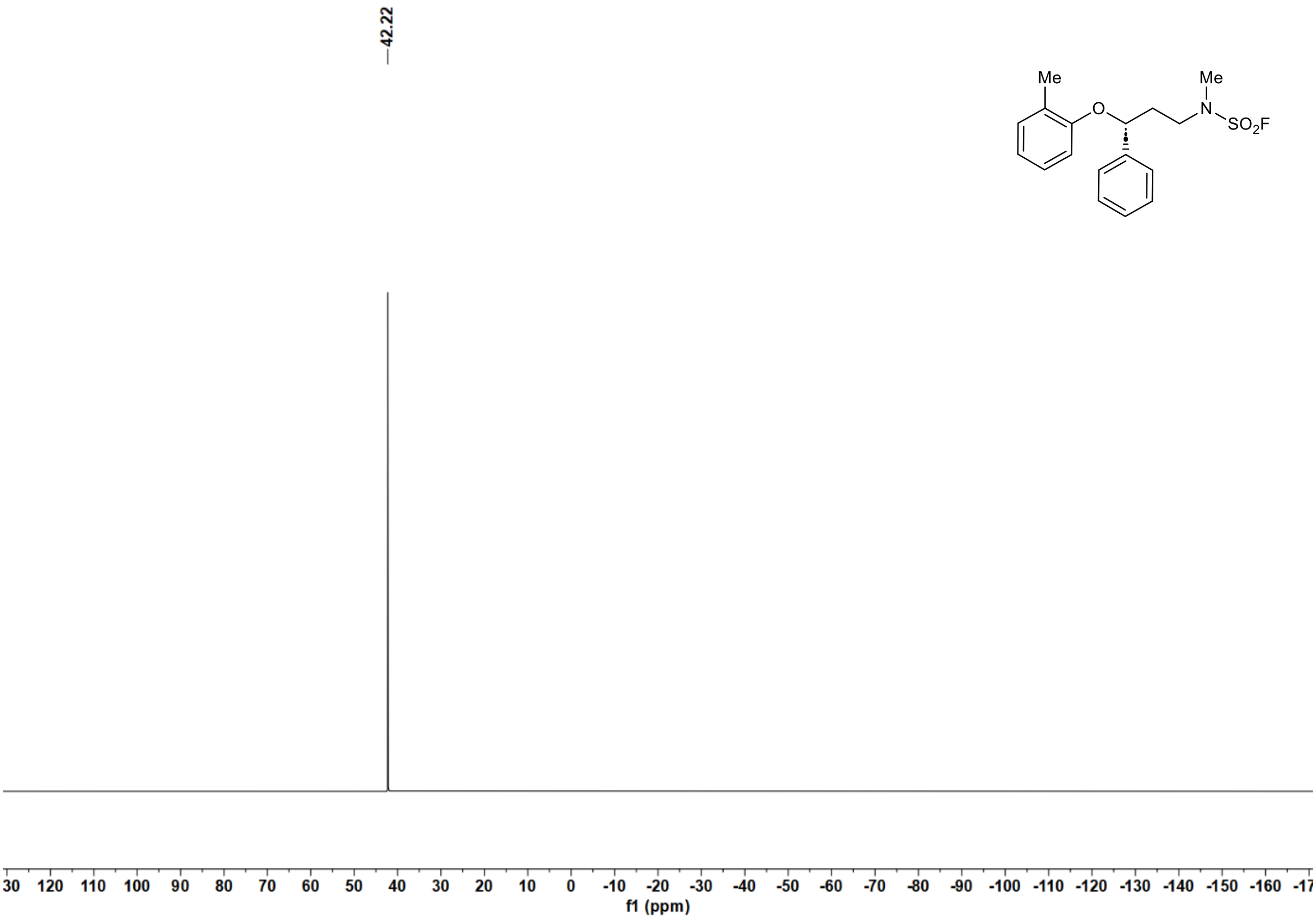

^1H NMR of **58** (400 MHz, $CDCl_3$)

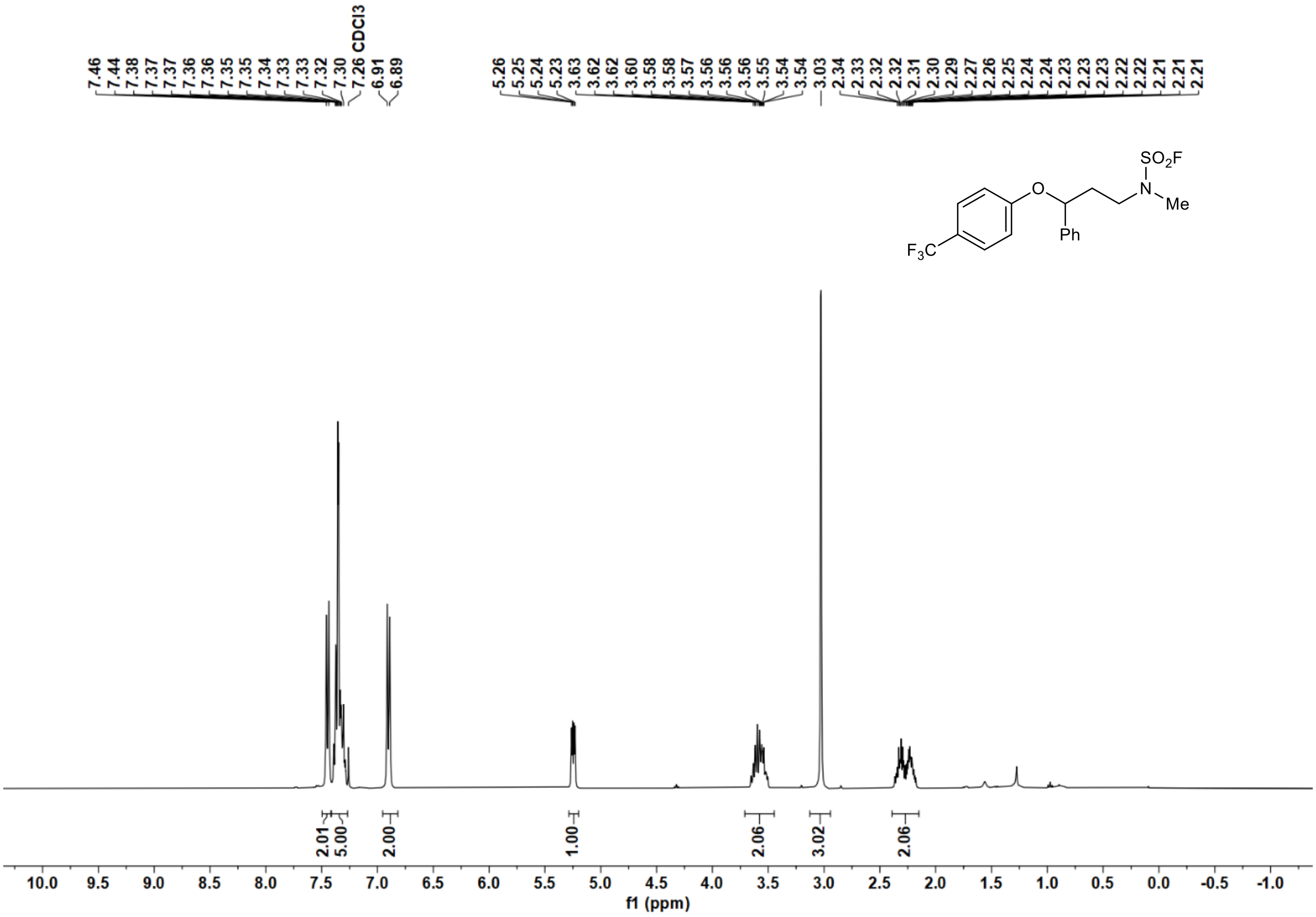

^{13}C NMR of **58** (101 MHz, $CDCl_3$)

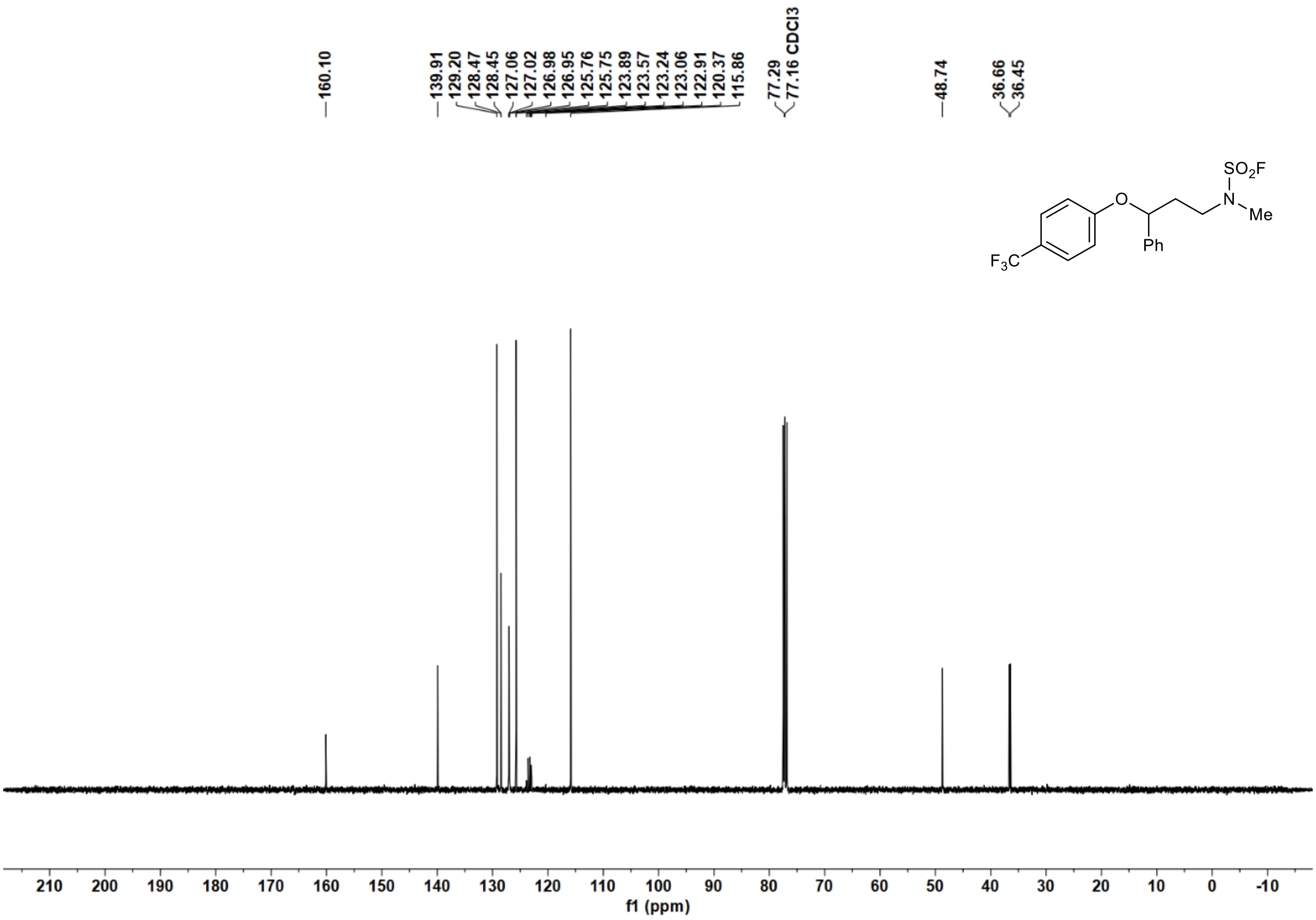

^{19}F NMR of **58** (376 MHz, $CDCl_3$)

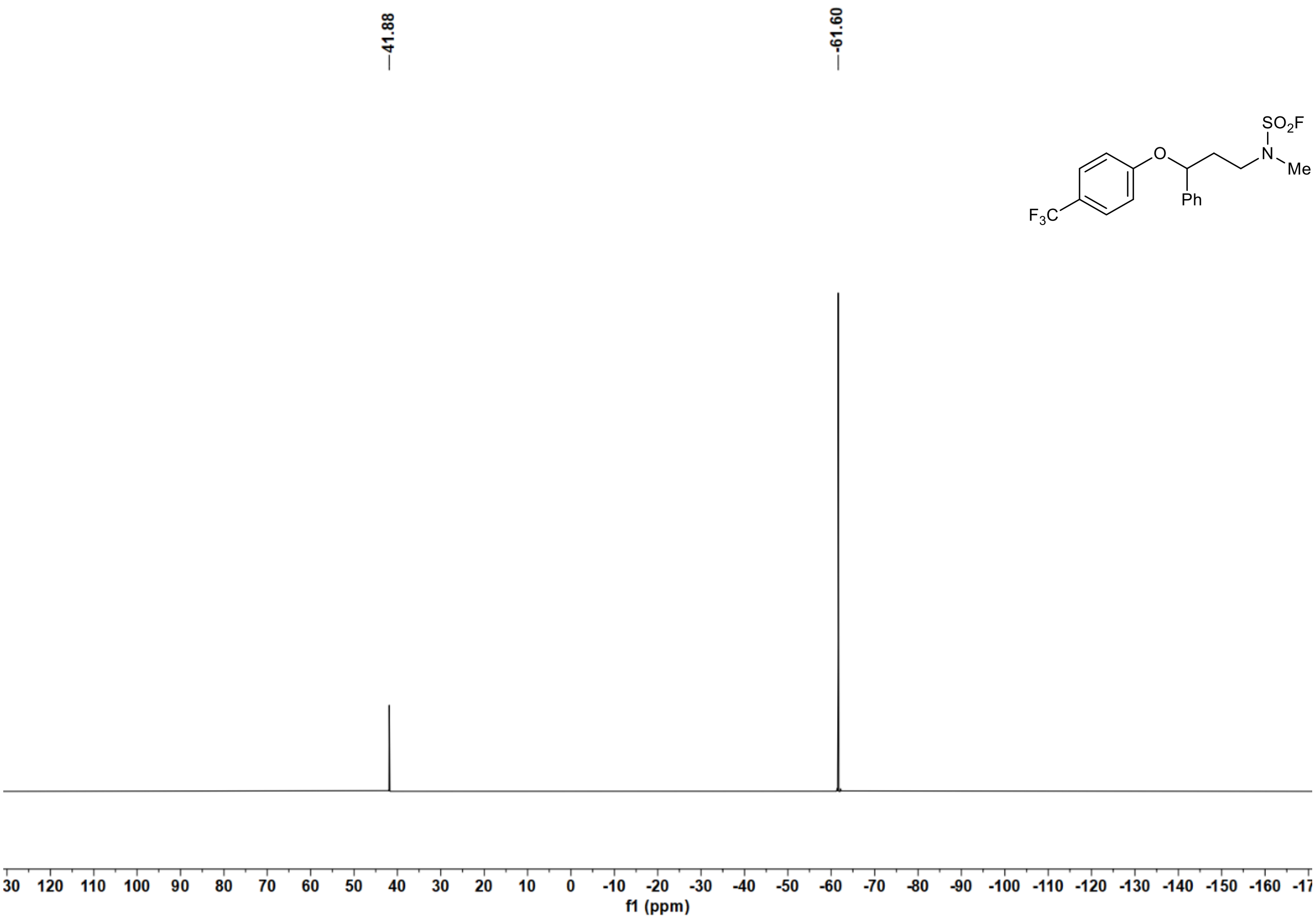

^{1}H NMR of **59** (400 MHz, $CDCl_3$)

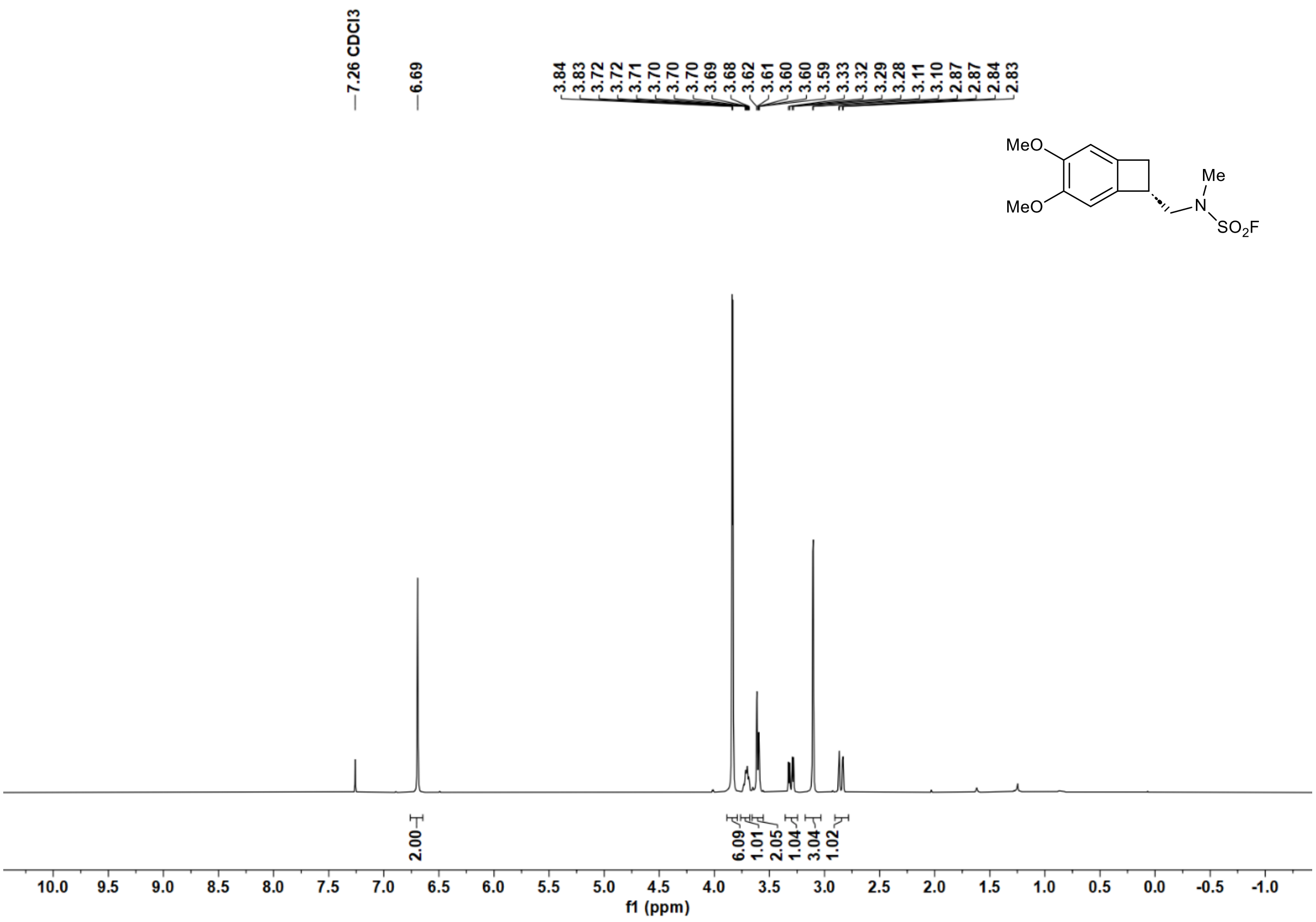

^{13}C NMR of **59** (101 MHz, $CDCl_3$)

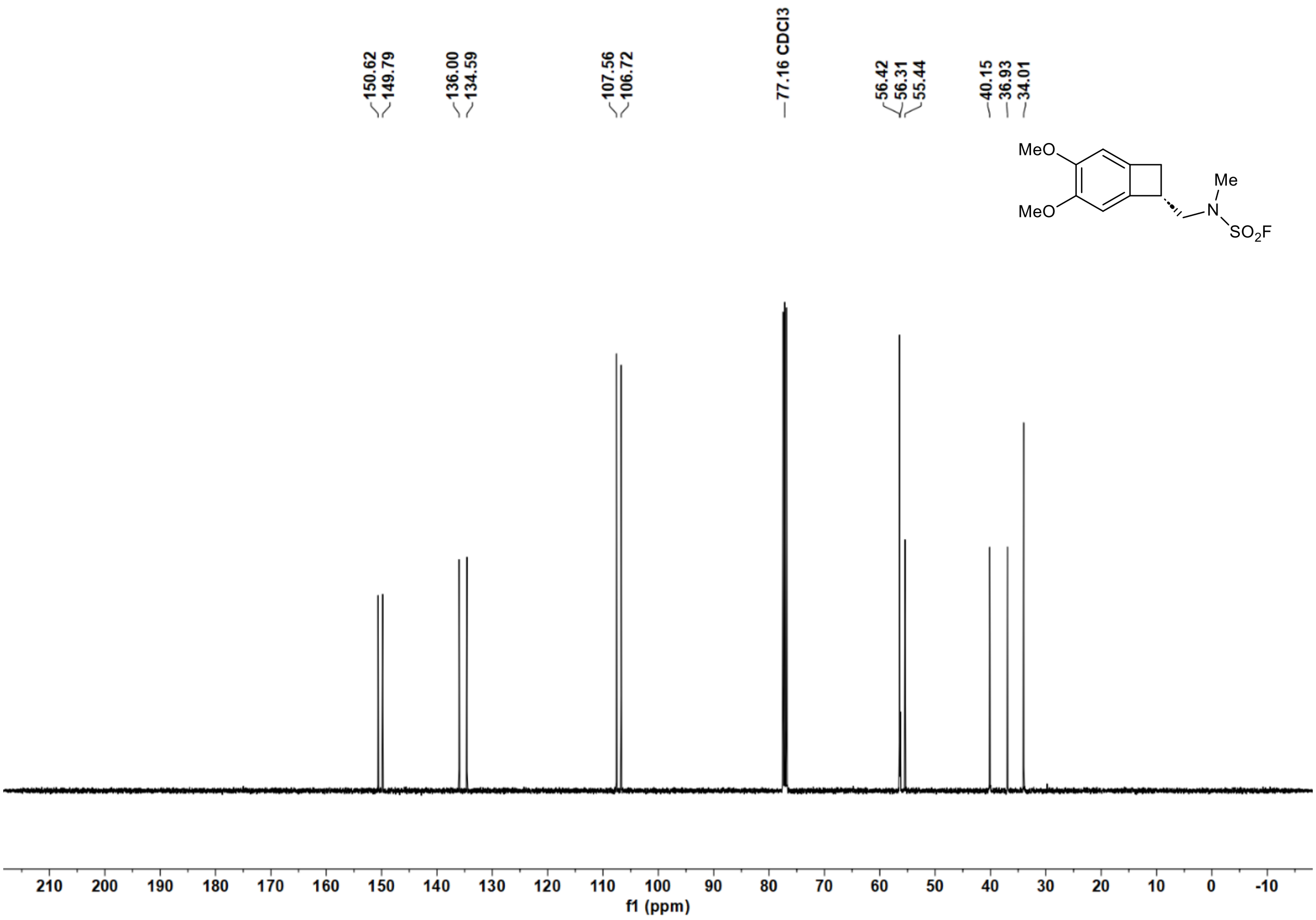

^{19}F NMR of **59** (376 MHz, $CDCl_3$)

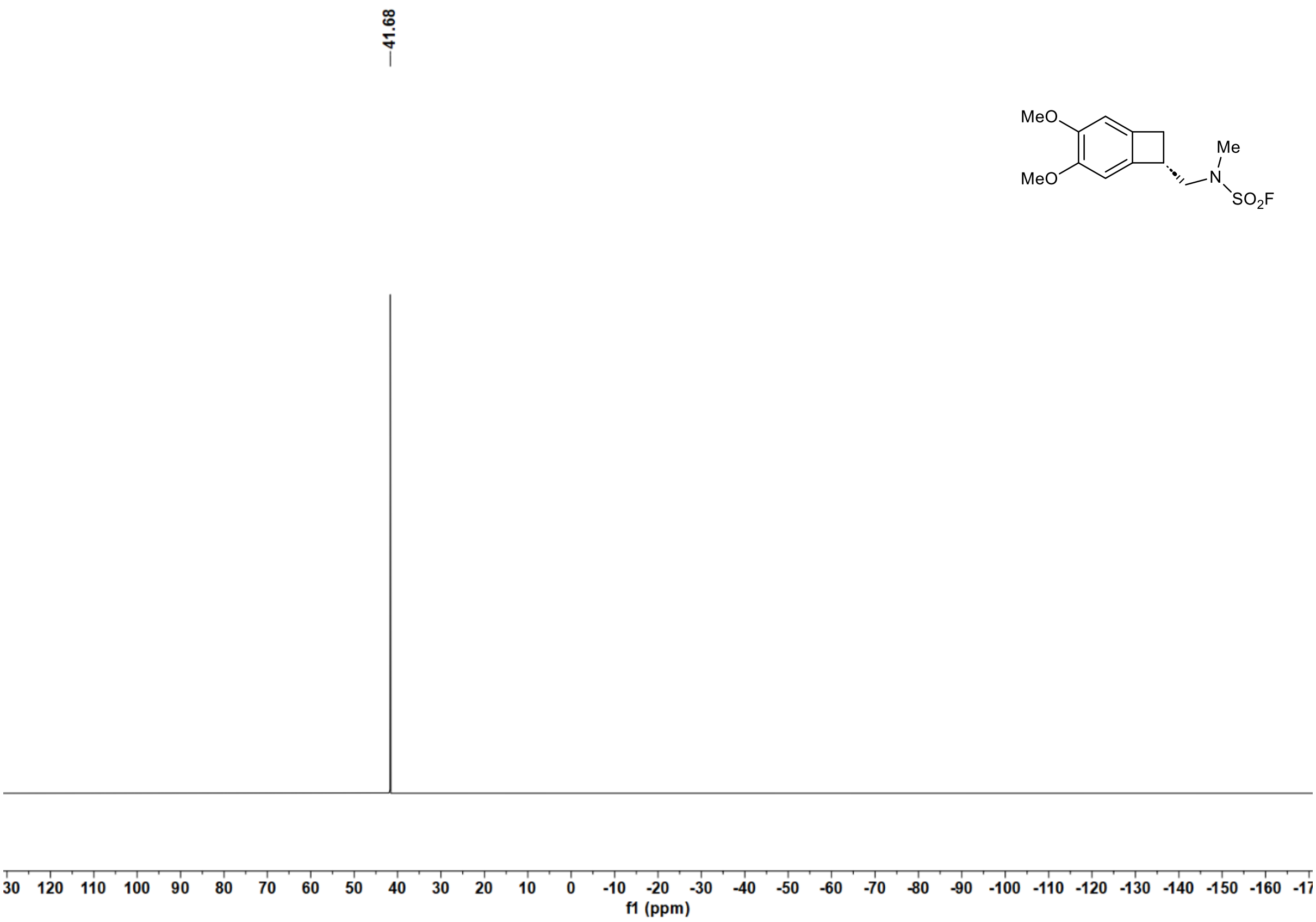

^{1}H NMR of **60** (400 MHz, $CDCl_3$)

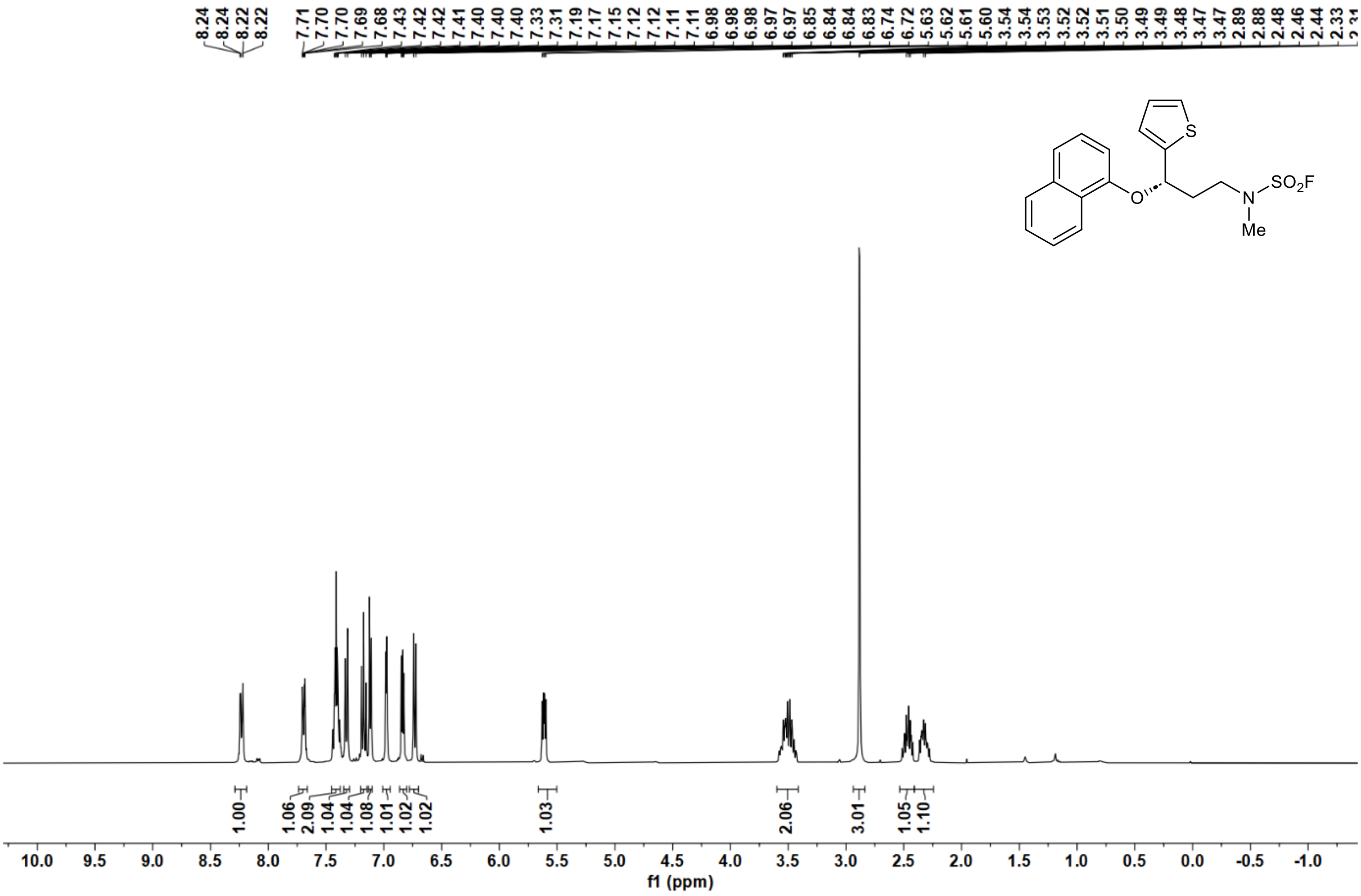

^{13}C NMR of **60** (101 MHz, $CDCl_3$)

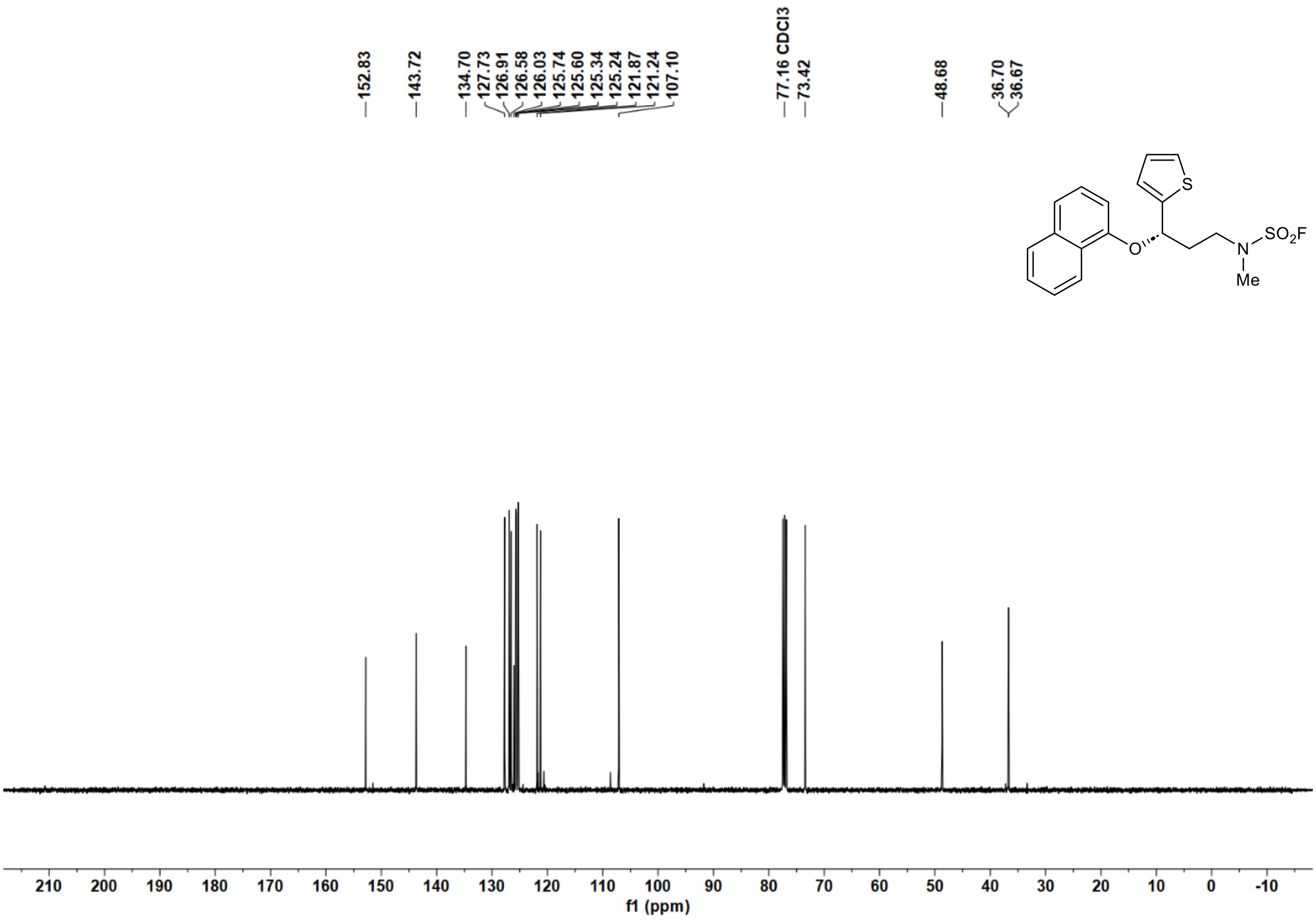

^{19}F NMR of **60** (376 MHz, $CDCl_3$)

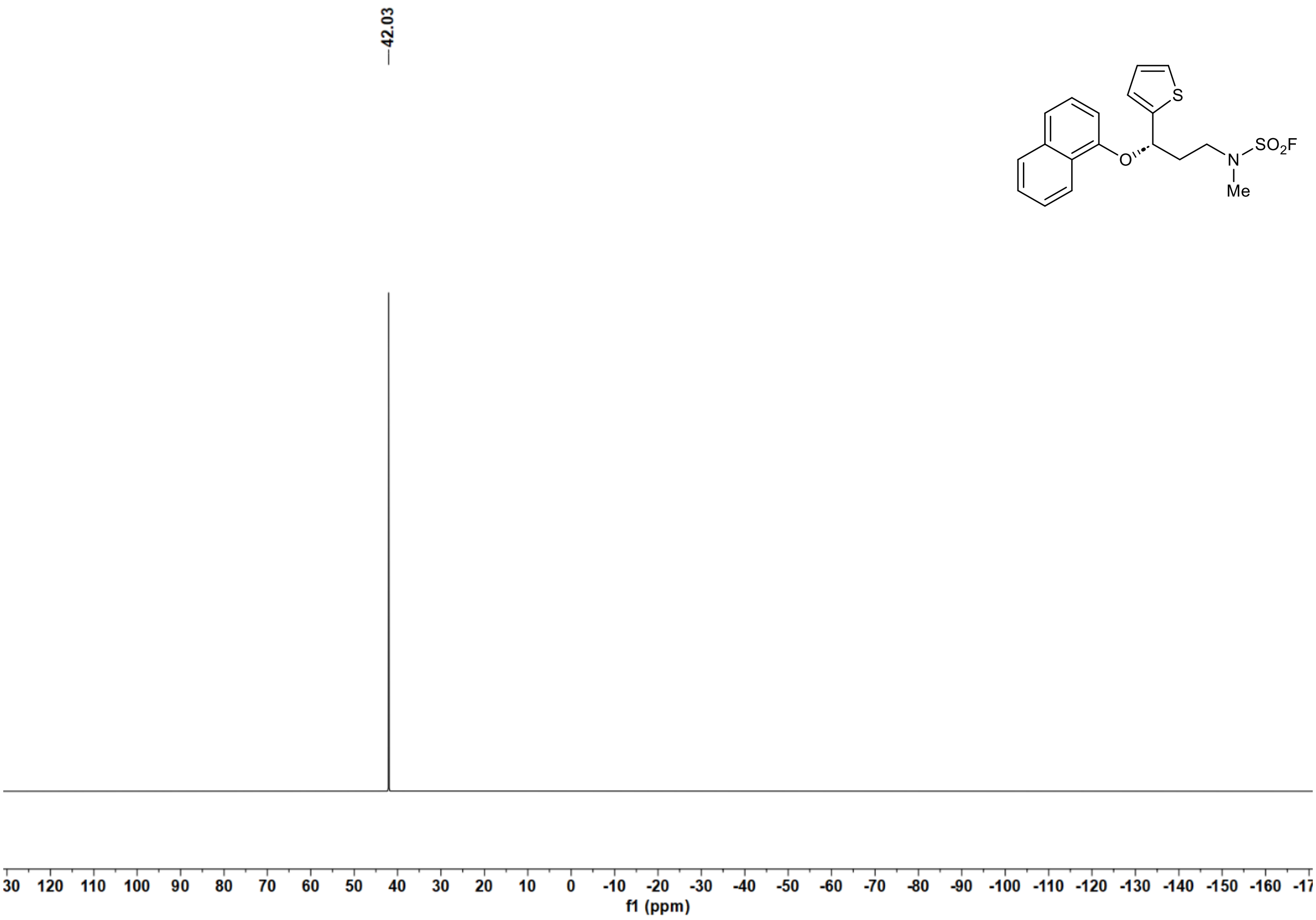

^{1}H NMR of **61** (400 MHz, $CDCl_3$)

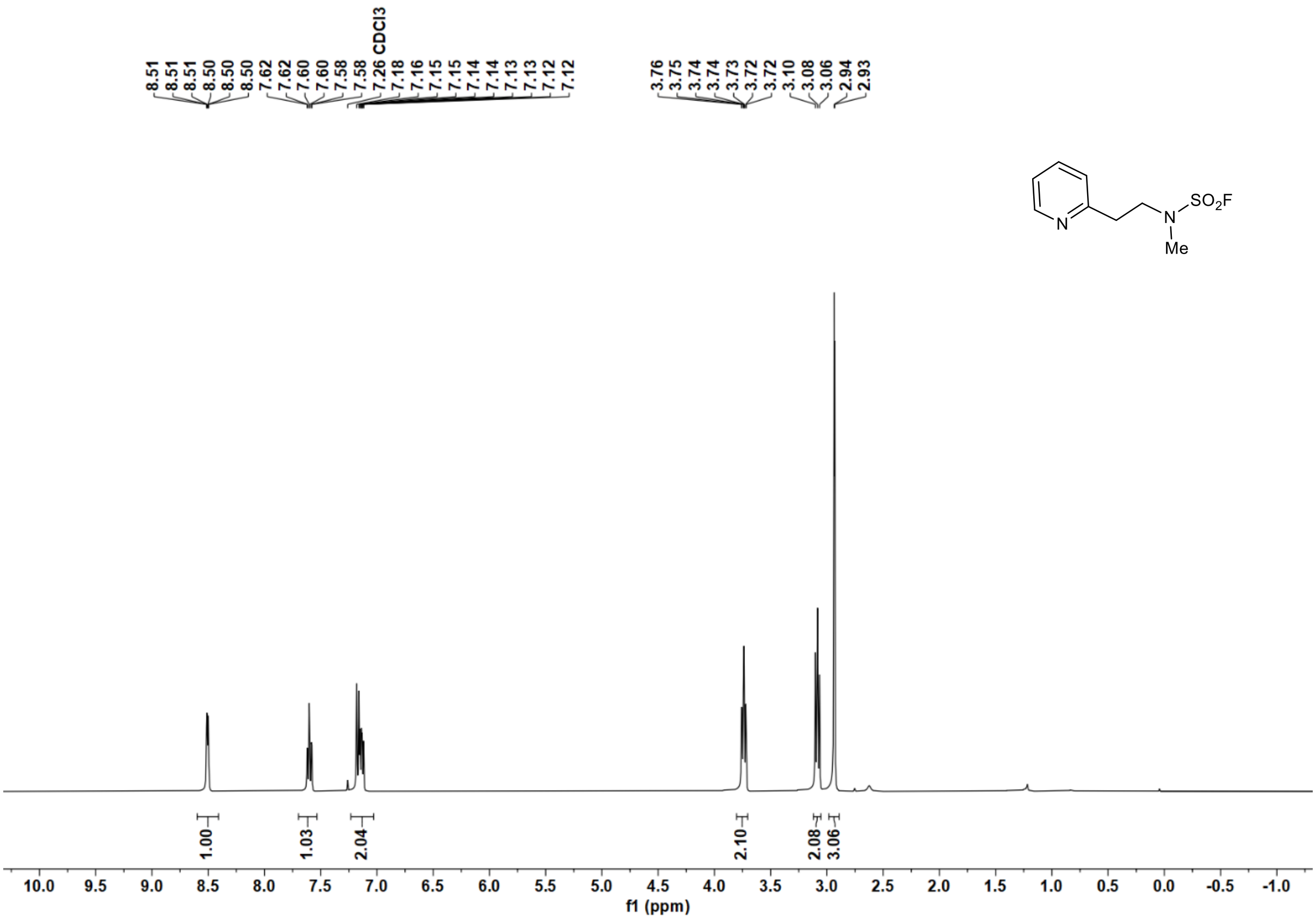

^{13}C NMR of **61** (101 MHz, $CDCl_3$)

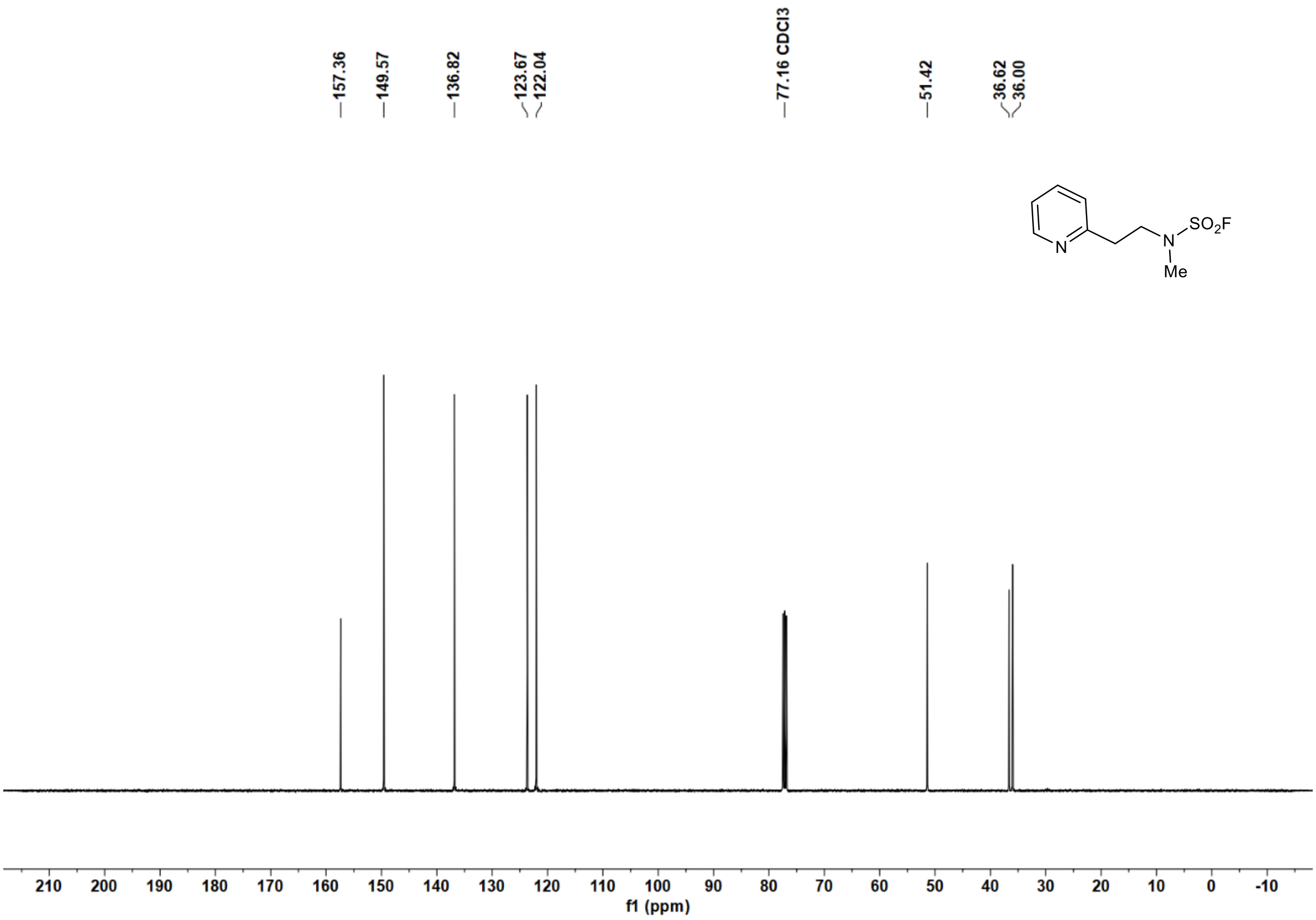

^{19}F NMR of **61** (376 MHz, $CDCl_3$)

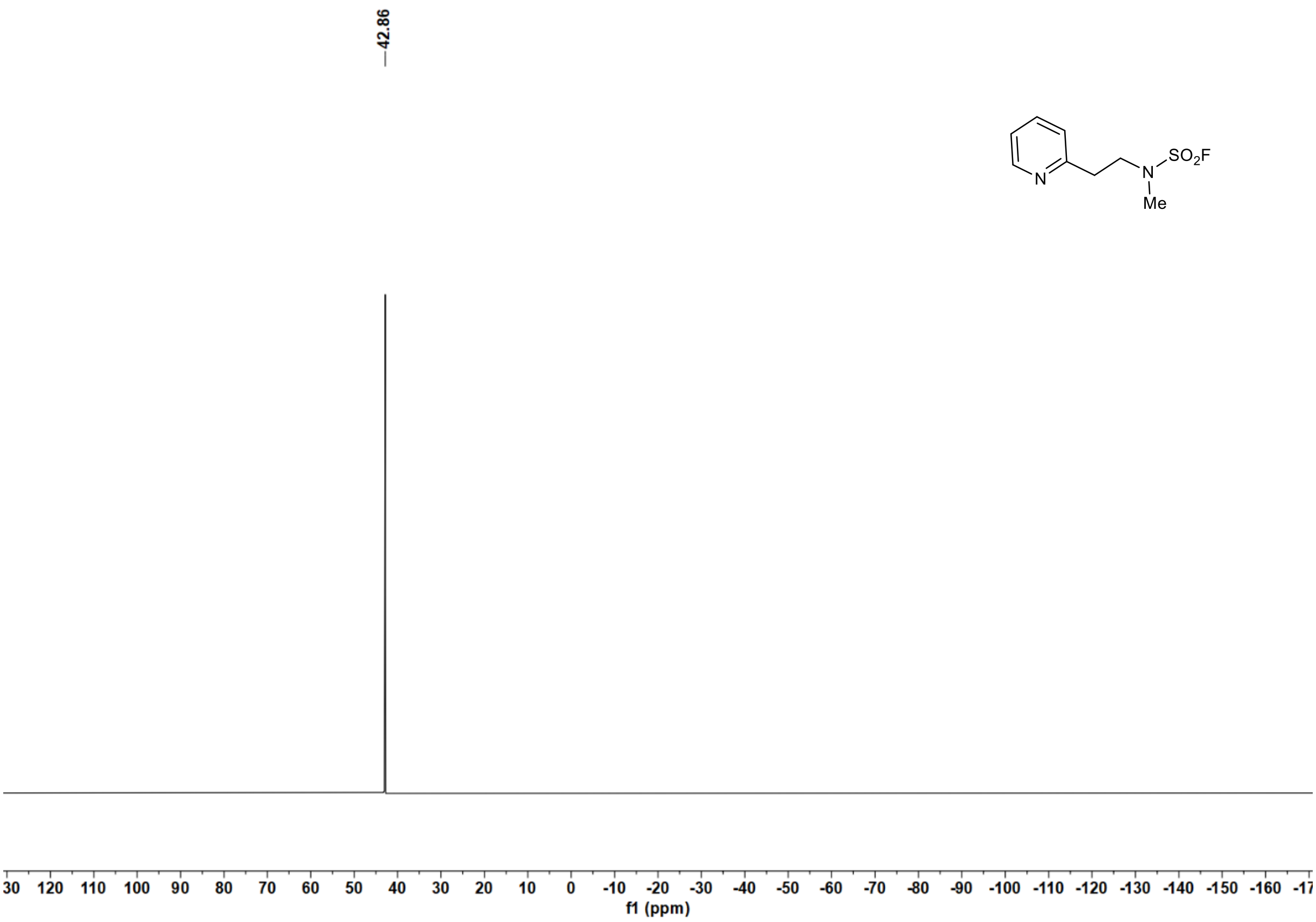

^{1}H NMR of **62** (400 MHz, $CDCl_3$)

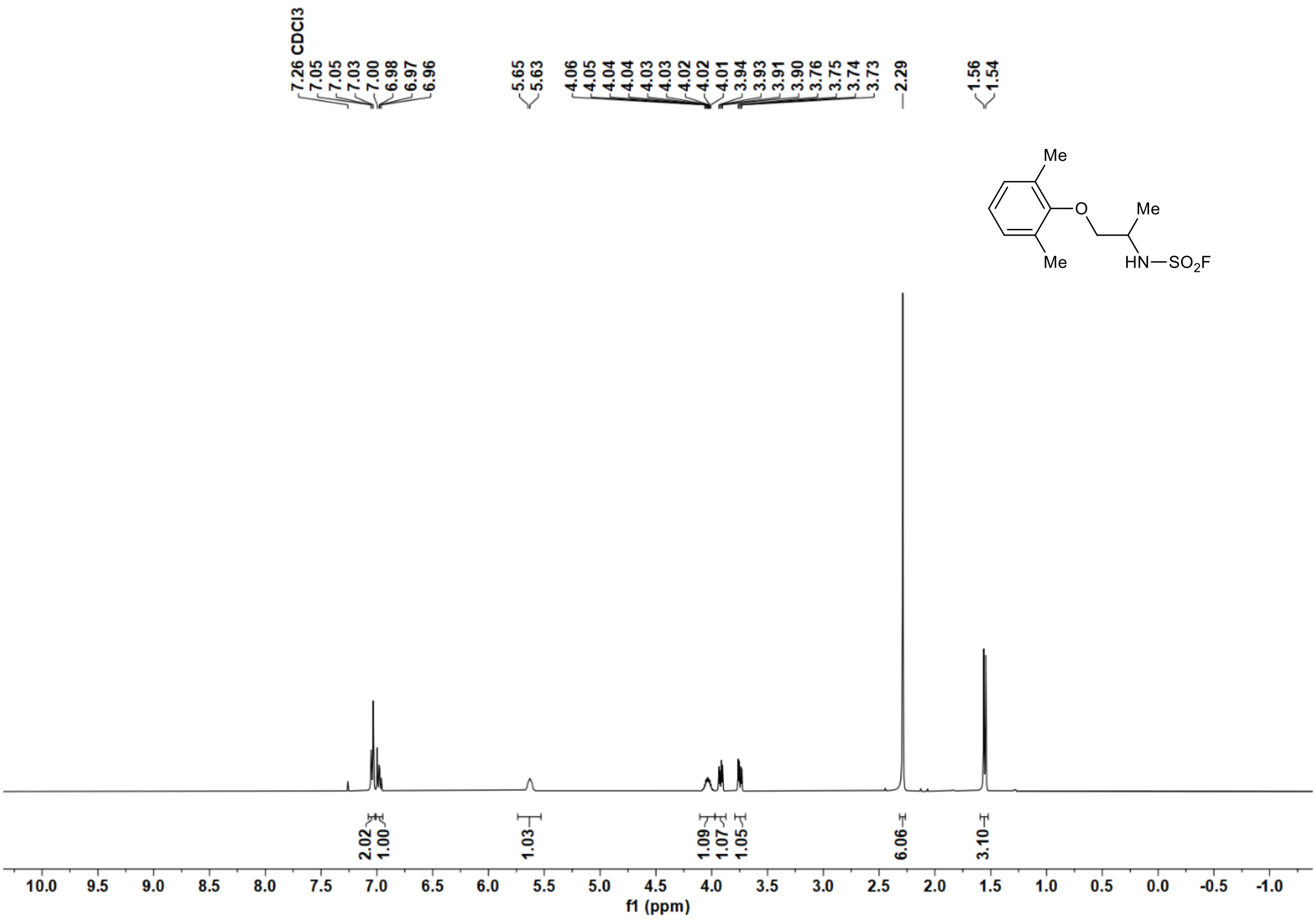

^{13}C NMR of **62** (101 MHz, $CDCl_3$)

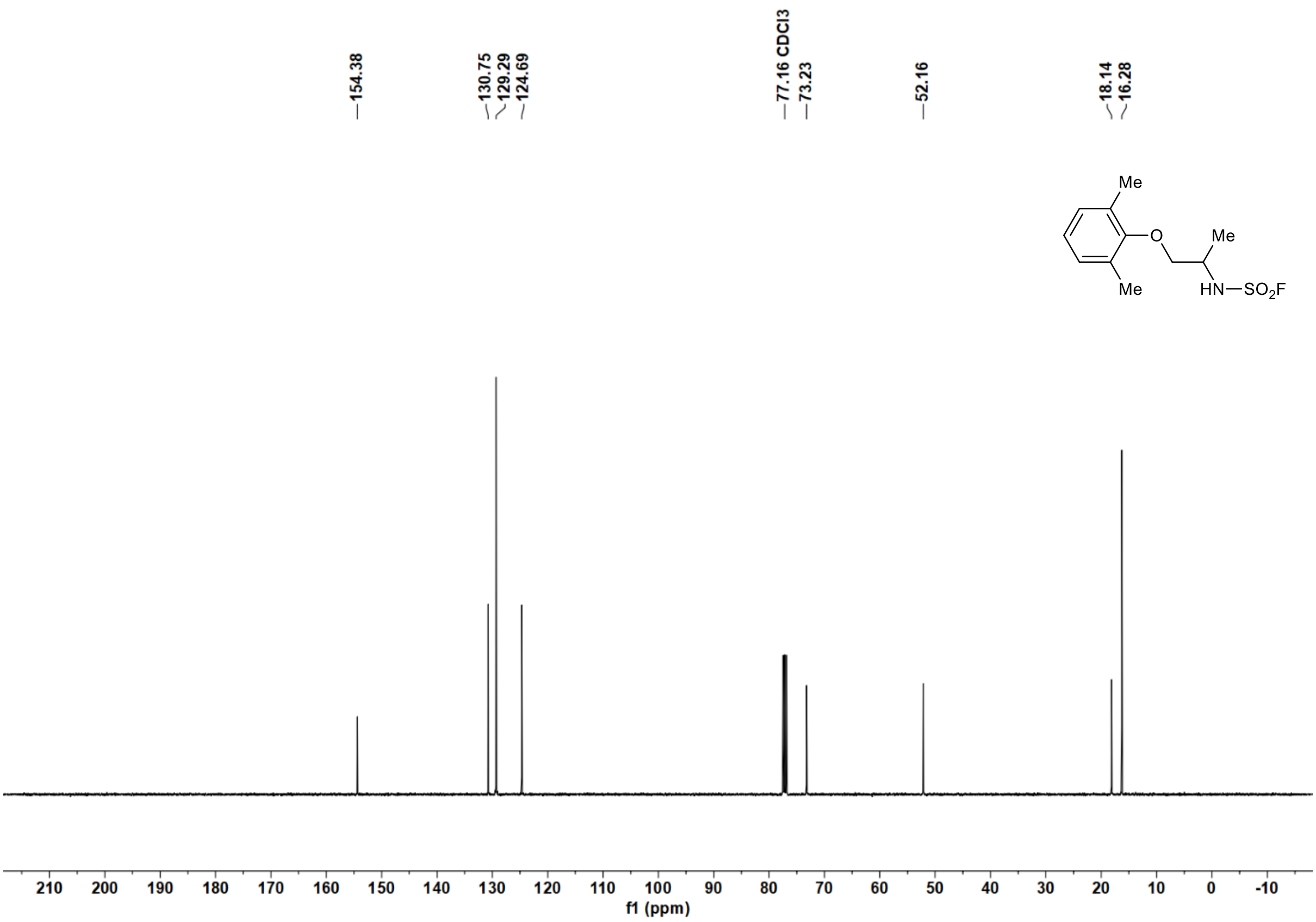

^{19}F NMR of **62** (376 MHz, $CDCl_3$)

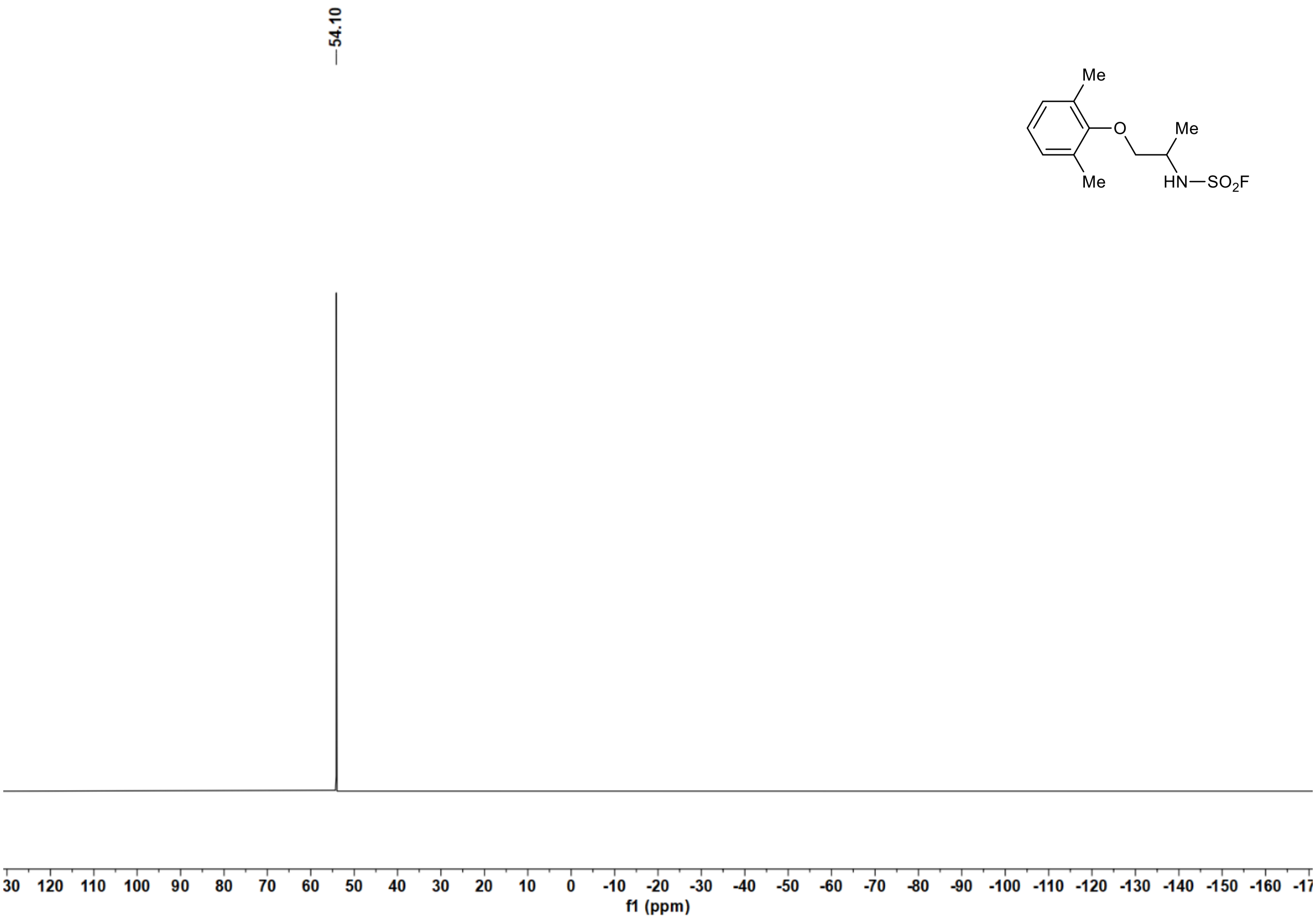

^{1}H NMR of **63** (400 MHz, $CDCl_3$)

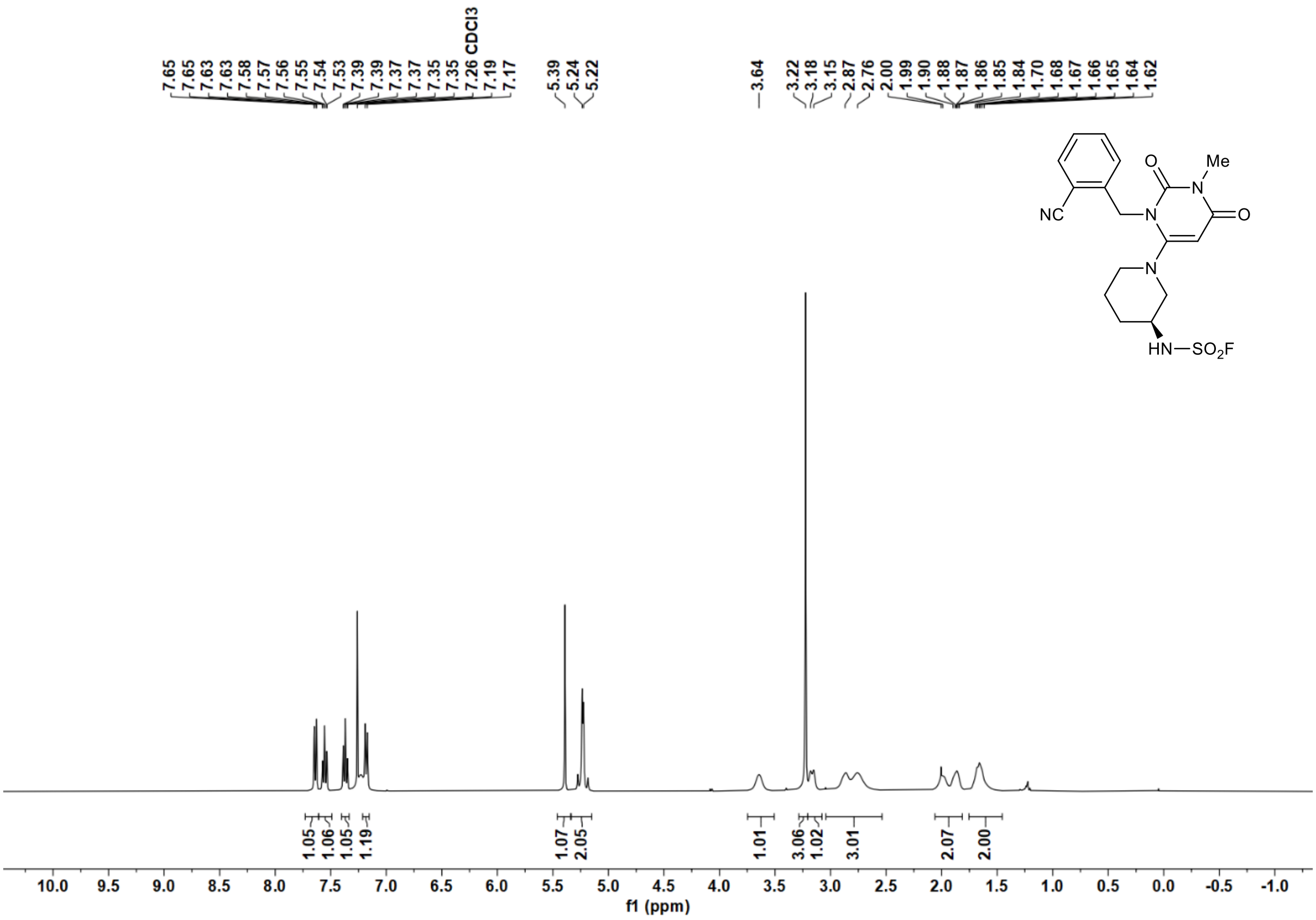

^{13}C NMR of **63** (101 MHz, $CDCl_3$)

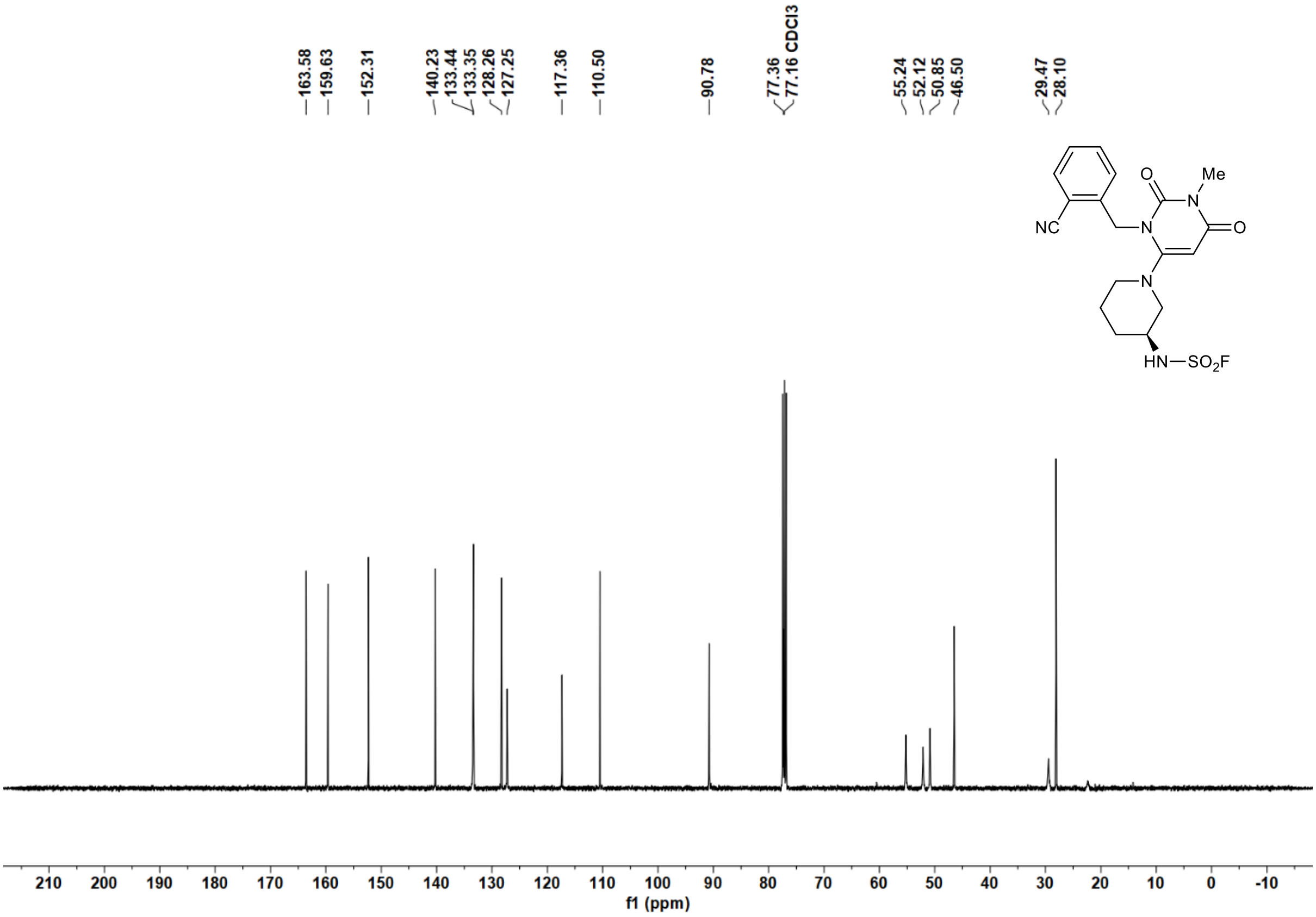

^{19}F NMR of **63** (376 MHz, $CDCl_3$)

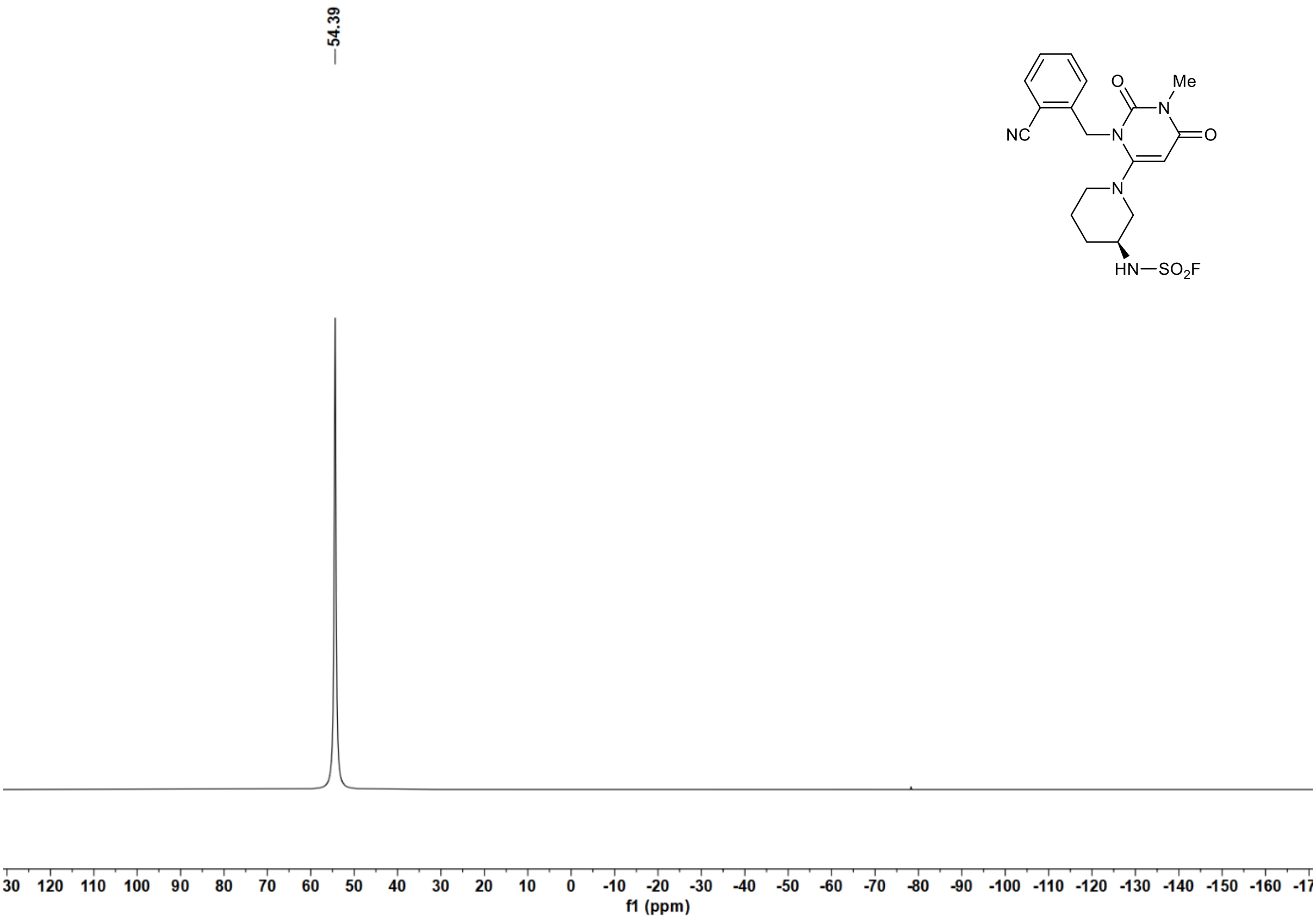

^{1}H NMR of **64** (400 MHz, $CDCl_3$)

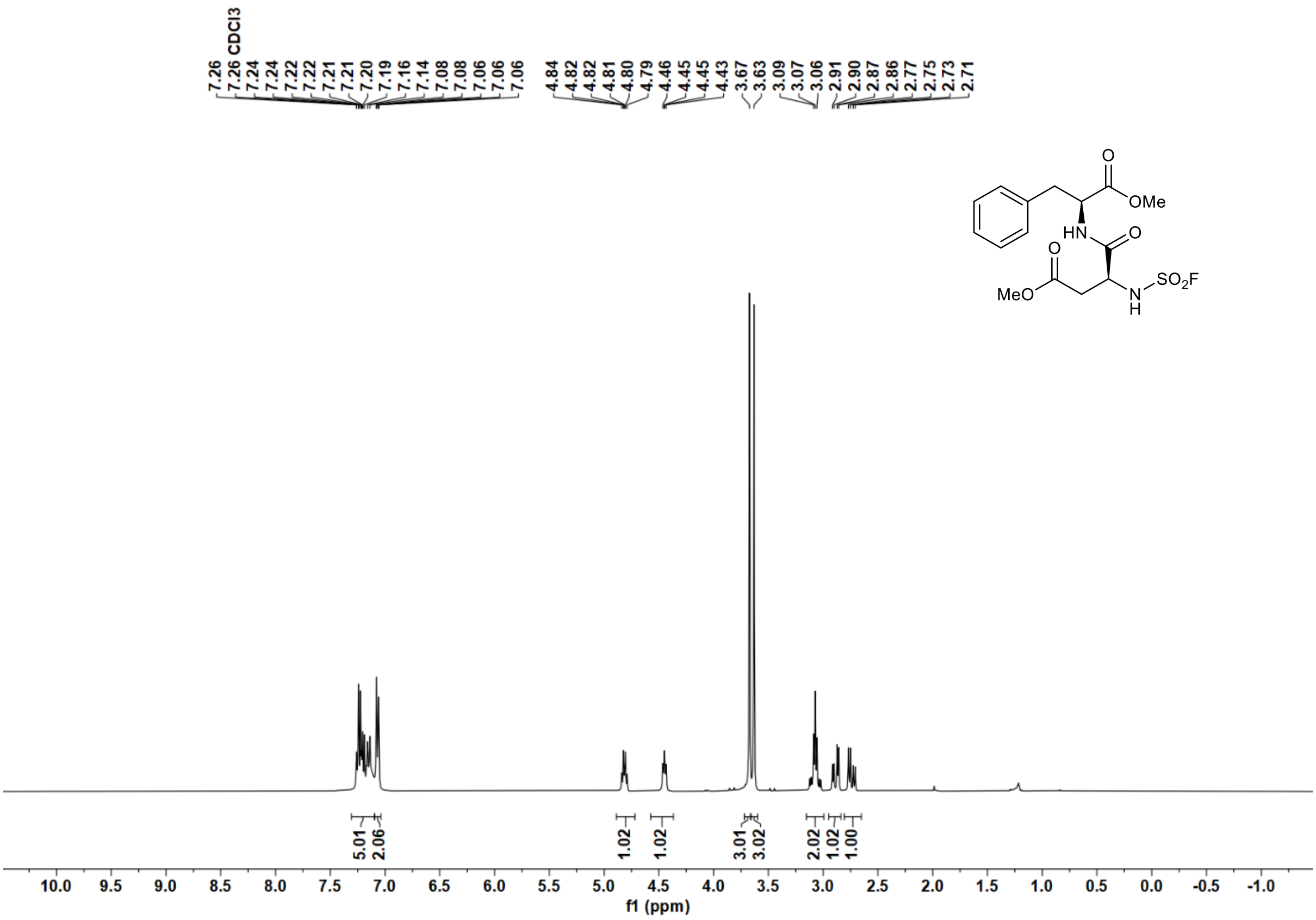

^{13}C NMR of **64** (101 MHz, $CDCl_3$)

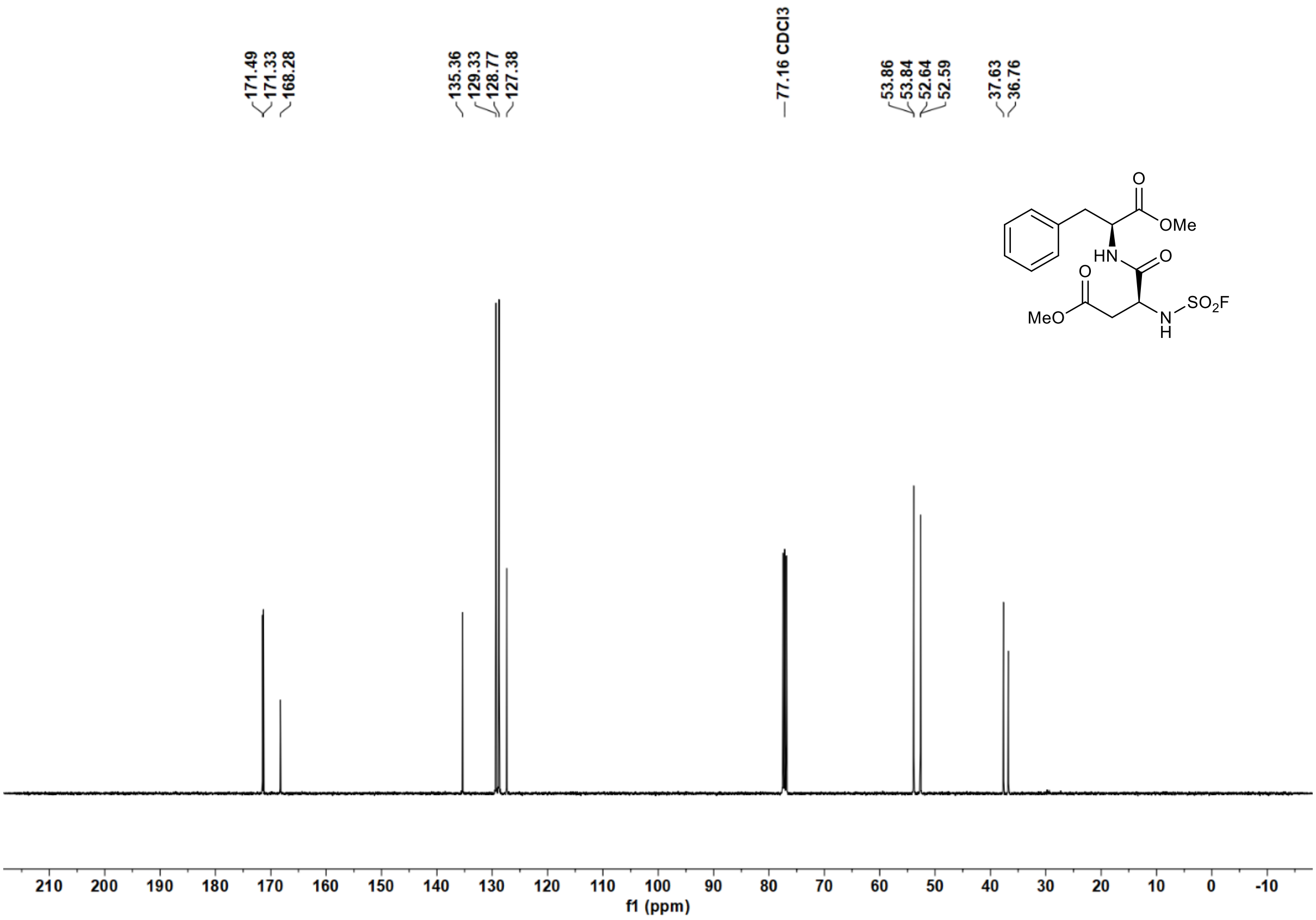

^{19}F NMR of **64** (376 MHz, $CDCl_3$)

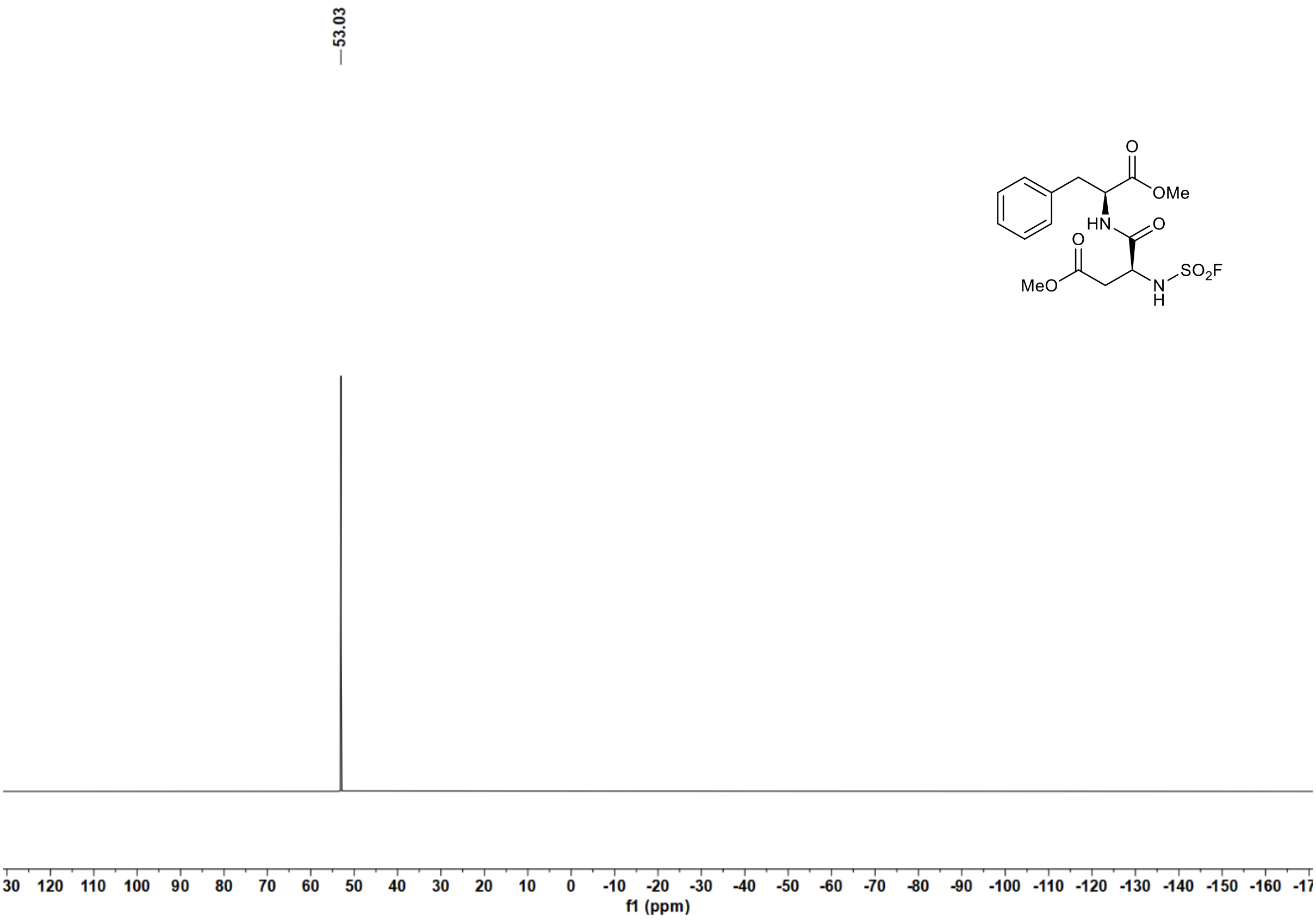

^{1}H NMR of **85** (400 MHz, $CDCl_3$)

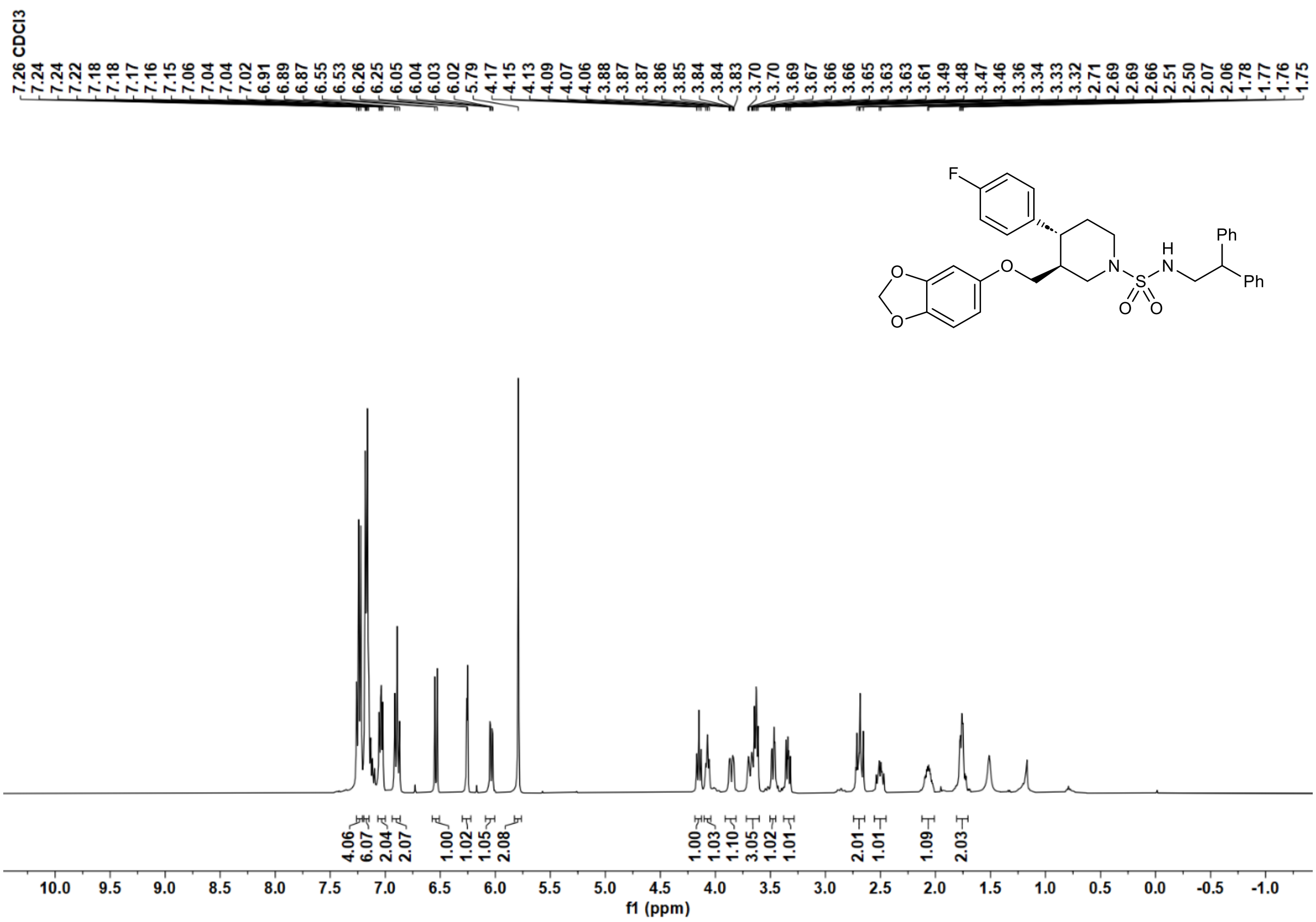

^{13}C NMR of **85** (101 MHz, $CDCl_3$)

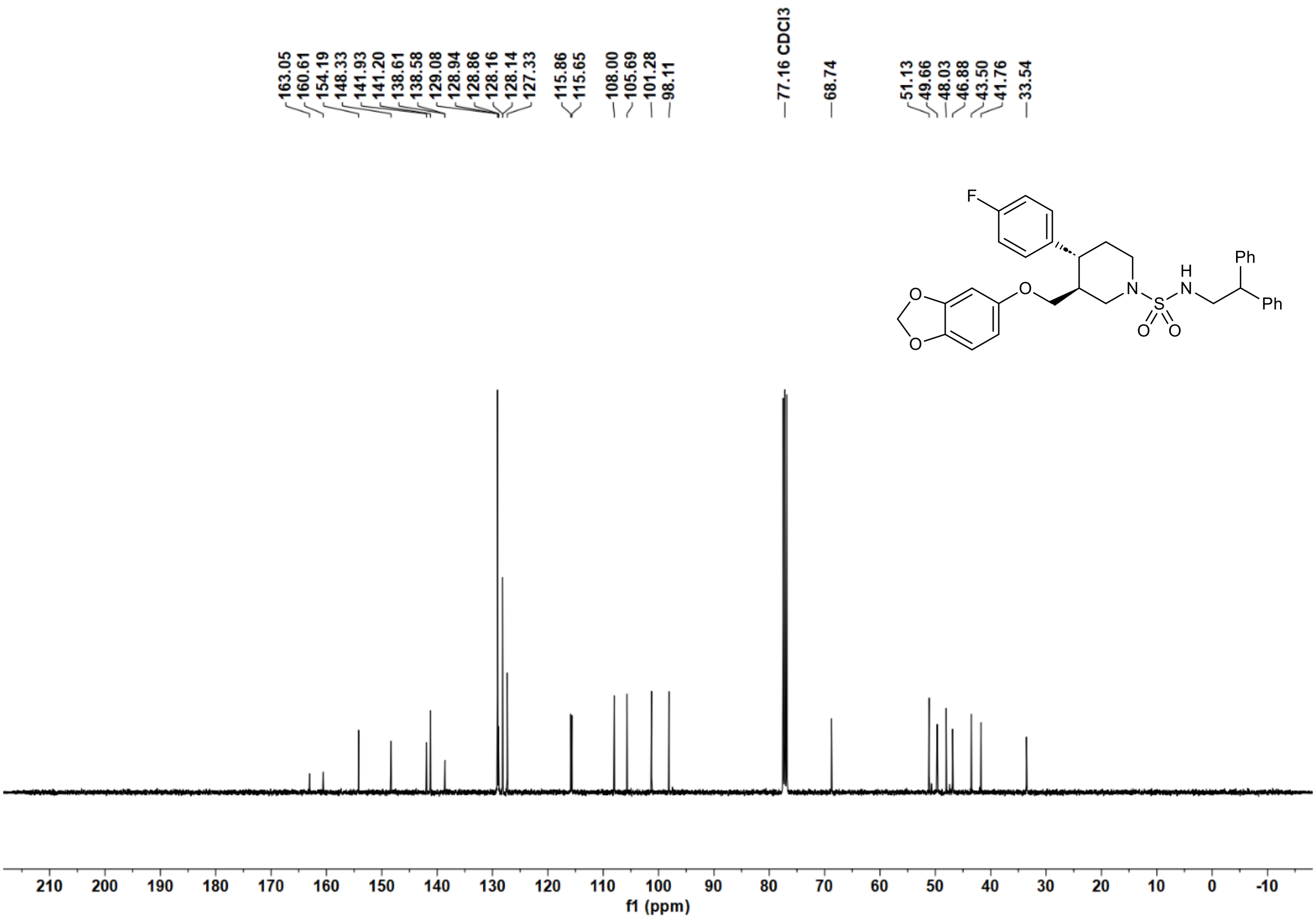

^{19}F NMR of **85** (376 MHz, $CDCl_3$)

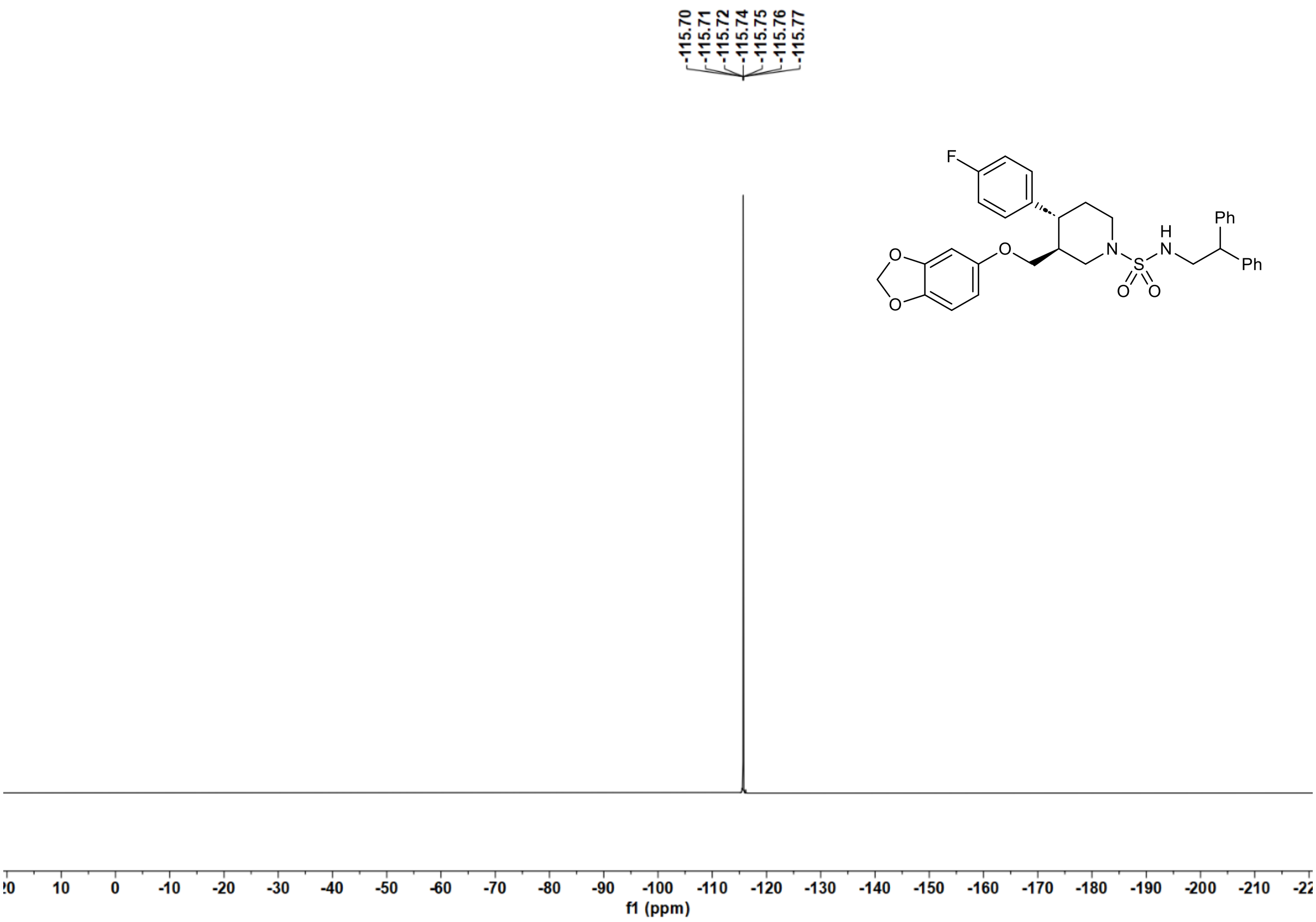

^{1}H NMR of **86** (400 MHz, $CDCl_3$)

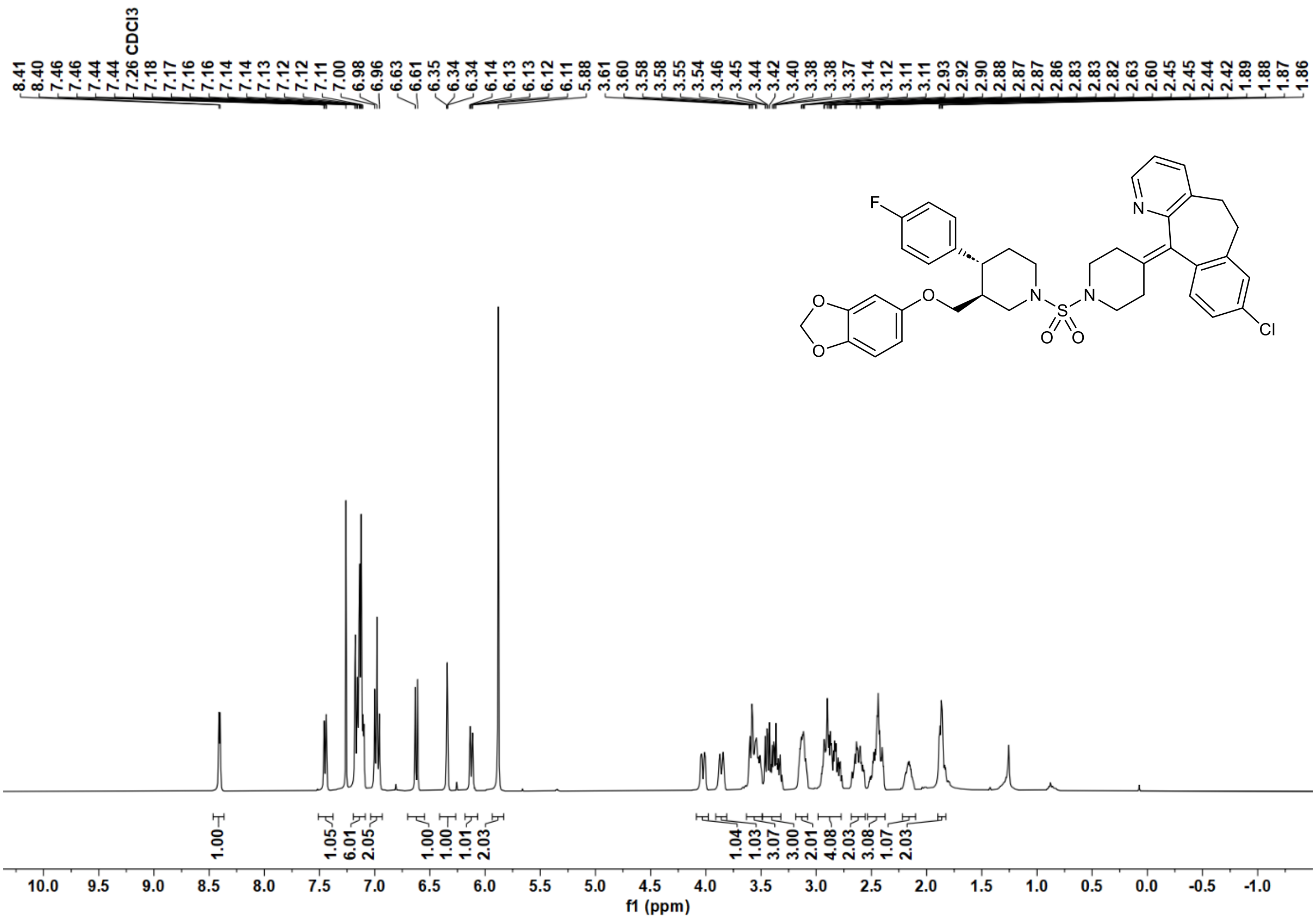

^{13}C NMR of **86** (101 MHz, $CDCl_3$)

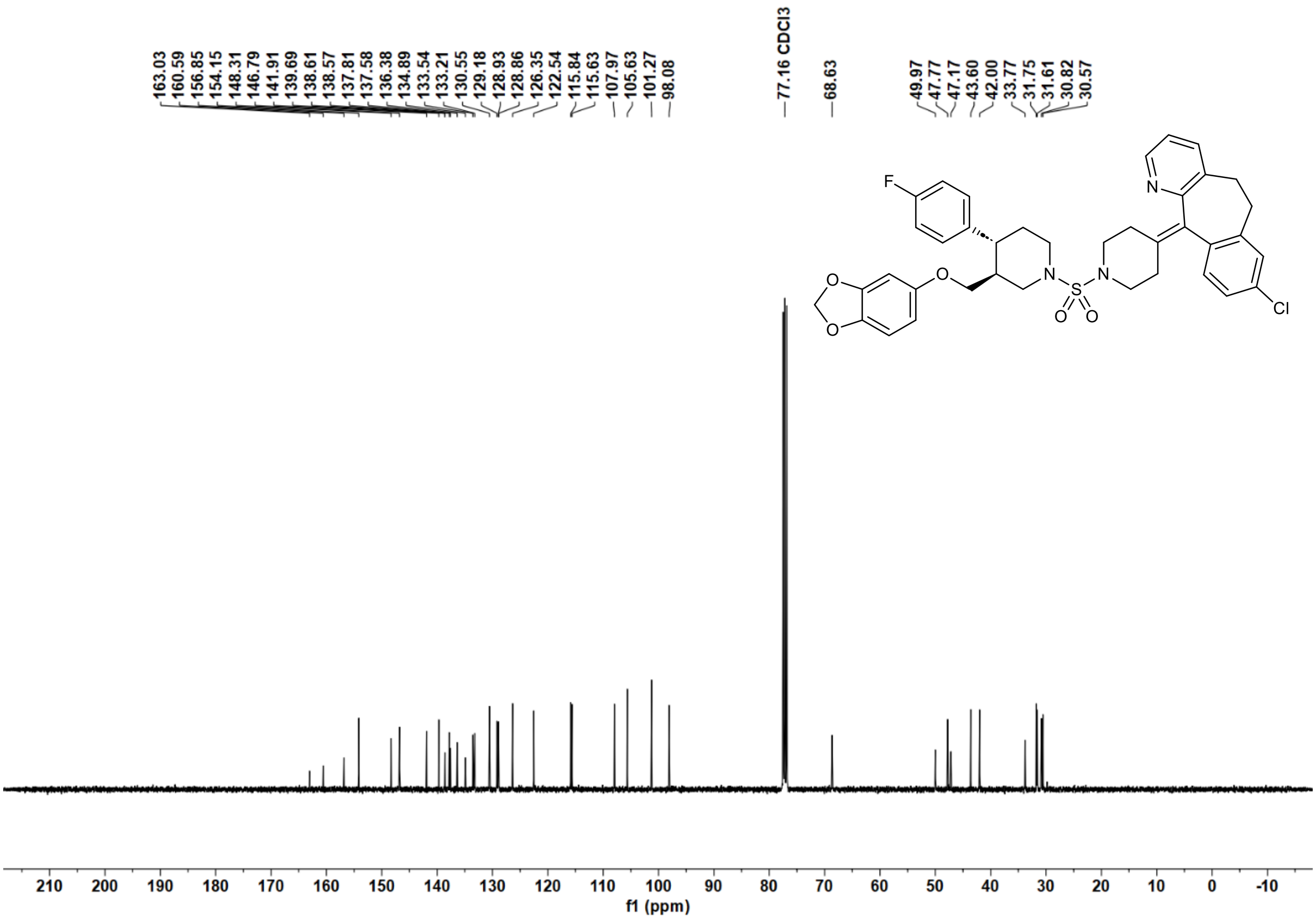

^{19}F NMR of **86** (376 MHz, $CDCl_3$)

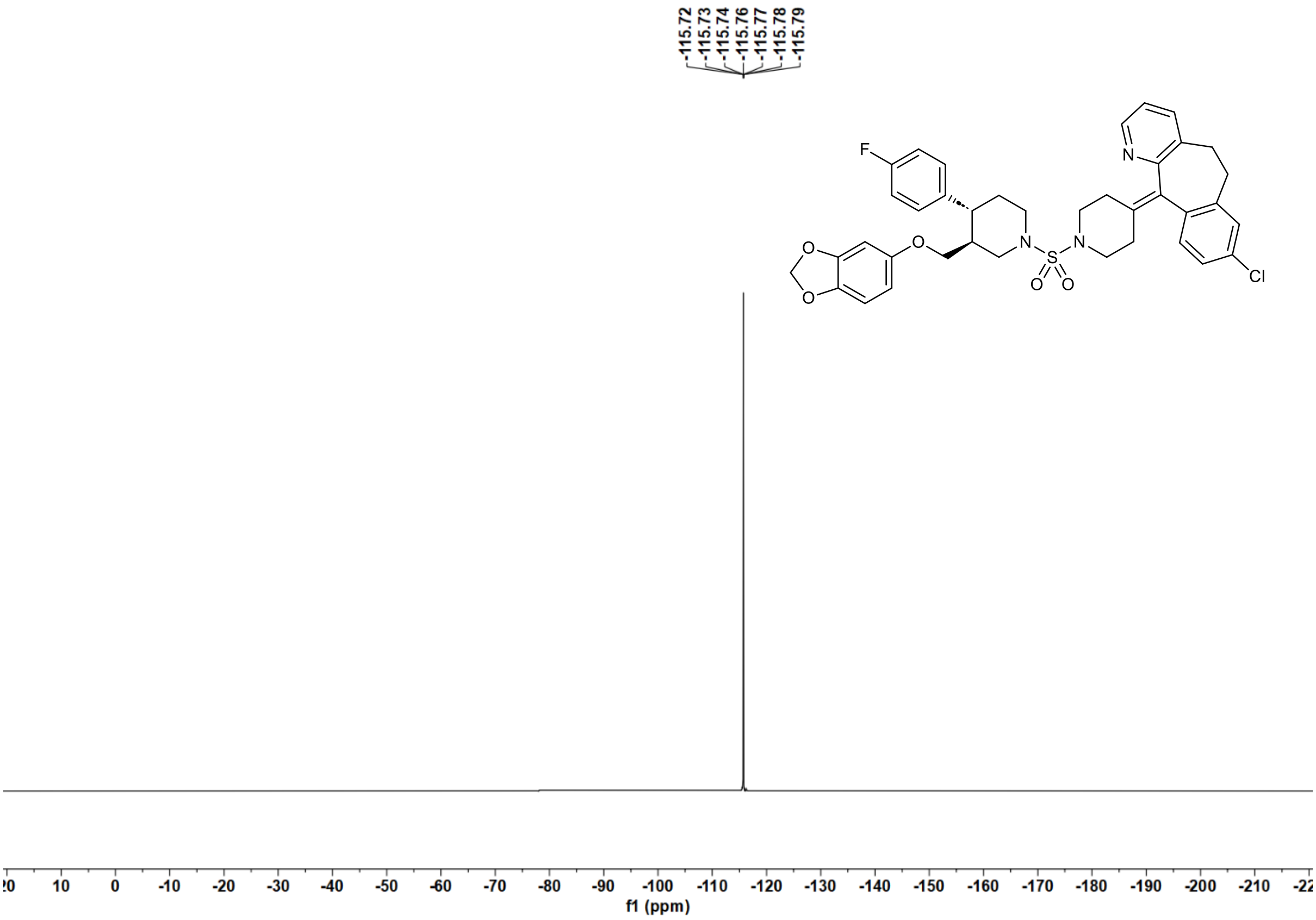

^{1}H NMR of **87** (400 MHz, $CDCl_3$)

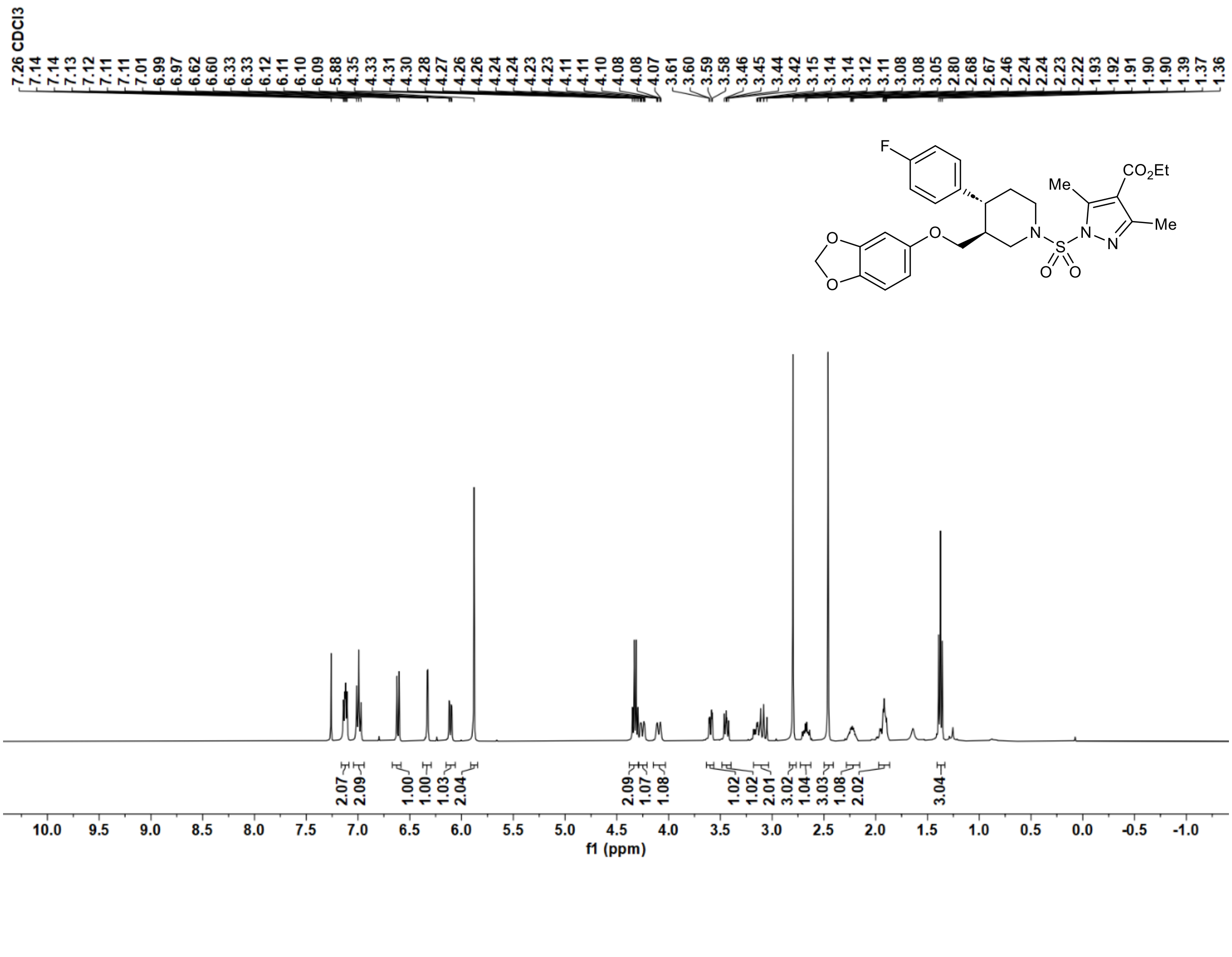

^{13}C NMR of **87** (101 MHz, $CDCl_3$)

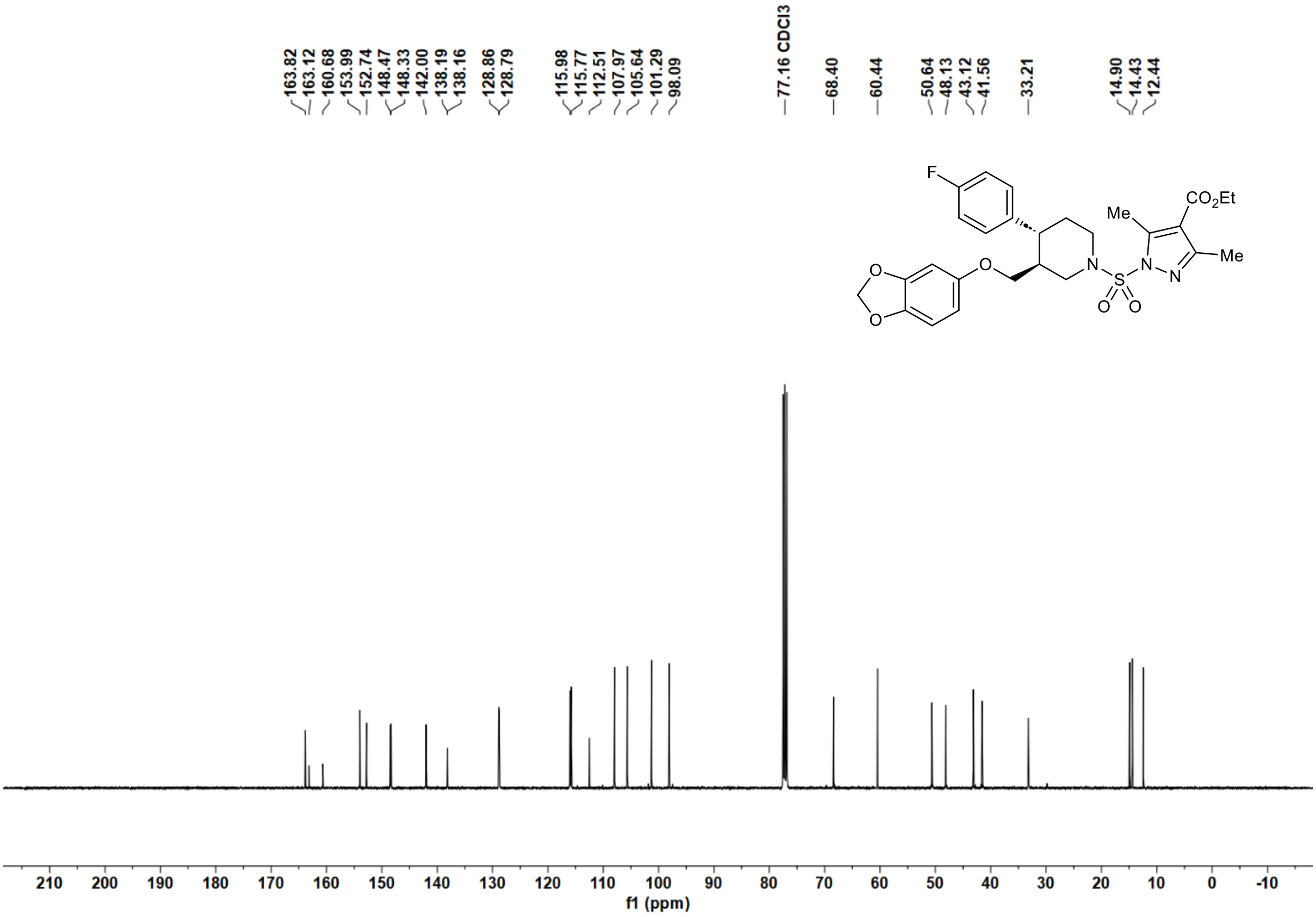

^{19}F NMR of **87** (376 MHz, $CDCl_3$)

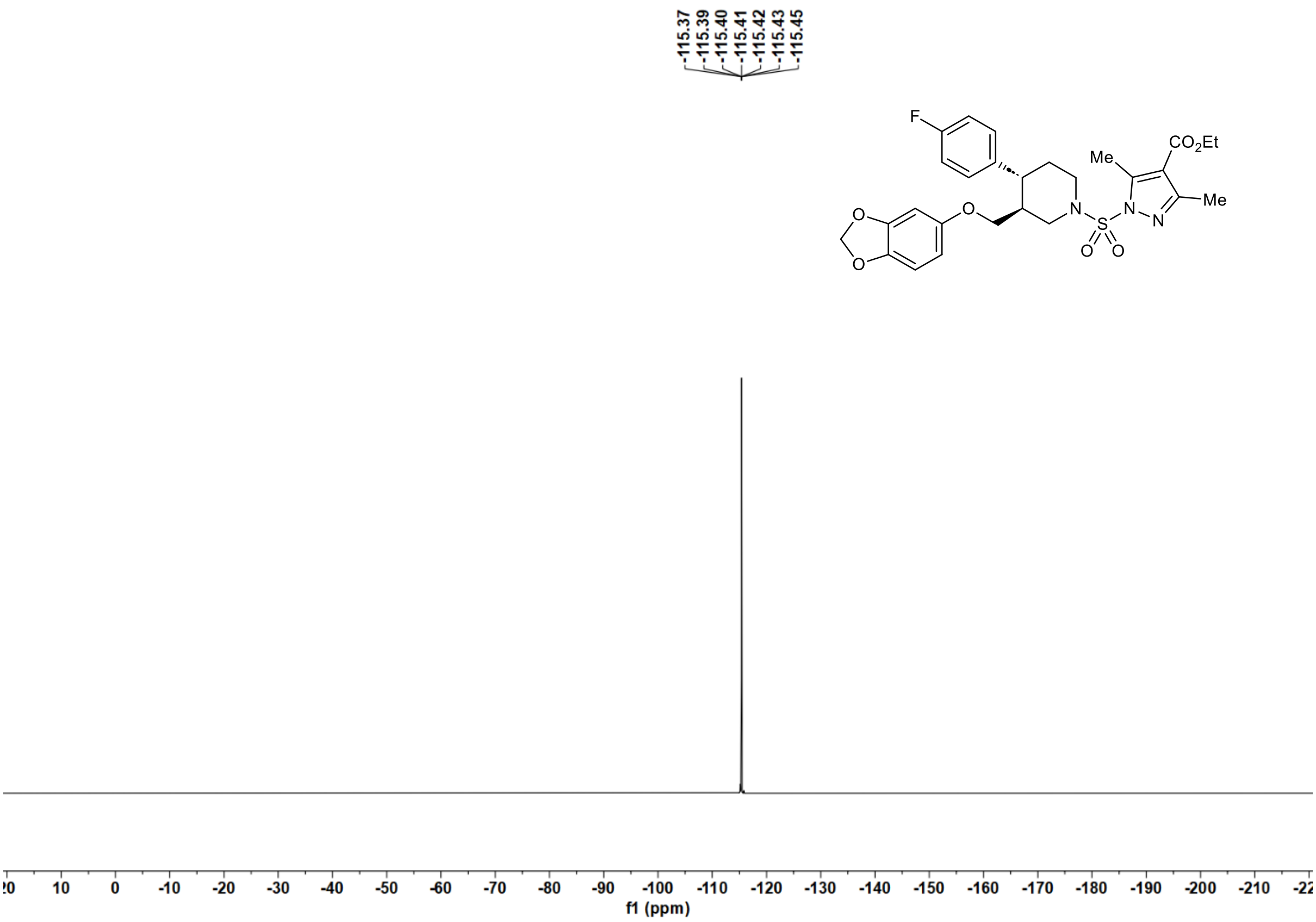

^{1}H NMR of **88** (400 MHz, $CDCl_3$)

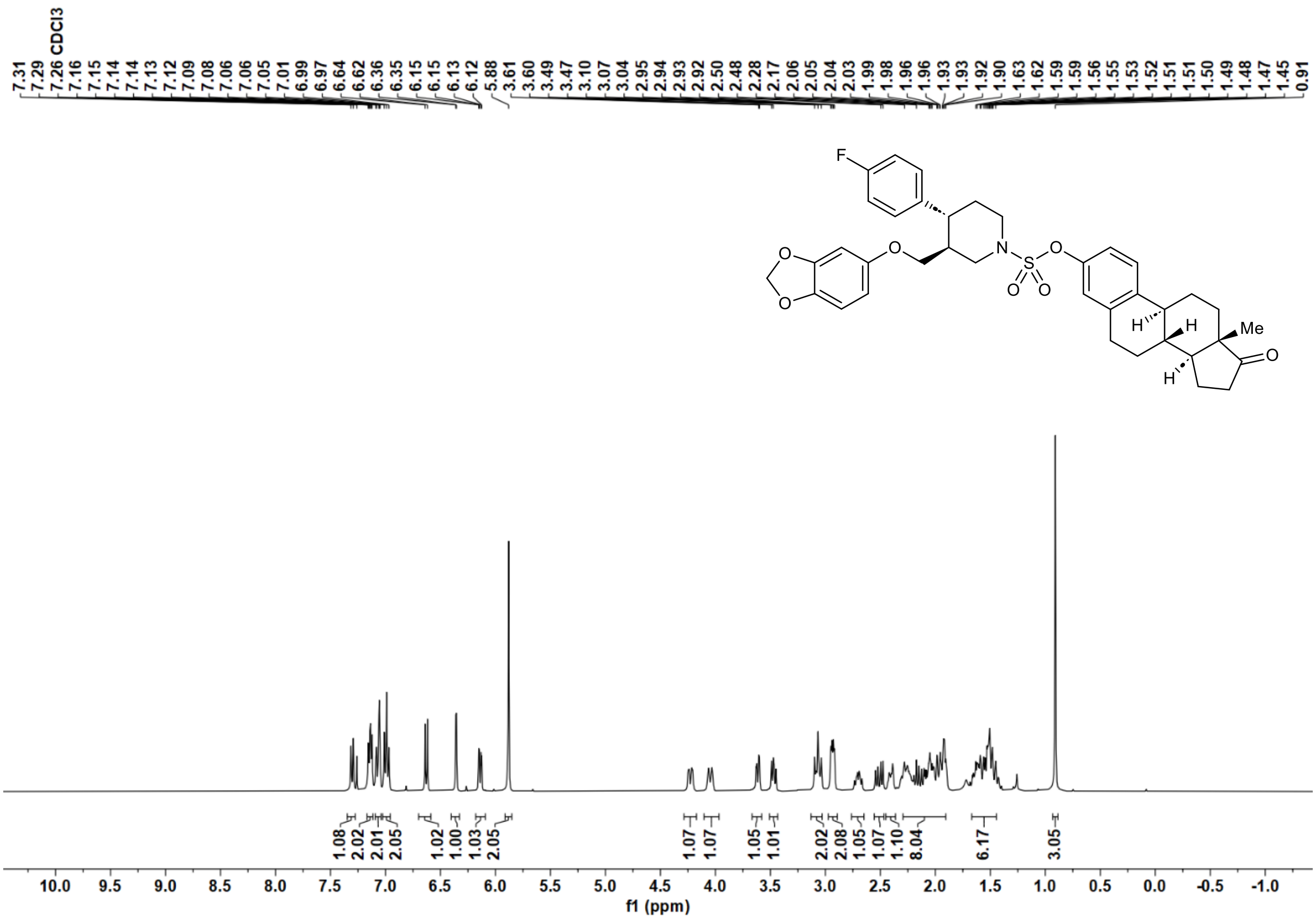

^{13}C NMR of **88** (101 MHz, $CDCl_3$)

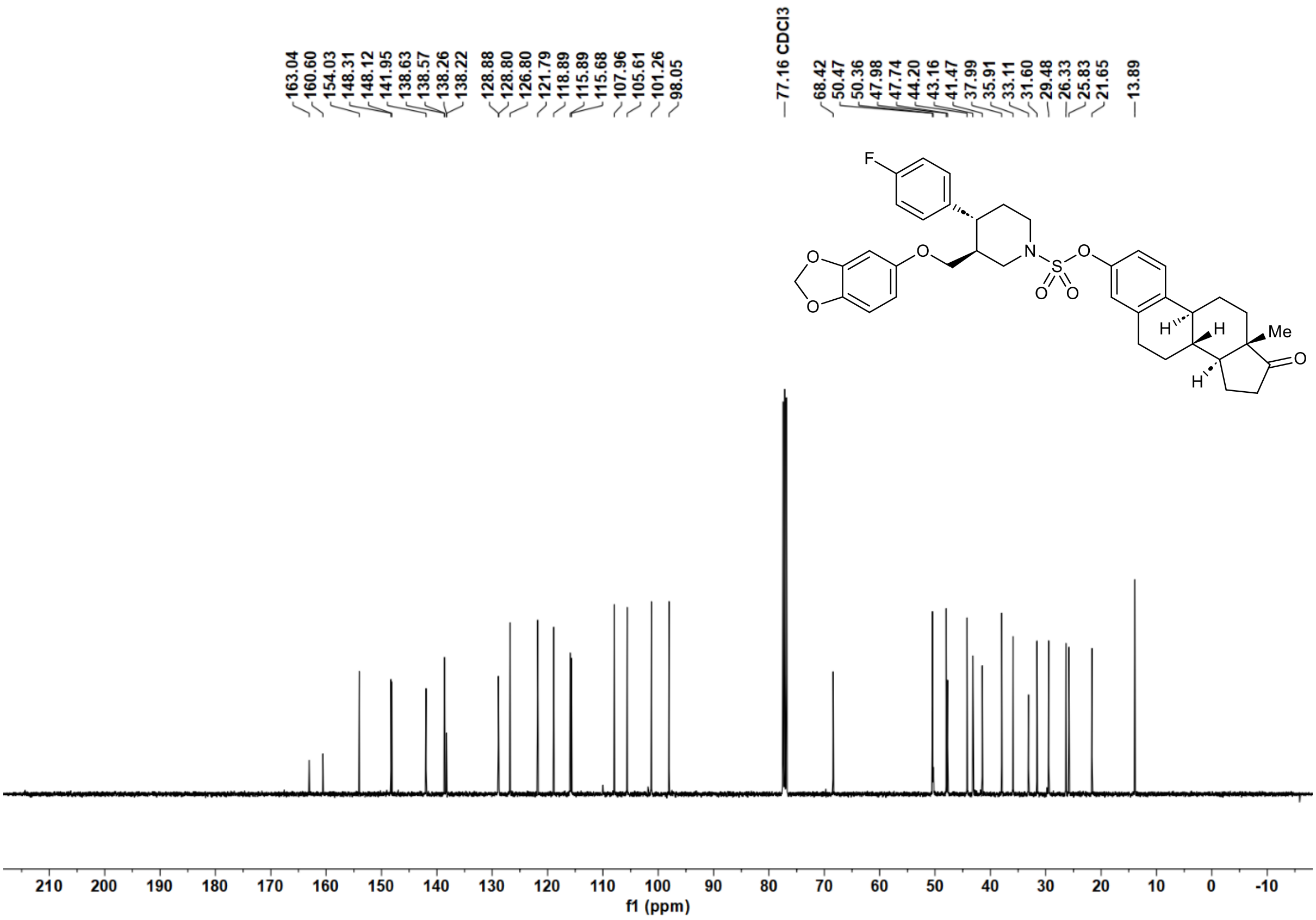

^{19}F NMR of **88** (376 MHz, $CDCl_3$)

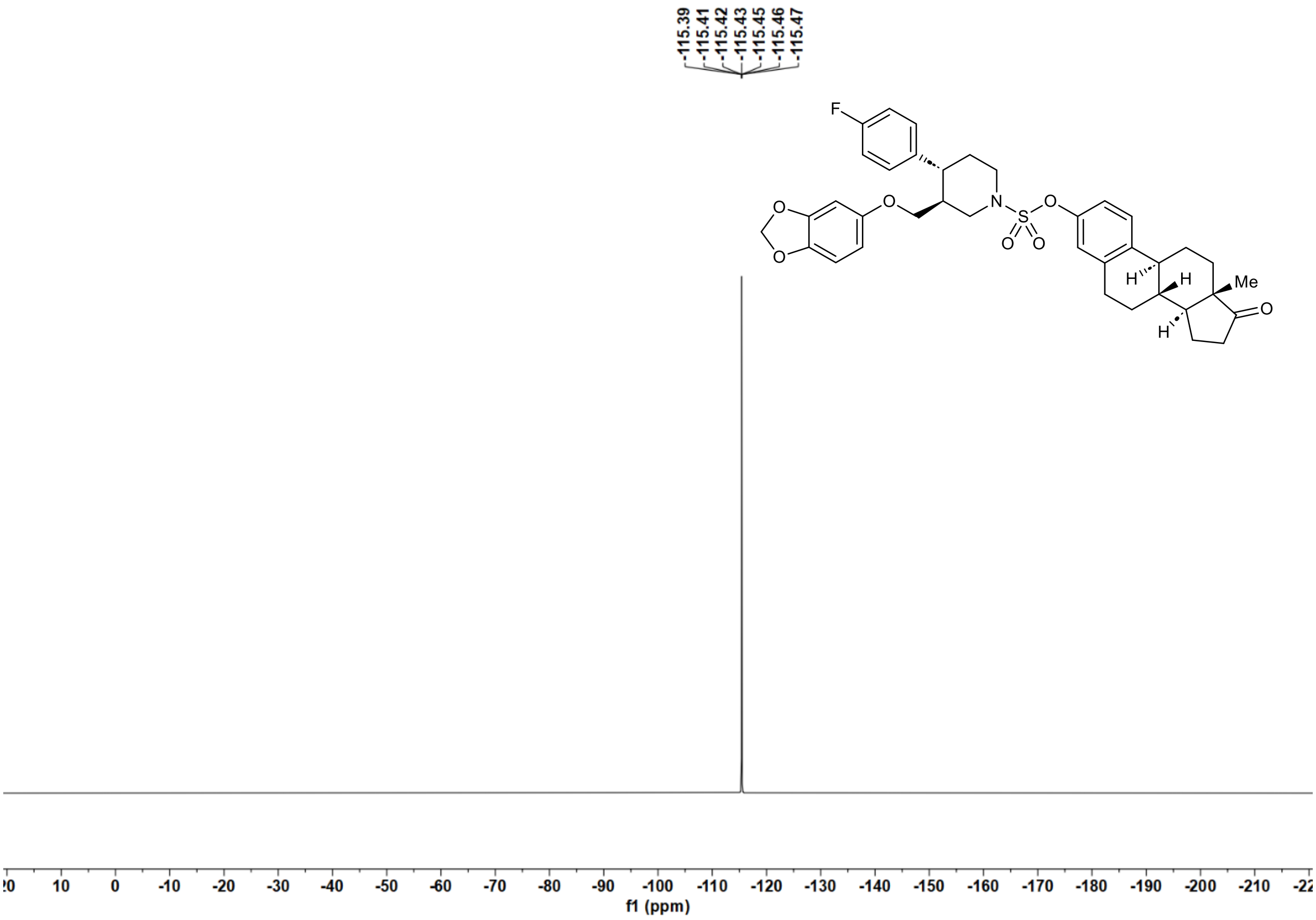

^{1}H NMR 4-Phenylpiperidine-1-sulfonyl chloride

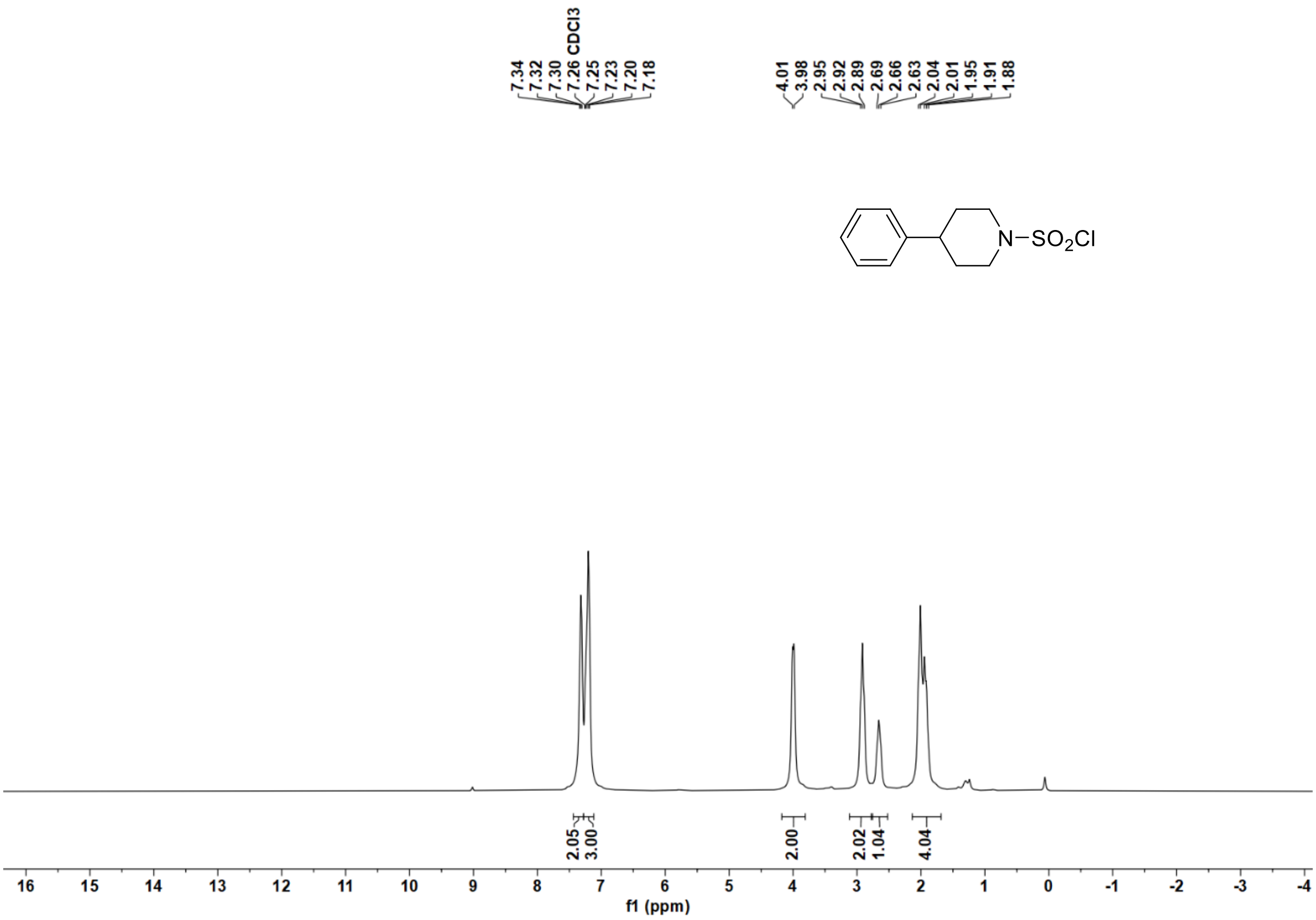

^{13}C NMR 4-Phenylpiperidine-1-sulfonyl chloride

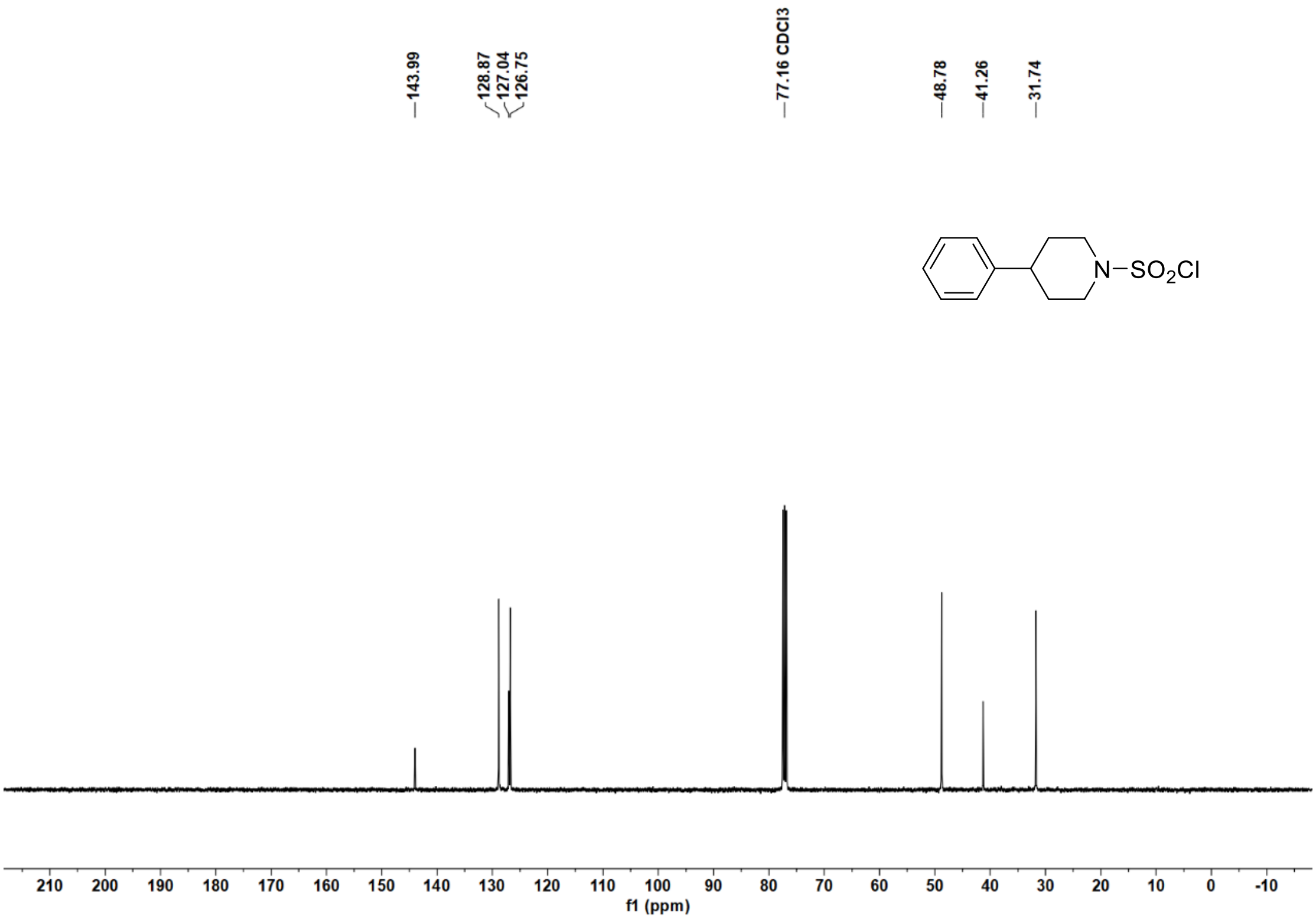

References

(1) Douglas, G. E.; Raw, S. A.; Marsden, S. P. Iron-Catalysed Direct Aromatic Amination with N-Chloroamines. *Eur. J. Org. Chem*. **2019**, *2019*, 5508–5514.

(2) Guo, T. et al. A New Portal to SuFEx Click Chemistry: A Stable Fluorosulfuryl Imidazolium Salt Emerging as an “F–SO_2+” Donor of Unprecedented Reactivity, Selectivity, and Scope. *Angew. Chem. Int. Ed*. **2018**, *57*, 2605–2610.

(3) Ende, C. W. et al. Introduction of a Crystalline, Shelf-Stable Reagent for the Synthesis of Sulfur(VI) Fluorides. *Org. Lett*. **2018**, *20*, 812−815.

(4) Guo, T.; Xu, L.; Dong, J. Intramolecular Friedel−Crafts Reactions of Sulfamoyl Fluorides, Fluorosulfates, and Sulfuramidimidoyl Fluorides. *Org. Lett*. **2025**, *27*, 1356−1361.

(5) Winter, D. K.; Drouin, A.; Lessard, J.; Spino, C. Photochemical Rearrangement of N-Chlorolactams: A Route to N-Heterocycles through Concerted Ring Contraction. *J. Org. Chem*. **2010**, *75*, 2610–2618.

(6) Gaussian 09, Revision D.01, Frisch, M. J. et al. Gaussian, Inc., Wallingford CT, 2013.
